# Revision of *Belvosia* Robineau-Desvoidy (Diptera, Tachinidae) and 33 new species from Area de Conservación Guanacaste in northwestern Costa Rica with a key to known North and Mesoamerican species

**DOI:** 10.3897/BDJ.11.e103667

**Published:** 2023-06-30

**Authors:** AJ Fleming, Norman Woodley, M. Alex Smith, Winnie Hallwachs, Daniel H Janzen

**Affiliations:** 1 Agriculture Agri-Food Canada, Ottawa, Canada Agriculture Agri-Food Canada Ottawa Canada; 2 ARS USDA, Arizona, United States of America ARS USDA Arizona United States of America; 3 University of Guelph, Guelph, Canada University of Guelph Guelph Canada; 4 Department of Biology, University of Pennsylvania, Philadelphia, Philadelphia, Pennsylvania, United States of America Department of Biology, University of Pennsylvania, Philadelphia Philadelphia, Pennsylvania United States of America

**Keywords:** Caterpillar, tropical, Goniini, parasitoid, fly, rain forest, dry forest, cloud forest, Area de Conservación Guanacaste, ACG

## Abstract

**Background:**

This revision is part of a continuing series of taxonomic work aimed at the description of new taxa and the redescription of known taxa of the Tachinidae of Area de Conservación Guanacaste in northwestern Costa Rica. Here we describe 33 new species in the genus *Belvosia* Robineau-Desvoidy, 1830 (Diptera: Tachinidae). All species described here were reared from this ongoing inventory of wild-caught caterpillars spanning a variety of families (Lepidoptera: Erebidae, Eupterotidae, Noctuidae, Notodontidae, Saturniidae, and Sphingidae). We provide a morphological description of each species with limited information on life history, molecular data, and photographic documentation. In addition to the new species, the authors provide a redescription of the genus *Belvosia*, as well as provide a key to the identification of the species present in the Meso- and North-American fauna.

**New information:**

The following 33 new species of *Belvosia* Robineau-Desvoidy, 1830, all authored by Fleming & Woodley, are described: *Belvosiaadrianguadamuzi* Fleming & Woodley **sp. n.**, *Belvosiaanacarballoae* Fleming & Woodley **sp. n.**, *Belvosiaangelhernandezi* Fleming & Woodley **sp. n.**, *Belvosiabrigittevilchezae* Fleming & Woodley **sp. n.**, *Belvosiaalixtomoragai* Fleming & Woodley **sp. n.**, *Belvosiacarolinacanoae* Fleming & Woodley **sp. n.**, *Belvosiaciriloumanai* Fleming & Woodley **sp. n.**, *Belvosiadiniamartinezae* Fleming & Woodley **sp. n.**, *Belvosiaduniagarciae* Fleming & Woodley **sp. n.**, *Belvosiaduvalierbricenoi* Fleming & Woodley **sp. n.**, *Belvosiaeldaarayae* Fleming & Woodley **sp. n.**, *Belvosiaeliethcantillanoae* Fleming & Woodley **sp. n.**, *Belvosiafreddyquesadai* Fleming & Woodley **sp. n.**, *Belvosiagloriasihezarae* Fleming & Woodley **sp. n.**, *Belvosiaguillermopereirai* Fleming & Woodley **sp. n.**, *Belvosiaharryramirezi* Fleming & Woodley **sp. n.**, *Belvosiahazelcambroneroae* Fleming & Woodley **sp. n.**, *Belvosiajorgehernandezi* Fleming & Woodley **sp. n.**, *Belvosiajosecortezi* Fleming & Woodley **sp. n.**, *Belvosiajoseperezi* Fleming & Woodley **sp. n.**, *Belvosiakeinoraragoni* Fleming & Woodley **sp. n.**, *Belvosialuciariosae* Fleming & Woodley **sp. n.**, *Belvosiamanuelpereirai* Fleming & Woodley **sp. n.**, *Belvosiamanuelriosi* Fleming & Woodley **sp. n.**, *Belvosiaminorcarmonai* Fleming & Woodley **sp. n.**, *Belvosiaosvaldoespinozai* Fleming & Woodley **sp. n.**, *Belvosiapabloumanai* Fleming & Woodley **sp. n.**, *Belvosiapetronariosae* Fleming & Woodley **sp. n.**, *Belvosiaricardocaleroi* Fleming & Woodley **sp. n.**, *Belvosiarobertoespinozai* Fleming & Woodley **sp. n.**, *Belvosiarostermoragai* Fleming & Woodley **sp. n.**, *Belvosiaruthfrancoae* Fleming & Woodley **sp. n.**, *Belvosiasergioriosi* Fleming & Woodley **sp. n.**

*Belvosiacanalis* Aldrich, 1928 is reared and recorded from the inventory; new information relative to host is provided and the species is rediscribed.

The following are proposed by Fleming & Woodley as new synonyms of *Belvosia* Robineau-Desvoidy, 1830: *Brachybelvosia* Townsend, 1927 **syn. n.**, *Belvosiomimops* Townsend, 1935 **syn. n.**

The following three new combinations are proposed as a result of the new synonymies: *Belvosiabrasilensis* (Townsend, 1927), **comb. n.**; and *Belvosiabarbiellinii* (Townsend, 1935), **comb. n.**

The authors also propose the following new synonymies: *Belvosiabrasilensis* (Townsend, 1927) = *Belvosiaaurulenta* (Bigot, 1888), **syn. n.**; *Belvosiapollinosa* Rowe, 1933 = *Belvosiaborealis* Aldrich, 1928 **syn. n.**; *Belvosiaweyenberghiana* (Wulp, 1883) = *Belvosiafuliginosa* (Walker, 1853) **syn. n.**; *Belvosiabrasiliensis* Townsend, 1927 = *Belvosiafuliginosa* (Walker, 1853) **syn. n.**; *Belvosialuteola* Coquillett, 1900 = *Belvosiaochriventris* (Wulp, 1890) **syn. n.**; *Belvosiasocia* (Walker, 1853) = *Belvosiaproxima* (Walker, 1853) **syn. n.**; *Belvosiachrysopyga* (Bigot, 1887) = *Belvosiaunifasciata* (Robineau-Desvoidy, 1830) **syn. n.**; *Belvosiachrysopygata* (Bigot, 1888) = *Belvosiaunifasciata* (Robineau-Desvoidy, 1830) **syn. n.**

## Introduction

The tribe Goniini is widely considered to be monophyletic by tachinid workers (O’Hara and Cooper 1992) based on the apomorphic "microtype" eggs that females of this tribe lay on foliage. The tiny eggs are laid in large quantities on the leaves of the food plant of the tachinid’s insect host, where they may be fortuitously ingested by a host larva. If an egg is ingested, the larva hatches and burrows through the gut wall to develop elsewhere in the host’s body. Multiple eggs can be ingested by a single host larva. In the case of *Belvosia*, the lepidopteran larval host begins the process of pupation, whereupon it is killed and the *Belvosia* larva(e) then pupate within the host's pupal case.

The genus *Belvosia* Robineau-Desvoidy 1830, is an exclusively New World genus in the tribe Goniini of the subfamily Exoristinae (sensu Herting 1984). *Belvosia* (Exoristinae, Goniini) was erected by Robineau-Desvoidy 1830 with the description of the type species, *Belvosiabicincta* (Robineau-Desvoidy 1830), dedicating the generic name to the French naturalist Palisot de Beauvois. The original description of *B.bicincta* was based on multiple syntypes from Antilles and Carolina [sic]; Townsend (1931) fixed a lectotype for the species at which point the type locality for *B.bicincta* was determined as West Indies. The genus has been constantly tinkered with since its inception. However the most comprehensive work to date on the genus was Aldrich (1928) revision of the genus *Belvosia*, which proposed seven new synonymies and described 19 new species bringing the total number of *Belvosia* to 36.

According to the most recent catalogs, there have been 15 species recognized as valid in the Nearctic Region (O'Hara and Wood 2004) and 64 species in America south of the United States (Guimarães 1977, Guimarães 1971; placed in several genera). Seven of these were recorded from both regions, so a total of 72 species have been described in the genus that are considered valid. The Neotropical species are particularly poorly known. Within, Area de Conservación Guanacaste in northwestern Costa Rica, 20 morphospecies have been sorted from reared material from an extensive inventory of Lepidoptera, their parasitoids and their hosts (http://janzen.sas.upenn.edu). DNA barcoding of extensive samples of each of these species revealed that three of them contained additional cryptic species, for a total of 33 species (Smith et al. 2006). Thus, we consider the species richness in *Belvosia* to be considerably greater than what has been formally described in the Neotropical Region.

All of the new species of *Belvosia* reared from ACG described in this paper are based on differences in external morphology, male terminalia, CO1 (DNA barcodes, cox1 or cytochrome oxidase 1) gene sequences, and on comparison by AJF and NW with other named species of *Belvosia* from other regions. It is important to note however, that these new species are not to be taken as an indication of the total number of species of *Belvosia* even in such a small country as Costa Rica. Comparisons of tachinids collected during the ACG inventory with those present in the national collection in the Museo Nacional de Costa Rica (formerly the INBIO collection) show minimal overlap in species, suggesting that the tachinid fauna in other parts of the country is quite different from that of ACG and requires much additional study. Our study provides descriptions of these 33 new species of *Belvosia* Townsend, we also synonymize two genera, adding 3 new combinations, thereby bringing the total number of species in the genus from 72 to 107. There may also be a small number of apparent species of *Belvosia* that have been reared by the ACG inventory that at present can either only be distinguished only by their gene sequences (henceforth referred to as DNA barcodes) and host records, or have insufficient material to make an accurate diagnosis; AJF and NEW have elected to leave such cases for later description when additional material is available.

## Materials and methods

### Project aims and rearing intensity

The rearing information and flies presented herein were collected by the ongoing ACG inventory of the caterpillars, their food plants, and their parasitoids (Janzen et al. 2009, Janzen et al. 2011, Janzen and Hallwachs 2011, Janzen and Hallwachs 2016, Janzen et al. 2020). The rearing methods used are described in detail at http://janzen.bio.upenn.edu/caterpillars/methodology/how/parasitoid_husbandry.htm.

Since its inception, this inventory has reared more than 750,000+ wild-caught caterpillars collected throughout the major ACG terrestrial ecosystems (Janzen et al. 2009, Janzen et al. 2020). This effort continues to provide an unprecedented amount of data (available at http://janzen.bio.upenn.edu/), providing an invaluable tri-trophic database image of parasitoid biology, including parasitoids, their hosts, and food plants. All frequencies of parasitism reported here must be considered against this background inventory, which will be analyzed in detail in future works by DHJ, WH and multiple co-authors.

The scope of our treatment of the genus is limited to those species found in the North and Mesoamerican regions, ranging from Canada to Panama's southern border with Colombia. While we included all known species in our comparisons and determinations of new species, only those species distributed within this region are included in our key.

The present study is part of a larger group of studies to document the tachinid species living within Area de Conservación Guanacaste (http://www.acguanacaste.ac.cr) (ACG) and provides names to new species as they are described (Fleming et al. 2014a, Fleming et al. 2014b, Fleming et al. 2015c, Fleming et al. 2015d, Fleming et al. 2015a, Fleming et al. 2015b, Fleming et al. 2016a, Fleming et al. 2016b, Fleming et al. 2017b, Fleming et al. 2017a, Fleming et al. 2019, Fleming et al. 2020). This series of taxonomic papers will represent a baseline for later, detailed ecological and behavioral accounts and studies extending across ACG ecological groups, whole ecosystems, and taxonomic assemblages much larger than a genus.

### Imaging and Dissections

The species accounts and descriptions presented in this paper are deliberately concise and complemented with a series of color photos, used to illustrate the morphological differences and similarities among them. The morphological terminology used follows the most recent anatomical terminology outlined in Cumming and Wood (2009), and mentioned subsequently in Cumming and Wood (2017). The characters in our descriptions are presented in order of appearance on the body from anterior to posterior and arranged by the headings **Head**, **Thorax**, **Abdomen** and **Male terminalia**. All dissections and photography were carried out following the methods detailed by (Fleming et al. 2014). Measurements and examples of anatomical landmarks discussed herein are illustrated in Fig. 1 and Fig. 2. Whenever possible males were selected preferentially as the holotype, since they often bear the most differences in external morphology and are thus better for morphologically-based species recognition. The authors note that in those cases where only one male was available, this was designated the holotype and not subjected to dissection.

### Voucher specimen management

The management of voucher specimens has been detailed in previous papers in this series (Fleming et al. 2014). In brief, caterpillars reared from the ACG inventory receive a unique voucher code in the format yy–SRNP–xxxxx. Parasitoids emerging from a caterpillar receive the same voucher code; when/if they are later individually processed for DNA barcoding, each specimen receives a second, unique voucher code in the format DHJPARxxxxxxx. The associated data for each voucher code are available at: http://janzen.bio.upenn.edu/caterpillars/database.lasso. All associated data and successful barcodes are permanently and publicly deposited in the Barcode of Life Data System (BOLD) (Ratnasingham and Hebert 2007). All inventoried specimens discussed herein were collected under Costa Rican government research permits issued to DHJ, and the Tachinidae samples were exported under permit by DHJ from Costa Rica to their final depository in the CNC. Tachinid identifications for the inventory are done by DHJ in coordination with a) visual inspection of morphology by AJF and NEW, b) DNA barcoding by MAS and BIO, and c) databasing and association with host caterpillars by DHJ and WH through the inventory itself.

The date of capture cited for each specimen is the date of eclosion of the fly and not the date of capture of the caterpillar. Eclosion date is much more representative of the time when that fly species is on the wing and therefore caught by net or Malaise trap, than is the time of capture of the parasitized caterpillar. The “collector” is the parataxonomist who found the caterpillar, rather than the person who later retrieved the newly eclosed fly and processed it by freezing, pinning, labelling and oven-drying. The primary type material of the newly-described species are housed in the Diptera collection of the Canadian National Collection (CNC).

Due to the overwhelming size the cumulative dataset, the paratype records collected for the present work were published separately through GBIF (DOI), and have also been included here as supplementary material (Suppl. material 1). Only data pertaining to the holotype and any imaged paratypes were included in the main text, all remaining specimens subsequently published as paratypes are housed at the CNC. In some cases, where the rearings were exceedingly numerous, we have elected to truncate the number of paratypes to 50, as reflected in the supplementary material.

### Acronyms for Depositories

- AMNH American Museum of Natural History, New York, New York, USA
- CAS California Academy of Sciences, San Francisco, California, USA
- CEA Estación Experimental Agronómica, Maipú, Maipú, Chile
- CNC Canadian National Collection of Insects, Arachnids and Nematodes, Ottawa, Canada
- IFML Instituto y Fundación Miguel Lillo, Tucumán, Argentina
- MCZ Museum of Comparative Zoology, Harvard University, Cambridge, Massachusetts, USA
- MACN Museo Argentino de Ciencias Naturales Bernardino Rivadavia, Buenos Aires, Argentina
- MLPA Museo de La Plata, La Plata, Argentina
- MNHN Muséum National d'Histoire Naturelle, Paris, France
- MZUT Museo di Zoologia, Instituto di Zoologia e Anatomia Comparata Universita di Torino
- NHMUK Natural History Museum, London, United Kingdom (formerly British Museum of Natural History)
- NHMW Naturhistorisches Museum Wien, Vienna, Austria
- SEMK Snow Entomological Museum, University of Kansas, Lawrence, Kansas, USA
- UMNH Utah Museum of Natural History, University of Utah, Salt Lake City, Utah, USA
- USNM National Museum of Natural History, Washington, D.C., U.S.A. (formerly United States National Museum)
- ZMUC Zoologisk Museum, Copenhagen, Denmark

### Interim names for undescribed host species

As in the other papers in this series, our convention for naming undescribed host species follows a standardized, interim naming system used for taxonomic units considered as distinct species and identified by DNA barcodes. Interim names are given in the format "*Eois* Janzen52" or "*Caviriaregina*DHJ01", where the "species epithet" is either composed of the name of the taxonomist who identified the species and a number or the name of a species-group followed by a code. This prevents confusion with already described species while maintaining traceability of each undescribed species and specimen within the ACG inventory project.

### DNA barcoding

We generated the standard DNA barcode region (5’ cytochrome c oxidase I (COI) gene) for all specimens of ACG *Belvosia*; these being made of DNA extracts from single legs using a standard glass fiber protocol (Ivanova et al. 2006). We amplified the DNA barcodes (658 bp near the 5’ terminus of the COI gene) using general insect primers and using standard protocols for production and quality control (Smith et al. 2006, Smith et al. 2007, Smith et al. 2008, Smith et al. 2009, Smith 2012). All DNA sequences, trace files and accessions have been deposited on the Barcode of Life Data System (BOLD) (Ratnasingham and Hebert 2007). BOLD can be consulted for metadata (including GenBank accession codes) associated with each sequence, by using the persistent DOI doi.org/10.5883/DS-ASBELVOS.

### Belvosiafreddyquesadai species complex

**Figure 3.**
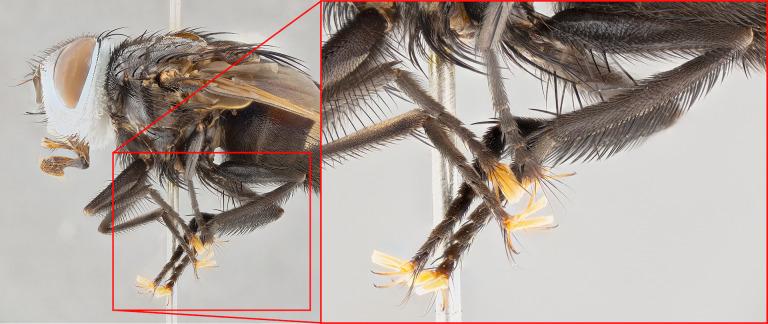
Detail of tibial comb stereotypical to the *freddyquesadai* species group, on male holotype of *Belvosiahazelcambroneroae* **sp. n.**

For the purposes of our morphological key, the authors have chosen to designate a new species complex. Despite being easily distinguishable at the molecular level, this group poses some difficulty when examined externally. The *Belvosiafreddyquesadai* species complex is distinguished from the other species of *Belvosia* using a combination of character states: (1) presence of dark brown/black basicosta (), (2) presence of a discernible sex patch, and (3) anterodorsal setae on hind tibia regular and comblike, typically at most 1.25X as long as width of supporting tibia (Fig. 3), with each seta separated from the other with regular spacing no more than the width of the base of the preceding seta, and the barcode. Included in this group are the following new species: *Belvosiafreddyquesadai* **sp. n.**, *Belvosiagloriasihezarae* **sp. n.**, *Belvosiaguillermopereirai* **sp. n.**, *Belvosiaharryramirezi* **sp. n.**, *Belvosiahazelcambroneroae* **sp. n.**, *Belvosiajorgehernandezi* **sp. n.**, *Belvosiajosecortezi* **sp. n.**, and *Belvosiajoseperezi* **sp. n.** Dissection of male terminalia, in addition to the barcode data remain the only clear way to distinguish the many of the species included in this complex.

## Taxon treatments

### Belvosia

Robineau-Desvoidy, 1830

CFD02E3D-A37C-5920-924E-A9F4CB2940AF

Belvosia Robineau-Desvoidy, 1830 – Robineau-Desvoidy 1830: 103. Type species: *Belvosiabicincta* Robineau-Desvoidy, 1830, by monotypy.
Latreillia Robineau-Desvoidy, 1830 – Robineau-Desvoidy 1830: 104. Type species: *Muscabifasciata* Fabricius, 1775 - Fabricius 1775, by subsequent designation of Coquillett 1910: 558. Junior homonym of *Latreillia* Roux, 1830. Synonymy by action of Aldrich 1928: 1.
Willistonia Brauer and Von Bergenstamm, 1889 – Brauer and Von Bergenstamm 1889: 97. Type species: *Muscaesuriens* Fabricius, 1805, – Fabricius 1805 [misidentified by Brauer and Von Bergenstamm, 1889 = *Willistoniaaldrichi* Townsend, 1931], by monotypy. Synonymy by action of Aldrich 1928: 1.
Latreillimyia Townsend, 1908 – Townsend 1908: 105, replacement name for *Latreillia* Robineau-Desvoidy, 1830. Synonymy by action of Aldrich 1928: 1.
Triachora Townsend, 1908 – Townsend 1908: 105. Type species: *Latreilliaunifasciata* Robineau-Desvoidy, 1830, by monotypy. Synonymy by action of Aldrich 1928: 1.
Goniomima Townsend, 1908 – Townsend 1908: 105. Type species: *Belvosialuteola* Coquillett, 1900, by monotypy. Synonymy by action of Aldrich, 1928: 1.
Belvosiomima Townsend, 1915 – Townsend 1915: 413. Type species: *Belvosiomimafosteri* Townsend, 1915, by original designation. Synonymy by action of Aldrich 1928: 1.
Belvoisia Loew, 1862 – Loew 1862: 35. Incorrect subsequent spelling of *Belvosia* Robineau-Desvoidy, 1830.
Belvosiopsis Townsend, 1927 – Townsend 1927: 248. Type species: *Belvosiopsisbrasiliensis* Townsend, 1927 [=*Belvosiafuliginosa* Walker, 1853], by original designation. Synonymy by action of Aldrich 1928: 1.
Pseudobelvosia Blanchard, 1954 – Blanchard 1954: 8. Type species: *Pseuodobelvosialugubris* Blanchard, 1954, by original designation. Synonymy by action of Guimarães 1971: 181.
Neobelvosiopsis Blanchard, 1954 – Blanchard 1954: 20. Type species: *Neobelvosiopsisbosqi* Blanchard, 1954, by original designation. Synonymy by action of Guimarães 1971: 181.
Parabelvosia Blanchard, 1954 – Blanchard 1954: 12. Type species: *Parabelvosiatibialis* Blanchard, 1954, by original designation. Synonymy by action of Guimarães 1971: 181.
Eubelvosiopsis Blanchard, 1954 – Blanchard 1954: 15. Type species: *Eubelvosiopsisformosana* Blanchard, 1954, by original designation. Synonymy by action of Guimarães 1971: 181.
**Conspectus of species currently included in *Belvosia* Robineau-Desvoidy, 1830**
aldrichi Townsend, 1931 – Townsend 1931: 468 (*Willistonia*). Holotype male (NHMW) [examined by NEW]. Type locality: Brazil [misidentified as *Muscaesuriens* sensu Brauer & Bergenstamm, and Aldrich, not Fabricius or Wiedemann]
analis Macquart, 1846 – Macquart 1846: 288 (*Belvosia*). Holotype male (depository unknown) [not examined, specimen not located in MNHN or NHMUK]. Type locality: Brazil. ***Nomen dubium***
ansata Reinhard, 1951 – Reinhard 1951: 2 (*Belvosia*). Holotype male (CNC) [examined by NEW & AJF]. Type locality: Mexico, Jalisco [as Michoacan, in error], Guadalajara.
argentifrons Aldrich, 1928 – Aldrich 1928: 32 (*Belvosia*). Holotype male (USNM) [examined by NEW & AJF]. Type locality: USA, Virginia, Falls Church.
atrata Walker, 1853 – Walker 1853: 284 (*Tachina*). Holotype male (NHMUK) [examined by NEW]. Type locality: Brazil.
auratilis Reinhard, 1951 – Reinhard 1951: 1 (*Belvosia*). Holotype male (CNC) [examined by NEW & AJF]. Type locality: Mexico, Jalisco [as Michoacan, in error], Guadalajara.
auripilosa Blanchard, 1954 – Blanchard 1954: 39 (*Willistonia*) Holotype male (MACN) [examined by NEW]. Type locality: Argentina.
aurulenta Bigot, 1888 – Bigot 1888: 84 (*Frontina*). Holotype male published as female (NHMUK) [examined by NEW]. Type locality: Brazil.
brasilensis Townsend, 1927 – Townsend 1927: 291 (*Brachybelvosia*). Correct original spelling by present revision. Lectotype male (USNM), Townsend's statement "Ht male” in Manual of Myiology IX (Townsend, 1941: 62) constitutes a lectotype designation [examined by NEW & AJF]. Type locality: Brazil, São Paulo, Itaquaquecetuba. **Comb. n. & Syn. n.**
brasiliensis Townsend 1927 – Townsend 1927: 248 (*Brachybelvosia*). Incorrect original spelling.
australis Aldrich, 1928 – Aldrich 1928: 8 (*Belvosia*). Holotype female (MCZ) [examined by NEW]. Type locality: Brazil, Rio Grande do Sul.
barbiellinii Townsend, 1935 – Townsend 1935: 229 (*Belvosiomimops*). Holotype male (USNM or lost). Type locality: Brazil, São Paulo, São Vicente. **Comb. n.**
barbosai Cortés and Campos, 1971 – Cortés and Campos 1971: 98 (*Triachora*). Holotype female (CEA) [examined by NEW]. Type locality: Chile, Tarapaca, Codpa.
basalis Walker, 1853 – Walker 1853: 285 (*Tachina*). One male syntype (NHMUK) [examined by NEW]. Type locality: South America.
bicincta Robineau-Desvoidy, 1830 – Robineau-Desvoidy 1830: 103 (*Belvosia*). Lectotype female (MNHN), by fixation of Townsend, 1931a: 176 (mention of “Ht” is regarded as a lectotype fixation) [examined by NEW]. Type locality: West Indies.
biezankoi Blanchard *in* Biezanko, 1961 – Biezanko 1961: 5 (*Willistonia*). Holotype male (MACN) [examined by NEW]. Type locality: Brazil, Parana, Curitiba [Type locality published as Argentina, Buenos Aires by Guimarães in error, as Blanchard did not cite the type locality in the original publication, type locality appears handwritten on the specimen labeled holotype in MACN (Mulieri et al. 2013)].
bifasciata Fabricius, 1775 – Fabricius 1775: 777 (*Musca*). Holotype unknown destroyed (ZMUC). Type locality: America (St. Croix). [The literature on this species is difficult to tease apart, it is likely that the current concept of *Belvosiabifasciata* R.D. is actually a complex of at least two separate species. Townsend 1941: 67 interpreted the locality of the Fabricius holotype as likely originating from Cuba, however based on the original collector cited by Fabricius it is more likely that the locality is St. Croix in what was then the Danish West Indies (Papavero 1973, Thompson 1981)]
borealis Aldrich, 1928 – Aldrich 1928: 28 (*Belvosia*). Holotype male (USNM) [examined by NEW & AJF]. Type locality: USA, Pensylvania, Harrisburg.
orion Brimley, 1928 – Brimley 1928: 205 (*Belvosia*). Holotype male (USNM) [examined by NEW & AJF]. Type locality: USA, North Carolina, Raleigh.
pollinosa Rowe, 1933 – Rowe 1933: 123 (*Belvosia*). Holotype male (UMNH) [examined by NEW]. Type locality: USA, Illinois, Alto Pass. **Syn. n.**
bosqi Blanchard, 1954 – Blanchard 1954: 20 (*Neobelvosiopsis*). Holotype female, published as male (MACN). Type locality: Argentina, Misiones, Loreto.
bruchi Blanchard, 1954 – Blanchard 1954: 34 (*Belvosiomima*). Holotype male (MACN) [examined by NEW]. Type locality: Argentina, Córdoba, Alta Gracia.
canadensis Curran, 1927b – Curran 1927b: 152 (*Belvosia*). Holotype male (CNC) [examined by NEW & AJF]. Type locality: Canada, Saskatchewan, Piapot Reserve.
canalis Aldrich, 1928 – Aldrich 1928: 44 (*Belvosia*). Holotype male (USNM) [examined by NEW & AJF]. Type locality: Panama, Canal Zone, Barro Colorado Island.
catamarcensis Blanchard, 1954 – Blanchard 1954: 37 (*Belvosiomima*). Holotype male (MACN) [examined by NEW]. Type locality: Argentina, Catamarca, Pomansillo.
chaetosa Blanchard, 1954 – Blanchard 1954: 28 (*Latreillimyia*). Holotype male (MACN) [examined by NEW]. Type locality: Argentina, Tucumán.
chiesai Blanchard, 1954 – Blanchard 1954: 42 (*Willistonia*). Two male syntypes (MACN) [examined by NEW]. Type locality: Argentina, Córdoba.
ciliata Aldrich, 1928 – Aldrich 1928: 22 (*Belvosia*). Holotype male (AMNH) [examined by NEW]. Type locality: Mexico.
contermina Walker, 1853 – Walker 1853: 285 (*Tachina*). Holotype male (NHMUK) [examined by NEW]. Type locality: South America.
desita Walker, 1861 – Walker 1861: 299 (*Eurigaster*). Holotype male (NHMUK) [examined by NEW]. Type locality: Mexico.
elusa Aldrich, 1928 – Aldrich 1928: 25 (*Belvosia*). Holotype female (AMNH) [examined by NEW]. Type locality: Brazil, Mato Grosso, Santa Anna da Chapada.
equinoctalis Townsend, 1912 – Townsend 1912: 348 (*Triachora*). Holotype male (USNM) [examined by NEW & AJF]. Type locality: Peru, Piura.
insularis Curran, 1927a – Curran 1927a: 4 (*Belvoisia*). Holotype female (AMNH) [examined by NEW]. Type locality: Puerto Rico, Barros [as Porto Rico, Barros].
antilliana Curran, 1927b – Curran 1927b: 151 (*Belvosia*). Type status not given, described in key only, from an unspecified number and unknown depository. Type locality: Brazil, Rio de Janeiro. **Syn. n.**
ferruginosa Townsend, 1895 – Townsend 1895: 71 (*Belvosia*). Holotype male (NHMUK) [examined by NEW]. Type locality: Jamaica, Bath.
formosa Aldrich, 1928 – Aldrich 1928: 23 (*Belvosia*). Holotype male (USNM) [examined by NEW & AJF]. Type locality: West Indies, St. Clair. [originally published as Belvosiaciliatavar.formosa Aldrich, 1928: 33]
formosana Blanchard, 1954 – Blanchard 1954: 15 (*Eubelvosiopsis*). Six female syntypes (MACN) [examined by NEW]. Type locality: Argentina, Formosa.
fosteri Townsend, 1915 – Townsend 1915: 414 (*Belvosiomima*). Holotype female (USNM) [examined by NEW & AJF]. Type locality: Paraguay, Sapucay.
frontalis Aldrich, 1928 – Aldrich 1928: 24 (*Belvosia*). Lectotype male (AMNH), designated by Arnaud, 1963: [examined by NEW]. Type locality: Brazil, Mato Grosso, Santa Anna da Chapada.
fuscisquamula Blanchard, 1954 – Blanchard 1954: 44 (*Willistonia*). Unspecified number of syntypes (only 1 male syntype remaining in MACN, remainder of type series presumably deposited in IFML) [examined by NEW]. Type locality: Argentina, Catamarca, Belen, Hualfin.
fuliginosa Walker, 1853 – Walker 1853: 286 (*Tachina*). Holotype male (NHMUK) [examined by NEW]. Type locality: unknown, presumed South America according to label on holotype.
weyenberghiana Wulp, 1883 – Wulp 1883: 26 (*Belvosia*). Depository and type status unknown. Type locality: Argentina. **Syn. n.**
brasiliensis Townsend, 1927 – Townsend 1927: 289 (*Belvosiopsis*). Holotype female (unknown). Type locality: Brazil, São Paulo, Itaquaquecetuba. **Syn. n.**
lata Aldrich, 1928 – Aldrich 1928: 39 (*Belvosia*). Holotype female (USNM) [examined by NEW]. Type locality: Guatemala, Puerto Barrios.
leucopyga Wulp, 1882 – Wulp 1882: 84 (*Belvosia*). Holotype female (NHMW) [examined by NEW]. Type locality: Brazil.
lilloi Blanchard, 1954 – Blanchard 1954: 47 (*Willistonia*). Holotype male (IFML). Type locality: Argentina, Tucuman.
lugubris Blanchard, 1954 – Blanchard 1954: 10 (*Pseudobelvosia*). Holotype female (MACN) [examined by NEW]. Type locality: Argentina, Misiones.
manni Aldrich, 1928 – Aldrich 1928: 7 (*Belvosia*) Holotype female (USNM) [Examined by NEW & AJF]. Type locality: Bolivia, Ixiamas.
matamorosa Reinhard, 1951 – Reinhard 1951: 3 (*Belvosia*). Holotype male (CNC) Type locality: Mexico, Puebla, Matamoros.
mira Reinhard, 1958 – Reinhard 1958: 232 (*Belvosia*). Holotype female (CAS) [examined by AJF]. Type locality: Mexico, Oaxaca, Tehuantepec.
naccina Reinhard, 1974 – Reinhard 1974: 1158 (*Belvosia*). Holotype male (CNC) [examined by NEW & AJF]. Type locality: Mexico, Veracruz, Jalapa.
nigrifrons Aldrich, 1928 – Aldrich 1928: 38 (*Belvosia*). Holotype female (USNM) [examined by NEW & AJF]. Type locality: El Salvador, Mirasol.
obesula Wulp, 1890 – Wulp 1890: 46 (*Cnephalia*). Holotype female (NHMUK) [examined by NEW]. Type locality: Mexico, Tabasco, Teapa.
ochriventris Wulp, 1890 – Wulp 1890: 47 (*Cnephalia*). Lectotype, female by present designation of NEW (NHMUK) [examined by NEW]. Type locality: Mexico, Guerrero, Tierra Colorada, 2000ft. The paralectotype female from Mexico, Guerrero, Amula, 6000 feet is not conspecific with the lectotype.
luteola Coquillett, 1900 – Coquillett 1900: 253 (*Belvosia*). Holotype male, published as female (USNM) [examined by NEW & AJF]. Type locality: Puerto Rico, Vieques Island. **Syn. n.**
omissa Aldrich, 1928 – Aldrich 1928: 21 (*Belvosia*). Holotype male (USNM) [examined by NEW & AJF]. Type locality: USA, Virginia, Falls Church.
piurana Townsend, 1911 – Townsend 1911: 143 (*Belvosia*). Holotype female (USNM) [examined by NEW & AJF]. Type locality: Peru, [Piura], Sullana.
potens Wiedemann, 1830 – Wiedemann 1830: 312 (*Tachina*). Three male syntypes (NHMW) [examined by NEW]. Type locality: Brazil, Rio de Janeiro. One syntype was apparently dissected by Townsend or Aldrich, as the male terminalia are glued to a label, and the abdomen is missing. This specimen bears a label “T. potens m/Rio Janeiro”, apparently in Wiedemann's hadwriting.
proxima Walker, 1853 – Walker 1853: 287 (*Tachina*). Holotype male (NHMUK) [examined by NEW]. Type locality: Brazil, Para.
socia Walker, 1853 – Walker 1853: 286 (*Tachina*). Holotype male (NHMUK) [examined by NEW]. Type locality: Brazil. **Syn. n.**
recticornis Macquart, 1855 – Macquart 1855: 118 (*Gonia*). Lectotype male (BMNH) [examined by NEW]. Type locality: unknown. [Lectotype label reads as follows: "LECTO-TYPE/Gonia recticornis ♀. Macq. [verso reads]Brauer WIEN. CVI[???]. (No 94)/Gonia recticornis Macq. SYNTYPE ♂ NO LOCALITY ex.Bigot Coll: B.M.1960-539./C. Recticornis. ♂ Gonia. id. Macq. J. Bigot." However the specimen labeled lectotype is in fact a male and the paralectotype is a female.] [This species was redescribed by Aldrich (1928) based on 34 specimens reared from Lepidoptera larvae collected in Panama, Mexico and Ecuador. We could not ascertain who may have published a lectotype designation.]
bella Giglio-Tos, 1893 – Giglio-Tos 1893: 3 (*Belvosia*). Holotype female (MZUT) [examined by NEW]. Type locality: Mexico. Synonymy by Aldrich 1928.
mexicana Aldrich, 1928 – Aldrich 1928: 11 (*Belvosia)*. Holotype male (USNM) [examined by NEW]. Type locality: Mexico, Ciudad de Mexico D.F.
ruficornis Aldrich, 1928 – Aldrich 1928: 16 (*Belvosia*). Lectotype male (AMNH), designated by Arnaud, 1963 [examined by NEW]. Type locality: Brazil, Mato Grosso, Santa Anna da Chapada. [Originally published as Belvosiarecticornisvar.ruficornis Aldrich, 1928: 16].
rufifrons Blanchard, 1954 – Blanchard 1954: 23 (*Belvosia*). Holotype male (MLPA) [examined by NEW]. Type locality: Argentina, Cordoba.
semiflava Aldrich, 1928 – Aldrich 1928: 11 (*Belvosia*). Holotype male (USNM) [examined by NEW & AJF]. Type locality: USA, New Mexico, White Mts., Rio Ruidoso.
slossonae Coquillett, 1895 – Coquillett 1895: 312 (*Belvosia*). Holotype female (AMNH) [examined by NEW]. Type locality: USA, Florida, Charlotte Harbor.
smithi Aldrich, 1928 – Aldrich 1928: 40 (*Belvosia*). Lectotype male (AMNH), designated by Arnaud, 1963 [examined by NEW]. Type locality: Brazil, Mato Grosso, Santa Anna da Chapada.
spinicoxa Aldrich, 1928 – Aldrich 1928: 41 (*Belvosia*). Holotype male (USNM) [examined by NEW & AJF]. Type locality: Paraguay, Sapucay.
splendens Curran, 1927b – Curran 1927b: 153 (*Belvosia*). Holotype male (CNC) [examined by NEW & AJF]. Type locality: Canada, Saskatchewan, Piapot First Nation.
tibialis Blanchard, 1954 – Blanchard 1954: 13 (*Parabelvosia*). Holotype male (MACN) [examined by NEW]. Type locality: Argentina, Misiones.
townsendi Aldrich, 1928 – Aldrich 1928: 33 (*Belvosia*). Holotype male (USNM) [examined by NEW & AJF]. Type locality: USA, Virginia, Oak Grove.
unifasciata Robineau-Desvoidy, 1830 – Robineau-Desvoidy 1830: 105 (*Latreillia*). Holotype unspecified sex (MNHN, lost according to Townsend 1941: 74). Type locality: USA, Pennsylvania, Philadelphia.
chrysopyga Bigot, 1887 – Bigot 1887: cxli (*Frontina*). Holotype female (NHMUK) [examined by NEW]. Type locality: Mexico. **Syn. n.**
chrysopygata Bigot, 1888 – Bigot 1888: 84. (*Frontina*). Unjustified emmendation of *Frontinachrysopyga* Bigot, 1887. **Syn. n.**
flavicauda Riley, 1870 – Riley 1870: 51 (*Exorista*). Lectotype male by present designation of D.M. Wood (USNM). Type locality: USA, Missouri.
vanderwulpi Williston, 1886 – Williston 1886: 303 (*Belvoisia*). Holotype female (SEMK) [examined by NEW]. Type locality: Hispaniola [as "San Domingo"]. [Originally published as *Belvoisia v. d. Wulpi* Williston, 1886: 303].
villaricana Reinhard, 1951 – Reinhard 1951: 4 (*Belvosia*). Holotype female (CNC) [examined by NEW & AJF]. Type locality: Paraguay, Villarica.
vittata Aldrich, 1928 – Aldrich 1928: 41 (*Belvosia*). Holotype male (USNM) [examined by NEW & AJF]. Type locality: Paraguay, Sapucay.
wiedemanni Aldrich, 1928 – Aldrich 1928: 19 (*Belvosia*). Holotype male (NHMW). Type locality: Brazil, Santa Catarina, Blumenau. [Aldrich noted that all 13 specimens in the type series had identical labels, and that the "type" was returned to NHMW along with 8 paratypes, 4 being retained at USNM. The holotype has a typical USNM "Type" label, and all paratypes bear typical USNM "Paratype" labels prepared by Aldrich]
williamsi Aldrich, 1928 – Aldrich 1928: 43 (*Belvosia*). Holotype male (USNM) [examined by NEW]. Type locality: Brazil, São Paulo, Campinas.
willinki Blanchard, 1954 – Blanchard 1954: 18 (*Eubelvosiopsis*). Holotype female (IFML). Type locality: Argentina, Misiones, Iguazú.
Belvosia Belvosia bicincta Robineau-Desvoidy, 1830

#### Description

**Male**, **Head**: head wide ranging from as wide as thorax to slightly wider; vertex 1/4–1/3 head width; gena 1/3 of head height, approximately 1/2 of eye height; with 1–3 rows of frontal setae; 1–3 of reclinate orbital setae (some species males with proclinate orbital setae present); ocellar setae most often absent, reduced to hair-like in some species; eye bare in all species; parafacial bare and wide, subequal to eye width; fronto-orbital plate ranging from shining silver or gold to brownish with a silver sheen, and displaying varying degrees of hirsuteness, with setulae not typically extending below lowest frontal seta; lower margin of face lower than vibrissa; facial ridge setulose, degree of hirsuteness ranging from halfway along facial ridge to 2/3 of its length; pedicel ranging from orange to black; postpedicel black to black with orange, 2–3X as long as pedicel; arista bare, usually distinctly-thickened on basal 4/5 almost to tip. **Thorax**: ranging from gold to black, sometimes with light gray to gold tomentum dorsally; four dorsal vittae, these can be thick and unbroken to thin and only scarcely present under certain angles of light; prosternum setose; postpronotum bearing three setae, middle basal seta in line with outer and inner basal setae; anterior margin of anepisternum setulose with long hair-like setulae either ranging from black to yellow or golden brown; chaetotaxy: acrostichal setae 3:3–4; dorsocentral setae 3:4; intra-alar setae 2:3; supra-alar setae 2:3; 3–6 katepisternal setae; scutellum ranging from black to gold tomentose, with 4–6 pairs of long flat marginal setae of subequal length; apical setae when present short often crossed and erect, at a slight upward angle from the plane of the rest of the scutellar marginal setae. **Legs**: most often black, many examples bearing yellow or reddish ground color, with yellow pulvilli of varying length; hind coxae bare. Anterodorsal row of setae on hind tibia either regular and comblike or irregular and not fringelike. Wing: ranging from pale translucent, to strongly infuscate, to dark gray (almost black); wing vein R_4+5_ setose, bearing only 2–3 setulae at base; calypters raging from dark gray infuscate to yellowish white. **Abdomen**: color ranging from grayish–brown to black; abdominal tomentosity ranging from strikingly yellow, often forming conspicuous bands to brilliant white or dull colored, and not forming distinct bands; in some species a narrow median black stripe is present; middorsal depression on syntergite 1+2 (ST1+2) ranging from halfway across tergite to almost reaching to hind margin; median marginal setae present on ST1+2–T5; median discal setae absent on all species; underside of abdomen with sex patch present in some species. **Male terminalia**: sternite 5 with a deeply excavated median cleft along posterior edge, smoothly U-shaped, margins covered in dense tomentum; posterior lobes rounded apically, either bare, with multiple fine hair-like setulae or with 2–3 strong setae surrounded by many shorter weaker setulae. Anterior plate of sternite 5 subequal to or longer than length of posterior lobes; unsclerotized "window" on anterior plate of sternite 5 ranging from absent to almost entirely transparent directly basal to posterior lobes, the shape of the window as well as its presence varies between species. Epandrium ranging from orange to black and variably hirsute. Cerci in posterior view variable between species ranging between rectangular, digitiform, to triangular, either longer than or only slightly shorter than surstyli; blunt and rounded at apex to apically pointed, either completely separate medially to fused basally along most of their length. Cerci in lateral view, often with a strong anterior curve on apex, giving it a clubbed appearance; cerci densely setose along basal 2/3rds, underside of cerci setose along entire length (visible in lateral profile). Surstylus in lateral view, almost equilateral along its length sometimes ending in a slightly downcurved apex making the structure appear bladelike; surstylus appearing to be separate and not fused with epandrium; when viewed posteriorly surstyli range from slightly divergent, to slightly convergent or bearing inward curved apices but not strongly convergent. Pregonite usually broad, well-developed, apically squared off or rounded, usually blunt, typically devoid of setulae. Postgonite, slightly narrowed, up to 1/3 as wide as pregonite, sharply pointed and curved at apex, typically short and scythelike, with few exceptions where postgonite is subequal in length to pregonite. Distiphallus broadly cone-shaped (in some species this cone or flare is much more pronounced, in others appearing square or barrel shaped), with a slender median longitudinal sclerotized reinforcement on its posterior surface and a broad, anterolateral, sclerotized acrophallus, on anterior surface near apex, ~1.3 times as long as basiphallus.

**Female** as in male differing in the following traits: **Head**: bearing two pairs of proclinate orbital setae. **Abdomen**: often slightly more globose than males; T5 folded over into a narrow slit a trait which is stereotypical of the tribe Goniini. In those cases where sexual dimorphism is observed the differing character states are mentioned in the species description. Sex patch absent in all female specimens.

#### Diagnosis

*Belvosia*, as in all other Goniini, are difficult to characterize to tribe based on morphological character states but can be reliably ascribed to the tribe (sensu Herting 1984) based on their microtype ovipary. *Belvosia* are a large, heavy-bodied tachinid, often with brilliant hymenoptera-like abdominal banding in brilliant white or gold. They can be diagnosed based on the following *gestalt* or combination of traits which can be considered as stereotypical to the group: prosternum setose; males of many species have two pairs of well-developed reclinate fronto-orbital setae (sometimes absent, proclinate in: *B.luteola*, *B.unifasciata*, *B.fosteri*, *B.ochriventris*, *B.slossonae*, *B.equinoctialis*), proclinate in all females; ocellar setae most frequently absent, however can appear reduced and hair-like in some species; eyes bare; facial ridge setose at least over 1/3–1/2 its height; frons distinctively wide in both sexes; parafacial bare; the three major setae of the postpronotum are arranged more or less in a line; usually 4 well developed katepisternals, but numbers can vary between 3–6; three stout postsutural supra-alar setae; abdominal discal setae absent in all species. Previous descriptions of the genus also made mention of the absence of any discernible sex patch in males, however, present evidence suggests that sex patches are in fact present in some species of *Belvosia* (*B.bicincta*, *B.ciriloumanai*, *B.duvalierbricenoi*, *B.freddyquesadai*, *B.gloriasihezarae*, *B.guillermopereirai*, *B.harryramirezi*, *B.hazelcambroneroae*, *B.jorgehernandezi*, *B.josecortezi*, *B.joseperezi*, *B.robertoespinozai*, *B.sergioriosi*). Distance between eye and subcranial margin often 1/3 the height of the head. As can be seen in the key to the Tachinidae in Wood and Zumbado 2010, *Belvosia* can be distinguished from *Lespesia* Robineau-Desvoidy 1830 which bears the following differences: eye bearing ommatrichia, well developed ocellar setae, and the facial margin arising level with vibrissa. Distinguished from *Atacta* Schiner by its robust size in addition to *Belvosia's* presence setulae on facial ridge.

#### Distribution

Ubiquitous throughout the New World, inhabiting a wide variety of ecosystems, from southeastern Canada and northeastern USA west to California and south to Argentina and Brazil.

#### Ecology

Within the ACG inventory, *Belvosia* has been reared from the following Lepidoptera hosts throughout the diverse ecosystems of the research area: Erebidae, Eupterotidae, Noctuidae, Notodontidae, Saturniidae, and Sphingidae. Ecological and natural history analysis of the thousands of rearing records will be provided later by the same authors of this work.

#### Taxon discussion

*Latreillia* Robineau-Desvoidy 1830, was proposed concurrently with *Belvosia* (Robineau-Desvoidy 1830), and originally included 10 species. Eight of the species were from the Old World; four of these are now recognized and are considered synonyms in four different genera (Crosskey 1980, Herting and Dely-Draskovits 1993), and the remaining four are unrecognized Palaearctic species (Herting and Dely-Draskovits 1993). Because Coquillett (1910) designated *Muscabifasciata* Fabricius, a typical species of *Belvosia* as now recognized, as type species, *Latreillia* became a synonym of that name. As *Belvosia* is restricted to the New World, none of the eight Old World species, including those that are unrecognized, belong to the genus. The tenth included species, *Latreilliaunifasciata* Robineau-Desvoidy, is another species of *Belvosia*. *Latreillimyia* Townsend, a replacement name for *Latreillia*, automatically becomes a synonym of *Belvosia*. *Triachora* Townsend, was proposed by Townsend to include only *Latreilliaunifasciata* Robineau-Desvoidy. It has been considered as a valid genus distinct from *Belvosia* by past authors (Sabrosky and Arnaud 1965, Guimarães 1971) for a group of about seven species. It was recently synonymized with *Belvosia* by Wood (Wood 1987, O’Hara and Wood 1998). Members of this species group are generally smaller than more typical *Belvosia*, and are not primarily black with yellow-gold abdominal bands, and males have proclinate orbital setae. However, the species exhibit the characters used to define *Belvosia* above. *Goniomima* Townsend, was proposed to include only *Belvosialuteola* Coquillett, and no additional species have ever been placed in the genus. Although it exhibits some apomorphic character states, such as the long setae on the male cerci and the narrowed abdomen similar to that found in some species of *Gonia*, *B.luteola* has the character states found in *Belvosia* and shows features that place it with the species formerly included in *Triachora*, such as the proclinate orbital setae found in males. *Goniomima* was synonymized with *Triachora* by Sabrosky and Arnaud (1965).

The previously described species *Belvosiaantillana* (Curran 1927b) was only included in a key without any reference to type material and no specimens have been located. We believe Curran was probably referring to *Belvosainsularis*, described from Puerto Rico in the same year (Curran 1927a), but inadvertantly used a different name. We therefore regard *B.antillana* as a synonym of *B.insularis*.

Aldrich (1928) treated *Belvosiaanalis* (Macquart 1846) as unrecognized within *Belvosia*; this paper cites the original type material used by Macquart (1846) as originating from Brazil, and presumably destroyed. Aldrich's treatment of this species was based on Macquart's original description where the abdomen was described as "caeruleo-nigro" which Aldrich took to mean as blue, thereby excluding it from the genus *Belvosia*. The type of *Belvosiaanalis* sensu Macquart is no longer present in the Paris Museum type list, having since been lost or destroyed. Coquillett later identified material from Mexico as belonging to *B.analis*. It was on the basis of these specimens that Aldrich conducted his diagnosis and erected the name *Belvosiaciliata* to include those specimens Coquillet had described. Since the original type material has been lost, the basis for *B.analis* Macquart cannot be ascertained, we are hereby are treating this species as a **nomen dubium**.

It is somewhat surprising that the synonymy of *B.pollinosa* with *B.borealis* has gone undetected before now. Rowe (1933) clearly noted the multiple median marginal setae on tergites 1+2 and three (i.e., more than a single pair on each segment), a character state only found in *B.borealis* in the North American fauna. Curran’s "n. sp." label on the holotype of *B.pollinosa* was presumably added around the time he was working on the genus, before the Aldrich (1928) revision. Rowe was apparently unaware of Aldrich’s paper, as the holotype of *B.pollinosa* keys easily to *B.borealis* in Aldrich’s key.

During his long and prolific career D. Monty Wood had occasion to examine *Belvosiaflavicauda* Riley, at the USNM. The original description cited 5 female syntypes in error, one captured and 4 reared from *Mamestraconfigurata* Walker, 1856. Careful examination by Dr. Wood, revealed the original captured specimen to be a male, along with 4 reared females. Prior to his passing in 2020, Monty was assisting AJ Fleming in the preparation of this paper where he suggested the inclusion of this male syntype as lectotype. We hereby propose the male syntype as the lectotype of *Belvosiaflavicauda* by present designation of D. Monty Wood.

After careful examination of the two syntypes of *Belvosiaochriventris* Wulp, it was determined that they are in fact not conspecific. In his *Biologia Centrali Americana*, Wulp described the similar characters within the syntypes and then makes mention of additional information pertaining to the syntype originating from Tierra Colorada; further describing the ground coloration of the abdomen and the presence of a dark stripe along its midline. Given the fact that more information was shared about this specimen, we have elected to designate it the lectotype of the species. We consider that the herein designated lectotype of *Belvosiaochriventris* is in fact conspecific with *Belvosialuteola* Coquillett and therefore must sink *B.luteola* as a synonym of the former based on the morphological similarities between the two. The second syntype from Mexico, Guerrero, Amula 6000ft, we hereby designate as a paralectotype. We are not currently able to make a determination on this specimen which at the present time we have chosen to leave as unresolved.

### Belvosia adrianguadamuzi

Fleming & Woodley sp. nov.

2BF46D5D-C968-51C2-B357-D4CC52E37B77

7041B9B2-FED6-4559-8B8F-9072581C75A1

#### Materials

- **Type status:** Holotype. **Occurrence:** occurrenceDetails: http://janzen.sas.upenn.edu; catalogNumber: DHJPAR0003566; recordedBy: D.H. Janzen, W. Hallwachs & Mariano Pereira; individualID: DHJPAR0003566; individualCount: 1; sex: male; lifeStage: adult; preparations: pinned; otherCatalogNumbers: HCIC670-05, 05-SRNP-58598, BOLD:AAA8366; occurrenceID: 7E16F31B-F6BF-5BA9-AA0E-9557FF093FCF; **Taxon:** scientificName: Belvosiaadrianguadamuzi; phylum: Arthropoda; class: Insecta; order: Diptera; family: Tachinidae; genus: Belvosia; specificEpithet: adrianguadamuzi; scientificNameAuthorship: Fleming & Woodley, 2023; **Location:** continent: Central America; country: Costa Rica; countryCode: CR; stateProvince: Guanacaste; county: Sector Mundo Nuevo; locality: Area de Conservacion Guanacaste; verbatimLocality: Porton Rivas; verbatimElevation: 570; verbatimLatitude: 10.7586; verbatimLongitude: -85.3727; verbatimCoordinateSystem: Decimal; decimalLatitude: 10.7586; decimalLongitude: -85.3727; **Identification:** identifiedBy: AJ Fleming; dateIdentified: 2018; **Event:** samplingProtocol: Reared from the larva of the Saturniidae, Periphobaarcaei; verbatimEventDate: 23-Sep-2005; **Record Level:** language: en; institutionCode: CNC; collectionCode: Insects; basisOfRecord: Pinned Specimen
- **Type status:** Paratype. **Occurrence:** occurrenceDetails: http://janzen.sas.upenn.edu; catalogNumber: DHJPAR0029520; recordedBy: D.H. Janzen, W. Hallwachs & Roster Moraga; individualID: DHJPAR0029520; individualCount: 1; sex: female; lifeStage: adult; preparations: pinned; otherCatalogNumbers: ASHYM941-09, 08-SRNP-22641, BOLD:AAA8366; occurrenceID: FB8C2BE1-E0DE-5507-B39F-A9B952A4D053; **Taxon:** scientificName: Belvosiaadrianguadamuzi; phylum: Arthropoda; class: Insecta; order: Diptera; family: Tachinidae; genus: Belvosia; specificEpithet: adrianguadamuzi; scientificNameAuthorship: Fleming & Woodley, 2023; **Location:** continent: Central America; country: Costa Rica; countryCode: CR; stateProvince: Guanacaste; county: Sector Del Oro; locality: Area de Conservacion Guanacaste; verbatimLocality: Quebrada Ayotal; verbatimElevation: 326; verbatimLatitude: 11.0095; verbatimLongitude: -85.5113; verbatimCoordinateSystem: Decimal; decimalLatitude: 11.0095; decimalLongitude: -85.5113; **Identification:** identifiedBy: AJ Fleming; dateIdentified: 2018; **Event:** samplingProtocol: Reared from the larva of the Saturniidae, Periphobaarcaei; verbatimEventDate: 03-Nov-2008; **Record Level:** language: en; institutionCode: CNC; collectionCode: Insects; basisOfRecord: Pinned Specimen
- **Type status:** Paratype. **Occurrence:** occurrenceDetails: http://janzen.sas.upenn.edu; catalogNumber: DHJPAR0036478; recordedBy: D.H. Janzen, W. Hallwachs & Lucia Rios; individualID: DHJPAR0036478; individualCount: 1; sex: male; lifeStage: adult; preparations: pinned; otherCatalogNumbers: ASHYE1389-09, 08-SRNP-24223, BOLD:AAA8366; occurrenceID: B79B64BC-07CC-5631-A592-BCFDA27465F2; **Taxon:** scientificName: Belvosiaadrianguadamuzi; phylum: Arthropoda; class: Insecta; order: Diptera; family: Tachinidae; genus: Belvosia; specificEpithet: adrianguadamuzi; scientificNameAuthorship: Fleming & Woodley, 2023; **Location:** continent: Central America; country: Costa Rica; countryCode: CR; stateProvince: Guanacaste; county: Sector Del Oro; locality: Area de Conservacion Guanacaste; verbatimLocality: Quebrada Salazar; verbatimElevation: 560; verbatimLatitude: 11.0022; verbatimLongitude: -85.4634; verbatimCoordinateSystem: Decimal; decimalLatitude: 11.0022; decimalLongitude: -85.4634; **Identification:** identifiedBy: AJ Fleming; dateIdentified: 2018; **Event:** samplingProtocol: Reared from the larva of the Saturniidae, Periphobaarcaei; verbatimEventDate: 17-Jun-2009; **Record Level:** language: en; institutionCode: CNC; collectionCode: Insects; basisOfRecord: Pinned Specimen

#### Description

**Male** (Fig. 4), length: 11–14mm. **Head**: head slightly wider than thorax; vertex 2/5 head width; gena 1/4 of head height, approximately 1/3 of eye height. Fronto-orbital plate dark ground color apically transitioning to lighter towards parafacial, entirely covered with silver tomentum giving the whole plate a shining character; ocellar setae weak and hair-like almost appearing absent, these arising lateral to anterior ocellus; one reclinate orbital seta outside of frontal rows; 2–3 irregular rows of frontal setae, with shorter black setulae interspersed throughout, these short black setulae extending beyond lowest frontal seta. Parafacial light yellow in ground color, densely covered in silver tomentum making the entire surface reflective and brilliant silver in appearance; bare overall, except for a small number of setulae extending just below lowest frontal setae; wide, approximately 2/3 of eye width when viewed laterally; facial ridge setose along 2/3 of its length, with a few sparse hair-like setulae emerging along outer edge of row; gena covered in light yellow yellow to reddish yellow setulae, sometimes with black setulae intermingled. Antenna, pedicel ranging from dark brownish orange, to distinctly lighter than postpedicel; postpedicel dark brownish black, 5X as long as pedicel. Palps, yellow-orange throughout and densely covered in short black setulae; slightly club shaped, but tapering to a slight point apically. **Thorax**: dark brown-black ground color throughout, with light gray tomentum dorsally, scutellum bearing a brassy-brown tomentum sometimes appearing black on some specimens; five distinct dorsal vittae, outer, inner, and one dorsocentral, these at times only becoming evident under certain angles of light. Lateral surfaces of thorax primarily covered in the same silver tomentum as on the dorsal surfaces; anterior margin of anepisternum densely hirsute with long reddish brown setulae becoming long black setulae along posterior margin; both katepisternum and anepimeron bearing the same long reddish setulae as on anepisternum; chaetotaxy: 3–4 strong setae on postpronotum arranged in a line; acrostichal setae 3:4; dorsocentral setae 3:4; intra-alar setae 3:3; supra-alar setae 2:3; 4–5 katepisternal setae (5th katepisternal sometimes weakly present below row of stronger katepisternals); scutellum, with four pairs of long flat marginal setae of subequal length, and up to two rows of median discal scutellar setae; apical setae present short crossed and erect, at a slight upward angle from the plane of the rest of the scutellar marginal setae. **Wing**: strongly infuscate, with a brilliant orange basicosta; both upper and lower calypters strongly infuscate, concolorous with the remainder of wing; wing vein R4+5, bearing 3–3 setulae at base; halteres orange stalk with dark black/brown capitulum. **Legs**: black, with yellow pulvilli; anterodorsal row of setae on hind tibia irregular and not fringelike, with several median setulae that are distinctly longer and stronger than others. **Abdomen**: flattened globose, black ground color; strikingly yellow abdominal tomentosity along anterior margin of T3, 50% of surface of T4 and 95% of surface of T5 which transitions to black along posterior apex; T4 bearing a narrow median black stripe bisecting the yellow band. Middorsal depression on ST1+2 reaching to hind margin of tergite. Median marginal setae present on ST1+2 and T3, and complete rows of setae on T4 and T5.

**Male terminalia** (Fig. 5): sternite 5 with a deeply excavated median cleft along posterior edge, smoothly and narrow with a small shoulder midway, margins covered in dense tomentum; posterior lobes rounded apically, with multiple fine hair-like setulae surrounded by many shorter weaker setulae. Anterior plate of sternite 5, 1/3 as long as posterior lobes; unsclerotized "window" on anterior plate of sternite 5 almost transparent directly basal to posterior lobes, shaped like two adjacent crescents. Cerci in posterior view broadly triangular, slightly shorter than surstyli; blunt and rounded at apex, completely separate medially to fused along basal 1/2. Cerci in lateral view, with a slight bend at apex, giving it a vaguely clubbed appearance; cerci densely setose along basal 2/3rds. Surstylus in lateral view, broad and bladelike, with a straight anterior edge and curved posterior edge; surstylus appearing to be separate and not fused with epandrium; when viewed posteriorly surstyli parallel and straight. Pregonite broad, well-developed, apically rounded, blunt, marginally setose. Postgonite, slightly narrowed, 1/3 as wide as pregonite, bluntly rounded with a slight curve at apex, short. Distiphallus narrow cone-shaped, with a slender median longitudinal sclerotized reinforcement on its posterior surface and a broad, anterolateral, sclerotized acrophallus, on anterior surface near apex, 1.5X as long as basiphallus; epiphallus, short and rounded, appearing as a small hump on dorsal surface of basiphallus.

**Female** (Fig. 6) length: 10–15mm, overall morphology as in male differing in the following traits: **Head**: bearing two pairs of proclinate orbital setae in addition to single pair of reclinate orbital seta. **Thorax**: scutellum with up to 6 pairs marginal scutellar setae although most often similar to males. **Abdomen**: slightly more globose than males.

#### Diagnosis

*Belvosiaadrianguadamuzi* **sp. n.** can be distinguished from all other *Belvosia* by the following combination of traits: dark setulae below lowest frontal setae, along with light setulae on parafacial, orange basicosta, four postsutural acrostichals, and T4 with gold tomentum over 50% of tergite.

#### Etymology

*Belvosiaadrianguadamuzi* **sp. n**, is named in honor of Sr. Adrian Guadamuz in recognition of his decades of being part of the Parataxonomist Program of Area de Conservación Guanacaste (http://www.acguanacaste.ac.cr) in northwestern Costa Rica (Janzen and Hallwachs 2011). Interim species-specific name included in previously circulating databases and publications, *Belvosia* Woodley01.

#### Distribution

Costa Rica, ACG (Guanacaste Province), 10–640 m elevation.

#### Ecology

*Belvosiaadrianguadamuzi* **sp. n.** has been reared 214 times from two species of Lepidoptera in the family Saturniidae, *Periphobaarcaei* Druce, 1886 (N=212), and *Automerisbanus* (Boisduval, 1875) (N= 2), in dry forest, dry-rain lowland intergrade, with only seven rearing events from rain forest.

### Belvosia anacarballoae

Fleming & Woodley sp. nov.

49B0A2FE-BA0A-5025-9EFC-95F13E3AAFB4

8BBD0FF1-3795-43C6-BC8C-4D0C39FAC0F2

#### Materials

- **Type status:** Holotype. **Occurrence:** occurrenceDetails: http://janzen.sas.upenn.edu; catalogNumber: DHJPAR0015214; recordedBy: D.H. Janzen, W. Hallwachs & Guillermo Pereira; individualID: DHJPAR0015214; individualCount: 1; sex: male; lifeStage: adult; preparations: pinned; otherCatalogNumbers: ASBE371-06, 05-SRNP-63685, BOLD:AAA2299; occurrenceID: 83D585DE-17E6-522B-8C5F-106FF215A9F0; **Taxon:** scientificName: Belvosiaanacarballoae; phylum: Arthropoda; class: Insecta; order: Diptera; family: Tachinidae; genus: Belvosia; specificEpithet: anacarballoae; scientificNameAuthorship: Fleming & Woodley, 2023; **Location:** continent: Central America; country: Costa Rica; countryCode: CR; stateProvince: Guanacaste; county: Sector Horizontes; locality: Area de Conservacion Guanacaste; verbatimLocality: Torre Esperanza; verbatimElevation: 85; verbatimLatitude: 10.7894; verbatimLongitude: -85.551; verbatimCoordinateSystem: Decimal; decimalLatitude: 10.7894; decimalLongitude: -85.551; **Identification:** identifiedBy: AJ Fleming; dateIdentified: 2022; **Event:** samplingProtocol: Reared from the larva of the Saturniidae, Automeriszozimanaguana; verbatimEventDate: 24-Jun-2006; **Record Level:** language: en; institutionCode: CNC; collectionCode: Insects; basisOfRecord: Pinned Specimen
- **Type status:** Paratype. **Occurrence:** occurrenceDetails: http://janzen.sas.upenn.edu; catalogNumber: DHJPAR0003591; recordedBy: D.H. Janzen, W. Hallwachs & Daniel H. Janzen; individualID: DHJPAR0003591; individualCount: 1; sex: male; lifeStage: adult; preparations: pinned; otherCatalogNumbers: HCIC695-05, 84-SRNP-1199, BOLD:AAA2299; occurrenceID: 4249A67C-20F7-58DA-ABF1-452BC663149C; **Taxon:** scientificName: Belvosiaanacarballoae; phylum: Arthropoda; class: Insecta; order: Diptera; family: Tachinidae; genus: Belvosia; specificEpithet: anacarballoae; scientificNameAuthorship: Fleming & Woodley, 2023; **Location:** continent: Central America; country: Costa Rica; countryCode: CR; stateProvince: Guanacaste; county: Sector Santa Rosa; locality: Area de Conservacion Guanacaste; verbatimLocality: Bosque Humedo; verbatimElevation: 290; verbatimLatitude: 10.8514; verbatimLongitude: -85.608; verbatimCoordinateSystem: Decimal; decimalLatitude: 10.8514; decimalLongitude: -85.608; **Identification:** identifiedBy: AJ Fleming; dateIdentified: 2022; **Event:** samplingProtocol: Reared from the larva of the Sphingidae, Manduca lanuginosa; verbatimEventDate: 16-Aug-1984; **Record Level:** language: en; institutionCode: CNC; collectionCode: Insects; basisOfRecord: Pinned Specimen
- **Type status:** Paratype. **Occurrence:** occurrenceDetails: http://janzen.sas.upenn.edu; catalogNumber: DHJPAR0040103; recordedBy: D.H. Janzen, W. Hallwachs & Guillermo Pereira; individualID: DHJPAR0040103; individualCount: 1; sex: female; lifeStage: adult; preparations: pinned; otherCatalogNumbers: ASHYE2271-11, 10-SRNP-13666, BOLD:AAA2299; occurrenceID: D6D35CCE-A99C-5B5A-962C-2A2697DA0997; **Taxon:** scientificName: Belvosiaanacarballoae; phylum: Arthropoda; class: Insecta; order: Diptera; family: Tachinidae; genus: Belvosia; specificEpithet: anacarballoae; scientificNameAuthorship: Fleming & Woodley, 2023; **Location:** continent: Central America; country: Costa Rica; countryCode: CR; stateProvince: Guanacaste; county: Potrerillos; locality: Area de Conservacion Guanacaste; verbatimLocality: Rio Azufrado; verbatimElevation: 95; verbatimLatitude: 10.8122; verbatimLongitude: -85.5444; verbatimCoordinateSystem: Decimal; decimalLatitude: 10.8122; decimalLongitude: -85.5444; **Identification:** identifiedBy: AJ Fleming; dateIdentified: 2022; **Event:** samplingProtocol: Reared from the larva of the Saturniidae, Automeriszozimanaguana; verbatimEventDate: 28-Aug-2010; **Record Level:** language: en; institutionCode: CNC; collectionCode: Insects; basisOfRecord: Pinned Specimen

#### Description

**Male** (Fig. 7), length: 11–13mm. **Head**: head slightly wider than thorax; vertex 1/3 head width; gena 1/4 of head height, approximately 1/3 of eye height. Fronto-orbital plate dark ground color, entirely covered with silver tomentum giving the whole plate a shining silver character; ocellar setae absent; reclinate orbital seta absent; 2–3 irregular rows of frontal setae, with shorter black setulae interspersed throughout, these short black setulae extending beyond lowest frontal seta. Parafacial light yellow in ground color, densely covered in silver tomentum making the entire surface reflective and brilliant silver in appearance; bare overall, except for a small number of setulae extending just below lowest frontal setae; facial ridge setose along 1/2 of its length, with a few sparse hair-like setulae emerging along outer edge of row; gena covered in black setulae. Antenna, pedicel dark brownish black, to concolorous with postpedicel; postpedicel, 3X as long as pedicel. Palps, yellow-orange throughout and densely covered in short black setulae; slightly clubbed, but gradually tapering to a slight point apically. **Thorax**: dark brown-black ground color throughout, with dark gray tomentum dorsally, scutellum light brown to dark yellow ground color bearing a brassy-brown tomentum; four distinct dorsal vittae, 2 outer, and 2 inner, these broken along suture. Lateral surfaces of thorax primarily covered in the same silver tomentum as on the dorsal surfaces; all pleura with densely hirsute areas populated with long black setulae becoming long black setulae along posterior margins; chaetotaxy: 3 strong setae on postpronotum arranged in a line; acrostichal setae 3:3; dorsocentral setae 3:4; intra-alar setae 2:3; supra-alar setae 2:3; 4 katepisternal setae; scutellum, with four pairs of long flat marginal setae of subequal length, and one rows of median discal scutellar setae; apical setae present short parallell and erect, at a slight upward angle from the plane of the rest of the scutellar marginal setae. **Wing**, strongly infuscate, with a brilliant orange basicosta; both upper and lower calypters strongly infuscate concolorous with remainder of wing; wing vein R4+5, bearing 3–3 setulae at base; halteres orange stalk and capitulum. **Legs**: black, with yellow pulvilli; Anterodorsal row of setae on hind tibia fringelike, formed by a very regular row of uniformly sized setae separated from each other by less than the width of their sockets. **Abdomen**: slightly flattened globose, brown ground color; bronze abdominal tomentosity along anterior margin of T3, and strikingly yellow on >50% of surface of T4 and all of T5 which; T4 bearing a narrow median black stripe bisecting the yellow band. Middorsal depression on ST1+2 reaching to hind margin of tergite. Median marginal setae present on ST1+2 and T3, and complete rows of setae on T4 and T5.

**Male terminalia** (Fig. 8): sternite 5 with a deeply excavated median cleft along posterior edge, smoothly U-shaped, margins covered in dense tomentum; posterior lobes coming to a rounded point apically, with strong bristle-like setulae surrounded by many shorter weaker setulae. Anterior plate of sternite 5 approximately 1/2 length of posterior lobes; unsclerotized "window" on anterior plate of sternite 5 ranging translucent directly basal to posterior lobes, elongate spanning the entire width of the posterior lobes. Cerci in posterior view triangular/blade-like in appearance, subequal to length of surstyli; completely separate medially. Cerci in lateral view. wide and appearing rounded apically, straight along lower margin with only a very slight anterior projection, not appearing clubbed apically; cerci setose along basal 2/3rds, underside of cerci setose along entire length (visible in lateral profile). Surstylus in lateral view, broadly rounded along its posterior edge giving the structure a leaf or oarlike appearance; surstylus appearing fused with epandrium; when viewed posteriorly surstyli appearing slightly convergent or bearing inward curved apices but not strongly convergent. Pregonite broad, well-developed, apically rounded, somewhat blunt, devoid of setulae. Postgonite, slightly narrowed, 1/3 as wide as pregonite, bluntly rounded with a slight curve at apex, short. Distiphallus broadly cone-shaped (in some species this cone or flare is much more pronounced, in others appearing square or barrel shaped), with a slender median longitudinal sclerotized reinforcement on its posterior surface and a broad, anterolateral, sclerotized acrophallus, on anterior surface near apex, 1.75X as long as basiphallus; epiphallus, short and rounded, appearing as a small hump on dorsal surface of basiphallus.

**Female** (Fig. 9) length: 11–14mm, overall morphology as in male differing in the following traits: **Head**: bearing 2–3 pairs of proclinate orbital setae in addition to single pair of reclinate orbital seta; gena 2/5 of eye height, inner row of 5-10 post-ocular setae; palps follow same general morphology of males, but are apically devoid of black setulae. **Thorax**: katepisternum with 4–5 strong setae; anterodorsal fringe on hind tibia with 3–4 interspersed much longer setae approximately 2x as long as setae of fringe. **Abdomen**: slightly more globose than males.

#### Diagnosis

*Belvosiaanacarballoae* can be distinguished from all other *Belvosia* by the following combination of traits: males without proclinate orbital setae, pilosity of gena, anepisternum, katepisternum black, basicosta brilliant orange, abdomen with dark ground color, median marginal setae present on syntergite 1+2, anterior margin of T3 bearing some minor gold tomentum <10%; gold tomentum on T4 ranging from 20–40% coverage of tergite, gold tomentum of tergites bissected medially by a middorsal stripe of dark tomentum.

#### Etymology

*Belvosiaanacarballoae* **sp. n.**, is named in honor of Sra. Ana Carballo in recognition of her decades of being part of the Parataxonomist Program of Area de Conservación Guanacaste (http://www.acguanacaste.ac.cr) in northwestern Costa Rica. Interim species-specific name included in previously circulating databases and publications, *Belvosia* Woodley02.

#### Distribution

Costa Rica, ACG (Provinces of Alajuela and Guanacaste), 10–1060 m elevation.

#### Ecology

Within the ACG inventory, *Belvosiaanacarballoae* has been reared 468 times from two families of Lepidoptera: Saturniidae, *Automerisbanus* (Boisduval, 1875) (N=11), *A.belti* Druce, 1886 (N=1), *A.celata* Lemaire, 1969 (N=7), *A.dagmarae* Brechlin & Meister, 2011 (N=33), *A.exigua* Lemaire, 1977 (N=12), *A.hamata* Schaus, 1906 (N=4), *A.io*DHJ01 (N=11), *A.pallidior* Draudt, 1929 (N=5), *A.tridens* Herrich-Schäffer, 1855 (N=32), *A.zozimanaguana* Brechlin & Meister, 2011 (N=334), *A.zugana* Druce, 1886 (N=1), *A.zugana*DHJ01 (N=1), *Hylesiacontinua* (Walker, 1865) (N=3), *Molippanibasa* Maassen & Weyding, 1885 (N=10), *M.similima* Jones, 1907 (N=1), *Periphobaarcaei* (Druce, 1886) (N=1); and Sphingidae, *Manducalanguinosa* (Edwards, 1887) (N=1); from cloud forest, dry forest, rain forest and dry-rain lowland intergrade.

### Belvosia angelhernandezi

Fleming & Woodley sp. nov.

54C98B7E-29C6-5CE5-B1E3-57DBAC41A311

2C0A837B-27AA-4026-8AAB-4E409B84B38E

#### Materials

- **Type status:** Holotype. **Occurrence:** occurrenceDetails: http://janzen.sas.upenn.edu; catalogNumber: DHJPAR0001781; recordedBy: D.H. Janzen, W. Hallwachs &Roster Moraga; individualID: DHJPAR0001781; individualCount: 1; sex: male; lifeStage: adult; preparations: pinned; otherCatalogNumbers: HCIC297-05, 99-SRNP-3906, BOLD:AAB8626; occurrenceID: 5BD73A00-E2A1-5BD8-AC6E-F3E10BA5E93E; **Taxon:** scientificName: Belvosiaangelhernandezi; phylum: Arthropoda; class: Insecta; order: Diptera; family: Tachinidae; genus: Belvosia; specificEpithet: angelhernandezi; scientificNameAuthorship: Fleming & Woodley, 2023; **Location:** continent: Central America; country: Costa Rica; countryCode: CR; stateProvince: Guanacaste; county: Sector El Hacha; locality: Area de Conservacion Guanacaste; verbatimLocality: Estacion Los Almendros; verbatimElevation: 290; verbatimLatitude: 11.0323; verbatimLongitude: -85.5278; verbatimCoordinateSystem: Decimal; decimalLatitude: 11.0323; decimalLongitude: -85.5278; **Identification:** identifiedBy: AJ Fleming; dateIdentified: 2022; **Event:** samplingProtocol: Reared from the larvae of the Saturniidae, Hylesiaumbrata; verbatimEventDate: 29-Oct-1999; **Record Level:** language: en; institutionCode: CNC; collectionCode: Insects; basisOfRecord: Pinned Specimen
- **Type status:** Paratype. **Occurrence:** occurrenceDetails: http://janzen.sas.upenn.edu; catalogNumber: DHJPAR0001694; recordedBy: D.H. Janzen, W. Hallwachs &Roster Moraga; individualID: DHJPAR0001694; individualCount: 1; sex: female; lifeStage: adult; preparations: pinned; otherCatalogNumbers: HCIC212-05, 99-SRNP-3997, BOLD:AAB8626; occurrenceID: 01958B1F-4CD8-5ACB-9201-59AE18AD3BD1; **Taxon:** scientificName: Belvosiaangelhernandezi; phylum: Arthropoda; class: Insecta; order: Diptera; family: Tachinidae; genus: Belvosia; specificEpithet: angelhernandezi; scientificNameAuthorship: Fleming & Woodley, 2023; **Location:** continent: Central America; country: Costa Rica; countryCode: CR; stateProvince: Guanacaste; county: Sector El Hacha; locality: Area de Conservacion Guanacaste; verbatimLocality: Estacion Los Almendros; verbatimElevation: 290; verbatimLatitude: 11.0323; verbatimLongitude: -85.5278; verbatimCoordinateSystem: Decimal; decimalLatitude: 11.0323; decimalLongitude: -85.5278; **Identification:** identifiedBy: AJ Fleming; dateIdentified: 2022; **Event:** samplingProtocol: Reared from the larvae of the Saturniidae, Hylesiaumbrata; verbatimEventDate: 12-Nov-1999; **Record Level:** language: en; institutionCode: CNC; collectionCode: Insects; basisOfRecord: Pinned Specimen
- **Type status:** Paratype. **Occurrence:** occurrenceDetails: http://janzen.sas.upenn.edu; catalogNumber: DHJPAR0001782; recordedBy: D.H. Janzen, W. Hallwachs &Roster Moraga; individualID: DHJPAR0001782; individualCount: 1; sex: male; lifeStage: adult; preparations: pinned; otherCatalogNumbers: HCIC298-05, 99-SRNP-3848, BOLD:AAB8626; occurrenceID: B134E37D-9324-5553-B8E3-41141C891E39; **Taxon:** scientificName: Belvosiaangelhernandezi; phylum: Arthropoda; class: Insecta; order: Diptera; family: Tachinidae; genus: Belvosia; specificEpithet: angelhernandezi; scientificNameAuthorship: Fleming & Woodley, 2023; **Location:** continent: Central America; country: Costa Rica; countryCode: CR; stateProvince: Guanacaste; county: Sector El Hacha; locality: Area de Conservacion Guanacaste; verbatimLocality: Estacion Los Almendros; verbatimElevation: 290; verbatimLatitude: 11.0323; verbatimLongitude: -85.5278; verbatimCoordinateSystem: Decimal; decimalLatitude: 11.0323; decimalLongitude: -85.5278; **Identification:** identifiedBy: AJ Fleming; dateIdentified: 2022; **Event:** samplingProtocol: Reared from the larvae of the Saturniidae, Hylesiaumbrata; verbatimEventDate: 03-Nov-1999; **Record Level:** language: en; institutionCode: CNC; collectionCode: Insects; basisOfRecord: Pinned Specimen

#### Description

**Male** (Fig. 10), length: 11–12mm. **Head**: head slightly wider than thorax; vertex 1/3 head width; gena 1/3 of head height, 2/5 of eye height. Fronto-orbital plate light brown–dark yellow in ground color, entirely covered with silver tomentum giving the whole plate a gold sheen transitioning to silver character; ocellar setae absent at most several hair-like setulae present on ocellar triangle; one reclinate orbital seta outside of frontal row; 1–3 small setae anterio to post-ocular setae; two rows of frontal setae, black setulae intermingled with setae, a few light colored yellow setulae extending below lowest frontal seta. Parafacial light yellow in ground color, densely covered in silver tomentum making the entire surface reflective and brilliant gold appearance; bare overall, except for a small number of setulae extending just below lowest frontal setae; facial ridge setose along 1/2–2/3 of its length, with a few sparse hair-like setulae emerging along outer edge of row; gena covered in yellow to reddish yellow setulae. Antenna, pedicel ranging from light brown to dark burnt orange, concolorous with postpedicel; postpedicel burnt orange, 4X as long as pedicel; arista bare distinctly-thickened on basal 4/5 almost to tip. Palps, yellow-orange throughout and densely covered in short black setulae; slightly clubbed, but gradually tapering to a slight point apically. **Thorax**: black ground color, with light gray tomentum throughout, when viewed dorsally tomentum appears thinner postsuturally; scutellum appearing reddish-black to the naked eye, under microscope reddish tomentum becomes apparent when view on an oblique caudal angle; scutum with four dorsal vittae, becoming more evident under certain angles of light, these broken at suture; lateral surface of thorax densely covered in long hair-like setulae, these setulae mostly black on proepimeron, and dorsal half of katepisternum with a few intermingled reddish-yellow hair-like setulae, these turning to mostly reddish yellow on anterior and caudal margin of anepisternum, katepimeron and anepimeron bearing mostly yellow setulae sometimes with a few black setulae; meron with a few yellow setulae intermingled with upper meral setae; chaetotaxy: 3–4 strong setae on postpronotum arranged in a line, acrostichal setae 3:3; dorsocentral setae 3–4:4; intra-alar setae 3:3; supra-alar setae 2:3; 4–5 katepisternal setae; scutellum, with 4–5 pairs of long flat marginal setae of subequal length; apical setae present, short straight and erect, at a slight upward angle from the plane of the rest of the scutellar marginal setae; complete row of scutellar discal setae just posterior to marginal setae. **Wing**: strongly infuscate, slightly orange at wing base, with a brilliant orange basicosta; both upper and lower calypters also infuscate concolorous with remainder of wing; wing vein R_4+5_ setose, bearing only 2–3 setulae at base; halteres orange stalk with dark black/brown capitulum. **Legs**: black overall, coxa on midleg and hindleg with a few reddish-yellow setulae; tarsal claws yellow with black tips, with yellow pulvilli 2/3 length of tarsal claws; anterodorsal row of setae on hind tibia irregularly sized not fringelike. **Abdomen**: globose, with black ground color; abdominal tomentosity dark bronze and sparse on T3 confined to lateral areas, just under resting wings, sparse bronze-gold tomentum along at most 30% of surface of T4 bisected medially by an area devoid of tomentum, densely gold tomentose on 95% of surface of T5 bisected medially by a dorsomedial narrow darkened strip; middorsal depression on ST1+2 reaching to hind margin of tergite, ventrobasally ST1+2 bearing a few light yellow setulae similar to those on thorax; median marginal setae present on ST1+2 and T3, and complete rows of setae on T4 and T5.

**Male terminalia** (Fig. 11) : sternite 5 with a deeply excavated median cleft along posterior edge, smoothly U-shaped, margins covered in dense tomentum; posterior lobes coming to a rounded point apically, with strong bristle-like setulae surrounded by many shorter weaker setulae. Anterior plate of sternite 5 approximately 1/2 length of posterior lobes; unsclerotized "window" on anterior plate of sternite 5 ranging translucent directly basal to posterior lobes, elongate spanning the entire width of the posterior lobes. Cerci in posterior view triangular/blade-like in appearance, subequal to length of surstyli; completely separate medially. Cerci in lateral view. wide and appearing rounded apically, straight along lower margin with only a very slight anterior projection, not appearing clubbed apically; cerci setose along basal 2/3rds, underside of cerci setose along entire length (visible in lateral profile). Surstylus in lateral view, broadly rounded along its posterior edge giving the structure a leaf or oarlike appearance; surstylus appearing fused with epandrium; when viewed posteriorly surstyli appearing slightly convergent or bearing inward curved apices but not strongly convergent. Pregonite broad, well-developed, apically rounded, somewhat blunt, devoid of setulae. Postgonite, slightly narrowed, 1/3 as wide as pregonite, bluntly rounded with a slight curve at apex, short. Distiphallus broadly cone-shaped (in some species this cone or flare is much more pronounced, in others appearing square or barrel shaped), with a slender median longitudinal sclerotized reinforcement on its posterior surface and a broad, anterolateral, sclerotized acrophallus, on anterior surface near apex, ~1.6X as long as basiphallus; epiphallus, short and rounded, appearing as a small hump on dorsal surface of basiphallus.

**Female** (Fig. 12) length: 11–12mm, overall morphology as in male differing in the following traits: **Head**: bearing 3–5 pairs of proclinate orbital setae in addition to single pair of reclinate orbital seta; gena 1/4 of eye height. **Thorax**: scutellum with up to 4–5 pairs marginal scutellar setae although most often similar to males. **Abdomen**: similar to males, differing only in terminalia.

#### Diagnosis

*Belvosiaangelhernandezi* **sp. n.** can be distinguished from all other *Belvosia* by the following combination of traits: fronto-orbital plate and parafacial silver tomentose, pilosity of gena, and lowest frontal setulae reddish-yellow, basicosta brilliant orange, abdomen with dark ground color, median marginal setae present on syntergite 1+2, anterior margin of T3 bearing some no gold tomentum <10%; gold tomentum on T4 ranging from 20–40% coverage of tergite, T5 entirely gold tomentose, gold tomentum of tergites bissected medially by a middorsal stripe of dark tomentum.

#### Etymology

*Belvosiaangelhernandezi* **sp. n.**, is named in honor of Sr. Angel Hernandez in recognition of his decades of being part of the Parataxonomist Program of Area de Conservación Guanacaste (http://www.acguanacaste.ac.cr) in northwestern Costa Rica. Interim species-specific name included in previously circulating databases and publications, *Belvosia* Woodley03A.

#### Distribution

Costa Rica, ACG (Guanacaste Province), 290 m elevation.

#### Ecology

*Belvosiaangelhernandezi* **sp. n.** has been reared 75 times from one species of Lepidoptera in the family Saturniidae, *Hylesiaumbrata* (Schaus, 1911), in dry forest, dry-rain lowland intergrade.

### Belvosia brigittevilchezae

Fleming & Woodley sp. nov.

E1574928-64BF-53B5-B425-68A318EB2E3F

E511EC13-57E2-4311-90D9-4789E80B8F0E

#### Materials

- **Type status:** Holotype. **Occurrence:** occurrenceDetails: http://janzen.sas.upenn.edu; catalogNumber: DHJPAR0024435; recordedBy: D.J. Janzen, W. Hallachs & Leonel Siezar; individualID: DHJPAR0024435; individualCount: 1; sex: male; lifeStage: adult; preparations: pinned; otherCatalogNumbers: ASTAW545-08, 08-SRNP-70056, BOLD:ABY9051; occurrenceID: 405F09D9-27BF-5E9D-A371-A450B12B0CF4; **Taxon:** scientificName: Belvosiabrigittevilchezae; phylum: Arthropoda; class: Insecta; order: Diptera; family: Tachinidae; genus: Belvosia; specificEpithet: brigittevilchezae; scientificNameAuthorship: Fleming & Woodley, 2023; **Location:** continent: Central America; country: Costa Rica; countryCode: CR; stateProvince: Guanacaste; county: Sector Pitilla; locality: Area de Conservacion Guanacaste; verbatimLocality: Manguera; verbatimElevation: 470; verbatimLatitude: 10.9959; verbatimLongitude: -85.3984; verbatimCoordinateSystem: Decimal; decimalLatitude: 10.9959; decimalLongitude: -85.3984; **Identification:** identifiedBy: AJ Fleming; dateIdentified: 2022; **Event:** samplingProtocol: Reared from the larvae of the Saturniidae, Hylesiadalina; verbatimEventDate: 02-Jun-2008; **Record Level:** language: en; institutionCode: CNC; collectionCode: Insects; basisOfRecord: Pinned Specimen
- **Type status:** Paratype. **Occurrence:** occurrenceDetails: http://janzen.sas.upenn.edu; catalogNumber: DHJPAR0001130; recordedBy: D.J. Janzen, W. Hallachs & Elieth Cantillano; individualID: DHJPAR0001130; individualCount: 1; sex: female; lifeStage: adult; preparations: pinned; otherCatalogNumbers: HCIC097-05, 03-SRNP-18486, BOLD:ABY9051; occurrenceID: 72F3A4EE-729C-5E90-A9BF-635F40BC8769; **Taxon:** scientificName: Belvosiabrigittevilchezae; phylum: Arthropoda; class: Insecta; order: Diptera; family: Tachinidae; genus: Belvosia; specificEpithet: brigittevilchezae; scientificNameAuthorship: Fleming & Woodley, 2023; **Location:** continent: Central America; country: Costa Rica; countryCode: CR; stateProvince: Guanacaste; county: Sector Del Oro; locality: Area de Conservacion Guanacaste; verbatimLocality: Quebrada Trigal; verbatimElevation: 290; verbatimLatitude: 11.0268; verbatimLongitude: -85.4955; verbatimCoordinateSystem: Decimal; decimalLatitude: 11.0268; decimalLongitude: -85.4955; **Identification:** identifiedBy: AJ Fleming; dateIdentified: 2022; **Event:** samplingProtocol: Reared from the larvae of the Saturniidae, Hylesiacontinua; verbatimEventDate: 24-Sep-2003; **Record Level:** language: en; institutionCode: CNC; collectionCode: Insects; basisOfRecord: Pinned Specimen
- **Type status:** Paratype. **Occurrence:** occurrenceDetails: http://janzen.sas.upenn.edu; catalogNumber: DHJPAR0001687; recordedBy: D.J. Janzen, W. Hallachs & gusaneros; individualID: DHJPAR0001687; individualCount: 1; sex: male; lifeStage: adult; preparations: pinned; otherCatalogNumbers: HCIC205-05, 01-SRNP-15600, BOLD:ABY9051; occurrenceID: D6C03F96-0A62-5E9A-B1FE-225F3BA1456A; **Taxon:** scientificName: Belvosiabrigittevilchezae; phylum: Arthropoda; class: Insecta; order: Diptera; family: Tachinidae; genus: Belvosia; specificEpithet: brigittevilchezae; scientificNameAuthorship: Fleming & Woodley, 2023; **Location:** continent: Central America; country: Costa Rica; countryCode: CR; stateProvince: Guanacaste; county: Sector Santa Rosa; locality: Area de Conservacion Guanacaste; verbatimLocality: Cafetal; verbatimElevation: 280; verbatimLatitude: 10.8583; verbatimLongitude: -85.6109; verbatimCoordinateSystem: Decimal; decimalLatitude: 10.8583; decimalLongitude: -85.6109; **Identification:** identifiedBy: AJ Fleming; dateIdentified: 2022; **Event:** samplingProtocol: Reared from the larvae of the Saturniidae, Hylesialineata; verbatimEventDate: 01-Sep-2001; **Record Level:** language: en; institutionCode: CNC; collectionCode: Insects; basisOfRecord: Pinned Specimen

#### Description

**Male** (Fig. 13), length: 9–12mm. **Head**: head slightly wider than thorax; vertex 1/3 head width; gena 1/4 of head height, 1/3 of eye height. Fronto-orbital plate light brilliant yellow-gold in ground color, entirely covered with gold tomentum; ocellar setae absent at most several hair-like setulae present on ocellar triangle; one slightly inwardly lateroclinate–reclinate orbital seta outside of frontal row; two rows of frontal setae, black setulae intermingled with setae, a few light colored yellow setulae extending below lowest frontal seta. Parafacial light yellow in ground color, some gold tomentosity up to 50% extending down from fronto-orbital plate, remainder densely covered in silver tomentum making the entire surface reflective and brilliant appearance; setulose along parafacial outside facial ridge, a small number of setulae extending just below lowest frontal setae; facial ridge setose along 2/3 of its length, with numerous yellow-blonde hair-like setulae emerging along outer edge of row; gena covered in yellow setulae. Antenna, pedicel ranging from light brown to dark burnt orange, contrasting with postpedicel; postpedicel black, 4X as long as pedicel; arista bare parallel sided only tapering to a point at tip. Palps, yellow-orange throughout and densely covered in short black setulae; slightly clubbed, but distinctly so, tapering to a slight point apically. **Thorax**: black ground color, with sparse light gray tomentum throughout, when viewed dorsally tomentum appears thinner postsuturally, almost glabrous; scutellum appearing dark brown-black to the naked eye, under microscope bronze tomentum becomes apparent when view on an oblique caudal angle; scutum with five dorsal vittae, one outer pair, one inner pair and one single dorsocentral postsutural becoming more evident under certain angles of light; lateral surface of thorax densely covered in long hair-like setulae, these setulae all reddish yellow but often reddish often with dense long black setulae only on proepimeron, proepisternum and anepisternum; meron with a few yellow setulae intermingled with upper meral setae; chaetotaxy: 3 strong setae on postpronotum arranged in a line, acrostichal setae 3:3; dorsocentral setae 3–4:4; intra-alar setae 2:3; supra-alar setae 2:3; 4–5 katepisternal setae; scutellum, with 4–5 pairs of long flat marginal setae of subequal length; apical setae present, short straight and erect, at a slight upward angle from the plane of the rest of the scutellar marginal setae; 1–2 complete rows of scutellar discal setae just posterior to marginal setae. **Wing**: strongly infuscate, slightly darkened but not orange at wing base, with a brilliant orange basicosta; both upper and lower calypters also infuscate concolorous with remainder of wing; wing vein R_4+5_ setose, bearing only 2–3 setulae at base; halteres orange stalk with dark black/brown capitulum. **Legs**: black overall, coxa on midleg and hindleg with a few reddish-yellow setulae; tarsal claws yellow with black tips, with yellow pulvilli subequal to length of tarsal claws; anterodorsal row of setae on hind tibia irregularly sized not fringelike. **Abdomen**: globose, with black ground color; abdominal tomentosity gold and sparse on T3 confined to anterior 10% of tergite, bronze-gold tomentum along at most 40% of surface of T4 bisected medially by an area devoid of tomentum, densely gold tomentose on 95% of surface of T5 bisected medially by a dorsomedial narrow darkened strip; sparse silver tomentum present ventrally, along tergal margins; middorsal depression on ST1+2 reaching to hind margin of tergite, ventrobasally ST1+2 bearing a few light yellow setulae similar to those on thorax; median marginal setae present on ST1+2 and T3, and complete rows of setae on T4 and T5.

**Male terminalia** (Fig. 14): sternite 5 with a deeply excavated median cleft along posterior edge, vaguely Y-shaped with a distinct shoulder, marginally tomentose; posterior lobes somewhat rounded apically, with multiple strong setulae. Anterior plate of sternite 5 1/2 as longer as posterior lobes; unsclerotized "window" on anterior plate of sternite 5 ranging from translucent directly basal to posterior lobes, sinusoidal in shape almost as a flat rounded "W". Cerci in posterior view triangular, subequal in length to surstyli; slightly rounded at apex, used along basal half separating apically; in lateral view, with a slight arc at apex; densely setose along basal 2/3rds, underside of cerci bare along anterior 1/2. Surstylus in lateral view, slightly arcuate, tapering apically to a sharp point ending in a slightly downcurved apex making the structure appear somewhat scythelike; surstylus appearing to be fused with epandrium; when viewed posteriorly surstyli slightly divergent. Pregonite usually broad, well-developed, apically squared off, blunt, devoid of setulae. Postgonite, slightly narrowed, 1/3 as wide as pregonite, rounded apically, subequal in length to pregonite. Distiphallus flared broadly cone-shaped, with a slender median longitudinal sclerotized reinforcement on its posterior surface and a broad, anterolateral, sclerotized acrophallus, on anterior surface near apex, ~1.5X as long as basiphallus.

**Female** (Fig. 15) length: 9–13mm, overall morphology as in male differing in the following traits: **Head**: bearing 3–4 pairs of proclinate orbital setae in addition to single pair of reclinate orbital setal; lacking any setae in front of post-ocular setae; fronto-orbital plate lacking any gold tomentum with only sparse gray tomentum present, tomentosity so sparse that when viewed from above the fronto-orbital plate can appear glabrous. **Thorax**: dark gray tomentose throughout, brighter on lateral edges of scutum surrounding supraalar setae. Wing: wing surface dark smokey gray, strongly infuscate, lighter than males. **Abdomen**: middorsal stripe on T5 gold often incomplete, in terminalia.

#### Diagnosis

*Belvosiabrigittevilchezae* **sp. n.** can be distinguished from all other *Belvosia* by the following combination of traits: fronto-orbital plate gold tomentose (dull gray in females) with a silver parafacial, pilosity of gena, and lowest frontal setulae yellow; basicosta brilliant orange; pilosity of katepisternum, meron and anepimeron, long and pale; abdomen with dark ground color, median marginal setae present on syntergite 1+2.

#### Etymology

*Belvosiabrigittevilchezae* **sp. n**, is named in honor of Sra. Brigitte Vilchez in recognition of her decades of being part of the Parataxonomist Program of Area de Conservación Guanacaste (http://www.acguanacaste.ac.cr) in northwestern Costa Rica (Janzen and Hallwachs 2011). Interim species-specific name included in previously circulating databases and publications, *Belvosia* Woodley03B.

#### Distribution

Costa Rica, ACG (Guanacaste Province), 10–1150 m elevation.

#### Ecology

*Belvosiabrigittevilchezae* **sp. n.** has been reared 180 times from four species of Lepidoptera in the family Saturniidae, *Automeriszozimanaguana* Brechlin & Mesiter, 2011 (N=1), *Hylesiacontinua* (Walker, 1865) (N=64), *H.dalina*DHJ02 (N=2), *H.lineata* Schaus, 1911 (N=115), in rain forest, dry forest, and dry-rain lowland intergrade.

### Belvosia calixtomoragai

Fleming & Woodley sp. nov.

B052CAB5-9351-5B51-B7BD-2BE97D85E996

A479837F-8E26-443F-8737-D802E7CE5CA1

#### Materials

- **Type status:** Holotype. **Occurrence:** occurrenceDetails: http://janzen.sas.upenn.edu; catalogNumber: DHJPAR0019489; recordedBy: D.H. Janzen, W. Hallwachs & Roberto Espinoza; individualID: DHJPAR0019489; individualCount: 1; sex: male; lifeStage: adult; preparations: pinned; otherCatalogNumbers: ASTAB037-07, 07-SRNP-21030, BOLD:AAA2582; occurrenceID: D2CAA1A4-9AFF-5933-B70E-C07E708E4E4D; **Taxon:** scientificName: Belvosiacalixtomoragai; phylum: Arthropoda; class: Insecta; order: Diptera; family: Tachinidae; genus: Belvosia; specificEpithet: calixtomoragai; scientificNameAuthorship: Fleming & Woodley, 2023; **Location:** continent: Central America; country: Costa Rica; countryCode: CR; stateProvince: Guanacaste; county: Sector Del Oro; locality: Area de Conservacion Guanacaste; verbatimLocality: Quebrada Lajosa; verbatimElevation: 400; verbatimLatitude: 11.0331; verbatimLongitude: -85.4288; verbatimCoordinateSystem: Decimal; decimalLatitude: 11.0331; decimalLongitude: -85.4288; **Identification:** identifiedBy: AJ Fleming; dateIdentified: 2022; **Event:** samplingProtocol: Reared from the larvae of the Saturniidae, Hylesiacontinua; verbatimEventDate: 30-Apr-2007; **Record Level:** language: en; institutionCode: CNC; collectionCode: Insects; basisOfRecord: Pinned Specimen
- **Type status:** Paratype. **Occurrence:** occurrenceDetails: http://janzen.sas.upenn.edu; catalogNumber: DHJPAR0003866; recordedBy: D.H. Janzen, W. Hallwachs & Daniel H. Janzen; individualID: DHJPAR0003866; individualCount: 1; sex: male; lifeStage: adult; preparations: pinned; otherCatalogNumbers: ASBE209-06, 79-SRNP-55C.1,; occurrenceID: 1ED6F1EE-1321-5DD7-8AAD-CBA9C10C8877; **Taxon:** scientificName: Belvosiacalixtomoragai; phylum: Arthropoda; class: Insecta; order: Diptera; family: Tachinidae; genus: Belvosia; specificEpithet: calixtomoragai; scientificNameAuthorship: Fleming & Woodley, 2023; **Location:** continent: Central America; country: Costa Rica; countryCode: CR; stateProvince: Guanacaste; county: Sector Santa Rosa; locality: Area de Conservacion Guanacaste; verbatimLocality: Bosque San Emilio; verbatimElevation: 300; verbatimLatitude: 10.8439; verbatimLongitude: -85.6138; verbatimCoordinateSystem: Decimal; decimalLatitude: 10.8439; decimalLongitude: -85.6138; **Identification:** identifiedBy: AJ Fleming; dateIdentified: 2022; **Event:** samplingProtocol: Reared from the larvae of the Saturniidae, Hylesialineata; verbatimEventDate: 15-Jun-1979; **Record Level:** language: en; institutionCode: CNC; collectionCode: Insects; basisOfRecord: Pinned Specimen
- **Type status:** Paratype. **Occurrence:** occurrenceDetails: http://janzen.sas.upenn.edu; catalogNumber: DHJPAR0019472; recordedBy: D.H. Janzen, W. Hallwachs & Roberto Espinoza; individualID: DHJPAR0019472; individualCount: 1; sex: female; lifeStage: adult; preparations: pinned; otherCatalogNumbers: ASTAB020-07, 07-SRNP-21042, BOLD:AAA2582; occurrenceID: 79632DFB-E225-5ED3-BE44-7069348B91AE; **Taxon:** scientificName: Belvosiacalixtomoragai; phylum: Arthropoda; class: Insecta; order: Diptera; family: Tachinidae; genus: Belvosia; specificEpithet: calixtomoragai; scientificNameAuthorship: Fleming & Woodley, 2023; **Location:** continent: Central America; country: Costa Rica; countryCode: CR; stateProvince: Guanacaste; county: Sector Del Oro; locality: Area de Conservacion Guanacaste; verbatimLocality: Quebrada Lajosa; verbatimElevation: 400; verbatimLatitude: 11.0331; verbatimLongitude: -85.4288; verbatimCoordinateSystem: Decimal; decimalLatitude: 11.0331; decimalLongitude: -85.4288; **Identification:** identifiedBy: AJ Fleming; dateIdentified: 2022; **Event:** samplingProtocol: Reared from the larvae of the Saturniidae, Hylesiacontinua; verbatimEventDate: 14-May-2007; **Record Level:** language: en; institutionCode: CNC; collectionCode: Insects; basisOfRecord: Pinned Specimen

#### Description

**Male** (Fig. 16), length: 9–12mm. **Head**: head slightly wider than thorax; vertex 1/3 head width; gena 1/5 of head height, 1/4 of eye height. Fronto-orbital plate ranging from dull silver or pale gray to less often slightly greenish gold with gold tomentum at most on upper 2/3, with three rows of frontal setae, black hair-like setulae intermingled with setae, with a few light colored yellow setulae extending below lowest frontal seta; ocellar setae absent at most several hair-like setulae present on ocellar triangle; row of 2–10 short strong setae directly anterior to post-ocular row; one slightly inwardly lateroclinate–reclinate orbital seta outside of frontal row; 2–3 rows of frontal setae, black setulae intermingled with setae, and a few light colored yellow setulae extending below lowest frontal seta. Parafacial light yellow in ground color, densely covered in silver tomentum making the entire surface reflective and brilliant appearance; setulose along parafacial outside facial ridge, a small number of setulae extending just below lowest frontal setae; facial ridge setose along 2/3–4/5 of its length, with numerous yellow-yellow hair-like setulae emerging along outer edge of row; gena covered in yellow setulae. Antenna, pedicel burnt orange, contrasting with postpedicel; postpedicel black, 3X as long as pedicel; arista bare gradually tapering to a point at tip. Palps, yellow-orange throughout and densely covered in short black setulae; only slightly clubbed, tapering to a slight point apically, devoid of setulae apically. **Thorax**: black ground color, with light gray tomentum throughout, when viewed dorsally tomentum appears dense and silver postsuturally; scutellum appearing dark brown-black to the naked eye, under microscope dense bronze tomentum becomes apparent when view on an oblique caudal angle; scutum with four dorsal vittae, one outer pair, one inner pair broken at suture; lateral surface of thorax densely covered in long hair-like setulae, these setulae mostly reddish, caudal half of anepimeron densely covered in long black setulae, these turning to mostly reddish yellow on anterior and caudal margin of anepisternum, remainder of surfaces with dense long reddish-yellow setuale and with a few yellow setulae intermingled with upper meral setae; chaetotaxy: 3 strong setae on postpronotum arranged in a line, acrostichal setae 3:3; dorsocentral setae 3–4:4; intra-alar setae 3:3; supra-alar setae 2:3; 4–5 katepisternal setae; scutellum, with 4–5 pairs of long flat marginal setae of subequal length; apical setae present, short straight and erect, at a slight upward angle from the plane of the rest of the scutellar marginal setae; 1 complete row of scutellar discal setae just posterior to marginal setae. **Wing**: strongly infuscate, slightly darkened but not orange at wing base, with a brilliant orange basicosta; both upper and lower calypters also infuscate concolorous with remainder of wing; wing vein R_4+5_ setose, bearing only 2–3 setulae at base; halteres orange stalk with dark black/brown capitulum. **Legs**: black overall, coxa on midleg and hindleg with a few reddish-yellow setulae; tarsal claws yellow with black tips, with orange pulvilli subequal to length of tarsal claws; anterodorsal row of setae on hind tibia irregularly sized not fringelike. **Abdomen**: globose, with black ground color, orange lateroventrally on ST1+2–T4; bronze to gold tomentosity along anterior 10% of T3, gold tomentum along anterior 80% of surface of T4 bisected medially by an area devoid of tomentum, densely gold tomentose throughout T5 reaching to hind margin of tergite; sparse silver tomentum present ventrally, along tergal margins; middorsal depression on ST1+2 reaching to hind margin of tergite, ventrobasally ST1+2 bearing a few light yellow setulae similar to those on thorax; median marginal setae present on ST1+2 and T3, and complete rows of setae on T4 and T5.

**Male terminalia** (Fig. 17): sternite 5 with a deeply excavated median cleft along posterior edge, deep and Y-shaped, margins covered in dense tomentum; posterior lobes, bare and rounded apically, tapering and becoming hirsute with tomentum basally, with multiple bristle-like setulae. Anterior plate of sternite 5, 1/2 length of posterior lobes; unsclerotized "window" on anterior plate of sternite 5, directly basal to posterior lobes, elongate and sinuous, vaguely "w" shaped, spanning almost the entire width of the sternite. Cerci in posterior view triangular, slightly shorter than surstyli; rounded at apex yet slightly pointed, fused along most of their length, only separating along anterior 1/4. Cerci rounded/blunt in lateral view with a very slight anterior curve on apex, giving it a slightly clubbed appearance; cerci densely setose along basal 2/3rds, underside of cerci setose along entire length. Surstylus in lateral view, scythelike ending in a slightly downcurved and tapered apex making the structure appear bladelike; surstylus appearing fused with epandrium; when viewed posteriorly surstyli slightly divergent or with a slight outward curved at apices. Pregonite broad, well-developed, apically squared off or rounded, blunt, devoid of setulae, marginally thickened, heavily sclerotized. Postgonite, slightly narrowed, 1/3 as wide as pregonite, blunt and curved at apex, subequal in length to pregonite. Epiphallus well developed and apically hooked. Distiphallus apically flared, broadly cone-shaped, with a slender median longitudinal sclerotized reinforcement on its posterior surface and a broad, anterolateral, sclerotized acrophallus, on anterior surface near apex, ~1.7X as long as basiphallus.

**Female** (Fig. 18) length: 9–13mm, overall morphology as in male differing in the following traits: **Head**: fronto-orbital plate only dull gray, sometimes appearing devoid of tomentum, bearing 3 pairs of proclinate orbital setae in addition to single pair of reclinate orbital seta. **Thorax**: hair-like setulae of anepisternum entirely black. **Abdomen**: as in the males differing only in terminalia.

#### Diagnosis

*Belvosiacalixtomoragai* **sp. n.** can be distinguished from all other *Belvosia* by the following combination of traits: fronto-orbital with dull gray or silver tomentum (sometimes tomentosity can be sparse as to make the fronto-orbital plate appear yellow, but distinctly not gold) and silver parafacial, pilosity of gena, and lowest frontal setulae yellow, setulae below lowest frontal seta pale yellow, basicosta brilliant orange; pilosity of katepisternum, meron and anepimeron, with mostly black setulae, thorax with only three postsutural acrostichals; abdomen with dark ground color, median marginal setae present on syntergite 1+2, gold tomentum on T4 ranging from covering more than 50% of tergite.

#### Etymology

*Belvosiacalixtomoragai* **sp. n**, is named in honor of Sr. Calixto Moraga in recognition of his decades of being part of the Parataxonomist Program of Area de Conservación Guanacaste (http://www.acguanacaste.ac.cr) in northwestern Costa Rica (Janzen and Hallwachs 2011). Interim species-specific name included in previously circulating databases and publications, *Belvosia* Woodley03C.

#### Distribution

Costa Rica, ACG (Provinces of Alajuela and Guanacaste), 95–1150m elevation.

#### Ecology

*Belvosiacalixtomoragai* **sp. n.** has been reared 33 times from five species of Lepidoptera in the family Saturniidae, *Hylesiacontinua* (Walker, 1865) (N=24), *H.dalina* Schaus, 1911 (N=1), *H.* Janzen22 (N=4), *H.lineata* Schaus, 1911 (N=3), *H.rubrifrons* Druce, 1886 (N=2), in rain forest, dry forest, and dry-rain lowland intergrade.

### Belvosia canalis

Aldrich, 1928

5983D59D-D329-59C4-82BC-3892A1BCE746

#### Materials

- **Type status:** Other material. **Occurrence:** occurrenceDetails: http://janzen.sas.upenn.edu; catalogNumber: DHJPAR0016446; recordedBy: D.H. Janzen, W. Hallwachs & Harry Ramirez; individualID: DHJPAR0016446; individualCount: 1; sex: Male; lifeStage: adult; preparations: pinned; otherCatalogNumbers: ASTAP650-07, 06-SRNP-47724, BOLD:AAA6542; occurrenceID: 550A34C4-A02E-5FF7-ADBF-5FD752220CD1; **Taxon:** scientificName: Belvosiacanalis; phylum: Arthropoda; class: Insecta; order: Diptera; family: Tachinidae; genus: Belvosia; specificEpithet: canalis; scientificNameAuthorship: Aldrich, 1928; **Location:** continent: Central America; country: Costa Rica; countryCode: CR; stateProvince: Guanacaste; county: Sector Cacao; locality: Area de Conservacion Guanacaste; verbatimLocality: Sendero Palmas; verbatimElevation: 675; verbatimLatitude: 10.8964; verbatimLongitude: -85.4737; verbatimCoordinateSystem: Decimal; decimalLatitude: 10.8964; decimalLongitude: -85.4737; **Identification:** identifiedBy: AJ Fleming; dateIdentified: 2022; **Event:** samplingProtocol: Reared from the larvae of the Saturniidae, Automerisanikmeisterae; verbatimEventDate: 21-Nov-2006; **Record Level:** language: en; institutionCode: CNC; collectionCode: Insects; basisOfRecord: Pinned Specimen
- **Type status:** Other material. **Occurrence:** occurrenceDetails: http://janzen.sas.upenn.edu; catalogNumber: DHJPAR0019929; recordedBy: D.H. Janzen, W. Hallwachs & Carolina Cano; individualID: DHJPAR0019929; individualCount: 1; sex: Female; lifeStage: adult; preparations: pinned; otherCatalogNumbers: ASTA1212-07, 07-SRNP-1594, BOLD:AAA6542; occurrenceID: 995575AA-252E-5F2A-8899-092F46D1873C; **Taxon:** scientificName: Belvosiacanalis; phylum: Arthropoda; class: Insecta; order: Diptera; family: Tachinidae; genus: Belvosia; specificEpithet: canalis; scientificNameAuthorship: Aldrich, 1928; **Location:** continent: Central America; country: Costa Rica; countryCode: CR; stateProvince: Alajuela; county: Sector San Cristobal; locality: Area de Conservacion Guanacaste; verbatimLocality: Sendero Vivero; verbatimElevation: 730; verbatimLatitude: 10.8674; verbatimLongitude: -85.3874; verbatimCoordinateSystem: Decimal; decimalLatitude: 10.8674; decimalLongitude: -85.3874; **Identification:** identifiedBy: AJ Fleming; dateIdentified: 2022; **Event:** samplingProtocol: Reared from the larvae of the Saturniidae, Automerispostalbida; verbatimEventDate: 22-Aug-2007; **Record Level:** language: en; institutionCode: CNC; collectionCode: Insects; basisOfRecord: Pinned Specimen
- **Type status:** Other material. **Occurrence:** occurrenceDetails: http://janzen.sas.upenn.edu; catalogNumber: 89-SRNP-141; recordedBy: D.H. Janzen, W. Hallwachs & Carolina Cano; individualID: 89-SRNP-141; individualCount: 1; sex: Male; lifeStage: adult; preparations: pinned; otherCatalogNumbers: 89-SRNP-141; occurrenceID: 0BF1DEA9-2046-5872-BCDB-143196D27FD5; **Taxon:** scientificName: Belvosiacanalis; phylum: Arthropoda; class: Insecta; order: Diptera; family: Tachinidae; genus: Belvosia; specificEpithet: canalis; scientificNameAuthorship: Aldrich, 1928; **Location:** continent: Central America; country: Costa Rica; countryCode: CR; stateProvince: Guanacaste; county: Sector Pitilla; locality: Area de Conservacion Guanacaste; verbatimLocality: Estacion Pitilla; verbatimElevation: 675; verbatimLatitude: 10.989310; verbatimLongitude: -85.425810; verbatimCoordinateSystem: Decimal; decimalLatitude: 10.98931; decimalLongitude: -85.42581; **Identification:** identifiedBy: AJ Fleming; dateIdentified: 2022; **Event:** samplingProtocol: Reared from the larvae of the Saturniidae, Automeris anikmeisteraeDHJ01; verbatimEventDate: Jul-07-1989; **Record Level:** language: en; institutionCode: CNC; collectionCode: Insects; basisOfRecord: Pinned Specimen

#### Description

**Male** (Fig. 19), length: 10–11mm. **Head**: head slightly wider than thorax; vertex 1/4 head width; gena 1/4 of head height, 1/3 of eye height. Fronto-orbital plate gold with slight greenish tinge, can often appear devoid of tomentum around vertex, with two rows of frontal setae, populated with short black hair-like setulae intermingled with setae, with a few dark colored setulae extending below lowest frontal seta; ocellar setae present weak and lateroclinate, somewhat hair-like adjacent to anterior ocellus; orbital setae absent. Parafacial light yellow in ground color, densely covered in silver tomentum but often with a gold sheen, particularly around facial carina, entire surface reflective and brilliant appearance; almost bare along parafacial outside facial ridge, with only a small number of setulae extending just below lowest frontal setae; facial ridge setose along 3/4 of its length, with few reddish yellow hair-like setulae emerging along outer edge of row; gena covered in reddish yellow setulae. Antenna, pedicel black, concolorous with postpedicel; postpedicel black, 4X as long as pedicel; arista bare gradually tapering to a point at tip. Palps, orange throughout and densely covered in short black setulae; only slightly clubbed, tapering to a slight point apically, devoid of setulae apically. Vibrissa approximately 1 pedicel length from facial margin. **Thorax**: black ground color, with light gray tomentum throughout; scutellum ground color light brown almost yellow, distinctly lighter than scutum, under microscope glabrous throughout with bronze tomentum only along margins; scutum with five dorsal vittae, one outer pair, one inner pair broken at suture, and one dorsocentrally appearing postsuturally; lateral surface of thorax densely covered in long hair-like setulae, these setulae all black; chaetotaxy: 3 strong setae on postpronotum arranged in a line, acrostichal setae 3:3; dorsocentral setae 3:4; intra-alar setae 3:3; supra-alar setae 2:3; 4–5 katepisternal setae; scutellum, with 4–5 pairs of long marginal setae of subequal length; apical scutellar setae short, weak and erect, inserted above the plane of the marginal setae; 1 complete row of scutellar discal setae just posterior to marginal setae. **Wing**: strongly infuscate, slightly darkened but not orange at wing base, basicosta black to dark brown with slight accent of orange along caudal edge; both upper and lower calypters also infuscate concolorous with remainder of wing; wing vein R_4+5_ setose, bearing only 2–3 setulae at base; halteres orange stalk with dark black/brown capitulum. **Legs**: black overall, lightly covered in shimmering silver tomentum, coxa on midleg and hindleg covered in black setulae; tarsal claws yellow-orange with black tips, with orange pulvilli subequal to length of tarsal claws; anterodorsal row of setae on hind tibia regularly sized almost fringelike, but with 3–4 longer stronger setae at least 2X as long as others. **Abdomen**: large, flattened globose, with black ground color; tomentum absent from T1+2 and T3, gold tomentum along anterior 40% of surface of T4 becoming more apparent under different angles, bisected medially by an area devoid of tomentum, densely gold tomentose throughout T5 not reaching to hind margin of tergite, black along caudal 10% of tergite, where it is devoid of gold; ventral surfaces of T3–T5 densely hirsute, but no distinct sex-patches present; middorsal depression on ST1+2 reaching to hind margin of tergite; one pair of median marginal setae present on ST1+2 and T3, and complete rows of setae on T4 and T5; T5 devoid of any setulae in the area of gold tomentosity.

**Male terminalia** (Fig. 20): sternite 5 with a deeply excavated median cleft along posterior edge, smoothly Y-shaped, margins covered in dense tomentum; posterior lobes rounded apically, with multiple setulae surrounded by many shorter weaker setulae. Anterior plate of sternite 5, 1/2X length of posterior lobes; unsclerotized "window" on anterior plate of sternite 5, elongate translucent, strongly arcuate convex. Cerci in posterior view triangular, with a sharp shoulder at apex; apically pointed, fused along 2/3 of length. Cerci in lateral view, straight with a mild hook at tip, densely setose along basal 2/3rds. Surstylus in lateral view, oar shaped and rounded pinched basally; surstylus appearing fused with epandrium; when viewed posteriorly surstyli slightly convergent and thickened, reminiscent of a kukri-type knife. Pregonite broad, well-developed, apically squared off, with 3–5 marginal setulae. Postgonite, narrow 1/3 as wide as pregonite, sharply blunt at apex, subequal in length to pregonite. Distiphallus elongate and barrel shaped, median longitudinal sclerotized reinforcement short, appearing as a small process, anterolateral, sclerotized acrophallus, on anterior surface near apex, ~1.7X as long as basiphallus.

**Female** (Fig. 21) length: 11–13mm, overall morphology as in male differing in the following traits: **Head**: fronto-orbital plate dull gray, sometimes appearing devoid of tomentum along vertex, bearing 3–4 pairs of proclinate orbital setae in addition to 1–2 pairs of reclinate orbital seta; profile of head not rounded as in males; vertex 1/3 of head width; palps slightly more clubbed than males **Thorax**: Thoracic chaetotaxy, and tomentum as in males. **Abdomen**: more globose than males, lacking the flattened character, setulae on abdomen not as dense appearing far less hirsute than male abdomen; differing in terminalia, and the gold tomentosity on T4 extending over 40-50% of tergal surface.

#### Diagnosis

*Belvosiacanalis* Aldrich 1928 can be distinguished from all other *Belvosia* by the following combination of traits: fronto-orbital plate with a pale gold bronze tomentum, basicosta partly black/dark brown, gold tomentum on T4 covering at most 50% of tergite, bisected medially by a dark strip so that two distinct tomentose patches on T4 appear separated from T5, T5 entirely gold with a slight blackening around median pair of marginal setae (gold tomentum resumes so that the underside is gold), abdomen slightly flattened with T5 slightly open vaguely exposing the genital capsule.

#### Distribution

From Costa Rica south to Brazil; Costa Rica, ACG (Provinces of Alajuela and Guanacaste), 95–1220 m elevation.

##### Taxon Statement

Interim species-specific name included in previously circulating databases and publications, *Belvosia* Woodley08.

#### Ecology

Within the ACG inventory, *Belvosiacanalis* has been reared 152 times from two families of Lepidoptera Saturniidae: *Automerisanikmeisterae* Brechlin & Meister, 2011 (N=30), *A.banus* (Boisduval, 1875) (N=30), *A.niepelti* Draudt, 1929 (N=9), *A.postalbida* Schaus, 1900 (N=82), and one record Erebidae, *Dysschemajansonis* (Butler, 1870) (N=1); from cloud forest, dry forest, rain forest and dry-rain lowland intergrade.

### Belvosia carolinacanoae

Fleming & Woodley sp. nov.

00BD530B-7F9E-56C5-8387-B428E727E632

81DA3B22-9450-400E-BC7A-BC9DAC647181

#### Materials

- **Type status:** Holotype. **Occurrence:** occurrenceDetails: http://janzen.sas.upenn.edu; catalogNumber: DHJPAR0065397; recordedBy: D.H. Janzen, W. Hallwachs & Dinia Martinez; individualID: DHJPAR0065397; individualCount: 1; sex: Male; lifeStage: adult; preparations: pinned; otherCatalogNumbers: ACGBA11837-21, 20-SRNP-70318, BOLD:AAB8626; occurrenceID: 8B74B807-F957-5A57-A4E1-DEE9C5EFF0F7; **Taxon:** scientificName: Belvosiacarolinacanoae; phylum: Arthropoda; class: Insecta; order: Diptera; family: Tachinidae; genus: Belvosia; specificEpithet: carolinacanoae; scientificNameAuthorship: Fleming & Woodley, 2023; **Location:** continent: Central America; country: Costa Rica; countryCode: CR; stateProvince: Guanacaste; county: Sector Pitilla; locality: Area de Conservacion Guanacaste; verbatimLocality: Medrano; verbatimElevation: 380; verbatimLatitude: 11.016; verbatimLongitude: -85.3805; verbatimCoordinateSystem: Decimal; decimalLatitude: 11.016; decimalLongitude: -85.3805; **Identification:** identifiedBy: AJ Fleming; dateIdentified: 2022; **Event:** samplingProtocol: Reared from the larvae of the Saturniidae, Hylesiadalina; verbatimEventDate: 20-Mar-2020; **Record Level:** language: en; institutionCode: CNC; collectionCode: Insects; basisOfRecord: Pinned Specimen
- **Type status:** Paratype. **Occurrence:** occurrenceDetails: http://janzen.sas.upenn.edu; catalogNumber: DHJPAR0065381; recordedBy: D.H. Janzen, W. Hallwachs & Ricardo Calero; individualID: DHJPAR0065381; individualCount: 1; sex: Male; lifeStage: adult; preparations: pinned; otherCatalogNumbers: ACGBA11821-21, 20-SRNP-70216, BOLD:AAB8626; occurrenceID: 29278A7B-C6AC-58F4-88D7-AB2C27B0C8E3; **Taxon:** scientificName: Belvosiacarolinacanoae; phylum: Arthropoda; class: Insecta; order: Diptera; family: Tachinidae; genus: Belvosia; specificEpithet: carolinacanoae; scientificNameAuthorship: Fleming & Woodley, 2023; **Location:** continent: Central America; country: Costa Rica; countryCode: CR; stateProvince: Guanacaste; county: Sector Pitilla; locality: Area de Conservacion Guanacaste; verbatimLocality: Medrano; verbatimElevation: 380; verbatimLatitude: 11.016; verbatimLongitude: -85.3805; verbatimCoordinateSystem: Decimal; decimalLatitude: 11.016; decimalLongitude: -85.3805; **Identification:** identifiedBy: AJ Fleming; dateIdentified: 2022; **Event:** samplingProtocol: Reared from the larvae of the Saturniidae, Hylesiadalina; verbatimEventDate: 18-Mar-2020; **Record Level:** language: en; institutionCode: CNC; collectionCode: Insects; basisOfRecord: Pinned Specimen
- **Type status:** Paratype. **Occurrence:** occurrenceDetails: http://janzen.sas.upenn.edu; catalogNumber: DHJPAR0019474; recordedBy: D.H. Janzen, W. Hallwachs & Roster Moraga; individualID: DHJPAR0019474; individualCount: 1; sex: Female; lifeStage: adult; preparations: pinned; otherCatalogNumbers: ASTAB022-07, 07-SRNP-21164, BOLD:AAB8626; occurrenceID: E53A2649-064A-51C1-85E8-C54D3A597C0D; **Taxon:** scientificName: Belvosiacarolinacanoae; phylum: Arthropoda; class: Insecta; order: Diptera; family: Tachinidae; genus: Belvosia; specificEpithet: carolinacanoae; scientificNameAuthorship: Fleming & Woodley, 2023; **Location:** continent: Central America; country: Costa Rica; countryCode: CR; stateProvince: Guanacaste; county: Sector Del Oro; locality: Area de Conservacion Guanacaste; verbatimLocality: Puente Mena; verbatimElevation: 280; verbatimLatitude: 11.0456; verbatimLongitude: -85.4574; verbatimCoordinateSystem: Decimal; decimalLatitude: 11.0456; decimalLongitude: -85.4574; **Identification:** identifiedBy: AJ Fleming; dateIdentified: 2022; **Event:** samplingProtocol: Reared from the larvae of the Saturniidae, Hylesiacontinua; verbatimEventDate: 18-May-2007; **Record Level:** language: en; institutionCode: CNC; collectionCode: Insects; basisOfRecord: Pinned Specimen

#### Description

**Male** (Fig. 22), length: 9–10mm. **Head**: head slightly wider than thorax; vertex 1/3 head width; gena 1/4 of head height, 1/3 of eye height. Fronto-orbital plate dull silver to pale gray can appear glabrous, can have hints of greenish gold around frontal setae. with two rows of frontal setae, black hair-like setulae intermingled with setae, with a few black colored setulae extending below lowest frontal seta; ocellar setae absent at most several hair-like setulae present on ocellar triangle; three proclinate orbital setae and one reclinate orbital seta outside of frontal row. Parafacial light yellow in ground color, densely covered in silver tomentum making the entire surface reflective and brilliant appearance; slightly setulose with yellow setulae along parafacial outside facial ridge (lower half), a small number of setulae extending just below lowest frontal setae, these mostly black; facial ridge setose along 3/4 of its length; gena covered in black setulae. Antenna, pedicel burnt orange, contrasting with postpedicel; postpedicel black, 3X as long as pedicel; arista bare gradually tapering to a point at tip. Palps, yellow-orange throughout and densely covered in short black setulae; gradually tapering to a slight point apically, devoid of setulae apically. **Thorax**: black ground color, with light gray tomentum throughout, when viewed dorsally tomentum appears dense and silver postsuturally; scutellum appearing dark brown-black to the naked eye, under microscope light bronze tomentum becomes apparent when view on an oblique caudal angle; scutum with four dorsal vittae, one outer pair, one inner pair broken at suture; lateral surface of thorax densely covered in long hair-like setulae, these setulae all black with the exception of the lowest portion of the katepisternum where the setulae turn to a reddish-brown, katepimeron with a small tuft of yellow yellow setulae; chaetotaxy: 3 strong setae on postpronotum arranged in a line, acrostichal setae 3:4; dorsocentral setae 3:4; intra-alar setae 3:3; supra-alar setae 2:3; 4 katepisternal setae, outer pair extremely strong, more than double the thickness of inner pairscutellum, with 4–5 pairs of long flat marginal setae of subequal length; apical setae present, short straight and erect, at a slight upward angle from the plane of the rest of the scutellar marginal setae; 1 complete row of scutellar discal setae just posterior to marginal setae. **Wing**: strongly infuscate, slightly darkened but not orange at wing base, with a brilliant orange basicosta; both upper and lower calypters also infuscate concolorous with remainder of wing; wing vein R_4+5_ setose, bearing only 2–3 setulae at base; halteres orange stalk with dark black/brown capitulum. **Legs**: black overall, coxa on midleg and hindleg with a few reddish-yellow setulae; tarsal claws yellow with black tips, with orange pulvilli subequal to length of tarsal claws; anterodorsal row of setae on hind tibia regularly sized and fringelike with 2–3 longer setae protruding. **Abdomen**: globose, with dark maroon ground color; bronze to gold tomentosity absent on T3, gold tomentum along anterior 50% of surface of T4 bisected medially by an area devoid of tomentum (only visible on some angles of light), densely gold tomentose throughout T5 reaching to hind margin of tergite, black around insertions of marginal setae; middorsal depression on ST1+2 reaching to hind margin of tergite, ventrobasally ST1+2 bearing a few light yellow setulae similar to those on thorax, no sex patch present; median marginal setae weak almost hair-like on ST1+2, strong on T3, and complete rows of setae on T4 and T5.

**Male terminalia** (Fig. 23) : sternite 5 with a deeply excavated median cleft along posterior edge, widely Y-shaped with a slight shoulder, marginally tomentose; posterior lobes rounded apically, with multiple strong setulae, surrounded by shorter hair-like setulae. Anterior plate of sternite 5, 1/2 as long as posterior lobes; unsclerotized "window" on anterior plate of sternite 5 translucent directly basal to posterior lobes, rectangular in shape, curving slightly upward at tips. Cerci in posterior view triangular, subequal to slightly shorter than surstyli; pointed at apex, fused along basal half, separating apically; in lateral view, with a slight arc at apex; densely setose along basal 3/4ths, underside of cerci bare along anterior 1/2. Surstylus in lateral view, slightly straight, strongly tapering apically to a sharp point ending in a slightly downcurved apex; surstylus appearing to be fused with epandrium; when viewed posteriorly surstyli slightly divergent apically. Pregonite broad, well-developed, apically squared off, blunt, devoid of setulae. Postgonite, slightly narrowed, 1/3 as wide as pregonite, rounded apically, curved, shorter than pregonite. Distiphallus slightly flared, more barrel shaped than cone-shaped, with a short and slender median longitudinal sclerotized reinforcement on its posterior surface and a broad, anterolateral, sclerotized acrophallus, on anterior surface near apex, ~1.6X as long as basiphallus, slight club apically.

**Female** (Fig. 24) length: 10–11mm, overall morphology as in males except in the following character states: three proclinate orbital setae and one reclinate orbital seta outside of frontal row; chaetotaxy: acrostichal setae 4:3–4. Abdomen, slightyl more globose, with dark maroon ground color.

#### Diagnosis

*Belvosiacarolinacanoae* **sp. n.** can be distinguished from all other *Belvosia* by the following combination of traits: fronto-orbital gray tomentose with a silver parafacial, pilosity of gena, and lowest frontal setulae dark, basicosta brilliant orange, abdomen with dark ground color, median marginal setae on syntergite 1+2 weak and hair-like almost absent, T4 bearing gold tomentum at least 10% coverage.

#### Etymology

*Belvosiacarolinacanoae* **sp. n**, is named in honor of Sra. Carolina Cano in recognition of her decades of being part of the Parataxonomist Program of Area de Conservación Guanacaste (http://www.acguanacaste.ac.cr) in northwestern Costa Rica (Janzen and Hallwachs 2011). Interim species-specific name included in previously circulating databases and publications, *Belvosia* Woodley03D.

#### Distribution

Costa Rica, ACG, Guanacaste Province, 280–400 m elevation.

#### Ecology

*Belvosiacarolinacanoae* **sp. n.** has been reared seven times from two species of Lepidoptera in the family Saturniidae, *Hylesiacontinua* (Walker, 1865) (N=2), and *Hylesiadalina* Schaus, 1911 (N=5) dry forest, and dry-rain lowland intergrade.

### Belvosia ciriloumanai

Fleming & Woodley sp. nov.

7637C9A0-839E-510B-B5AF-635755788FB5

5A6B6AC8-3FDE-4C32-AE9C-390B45260830

#### Materials

- **Type status:** Holotype. **Occurrence:** occurrenceDetails: http://janzen.sas.upenn.edu; catalogNumber: DHJPAR0001805; recordedBy: D.H. Janzen, W. Hallwachs & Manuel Pereira; individualID: DHJPAR0001805; individualCount: 1; sex: Male; lifeStage: adult; preparations: pinned; otherCatalogNumbers: HCIC321-05, 03-SRNP-16753, BOLD:AAA1520; occurrenceID: 84CC3820-75C3-53B7-A86D-80A6CDA807B2; **Taxon:** scientificName: Belvosiaciriloumanai; phylum: Arthropoda; class: Insecta; order: Diptera; family: Tachinidae; genus: Belvosia; specificEpithet: ciriloumanai; scientificNameAuthorship: Fleming & Woodley, 2022; **Location:** continent: Central America; country: Costa Rica; countryCode: CR; stateProvince: Guanacaste; county: Sector Del Oro; locality: Area de Conservacion Guanacaste; verbatimLocality: Puente Mena; verbatimElevation: 280; verbatimLatitude: 11.0456; verbatimLongitude: -85.4574; verbatimCoordinateSystem: Decimal; decimalLatitude: 11.0456; decimalLongitude: -85.4574; **Identification:** identifiedBy: AJ Fleming; dateIdentified: 2022; **Event:** samplingProtocol: Reared from the larvae of the Sphingidae, Enyoocypete; verbatimEventDate: 30-Aug-2002; **Record Level:** language: en; institutionCode: CNC; collectionCode: Insects; basisOfRecord: Pinned Specimen
- **Type status:** Paratype. **Occurrence:** occurrenceDetails: http://janzen.sas.upenn.edu; catalogNumber: DHJPAR0002055; recordedBy: D.H. Janzen, W. Hallwachs & Lucia Rios; individualID: DHJPAR0002055; individualCount: 1; sex: Female; lifeStage: adult; preparations: pinned; otherCatalogNumbers: HCIC571-05, 03-SRNP-2804, BOLD:AAA1520; occurrenceID: E7BFABBA-419B-517A-BB7E-43322157DD73; **Taxon:** scientificName: Belvosiaciriloumanai; phylum: Arthropoda; class: Insecta; order: Diptera; family: Tachinidae; genus: Belvosia; specificEpithet: ciriloumanai; scientificNameAuthorship: Fleming & Woodley, 2022; **Location:** continent: Central America; country: Costa Rica; countryCode: CR; stateProvince: Guanacaste; county: Sector Del Oro; locality: Area de Conservacion Guanacaste; verbatimLocality: Quebrada Trigal; verbatimElevation: 290; verbatimLatitude: 11.0268; verbatimLongitude: -85.4955; verbatimCoordinateSystem: Decimal; decimalLatitude: 11.0268; decimalLongitude: -85.4955; **Identification:** identifiedBy: AJ Fleming; dateIdentified: 2022; **Event:** samplingProtocol: Reared from the larvae of the Sphingidae, Enyoocypete; verbatimEventDate: 08-Jul-2003; **Record Level:** language: en; institutionCode: CNC; collectionCode: Insects; basisOfRecord: Pinned Specimen
- **Type status:** Paratype. **Occurrence:** occurrenceDetails: http://janzen.sas.upenn.edu; catalogNumber: DHJPAR0003666; recordedBy: D.H. Janzen, W. Hallwachs & Daniel H. Janzen; individualID: DHJPAR0003666; individualCount: 1; sex: Male; lifeStage: adult; preparations: pinned; otherCatalogNumbers: ASBE009-06, 84-SRNP-490, BOLD:AAA1520; occurrenceID: 332F9858-C1B6-5D9F-9FC8-0A35CFDAF5B6; **Taxon:** scientificName: Belvosiaciriloumanai; phylum: Arthropoda; class: Insecta; order: Diptera; family: Tachinidae; genus: Belvosia; specificEpithet: ciriloumanai; scientificNameAuthorship: Fleming & Woodley, 2022; **Location:** continent: Central America; country: Costa Rica; countryCode: CR; stateProvince: Guanacaste; county: Sector Santa Rosa; locality: Area de Conservacion Guanacaste; verbatimLocality: Bosque San Emilio; verbatimElevation: 300; verbatimLatitude: 10.8439; verbatimLongitude: -85.6138; verbatimCoordinateSystem: Decimal; decimalLatitude: 10.8439; decimalLongitude: -85.6138; **Identification:** identifiedBy: AJ Fleming; dateIdentified: 2022; **Event:** samplingProtocol: Reared from the larvae of the Sphingidae, Enyoocypete; verbatimEventDate: 20-Jul-1984; **Record Level:** language: en; institutionCode: CNC; collectionCode: Insects; basisOfRecord: Pinned Specimen

#### Description

**Male** (Fig. 25), length: 12–14mm. **Head**: head slightly wider than thorax; vertex 1/3 head width; gena 1/4 of head height, 2/5 of eye height. Fronto-orbital plate ranging from dull silver to pale gray often with slight hints of gold, with two rows of frontal setae, black hair-like setulae intermingled with setae, with a few dark colored setulae extending below lowest frontal seta; ocellar setae absent at most several hair-like setulae present on ocellar triangle; orbital setae absent; inner row of 5-10 post-ocular setae. Parafacial light yellow in ground color, densely covered in silver tomentum making the entire surface reflective and brilliant appearance; setulose along parafacial outside facial ridge, a small number of setulae extending just below lowest frontal setae; facial ridge setose along 2/3 of its length, with numerous black hair-like setulae emerging along outer edge of row; gena covered in black setulae. Antenna, pedicel black, concolorous with postpedicel; postpedicel black, 4X as long as pedicel; arista bare gradually tapering to a point at tip. Palps, yellow-orange throughout and densely covered in short black setulae; slightly clubbed, tapering to a slight point apically, devoid of setulae apically. **Thorax**: black ground color, with light gray tomentum throughout presuturally, thinning centrally postsuturally, and transitioning to brown-bronze laterally when viewed from a caudal angle; scutellum appearing dark brown-black to the naked eye, under microscope glabrous adjacent to scutum, abruptly transitioning to dense bronze tomentum which becomes apparent when view on an oblique caudal angle; scutum with four dorsal vittae, one outer pair, one inner pair broken at suture; lateral surface of thorax densely covered in long hair-like setulae, these setulae all black; chaetotaxy: 3–4 strong setae on postpronotum arranged in a line, acrostichal setae 3:3–5; dorsocentral setae 3–4:4; intra-alar setae 3:3; supra-alar setae 2:3; 4–5 katepisternal setae; scutellum, with 5–6 pairs of long flat marginal setae of subequal length; apical setae absent; 1 complete row of scutellar discal setae just posterior to marginal setae. **Wing**: strongly infuscate, slightly darkened but not orange at wing base, basicosta black with slight accent of orange along caudal edge; both upper and lower calypters also infuscate concolorous with remainder of wing; wing vein R_4+5_ setose, bearing only 2–3 setulae at base; halteres orange stalk with dark black/brown capitulum. **Legs**: black overall, covered in shimmering bronze tomentum, coxa on midleg and hindleg covered in black setulae; tarsal claws yellow-orange with black tips, with orange pulvilli subequal to length of tarsal claws; anterodorsal row of setae on hind tibia irregularly sized not fringelike. **Abdomen**: large, flattened globose, with black ground color, brown lateroventrally on ST1+2–T4; bronze to gold tomentosity along anterior 5% of T3 almost not visibly so, only when viewed from a very strong caudal angle, gold tomentum along anterior 40-50% of surface of T4 becoming more apparent under different angles, bisected medially by an area devoid of tomentum, densely gold tomentose throughout T5 not reaching to hind margin of tergite, black along caudal 10% of tergite, where it is devoid of gold; ventral surfaces of T3–T5 densely hirsute, reminiscent of sex-patches present in other Goniini, but lacking any definitive shape or form; middorsal depression on ST1+2 reaching to hind margin of tergite; one pair of median marginal setae present on ST1+2 and T3, and complete rows of setae on T4 and T5; T5 devoid of any setulae in the area of gold tomentosity.

**Male terminalia** (Fig. 26): sternite 5 with a deeply excavated median cleft along posterior edge, roughly Y-shaped, margins covered in dense tomentum; posterior lobes rounded apically, densely covered in multiple long, fine hair-like setulae. Anterior plate of sternite 5, 1/2 length of posterior lobes; unsclerotized "window" on anterior plate of sternite 5 rectangular, nearly transparent directly basal to posterior lobes. Cerci in posterior view variable, with two distinctive shoulders each tapering down by 1/2 previous width, overall triangular, lenght subequal to that of surstyli; blunted triangular at apex, medially to fused along 1/3 of their length. Cerci in lateral view, with a strong anterior curve on apex, giving it a pinched- slightly clubbed appearance; densely setose along most of its length, only bare at apex. Surstylus in lateral view, almost equilateral along its length with a slight curve; surstylus appearing to be separate and not fused with epandrium; when viewed posteriorly surstyli straight, not convergent. Pregonite broad and well developed, apically squared off, blunt, devoid of setulae. Postgonite, slightly narrowed, 1/3 as wide as pregonite, curved at apex, short and scythelike. Distiphallus broadly cone-shaped with a pronounced flare, with a slender median longitudinal sclerotized reinforcement on its posterior surface not reaching apex and a broad, anterolateral, sclerotized acrophallus, thickened apically appearing clubbed, only slightly ~1.8X as long as basiphallus.

**Female** (Fig. 27) length: 12–15mm, overall morphology as in male differing in the following traits: **Head**: fronto-orbital plate dull gray, sometimes appearing devoid of tomentum, bearing 3–4 pairs of proclinate orbital setae in addition to single pair of reclinate orbital seta; postpedicel 2–3X as long as pedicel; gena 1/3 of head height, 1/2 of eye height. **Thorax**: chaetotaxy as in males. **Abdomen**: as in the males differing only in terminalia, overall abdomen not as hirsute as in males, particularly apparent on the underside.

#### Diagnosis

*Belvosiaciriloumanai* **sp. n.** can be distinguished from all other *Belvosia* by the following combination of traits: fronto-orbital plate gray tomentose with a silver parafacial, pilosity of gena, and lowest frontal setulae black, basicosta black-dark brown, both calypters dark infuscate, median marginal setae present on syntergite 1+2, anterior margin of T3 at most with gold tomentum along anterior 5% and T4 with gold tomentum over 10% of tergite, gold tomentum on T5 ending before last marginal setae making the apex of the tergite black, gold tomentum of tergites bissected medially by a middorsal stripe of dark tomentum.

#### Etymology

*Belvosiaciriloumanai* **sp. n**, is named in honor of Sr. Cirilo Umaña in recognition of his decades of being part of the Parataxonomist Program of Area de Conservación Guanacaste (http://www.acguanacaste.ac.cr) in northwestern Costa Rica (Janzen and Hallwachs 2011). Interim species-specific name included in previously circulating databases and publications, *Belvosia* Woodley04A.

#### Distribution

Costa Rica, ACG (Provinces of Alajuela and Guanacaste), 50–740 m elevation.

#### Ecology

*Belvosia* Woodley04A **sp. n.** has been reared 420 times from five species of Lepidoptera in the family Sphingidae, *Aleuroniphis* (Walker, 1856) (N=3), *Enyocavifer* (Rothschild & Jordan, 1903) (N=1), *Enyoocypete* (Linnaeus, 1758) (N=390), *Unzelajapix* (Cramer, 1776) (N=22), *U.pronoe* Druce, 1894 (N=3), in rain forest, dry forest, and dry-rain lowland intergrade.

### Belvosia diniamartinezae

Fleming & Woodley sp. nov.

655349A0-68FF-54E6-894B-D348F5D6676E

92EB6C58-5528-446C-8AA1-B72676E60832

#### Materials

- **Type status:** Holotype. **Occurrence:** occurrenceDetails: http://janzen.sas.upenn.edu; catalogNumber: DHJPAR0003654; recordedBy: D.H. Janzen, W. Hallwachs & Lucia Rios; individualID: DHJPAR0003654; individualCount: 1; sex: Male; lifeStage: adult; preparations: pinned; otherCatalogNumbers: HCIC758-05, 00-SRNP-2670, BOLD:AAA1520; occurrenceID: 3428C3F5-63F4-5603-90D4-2DE37E6E9CF8; **Taxon:** scientificName: Belvosiadiniamartinezae; phylum: Arthropoda; class: Insecta; order: Diptera; family: Tachinidae; genus: Belvosia; specificEpithet: diniamartinezae; scientificNameAuthorship: Fleming & Woodley, 2023; **Location:** continent: Central America; country: Costa Rica; countryCode: CR; stateProvince: Guanacaste; county: Sector El Hacha; locality: Area de Conservacion Guanacaste; verbatimLocality: Estacion Los Almendros; verbatimElevation: 290; verbatimLatitude: 11.0323; verbatimLongitude: -85.5278; verbatimCoordinateSystem: Decimal; decimalLatitude: 11.0323; decimalLongitude: -85.5278; **Identification:** identifiedBy: AJ Fleming; dateIdentified: 2022; **Event:** samplingProtocol: Reared from the larvae of the Sphingidae, Enyoocypete; verbatimEventDate: 10-Jul-2000; **Record Level:** language: en; institutionCode: CNC; collectionCode: Insects; basisOfRecord: Pinned Specimen
- **Type status:** Paratype. **Occurrence:** occurrenceDetails: http://janzen.sas.upenn.edu; catalogNumber: DHJPAR0003648; recordedBy: D.H. Janzen, W. Hallwachs & Lucia Rios; individualID: DHJPAR0003648; individualCount: 1; sex: Female; lifeStage: adult; preparations: pinned; otherCatalogNumbers: HCIC752-05, 00-SRNP-2624, BOLD:AAA1520; occurrenceID: 34EDD49A-7843-56A6-9918-DA6E54D163D7; **Taxon:** scientificName: Belvosiadiniamartinezae; phylum: Arthropoda; class: Insecta; order: Diptera; family: Tachinidae; genus: Belvosia; specificEpithet: diniamartinezae; scientificNameAuthorship: Fleming & Woodley, 2023; **Location:** continent: Central America; country: Costa Rica; countryCode: CR; stateProvince: Guanacaste; county: Sector El Hacha; locality: Area de Conservacion Guanacaste; verbatimLocality: Estacion Los Almendros; verbatimElevation: 290; verbatimLatitude: 11.0323; verbatimLongitude: -85.5278; verbatimCoordinateSystem: Decimal; decimalLatitude: 11.0323; decimalLongitude: -85.5278; **Identification:** identifiedBy: AJ Fleming; dateIdentified: 2022; **Event:** samplingProtocol: Reared from the larvae of the Sphingidae, Enyoocypete; verbatimEventDate: 14-Jul-2000; **Record Level:** language: en; institutionCode: CNC; collectionCode: Insects; basisOfRecord: Pinned Specimen
- **Type status:** Paratype. **Occurrence:** occurrenceDetails: http://janzen.sas.upenn.edu; catalogNumber: DHJPAR0003671; recordedBy: D.H. Janzen, W. Hallwachs & gusaneros; individualID: DHJPAR0003671; individualCount: 1; sex: Male; lifeStage: adult; preparations: pinned; otherCatalogNumbers: ASBE014-06, 88-SRNP-134, BOLD:AAA1520; occurrenceID: 17834835-29BF-58BF-9DB2-BE3C627AF4E6; **Taxon:** scientificName: Belvosiadiniamartinezae; phylum: Arthropoda; class: Insecta; order: Diptera; family: Tachinidae; genus: Belvosia; specificEpithet: diniamartinezae; scientificNameAuthorship: Fleming & Woodley, 2023; **Location:** continent: Central America; country: Costa Rica; countryCode: CR; stateProvince: Guanacaste; county: Sector Santa Rosa; locality: Area de Conservacion Guanacaste; verbatimLocality: Bosque Humedo; verbatimElevation: 290; verbatimLatitude: 10.8514; verbatimLongitude: -85.608; verbatimCoordinateSystem: Decimal; decimalLatitude: 10.8514; decimalLongitude: -85.608; **Identification:** identifiedBy: AJ Fleming; dateIdentified: 2022; **Event:** samplingProtocol: Reared from the larvae of the Sphingidae, Enyoocypete; verbatimEventDate: 06-Jul-1988; **Record Level:** language: en; institutionCode: CNC; collectionCode: Insects; basisOfRecord: Pinned Specimen

#### Description

**Male** (Fig. 28), length: 13–14mm. **Head**: head slightly wider than thorax; vertex 1/3 head width; gena 1/4 of head height, 2/5 of eye height. Fronto-orbital plate ranging dull silver to pale gold, with two distinct rows of frontal setae, densely popuplated with black hair-like setulae intermingled, a few dark colored setulae extending below lowest frontal seta; ocellar setae absent, at most several hair-like setulae present on ocellar triangle; row of 1–3 short strong setae directly anterior to post-ocular row; orbital setae absent. Parafacial light yellow in ground color, densely covered in silver tomentum making the entire surface reflective and brilliant appearance; setulose along parafacial outside facial ridge; facial ridge setose along 3/4 of its length, with numerous black hair-like setulae emerging along outer edge; gena covered in strong black setulae. Antenna, pedicel black, concolorous with postpedicel; postpedicel black, 4X as long as pedicel; arista bare gradually tapering to a point at tip. Palps, yellow-orange throughout and densely covered in short black setulae; slender and near equilateral, only slightly curved at apex but not clubbed, tapering to a slight point apically, devoid of setulae basally. **Thorax**: black ground color, with light gray tomentum throughout presuturally, thinning centrally postsuturally, and transitioning to brown-bronze laterally when viewed from a caudal angle; scutellum appearing dark brown-black to the naked eye, under microscope glabrous adjacent to scutum, abruptly transitioning to dense bronze tomentum which becomes apparent when view on an oblique caudal angle; scutum with four dorsal vittae, one outer pair, one inner pair broken at suture; lateral surface of thorax densely covered in long hair-like setulae, these setulae all black; chaetotaxy: 3–4 strong setae on postpronotum arranged in a line, acrostichal setae 3:3–5; dorsocentral setae 3–4:4; intra-alar setae 3:3; supra-alar setae 2:3; 4–5 katepisternal setae; scutellum, with 5–6 pairs of long flat marginal setae of subequal length; apical setae absent; 1 complete row of scutellar discal setae just posterior to marginal setae. **Wing**: strongly infuscate, slightly darkened but not orange at wing base, basicosta black with slight accent of orange along caudal edge; both upper and lower calypters also infuscate concolorous with remainder of wing; wing vein R_4+5_ setose, bearing only 2–3 setulae at base; halteres orange stalk with dark black/brown capitulum. **Legs**: black overall, covered in shimmering bronze tomentum, coxa on midleg and hindleg covered in black setulae; tarsal claws yellow-orange with black tips, and orange pulvilli subequal to length of tarsal claws; anterodorsal row of setae on hind tibia irregularly sized not fringelike. **Abdomen**: large, flattened globose, with black ground color, brown lateroventrally on ST1+2–T4; bronze to gold tomentosity along anterior 5% of T3 almost not visibly so, only when viewed from a very strong caudal angle, gold tomentum along anterior 40-50% of surface of T4 becoming more apparent under different angles, bisected medially by an area devoid of tomentum, densely gold tomentose throughout T5 not reaching to hind margin of tergite, black along caudal 10% of tergite, where it is devoid of gold; "sex patch" present on ventral surfaces of T3–T5 which are densely hirsute, but lacking any definitive shape or form; one pair of median marginal setae present on ST1+2 and T3, and complete rows of setae on T4 and T5; T5 devoid of any setulae in the area of gold tomentosity.

**Male terminalia** (Fig. 29): sternite 5 with a deeply excavated median cleft along posterior edge, roughly Y-shaped, however shoulders lack definition making almost V-shaped, margins covered in dense tomentum; posterior lobes rounded apically, densely covered in multiple long, fine hair-like setulae. Anterior plate of sternite 5 1/2 length of posterior lobes; unsclerotized "window" on anterior plate of sternite 5 rectangular, nearly transparent directly basal to posterior lobes. Cerci in posterior view, overall triangular, slightly longer than surstyli; blunted triangular at apex, medially fused, separating only along anterior 1/3 of their length. Cerci in lateral view, with a strong anterior curve on apex, giving it a pinched- slightly clubbed appearance; densely setose along almost its length, only bare at apex. Surstylus in lateral view, equilateral along its length straight, only slightly curved digitiform; surstylus appearing to be separate and not fused with epandrium; when viewed posteriorly surstyli straight, not convergent. Pregonite broad and well developed, apically squared off, blunt, with a spars margin of 3–5 setulae. Postgonite, slightly narrowed, 1/3 as wide as pregonite, curved at apex, short and scythelike. Distiphallus broadly cone-shaped with a pronounced flare, with a slender median longitudinal sclerotized reinforcement on its posterior surface not reaching apex and a broad, anterolateral, sclerotized acrophallus, thickened apically appearing clubbed, ~1.8X as long as basiphallus.

**Female** (Fig. 30) length: 13–15mm, overall morphology as in male differing in the following traits: **Head**: fronto-orbital plate dull gray, sometimes appearing devoid of tomentum, bearing 3–4 pairs of proclinate orbital setae in addition to single pair of reclinate orbital seta; postpedicel 2–3X as long as pedicel; gena 1/4 head height, and 1/3 of eye height. **Thorax**: chaetotaxy as in males. **Abdomen**: no apparent sex patch present, remainder as in the males differing only in terminalia, overall abdomen not as hirsute as in males, particularly apparent on the underside.

#### Diagnosis

*Belvosiadiniamartinezae* **sp. n.** can be distinguished from all other *Belvosia* by the following combination of traits: gena with black setae, male fronto-orbital plate with traces of gold, males with 1–2 small setulae directly anterior to postocular row, 3–5 in females, wings with black basicosta, base of scutum with a regular row of strong of marginal setae, abdomen T3 with traces of gold tomentum directly adjacent to ST1+2.

#### Etymology

*Belvosiadiniamartinezae* **sp. n**, is named in honor of Sra. Dinia Martinez in recognition of her decades of being part of the Parataxonomist Program of Area de Conservación Guanacaste (http://www.acguanacaste.ac.cr) in northwestern Costa Rica (Janzen and Hallwachs 2011). Interim species-specific name included in previously circulating databases and publications, *Belvosia* Woodley04B.

#### Distribution

Costa Rica, ACG (Provinces of Alajuela and Guanacaste), 7–675 m elevation.

#### Ecology

*Belvosiadiniamartinezae* **sp. n.** has been reared 107 times from three species of Lepidoptera in the family Sphingidae, *Enyolugubris* (Linnaeus, 1771) (N=3), *Enyoocypete* (Linnaeus, 1758) (N=99), *Unzelajapix* (Cramer, 1776) (N=6), in rain forest, dry forest, and dry-rain lowland intergrade.

### Belvosia duniagarciae

Fleming & Woodley sp. nov.

523F4A8C-7334-55CB-8D7A-0267E176632E

23F8832B-C81F-43D5-A7AC-579D193B0248

#### Materials

- **Type status:** Holotype. **Occurrence:** occurrenceDetails: http://janzen.sas.upenn.edu; catalogNumber: DHJPAR0002009; recordedBy: D.H. Janzen, W. Hallwachs & Carolina Cano; individualID: DHJPAR0002009; individualCount: 1; sex: Male; lifeStage: adult; preparations: pinned; otherCatalogNumbers: HCIC525-05, 02-SRNP-20277, BOLD:AAA1520; occurrenceID: 27E463FF-0B63-535D-BE7F-E4D56C3E848A; **Taxon:** scientificName: Belvosiaduniagarciae; phylum: Arthropoda; class: Insecta; order: Diptera; family: Tachinidae; genus: Belvosia; specificEpithet: duniagarciae; scientificNameAuthorship: Fleming & Woodley, 2023; **Location:** continent: Central America; country: Costa Rica; countryCode: CR; stateProvince: Alajuela; county: Sector San Cristobal; locality: Area de Conservacion Guanacaste; verbatimLocality: Potrero Argentina; verbatimElevation: 520; verbatimLatitude: 10.8902; verbatimLongitude: -85.388; verbatimCoordinateSystem: Decimal; decimalLatitude: 10.8902; decimalLongitude: -85.388; **Identification:** identifiedBy: AJ Fleming; dateIdentified: 2022; **Event:** samplingProtocol: Reared from the larvae of the Sphingidae, Unzelajapix; verbatimEventDate: 17-Jan-2003; **Record Level:** language: en; institutionCode: CNC; collectionCode: Insects; basisOfRecord: Pinned Specimen
- **Type status:** Paratype. **Occurrence:** occurrenceDetails: http://janzen.sas.upenn.edu; catalogNumber: DHJPAR0002026; recordedBy: D.H. Janzen, W. Hallwachs & Lucia Rios; individualID: DHJPAR0002026; individualCount: 1; sex: Male; lifeStage: adult; preparations: pinned; otherCatalogNumbers: HCIC542-05, 99-SRNP-2090.01, BOLD:AAA1520; occurrenceID: 7846E08A-2B45-59F2-B255-996357DD00FD; **Taxon:** scientificName: Belvosiaduniagarciae; phylum: Arthropoda; class: Insecta; order: Diptera; family: Tachinidae; genus: Belvosia; specificEpithet: duniagarciae; scientificNameAuthorship: Fleming & Woodley, 2023; **Location:** continent: Central America; country: Costa Rica; countryCode: CR; stateProvince: Guanacaste; county: Sector Orosi; locality: Area de Conservacion Guanacaste; verbatimLocality: Maderos; verbatimElevation: 510; verbatimLatitude: 11.0049; verbatimLongitude: -85.4749; verbatimCoordinateSystem: Decimal; decimalLatitude: 11.0049; decimalLongitude: -85.4749; **Identification:** identifiedBy: AJ Fleming; dateIdentified: 2022; **Event:** samplingProtocol: Reared from the larvae of the Sphingidae, Unzelajapix; verbatimEventDate: 01-Mar-1999; **Record Level:** language: en; institutionCode: CNC; collectionCode: Insects; basisOfRecord: Pinned Specimen
- **Type status:** Paratype. **Occurrence:** occurrenceDetails: http://janzen.sas.upenn.edu; catalogNumber: DHJPAR0001819; recordedBy: D.H. Janzen, W. Hallwachs & Elieth Cantillano; individualID: DHJPAR0001819; individualCount: 1; sex: Female; lifeStage: adult; preparations: pinned; otherCatalogNumbers: HCIC335-05, 02-SRNP-16709, BOLD:AAA1520; occurrenceID: 75D4D5EE-754F-54C2-AB5A-5F8080046165; **Taxon:** scientificName: Belvosiaduniagarciae; phylum: Arthropoda; class: Insecta; order: Diptera; family: Tachinidae; genus: Belvosia; specificEpithet: duniagarciae; scientificNameAuthorship: Fleming & Woodley, 2023; **Location:** continent: Central America; country: Costa Rica; countryCode: CR; stateProvince: Guanacaste; county: Sector El Hacha; locality: Area de Conservacion Guanacaste; verbatimLocality: Finca Araya; verbatimElevation: 295; verbatimLatitude: 11.0154; verbatimLongitude: -85.5113; verbatimCoordinateSystem: Decimal; decimalLatitude: 11.0154; decimalLongitude: -85.5113; **Identification:** identifiedBy: AJ Fleming; dateIdentified: 2022; **Event:** samplingProtocol: Reared from the larvae of the Sphingidae, Enyoocypete; verbatimEventDate: 04-Aug-2002; **Record Level:** language: en; institutionCode: CNC; collectionCode: Insects; basisOfRecord: Pinned Specimen

#### Description

**Male** (Fig. 31), length: 11–14mm. **Head**: head slightly wider than thorax; vertex 1/3 head width; gena 1/4 of head height, 1/3 of eye height; ocellar setae absent at most several hair-like setulae present on ocellar triangle; row of 2–10 short strong setae directly anterior to post-ocular row; fronto-orbital plate ranging from dull silver or pale gray to slightly greenish gold on upper 2/3, with 2–3 rows of irregular frontal setae, black hair-like setulae intermingled with setae, with a few dark setulae extending below lowest frontal seta; one reclinate orbital seta outside of frontal row; parafacial bare and silver, nearly 1/2 of eye width when viewed laterally; facial ridge setulose along 2/3–4/5 of its length, with a few sparse black hair-like setulae along outer edge of row; gena covered in yellow to reddish black setulae; pedicel black concolorous with postpedicel; postpedicel dark brown to black, 2X as long as pedicel; arista bare distinctly-thickened on basal 4/5 almost to tip. Palps, yellow-orange throughout and densely covered in short black setulae; slender and near equilateral, only slightly curved at apex but not clubbed, tapering to a slight point apically, devoid of setulae basally. **Thorax**: black ground color, with light gray tomentum throughout presuturally, thinning centrally postsuturally, and transitioning to brown-bronze laterally when viewed from a caudal angle; scutellum appearing dark brown-black to the naked eye, under microscope glabrous adjacent to scutum, abruptly transitioning to dense bronze tomentum which becomes apparent when view on an oblique caudal angle; scutum with four dorsal vittae, one outer pair, one inner pair broken at suture; lateral surface of thorax densely covered in long hair-like setulae, these setulae all black; chaetotaxy: 3–4 strong setae on postpronotum arranged in a line, acrostichal setae 3:3–5; dorsocentral setae 3–4:4; intra-alar setae 3:3; supra-alar setae 2:3; 4–5 katepisternal setae; scutellum, with 5–6 pairs of long flat marginal setae of subequal length; apical setae absent; 1 complete row of scutellar discal setae just posterior to marginal setae. **Wing**: strongly infuscate, slightly darkened but not orange at wing base, basicosta black with slight accent of orange along caudal edge; both upper and lower calypters also infuscate concolorous with remainder of wing; wing vein R_4+5_ setose, bearing only 2–3 setulae at base; halteres orange stalk with dark black/brown capitulum. **Legs**: black overall, covered in shimmering bronze tomentum, coxa on midleg and hindleg covered in black setulae; tarsal claws yellow-orange with black tips, and orange pulvilli subequal to length of tarsal claws; anterodorsal row of setae on hind tibia irregularly sized not fringelike. **Abdomen**: large, flattened globose, with black ground color, brown–black lateroventrally on ST1+2–T4; gold tomentum only present along anterior 80–90% of surface of T5, bisected medially by an area devoid of tomentum, densely gold tomentose throughout T5 not reaching to hind margin of tergite, black along caudal 10% of tergite, where it is devoid of gold; "sex patch" present on ventral surfaces of T3–T5 which are densely hirsute, but lacking any definitive shape or form; one pair of median marginal setae present on ST1+2; 1–2 pairs present on T3, and complete rows of setae on T4 and T5; T5 devoid of any setulae in the area of gold tomentosity.

**Male terminalia** (Fig. 32): sternite 5 with a deeply excavated median cleft along posterior edge, roughly V-shaped, margins covered in dense tomentum; posterior lobes rounded apically, densely covered in multiple long, fine hair-like setulae. Anterior plate of sternite 5, 1/2 length of posterior lobes; unsclerotized "window" on anterior plate of sternite 5 shaped like a flattened "w", nearly transparent directly basal to posterior lobes. Cerci in posterior view, elongated triangular, slightly longer than surstyli, pointed at apex with only a slight shoulder, medially fused, separating only along anterior 1/3 of their length. Cerci in lateral view, over all anteriorly curved, more acutely at apex, making them appear almost like an incomplete hook; densely setose along almost 2/3 of its length, only bare at apex. Surstylus in lateral view, equilateral along its length with soft but continuous curve, vaguely digitiform; surstylus appearing to be fused with epandrium; when viewed posteriorly surstyli straight, tips slightly divergent. Pregonite broad and well developed, apically squared off, blunt, devoid of setulae. Postgonite, slightly narrowed, 1/3 as wide as pregonite, curved at apex, short and scythelike. Distiphallus broadly cone-shaped with a pronounced flare, with a slender median longitudinal sclerotized reinforcement on its posterior surface not reaching apex and a broad, anterolateral, sclerotized acrophallus, thickened apically appearing clubbed, ~1.3X as long as basiphallus.

**Female** (Fig. 33) length: 11–14mm, overall morphology as in male differing in the following traits: **Head**: fronto-orbital plate dull gray, sometimes appearing devoid of tomentum, bearing 3 pairs of proclinate orbital setae in addition to single pair of reclinate orbital seta; row of setae directly anterior to post-ocular row absent. Palps, slender and only slightly curved at apex but not clubbed, tapering apically, devoid of setulae basally. **Thorax**: chaetotaxy as in males. **Abdomen**: as in the males differing only in terminalia.

#### Diagnosis

*Belvosiaduniagarciae* **sp. n.** can be distinguished from all other *Belvosia* by the following combination of traits: fronto-orbital plate pale silver gray, gena 1/3 of eye height, with a row of 5–10 small setulae directly anterior to postocular row, post sutural scutum mostly silver, both calypters dark, black basicosta, and apex of T5 black tomentose.

#### Etymology

*Belvosiaduniagarciae* **sp. n**, is named in honor of Sr. Adrian Guadamuz in recognition of his decades of being part of the Parataxonomist Program of Area de Conservación Guanacaste (http://www.acguanacaste.ac.cr) in northwestern Costa Rica (Janzen and Hallwachs 2011). Interim species-specific name included in previously circulating databases and publications, *Belvosia* Woodley04C.

#### Distribution

Costa Rica, ACG (Provinces of Alajuela and Guanacaste), 90–710 m elevation.

#### Ecology

*Belvosiaduniagarciae* **sp. n.** has been reared 126 times from four species of Lepidoptera in the family Sphingidae, *Aleuroniphis* (Walker, 1856) (N=1), *Enyoocypete* (Linnaeus, 1758) (N=32), *Unzelajapix* (Cramer, 1776) (N=91), *U.pronoe* Druce, 1894 (N=2), in rain forest, dry forest, and dry-rain lowland intergrade.

### Belvosia duvalierbricenoi

Fleming & Woodley sp. nov.

5333E906-6D10-518D-8841-C9B88A0C3579

2146FEAE-7B48-413A-8CEF-1A2D82A10970

#### Materials

- **Type status:** Holotype. **Occurrence:** occurrenceDetails: http://janzen.sas.upenn.edu; catalogNumber: DHJPAR0001725; recordedBy: D.H. Janzen, W. Hallwachs & gusaneros; individualID: DHJPAR0001725; individualCount: 1; sex: Male; lifeStage: adult; preparations: pinned; otherCatalogNumbers: HCIC243-05, 00-SRNP-8878, BOLD:ABZ6042; occurrenceID: 26A7F525-B361-5D60-8E9E-8DE0833B2508; **Taxon:** scientificName: Belvosiaduvalierbricenoi; phylum: Arthropoda; class: Insecta; order: Diptera; family: Tachinidae; genus: Belvosia; specificEpithet: duvalierbricenoi; scientificNameAuthorship: Fleming & Woodley, 2023; **Location:** continent: Central America; country: Costa Rica; countryCode: CR; stateProvince: Guanacaste; county: Sector Santa Rosa; locality: Area de Conservacion Guanacaste; verbatimLocality: Cuesta Canyon Tigre; verbatimElevation: 270; verbatimLatitude: 10.817; verbatimLongitude: -85.6437; verbatimCoordinateSystem: Decimal; decimalLatitude: 10.817; decimalLongitude: -85.6437; **Identification:** identifiedBy: AJ Fleming; dateIdentified: 2022; **Event:** samplingProtocol: Reared from the larvae of the Sphingidae, Eumorphasatellitia; verbatimEventDate: 24-Jul-2000; **Record Level:** language: en; institutionCode: CNC; collectionCode: Insects; basisOfRecord: Pinned Specimen
- **Type status:** Paratype. **Occurrence:** occurrenceDetails: http://janzen.sas.upenn.edu; catalogNumber: DHJPAR0001716; recordedBy: D.H. Janzen, W. Hallwachs & Minor Carmona; individualID: DHJPAR0001716; individualCount: 1; sex: Female; lifeStage: adult; preparations: pinned; otherCatalogNumbers: HCIC234-05, 04-SRNP-40980, BOLD:ABZ6042; occurrenceID: 8E864A45-64E7-555E-8DBA-574A20B91D0F; **Taxon:** scientificName: Belvosiaduvalierbricenoi; phylum: Arthropoda; class: Insecta; order: Diptera; family: Tachinidae; genus: Belvosia; specificEpithet: duvalierbricenoi; scientificNameAuthorship: Fleming & Woodley, 2023; **Location:** continent: Central America; country: Costa Rica; countryCode: CR; stateProvince: Alajuela; county: Sector Rincon Rain Forest; locality: Area de Conservacion Guanacaste; verbatimLocality: Camino Rio Francia; verbatimElevation: 410; verbatimLatitude: 10.9043; verbatimLongitude: -85.2865; verbatimCoordinateSystem: Decimal; decimalLatitude: 10.9043; decimalLongitude: -85.2865; **Identification:** identifiedBy: AJ Fleming; dateIdentified: 2022; **Event:** samplingProtocol: Reared from the larvae of the Sphingidae, Pachygonidiadrucei; verbatimEventDate: 12-Jun-2004; **Record Level:** language: en; institutionCode: CNC; collectionCode: Insects; basisOfRecord: Pinned Specimen
- **Type status:** Paratype. **Occurrence:** occurrenceDetails: http://janzen.sas.upenn.edu; catalogNumber: DHJPAR0002044; recordedBy: D.H. Janzen, W. Hallwachs & Daniel H. Janzen; individualID: DHJPAR0002044; individualCount: 1; sex: Male; lifeStage: adult; preparations: pinned; otherCatalogNumbers: HCIC560-05, 84-SRNP-776, BOLD:ABZ6042; occurrenceID: C5006322-8F95-5B02-8DAF-D10B78559A21; **Taxon:** scientificName: Belvosiaduvalierbricenoi; phylum: Arthropoda; class: Insecta; order: Diptera; family: Tachinidae; genus: Belvosia; specificEpithet: duvalierbricenoi; scientificNameAuthorship: Fleming & Woodley, 2023; **Location:** continent: Central America; country: Costa Rica; countryCode: CR; stateProvince: Guanacaste; county: Sector Santa Rosa; locality: Area de Conservacion Guanacaste; verbatimLocality: Cortafuegos Naranjo; verbatimElevation: 285; verbatimLatitude: 10.8352; verbatimLongitude: -85.6248; verbatimCoordinateSystem: Decimal; decimalLatitude: 10.8352; decimalLongitude: -85.6248; **Identification:** identifiedBy: AJ Fleming; dateIdentified: 2022; **Event:** samplingProtocol: Reared from the larvae of the Sphingidae, Eumorphasatellitia; verbatimEventDate: 28-Jul-1984; **Record Level:** language: en; institutionCode: CNC; collectionCode: Insects; basisOfRecord: Pinned Specimen

#### Description

**Male** (Fig. 34), length: 12–15mm. **Head**: head slightly wider than thorax; vertex 1/3 head width; gena 1/4 of head height, 2/5 of eye height; ocellar setae absent at most several hair-like setulae present on ocellar triangle; fronto-orbital plate ranging from dull silver or pale gray to slightly greenish gold, with 2–3 rows of irregular frontal setae, black hair-like setulae intermingled with setae, with a few dark setulae extending below lowest frontal seta; one reclinate orbital seta outside of frontal row; parafacial bare and silver, nearly 1/2 of eye width when viewed laterally; facial ridge setulose along 2/3–4/5 of its length, with a few sparse black hair-like setulae along outer edge of row; gena covered in reddish-black; pedicel black concolorous with postpedicel; postpedicel dark brown to black, almost 3X as long as pedicel; arista bare distinctly-thickened on basal 4/5 almost to tip. Palps, yellow-orange throughout and densely covered in short black setulae; slender at base teardrop shaped at apex but not clubbed, tapering to a slight point apically, devoid of setulae basally. **Thorax**: black ground color, with light gray tomentum throughout presuturally, thinning centrally postsuturally, and transitioning to brown-bronze laterally when viewed from a caudal angle; scutellum appearing dark brown-black to the naked eye, under microscope glabrous adjacent to scutum, abruptly transitioning to dense bronze tomentum which becomes apparent when view on an oblique caudal angle; scutum with four dorsal vittae, one outer pair, one inner pair broken at suture; lateral surface of thorax densely covered in long hair-like setulae, these setulae all black; chaetotaxy: 3–4 strong setae on postpronotum arranged in a line, acrostichal setae 3:3–5; dorsocentral setae 3–4:4; intra-alar setae 3:3; supra-alar setae 2:3; 4–5 katepisternal setae; scutellum, with 5–6 pairs of long flat marginal setae of subequal length; apical setae absent; 1 complete row of scutellar discal setae just posterior to marginal setae. **Wing**: strongly infuscate, slightly darkened but not orange at wing base, basicosta black with slight accent of orange along caudal edge; both upper and lower calypters also infuscate concolorous with remainder of wing; wing vein R_4+5_ setose, bearing only 2–3 setulae at base; halteres orange stalk with dark black/brown capitulum. **Legs**: black overall, covered in shimmering bronze tomentum, coxa on midleg and hindleg covered in black setulae; tarsal claws yellow-orange with black tips, and orange pulvilli subequal to length of tarsal claws; anterodorsal row of setae on hind tibia irregularly sized not fringelike. **Abdomen**: large, flattened globose, with black ground color, brown–black lateroventrally on ST1+2–T4; gold tomentum along anterior 10% of T4, and anterior 60–70% of surface of T5, bisected medially by an area devoid of tomentum, T5 black along caudal 30% of tergite, where it is devoid of gold; "sex patch" present on ventral surfaces of T3–T4 which are densely hirsute, but lacking any definitive shape or form; one pair of median marginal setae present on ST1+2, 1–2 pairs present on T3, and complete rows of setae on T4 and T5; T5 devoid of any setulae in the area of gold tomentosity.

**Male terminalia** (Fig. 35) : sternite 5 with a deeply excavated median cleft along posterior edge, roughly V-shaped, margins covered in dense tomentum; posterior lobes rounded apically, densely covered in multiple long, strong setae, surrounded by shorter hair-like setulae. Anterior plate of sternite 5, 1/2 length of posterior lobes; unsclerotized "window" on anterior plate of sternite 5 shaped like a flattened "w", nearly transparent directly basal to posterior lobes. Cerci in posterior view, elongated triangular 2X as long as wide, slightly longer than surstyli, pointed at apex with only a slight shoulder, medially fused, separating only along anterior 1/3 of their length. Cerci in lateral view, over all anteriorly curved, more acutely at apex, making them appear almost like an incomplete hook; densely setose along almost 2/3 of its length, only bare at apex. Surstylus in lateral view, equilateral along its length with soft but continuous curve, vaguely digitform; surstylus appearing to be fused with epandrium; when viewed posteriorly surstyli straight, tips slightly divergent. Pregonite broad and well developed, apically squared off, blunt, devoid of setulae. Postgonite, slightly narrowed, 1/3 as wide as pregonite, curved at apex, short and scythelike. Distiphallus broadly cone-shaped with a pronounced flare, with a slender median longitudinal sclerotized reinforcement on its posterior surface not reaching apex and a broad, anterolateral, sclerotized acrophallus, thickened apically appearing clubbed, ~1.4X as long as basiphallus.

**Female** (Fig. 36) length: 13–15mm, overall morphology as in male differing in the following traits: **Head**: fronto-orbital plate dull gray, sometimes appearing devoid of tomentum, bearing 3–4 pairs of proclinate orbital setae in addition to single pair of reclinate orbital seta; row of setae directly anterior to post-ocular row absent. **Thorax**: chaetotaxy as in males. **Abdomen**: as in the males differing only in terminalia.

#### Diagnosis

*Belvosiaduvalierbricenoi* **sp. n.** can be distinguished from all other *Belvosia* by the following combination of traits: fronto-orbital plate pale silver gray, genal setulae dark reddish colored, devoid of setulae anterior to postocular row, post sutural scutum mostly silver, both calypters dark, black basicosta, and apex of T5 black tomentose.

#### Etymology

*Belvosiaduvalierbricenoi* **sp. n**, is named in honor of Sr. Duvalie Briceño in recognition of his decades of being part of the Parataxonomist Program of Area de Conservación Guanacaste (http://www.acguanacaste.ac.cr) in northwestern Costa Rica (Janzen and Hallwachs 2011). Interim species-specific name included in previously circulating databases and publications, *Belvosia* Woodley04D.

#### Distribution

Costa Rica, ACG (Provinces of Alajuela and Guanacaste), 90–710 m elevation.

#### Ecology

*Belvosiaduvalierbricenoi* **sp. n.** has been reared 99 times from nine species of Lepidoptera in the family Sphingidae, *Aelloposfadus* (Cramer, 1776) (N=1), *Enyolugubris* (Linnaeus, 1771) (N=1), *Enyoocypete* (Linnaeus, 1758) (N=8), *Eumorphalabruscae* (Linnaeus, 1758) (N=2), *E.satellitia* (Linnaeus, 1771) (N=62), *E.triangulum* (Rothschild & Jordan, 1903) (N=1), *Pachygonidiadrucei* (Rothschild & Jordan, 1903) (N=17), *P.subhamata* (Walker, 1856) (N=2), *Unzelajapix* (Cramer, 1776) (N=5) in rain forest, dry forest, and dry-rain lowland intergrade.

### Belvosia eldaarayae

Fleming & Woodley sp. nov.

283646BB-D2B7-5C97-870B-973E191E703C

67F59506-54F7-496A-A947-256F0AC58F69

#### Materials

- **Type status:** Holotype. **Occurrence:** occurrenceDetails: http://janzen.sas.upenn.edu; catalogNumber: DHJPAR0001158; recordedBy: D.H. Janzen, W. Hallwachs & gusaneros; individualID: DHJPAR0001158; individualCount: 1; sex: Male; lifeStage: adult; preparations: pinned; otherCatalogNumbers: HCIC038-05, 96-SRNP-7260.13, BOLD:AAC0626; occurrenceID: 39907FCC-A4CC-5643-919A-741F81F6F7CD; **Taxon:** scientificName: Belvosiaeldaarayae; phylum: Arthropoda; class: Insecta; order: Diptera; family: Tachinidae; genus: Belvosia; specificEpithet: eldaarayae; scientificNameAuthorship: Fleming & Woodley, 2023; **Location:** continent: Central America; country: Costa Rica; countryCode: CR; stateProvince: Guanacaste; county: Sector Santa Rosa; locality: Area de Conservacion Guanacaste; verbatimLocality: Bosque San Emilio; verbatimElevation: 300; verbatimLatitude: 10.8439; verbatimLongitude: -85.6138; verbatimCoordinateSystem: Decimal; decimalLatitude: 10.8439; decimalLongitude: -85.6138; **Identification:** identifiedBy: AJ Fleming; dateIdentified: 2022; **Event:** samplingProtocol: Reared from the larvae of the Saturniidae, Rothschildiaerycina; verbatimEventDate: 20-Apr-1997; **Record Level:** language: en; institutionCode: CNC; collectionCode: Insects; basisOfRecord: Pinned Specimen
- **Type status:** Paratype. **Occurrence:** occurrenceDetails: http://janzen.sas.upenn.edu; catalogNumber: DHJPAR0001155; recordedBy: D.H. Janzen, W. Hallwachs; individualID: DHJPAR0001155; individualCount: 1; sex: Female; lifeStage: adult; preparations: pinned; otherCatalogNumbers: HCIC014-05, 01-SRNP-12402; occurrenceID: F2E567EE-9EDD-5EFA-8EBB-77A44CACF9AC; **Taxon:** scientificName: Belvosiaeldaarayae; phylum: Arthropoda; class: Insecta; order: Diptera; family: Tachinidae; genus: Belvosia; specificEpithet: eldaarayae; scientificNameAuthorship: Fleming & Woodley, 2023; **Location:** continent: Central America; country: Costa Rica; countryCode: CR; stateProvince: Guanacaste; county: Sector Santa Rosa; locality: Area de Conservacion Guanacaste; verbatimLocality: Cortafuegos Naranjo; verbatimElevation: 285; verbatimLatitude: 10.8352; verbatimLongitude: -85.6248; verbatimCoordinateSystem: Decimal; decimalLatitude: 10.8352; decimalLongitude: -85.6248; **Identification:** identifiedBy: AJ Fleming; dateIdentified: 2022; **Event:** samplingProtocol: Reared from the larvae of the Saturniidae, Rothschildia lebeau; verbatimEventDate: 19-May-2001; **Record Level:** language: en; institutionCode: CNC; collectionCode: Insects; basisOfRecord: Pinned Specimen
- **Type status:** Paratype. **Occurrence:** occurrenceDetails: http://janzen.sas.upenn.edu; catalogNumber: 82-SRNP-313; recordedBy: D.H. Janzen, W. Hallwachs & Lucia Rios; individualID: 82-SRNP-313; individualCount: 1; sex: Male; lifeStage: adult; preparations: pinned; otherCatalogNumbers: 82-SRNP-313; occurrenceID: 56118CC4-18BB-565E-B112-11D7FB3B530E; **Taxon:** scientificName: Belvosiaeldaarayae; phylum: Arthropoda; class: Insecta; order: Diptera; family: Tachinidae; genus: Belvosia; specificEpithet: eldaarayae; scientificNameAuthorship: Fleming & Woodley, 2023; **Location:** continent: Central America; country: Costa Rica; countryCode: CR; stateProvince: Guanacaste; county: Sector Santa Rosa; locality: Area de Conservacion Guanacaste; verbatimLocality: Area Administrativa; verbatimElevation: 295; verbatimLatitude: 10.837640; verbatimLongitude: -85.618710; verbatimCoordinateSystem: Decimal; decimalLatitude: 10.83764; decimalLongitude: -85.61871; **Identification:** identifiedBy: AJ Fleming; dateIdentified: 2022; **Event:** samplingProtocol: Reared from the larvae of the Saturniidae, Rothschildia lebeau; verbatimEventDate: 25-Jun-1982; **Record Level:** language: en; institutionCode: CNC; collectionCode: Insects; basisOfRecord: Pinned Specimen

#### Description

**Male** (Fig. 37), length: 12–14mm. **Head**: head slightly wider than thorax; vertex 1/3 head width; gena 1/4 of head height, 1/3 of eye height. Fronto-orbital plate brilliant silver with three distinct rows of frontal setae, sparsely populated with short black hair-like setulae intermingled with setae, with a few dark colored setulae extending below lowest frontal seta; ocellar setae absent at most several hair-like setulae present on ocellar triangle; orbital setae absent. Parafacial light yellow in ground color, densely covered in silver tomentum making the entire surface reflective and brilliant appearance; almost bare along parafacial outside facial ridge, with only a small number of setulae extending just below lowest frontal setae; facial ridge setose along 3/4 of its length, with few black hair-like setulae emerging along outer edge of row; gena covered in black setulae. Antenna, pedicel black, concolorous with postpedicel; postpedicel black, 5X as long as pedicel; arista bare gradually tapering to a point at tip. Palps, orange apically darkening to a brown color basally and densely covered in short black setulae; only slightly clubbed, tapering to a slight point apically, devoid of setulae apically. Profile distinctly pointed at antennal insertion point giving the head a conical appearance when viewed laterally. **Thorax**: black ground color, with light gray tomentum throughout presuturally, postsuturally transitioning to brown-bronze when viewed from a caudal angle; scutellum appearing dark brown-black to the naked eye, under microscope glabrous adjacent to scutum, abruptly transitioning to dense bronze tomentum which becomes apparent when view on an oblique caudal angle; scutum with four dorsal vittae, one outer pair, one inner pair broken at suture; lateral surface of thorax densely covered in long hair-like setulae, these setulae all black, anepimeron covered bearing the same brown-bronze tomentum present on the scutum, remainder of pleural surfaces gray tomentose; chaetotaxy: 5–6 strong setae on postpronotum arranged in a line, acrostichal setae 3:3–4; dorsocentral setae 3:4; intra-alar setae 3:3; supra-alar setae 2:3; 4–5 katepisternal setae; scutellum, with 5–6 pairs of long flat marginal setae of subequal length; apical often absent but when present these are short, weak and erect, inserted above the plane of the marginal setae; 1 complete row of scutellar discal setae just posterior to marginal setae. **Wing**: strongly infuscate, slightly darkened but not orange at wing base, basicosta black with slight accent of orange along caudal edge; both upper and lower calypters also infuscate concolorous with remainder of wing; wing vein R_4+5_ setose, bearing only 2–3 setulae at base; halteres orange stalk with dark black/brown capitulum. **Legs**: black overall, covered in shimmering bronze tomentum, coxa on midleg and hindleg covered in black setulae; tarsal claws yellow-orange with black tips, with orange pulvilli subequal to length of tarsal claws; anterodorsal row of setae on hind tibia irregularly sized not fringelike. **Abdomen**: large, flattened globose, with black ground color, brown lateroventrally on ST1+2–T4; tomentum absent from T3, gold tomentum along anterior 10-15% of surface of T4 becoming more apparent under different angles, bisected medially by an area devoid of tomentum, densely gold tomentose throughout T5 not reaching to hind margin of tergite, black along caudal 10% of tergite, where it is devoid of gold; ventral surfaces of T3–T5 densely hirsute, reminiscent of sex-patches present in other Goniini, but lacking any definitive shape or form; middorsal depression on ST1+2 reaching to hind margin of tergite; one pair of median marginal setae present on ST1+2 and T3, and complete rows of setae on T4 and T5; T5 devoid of any setulae in the area of gold tomentosity.

**Male terminalia** (Fig. 38): sternite 5 with a deeply excavated median cleft along posterior edge, roughly V-shaped, margins covered in dense tomentum; posterior lobes rounded apically, densely covered in multiple long, fine hair-like setulae. Anterior plate of sternite 5, subequal to length of posterior lobes; unsclerotized "window" on anterior plate of sternite 5 rectangular, translucent. Cerci in posterior view, short, stubby triangular, marginally longer than wide, slightly longer than surstyli, pointed at apex, medially fused, separating only along anterior 2/5 of their length. Cerci in lateral view, over all slightly anteriorly curved, more acutely at apex; densely setose along almost 2/3 of its length, only bare at apex. Surstylus in lateral view, rounded along posterior edge and flat along anterior edge making the process look like a cleaver-type blade; surstylus appearing to be fused with epandrium; when viewed posteriorly surstyli straight. Pregonite broad and well developed, apically squared off, blunt, devoid of setulae. Postgonite, slightly narrowed, 1/3 as wide as pregonite, curved at apex, longer than pregonite, scythelike. Distiphallus broadly cone-shaped with a pronounced flare, with a slender median longitudinal sclerotized reinforcement on its posterior surface not reaching apex and a broad, anterolateral, sclerotized acrophallus, thickened apically appearing clubbed, ~1.6X as long as basiphallus.

**Female** (Fig. 39) length: 10–14mm, overall morphology as in male differing in the following traits: **Head**: fronto-orbital plate dull gray, sometimes appearing devoid of tomentum along vertex, bearing 4–6 pairs of proclinate orbital setae in addition to 1–2 pairs of reclinate orbital seta; profile of head not rounded as in males. **Thorax**: Thoracic chaetotaxy: acrostichal setae 3:4; dorsocentral setae 3:4; intra-alar setae 2:3; supra-alar setae 2:3. **Abdomen**: more globose than males, lacking the flattened character, setulae on abdomen not as dense appearing far less hirsute than male abdomen; differing in terminalia, and the gold tomentosity on T4 extending over 40-50% of tergal surface.

#### Diagnosis

*Belvosiaeldaarayae* **sp. n.** can be distinguished from all other *Belvosia* by the following combination of traits: fronto-orbital plate pale silver gray, gena covered in black setulae, post sutural scutum mostly brassy-brown tomentose, both calypters dark, black basicosta, and apex of T5 black tomentose.

#### Etymology

*Belvosiaeldaarayae* **sp. n**, is named in honor of Sra. Elda Araya in recognition of her decades of being part of the Parataxonomist Program of Area de Conservación Guanacaste (http://www.acguanacaste.ac.cr) in northwestern Costa Rica (Janzen and Hallwachs 2011). Interim species-specific name included in previously circulating databases and publications, *Belvosia* Woodley05.

#### Distribution

Costa Rica, ACG (Provinces of Alajuela and Guanacaste), 96–690 m elevation.

#### Ecology

*Belvosiaeldaarayae* **sp. n.** has been reared 64 times from three species of Lepidoptera in the family Saturniidae, *Rothschildiaerycina* (Shaw, 1796) (N=5), *R.lebeau* (Guerin-Meneville, 1868) (N=58), *R.triloba* Rothschild, 1907 (N=1) in rain forest, dry forest, and dry-rain lowland intergrade.

### Belvosia eliethcantillanoae

Fleming & Woodley, 2019 sp. nov.

6DA90E71-641F-51BA-9533-2FF5D6109C78

86C1D924-9CAB-46BE-91F5-910731B51436

#### Materials

- **Type status:** Holotype. **Occurrence:** occurrenceDetails: http://janzen.sas.upenn.edu; catalogNumber: DHJPAR0001161; recordedBy: D.H. Janzen, W. Hallwachs & Juan Acosta; individualID: DHJPAR0001161; individualCount: 1; sex: Male; lifeStage: adult; preparations: pinned; otherCatalogNumbers: HCIC062-05,95-SRNP-4739, BOLD:ABZ6041; occurrenceID: 80083D31-E6BF-547E-A9E2-27EA4DBB2934; **Taxon:** scientificName: Belvosiaeliethcantillanoae; phylum: Arthropoda; class: Insecta; order: Diptera; family: Tachinidae; genus: Belvosia; specificEpithet: eliethcantillanoae; scientificNameAuthorship: Fleming & Woodley, 2023; **Location:** continent: Central America; country: Costa Rica; countryCode: CR; stateProvince: Guanacaste; county: La Esperanza; locality: Area de Conservacion Guanacaste; verbatimLocality: Acosta; verbatimElevation: 500; verbatimCoordinateSystem: Decimal; **Identification:** identifiedBy: AJ Fleming; dateIdentified: 2022; **Event:** samplingProtocol: Reared from the larvae of the Sphingidae, Manduca Janzen01; verbatimEventDate: 05-Aug-1995; **Record Level:** language: en; institutionCode: CNC; collectionCode: Insects; basisOfRecord: Pinned Specimen
- **Type status:** Paratype. **Occurrence:** occurrenceDetails: http://janzen.sas.upenn.edu; catalogNumber: 84-SRNP-408A; recordedBy: D.H. Janzen, W. Hallwachs & gusaneros; individualID: 84-SRNP-408A; individualCount: 1; sex: Male; lifeStage: adult; preparations: pinned; otherCatalogNumbers: 84-SRNP-408A; occurrenceID: ACCE6874-5DD6-520C-9B96-A809A971F4C0; **Taxon:** scientificName: Belvosiaeliethcantillanoae; phylum: Arthropoda; class: Insecta; order: Diptera; family: Tachinidae; genus: Belvosia; specificEpithet: eliethcantillanoae; scientificNameAuthorship: Fleming & Woodley, 2023; **Location:** continent: Central America; country: Costa Rica; countryCode: CR; stateProvince: Guanacaste; county: Sector Santa Rosa; locality: Area de Conservacion Guanacaste; verbatimLocality: Bosque Humedo; verbatimElevation: 290; verbatimLatitude: 10.851450; verbatimLongitude: -85.608010; verbatimCoordinateSystem: Decimal; decimalLatitude: 10.85145; decimalLongitude: -85.60801; **Identification:** identifiedBy: AJ Fleming; dateIdentified: 2022; **Event:** samplingProtocol: Reared from the larvae of the Sphingidae, Manducadilucida; verbatimEventDate: Aug-26-1984; **Record Level:** language: en; institutionCode: CNC; collectionCode: Insects; basisOfRecord: Pinned Specimen
- **Type status:** Paratype. **Occurrence:** occurrenceDetails: http://janzen.sas.upenn.edu; catalogNumber: DHJPAR0001874; recordedBy: D.H. Janzen, W. Hallwachs & Daniel H. Janzen; individualID: DHJPAR0001874; individualCount: 1; sex: Female; lifeStage: adult; preparations: pinned; otherCatalogNumbers: HCIC390-05,84-SRNP-1642, BOLD:ABZ6041; occurrenceID: 7FB189D1-2547-501A-BE61-FC615B199105; **Taxon:** scientificName: Belvosiaeliethcantillanoae; phylum: Arthropoda; class: Insecta; order: Diptera; family: Tachinidae; genus: Belvosia; specificEpithet: eliethcantillanoae; scientificNameAuthorship: Fleming & Woodley, 2023; **Location:** continent: Central America; country: Costa Rica; countryCode: CR; stateProvince: Guanacaste; county: Sector Santa Rosa; locality: Area de Conservacion Guanacaste; verbatimLocality: Bosque Humedo; verbatimElevation: 290; verbatimLatitude: 10.8514; verbatimLongitude: -85.608; verbatimCoordinateSystem: Decimal; decimalLatitude: 10.8514; decimalLongitude: -85.608; **Identification:** identifiedBy: AJ Fleming; dateIdentified: 2022; **Event:** samplingProtocol: Reared from the larvae of the Sphingidae, Amphonyxduponchel; verbatimEventDate: 08-Aug-1984; **Record Level:** language: en; institutionCode: CNC; collectionCode: Insects; basisOfRecord: Pinned Specimen

#### Description

**Male** (Fig. 40), length: 14–17mm. **Head**: head slightly wider than thorax; vertex 1/3 head width; gena 1/4 of head height, 1/3 of eye height. Fronto-orbital plate dull gray to brilliant silver with three distinct rows of frontal setae, with short black hair-like setulae intermingled with setae, with a few dark colored setulae extending below lowest frontal seta; ocellar setae absent at most several hair-like setulae present on ocellar triangle; one pair of reclinate orbital setae. Parafacial light yellow in ground color, densely covered in silver tomentum making the entire surface reflective and brilliant silver with a light gold sheen; almost bare along parafacial outside facial ridge, with only a small number of setulae extending just below lowest frontal setae; facial ridge setose along 3/4 of its length, with shorth black hair-like setulae emerging along outer edge of row; gena covered in black setulae. Antenna, pedicel black, concolorous with postpedicel; postpedicel black, 4–5X as long as pedicel; arista bare gradually tapering to a point at tip. Vibrissa arising above oral margin by length of 1 pedicel. Palps, yellow orange throughout and densely covered in short black setulae; only slightly clubbed, tapering to a slight point apically, devoid of setulae apically. **Thorax**: darkened orange ground color, with light gray tomentum throughout pre- and post- suturally, this tomentum tapering off adjacent to scutellum, sometimes bronze brown tomentosity visible confined to postalar callosity; scutellum appearing light yellow-orange to the naked eye, under microscope glabrous adjacent to scutum, abruptly transitioning to dense bronze tomentum which becomes apparent when view on an oblique caudal angle; scutum with four dorsal vittae, one outer pair, one inner pair broken at suture; lateral surface of thorax densely covered in long hair-like setulae, these setulae all black, anepimeron covered bearing the same brown-bronze tomentum present on the scutellum, remainder of pleural surfaces gray tomentose; chaetotaxy: 3–4 strong setae on postpronotum arranged in a line, acrostichal setae 3:3–4; dorsocentral setae 3:4; intra-alar setae 2:3; supra-alar setae 2:3; 4–5 katepisternal setae; scutellum, with 4–5 pairs of long flat marginal setae of subequal length; apical often absent but when present these are short, weak and erect, inserted above the plane of the marginal setae; 2 pairs of median discal scutellar setae. **Wing**: strongly infuscate, slightly darkened but not orange at wing base, basicosta black with slight accent of orange along caudal edge; both upper and lower calypters also infuscate concolorous with remainder of wing; wing vein R_4+5_ setose, bearing only 2–3 setulae at base; halteres orange stalk with dark black/brown capitulum. **Legs**: black overall, covered in shimmering bronze tomentum, coxa on midleg and hindleg covered in black setulae; tarsal claws yellow-orange with black tips, with orange pulvilli subequal to length of tarsal claws; anterodorsal row of setae on hind tibia irregularly sized not fringelike. **Abdomen**: large, flattened globose, with black ground color, brown lateroventrally on ST1+2–T4; tomentum absent from ST1+2–T3, gold tomentum along anterior >60% of surface of T4 apparent under all lighting angles, not bisected medially by an area devoid of tomentum, densely gold tomentose throughout T5 not reaching to hind margin of tergite, black along caudal 10% of tergite, where it is devoid of gold; ventral surfaces of T3–T5 lightly hirsute; middorsal depression on ST1+2 reaching to hind margin of tergite; one pair of median marginal setae present on ST1+2 and T3, and complete rows of setae on T4 and T5; T5 devoid of any setulae in the area of gold tomentosity.

**Male terminalia** (Fig. 41): sternite 5 with a deeply excavated median cleft along posterior edge, smoothly Y-shaped, margins covered in dense tomentum; posterior lobes rounded apically, with multiple strong setae surrounded by many shorter weaker setulae. Anterior plate of sternite 5, 1/2 length of posterior lobes; unsclerotized "window" on anterior plate of sternite 5 translucent directly basal to posterior lobes, flattened rectangular with a slight upward arc at extremities. Cerci in posterior view like an isosceles triangle, narrow and parallel sided, slightly longer than surstyli; slightly rounded at apex, medially to fused along most of its length only separate on anterior 1/4. Cerci in lateral view, obclavate with a moderate anterior curve at apex; cerci densely setose along basal 4/5ths. Surstylus in lateral view, wide almost equilateral along its length broadly downcurved, appearing digitiform; surstylus appearing to be fused with epandrium; when viewed posteriorly surstyli slightly convergent, not angled inwards so as to not be clearly visible under cerci when viewed from a caudal angle. Pregonite usually broad, well-developed, apically squared off appearing subrectangular, with 2–3 strong setulae along inner margin. Postgonite, slightly narrowed, 1/3 as wide as pregonite, rounded clublike at apex. Distiphallus broadly cone-shaped with a pronounced flare, with a slender median longitudinal sclerotized reinforcement on its posterior surface not reaching apex and a broad, anterolateral, sclerotized acrophallus, thickened apically appearing clubbed, 1.4X longer than basiphallus.

**Female** (Fig. 42) length: 15–17mm, overall morphology as in male differing in the following traits: **Head**: fronto-orbital plate dull gray, sometimes appearing devoid of tomentum along vertex, bearing 4–6 pairs of proclinate orbital setae in addition to 1–2 pairs of reclinate orbital seta; profile of head not rounded as in males; gena 1/3 of head height, and 1/2 of eye height. **Thorax**: Thoracic chaetotaxy: acrostichal setae 3:4; dorsocentral setae 3:4; intra-alar setae 2:3; supra-alar setae 2:3. **Abdomen**: more globose than males, lacking the flattened character, setulae on abdomen not as dense appearing far less hirsute than male abdomen; differing in terminalia, and T3 bearing goldish tomentum on ventral surface.

#### Diagnosis

*Belvosiaeliethcantillanoae* **sp. n.** can be distinguished from all other *Belvosia* by the following combination of traits: genal setulae dark, basicosta black, female with palps rounded apically, postpedicel more than 2X longer than pedicel, both calypters dark infuscate, T4 over 60% gold tomentose, cercus narrow and triangular, surstylus narrow and digitiform.

#### Etymology

*Belvosiaeliethcantillanoae* **sp. n**, is named in honor of Sra. Elieth Cantillano in recognition of her decades of being part of the Parataxonomist Program of Area de Conservación Guanacaste (http://www.acguanacaste.ac.cr) in northwestern Costa Rica (Janzen and Hallwachs 2011). Interim species-specific name included in previously circulating databases and publications, *Belvosia* Woodley06.

#### Distribution

Costa Rica, ACG, Guanacaste Province, 105–550 m elevation.

#### Ecology

*Belvosiaeliethcantillanoae* **sp. n.** has been reared 97 times from 14 species of Lepidoptera in two families Saturniidae: *Periphobaarcaei* (Druce, 1886) (N=1) and Sphingidae, *Aelloposfadus* (N=1), *Agriuscingulata* (Fabricius, 1775) (N=4), *Amphonyxduponchel* Poey, 1832 (N=2), *Cocytiusanteus* (Drury, 1773) (N=1), *Lintneriamerops* (Boisduval, 1870) (N=3), *Manducadilucida* (Edwards, 1887) (N=67), *M.florestan* (Stoll, 1772) (N=2), *M.* Janzen01 (N=1), *M.muscosa* (Rothschild & Jordan, 1903) (N=6), *M.occulta* (Rothschild & Jordan, 1903) (N=1), *M.rustica* (Fabricius, 1775) (N=4), *M.sexta*DHJ03 (N=1), *Neococytiuscluentius* (Cramer, 1776) (N=2) in rain forest, dry forest, and dry-rain lowland intergrade.

### Belvosia freddyquesadai

Fleming & Woodley sp. nov.

04BF936A-E9C8-5697-9E76-9FB4F3A98C19

48B525E3-D1BE-4CCB-9947-F2098036BF3D

#### Materials

- **Type status:** Holotype. **Occurrence:** occurrenceDetails: http://janzen.sas.upenn.edu; catalogNumber: DHJPAR0054965; recordedBy: D.H. Janzen, W. Hallwachs & Manuel Rios; individualID: DHJPAR0054965; individualCount: 1; sex: Male; lifeStage: adult; preparations: pinned; otherCatalogNumbers: ASHYH1512-14, 14-SRNP-30020, BOLD:AAA8475; occurrenceID: E643BC2A-6A3F-593E-9E1F-D3B0614B4EFD; **Taxon:** scientificName: Belvosiafreddyquesadai; phylum: Arthropoda; class: Insecta; order: Diptera; family: Tachinidae; genus: Belvosia; specificEpithet: freddyquesadai; scientificNameAuthorship: Fleming & Woodley, 2023; **Location:** continent: Central America; country: Costa Rica; countryCode: CR; stateProvince: Guanacaste; county: Sector Pitilla; locality: Area de Conservacion Guanacaste; verbatimLocality: Estacion Pitilla; verbatimElevation: 675; verbatimLatitude: 10.9893; verbatimLongitude: -85.4258; verbatimCoordinateSystem: Decimal; decimalLatitude: 10.9893; decimalLongitude: -85.4258; **Identification:** identifiedBy: AJ Fleming; dateIdentified: 2022; **Event:** samplingProtocol: Reared from the larvae of the Sphingidae, Xylophaneschiron; verbatimEventDate: 25-Feb-2014; **Record Level:** language: en; institutionCode: CNC; collectionCode: Insects; basisOfRecord: Pinned Specimen
- **Type status:** Paratype. **Occurrence:** occurrenceDetails: http://janzen.sas.upenn.edu; catalogNumber: DHJPAR0023250; recordedBy: D.H. Janzen, W. Hallwachs & Calixto Moraga; individualID: DHJPAR0023250; individualCount: 1; sex: Female; lifeStage: adult; preparations: pinned; otherCatalogNumbers: ASTAW411-08, 07-SRNP-65788, BOLD:AAA8475; occurrenceID: 25A053DC-7D91-5730-8456-5B5C9D934FEC; **Taxon:** scientificName: Belvosiafreddyquesadai; phylum: Arthropoda; class: Insecta; order: Diptera; family: Tachinidae; genus: Belvosia; specificEpithet: freddyquesadai; scientificNameAuthorship: Fleming & Woodley, 2023; **Location:** continent: Central America; country: Costa Rica; countryCode: CR; stateProvince: Alajuela; county: Brasilia; locality: Area de Conservacion Guanacaste; verbatimLocality: Camino Ensayo; verbatimElevation: 500; verbatimLatitude: 10.9515; verbatimLongitude: -85.3739; verbatimCoordinateSystem: Decimal; decimalLatitude: 10.9515; decimalLongitude: -85.3739; **Identification:** identifiedBy: AJ Fleming; dateIdentified: 2022; **Event:** samplingProtocol: Reared from the larvae of the Sphingidae, Xylophaneschiron; verbatimEventDate: 19-Dec-2007; **Record Level:** language: en; institutionCode: CNC; collectionCode: Insects; basisOfRecord: Pinned Specimen
- **Type status:** Paratype. **Occurrence:** occurrenceDetails: http://janzen.sas.upenn.edu; catalogNumber: DHJPAR0059786; recordedBy: D.H. Janzen, W. Hallwachs & Freddy Quesada; individualID: DHJPAR0059786; individualCount: 1; sex: Male; lifeStage: adult; preparations: pinned; otherCatalogNumbers: ACGBA6207-16, 16-SRNP-31290, BOLD:AAA8475; occurrenceID: C72A52C8-63A9-5E08-BD2F-2668A275DD9A; **Taxon:** scientificName: Belvosiafreddyquesadai; phylum: Arthropoda; class: Insecta; order: Diptera; family: Tachinidae; genus: Belvosia; specificEpithet: freddyquesadai; scientificNameAuthorship: Fleming & Woodley, 2023; **Location:** continent: Central America; country: Costa Rica; countryCode: CR; stateProvince: Guanacaste; county: Sector Pitilla; locality: Area de Conservacion Guanacaste; verbatimLocality: Sendero Laguna; verbatimElevation: 680; verbatimLatitude: 10.9888; verbatimLongitude: -85.4234; verbatimCoordinateSystem: Decimal; decimalLatitude: 10.9888; decimalLongitude: -85.4234; **Identification:** identifiedBy: AJ Fleming; dateIdentified: 2022; **Event:** samplingProtocol: Reared from the larvae of the Sphingidae, Xylophaneschiron; verbatimEventDate: 14-Sep-2016; **Record Level:** language: en; institutionCode: CNC; collectionCode: Insects; basisOfRecord: Pinned Specimen

#### Description

**Male** (Fig. 43), length: 14–17mm. **Head**: head slightly wider than thorax; vertex 1/4 head width; gena 1/3 of head height, 2/5 of eye height. Fronto-orbital plate light black in ground color, lightly covered with gray tomentum giving majority of the plate a glabrous dark gray sheen transitioning to silver; ocellar setae absent at most several hair-like setulae present on ocellar triangle; inner row of 5-10 post-ocular setae; reclinate orbital seta absent; two rows of frontal setae, black setulae intermingled with setae, several black setulae present below lowest frontal setae. Parafacial dark yellow in ground color, densely covered in silver tomentum making the entire surface reflective brilliant silver appearance; bare overall, except for a small number of setulae extending just below lowest frontal setae; facial ridge setose along 1/3–1/2 of its length, with a few sparse hair-like setulae emerging along outer edge of row; gena covered in black setulae. Antenna, pedicel black, concolorous with postpedicel; postpedicel, 1.5X as long as pedicel; arista bare, with a regular taper along most of its length only thickened on basal 1/5 almost to tip. Palps, yellow-orange throughout and densely covered in short black setulae; slightly clubbed, but gradually tapering to a slight point apically. **Thorax**: black ground color, with light gray tomentum throughout, when viewed dorsally tomentum appears thinner postsuturally, some bronze tomentum on the postalar callosity; scutellum appearing reddish-black in ground color, under microscope bronze tomentum becomes apparent when view on an oblique caudal angle; scutum with four dorsal vittae, becoming more evident under certain angles of light, these broken at suture; lateral surface of thorax densely covered in long black hair-like setulae; chaetotaxy: 3–4 strong setae on postpronotum arranged in a line, acrostichal setae 3:4–6 often with 2 extra setae appearing just adjacent to acrostichal setae; dorsocentral setae 3:4; intra-alar setae 3:3; supra-alar setae 2:3; 4–6 katepisternal setae; scutellum, with 5–6 pairs of long flat marginal setae of subequal length; apical setae absent; complete row of scutellar discal setae just posterior to marginal setae, approximately 1/3 as long as scutellar marginals. **Wing**: strongly infuscate, slightly orange at wing base, black basicosta, with some orange along posterior margin; both upper and lower calypters also infuscate concolorous with remainder of wing; wing vein R_4+5_ setose, bearing only 2–3 setulae at base; halteres orange stalk with dark black/brown capitulum. **Legs**: black overall, coxa on midleg and hindleg with a few reddish-yellow setulae; tarsal claws yellow with black tips, with yellow pulvilli 2/3 length of tarsal claws; Anterodorsal row of setae on hind tibia fringelike, formed by a very regular row of uniformly sized setae separated from each other by less than the width of their socket. **Abdomen**: globose, with dark burgundy-black ground color; abdominal tomentosity on T3-T4 bronze confined to the anterior margin of the tergite, at most anterior 10% of surface, T5 densely gold tomentose on 95% of surface absent along posterior 5%, which appears as glabrous black; middorsal depression on ST1+2 reaching to hind margin of tergite, median marginal setae present on ST1+2 wide set, stout but short, approximately 1/2 as long as median marginals on T3, T3 also with 1 pair of median marginal setae, and complete rows of marginal setae on T4 and T5; ventral surfaces of T3–T4 with clearly defined sex-patches extending from underside of tergite to lateral surface.

**Male terminalia** (Fig. 44): sternite 5 with a deeply excavated median cleft along posterior edge, roughly V-shaped, margins covered in dense tomentum; posterior lobes rounded apically, with multiple fine setae surrounded by many shorter weaker setulae. Anterior plate of sternite 5, 1/2 length of posterior lobes; unsclerotized "window" on anterior plate of sternite 5 translucent directly basal to posterior lobes, flattened rectangular with a slight upward arc at extremities. Cerci in posterior view like an isosceles triangle, 2x as long as wide, narrow and parallel sided, slightly longer than surstyli; pointed at apex, medially to fused along most of its length only separate on anterior 1/2. Cerci in lateral view, obclavate with a moderate anterior curve at apex; cerci densely setose along basal 4/5ths. Surstylus in lateral view, wide almost equilateral along its length broadly downcurved, appearing digitiform; surstylus appearing to be fused with epandrium; when viewed posteriorly surstyli straight. Pregonite broad, well-developed, apically rounded, with 2–3 strong setulae along inner margin. Postgonite, slightly narrowed, 1/3 as wide as pregonite, short and sharp at apex. Distiphallus broadly cone-shaped with a pronounced flare, with a slender median longitudinal sclerotized reinforcement on its posterior surface not reaching apex and a broad, anterolateral, sclerotized acrophallus, thickened apically appearing clubbed, 1.5X longer than basiphallus.

**Female** (Fig. 45) length: 14–17mm, overall morphology as in male differing in the following traits: **Head**: fronto-orbital plate dull gray, sometimes appearing devoid of tomentum along vertex, bearing 4–6 pairs of proclinate orbital setae in addition to 1–2 pairs of reclinate orbital seta; profile of head not rounded as in males. **Thorax**: Thoracic chaetotaxy: acrostichal setae 3–4:4; dorsocentral setae 3–4:4; intra-alar setae 2–3:3; supra-alar setae 2:3. **Abdomen**: more globose than males, lacking the flattened character, setulae on abdomen not as dense appearing far less hirsute than male abdomen; differing in terminalia, and T3 bearing goldish tomentum on ventral surface.

#### Diagnosis

*Belvosiafreddyquesadai* **sp. n.** can be distinguished from all other *Belvosia* by the following combination of traits: gena covered in black setulae, inner row of 5-10 post-ocular setae, black basicosta, both calypters infuscate, anterodorsal setae on hind tibia comblike and regular, median marginal setae on ST1+2 reduced to absent, and T5 black apically.

#### Etymology

*Belvosiafreddyquesadai* **sp. n**, is named in honor of Sr. Freddy Quesada in recognition of his decades of being part of the Parataxonomist Program of Area de Conservación Guanacaste (http://www.acguanacaste.ac.cr) in northwestern Costa Rica (Janzen and Hallwachs 2011). Interim species-specific name included in previously circulating databases and publications, *Belvosia* Woodley07A.

#### Distribution

Costa Rica, ACG (Provinces of Alajuela and Guanacaste), 95–1020m elevation

#### Ecology

*Belvosiafreddyquesadai* **sp. n.** has been reared 30 times from eight species of Lepidoptera in the family Sphingidae, *Erynnyisello* (Linnaeus, 1758) (N=1), *Xylophanesadalia* (Druce, 1881) (N=3), *X.ceratomioides* (Grote & Robinson, 1867) (N=1), *X.chiron* (Drury, 1773) (N=19), *X.germen* (Schaus, 1890) (N=1), *X.hannemanni* (Closs, 1917) (N=1), *X.maculator* (Boisduval, 1875) (N=1), *X.zurcheri* (Druce, 1894) (N=4) in cloud forest, dry foresrt, rain forest, and dry-rain lowaland intergrade.

### Belvosia gloriasihezarae

Fleming & Woodley sp. nov.

F2BEC6D0-4B68-5A73-98DB-7E0518C3AF73

D5977388-3553-4514-BF74-D69DB64335C4

#### Materials

- **Type status:** Holotype. **Occurrence:** occurrenceDetails: http://janzen.sas.upenn.edu; catalogNumber: DHJPAR0001985; recordedBy: D.H. Janzen, W. Hallwachs & gusaneros; individualID: DHJPAR0001985; individualCount: 1; sex: Male; lifeStage: adult; preparations: pinned; otherCatalogNumbers: HCIC501-05, 94-SRNP-3273, BOLD:AAA8475; occurrenceID: B785C126-B5C5-5B43-AD9A-AC83AFB08EAB; **Taxon:** scientificName: Belvosiagloriasihezarae; phylum: Arthropoda; class: Insecta; order: Diptera; family: Tachinidae; genus: Belvosia; specificEpithet: gloriasihezarae; scientificNameAuthorship: Fleming & Woodley, 2023; **Location:** continent: Central America; country: Costa Rica; countryCode: CR; stateProvince: Guanacaste; county: Sector Santa Rosa; locality: Area de Conservacion Guanacaste; verbatimLocality: Tanquetas; verbatimElevation: 295; verbatimLatitude: 10.8708; verbatimLongitude: -85.6053; verbatimCoordinateSystem: Decimal; decimalLatitude: 10.8708; decimalLongitude: -85.6053; **Identification:** identifiedBy: AJ Fleming; dateIdentified: 2022; **Event:** samplingProtocol: Reared from the larvae of the Sphingidae, Aelloposfadus; verbatimEventDate: 02-Jul-1994; **Record Level:** language: en; institutionCode: CNC; collectionCode: Insects; basisOfRecord: Pinned Specimen
- **Type status:** Paratype. **Occurrence:** occurrenceDetails: http://janzen.sas.upenn.edu; catalogNumber: DHJPAR0001864; recordedBy: D.H. Janzen, W. Hallwachs & gusaneros; individualID: DHJPAR0001864; individualCount: 1; sex: Male; lifeStage: adult; preparations: pinned; otherCatalogNumbers: HCIC380-05, 88-SRNP-482, BOLD:AAA8475; occurrenceID: E3F37F5E-7098-57DD-8C68-444763D7DDCC; **Taxon:** scientificName: Belvosiagloriasihezarae; phylum: Arthropoda; class: Insecta; order: Diptera; family: Tachinidae; genus: Belvosia; specificEpithet: gloriasihezarae; scientificNameAuthorship: Fleming & Woodley, 2023; **Location:** continent: Central America; country: Costa Rica; countryCode: CR; stateProvince: Guanacaste; county: Sector Orosi; locality: Area de Conservacion Guanacaste; verbatimLocality: Estacion Maritza; verbatimElevation: 570; verbatimLatitude: 10.9592; verbatimLongitude: -85.4951; verbatimCoordinateSystem: Decimal; decimalLatitude: 10.9592; decimalLongitude: -85.4951; **Identification:** identifiedBy: AJ Fleming; dateIdentified: 2022; **Event:** samplingProtocol: Reared from the larvae of the Sphingidae, Erinnyis ello; verbatimEventDate: 22-Jul-1988; **Record Level:** language: en; institutionCode: CNC; collectionCode: Insects; basisOfRecord: Pinned Specimen
- **Type status:** Paratype. **Occurrence:** occurrenceDetails: http://janzen.sas.upenn.edu; catalogNumber: DHJPAR0052415; recordedBy: D.H. Janzen, W. Hallwachs & Guillermo Pereira; individualID: DHJPAR0052415; individualCount: 1; sex: Female; lifeStage: adult; preparations: pinned; otherCatalogNumbers: ASHYM1769-13, 13-SRNP-15205, BOLD:AAA8475; occurrenceID: BDE05808-AD4F-57B4-897D-F581DB200AE6; **Taxon:** scientificName: Belvosiagloriasihezarae; phylum: Arthropoda; class: Insecta; order: Diptera; family: Tachinidae; genus: Belvosia; specificEpithet: gloriasihezarae; scientificNameAuthorship: Fleming & Woodley, 2023; **Location:** continent: Central America; country: Costa Rica; countryCode: CR; stateProvince: Guanacaste; county: Sector Santa Rosa; locality: Area de Conservacion Guanacaste; verbatimLocality: Sendero Natural; verbatimElevation: 290; verbatimLatitude: 10.8357; verbatimLongitude: -85.6125; verbatimCoordinateSystem: Decimal; decimalLatitude: 10.8357; decimalLongitude: -85.6125; **Identification:** identifiedBy: AJ Fleming; dateIdentified: 2022; **Event:** samplingProtocol: Reared from the larvae of the Sphingidae, Aellopostitan; verbatimEventDate: 12-Jul-2013; **Record Level:** language: en; institutionCode: CNC; collectionCode: Insects; basisOfRecord: Pinned Specimen

#### Description

**Male** (Fig. 46), length: 14–15mm. **Head**: head slightly wider than thorax; vertex 1/3 head width; gena 1/5 of head height, 1/4 of eye height. Fronto-orbital plate light black in ground color, lightly covered with gray tomentum giving majority of the plate a glabrous dark gray sheen transitioning to silver; ocellar setae absent at most several hair-like setulae present on ocellar triangle; inner row of 5-10 post-ocular setae; reclinate orbital seta absent; two rows of frontal setae, black setulae intermingled with setae, several black setulae present below lowest frontal setae. Parafacial dark yellow in ground color, densely covered in silver tomentum making the entire surface reflective brilliant silver appearance; bare overall, except for a small number of setulae extending just below lowest frontal setae; facial ridge setose along 1/3–1/2 of its length, with a few sparse hair-like setulae emerging along outer edge of row; gena covered in black setulae. Antenna, pedicel black, concolorous with postpedicel; postpedicel, 1.5X as long as pedicel; arista bare, with a regular taper along most of its length only thickened on basal 1/5 almost to tip. Palps, yellow-orange throughout and densely covered in short black setulae; slightly clubbed, but gradually tapering to a slight point apically. **Thorax**: black ground color, with light gray tomentum throughout, when viewed dorsally tomentum appears thinner postsuturally, some bronze tomentum on the postalar callosity and posterior edge of scutum; scutellum appearing reddish-yellow in ground color, anterior edge darker than posterior, under microscope bronze tomentum becomes apparent when view on an oblique caudal angle; scutum with four dorsal vittae, becoming more evident under certain angles of light, these broken at suture; lateral surface of thorax densely covered in long black hair-like setulae; chaetotaxy: 3–4 strong setae on postpronotum arranged in a line, acrostichal setae 3:4–6 often with 2 extra setae appearing just adjacent to acrostichal setae; dorsocentral setae 3:4; intra-alar setae 3:3; supra-alar setae 2:3; 4–6 katepisternal setae; small tuft of yellow hair-like setulae at the base of the postalar callosity; scutellum, with 5–6 pairs of long flat marginal setae of subequal length; apical setae absent; complete row of scutellar discal setae just posterior to marginal setae, these setae 1/3 as long as scutelar marginals. **Wing**: strongly infuscate, slightly orange at wing base, black basicosta, with some orange along posterior margin; both upper and lower calypters whitish with a fringe of pale setulae; wing vein R_4+5_ setose, bearing only 2–3 setulae at base; halteres orange stalk with dark black/brown capitulum. **Legs**: black overall, coxa on midleg and hindleg with a few reddish-yellow setulae; tarsal claws yellow with black tips, with yellow pulvilli 2/3 length of tarsal claws; Anterodorsal row of setae on hind tibia fringelike, formed by a very regular row of uniformly sized setae separated from each other by less than the width of their socket. **Abdomen**: globose, with dark burgundy-black ground color; gold tomentum at most on anterior 10% of T3, T4 with gold tomentum over anterior 50% tergite, T5 densely gold tomentose on 95% of surface absent along posterior 50%, which appears as glabrous black; middorsal depression on ST1+2 reaching to hind margin of tergite, median marginal setae absent on ST1+2, T3 also with 1 pair of reduced median marginal setae these approximately 1/2 as long as marginals on T4, and complete rows of marginal setae on T4 and T5; ventral surfaces of T3–T4 with clearly defined sex-patches extending from underside of tergite to lateral surface.

**Male terminalia** (Fig. 47): sternite 5 with a deeply excavated median cleft along posterior edge, smoothly Y-shaped, margins apparently bare; posterior lobes slightly pointed apically, with a wide fringe of strong setulae surrounded by many shorter weaker setulae. Anterior plate of sternite 5, 1/2 length of posterior lobes; unsclerotized "window" on anterior plate of sternite 5 translucent directly basal to posterior lobes, rectangular with upturned extremes giving the entire structure an almost flat "w". Cerci in posterior view like an isosceles triangle, slightly longer than surstyli; slightly rounded at apex, medially to fused along 2/3 its length. Cerci in lateral view, digitiform arced, with a broad anterior curve along its length; cerci densely setose along basal 1/2. Surstylus in lateral view, narrow, tapered, pointed at tips, straight with no apparent curve; surstylus appearing to be fused with epandrium; when viewed posteriorly surstyli appearing slightly divergent at tips, but not broadly not angled outwards. Pregonite broad, well-developed, apically squared off appearing subrectangular, bare. Postgonite, slightly narrowed, 1/3 as wide as pregonite, pointed at tip slightly blade-shaped at apex. Distiphallus broadly cone-shaped with a pronounced flare, with a slender median longitudinal sclerotized reinforcement on its posterior surface not reaching apex and a broad, anterolateral, sclerotized acrophallus, thickened apically appearing clubbed, 1.75X longer than basiphallus.

**Female** (Fig. 48) length: 14–15mm, overall morphology as in male differing in the following traits: **Head**: fronto-orbital plate dull gray, sometimes appearing devoid of tomentum along vertex, bearing 3–4 pairs of proclinate orbital setae in addition to 1–2 pairs of reclinate orbital seta; gena 1/3 head height and 2/5 eye height. **Thorax**: Thoracic chaetotaxy: acrostichal setae 3:4; dorsocentral setae 3:4; intra-alar setae 2:3; supra-alar setae 2:3. **Abdomen**: more globose than males, lacking the flattened character, setulae on abdomen not as dense appearing far less hirsute than male abdomen; differing in terminalia, and T3 bearing goldish tomentum on ventral surface.

#### Diagnosis

*Belvosiagloriasihezarae* **sp. n.** can be distinguished from all other *Belvosia* by the following combination of traits: gena covered in black setulae, black basicosta, white calypters, anterodorsal setae on hind tibia comblike and regular, and T5 black apically.

#### Etymology

*Belvosiagloriasihezarae* **sp. n**, is named in honor of Sra. Gloria Sihezare in recognition of her decades of being part of the Parataxonomist Program of Area de Conservación Guanacaste (http://www.acguanacaste.ac.cr) in northwestern Costa Rica (Janzen and Hallwachs 2011). Interim species-specific name included in previously circulating databases and publications, *Belvosia* Woodley07B.

#### Distribution

Costa Rica, ACG, Alajuela and Guanacaste Provinces, 96–640m elevation

#### Ecology

*Belvosiagloriasihezarae* **sp. n.** has been reared 59 times from seven species of Lepidoptera in the family Sphingidae, *Aelloposceculus* (Cramer, 1777) (N=13), *A.fadus* (Cramer, 1775) (N=15), *A.titan* (Cramer, 1777) (N=9), *Erinnyiscrameri* (Schaus, 1898) (N=5) *E.ello* (Linnaeus, 1758) (N=14), *E.oenotrus* (Cramer, 1780) (N=1), *Eupyrrhoglossumsagra* (Poey, 1832) (N=2), in dry foresrt, rain forest, and dry-rain lowland intergrade.

### Belvosia guillermopereirai

Fleming & Woodley sp. nov.

A822A522-AD99-5EC2-8C30-8EA462858407

4167EC75-C454-4EDB-A663-313B2ED91CD6

#### Materials

- **Type status:** Holotype. **Occurrence:** occurrenceDetails: http://janzen.sas.upenn.edu; catalogNumber: DHJPAR0003933; recordedBy: D.H. Janzen, W. Hallwachs & Xavier Basurto; individualID: DHJPAR0003933; individualCount: 1; sex: Male; lifeStage: adult; preparations: pinned; otherCatalogNumbers: ASBE276-06, 03-SRNP-14258, BOLD:AAA8475; occurrenceID: 84741541-BD65-52D5-8AF6-FE4F58F7E9EB; **Taxon:** scientificName: Belvosiaguillermopereirai; phylum: Arthropoda; class: Insecta; order: Diptera; family: Tachinidae; genus: Belvosia; specificEpithet: guillermopereirai; scientificNameAuthorship: Fleming & Woodley, 2023; **Location:** continent: Central America; country: Costa Rica; countryCode: CR; stateProvince: Guanacaste; county: Sector Santa Rosa; locality: Area de Conservacion Guanacaste; verbatimLocality: Area Administrativa; verbatimElevation: 295; verbatimLatitude: 10.8376; verbatimLongitude: -85.6187; verbatimCoordinateSystem: Decimal; decimalLatitude: 10.8376; decimalLongitude: -85.6187; **Identification:** identifiedBy: AJ Fleming; dateIdentified: 2022; **Event:** samplingProtocol: Reared from the larvae of the Sphingidae, Pachyliasyces; verbatimEventDate: 15-Aug-2003; **Record Level:** language: en; institutionCode: CNC; collectionCode: Insects; basisOfRecord: Pinned Specimen
- **Type status:** Paratype. **Occurrence:** occurrenceDetails: http://janzen.sas.upenn.edu; catalogNumber: DHJPAR0001869; recordedBy: D.H. Janzen, W. Hallwachs & Daniel H. Janzen; individualID: DHJPAR0001869; individualCount: 1; sex: Female; lifeStage: adult; preparations: pinned; otherCatalogNumbers: HCIC385-05, 84-SRNP-880,; occurrenceID: 9F953D8C-AA90-5928-BFD2-168B3E487403; **Taxon:** scientificName: Belvosiaguillermopereirai; phylum: Arthropoda; class: Insecta; order: Diptera; family: Tachinidae; genus: Belvosia; specificEpithet: guillermopereirai; scientificNameAuthorship: Fleming & Woodley, 2023; **Location:** continent: Central America; country: Costa Rica; countryCode: CR; stateProvince: Guanacaste; county: Sector Santa Rosa; locality: Area de Conservacion Guanacaste; verbatimLocality: Bosque Humedo; verbatimElevation: 290; verbatimLatitude: 10.8514; verbatimLongitude: -85.608; verbatimCoordinateSystem: Decimal; decimalLatitude: 10.8514; decimalLongitude: -85.608; **Identification:** identifiedBy: AJ Fleming; dateIdentified: 2022; **Event:** samplingProtocol: Reared from the larvae of the Sphingidae, Pachyliaficus; verbatimEventDate: 21-Jul-1984; **Record Level:** language: en; institutionCode: CNC; collectionCode: Insects; basisOfRecord: Pinned Specimen
- **Type status:** Paratype. **Occurrence:** occurrenceDetails: http://janzen.sas.upenn.edu; catalogNumber: DHJPAR0002000; recordedBy: D.H. Janzen, W. Hallwachs & Daniel H. Janzen; individualID: DHJPAR0002000; individualCount: 1; sex: Male; lifeStage: adult; preparations: pinned; otherCatalogNumbers: HCIC516-05, 78-SRNP-55,; occurrenceID: 40D037D9-E692-59C4-A169-B2C4B3755F99; **Taxon:** scientificName: Belvosiaguillermopereirai; phylum: Arthropoda; class: Insecta; order: Diptera; family: Tachinidae; genus: Belvosia; specificEpithet: guillermopereirai; scientificNameAuthorship: Fleming & Woodley, 2023; **Location:** continent: Central America; country: Costa Rica; countryCode: CR; stateProvince: Guanacaste; county: Sector Santa Rosa; locality: Area de Conservacion Guanacaste; verbatimLocality: Bosque Humedo; verbatimElevation: 290; verbatimLatitude: 10.8514; verbatimLongitude: -85.608; verbatimCoordinateSystem: Decimal; decimalLatitude: 10.8514; decimalLongitude: -85.608; **Identification:** identifiedBy: AJ Fleming; dateIdentified: 2022; **Event:** samplingProtocol: Reared from the larvae of the Sphingidae, Pachyliaficus; verbatimEventDate: 10-Jul-1978; **Record Level:** language: en; institutionCode: CNC; collectionCode: Insects; basisOfRecord: Pinned Specimen

#### Description

**Male** (Fig. 49), length: 11–13mm. **Head**: head slightly wider than thorax; vertex 1/3 head width; gena 1/3 of head height, 2/5 of eye height. Fronto-orbital plate black in ground color, lightly covered with gray tomentum giving majority of the plate a glabrous dark gray sheen transitioning to silver; ocellar setae absent at most several hair-like setulae present on ocellar triangle; reclinate orbital seta absent; two rows of frontal setae, black setulae intermingled with setae. Parafacial dark yellow in ground color, densely covered in silver tomentum making the entire surface reflective brilliant silver appearance; bare overall, except for a small number of setulae extending just below lowest frontal setae; facial ridge setose along 1/3–1/2 of its length, with a few sparse hair-like setulae emerging along outer edge of row; gena covered in black setulae. Antenna, pedicel black, concolorous with postpedicel; postpedicel, 1.5X as long as pedicel; arista bare gradually tapered. Palps, yellow-orange throughout and densely covered in short black setulae; slightly clubbed, but gradually tapering to a slight point apically. **Thorax**: black ground color, with light gray tomentum throughout, when viewed dorsally tomentum appears thinner postsuturally, some bronze tomentum on the postalar callosity; scutellum appearing glabrous reddish-black to the naked eye, under microscope bronze tomentum becomes apparent when view on an oblique caudal angle; scutum with four dorsal vittae, becoming more evident under certain angles of light, these broken at suture; lateral surface of thorax densely covered in long black hair-like setulae; chaetotaxy: 3–4 strong setae on postpronotum arranged in a line, acrostichal setae 3:4–6 often with 2 extra setae appearing just adjacent to acrostichal setae; dorsocentral setae 3:4; intra-alar setae 3:3; supra-alar setae 2:3; 4–6 katepisternal setae; scutellum, with 5–6 pairs of long flat marginal setae of subequal length; apical setae absent; complete row of scutellar discal setae just posterior to marginal setae. **Wing**: strongly infuscate, slightly orange at wing base, black basicosta, with some orange along posterior margin; both upper and lower calypters also infuscate concolorous with remainder of wing; wing vein R_4+5_ setose, bearing only 2–3 setulae at base; halteres orange stalk with dark black/brown capitulum. **Legs**: black overall, coxa on midleg and hindleg with a few reddish-yellow setulae; tarsal claws yellow with black tips, with yellow pulvilli 2/3 length of tarsal claws; Anterodorsal row of setae on hind tibia fringelike, formed by a very regular row of uniformly sized setae separated from each other by less than the width of their socket. **Abdomen**: globose, with dark burgundy-black ground color; abdominal tomentosity on T1+2-T3 bronze, confined to the anterior margin of the tergite, at most anterior 10% of surface, T4 with gold tomentum over anterior 1/3 of the surface, T5 densely gold tomentose on 90% of surface absent along posterior 10%, which appears as glabrous black; middorsal depression on ST1+2 reaching to hind margin of tergite, median marginal setae present on ST1+2 wide set, stout but short, approximately 1/2 as long as median marginals on T3, T3 also with 1 pair of median marginal setae, very strongly appressed to abdomen, and complete rows of marginal setae on T4 and T5; ventral surfaces of T3–T4 with clearly defined sex-patches extending from underside of tergite to lateral surface.

**Male terminalia** (Fig. 50): sternite 5 with a deeply excavated median cleft along posterior edge, Y-shaped, margins covered in dense tomentum; posterior lobes rounded apically, with multiple strong setulae surrounded by many shorter weaker hair-like setulae. Anterior plate of sternite 5, 1/2 as long as posterior lobes; unsclerotized "window" on anterior plate of sternite 5 translucent directly basal to posterior lobes, rectangular shaped, with a slight bow to basal edge. Cerci in posterior view broadly triangular, longer than surstyli; blunt and rounded at apex, fused medially along 1/2 of their length. Cerci in lateral view, with a strong anterior curve arc beginning on anterior 1/3 to apex; cerci densely setose along basal 2/3rds, setae becoming 2x as long on basal 1/2. Surstylus in lateral view, almost equilateral along appearing digitiform rounded apically; surstylus appearing to be separate and not fused with epandrium; when viewed posteriorly surstyli slightly convergent. Pregonite usually broad, well-developed, apically squared off or rounded, usually blunt, basally setulose. Postgonite, slightly narrowed, 1/3 as wide as pregonite, sharply pointed and curved at apex, typically short and scythelike, with few exceptions where postgonite is subequal in length to pregonite. Distiphallus broadly cone-shaped (in some species this cone or flare is much more pronounced, in others appearing square or barrel shaped), with a slender median longitudinal sclerotized reinforcement on its posterior surface and a broad, anterolateral, sclerotized acrophallus, on anterior surface near apex, 1.6X as long as basiphallus.

**Female** (Fig. 51) length: 11–13mm, overall morphology as in male differing in the following traits: **Head**: fronto-orbital plate dull gray, sometimes appearing devoid of tomentum along vertex, bearing 3–4 pairs of proclinate orbital setae in addition to 1–2 pairs of reclinate orbital seta; profile of head not rounded as in males. **Thorax**: Thoracic chaetotaxy: acrostichal setae 3:4; dorsocentral setae 3:4; intra-alar setae 2:3; supra-alar setae 2:3. **Abdomen**: more globose than males, lacking the flattened character, setulae on abdomen not as dense appearing far less hirsute than male abdomen; differing in terminalia, and T3 bearing goldish tomentum on ventral surface.

#### Diagnosis

*Belvosiaguillermopereirai* **sp. n.** can be distinguished from all other *Belvosia* by the following combination of traits: gena covered in black setulae, black basicosta, both calypters dark, anterodorsal setae on hind tibia comblike and regular, and T5 black apically, sex patch present; male terminalia: epandrium densely hirsute, surstyli digitiform and apically rounded, subequal to length of cerci.

#### Etymology

*Belvosiaguillermopereirai* **sp. n**, is named in honor of Sr. Guillermo Pereira in recognition of his decades of being part of the Parataxonomist Program of Area de Conservación Guanacaste (http://www.acguanacaste.ac.cr) in northwestern Costa Rica (Janzen and Hallwachs 2011). Interim species-specific name included in previously circulating databases and publications, *Belvosia* Woodley07C.

#### Distribution

Costa Rica, ACG, Alajuela and Guanacaste Provinces, 10–620m elevation

#### Ecology

*Belvosiaguillermopereirai* **sp. n.** has been reared 34 times from six species of Lepidoptera in the family Sphingidae, *Erinnyisalope* (Drury, 1773) (N=4), *E.ello* (Linnaeus, 1758) (N=2), *E.oenotrus* (Cramer, 1780) (N=1), *Pachyliaficus* (Linnaeus, 1758) (N=11), *P.syces* (Hübner, 1819) (N=15), *Xylophaneschiron* (Drury, 1773) (N=1) in dry foresrt, rain forest, and dry-rain lowland intergrade.

### Belvosia harryramirezi

Fleming & Woodley sp. nov.

B56026DC-5937-5BDB-85E6-58154BF61CDF

DF6EB84D-00F6-494D-B911-4B95E02B1D3F

#### Materials

- **Type status:** Holotype. **Occurrence:** occurrenceDetails: http://janzen.sas.upenn.edu; catalogNumber: DHJPAR0001857; recordedBy: D.H. Janzen, W. Hallwachs & gusaneros; individualID: DHJPAR0001857; individualCount: 1; sex: Male; lifeStage: adult; preparations: pinned; otherCatalogNumbers: HCIC373-05, 93-SRNP-2982,; occurrenceID: CC920D16-287A-59FD-A8DA-87582E9E21EF; **Taxon:** scientificName: Belvosiaharryramirezi; phylum: Arthropoda; class: Insecta; order: Diptera; family: Tachinidae; genus: Belvosia; specificEpithet: harryramirezi; scientificNameAuthorship: Fleming & Woodley, 2023; **Location:** continent: Central America; country: Costa Rica; countryCode: CR; stateProvince: Guanacaste; county: Sector Santa Rosa; locality: Area de Conservacion Guanacaste; verbatimLocality: Area Administrativa; verbatimElevation: 295; verbatimLatitude: 10.8376; verbatimLongitude: -85.6187; verbatimCoordinateSystem: Decimal; decimalLatitude: 10.8376; decimalLongitude: -85.6187; **Identification:** identifiedBy: AJ Fleming; dateIdentified: 2022; **Event:** samplingProtocol: Reared from the larvae of the Sphingidae, Callionimafalcifera; verbatimEventDate: 26-Jul-1993; **Record Level:** language: en; institutionCode: CNC; collectionCode: Insects; basisOfRecord: Pinned Specimen
- **Type status:** Paratype. **Occurrence:** occurrenceDetails: http://janzen.sas.upenn.edu; catalogNumber: DHJPAR0001972; recordedBy: D.H. Janzen, W. Hallwachs & gusaneros; individualID: DHJPAR0001972; individualCount: 1; sex: Male; lifeStage: adult; preparations: pinned; otherCatalogNumbers: HCIC488-05, 87-SRNP-383, BOLD:ABY4918; occurrenceID: 35758F6C-9406-59AE-BAE0-B3AF34CA644C; **Taxon:** scientificName: Belvosiaharryramirezi; phylum: Arthropoda; class: Insecta; order: Diptera; family: Tachinidae; genus: Belvosia; specificEpithet: harryramirezi; scientificNameAuthorship: Fleming & Woodley, 2023; **Location:** continent: Central America; country: Costa Rica; countryCode: CR; stateProvince: Guanacaste; county: Sector Santa Rosa; locality: Area de Conservacion Guanacaste; verbatimLocality: Cafetal; verbatimElevation: 280; verbatimLatitude: 10.8583; verbatimLongitude: -85.6109; verbatimCoordinateSystem: Decimal; decimalLatitude: 10.8583; decimalLongitude: -85.6109; **Identification:** identifiedBy: AJ Fleming; dateIdentified: 2022; **Event:** samplingProtocol: Reared from the larvae of the Sphingidae, Aellopostitan; verbatimEventDate: 10-Jul-1987; **Record Level:** language: en; institutionCode: CNC; collectionCode: Insects; basisOfRecord: Pinned Specimen
- **Type status:** Paratype. **Occurrence:** occurrenceDetails: http://janzen.sas.upenn.edu; catalogNumber: DHJPAR0061268; recordedBy: D.H. Janzen, W. Hallwachs & Keiner Aragon; individualID: DHJPAR0061268; individualCount: 1; sex: Female; lifeStage: adult; preparations: pinned; otherCatalogNumbers: ACGBA7651-17, 17-SRNP-45650, BOLD:ABY4918; occurrenceID: 25D8F9ED-BC3D-539B-9C84-32C5CA356426; **Taxon:** scientificName: Belvosiaharryramirezi; phylum: Arthropoda; class: Insecta; order: Diptera; family: Tachinidae; genus: Belvosia; specificEpithet: harryramirezi; scientificNameAuthorship: Fleming & Woodley, 2023; **Location:** continent: Central America; country: Costa Rica; countryCode: CR; stateProvince: Alajuela; county: Sector Rincon Rain Forest; locality: Area de Conservacion Guanacaste; verbatimLocality: Palomo; verbatimElevation: 96; verbatimLatitude: 10.9619; verbatimLongitude: -85.2804; verbatimCoordinateSystem: Decimal; decimalLatitude: 10.9619; decimalLongitude: -85.2804; **Identification:** identifiedBy: AJ Fleming; dateIdentified: 2022; **Event:** samplingProtocol: Reared from the larvae of the Sphingidae, Xylophaneschiron; verbatimEventDate: 20-Jun-2017; **Record Level:** language: en; institutionCode: CNC; collectionCode: Insects; basisOfRecord: Pinned Specimen

#### Description

**Male** (Fig. 52) , length: 14–16mm. **Head**: head slightly wider than thorax; vertex 1/3 head width; gena 1/5 of head height, 1/4 of eye height. Fronto-orbital plate light black in ground color, lightly covered with gray tomentum giving majority of the plate a glabrous dark gray sheen transitioning to silver; ocellar setae absent at most several hair-like setulae present on ocellar triangle; reclinate orbital seta absent; two rows of frontal setae, black setulae intermingled with setae. Parafacial dark yellow in ground color, densely covered in silver tomentum making the entire surface reflective brilliant silver appearance; bare overall, except for a small number of setulae extending just below lowest frontal setae; facial ridge setose along 1/2 of its length, with a few sparse hair-like setulae emerging along outer edge of row; gena covered in black setulae. Antenna, pedicel black, concolorous with postpedicel; postpedicel, 1.5X as long as pedicel; arista bare and tapered. Palps, yellow-orange throughout and densely covered in short black setulae; twice as thick along anterior 1/2 appearing like a broad club, with a gradual taper apically. **Thorax**: black ground color, with light gray tomentum throughout, when viewed dorsally tomentum appears thinner postsuturally, transitioning to yellow ground color directly adjacent to scutellum, some bronze tomentum on the postalar callosity; scutellum appearing reddish-black to the naked eye, under microscope bronze tomentum becomes apparent when view on an oblique caudal angle; scutum with four dorsal vittae, becoming more evident under certain angles of light, these broken at suture; lateral surface of thorax densely covered in long black hair-like setulae, with some reddish brown setulae intermingled; chaetotaxy: 3 strong setae on postpronotum arranged in a line, acrostichal setae 3:4–6 often with 2 extra setae appearing just adjacent to acrostichal setae; dorsocentral setae 3:4; intra-alar setae 3:3; supra-alar setae 2:3; 4–6 katepisternal setae; scutellum, with 5–6 pairs of long flat marginal setae of subequal length; apical setae absent; complete row of scutellar discal setae just posterior to marginal setae. **Wing**: strongly infuscate, black basicosta, with some orange along posterior margin; both upper and lower calypters infuscate concolorous with remainder of wing; wing vein R_4+5_ setose, bearing only 2–3 setulae at base; halteres orange stalk with dark black/brown capitulum. **Legs**: black overall, coxa on midleg and hindleg with a few reddish-yellow setulae; tarsal claws yellow with black tips, with yellow pulvilli 2/3 length of tarsal claws; Anterodorsal row of setae on hind tibia fringelike, formed by a very regular row of uniformly sized setae separated from each other by less than the width of their socket. **Abdomen**: globose, with dark burgundy-black ground color; T3 with bronze tomentum, confined to the anterior margin of the tergite, at most anterior 10% of surface, T4 gold tomentose along anterior 10-15% of tergite and T5 densely gold tomentose on 95% of surface absent along posterior 5%, which appears as glabrous black; middorsal depression on ST1+2 reaching to hind margin of tergite, median marginal setae present on ST1+2 wide set, stout but short, approximately 1/2 as long as median marginals on T3, T3 also with 1 pair of median marginal setae, and complete rows of marginal setae on T4 and T5; ventral surfaces of T3–T4 with clearly defined sex-patches extending from underside of tergite to lateral surface.

**Male terminalia** (Fig. 53): sternite 5 with a deeply excavated median cleft along posterior edge, elongate, somewhat U shaped with a shoulder midway along cleft, margins covered in dense tomentum; posterior lobes rounded apically, hirsute, with multiple strong setae surrounded by many shorter weaker setulae. Anterior plate of sternite 5 1/2X as long as posterior lobes; unsclerotized "window" on anterior plate of sternite 5 almost entirely transparent directly basal to posterior lobes, roughly "W" shaped. Cerci in posterior view triangular, longer than surstyli; slightly rounded at apex, completely separate medially along distal 1/2. Cerci in lateral view, with a strong anterior curve on anterior 1/2, giving it an overall arced appearance; densely setose along basal 1/2. Surstylus in lateral view, almost equilateral along its entire length making the structure appear digitiform; surstylus appearing to be fused with epandrium; when viewed posteriorly surstyli divergent with outward curved apices. Pregonite broad, yet stout, well-developed, apically rounding off, usually blunt, with 2-4 strong marginal setulae. Postgonite, slightly narrowed, 1/3 as wide as pregonite, 1/2 as long as pregonite. Distiphallus broadly cone-shaped (in some species this cone or flare is much more pronounced, in others appearing square or barrel shaped), with a slender median longitudinal sclerotized reinforcement on its posterior surface, with a strong hook at tip, and a broad, anterolateral, sclerotized acrophallus, on anterior surface near apex, ~1.3X as long as basiphallus.

**Female** (Fig. 54) length: 14–16mm, overall morphology as in male differing in the following traits: **Head**: fronto-orbital plate dull gray, sometimes appearing devoid of tomentum along vertex, bearing 3–4 pairs of proclinate orbital setae in addition to 1 pair of reclinate orbital seta. **Thorax**: Thoracic chaetotaxy: acrostichal setae 4:4; dorsocentral setae 3:4; intra-alar setae 2:3; supra-alar setae 2:3. **Abdomen**: more globose than males, lacking the flattened character, setulae on abdomen not as dense, appearing far less hirsute than male abdomen (lacking sex patch); differing in terminalia, and T3 bearing goldish tomentum on ventral surface.

#### Diagnosis

*Belvosiaharryramirezi* **sp. n.** can be distinguished from all other *Belvosia* by the following combination of traits: gena covered in black setulae, black basicosta, both calypters dark, anterodorsal setae on hind tibia comblike and regular, and T5 black apically, sex patch present; male terminalia: epandrium densely hirsute, surstyli digitiform and apically rounded, subequal to length of cerci.

#### Etymology

*Belvosiaharryramirezi* **sp. n**, is named in honor of Sr. Harry Ramirez in recognition of his decades of being part of the Parataxonomist Program of Area de Conservación Guanacaste (http://www.acguanacaste.ac.cr) in northwestern Costa Rica (Janzen and Hallwachs 2011). Interim species-specific name included in previously circulating databases and publications, *Belvosia* Woodley07D.

#### Distribution

Costa Rica, ACG, Alajuela and Guanacaste Provinces, 40–645m elevation

#### Ecology

*Belvosiaharryramirezi* **sp. n.** has been reared 30 times from seven species of Lepidoptera in the family Sphingidae, *Aelloposfadus* (Cramer, 1775) (N=1), *A.titan* (Cramer, 1777) (N=5), *Callionimafalcifera* (Gehlen, 1943) (N=5), *Cautethiaspuria* (Boisduval, 1875) (N=1), *C.yucatana* Clark, 1919 (N=10), *Xylophanesanubus* (Cramer, 1777) (N=1), *X.chiron* (Drury, 1773) (N=7), in dry foresrt, rain forest, and dry-rain lowland intergrade.

### Belvosia hazelcambroneroae

Fleming && Woodley sp. nov.

B0A5B097-D822-5DCD-A081-34C067F7D734

63E09888-374F-4ECE-9386-80CC55F15F75

#### Materials

- **Type status:** Holotype. **Occurrence:** occurrenceDetails: http://janzen.sas.upenn.edu; catalogNumber: DHJPAR0001979; recordedBy: D.H. Janzen, W. Hallwachs & Daniel H. Janzen; individualID: DHJPAR0001979; individualCount: 1; sex: Male; lifeStage: adult; preparations: pinned; otherCatalogNumbers: HCIC495-05, 84-SRNP-456b, BOLD:AAB5651; occurrenceID: DDACD0AE-12DF-5F43-ACFB-075831B79672; **Taxon:** scientificName: Belvosiahazelcambroneroae; phylum: Arthropoda; class: Insecta; order: Diptera; family: Tachinidae; genus: Belvosia; specificEpithet: hazelcambroneroae; scientificNameAuthorship: Fleming & Woodley, 2023; **Location:** continent: Central America; country: Costa Rica; countryCode: CR; stateProvince: Guanacaste; county: Sector Santa Rosa; locality: Area de Conservacion Guanacaste; verbatimLocality: Bosque Humedo; verbatimElevation: 290; verbatimLatitude: 10.8514; verbatimLongitude: -85.608; verbatimCoordinateSystem: Decimal; decimalLatitude: 10.8514; decimalLongitude: -85.608; **Identification:** identifiedBy: AJ Fleming; dateIdentified: 2022; **Event:** samplingProtocol: Reared from the larvae of the Sphingidae, Aelloposfadus; verbatimEventDate: 17-Jul-1984; **Record Level:** language: en; institutionCode: CNC; collectionCode: Insects; basisOfRecord: Pinned Specimen
- **Type status:** Paratype. **Occurrence:** occurrenceDetails: http://janzen.sas.upenn.edu; catalogNumber: DHJPAR0001838; recordedBy: D.H. Janzen, W. Hallwachs & Guillermo Pereira; individualID: DHJPAR0001838; individualCount: 1; sex: Female; lifeStage: adult; preparations: pinned; otherCatalogNumbers: HCIC354-05, 98-SRNP-8093, BOLD:AAB5651; occurrenceID: 9D468073-F0B0-5C6A-B0F2-979E5B85B24D; **Taxon:** scientificName: Belvosiahazelcambroneroae; phylum: Arthropoda; class: Insecta; order: Diptera; family: Tachinidae; genus: Belvosia; specificEpithet: hazelcambroneroae; scientificNameAuthorship: Fleming & Woodley, 2023; **Location:** continent: Central America; country: Costa Rica; countryCode: CR; stateProvince: Guanacaste; county: Sector Santa Rosa; locality: Area de Conservacion Guanacaste; verbatimLocality: Quebrada Costa Rica; verbatimElevation: 275; verbatimLatitude: 10.8274; verbatimLongitude: -85.6365; verbatimCoordinateSystem: Decimal; decimalLatitude: 10.8274; decimalLongitude: -85.6365; **Identification:** identifiedBy: AJ Fleming; dateIdentified: 2022; **Event:** samplingProtocol: Reared from the larvae of the Sphingidae, Aelloposfadus; verbatimEventDate: 20-Jul-1998; **Record Level:** language: en; institutionCode: CNC; collectionCode: Insects; basisOfRecord: Pinned Specimen
- **Type status:** Paratype. **Occurrence:** occurrenceDetails: http://janzen.sas.upenn.edu; catalogNumber: DHJPAR0001868; recordedBy: D.H. Janzen, W. Hallwachs & Daniel H. Janzen; individualID: DHJPAR0001868; individualCount: 1; sex: Male; lifeStage: adult; preparations: pinned; otherCatalogNumbers: HCIC384-05, 84-SRNP-1461, BOLD:AAB5651; occurrenceID: 97E0AE1B-3797-55DA-82D4-DA312088AD2C; **Taxon:** scientificName: Belvosiahazelcambroneroae; phylum: Arthropoda; class: Insecta; order: Diptera; family: Tachinidae; genus: Belvosia; specificEpithet: hazelcambroneroae; scientificNameAuthorship: Fleming & Woodley, 2023; **Location:** continent: Central America; country: Costa Rica; countryCode: CR; stateProvince: Guanacaste; county: Sector Santa Rosa; locality: Area de Conservacion Guanacaste; verbatimLocality: Bosque San Emilio; verbatimElevation: 300; verbatimLatitude: 10.8439; verbatimLongitude: -85.6138; verbatimCoordinateSystem: Decimal; decimalLatitude: 10.8439; decimalLongitude: -85.6138; **Identification:** identifiedBy: AJ Fleming; dateIdentified: 2022; **Event:** samplingProtocol: Reared from the larvae of the Sphingidae, Erinnyisoenotrus; verbatimEventDate: 11-Jul-1984; **Record Level:** language: en; institutionCode: CNC; collectionCode: Insects; basisOfRecord: Pinned Specimen

#### Description

**Male** (Fig. 55) , length: 14–15mm. **Head**: head slightly wider than thorax; vertex 1/3 head width; gena 1/4 of head height, 2/5 of eye height. Fronto-orbital plate dark gray in ground color, lightly covered with gray tomentum giving majority of the plate a glabrous dark gray sheen transitioning to silver; ocellar setae absent at most several hair-like setulae present on ocellar triangle; reclinate orbital seta absent; two rows of frontal setae, black setulae intermingled with setae. Parafacial dark yellow in ground color, densely covered in silver tomentum making the entire surface reflective brilliant silver appearance; bare overall, except for a small number of black setulae extending just below lowest frontal setae; facial ridge setose along 1/2 of its length, with a few sparse hair-like setulae emerging along outer edge of row; gena covered in black setulae. Antenna, pedicel black, concolorous with postpedicel; postpedicel, 2X as long as pedicel; arista bare distinctly-thickened on basal 4/5 almost to tip. Palps, yellow-orange throughout and densely covered in short black setulae; slightly clubbed. **Thorax**: black ground color, with light gray tomentum throughout, when viewed dorsally tomentum appears thinner postsuturally, scutum transitioning yellow ground color directly adjacent to scutellum, some bronze tomentum on the postalar callosity; scutellum appearing glabrous reddish-orange to the naked eye, under microscope bronze tomentum becomes apparent when viewed on an oblique caudal angle; scutum with four dorsal vittae, becoming more evident under certain angles of light, these broken at suture; lateral surface of thorax densely covered in long black hair-like setulae with some reddish-brown setulae intermingled; chaetotaxy: 3–4 strong setae on postpronotum arranged in a line, acrostichal setae 3:4–6 often with 2 extra setae appearing just adjacent to acrostichal setae; dorsocentral setae 3:4; intra-alar setae 3:3; supra-alar setae 2:3; 4–6 katepisternal setae; scutellum, with 5–6 pairs of long flat marginal setae of subequal length; apical setae absent; complete row of scutellar discal setae just posterior to marginal setae. **Wing**: strongly infuscate, slightly orange at wing base, black basicosta, with some orange along posterior margin; both upper and lower calypters also infuscate concolorous with remainder of wing; wing vein R_4+5_ setose, bearing only 2–3 setulae at base; halteres orange stalk with dark black/brown capitulum. **Legs**: black overall, coxa on midleg and hindleg with a few reddish-yellow setulae; tarsal claws yellow with black tips, with yellow pulvilli 2/3 length of tarsal claws; Anterodorsal row of setae on hind tibia fringelike, formed by a very regular row of uniformly sized setae separated from each other by less than the width of their socket. **Abdomen**: globose, with dark burgundy-black ground color; abdominal tomentosity on T3 gold along anterior 10% of tergite, when viewed caudally bronze tomentum becomes apparent. T4 gold tomentose along anterior 60% of tergite, T5 densely gold tomentose on 95% of surface absent along posterior 5%, which appears as glabrous black; middorsal depression on ST1+2 reaching to hind margin of tergite, median marginal setae present on ST1+2 wide set, short and weak, approximately 1/2 as long as median marginals on T3, T3 also with 1 pair of median marginal setae, and complete rows of marginal setae on T4 and T5; ventral surfaces of T3–T4 with clearly defined sex-patches extending from underside of tergite to lateral surface.

**Male terminalia** (Fig. 56) : sternite 5 with a deeply excavated median cleft along posterior edge, vaguely Y-shaped with a soft shoulder present midway, margins covered in dense tomentum; posterior lobes rounded apically, with multiple strong hair-like setae surrounded by many shorter weaker setulae. Anterior plate of sternite 5 1/2 as long as posterior lobes; unsclerotized "window" on anterior plate of sternite 5 translucent directly basal to posterior lobes, rectangular with apices upturned giving the entire structure a flattened "w" shape. Cerci in posterior view triangular, 2X as long as wide tapering to point, length equal to surstyli; pointed at apex, separate along half its length. Cerci in lateral view, with a strong anterior curve at apex, giving it a hooked appearance; cerci densely setose along basal 2/3rds. Surstylus in lateral view, almost equilateral along its length with a slight arc overall posterior margin rounded making the structure appear bladelike; surstylus appearing to be separate and not fused with epandrium; when viewed posteriorly surstyli straight. Pregonite broad, well-developed, apically squared devoid of setulae. Postgonite, slightly narrowed, 1/3 as wide as pregonite, pointed at apex, subequal in length to pregonite. Distiphallus broadly cone-shaped (in some species this cone or flare is much more pronounced, in others appearing square or barrel shaped), with a slender median longitudinal sclerotized reinforcement on its posterior surface and a broad, anterolateral, sclerotized acrophallus, on anterior surface near apex, ~1.4X as long as basiphallus.

**Female** (Fig. 57) length: 14–15mm, overall morphology as in male differing in the following traits: **Head**: fronto-orbital plate dull gray, sometimes appearing devoid of tomentum along vertex, bearing 4–6 pairs of proclinate orbital setae in addition to 1–2 pairs of reclinate orbital seta; gena 1/4 of head height and 1/3 of eye height. **Thorax**: Thoracic chaetotaxy: acrostichal setae 3:4; dorsocentral setae 3:4; intra-alar setae 2:3; supra-alar setae 2:3. **Abdomen**: more globose than males, lacking the flattened character, setulae on abdomen not as dense appearing far less hirsute than male abdomen; differing in terminalia, and T3 bearing bronze tomentum on ventral surface.

#### Diagnosis

*Belvosiahazelcambroneroae* **sp. n.** can be distinguished from all other *Belvosia* by the following combination of traits: fronto-orbital plate dark gray, gena 2/5 of eye height, with a row of 5–10 small setulae directly anterior to postocular row, scutum with light gray tomentum throughout, tomentum appearing thinner postsuturally, both calypters infuscate, black basicosta, and apex of T5 black tomentose.

#### Etymology

*Belvosiahazelcambroneroae* **sp. n**, is named in honor of Sra. Hazel Cambronero in recognition of her decades of being part of the Parataxonomist Program of Area de Conservación Guanacaste (http://www.acguanacaste.ac.cr) in northwestern Costa Rica (Janzen and Hallwachs 2011). Interim species-specific name included in previously circulating databases and publications, *Belvosia* Woodley07E.

#### Distribution

Costa Rica, ACG, Guanacaste Province, 220–480m elevation

#### Ecology

*Belvosiahazelcambroneroae* **sp. n.** has been reared 38 times from four species of Lepidoptera in two families Sphingidae, *Aelloposfadus* (Cramer, 1775) (N=28), *Erinnyisoenotrus* (Cramer, 1780) (N=8), *Nyceryxcoffaeae* (Walker, 1856) (N=1), and one species of Erebidae, *Parathyriscedonulli* (Stoll, 1781) (N=1), in dry foresrt, rain forest, and dry-rain lowland intergrade.

### Belvosia jorgehernandezi

Fleming & Woodley sp. nov.

094832DF-6964-5E1C-A4E1-136005792711

34806FBE-487F-46DD-AB0C-921E1E9A36B5

#### Materials

- **Type status:** Holotype. **Occurrence:** occurrenceDetails: http://janzen.sas.upenn.edu; catalogNumber: DHJPAR0001288; recordedBy: D.H. Janzen, W. Hallwachs & Harry Ramirez; individualID: DHJPAR0001288; individualCount: 1; sex: Male; lifeStage: adult; preparations: pinned; otherCatalogNumbers: HCIC129-05, 00-SRNP-9157, BOLD:AER5028; occurrenceID: 932FA7FD-A8B1-5EFE-B74D-B5A8637046FC; **Taxon:** scientificName: Belvosiajorgehernandezi; phylum: Arthropoda; class: Insecta; order: Diptera; family: Tachinidae; genus: Belvosia; specificEpithet: jorgehernandezi; scientificNameAuthorship: Fleming & Woodley, 2023; **Location:** continent: Central America; country: Costa Rica; countryCode: CR; stateProvince: Guanacaste; county: Sector Cacao; locality: Area de Conservacion Guanacaste; verbatimLocality: Estacion Cacao; verbatimElevation: 1150; verbatimLatitude: 10.9269; verbatimLongitude: -85.4682; verbatimCoordinateSystem: Decimal; decimalLatitude: 10.9269; decimalLongitude: -85.4682; **Identification:** identifiedBy: AJ Fleming; dateIdentified: 2022; **Event:** samplingProtocol: Reared from the larvae of the Sphingidae, Xylophanestersa; verbatimEventDate: 25-Apr-2000; **Record Level:** language: en; institutionCode: CNC; collectionCode: Insects; basisOfRecord: Pinned Specimen
- **Type status:** Paratype. **Occurrence:** occurrenceDetails: http://janzen.sas.upenn.edu; catalogNumber: DHJPAR0054963; recordedBy: D.H. Janzen, W. Hallwachs & Calixto Moraga; individualID: DHJPAR0054963; individualCount: 1; sex: Female; lifeStage: adult; preparations: pinned; otherCatalogNumbers: ASHYH1510-14, 14-SRNP-30163, BOLD:AER5028; occurrenceID: 2BB19429-0D05-587E-85EF-3DB40A705502; **Taxon:** scientificName: Belvosiajorgehernandezi; phylum: Arthropoda; class: Insecta; order: Diptera; family: Tachinidae; genus: Belvosia; specificEpithet: jorgehernandezi; scientificNameAuthorship: Fleming & Woodley, 2023; **Location:** continent: Central America; country: Costa Rica; countryCode: CR; stateProvince: Guanacaste; locality: Area de Conservacion Guanacaste; verbatimLocality: Sendero Carica; verbatimElevation: 660; verbatimLatitude: 10.9928; verbatimLongitude: -85.4294; verbatimCoordinateSystem: Decimal; decimalLatitude: 10.9928; decimalLongitude: -85.4294; **Identification:** identifiedBy: AJ Fleming; dateIdentified: 2022; **Event:** samplingProtocol: Reared from the larvae of the Sphingidae, Xylophanestersa; verbatimEventDate: 02-Apr-2014; **Record Level:** language: en; institutionCode: CNC; collectionCode: Insects; basisOfRecord: Pinned Specimen
- **Type status:** Paratype. **Occurrence:** occurrenceDetails: http://janzen.sas.upenn.edu; catalogNumber: DHJPAR0059127; recordedBy: D.H. Janzen, W. Hallwachs & Cirilo Umana; individualID: DHJPAR0059127; individualCount: 1; sex: Male; lifeStage: adult; preparations: pinned; otherCatalogNumbers: ACGBA5544-16, 16-SRNP-75211, BOLD:AER5028; occurrenceID: 288976BE-F80B-5AA5-B34D-BE89FD0B7D8A; **Taxon:** scientificName: Belvosiajorgehernandezi; phylum: Arthropoda; class: Insecta; order: Diptera; family: Tachinidae; genus: Belvosia; specificEpithet: jorgehernandezi; scientificNameAuthorship: Fleming & Woodley, 2023; **Location:** continent: Central America; country: Costa Rica; countryCode: CR; stateProvince: Alajuela; county: Sector Rincon Rain Forest; locality: Area de Conservacion Guanacaste; verbatimLocality: Finca Esmeralda; verbatimElevation: 123; verbatimLatitude: 10.9355; verbatimLongitude: -85.2531; verbatimCoordinateSystem: Decimal; decimalLatitude: 10.9355; decimalLongitude: -85.2531; **Identification:** identifiedBy: AJ Fleming; dateIdentified: 2022; **Event:** samplingProtocol: Reared from the larvae of the Sphingidae, Xylophanescthulhu; verbatimEventDate: 28-Mar-2016; **Record Level:** language: en; institutionCode: CNC; collectionCode: Insects; basisOfRecord: Pinned Specimen

#### Description

**Male** (Fig. 58) , length: 14–16mm. **Head**: head slightly wider than thorax; vertex 1/3 head width; gena 1/4 of head height, 1/3 of eye height. Fronto-orbital plate light black in ground color, lightly covered with gray tomentum giving majority of the plate a glabrous dark gray sheen transitioning to silver; ocellar setae absent at most several hair-like setulae present on ocellar triangle; reclinate orbital seta absent; two rows of frontal setae, black setulae intermingled with setae. Parafacial dark yellow in ground color, densely covered in silver tomentum making the entire surface reflective brilliant silver appearance; bare overall, except for a small number of setulae extending just below lowest frontal setae; facial ridge setose along 1/2 of its length, with a few sparse hair-like setulae emerging along outer edge of row; gena covered in black setulae. Antenna, pedicel black, concolorous with postpedicel; postpedicel, 2X as long as pedicel; arista bare distinctly-thickened on basal 4/5 almost to tip. Palps, yellow-orange throughout and densely covered in short black setulae; slightly clubbed, but gradually tapering to a slight point apically. **Thorax**: black ground color, transition to dark burnt orange adjadcent to scutellem with light gray tomentum throughout, when viewed dorsally tomentum appears thinner postsuturally, bronze tomentum on the postalar callosity; scutellum appearing reddish-black to the naked eye, under microscope bronze tomentum becomes apparent when view on an oblique caudal angle; scutum with four dorsal vittae, becoming more evident under certain angles of light, these broken at suture; lateral surface of thorax densely covered in long black hair-like setulae; chaetotaxy: 3 strong setae on postpronotum arranged in a line, acrostichal setae 3:4 often with 2 extra setae appearing just adjacent to acrostichal setae; dorsocentral setae 3:4; intra-alar setae 3:3; supra-alar setae 2:3; 4–6 katepisternal setae; scutellum, with 5–6 pairs of long flat marginal setae of subequal length; apical setae absent; complete row of scutellar discal setae just posterior to marginal setae. **Wing**: strongly infuscate, slightly orange at wing base, black basicosta, with some orange along posterior margin; both upper and lower calypters also infuscate concolorous with remainder of wing; wing vein R_4+5_ setose, bearing only 2–3 setulae at base; halteres orange stalk with dark black/brown capitulum. **Legs**: black overall, coxa on midleg and hindleg with a few reddish-yellow setulae; tarsal claws yellow with black tips, with yellow pulvilli 2/3 length of tarsal claws; anterodorsal row of setae on hind tibia fringelike, formed by a very regular row of uniformly sized setae separated from each other by less than the width of their socket. **Abdomen**: globose, with dark burgundy-black ground color; abdominal tomentosity on T3 bronze confined to the anterior margin of the tergite, at most anterior 10% of surface, T4 with gold tomentosity over anterior 50-60% of tergite, T5 densely gold tomentose on 95% of surface absent along posterior 5%, which appears as glabrous black; middorsal depression on ST1+2 reaching to hind margin of tergite, median marginal setae present on ST1+2 wide set, T3 with 1 pair of median marginal setae, and complete rows of marginal setae on T4 and T5; ventral surfaces of T3–T4 with clearly defined sex-patches extending from underside of tergite to lateral surface.

**Male terminalia** (Fig. 59) : sternite 5 with a deeply excavated median cleft along posterior edge, Y-shaped, margins covered in dense tomentum; posterior lobes rounded apically, either bare, with multiple strong setae surrounded by many shorter weaker setulae. Anterior plate of sternite 5, 1/2 length of posterior lobes; unsclerotized "window" on anterior plate of sternite 5 subrectangular directly basal to posterior lobes, slightly umbonate convex on anterior edge, and a slight upcurve at lateral apices. Cerci in posterior view sharply pointed triangular with a narrow base, length to tips 1.8X basal with, slightly longer than sursyli; apically pointed, separate along anterior 1/2. Cerci in lateral view, with a strong anterior curve on apex, thickened basally tapering to apex; cerci densely setose along basal 2/3rds. Surstylus in lateral view, almost equilateral along its length with no definitive curvature, digitiform; surstylus appearing to be separate and not fused with epandrium; when viewed posteriorly surstyli straight. Pregonite broad, well-developed, apically squared off, with one setula along margin. Postgonite, slightly narrowed, 1/3 as wide as pregonite, sharply pointed and curved at apex. Distiphallus cone-shaped, with a slender median longitudinal sclerotized reinforcement on its posterior surface and a broad, anterolateral, sclerotized acrophallus, on anterior surface near apex, ~1.4X as long as basiphallus; epiphallus when visible, short and rounded, appearing as a small hump on dorsal surface of basiphallus.

**Female** (Fig. 60) length: 14–16mm, overall morphology as in male differing in the following traits: **Head**: fronto-orbital plate dull gray, sometimes appearing devoid of tomentum along vertex, bearing 4–6 pairs of proclinate orbital setae in addition to 1–2 pairs of reclinate orbital setae. **Thorax**: Thoracic chaetotaxy: acrostichal setae 3:4; dorsocentral setae 3:4; intra-alar setae 2:3; supra-alar setae 2:3. **Abdomen**: larger and more flattened than males, setulae on abdomen not as dense appearing far less hirsute than male abdomen; differing in terminalia, T3 with traces of gold tomentum directly posterior to tergite ST1+2, and T4 and T5 bearing gold tomentum throughout including ventral surface.

#### Diagnosis

*Belvosiajorgehernandezi* **sp. n.** can be distinguished from all other *Belvosia* by the following combination of traits: fronto-orbital plate pale silver gray, gena 1/3 of eye height, post sutural scutum mostly silver, both calypters dark, black basicosta, anterodorsal row of setae on hind tibia fringelike, and apex of T5 black tomentose.

#### Etymology

*Belvosiajorgehernandezi* **sp. n**, is named in honor of Sr. Jorge Hernandez in recognition of his decades of being part of the Parataxonomist Program of Area de Conservación Guanacaste (http://www.acguanacaste.ac.cr) in northwestern Costa Rica (Janzen and Hallwachs 2011). Interim species-specific name included in previously circulating databases and publications, *Belvosia* Woodley07F.

#### Distribution

Costa Rica, ACG, Alajuela Province, 2–1150m elevation.

#### Ecology

*Belvosiajorgehernandezi* **sp. n.** has been reared nine times from two species of Lepidoptera in the family Sphingidae, *Xylophanescthulhu* Haxaire & Vaglia, 2008 (N=1), *Xylophanestersa* (Linnaeus, 1771) (N=8), in cloud forest, dry foresrt, and rain forest.

### Belvosia josecortezi

Fleming & Woodley sp. nov.

CF01C48A-B71D-573A-8886-4B28AB8696C2

FC549EB0-05F8-4EFB-A41C-E7631DD074BC

#### Materials

- **Type status:** Holotype. **Occurrence:** occurrenceDetails: http://janzen.sas.upenn.edu; catalogNumber: DHJPAR0029527; recordedBy: D.H. Janzen, W. Hallwachs & Duvalier Briceno; individualID: DHJPAR0029527; individualCount: 1; sex: Male; lifeStage: adult; preparations: pinned; otherCatalogNumbers: ASHYM948-09, 08-SRNP-65800, BOLD:ABY4919; occurrenceID: AA775FC4-5FC9-5EDE-BB88-6BDE07F066ED; **Taxon:** scientificName: Belvosiajosecortezi; phylum: Arthropoda; class: Insecta; order: Diptera; family: Tachinidae; genus: Belvosia; specificEpithet: josecortezi; scientificNameAuthorship: Fleming & Woodley, 2023; **Location:** continent: Central America; country: Costa Rica; countryCode: CR; stateProvince: Alajuela; county: Brasilia; locality: Area de Conservacion Guanacaste; verbatimLocality: Gallinazo; verbatimElevation: 360; verbatimLatitude: 11.0183; verbatimLongitude: -85.372; verbatimCoordinateSystem: Decimal; decimalLatitude: 11.0183; decimalLongitude: -85.372; **Identification:** identifiedBy: AJ Fleming; dateIdentified: 2022; **Event:** samplingProtocol: Reared from the larvae of the Sphingidae, Xylophaneschiron; verbatimEventDate: 21-Sep-2008; **Record Level:** language: en; institutionCode: CNC; collectionCode: Insects; basisOfRecord: Pinned Specimen
- **Type status:** Paratype. **Occurrence:** occurrenceDetails: http://janzen.sas.upenn.edu; catalogNumber: DHJPAR0001999; recordedBy: D.H. Janzen, W. Hallwachs & Daniel H. Janzen; individualID: DHJPAR0001999; individualCount: 1; sex: Male; lifeStage: adult; preparations: pinned; otherCatalogNumbers: HCIC515-05, 82-SRNP-762,; occurrenceID: 33533271-0627-53AB-96B9-AB1DF216C288; **Taxon:** scientificName: Belvosiajosecortezi; phylum: Arthropoda; class: Insecta; order: Diptera; family: Tachinidae; genus: Belvosia; specificEpithet: josecortezi; scientificNameAuthorship: Fleming & Woodley, 2023; **Location:** continent: Central America; country: Costa Rica; countryCode: CR; stateProvince: Guanacaste; county: Sector Santa Rosa; locality: Area de Conservacion Guanacaste; verbatimLocality: Bosque San Emilio; verbatimElevation: 300; verbatimLatitude: 10.8439; verbatimLongitude: -85.6138; verbatimCoordinateSystem: Decimal; decimalLatitude: 10.8439; decimalLongitude: -85.6138; **Identification:** identifiedBy: AJ Fleming; dateIdentified: 2022; **Event:** samplingProtocol: Reared from the larvae of the unknowable, unknowable; **Record Level:** language: en; institutionCode: CNC; collectionCode: Insects; basisOfRecord: Pinned Specimen
- **Type status:** Paratype. **Occurrence:** occurrenceDetails: http://janzen.sas.upenn.edu; catalogNumber: DHJPAR0002003; recordedBy: D.H. Janzen, W. Hallwachs & Daniel H. Janzen; individualID: DHJPAR0002003; individualCount: 1; sex: Female; lifeStage: adult; preparations: pinned; otherCatalogNumbers: HCIC519-05, 82-SRNP-20, BOLD:ABY4919; occurrenceID: 05502304-CE01-5610-B8AF-F6C5F977CEF4; **Taxon:** scientificName: Belvosiajosecortezi; phylum: Arthropoda; class: Insecta; order: Diptera; family: Tachinidae; genus: Belvosia; specificEpithet: josecortezi; scientificNameAuthorship: Fleming & Woodley, 2023; **Location:** continent: Central America; country: Costa Rica; countryCode: CR; stateProvince: Guanacaste; county: Sector Santa Rosa; locality: Area de Conservacion Guanacaste; verbatimLocality: Bosque Humedo; verbatimElevation: 290; verbatimLatitude: 10.8514; verbatimLongitude: -85.608; verbatimCoordinateSystem: Decimal; decimalLatitude: 10.8514; decimalLongitude: -85.608; **Identification:** identifiedBy: AJ Fleming; dateIdentified: 2022; **Event:** samplingProtocol: Reared from the larvae of the unknowable, unknowable; **Record Level:** language: en; institutionCode: CNC; collectionCode: Insects; basisOfRecord: Pinned Specimen

#### Description

**Male** (Fig. 61), length: 12–14mm. **Head**: head slightly wider than thorax; vertex 1/3 head width; gena 1/4 of head height, 2/5 of eye height. Fronto-orbital plate light black in ground color, lightly covered with gray tomentum; ocellar setae absent at most several hair-like setulae present on ocellar triangle; one pair of slightly lateraloclinae orbital seta; two rows of frontal setae, black setulae intermingled with setae. Parafacial dark yellow in ground color, densely covered in silver tomentum making the entire surface reflective brilliant silver appearance; bare overall, except for 2–4 setulae extending just below lowest frontal setae; facial ridge setose along 1/2 of its length, with a few sparse hair-like setulae emerging along outer edge of row; gena covered in black setulae. Antenna, pedicel black, concolorous with postpedicel; postpedicel, 1/2 as long as pedicel; arista bare distinctly-thickened on basal 4/5 almost to tip. Palps, yellow-orange throughout and densely covered in short black setulae; slightly clubbed, but gradually tapering to a slight point apically. **Thorax**: black ground color transitioning to a dark reddish yellow directly adjacent to scutellum, with light gray tomentum throughout, when viewed dorsally tomentum appears thinner postsuturally, some bronze tomentum on the postalar callosity; scutellum appearing reddish-black to the naked eye, under microscope bronze tomentum becomes apparent when view on an oblique caudal angle; scutum with four dorsal vittae, becoming more evident under certain angles of light, these broken at suture; lateral surface of thorax densely covered in long black hair-like setulae; chaetotaxy: 3–4 strong setae on postpronotum arranged in a line, acrostichal setae 3:4 often with 2 extra setae appearing just adjacent to acrostichal setae; dorsocentral setae 3:4; intra-alar setae 3:3; supra-alar setae 2:3; 4–6 katepisternal setae; scutellum, with 5–6 pairs of long flat marginal setae of subequal length; apical setae absent; complete row of scutellar discal setae just posterior to marginal setae. **Wing**: strongly infuscate, slightly orange at wing base, black basicosta, with some orange along posterior margin; both upper and lower calypters also infuscate concolorous with remainder of wing; wing vein R_4+5_ setose, bearing only 2–3 setulae at base; halteres orange stalk with dark black/brown capitulum. **Legs**: black overall, coxa on midleg and hindleg with a few reddish-yellow setulae; tarsal claws yellow with black tips, with yellow pulvilli 2/3 length of tarsal claws; Anterodorsal row of setae on hind tibia fringelike, formed by a very regular row of uniformly sized setae separated from each other by less than the width of their socket. **Abdomen**: globose, with dark burgundy-black ground color; T3 with traces of gold tomentum directly adjacent to ST1+2, T4 with gold tomentum along anterior 60% of tergite, T5 densely gold tomentose on 95% of surface absent along posterior 5%, which appears as glabrous black; middorsal depression on ST1+2 reaching to hind margin of tergite, median marginal setae present on ST1+2 wide set, stout but short, approximately 1/2 as long as median marginals on T3, T3 also with 1 pair of median marginal setae, and complete rows of marginal setae on T4 and T5; ventral surfaces of T3–T4 with clearly defined sex-patches extending from underside of tergite to lateral surface.

**Male terminalia** (Fig. 62): sternite 5 with a deeply excavated median cleft along posterior edge, vaguely Y-shaped with a slight shoulder, margins covered in dense tomentum; posterior lobes rounded apically, with multiple strong setae surrounded by many shorter weaker setulae. Anterior plate of sternite 5. 1/2 length of posterior lobes; unsclerotized "window" on anterior plate translucent directly basal to posterior lobes, appearing slightly arcuate with a curved anteriro surface. Cerci in posterior view, triangular width 2/3 of length, slightly longer than surstyli; rounded at apex separate medially along 1/2 of their length. Cerci in lateral view, often with a strong anterior curve on apex, giving it a curved appearance, terminating in a slight hook; densely setose along basal 2/3rds. Surstylus in lateral view, almost equilateral along its length sometimes with a very slight curve along its length, apically pointed making the structure appear bladelike; surstylus appearing to be separate and not fused with epandrium; when viewed posteriorly surstyli straight. Pregonite usually broad, well-developed, apically squared off or rounded, with 2–5 thin setulae along margin. Postgonite, slightly narrowed, 1/3 as wide as pregonite, blunt and curved at apex. Distiphallus broadly cone-shaped, with a slender median longitudinal sclerotized reinforcement on its posterior surface and a broad, anterolateral, sclerotized acrophallus, on anterior surface near apex, 1.9X as long as basiphallus.

**Female** (Fig. 63) length: 12–14mm, overall morphology as in male differing in the following traits: **Head**: fronto-orbital plate dull gray, sometimes appearing devoid of tomentum along vertex, bearing 4–6 pairs of proclinate orbital setae in addition to 1–2 pairs of reclinate orbital seta; profile of head not rounded as in males; gena 1/4 head height and 1/3 eye height. **Thorax**: Thoracic chaetotaxy: acrostichal setae 3:4; dorsocentral setae 3:4; intra-alar setae 2:3; supra-alar setae 2:3. **Abdomen**: more globose than males, lacking the flattened character, setulae on abdomen not as dense appearing far less hirsute than male abdomen; differing in terminalia, and T3 bearing goldish tomentum on ventral surface.

#### Diagnosis

*Belvosiajosecortezi* **sp. n.** can be distinguished from all other *Belvosia* by the following combination of traits: fronto-orbital plate pale silver gray, gena 2/5 of eye height, covered in black setulae, post sutural scutum mostly silver, both calypters dark, black basicosta with orange along caudal edge, anterodorsal row of setae on hind tibia fringelike and apex of T5 black tomentose.

#### Etymology

*Belvosiajosecortezi* **sp. n**, is named in honor of Sr. Jose Cortez in recognition of his decades of being part of the Parataxonomist Program of Area de Conservación Guanacaste (http://www.acguanacaste.ac.cr) in northwestern Costa Rica (Janzen and Hallwachs 2011). Interim species-specific name included in previously circulating databases and publications, *Belvosia* Woodley07G.

#### Distribution

Costa Rica, ACG, Alajuela and Guanacaste Provinces, 2–660m elevation.

#### Ecology

*Belvosiajosecortezi* **sp. n.** has been reared 51 times from ten species of Lepidoptera in the family Sphingidae, *Callionimadenticulata* (Schaus, 1895) (N=1), *Unzelajapix* (Cramer, 1776) (N=1), *Xylophanesanubus* (Cramer, 1777) (N=3), *X.ceratomioides* (Grote & Robinson, 1867) (N=1), *X.chiron* (Drury, 1773) (N=15), *X.guianensis* (Rothschild, 1894) (N=9), *X.libya*DHJ02 (N=1), *X.pluto* (Fabricius, 1777) (N=16), *X.porcus* (Hübner, 1823) (N=1), *X.zurcheri* (Druce, 1894) (N=1), and two unknwon hosts collected and reared out from pupae, in dry forest, and rain forest, and dry-rain lowland intergrades.

### Belvosia joseperezi

Fleming & Woodley sp. nov.

9DA8F466-0247-5F2F-BDB6-ECE63CB8D1AD

DD6D483A-1B9A-48B7-8BDE-CD2D91B8FE36

#### Materials

- **Type status:** Holotype. **Occurrence:** occurrenceDetails: http://janzen.sas.upenn.edu; catalogNumber: DHJPAR0001853; recordedBy: D.H. Janzen, W. Hallwachs & gusaneros; individualID: DHJPAR0001853; individualCount: 1; lifeStage: adult; preparations: pinned; otherCatalogNumbers: HCIC369-05, 94-SRNP-5253, BOLD:AAA8475; occurrenceID: 4AD1AFBD-0D84-5AB1-9DF7-6D5FC2AC5788; **Taxon:** scientificName: Belvosiajoseperezi; phylum: Arthropoda; class: Insecta; order: Diptera; family: Tachinidae; genus: Belvosia; specificEpithet: joseperezi; scientificNameAuthorship: Fleming & Woodley, 2023; **Location:** continent: Central America; country: Costa Rica; countryCode: CR; stateProvince: Guanacaste; county: Sector Santa Rosa; locality: Area de Conservacion Guanacaste; verbatimLocality: Tanquetas; verbatimElevation: 295; verbatimLatitude: 10.8708; verbatimLongitude: -85.6053; verbatimCoordinateSystem: Decimal; decimalLatitude: 10.8708; decimalLongitude: -85.6053; **Identification:** identifiedBy: AJ Fleming; dateIdentified: 2022; **Event:** samplingProtocol: Reared from the larvae of the Sphingidae, Erinnyisobscura; verbatimEventDate: 19-Aug-1994; **Record Level:** language: en; institutionCode: CNC; collectionCode: Insects; basisOfRecord: Pinned Specimen
- **Type status:** Paratype. **Occurrence:** occurrenceDetails: http://janzen.sas.upenn.edu; catalogNumber: DHJPAR0001965; recordedBy: D.H. Janzen, W. Hallwachs & gusaneros; individualID: DHJPAR0001965; individualCount: 1; lifeStage: adult; preparations: pinned; otherCatalogNumbers: HCIC481-05, 94-SRNP-5251, BOLD:AAA8475; occurrenceID: 1F562A7A-0417-590A-8416-332CDBAC98E9; **Taxon:** scientificName: Belvosiajoseperezi; phylum: Arthropoda; class: Insecta; order: Diptera; family: Tachinidae; genus: Belvosia; specificEpithet: joseperezi; scientificNameAuthorship: Fleming & Woodley, 2023; **Location:** continent: Central America; country: Costa Rica; countryCode: CR; stateProvince: Guanacaste; county: Sector Santa Rosa; locality: Area de Conservacion Guanacaste; verbatimLocality: Tanquetas; verbatimElevation: 295; verbatimLatitude: 10.8708; verbatimLongitude: -85.6053; verbatimCoordinateSystem: Decimal; decimalLatitude: 10.8708; decimalLongitude: -85.6053; **Identification:** identifiedBy: AJ Fleming; dateIdentified: 2022; **Event:** samplingProtocol: Reared from the larvae of the Sphingidae, Erinnyisobscura; verbatimEventDate: 20-Aug-1994; **Record Level:** language: en; institutionCode: CNC; collectionCode: Insects; basisOfRecord: Pinned Specimen
- **Type status:** Paratype. **Occurrence:** occurrenceDetails: http://janzen.sas.upenn.edu; catalogNumber: DHJPAR0057872; recordedBy: D.H. Janzen, W. Hallwachs & Ricardo Calero; individualID: DHJPAR0057872; individualCount: 1; lifeStage: adult; preparations: pinned; otherCatalogNumbers: MHMYK10572-15, 15-SRNP-70959, BOLD:AAA8475; occurrenceID: 1C0F3889-D8D8-5CAA-BF32-921426CEFF76; **Taxon:** scientificName: Belvosiajoseperezi; phylum: Arthropoda; class: Insecta; order: Diptera; family: Tachinidae; genus: Belvosia; specificEpithet: joseperezi; scientificNameAuthorship: Fleming & Woodley, 2023; **Location:** continent: Central America; country: Costa Rica; countryCode: CR; stateProvince: Guanacaste; county: Sector Pitilla; locality: Area de Conservacion Guanacaste; verbatimLocality: Medrano; verbatimElevation: 380; verbatimLatitude: 11.016; verbatimLongitude: -85.3805; verbatimCoordinateSystem: Decimal; decimalLatitude: 11.016; decimalLongitude: -85.3805; **Identification:** identifiedBy: AJ Fleming; dateIdentified: 2022; **Event:** samplingProtocol: Reared from the larvae of the Sphingidae, Erinnyisobscura; verbatimEventDate: 26-Jun-2015; **Record Level:** language: en; institutionCode: CNC; collectionCode: Insects; basisOfRecord: Pinned Specimen

#### Description

**Male** (Fig. 64) : length: 14–15mm. **Head**: head slightly wider than thorax; vertex 1/3 head width; gena 1/3 of head height, 1/2 of eye height. Fronto-orbital plate light black in ground color, lightly covered with gray tomentum giving majority of the plate a dark gray sheen transitioning to silver; ocellar setae absent at most several hair-like setulae present on ocellar triangle; reclinate orbital seta absent; two rows of frontal setae, black setulae intermingled with setae. Parafacial dark yellow in ground color, densely covered in silver tomentum making the entire surface reflective brilliant silver appearance; bare overall, except for a 5–8 black setulae extending just below lowest frontal setae; facial ridge setose along 2/5 of its length, with a few sparse hair-like setulae emerging along outer edge of row; gena covered in black setulae. Antenna, pedicel black, concolorous with postpedicel; postpedicel, 1/2 as long as pedicel; arista bare distinctly-thickened on basal 4/5 almost to tip. Palps, yellow-orange throughout and densely covered in short black setulae; slightly clubbed, but gradually tapering to a slight point apically. **Thorax**: black ground color transitioning to a dark reddish yellow directly adjacent to scutellum, with light gray tomentum throughout, when viewed dorsally tomentum appears thinner postsuturally, some bronze tomentum on the postalar callosity; scutellum appearing reddish-black to the naked eye, under microscope bronze tomentum becomes apparent when view on an oblique caudal angle; scutum with four dorsal vittae, becoming more evident under certain angles of light, these broken at suture; lateral surface of thorax densely covered in long black hair-like setulae; chaetotaxy: 3–4 strong setae on postpronotum arranged in a line, acrostichal setae 3:4 often with 2 extra setae appearing just adjacent to acrostichal setae; dorsocentral setae 3:4; intra-alar setae 3:3; supra-alar setae 2:3; 4–6 katepisternal setae; scutellum, with 5–6 pairs of long flat marginal setae of subequal length; apical setae absent; complete row of scutellar discal setae just posterior to marginal setae. **Wing**: strongly infuscate, slightly orange at wing base, black basicosta, with some orange along posterior margin; both upper and lower calypters also infuscate concolorous with remainder of wing; wing vein R_4+5_ setose, bearing only 2–3 setulae at base; halteres orange stalk with dark black/brown capitulum. **Legs**: black overall, coxa on midleg and hindleg with a few reddish-yellow setulae; tarsal claws yellow with black tips, with yellow pulvilli 2/3 length of tarsal claws; Anterodorsal row of setae on hind tibia fringelike, formed by a very regular row of uniformly sized setae separated from each other by less than the width of their socket. **Abdomen**: globose, with dark burgundy-black ground color; T3 with traces of gold tomentum directly adjacent to ST1+2, T4 with gold tomentum along anterior 60% of tergite, T5 densely gold tomentose on 95% of surface absent along posterior 5%, which appears as glabrous black; middorsal depression on ST1+2 reaching to hind margin of tergite, median marginal setae present on ST1+2 wide set, stout and short, less than 1/2 as long as median marginals on T3, T3 also with 1 pair of median marginal setae, and complete rows of marginal setae on T4 and T5; ventral surfaces of T3–T4 with clearly defined sex-patches extending from underside of tergite to lateral surface.

**Male terminalia** (Fig. 65) : sternite 5 with a deeply excavated median cleft along posterior edge, vaguely Y-shaped, margins covered in dense tomentum; posterior lobes rounded apically, with multiple strong setae surrounded by many shorter weaker setulae. Anterior plate of sternite 5 1/2 as long as posterior lobes; unsclerotized "window" on anterior plate of sternite 5 translucent directly basal to posterior lobes, rectangular arcuate with a slight convex umbo along anterior edge. Cerci in posterior view sharply pointed wide based triangular, length to tips 1.3X basal width, with a strong taper beginning 2/5 down length, equal in length to surstyli; apically pointed, either fused along basal half. Cerci in lateral view, with a slight thickening basally not pronounced, and soft anterior curve on apex, giving it a mildly arcuate appearance; cerci densely setose along basal 2/3rds. Surstylus in lateral view, almost equilateral along its length with slightly anterior curve along its lenght, digitiform; surstylus appearing to be separate and not fused with epandrium; when viewed posteriorly surstyli slightly convergent. Pregonite usually broad, well-developed, apically squared off or rounded, with 3–5 marginal setulae. Postgonite, narrowed, 1/3 as wide as pregonite, blunt and rounded with a curved at apex, subequal in length to pregonite. Distiphallus broadly cone-shaped (in some species this cone or flare is much more pronounced, in others appearing square or barrel shaped), with a slender median longitudinal sclerotized reinforcement on its posterior surface and a broad, anterolateral, sclerotized acrophallus, on anterior surface near apex, ~1.4X as long as basiphallus.

**Female**: unknown at this time.

#### Diagnosis

*Belvosiajoseperezi* **sp. n.** can be distinguished from all other *Belvosia* by the following combination of traits: fronto-orbital plate light grey tomentose, with black ground color clearly visible sometimes appearing glabrous; gena 1/2 of eye height covered in black setulae, both calypters dark, black basicosta, and apex of T5 black tomentose.

#### Etymology

*Belvosiajoseperezi* **sp. n**, is named in honor of Sr. Jose Perez in recognition of his decades of being part of the Parataxonomist Program of Area de Conservación Guanacaste (http://www.acguanacaste.ac.cr) in northwestern Costa Rica (Janzen and Hallwachs 2011). Interim species-specific name included in previously circulating databases and publications, *Belvosia* Woodley07H.

#### Distribution

Costa Rica, ACG, Guanacaste Province, 290–380m elevation.

#### Ecology

*Belvosiajoseperezi* **sp. n.** has been reared four times from one species of Lepidoptera in the family Sphingidae, *Erinnyisobscura* (Fabricius, 1775) (N=4), in dry forest.

### Belvosia keinoraragoni

Fleming & Woodley sp. nov.

5535D972-02EE-5EFC-A0D0-312FFC387964

973B178E-0162-42D6-A9CB-CAAC5358DDF7

#### Materials

- **Type status:** Holotype. **Occurrence:** occurrenceDetails: http://janzen.sas.upenn.edu; catalogNumber: DHJPAR0037236; recordedBy: D.H. Janzen, W. Hallwachs & Guillermo Pereira; individualID: DHJPAR0037236; individualCount: 1; sex: Male; lifeStage: adult; preparations: pinned; otherCatalogNumbers: ASHYC3981-10, 09-SRNP-14321, BOLD:AAB3033; occurrenceID: 4D607B44-170D-5896-9FC6-DE009382CB10; **Taxon:** scientificName: Belvosiakeinoraragoni; phylum: Arthropoda; class: Insecta; order: Diptera; family: Tachinidae; genus: Belvosia; specificEpithet: keinoraragoni; scientificNameAuthorship: Fleming & Woodley, 2023; **Location:** continent: Central America; country: Costa Rica; countryCode: CR; stateProvince: Guanacaste; county: Sector Santa Rosa; locality: Area de Conservacion Guanacaste; verbatimLocality: Camino Borrachos; verbatimElevation: 295; verbatimLatitude: 10.8429; verbatimLongitude: -85.6161; verbatimCoordinateSystem: Decimal; decimalLatitude: 10.8429; decimalLongitude: -85.6161; **Identification:** identifiedBy: AJ Fleming; dateIdentified: 2022; **Event:** samplingProtocol: Reared from the larvae of the Saturniidae, Eacles imperialisDHJ02; verbatimEventDate: 22-Sep-2009; **Record Level:** language: en; institutionCode: CNC; collectionCode: Insects; basisOfRecord: Pinned Specimen
- **Type status:** Paratype. **Occurrence:** occurrenceDetails: http://janzen.sas.upenn.edu; catalogNumber: DHJPAR0001919; recordedBy: D.H. Janzen, W. Hallwachs & gusaneros; individualID: DHJPAR0001919; individualCount: 1; sex: Female; lifeStage: adult; preparations: pinned; otherCatalogNumbers: HCIC435-05, 91-SRNP-380.29, BOLD:AAB3033; occurrenceID: 3F2B37C9-304B-5568-9E4D-FF86C4D72A95; **Taxon:** scientificName: Belvosiakeinoraragoni; phylum: Arthropoda; class: Insecta; order: Diptera; family: Tachinidae; genus: Belvosia; specificEpithet: keinoraragoni; scientificNameAuthorship: Fleming & Woodley, 2023; **Location:** continent: Central America; country: Costa Rica; countryCode: CR; stateProvince: Guanacaste; county: Sector Santa Rosa; locality: Area de Conservacion Guanacaste; verbatimLocality: Bosque Encino Guacimal; verbatimElevation: 285; verbatimLatitude: 10.8688; verbatimLongitude: -85.6023; verbatimCoordinateSystem: Decimal; decimalLatitude: 10.8688; decimalLongitude: -85.6023; **Identification:** identifiedBy: AJ Fleming; dateIdentified: 2022; **Event:** samplingProtocol: Reared from the larvae of the Saturniidae, Eacles imperialisDHJ02; verbatimEventDate: 25-Jul-1991; **Record Level:** language: en; institutionCode: CNC; collectionCode: Insects; basisOfRecord: Pinned Specimen
- **Type status:** Paratype. **Occurrence:** occurrenceDetails: http://janzen.sas.upenn.edu; catalogNumber: DHJPAR0001928; recordedBy: D.H. Janzen, W. Hallwachs & gusaneros; individualID: DHJPAR0001928; individualCount: 1; sex: Male; lifeStage: adult; preparations: pinned; otherCatalogNumbers: HCIC444-05, 87-SRNP-602, BOLD:AAB3033; occurrenceID: 0F3E5350-98E2-5C63-9902-C5969143518D; **Taxon:** scientificName: Belvosiakeinoraragoni; phylum: Arthropoda; class: Insecta; order: Diptera; family: Tachinidae; genus: Belvosia; specificEpithet: keinoraragoni; scientificNameAuthorship: Fleming & Woodley, 2023; **Location:** continent: Central America; country: Costa Rica; countryCode: CR; stateProvince: Guanacaste; county: Sector Santa Rosa; locality: Area de Conservacion Guanacaste; verbatimLocality: Sendero Natural; verbatimElevation: 290; verbatimLatitude: 10.8357; verbatimLongitude: -85.6125; verbatimCoordinateSystem: Decimal; decimalLatitude: 10.8357; decimalLongitude: -85.6125; **Identification:** identifiedBy: AJ Fleming; dateIdentified: 2022; **Event:** samplingProtocol: Reared from the larvae of the Saturniidae, Eacles imperialisDHJ02; verbatimEventDate: 20-Aug-1987; **Record Level:** language: en; institutionCode: CNC; collectionCode: Insects; basisOfRecord: Pinned Specimen

#### Description

**Male** (Fig. 66), length: 11–15mm. **Head**: head wider than thorax; vertex 1/3 head width; gena 1/3 of head height, 2/5 of eye height. Fronto-orbital plate silver with varying tonality of gold tomentum (ranging from very yellow-gold–silver with brassy tones), darkening slightly apically in some cases appearing glabrous or void of tomentum apically, with two rows of frontal setae, populated with short black hair-like setulae intermingled with setae, with a few dark colored setulae extending below lowest frontal seta; ocellar setae weak and slightly lateroclinate, ranging from hair-like to distinctly present, adjacent to anterior ocellus; orbital setae absent. Parafacial light yellow in ground color, densely covered in silver tomentum, entire surface reflective and brilliant appearance; almost bare along parafacial outside facial ridge, with only a small number of setulae extending just below lowest frontal setae; facial ridge setose along 1/2–3/4 of its length, with few black hair-like setulae emerging along outer edge of row; gena covered in black setulae. Antenna, pedicel black, concolorous with postpedicel; postpedicel black, 3X as long as pedicel; arista bare gradually tapering to a point at tip. Palps, orange throughout and densely covered in short black setulae; tapering to a slight point apically, devoid of setulae apically. Vibrissa approximately 2 pedicel lengths from facial margin. **Thorax**: black ground color throughout, except around post-alar callus where it is lighter brown, with light gray tomentum throughout; scutellum ground color light brown almost yellow, distinctly lighter than scutum, under microscope bronze tomentum throughout becomes visible; scutum with four dorsal vittae, one outer pair, one inner pair, both broken at suture; lateral surface of thorax densely covered in long hair-like setulae, these setulae all black; chaetotaxy: 3–4 strong setae on postpronotum arranged in a line, acrostichal setae 3:3–4; dorsocentral setae 3:4; intra-alar setae 3:3; supra-alar setae 2:3; 4–5 katepisternal setae; scutellum, with 4–5 pairs of long marginal setae of subequal length; apical scutellar setae absent; 1 complete row of scutellar discal setae just posterior to marginal setae. **Wing**: infuscate, slightly darkened orange at wing base, basicosta black to dark brown with slight accent of orange along caudal edge; both upper and lower calypters also infuscate concolorous with remainder of wing; wing vein R_4+5_ setose, bearing only 2–3 setulae at base; halteres orange stalk with dark black/brown capitulum. **Legs**: black overall, lightly covered in shimmering silver tomentum, coxa on midleg and hindleg covered in black setulae; tarsal claws yellow-orange with black tips, with orange pulvilli subequal to length of tarsal claws; anterodorsal row of setae on hind tibia irregularly sized not fringelike, with 3–4 longer stronger setae at least 2X as long as others. **Abdomen**: large, flattened globose, with orange ground color, bisected dorsomedially by an area of darker brown almost black ground color; tomentum absent from T1+2 and T3 with only very slight bronzy tomentum along anterior margin, gold tomentum covering anterior 60% of surface of T4 , bisected medially by an area devoid of tomentum, densely gold tomentose throughout T5 not reaching to hind margin of tergite, black along caudal 10% of tergite, where it is devoid of gold; ventral surfaces of T3–T5 with no distinct sex-patches present; middorsal depression on ST1+2 reaching to hind margin of tergite; one pair of median marginal setae present on ST1+2 and T3, and complete rows of setae on T4 and T5; T5 devoid of any setulae in the area of gold tomentosity.

**Male terminalia** (Fig. 67): sternite 5 with a deeply excavated median cleft along posterior edge, smoothly Y-shaped, margins covered in dense tomentum; posterior lobes rounded apically, with multiple strong setulae surrounded by many shorter weaker setulae. Anterior plate of sternite 5, 3/4ths length of posterior lobes; unsclerotized "window" on anterior plate of sternite 5 vaguely translucent directly basal to posterior lobes, ovoid rectangular. Cerci in posterior view triangular, slightly longer than surstyli; rounded at apex, medially to fused along posterior 2/3 of their length. Cerci when viewed laterally, narrow apically widening basally giving it a subtriangular shape, apically displaying an elongate indentation occupying 1/3 of length of cercus, inferior edge beyond indentation with a slight convexity. Surstylus in lateral view, subequal in length to cercus, narrow basally, widening to a broad spatulate shape, apically rounded with more curvature along upper edge; surstylus appearing to be separate and not fused with epandrium; when viewed posteriorly slightly convergent. Pregonite broad, well-developed, apically squared blunt, with 2–3 marginal setulae. Postgonite, narrowed, 1/3 as wide as pregonite, rounded and blunt at apex, subequal in length to pregonite. Distiphallus bean shaped, with a slender median longitudinal sclerotized reinforcement on its posterior surface and a broad, anterolateral, sclerotized acrophallus, on anterior surface near apex, 2X as long as basiphallus.

**Female (Fig. 68)** length: 11–14mm, overall morphology as in male differing in the following traits: **Head**: fronto-orbital plate uniformly silver gray with darkened area much larger and shinier, bearing 3–4 pairs of proclinate orbital setae in addition to 1–2 pairs of reclinate orbital seta; profile of head not rounded as in males; vertex 1/3 of head width; palps slightly more pointed than males; gena 1/4 head height and 2/5 eye height. **Thorax**: Thoracic chaetotaxy, and tomentum as in males; setulae of anepimeron reddish yellow contrasting males black setulae. **Abdomen**: more globose than males, lacking the flattened character, setulae on abdomen not as dense appearing far less hirsute than male abdomen; differing in terminalia, and with very slight gold tomentum along anterior margin of T3.

#### Diagnosis

*Belvosiakeinoraragoni* **sp. n.** can be distinguished from all other *Belvosia* by the following combination of traits: fronto-orbital plate silver with slight gold tonality, T3 with silver tomentum extending to underside of tergite, pilosity of gena, anepisternum, katepisternum black, basicosta black, with no apparent sex patch on T3–T5. Interim species-specific name included in previously circulating databases and publications, *Belvosia* Woodley09.

#### Etymology

*Belvosiakeinoraragoni* **sp. n**, is named in honor of Sr. Keinor Aragon in recognition of his decades of being part of the Parataxonomist Program of Area de Conservación Guanacaste (http://www.acguanacaste.ac.cr) in northwestern Costa Rica (Janzen and Hallwachs 2011).

#### Distribution

Costa Rica, ACG, Guanacaste Province, 155–470m elevation.

#### Ecology

*Belvosiakeinoraragoni* **sp. n.** has been reared 76 times from two species of Lepidoptera in the family Saturniidae, *Eaclesimperialis*DHJ01 (N=1), Eacles imperialisDHJ02 (N=75), in rain forest, dry forest, and dry-rain lowland intergrade.

### Belvosia luciariosae

Fleming & Woodley sp. nov.

6C51C294-7EAE-59F0-AFD4-8F37742FC581

9142170E-25DA-4C28-8A4B-36E746204B3F

#### Materials

- **Type status:** Holotype. **Occurrence:** occurrenceDetails: http://janzen.sas.upenn.edu; catalogNumber: DHJPAR0001205; recordedBy: D.H. Janzen, W. Hallwachs & Guillermo Pereira; individualID: DHJPAR0001205; individualCount: 1; sex: Male; lifeStage: adult; preparations: pinned; otherCatalogNumbers: HCIC128-05, 02-SRNP-12718, BOLD:AAB4351; occurrenceID: B376B0C9-1395-5DB9-8F14-E54D148C575B; **Taxon:** scientificName: Belvosialuciariosae; phylum: Arthropoda; class: Insecta; order: Diptera; family: Tachinidae; genus: Belvosia; specificEpithet: luciariosae; scientificNameAuthorship: Fleming & Woodley, 2023; **Location:** continent: Central America; country: Costa Rica; countryCode: CR; stateProvince: Guanacaste; county: Sector Santa Rosa; locality: Area de Conservacion Guanacaste; verbatimLocality: Tanquetas; verbatimElevation: 295; verbatimLatitude: 10.8708; verbatimLongitude: -85.6053; verbatimCoordinateSystem: Decimal; decimalLatitude: 10.8708; decimalLongitude: -85.6053; **Identification:** identifiedBy: AJ Fleming; dateIdentified: 2022; **Event:** samplingProtocol: Reared from the larvae of the Saturniidae, Citheronialobesis; verbatimEventDate: 15-Aug-2002; **Record Level:** language: en; institutionCode: CNC; collectionCode: Insects; basisOfRecord: Pinned Specimen
- **Type status:** Paratype. **Occurrence:** occurrenceDetails: http://janzen.sas.upenn.edu; catalogNumber: DHJPAR0001898; recordedBy: D.H. Janzen, W. Hallwachs & gusaneros; individualID: DHJPAR0001898; individualCount: 1; sex: Female; lifeStage: adult; preparations: pinned; otherCatalogNumbers: HCIC414-05, 93-SRNP-2944,; occurrenceID: 65F54A7A-58B6-5F08-919A-7A3226A15B7A; **Taxon:** scientificName: Belvosialuciariosae; phylum: Arthropoda; class: Insecta; order: Diptera; family: Tachinidae; genus: Belvosia; specificEpithet: luciariosae; scientificNameAuthorship: Fleming & Woodley, 2023; **Location:** continent: Central America; country: Costa Rica; countryCode: CR; stateProvince: Guanacaste; county: Sector Santa Rosa; locality: Area de Conservacion Guanacaste; verbatimLocality: Bosque Encino Guacimal; verbatimElevation: 285; verbatimLatitude: 10.8688; verbatimLongitude: -85.6023; verbatimCoordinateSystem: Decimal; decimalLatitude: 10.8688; decimalLongitude: -85.6023; **Identification:** identifiedBy: AJ Fleming; dateIdentified: 2022; **Event:** samplingProtocol: Reared from the larvae of the Saturniidae, Citheronialobesis; verbatimEventDate: 05-Aug-1993; **Record Level:** language: en; institutionCode: CNC; collectionCode: Insects; basisOfRecord: Pinned Specimen
- **Type status:** Paratype. **Occurrence:** occurrenceDetails: http://janzen.sas.upenn.edu; catalogNumber: DHJPAR0001910; recordedBy: D.H. Janzen, W. Hallwachs & gusaneros; individualID: DHJPAR0001910; individualCount: 1; sex: Male; lifeStage: adult; preparations: pinned; otherCatalogNumbers: HCIC426-05, 91-SRNP-877.1,; occurrenceID: AB0FDFC6-2264-523B-A257-0370B311A9AC; **Taxon:** scientificName: Belvosialuciariosae; phylum: Arthropoda; class: Insecta; order: Diptera; family: Tachinidae; genus: Belvosia; specificEpithet: luciariosae; scientificNameAuthorship: Fleming & Woodley, 2023; **Location:** continent: Central America; country: Costa Rica; countryCode: CR; stateProvince: Guanacaste; county: Sector Santa Rosa; locality: Area de Conservacion Guanacaste; verbatimLocality: Area Administrativa; verbatimElevation: 295; verbatimLatitude: 10.8376; verbatimLongitude: -85.6187; verbatimCoordinateSystem: Decimal; decimalLatitude: 10.8376; decimalLongitude: -85.6187; **Identification:** identifiedBy: AJ Fleming; dateIdentified: 2022; **Event:** samplingProtocol: Reared from the larvae of the Saturniidae, Citheronialobesis; verbatimEventDate: 20-Jul-1991; **Record Level:** language: en; institutionCode: CNC; collectionCode: Insects; basisOfRecord: Pinned Specimen

#### Description

**Male** (Fig. 69), length: 11–13mm. **Head**: head wider than thorax; vertex 1/3 head width; gena 1/5 of head height, 1/3 of eye height. Fronto-orbital plate silver with no gold tomentum, darkening slightly apically in some cases appearing glabrous or devoid of tomentum apically, with two rows of frontal setae, populated with short black hair-like setulae intermingled with setae, with a few dark colored setulae extending below lowest frontal seta; ocellar setae weak and slightly lateroclinate, ranging from hair-like to distinctly present, adjacent to anterior ocellus; orbital setae absent. Parafacial light yellow in ground color, densely covered in silver tomentum, entire surface reflective and brilliant appearance; almost bare along parafacial outside facial ridge, with only a small number of setulae extending just below lowest frontal setae; facial ridge setose along 2/3 of its length, with few black hair-like setulae emerging along outer edge of row; gena covered in black setulae. Antenna, pedicel black with orange base, overall concolorous with postpedicel; postpedicel black, 3–4X as long as pedicel; arista bare gradually tapering to a point at tip. Palps, orange throughout and densely covered in short black setulae; tapering to a slight point apically, devoid of setulae apically. Vibrissa approximately 1–2 pedicel lengths from facial margin. **Thorax**: black ground color throughout, except around post-alar callus where it is lighter brown, with light gray tomentum throughout; scutellum ground color light brown almost yellow, distinctly lighter than scutum, under microscope bronze tomentum throughout becomes visible; scutum with four dorsal vittae, one outer pair, one inner pair, both broken at suture; lateral surface of thorax densely covered in long hair-like setulae, these setulae all black; chaetotaxy: 4–6 strong setae on postpronotum arranged in a line, acrostichal setae 3–4:3–4; dorsocentral setae 3–4:4; intra-alar setae 2–3:3; supra-alar setae 2–3:3; 4–6 katepisternal setae; scutellum, with 4–5 pairs of long marginal setae of subequal length; apical scutellar setae absent; 1 complete row of scutellar discal setae just posterior to marginal setae. **Wing**: infuscate, slightly darkened orange at wing base, basicosta black to dark brown with slight accent of orange along caudal edge; both upper and lower calypters also infuscate concolorous with remainder of wing; wing vein R_4+5_ setose, bearing only 2–3 setulae at base; halteres orange stalk with dark black/brown capitulum. **Legs**: black overall, lightly covered in shimmering silver tomentum, coxa on midleg and hindleg covered in black setulae; tarsal claws yellow-orange with black tips, with orange pulvilli subequal to length of tarsal claws; anterodorsal row of setae on hind tibia irregularly sized not fringelike, with 3–4 longer stronger setae at least 2X as long as others. **Abdomen**: large, flattened globose, with orange ground color, bisected dorsomedially by an area of darker brown almost black ground color; tomentum absent from T1+2 and T3 with only very slight gold tomentum along anterior margin, gold tomentum covering anterior 70-80% of surface of T4 , bisected medially by an area devoid of tomentum, densely gold tomentose throughout T5 not reaching to hind margin of tergite, black along caudal 10% of tergite, where it is devoid of gold; entire surface of T3 uniformly lightly brown rusty tomentose including underside (apparent under certain angles of light); ventral surfaces of T3–T5 with no distinct sex-patches present; middorsal depression on ST1+2 reaching to hind margin of tergite; one pair of median marginal setae present on ST1+2 and T3, and complete rows of setae on T4 and T5; T5 devoid of any setulae in the area of gold tomentosity.

**Male terminalia** (Fig. 70): sternite 5 with a deeply excavated median cleft along posterior edge, smoothly Y-shaped, margins covered in dense tomentum; posterior lobes rounded apically, with multiple strong stout setae surrounded by finer hair-like setulae. Anterior plate of sternite 5 subequal to length of posterior lobes; unsclerotized "window" on anterior plate of sternite 5 almost entirely transparent directly basal to posterior lobes, vaguely rectangular in shape with slightly upturned corners. Cerci in posterior view, wide based triangular, only slightly longer than surstyli, almost equal in length; blunt and rounded at apex, medially fused along 1/2 their length. Cerci in lateral view, often straight along 90% of their length with a strong anterior curve on apex, giving it a clubbed appearance; cerci densely setose along basal 2/3rds. Surstylus in lateral view, almost equilateral along its length making the structure appear digitiform; surstylus appearing to be separate and not fused with epandrium; when viewed posteriorly surstyli slightly divergent, curving outwards at their apices. Pregonite usually broad, slightly elongate and well-developed, apically rounded, with a few marginal setulae. Postgonite, slightly narrowed, 1/3 as wide as pregonite, short and arced. Distiphallus broadly cone-shaped appearing somewhat square or barrel shaped, with a slender median longitudinal sclerotized reinforcement on its posterior surface and a broad, anterolateral, sclerotized acrophallus, on anterior surface near apex, ~2.2X as long as basiphallus.

**Female** (Fig. 71) length: 12–13mm, overall morphology as in male differing in the following traits: **Head**: fronto-orbital plate uniformly silver gray with darkened area much larger and shinier, bearing 3–4 pairs of proclinate orbital setae in addition to 1–2 pairs of reclinate orbital seta; profile of head not rounded as in males; vertex 1/3 of head width; palps slightly more pointed than males; gena 1/4 head height and 1/3 eye height. **Thorax**: Thoracic chaetotaxy, and tomentum as in males; setulae of anepimeron black. **Abdomen**: more globose than males, lacking the flattened character, setulae on abdomen not as dense appearing far less hirsute than male abdomen; differing in terminalia, and with very slight gold tomentum along anterior margin of T3.

#### Diagnosis

*Belvosialuciariosae* **sp. n.** can be distinguished from all other *Belvosia* by the following combination of traits: fronto-orbital plate silver with no gold tonality, T3 entirely rusty gold tomentose, pilosity of gena, anepisternum, katepisternum black, basicosta black, with no apparent sex patch on T3–T5.

#### Etymology

*Belvosialuciariosae* **sp. n**, is named in honor of Sra. Lucia Rios in recognition of her decades of being part of the Parataxonomist Program of Area de Conservación Guanacaste (http://www.acguanacaste.ac.cr) in northwestern Costa Rica (Janzen and Hallwachs 2011). Interim species-specific name included in previously circulating databases and publications, *Belvosia* Woodley10.

#### Distribution

Costa Rica, ACG, Alajuela and Guanacaste Provinces, 160–645m elevation.

#### Ecology

*Belvosialuciariosae* **sp. n.** has been reared 52 times from two species of Lepidoptera in the family Saturniidae, *Citheroniabellavista* Draudt, 1830 (N=1), *Citheronialobesis* Rothschild, 1907 (N=51), in rain forest, dry forest, and dry-rain lowland intergrade.

### Belvosia manuelpereirai

Fleming & Woodley sp. nov.

5A53CF56-BC2F-5E75-A8BE-DF5695271884

4C6F73D9-63C1-4EE6-BAE8-364029AA47AD

#### Materials

- **Type status:** Holotype. **Occurrence:** occurrenceDetails: http://janzen.sas.upenn.edu; catalogNumber: DHJPAR0001217; recordedBy: D.H. Janzen, W. Hallwachs & gusaneros; individualID: DHJPAR0001217; individualCount: 1; sex: Male; lifeStage: adult; preparations: pinned; otherCatalogNumbers: HCIC135-05, 02-SRNP-12585, BOLD:AAC9692; occurrenceID: B1476E59-B563-5126-BEA8-B613CB6F1A3F; **Taxon:** scientificName: Belvosiamanuelpereirai; phylum: Arthropoda; class: Insecta; order: Diptera; family: Tachinidae; genus: Belvosia; specificEpithet: manuelpereirai; scientificNameAuthorship: Fleming & Woodley, 2023; **Location:** continent: Central America; country: Costa Rica; countryCode: CR; stateProvince: Guanacaste; county: Sector Santa Elena; locality: Area de Conservacion Guanacaste; verbatimLocality: Vado Quebrada Calera; verbatimElevation: 305; verbatimLatitude: 10.8668; verbatimLongitude: -85.6465; verbatimCoordinateSystem: Decimal; decimalLatitude: 10.8668; decimalLongitude: -85.6465; **Identification:** identifiedBy: AJ Fleming; dateIdentified: 2022; **Event:** samplingProtocol: Reared from the larvae of the Notodontidae, Dasylophia placida; verbatimEventDate: 25-Jul-2002; **Record Level:** language: en; institutionCode: CNC; collectionCode: Insects; basisOfRecord: Pinned Specimen
- **Type status:** Paratype. **Occurrence:** occurrenceDetails: http://janzen.sas.upenn.edu; catalogNumber: DHJPAR0001211; recordedBy: D.H. Janzen, W. Hallwachs & gusaneros; individualID: DHJPAR0001211; individualCount: 1; sex: Female; lifeStage: adult; preparations: pinned; otherCatalogNumbers: HCIC176-05, 02-SRNP-12561,; occurrenceID: ECA8FC73-6BD2-5960-AFFF-86177E090602; **Taxon:** scientificName: Belvosiamanuelpereirai; phylum: Arthropoda; class: Insecta; order: Diptera; family: Tachinidae; genus: Belvosia; specificEpithet: manuelpereirai; scientificNameAuthorship: Fleming & Woodley, 2023; **Location:** continent: Central America; country: Costa Rica; countryCode: CR; stateProvince: Guanacaste; county: Sector Santa Elena; locality: Area de Conservacion Guanacaste; verbatimLocality: Vado Quebrada Calera; verbatimElevation: 305; verbatimLatitude: 10.8668; verbatimLongitude: -85.6465; verbatimCoordinateSystem: Decimal; decimalLatitude: 10.8668; decimalLongitude: -85.6465; **Identification:** identifiedBy: AJ Fleming; dateIdentified: 2022; **Event:** samplingProtocol: Reared from the larvae of the Notodontidae, Dasylophia placida; verbatimEventDate: 06-Aug-2002; **Record Level:** language: en; institutionCode: CNC; collectionCode: Insects; basisOfRecord: Pinned Specimen
- **Type status:** Paratype. **Occurrence:** occurrenceDetails: http://janzen.sas.upenn.edu; catalogNumber: DHJPAR0016472; recordedBy: D.H. Janzen, W. Hallwachs & Jose Cortez; individualID: DHJPAR0016472; individualCount: 1; sex: Male; lifeStage: adult; preparations: pinned; otherCatalogNumbers: ASTAP676-07, 06-SRNP-60380, BOLD:AAC9692; occurrenceID: 43585C47-B3D2-5CBB-B306-82B44FAE587C; **Taxon:** scientificName: Belvosiamanuelpereirai; phylum: Arthropoda; class: Insecta; order: Diptera; family: Tachinidae; genus: Belvosia; specificEpithet: manuelpereirai; scientificNameAuthorship: Fleming & Woodley, 2023; **Location:** continent: Central America; country: Costa Rica; countryCode: CR; stateProvince: Guanacaste; county: Sector Mundo Nuevo; locality: Area de Conservacion Guanacaste; verbatimLocality: Quebrada Tibio Perla; verbatimElevation: 330; verbatimLatitude: 10.7626; verbatimLongitude: -85.4298; verbatimCoordinateSystem: Decimal; decimalLatitude: 10.7626; decimalLongitude: -85.4298; **Identification:** identifiedBy: AJ Fleming; dateIdentified: 2022; **Event:** samplingProtocol: Reared from the larvae of the Notodontidae, Xylodontaguarana; verbatimEventDate: 17-Jan-2007; **Record Level:** language: en; institutionCode: CNC; collectionCode: Insects; basisOfRecord: Pinned Specimen

#### Description

**Male** (Fig. 72), length: 9–12mm. **Head**: head wider than thorax; vertex 1/3 head width; gena 1/4 of head height, 1/3 of eye height. Fronto-orbital plate dull gray with a silver sheen and with no gold tomentum, darkening slightly apically in some cases appearing glabrous or devoid of tomentum apically, with 2–3 irregular rows of frontal setae, populated with short black hair-like setulae intermingled with setae; ocellar setae absent; orbital setae absent. Parafacial light yellow in ground color, densely covered in silver tomentum, entire surface reflective and brilliant appearance; almost bare along parafacial outside facial ridge, with only 1–2 setulae extending just below lowest frontal setae; facial ridge setose along 2/3 of its length; gena covered in black setulae. Antenna, pedicel darkened orange sometimes appearing dark brown or black, overall concolorous with postpedicel; postpedicel dark brown with orange accent, 3–4X as long as pedicel; arista bare gradually tapering to a point at tip. Palps, orange throughout and densely covered in short black setulae; tapering to a sharp point apically, devoid of setulae apically. Vibrissa approximately 1 pedicel length from facial margin. **Thorax**: black ground color throughout, with light gray tomentum throughout, except around post-alar callus where it is lighter brown and bronze tomentose; scutellum ground color light brown almost yellow, distinctly lighter than scutum, under microscope bronze tomentum throughout becomes visible; scutum with four dorsal vittae, one outer pair, one inner pair, both broken at suture; lateral surface of thorax densely covered in long hair-like setulae, these setulae all black; chaetotaxy: 3–4 strong setae (4 setae on N=1) on postpronotum arranged in a line, acrostichal setae 3:3–5; dorsocentral setae 3:4; intra-alar setae 3:3; supra-alar setae 2:3; 4 katepisternal setae; scutellum, with 4–5 pairs of long marginal setae of subequal length; apical scutellar setae short erect, inserted slightly above plane of marginal setae; 1 complete row of scutellar discal setae just posterior to marginal setae. **Wing**, infuscate, slightly darkened gray at wing base, basicosta brilliant orange; both upper and lower calypters also infuscate concolorous with remainder of wing; wing vein R_4+5_ setose, bearing only 2–3 setulae at base; halteres orange stalk with dark black/brown capitulum. **Legs**: black overall, lightly covered in shimmering silver tomentum, coxa on midleg and hindleg covered in black setulae; tarsal claws yellow-orange with black tips, with burnt umber pulvilli shorter than length of tarsal claws; anterodorsal row of setae on hind tibia regularly sized fringelike, with 1 longer stronger setae at least 2X as long as others. **Abdomen**: medium (compared to other congeneric species), rounded globose, black ground color; tomentum absent from T1+2, light dusting of bronze tomentum on T3 with only very slight gold tomentum along anterior margin, gold tomentum covering anterior 70-80% of surface of T4 , bisected medially by an area devoid of tomentum, densely gold tomentose throughout T5 not reaching to hind margin of tergite; ventral surfaces of T3–T5 with no distinct sex-patches present, but with light gold tomentum throughout; middorsal depression on ST1+2 reaching to hind margin of tergite; ST1+2 with no median marginal setae, one pair of median marginal setae present on T3, and complete rows of setae on T4 and T5.

**Male terminalia** (Fig. 73) : sternite 5 with an excavated median cleft along posterior edge, smoothly U-shaped, margins covered in dense tomentum; posterior lobes squared off apically, with 3–5 strong erect bristle-like setulae surrounded by many shorter weaker setulae. Anterior plate of sternite 5 subequal to length of posterior lobes; unsclerotized "window" on anterior plate of sternite 5 translucent directly basal to posterior lobes, flat basally, with 2 indentations along anterior edge, like a flattened "w". Cerci in posterior view triangular, short subequal to length of surstyli; separate medially along apical 2/3s of its length, appearing serrate along interior margins. Cerci in lateral view, narrow and appearing rounded apically, straight along lower margin with only a very slight anterior projection, not appearing clubbed apically; cerci setose along basal 2/3rds, underside of cerci bare. Surstylus in lateral view, wide broadly rounded, spatulate or oarlike appearance; surstylus appearing fused with epandrium; when viewed posteriorly surstyli appearing slightly convergent or bearing inward curved apices but not strongly convergent. Pregonite short, not well-developed, apically flat, somewhat blunt, devoid of setulae. Postgonite, short slightly narrowed, 1/3 as wide and 2/3rds as long as pregonite, rounded and blunt at apex. Distiphallus broadly cone-shaped and a broad, anterolateral, sclerotized acrophallus, on anterior surface near apex, 1.5X as long as basiphallus.

**Female** (Fig. 74) length: 10–12mm, overall morphology as in male differing in the following traits: **Head**: bearing three pairs of proclinate orbital setae in addition to single pair of reclinate orbital seta. **Abdomen**: gold tomentum along anterior 80% of surface of T4 and all of T5, much denser than in males; T4 bearing a narrow median black stripe bisecting yellow band; slightly more globose than males.

#### Diagnosis

*Belvosiamanuelpereirai* **sp. n.** can be distinguished from all other *Belvosia* by the following combination of traits: dorsal surfaces of scutum entirely silver tomentose, orange basicosta, pedicel brown concolorous with postpedicel, and median marginal setae absent from ST1+2.

#### Etymology

*Belvosiamanuelpereirai* **sp. n**, is named in honor of Sr. Manuel Pereira in recognition of his decades of being part of the Parataxonomist Program of Area de Conservación Guanacaste (http://www.acguanacaste.ac.cr) in northwestern Costa Rica (Janzen and Hallwachs 2011). Interim species-specific name included in previously circulating databases and publications, *Belvosia* Woodley11.

#### Distribution

Costa Rica, ACG, Guanacaste Province, 160–330 m elevation.

#### Ecology

*Belvosiamanuelpereirai* **sp. n.** has been reared 19 times from four species of Lepidoptera in the family Notodontidae, *Nycterotisplacida* (Schaus, 1892) (N=11), *Nycterotisravana*ICG02 (N=4), *Nycterotisxylinoides*DHJ02 (N=2), and *Xylodontaguarana* (Schaus, 1892) (N=2), in dry forest, dry-rain lowland intergrade.

### Belvosia manuelriosi

Fleming & Woodley sp. nov.

AAFA1878-3DA8-556C-B0FB-67917ED9D228

DE1EEEF6-4995-40D5-9857-308030F92488

#### Materials

- **Type status:** Holotype. **Occurrence:** occurrenceDetails: http://janzen.sas.upenn.edu; catalogNumber: DHJPAR0001246; recordedBy: D.H. Janzen, W. Hallwachs & gusaneros; individualID: DHJPAR0001246; individualCount: 1; lifeStage: adult; preparations: pinned; otherCatalogNumbers: HCIC181-05, 92-SRNP-2997, BOLD:ACE4203; occurrenceID: F56C4200-10F0-5C55-A93D-897554B2B0DD; **Taxon:** scientificName: Belvosiamanuelriosi; phylum: Arthropoda; class: Insecta; order: Diptera; family: Tachinidae; genus: Belvosia; specificEpithet: manuelriosi; scientificNameAuthorship: Fleming & Woodley, 2023; **Location:** continent: Central America; country: Costa Rica; countryCode: CR; stateProvince: Guanacaste; county: Sector Santa Rosa; locality: Area de Conservacion Guanacaste; verbatimLocality: Vado Cuajiniquil; verbatimElevation: 275; verbatimLatitude: 10.9404; verbatimLongitude: -85.6804; verbatimCoordinateSystem: Decimal; decimalLatitude: 10.9404; decimalLongitude: -85.6804; **Identification:** identifiedBy: AJ Fleming; dateIdentified: 2022; **Event:** samplingProtocol: Reared from the larvae of the Noctuidae, Diopa furculaDHJ02; verbatimEventDate: 07-Aug-1992; **Record Level:** language: en; institutionCode: CNC; collectionCode: Insects; basisOfRecord: Pinned Specimen
- **Type status:** Paratype. **Occurrence:** occurrenceDetails: http://janzen.sas.upenn.edu; catalogNumber: DHJPAR0001247; recordedBy: D.H. Janzen, W. Hallwachs; individualID: DHJPAR0001247; individualCount: 1; lifeStage: adult; preparations: pinned; otherCatalogNumbers: HCIC189-05, CR1000-344397,; occurrenceID: 3120394C-1DF4-5822-B89A-C015F4F299DE; **Taxon:** scientificName: Belvosiamanuelriosi; phylum: Arthropoda; class: Insecta; order: Diptera; family: Tachinidae; genus: Belvosia; specificEpithet: manuelriosi; scientificNameAuthorship: Fleming & Woodley, 2023; **Location:** continent: Central America; country: Costa Rica; countryCode: CR; stateProvince: Guanacaste; locality: Area de Conservacion Guanacaste; verbatimCoordinateSystem: Decimal; **Identification:** identifiedBy: AJ Fleming; dateIdentified: 2022; **Event:** samplingProtocol: unknown; **Record Level:** language: en; institutionCode: CNC; collectionCode: Insects; basisOfRecord: Pinned Specimen

#### Description

**Male** (Fig. 75), length: 11mm. **Head**: head wider than thorax; vertex 1/4 head width; gena 1/5 of head height, 1/4 of eye height. Fronto-orbital plate brilliant gold, dark brown at vertex and along posterior edge of eyes but returning to gold posterior to ocellar triangle, with 2 irregular rows of frontal setae, populated with short black hair-like setulae intermingled with setae; ocellar setae absent; two pairs of proclinate orbital setae present, along with one pair of posterior reclinate orbital setae. Parafacial light yellow in ground color, densely covered in gold tomentum, entire surface reflective and brilliant appearance; almost bare along parafacial outside facial ridge, with only 2–4 setulae extending just below lowest frontal setae; facial ridge setose along 4/5 of its length; gena covered in black setulae. Antenna, pedicel orange, contrasting postpedicel; postpedicel dark brown with orange accent, 4X as long as pedicel; arista bare gradually tapering to a point at tip. Palps, orange throughout and densely covered in short black setulae; tapering to a sharp point apically, somewhat oar-like devoid of setulae apically. Vibrissa approximately 3 pedicel lengths from facial margin. **Thorax**: black ground color throughout, with light grayish gold tomentum throughout, except around post-alar callus where it is lighter gray tomentose; scutellum ground color light brown almost yellow, distinctly lighter than scutum, gray tomentose; scutum with four thick pronounced dorsal vittae, one outer pair, one inner pair, unbroken across suture; lateral surface of thorax densely covered in long hair-like setulae, these setulae all black; chaetotaxy: 3 strong setae on postpronotum arranged in a line, acrostichal setae 3:4; dorsocentral setae 3:4; intra-alar setae 3:3; supra-alar setae 2:3; 4 katepisternal setae; scutellum, with 4–5 pairs of long marginal setae of subequal length; apical scutellar setae absent; one complete row of scutellar discal setae just posterior to marginal setae, approximately 1/2 length of scutellar marginal setae. **Wing**: pale infuscate, slightly darkened gray at wing base, basicosta brilliant orange; both upper and lower calypters white translucent, blushing to infsucate brown along central portion gradually transitioning to pale white along margins; wing vein R_4+5_ setose, bearing only 2–3 setulae at base; halteres orange stalk with dark black/brown capitulum. **Legs**: black overall, lightly covered in shimmering silver tomentum, coxa on midleg and hindleg covered in black setulae; tarsal claws yellow-orange with black tips, with burnt umber pulvilli shorter than length of tarsal claws; anterodorsal row of setae on hind tibia not regularly sized or fringelike, with several longer stronger setae at least 2X as long as others. **Abdomen**: medium (compared to other congeneric species), rounded globose, black ground color; silver tomentum present on posterior 50% of T1+2, T3 with only a solid covering of silver-gold tometum throughout, pale gold tomentum covering posterior 90% of surface of T4, densely gold tomentose throughout T5; ventral surfaces of T3–T5 with no distinct sex-patches present, but with light gold tomentum throughout; middorsal depression on ST1+2 reaching to hind margin of tergite; ST1+2 with no median marginal setae, one pair of median marginal setae present on T3, and complete rows of setae on T4 and T5.

**Female**: unknown at this time.

#### Diagnosis

*Belvosiamanuelriosi* **sp. n.** can be distinguished from all other *Belvosia* by the following combination of traits: fronto-orbital plate brilliant gold, males with proclinate orbital setae, pilosity of gena, anepisternum, katepisternum black, basicosta brilliant orange, abdomen with dark ground color, and median marginal setae absent from syntergite 1+2.

#### Etymology

*Belvosiamanuelriosi* **sp. n**, is named in honor of Sr. Manuel Rios in recognition of his decades of being part of the Parataxonomist Program of Area de Conservación Guanacaste (http://www.acguanacaste.ac.cr) in northwestern Costa Rica (Janzen and Hallwachs 2011). Interim species-specific name included in previously circulating databases and publications, *Belvosia* Woodley12.

#### Distribution

Costa Rica, ACG, Guanacaste Province, 275–305m elevation.

#### Ecology

*Belvosiamanuelriosi* **sp. n.** has been reared two times from two species of Lepidoptera in the family Notodontidae, *Diopafurcula*DHJ02 (N=1), *Nycterotisplacida* (Schaus, 1892), in dry forest.

### Belvosia minorcarmonai

Fleming & Woodley sp. nov.

0F9DDBE8-4ABD-5DF7-B6B9-7F3058C2A9FE

CEF8FF8F-688F-4958-B695-8386D851CC29

#### Materials

- **Type status:** Holotype. **Occurrence:** occurrenceDetails: http://janzen.sas.upenn.edu; catalogNumber: DHJPAR0001240; recordedBy: D.H. Janzen, W. Hallwachs & Mariano Pereira; individualID: DHJPAR0001240; individualCount: 1; sex: Male; lifeStage: adult; preparations: pinned; otherCatalogNumbers: HCIC133-05, 00-SRNP-9033, BOLD:AAG2421; occurrenceID: DC307283-439C-5E74-BD5E-C5830322521A; **Taxon:** scientificName: Belvosiaminorcarmonai; phylum: Arthropoda; class: Insecta; order: Diptera; family: Tachinidae; genus: Belvosia; specificEpithet: minorcarmonai; scientificNameAuthorship: Fleming & Woodley, 2023; **Location:** continent: Central America; country: Costa Rica; countryCode: CR; stateProvince: Guanacaste; county: Sector Cacao; locality: Area de Conservacion Guanacaste; verbatimLocality: Sendero Cima; verbatimElevation: 1460; verbatimLatitude: 10.9333; verbatimLongitude: -85.4573; verbatimCoordinateSystem: Decimal; decimalLatitude: 10.9333; decimalLongitude: -85.4573; **Identification:** identifiedBy: AJ Fleming; dateIdentified: 2022; **Event:** samplingProtocol: Reared from the larvae of the Eupterotidae, Neopreptosmarathusa; verbatimEventDate: 18-Jun-2000; **Record Level:** language: en; institutionCode: CNC; collectionCode: Insects; basisOfRecord: Pinned Specimen
- **Type status:** Paratype. **Occurrence:** occurrenceDetails: http://janzen.sas.upenn.edu; catalogNumber: DHJPAR0001241; recordedBy: D.H. Janzen, W. Hallwachs & Mariano Pereira; individualID: DHJPAR0001241; individualCount: 1; sex: Male; lifeStage: adult; preparations: pinned; otherCatalogNumbers: HCIC141-05, 01-SRNP-6397, BOLD:AAG2421; occurrenceID: F5A0DC20-58FF-5B18-AB45-0D9A5BF333AB; **Taxon:** scientificName: Belvosiaminorcarmonai; phylum: Arthropoda; class: Insecta; order: Diptera; family: Tachinidae; genus: Belvosia; specificEpithet: minorcarmonai; scientificNameAuthorship: Fleming & Woodley, 2023; **Location:** continent: Central America; country: Costa Rica; countryCode: CR; stateProvince: Guanacaste; county: Sector Cacao; locality: Area de Conservacion Guanacaste; verbatimLocality: Sendero Cima; verbatimElevation: 1460; verbatimLatitude: 10.9333; verbatimLongitude: -85.4573; verbatimCoordinateSystem: Decimal; decimalLatitude: 10.9333; decimalLongitude: -85.4573; **Identification:** identifiedBy: AJ Fleming; dateIdentified: 2022; **Event:** samplingProtocol: Reared from the larvae of the Eupterotidae, Neopreptosmarathusa; verbatimEventDate: 25-Jun-2001; **Record Level:** language: en; institutionCode: CNC; collectionCode: Insects; basisOfRecord: Pinned Specimen

#### Description

**Male** (Fig. 76), length: 12–13mm. **Head**: head wider than thorax; vertex 1/3 head width; gena 1/3 of head height, 1/2 of eye height. Fronto-orbital plate brassy gold tomentose throughout, darkening slightly apically in some cases appearing slightly glabrous apically, with 2–3 irregular rows of frontal setae, populated with short black hair-like setulae intermingled with setae; ocellar setae absent; 1 pair of slightly inwardly lateroclinate orbital setae present outside frontal row. Parafacial light yellow in ground color, densely covered in same brassy gold tomentum as on fronto-orbital plate, entire surface reflective and brilliant appearance; almost bare along parafacial outside facial ridge, with several black and reddish-yellow setulae intermingled with facial ridge setae and extending just below lowest frontal setae; facial ridge setose along 2/3 of its length; gena covered in yellow setulae. Antenna, pedicel darkened orange appearing dark brown or black, overall concolorous with postpedicel, covered in a brassy gold sheen; postpedicel dark brown almost black, 3–4X as long as pedicel; arista bare gradually tapering to a point at tip. Palps, orange throughout and densely covered in short black setulae; tapering to a sharp point apically, devoid of setulae apically. Vibrissa approximately 1 pedicel length from facial margin. **Thorax**: black ground color throughout, with brassy-gold tomentum throughout; scutellum ground color light brown almost yellow, distinctly lighter than scutum, under microscope bronze tomentum throughout becomes visible; scutum with five dorsal vittae, one outer pair, one inner pair, both broken at suture, and one dorsocentral vitta appearing postsuturally; lateral surface of thorax densely covered in long hair-like setulae, these setulae all reddish-yellow; chaetotaxy: 3 strong setae on postpronotum arranged in a line, acrostichal setae 3:4; dorsocentral setae 3:4; intra-alar setae 3:3; supra-alar setae 2:3; 4 katepisternal setae; scutellum, with 4–5 pairs of long marginal setae of subequal length; apical scutellar setae short erect, inserted slightly above plane of marginal setae; 1 complete row of scutellar discal setae just posterior to marginal setae. **Wing**: infuscate, slightly darkened yellow/orange at wing base, basicosta brilliant orange; both upper and lower calypters also infuscate concolorous with remainder of wing; wing vein R_4+5_ setose, bearing only 2–3 setulae at base; halteres orange stalk with dark black/brown capitulum. **Legs**: black overall, lightly covered in shimmering bronze tomentum, posterior margin of coxa on midleg and hindleg covered in yellow setulae; tarsal claws yellow-orange with black tips, with burnt umber pulvilli shorter than length of tarsal claws; anterodorsal row of setae on hind tibia irregular and not fringelike, with several longer stronger setae at least 2X as long as others. **Abdomen**: large and slightly flattened globose, black to dark burgundy ground color; tomentum absent from T1+2, light dusting of bronze tomentum on T3 with only very slight gold tomentum along anterior margin, dark bronze tomentum covering anterior 70-80% of surface of T4 , bisected medially by an area devoid of tomentum, subdued gold tomentose throughout T5 reaching to hind margin of tergite; ventral surfaces of T3–T5 extremely densely hirsute but with no distinct sex-patches present, with light gold tomentum throughout; middorsal depression on ST1+2 reaching to hind margin of tergite; ST1+2 with 3–4 pairs of median marginal setae, 3–4 pairs of median marginal setae present on T3, along with 3–4 pairs of lateral marginal setae, and complete rows of setae on T4 and T5.

**Male terminalia** (Fig. 77): sternite 5 with a deeply excavated wide median cleft along posterior edge, U-shaped, margins covered in dense tomentum; posterior lobes rounded apically, with a group of strong setulae surrounded by many shorter weaker setulae. Anterior plate of sternite 5 approximately 1/2 length of posterior lobes; unsclerotized "window" on anterior plate of sternite 5 elongate, translucent, rectangular, slight convex indentation at midline and slightly upturned at extremities. Cerci in posterior view triangular, equal to length of surstyli; pointed at apex, medially to fused along basal 1/2 of their length. Cerci in lateral view, inflated along basal 1/3rd, sharply tapered with a slight bend at apex, giving it a small nub; cerci setose along basal 2/3rds, underside of cerci setose along basal 2/3 of length. Surstylus in lateral view, pointed apically, leaf shaped slightly arcuate along inferior margin, and curved along superior margin; surstylus appearing not fused with epandrium; when viewed posteriorly surstyli straight not convergent. Pregonite broad, well-developed, apically rounded off, and blunt, devoid of setulae. Postgonite, narrow, 1/2 as wide as pregonite, blunt and round at apex, postgonite subequal in length to pregonite. Distiphallus broadly cone-shaped, with a slender median longitudinal sclerotized reinforcement on its posterior surface and a broad, sclerotized acrophallus, blunt and bulbous near apex, 1.5X length of basiphallus.

**Female**: unknown at this time.

#### Diagnosis

*Belvosiaminorcarmonai* **sp. n.** can be distinguished from all other *Belvosia* by the following combination of traits: yellow setulae below lowest frontal setae and gena, orange basicosta, ST1+2 with 2–4 pairs of median marginal setae, and complete rows of median marginal setae on T3–T5, and very light gold tomentum on T5.

#### Etymology

*Belvosiaminorcarmonai* **sp. n**, is named in honor of Sr. Minor Carmona in recognition of his decades of being part of the Parataxonomist Program of Area de Conservación Guanacaste (http://www.acguanacaste.ac.cr) in northwestern Costa Rica (Janzen and Hallwachs 2011). Interim species-specific name included in previously circulating databases and publications, *Belvosia* Woodley13.

#### Distribution

Costa Rica, ACG, Guanacaste Province, 1460m elevation.

#### Ecology

*Belvosiaminorcarmonai* **sp. n.** has been reared three times from one species of Lepidoptera in the family Eupterotidae, *Neopreptosmarathusa* (Druce, 1886) (N=3), in cloud forest.

### Belvosia osvaldoespinozai

Fleming & Woodley sp. nov.

6774E445-E1B1-5390-AFB9-DE931DB3AB5C

E3881A0D-CC2D-4A9E-8FF2-E733BDA1A20A

#### Materials

- **Type status:** Holotype. **Occurrence:** occurrenceDetails: http://janzen.sas.upenn.edu; catalogNumber: DHJPAR0001713; recordedBy: D.H. Janzen, W. Hallwachs & Mariano Pereira; individualID: DHJPAR0001713; individualCount: 1; sex: Male; lifeStage: adult; preparations: pinned; otherCatalogNumbers: HCIC231-05, 00-SRNP-9378, BOLD:AAB4355; occurrenceID: 88B03351-E58D-507E-9244-32FCFF91451A; **Taxon:** scientificName: Belvosiaosvaldoespinozai; phylum: Arthropoda; class: Insecta; order: Diptera; family: Tachinidae; genus: Belvosia; specificEpithet: osvaldoespinozai; scientificNameAuthorship: Fleming & Woodley, 2023; **Location:** continent: Central America; country: Costa Rica; countryCode: CR; stateProvince: Guanacaste; county: Sector Cacao; locality: Area de Conservacion Guanacaste; verbatimLocality: Sendero Salto; verbatimElevation: 1000; verbatimLatitude: 10.9302; verbatimLongitude: -85.4694; verbatimCoordinateSystem: Decimal; decimalLatitude: 10.9302; decimalLongitude: -85.4694; **Identification:** identifiedBy: AJ Fleming; dateIdentified: 2022; **Event:** samplingProtocol: Reared from the larvae of the Erebidae, Ochrodota marinaDHJ01; verbatimEventDate: 15-May-2000; **Record Level:** language: en; institutionCode: CNC; collectionCode: Insects; basisOfRecord: Pinned Specimen
- **Type status:** Paratype. **Occurrence:** occurrenceDetails: http://janzen.sas.upenn.edu; catalogNumber: DHJPAR0001714; recordedBy: D.H. Janzen, W. Hallwachs & Elda Araya; individualID: DHJPAR0001714; individualCount: 1; sex: Female; lifeStage: adult; preparations: pinned; otherCatalogNumbers: HCIC232-05, 04-SRNP-1331, BOLD:AAB4355; occurrenceID: ECFDE3BC-D8CB-53E8-AD88-A1136743B690; **Taxon:** scientificName: Belvosiaosvaldoespinozai; phylum: Arthropoda; class: Insecta; order: Diptera; family: Tachinidae; genus: Belvosia; specificEpithet: osvaldoespinozai; scientificNameAuthorship: Fleming & Woodley, 2023; **Location:** continent: Central America; country: Costa Rica; countryCode: CR; stateProvince: Alajuela; county: Sector San Cristobal; locality: Area de Conservacion Guanacaste; verbatimLocality: Puente Palma; verbatimElevation: 460; verbatimLatitude: 10.9163; verbatimLongitude: -85.3787; verbatimCoordinateSystem: Decimal; decimalLatitude: 10.9163; decimalLongitude: -85.3787; **Identification:** identifiedBy: AJ Fleming; dateIdentified: 2022; **Event:** samplingProtocol: Reared from the larvae of the Erebidae, Ochrodota pronapidesBE03; verbatimEventDate: 12-Apr-2004; **Record Level:** language: en; institutionCode: CNC; collectionCode: Insects; basisOfRecord: Pinned Specimen
- **Type status:** Paratype. **Occurrence:** occurrenceDetails: http://janzen.sas.upenn.edu; catalogNumber: DHJPAR0016466; recordedBy: D.H. Janzen, W. Hallwachs & Wilson Miranda Badilla; individualID: DHJPAR0016466; individualCount: 1; sex: Male; lifeStage: adult; preparations: pinned; otherCatalogNumbers: ASTAP670-07, 06-SRNP-65452, BOLD:AAB4355; occurrenceID: 2008F316-7FEE-5907-87D8-E7D8DE767C40; **Taxon:** scientificName: Belvosiaosvaldoespinozai; phylum: Arthropoda; class: Insecta; order: Diptera; family: Tachinidae; genus: Belvosia; specificEpithet: osvaldoespinozai; scientificNameAuthorship: Fleming & Woodley, 2023; **Location:** continent: Central America; country: Costa Rica; countryCode: CR; stateProvince: Guanacaste; county: Sector Pitilla; locality: Area de Conservacion Guanacaste; verbatimLocality: Sendero Trichoptera; verbatimElevation: 655; verbatimLatitude: 10.9857; verbatimLongitude: -85.4187; verbatimCoordinateSystem: Decimal; decimalLatitude: 10.9857; decimalLongitude: -85.4187; **Identification:** identifiedBy: AJ Fleming; dateIdentified: 2022; **Event:** samplingProtocol: Reared from the larvae of the Erebidae, Ochrodota marinaDHJ02; verbatimEventDate: 23-Dec-2006; **Record Level:** language: en; institutionCode: CNC; collectionCode: Insects; basisOfRecord: Pinned Specimen

#### Description

**Male** (Fig. 78), length: 9–11mm. **Head**: head wider than thorax; vertex 1/3 head width; gena 1/3 of head height, 2/5 of eye height. Fronto-orbital plate silver tomentose throughout, darkening to gray appearing glabrous apically, with 2–3 irregular rows of frontal setae, populated with short black hair-like setulae intermingled with setae; ocellar setae absent; 1 pair of slightly inwardly lateroclinate orbital setae present outside frontal row. Parafacial light yellow in ground color, densely covered in same silver tomentum as on fronto-orbital plate, entire surface reflective and brilliant appearance; almost bare along parafacial outside facial ridge, with a few reddish-yellow setulae intermingled with facial ridge setae and extending just below lowest frontal setae; facial ridge setose along 2/3 of its length; gena covered in yellow setulae. Antenna, pedicel appearing dark brown or black, overall concolorous with postpedicel; postpedicel dark brown almost black, 3–4X as long as pedicel; arista bare gradually tapering to a point at tip. Palps, burnt umber dark yellow throughout and densely covered in short black setulae; tapering to a sharp point apically, slightly clubbed, devoid of setulae apically. Vibrissa approximately 1 pedicel length from facial margin. **Thorax**: black ground color, with light pale-gray tomentum throughout, appearing glabrous to the naked eye; scutellum ground color light brown, distinctly lighter than scutum, under microscope bronze tomentum throughout becomes visible; scutum with four narrow dorsal vittae, one outer pair, one inner pair, both broken at suture, inner pair extending only slightly beyond first post-sutural dorsocentral seta; lateral surface of thorax densely covered in long hair-like setulae, these setulae all black; chaetotaxy: 3 strong setae on postpronotum arranged in a line, acrostichal setae 3:3; dorsocentral setae 3:4; intra-alar setae 3:3; supra-alar setae 2:3; 4 katepisternal setae; scutellum, with 4–5 pairs of long marginal setae of subequal length; apical scutellar setae short erect, inserted slightly above plane of marginal setae; 1 complete row of scutellar discal setae just posterior to marginal setae. **Wing**: infuscate, slightly darkened yellow/orange at wing base, basicosta mostly dark brown with only slight orange present along caudal margin; both upper and lower calypters also infuscate concolorous with remainder of wing; wing vein R_4+5_ setose, bearing only 2–3 setulae at base; halteres orange. **Legs**: black overall, lightly covered in shimmering bronze tomentum, posterior margin of coxa on midleg and hindleg covered in yellow setulae; tarsal claws yellow-orange with black tips, with burnt umber pulvilli shorter than length of tarsal claws; anterodorsal row of setae on hind tibia irregular and not fringelike, with several longer stronger setae at least 2X as long as others. **Abdomen**: small and rounded globose, black to dark burgundy ground color; tomentum absent from T1+2, light dusting of bronze tomentum on T3 with only very slight gold tomentum along anterior margin, dark bronze tomentum covering anterior 70-80% of surface of T4, bisected medially by an area devoid of tomentum, in some cases this bronze can appear as subdued gold under different angles of light, brilliant gold tomentose throughout T5 reaching to hind margin of tergite; ventral surfaces of T3–T5 extremely densely hirsute but with no distinct sex-patches present, with light gold tomentum throughout; middorsal depression on ST1+2 reaching to hind margin of tergite; ST1+2 with 1 pair of median marginal setae, pairs of median marginal setae present on T3, and complete rows of setae on T4 and T5.

**Male terminalia** (Fig. 79): sternite 5 with a deeply excavated wide median cleft along posterior edge, roughly U-shaped, margins covered in dense tomentum; posterior lobes rounded apically, with a group of strong setulae surrounded by many shorter weaker setulae. Anterior plate of sternite 5 approximately 2/3 length of posterior lobes; unsclerotized "window" on anterior plate of sternite 5 elongate, translucent, rectangular, slightly upturned at extremities. Cerci in posterior view triangular, equal to length of surstyli; pointed at apex, medially to fused along basal 2/3 of their length. Cerci in lateral view, inflated along basal 1/3rd, sharply tapered sinsusoid curved at apical 1/3, giving it a shallow wavy appearance; cerci setose along basal 2/3rds, underside of cerci setose along basal 2/3 of length. Surstylus in lateral view, pointed apically, straight slightly arcuate along inferior margin, and curved along superior margin, scimitar-like in appearance; surstylus appearing not fused with epandrium; when viewed posteriorly surstyli straight not convergent. Pregonite broad, well-developed, apically rounded off, and blunt, with 5–6 marginal setulae. Postgonite, narrow, 1/2 as wide as pregonite, sharply pointed and curved at apex, bladelike, postgonite subequal in length to pregonite. Distiphallus broadly cone-shaped, with a slender median longitudinal sclerotized reinforcement on its posterior surface and a broad, epiphallus appearing as a narrow raised, hooked protuberance, at base of distiphallus, sclerotized acrophallus, blunt and bulbous near apex, 1.2X length of basiphallus.

**Female** (Fig. 80) length: 9–11mm, overall morphology as in male differing in the following traits: **Head**: bearing 1–2 rows of frontal setae and 3–4 pairs of proclinate orbital setae in addition to single pair of reclinate orbital seta. **Abdomen**: dark bronze tomentum covering anterior 70-80% of surface of T4, bisected medially by an area devoid of tomentum, visible as subdued gold under different angles of light and all of T5, much denser than in males; T4 bearing a narrow median black stripe bisecting yellow band; slightly more globose than males.

#### Diagnosis

*Belvosiaosvaldoespinozai* **sp. n.** can be distinguished from all other *Belvosia* by the following combination of traits: yellow setulae on gena, frotoorbital plate silver, black basicosta, and T5 entirely gold tomentose.

#### Etymology

*Belvosiaosvaldoespinozai* **sp. n**, is named in honor of Sr. Osvaldo Espinoza in recognition of his decades of being part of the Parataxonomist Program of Area de Conservación Guanacaste (http://www.acguanacaste.ac.cr) in northwestern Costa Rica (Janzen and Hallwachs 2011). Interim species-specific name included in previously circulating databases and publications, *Belvosia* Woodley14.

#### Distribution

Costa Rica, ACG, Alajuela and Guanacaste Provinces, 320–1000m elevation.

#### Ecology

*Belvosiaosvaldoespinozai* **sp. n.** has been reared 27 times from four species of Lepidoptera in the family Erebidae, *Ochrodotamarina* Schaus, 1910 (N=1), *Ochrodotamarina*DHJ01 (N=3), *Ochrodotamarina*DHJ02 (N=20), *Ochrodotapronapides*BE03 (N=3), in cloud forest, rain forest and dry-rain lowland intergrade.

### Belvosia pabloumanai

Fleming & Woodley sp. nov.

BDC05CD8-B9FD-5339-A262-83B947F3C6DC

652AB911-0BAA-4E9A-AF91-EC68017A817A

#### Materials

- **Type status:** Holotype. **Occurrence:** occurrenceDetails: http://janzen.sas.upenn.edu; catalogNumber: DHJPAR0034347; recordedBy: D.H. Janzen, W. Hallwachs & Jose Perez; individualID: DHJPAR0034347; individualCount: 1; sex: Male; lifeStage: adult; preparations: pinned; otherCatalogNumbers: ASHYC999-09, 09-SRNP-40480, BOLD:AAD7041; occurrenceID: 0B9CC38C-5D12-5FC5-A9A7-91D8F8D59B70; **Taxon:** scientificName: Belvosiapabloumanai; phylum: Arthropoda; class: Insecta; order: Diptera; family: Tachinidae; genus: Belvosia; specificEpithet: pabloumanai; scientificNameAuthorship: Fleming & Woodley, 2023; **Location:** continent: Central America; country: Costa Rica; countryCode: CR; stateProvince: Alajuela; county: Sector Rincon Rain Forest; locality: Area de Conservacion Guanacaste; verbatimLocality: Camino Porvenir; verbatimElevation: 383; verbatimLatitude: 10.9038; verbatimLongitude: -85.2596; verbatimCoordinateSystem: Decimal; decimalLatitude: 10.9038; decimalLongitude: -85.2596; **Identification:** identifiedBy: AJ Fleming; dateIdentified: 2022; **Event:** samplingProtocol: Reared from the larvae of the Notodontidae, Antaealichyi; verbatimEventDate: 11-May-2009; **Record Level:** language: en; institutionCode: CNC; collectionCode: Insects; basisOfRecord: Pinned Specimen
- **Type status:** Paratype. **Occurrence:** occurrenceDetails: http://janzen.sas.upenn.edu; catalogNumber: DHJPAR0001253; recordedBy: D.H. Janzen, W. Hallwachs & Petrona Rios; individualID: DHJPAR0001253; individualCount: 1; sex: Male; lifeStage: adult; preparations: pinned; otherCatalogNumbers: HCIC140-05, 03-SRNP-37038, BOLD:AAD7041; occurrenceID: CE8744DE-D778-5FE5-9129-D00EBBC633E1; **Taxon:** scientificName: Belvosiapabloumanai; phylum: Arthropoda; class: Insecta; order: Diptera; family: Tachinidae; genus: Belvosia; specificEpithet: pabloumanai; scientificNameAuthorship: Fleming & Woodley, 2023; **Location:** continent: Central America; country: Costa Rica; countryCode: CR; stateProvince: Guanacaste; county: Sector Pitilla; locality: Area de Conservacion Guanacaste; verbatimLocality: Pasmompa; verbatimElevation: 440; verbatimLatitude: 11.0193; verbatimLongitude: -85.41; verbatimCoordinateSystem: Decimal; decimalLatitude: 11.0193; decimalLongitude: -85.41; **Identification:** identifiedBy: AJ Fleming; dateIdentified: 2022; **Event:** samplingProtocol: Reared from the larvae of the Notodontidae, Hapigiarepandens; verbatimEventDate: 03-Feb-2004; **Record Level:** language: en; institutionCode: CNC; collectionCode: Insects; basisOfRecord: Pinned Specimen
- **Type status:** Paratype. **Occurrence:** occurrenceDetails: http://janzen.sas.upenn.edu; catalogNumber: DHJPAR0001254; recordedBy: D.H. Janzen, W. Hallwachs & Fraysi Vargas; individualID: DHJPAR0001254; individualCount: 1; sex: Female; lifeStage: adult; preparations: pinned; otherCatalogNumbers: HCIC148-05, 02-SRNP-6337, BOLD:AAD7041; occurrenceID: 456F0B2E-BD01-58C5-AF4E-640F9E6C15C1; **Taxon:** scientificName: Belvosiapabloumanai; phylum: Arthropoda; class: Insecta; order: Diptera; family: Tachinidae; genus: Belvosia; specificEpithet: pabloumanai; scientificNameAuthorship: Fleming & Woodley, 2023; **Location:** continent: Central America; country: Costa Rica; countryCode: CR; stateProvince: Alajuela; county: Sector Rincon Rain Forest; locality: Area de Conservacion Guanacaste; verbatimLocality: Sendero Rincon; verbatimElevation: 430; verbatimLatitude: 10.8962; verbatimLongitude: -85.2777; verbatimCoordinateSystem: Decimal; decimalLatitude: 10.8962; decimalLongitude: -85.2777; **Identification:** identifiedBy: AJ Fleming; dateIdentified: 2022; **Event:** samplingProtocol: Reared from the larvae of the Notodontidae, Antaealichyi; verbatimEventDate: 02-Apr-2002; **Record Level:** language: en; institutionCode: CNC; collectionCode: Insects; basisOfRecord: Pinned Specimen

#### Description

**Male** (Fig. 81), length: 12–15mm. **Head**: head wider than thorax; vertex 1/3 head width; gena 1/3 of head height, 1/2 of eye height. Fronto-orbital plate silver tomentose throughout, with one row of frontal setae, populated with short black hair-like setulae intermingled with setae; ocellar setae absent; 1 pair of reclinate orbital setae present outside frontal row. Parafacial, densely covered in same silver tomentum as on fronto-orbital plate, entire surface reflective and brilliant appearance; almost bare along parafacial outside facial ridge, with a few black setulae intermingled with facial ridge setae and extending just below lowest frontal setae; facial ridge setose along 2/3 of its length; gena covered in yellow setulae. Antenna, pedicel appearing dark orange almost black, overall approaching color of postpedicel; postpedicel dark brown almost black, 3–4X as long as pedicel; arista bare gradually tapering to a point at tip. Palps, dark yellow throughout and densely covered in short black setulae; tapering to a sharp point apically, slightly spade shaped, devoid of setulae apically. Vibrissa approximately 1 pedicel length from facial margin. **Thorax**: black ground color, with light pale-gray tomentum throughout, appearing dusty to the naked eye; scutellum ground color light brown, distinctly lighter than scutum, under microscope bronze tomentum throughout becomes visible; scutum with four narrow dorsal vittae, one outer pair, one inner pair, both broken at suture, inner pair extending only slightly beyond first post-sutural dorsocentral seta; lateral surface of thorax densely covered in long hair-like setulae, these setulae all black; chaetotaxy: 3 strong setae on postpronotum arranged in a line, acrostichal setae 4:4; dorsocentral setae 3:4; intra-alar setae 2:4; supra-alar setae 2:3; 4 katepisternal setae; scutellum, with 4–5 pairs of long marginal setae of subequal length; apical scutellar setae short erect, inserted slightly above plane of marginal setae; 1 complete row of scutellar discal setae just posterior to marginal setae. **Wing**: infuscate, slightly darkened brown at wing base, basicosta mostly dark brown with only slight orange present along caudal margin; both upper and lower calypters also infuscate concolorous with remainder of wing; wing vein R_4+5_ setose, bearing only 2–3 setulae at base; halteres orange. **Legs**: black overall; tarsal claws yellow-orange with black tips, with burnt umber pulvilli shorter than length of tarsal claws; anterodorsal row of setae on hind tibia irregular and not fringelike, with several longer stronger setae at least 2X as long as others. **Abdomen**: small and rounded globose, black to dark burgundy ground color; tomentum absent from T1+2, light dusting of bronze tomentum on T3 with only very slight gold tomentum along anterior margin, subdued gold tomentum along anterior 20-40% of surface of T4, bisected medially by an area devoid of tomentum, brilliant gold tomentose throughout 95% of T5 reaching with black on hind margin of tergite; ventral surfaces of T3–T5 extremely densely hirsute but with no distinct sex-patches present, with light gold tomentum throughout; middorsal depression on ST1+2 reaching to hind margin of tergite; ST1+2 with 1 pair of median marginal setae, pairs of median marginal setae present on T3, and complete rows of setae on T4 and T5.

**Male terminalia** (Fig. 82): sternite 5 with a deeply excavated median cleft along posterior edge, roughly U-shaped, margins covered in dense tomentum; posterior lobes rounded apically, with a group of strong setulae surrounded by many shorter weaker setulae. Anterior plate of sternite 5 approximately 2/3 length of posterior lobes; unsclerotized "window" on anterior plate of sternite 5 translucent, rectangular, slightly arcuate. Cerci in posterior view short triangular, equal to length of surstyli, slightly inflated at midpoint; pointed at apex, medially to fused along basal 2/3 of their length. Cerci in lateral view, inflated along basal 1/3rd, sharply tapered with anterior curved at apical 1/3, giving it a shallow hooked appearance; cerci setose along basal 2/3rds, underside of cerci setose along basal 2/3 of length. Surstylus in lateral view, wide rounded apically, straight along inferior margin; surstylus appearing not fused with epandrium; when viewed posteriorly surstyli convergent. Pregonite broad, well-developed, apically squared off, and blunt, with 5–6 marginal setulae. Postgonite, narrow, 1/2 as wide as pregonite, sharply pointed and curved at apex, bladelike, postgonite subequal in length to pregonite. Distiphallus broadly cone-shaped, with a slender median longitudinal sclerotized reinforcement on its posterior surface and a broad, epiphallus appearing as a small raised protuberance at base of distiphallus, sclerotized acrophallus, blunt and bulbous near apex, 1.7X length of basiphallus.

**Female** (Fig. 83) length: 12–15mm, overall morphology as in male differing in the following traits: **Head**: bearing 1–2 rows of frontal setae and 2–3 pairs of proclinate orbital setae in addition to single pair of reclinate orbital seta; gena 1/4 head height and 1/3 of eye height. **Abdomen**: gold tomentum covering anterior 70-80% of surface of T4, bisected medially by an area devoid of tomentum, and all of T5, much denser than in males; T4 bearing a narrow median black stripe bisecting yellow band; slightly more globose than males.

#### Diagnosis

*Belvosiapabloumanai* **sp. n.** can be distinguished from all other *Belvosia* by the following combination of traits: gena covered in yellow setulae, dark basicosta, scutum mostly silver tomentose, and T5 black apically.

#### Etymology

*Belvosiapabloumanai* **sp. n**, is named in honor of Sr. Pablo Umaña in recognition of his decades of being part of the Parataxonomist Program of Area de Conservación Guanacaste (http://www.acguanacaste.ac.cr) in northwestern Costa Rica (Janzen and Hallwachs 2011). Interim species-specific name included in previously circulating databases and publications, *Belvosia* Woodley15.

#### Distribution

Costa Rica, ACG, Alajuela and Guanacaste Provinces, 383–585m elevation.

#### Ecology

*Belvosiapabloumanai* **sp. n.** has been reared six times from two species of Lepidoptera in the family Notodontidae, *Antaealichyi* Franclemont, 1942 (N=2), *Hapigiarepandens* Schaus, 1905 (N=4), in rain forest and dry-rain lowland intergrade.

### Belvosia petronariosae

Fleming & Woodley sp. nov.

F634464D-6340-5EE3-9705-CB7A84602E86

4FE63F7D-4870-402D-BE5F-6EA9C861BC86

#### Materials

- **Type status:** Holotype. **Occurrence:** occurrenceDetails: http://janzen.sas.upenn.edu; catalogNumber: DHJPAR0001226; recordedBy: D.H. Janzen, W. Hallwachs & gusaneros; individualID: DHJPAR0001226; individualCount: 1; sex: Male; lifeStage: adult; preparations: pinned; otherCatalogNumbers: HCIC118-05, 01-SRNP-21309, BOLD:AAB0407; occurrenceID: 1AA8A2F6-5602-5A52-8499-2665F155E505; **Taxon:** scientificName: Belvosiapetronariosae; phylum: Arthropoda; class: Insecta; order: Diptera; family: Tachinidae; genus: Belvosia; specificEpithet: petronariosae; scientificNameAuthorship: Fleming & Woodley, 2023; **Location:** continent: Central America; country: Costa Rica; countryCode: CR; stateProvince: Guanacaste; county: Sector Cacao; locality: Area de Conservacion Guanacaste; verbatimLocality: Sendero Maritza; verbatimElevation: 760; verbatimLatitude: 10.9364; verbatimLongitude: -85.4776; verbatimCoordinateSystem: Decimal; decimalLatitude: 10.9364; decimalLongitude: -85.4776; **Identification:** identifiedBy: AJ Fleming; dateIdentified: 2022; **Event:** samplingProtocol: Reared from the larvae of the Saturniidae, Arsenuraarianae; verbatimEventDate: 02-Mar-2002; **Record Level:** language: en; institutionCode: CNC; collectionCode: Insects; basisOfRecord: Pinned Specimen
- **Type status:** Paratype. **Occurrence:** occurrenceDetails: http://janzen.sas.upenn.edu; catalogNumber: DHJPAR0016353; recordedBy: D.H. Janzen, W. Hallwachs & Jose Alberto Sanchez; individualID: DHJPAR0016353; individualCount: 1; sex: Male; lifeStage: adult; preparations: pinned; otherCatalogNumbers: ASTAP382-06, 06-SRNP-57011, BOLD:AAB0407; occurrenceID: AF6A8384-278E-5C46-8089-89F04548DA14; **Taxon:** scientificName: Belvosiapetronariosae; phylum: Arthropoda; class: Insecta; order: Diptera; family: Tachinidae; genus: Belvosia; specificEpithet: petronariosae; scientificNameAuthorship: Fleming & Woodley, 2023; **Location:** continent: Central America; country: Costa Rica; countryCode: CR; stateProvince: Guanacaste; county: Sector Mundo Nuevo; locality: Area de Conservacion Guanacaste; verbatimLocality: Sendero Guanacaste; verbatimElevation: 660; verbatimLatitude: 10.7782; verbatimLongitude: -85.3946; verbatimCoordinateSystem: Decimal; decimalLatitude: 10.7782; decimalLongitude: -85.3946; **Identification:** identifiedBy: AJ Fleming; dateIdentified: 2022; **Event:** samplingProtocol: Reared from the larvae of the Saturniidae, Arsenuraarianae; verbatimEventDate: 20-Sep-2006; **Record Level:** language: en; institutionCode: CNC; collectionCode: Insects; basisOfRecord: Pinned Specimen
- **Type status:** Paratype. **Occurrence:** occurrenceDetails: http://janzen.sas.upenn.edu; catalogNumber: DHJPAR0016356; recordedBy: D.H. Janzen, W. Hallwachs & gusaneros; individualID: DHJPAR0016356; individualCount: 1; sex: Female; lifeStage: adult; preparations: pinned; otherCatalogNumbers: ASTAP385-06, 06-SRNP-21926, BOLD:AAB0407; occurrenceID: EF83A078-383C-525E-B9DD-DDD2E178C7C7; **Taxon:** scientificName: Belvosiapetronariosae; phylum: Arthropoda; class: Insecta; order: Diptera; family: Tachinidae; genus: Belvosia; specificEpithet: petronariosae; scientificNameAuthorship: Fleming & Woodley, 2023; **Location:** continent: Central America; country: Costa Rica; countryCode: CR; stateProvince: Guanacaste; county: Sector Del Oro; locality: Area de Conservacion Guanacaste; verbatimLocality: San Antonio; verbatimElevation: 335; verbatimLatitude: 11.0353; verbatimLongitude: -85.4453; verbatimCoordinateSystem: Decimal; decimalLatitude: 11.0353; decimalLongitude: -85.4453; **Identification:** identifiedBy: AJ Fleming; dateIdentified: 2022; **Event:** samplingProtocol: Reared from the larvae of the Saturniidae, Arsenuraarianae; verbatimEventDate: 14-Sep-2006; **Record Level:** language: en; institutionCode: CNC; collectionCode: Insects; basisOfRecord: Pinned Specimen

#### Description

**Male** (Fig. 84), length: 14–15mm. **Head**: head wider than thorax; vertex 1/2 head width; gena 1/4 of head height, 1/3 of eye height. Fronto-orbital plate silver-gray tomentose throughout, sometimes a bit lightly so along vertex, with 1–2 rows of frontal setae, populated with short black hair-like setulae intermingled with setae; ocellar setae absent; orbital setae absent. Parafacial, densely covered in same silver tomentum as on fronto-orbital plate, entire surface reflective and brilliant appearance; almost bare along parafacial outside facial ridge, with a few black setulae intermingled with facial ridge setae and extending just below lowest frontal setae; facial ridge setose along 2/3 of its length; gena covered in black setulae. Antenna, pedicel appearing orange almost covered in a silver tomentum; postpedicel dark brown almost black, 3–4X as long as pedicel; arista bare gradually tapering to a point at tip. Palps, dark yellow throughout and sparsely covered in short black setulae; tapering to a rounded apex, slightly sinusoid and clubbed shaped, devoid of setulae apically. Vibrissa approximately 1 pedicel length from facial margin. **Thorax**: black ground color along anterior portion, lightening to yellow orange along posterior 1/10th of scutum, with light pale-gray tomentum throughout, appearing dusty to the naked eye; scutellum ground color light brown, distinctly lighter than scutum, under microscope bronze tomentum throughout becomes visible; scutum with four narrow dorsal vittae, one outer pair, one inner pair, both broken at suture, inner pair extending only slightly beyond first post-sutural dorsocentral seta; lateral surface of thorax densely covered in long hair-like setulae, these setulae all black; chaetotaxy: 3–5 strong setae on postpronotum arranged in a line, acrostichal setae 4:4; dorsocentral setae 3:4; intra-alar setae 2:4 separated from dorsocentrals by 2X the gap separating dorsocentral setea from acrostichal setae; supra-alar setae 2:3; 4 katepisternal setae; scutellum, with 4–5 pairs of long marginal setae of subequal length; apical scutellar setae short erect, inserted slightly above plane of marginal setae; 1 complete row of scutellar discal setae just posterior to marginal setae. **Wing**: infuscate, slightly darkened brown at wing base, basicosta dark brown with only a slight accent of orange on margin; both upper and lower calypters also infuscate concolorous with remainder of wing; wing vein R_4+5_ setose, bearing only 2–3 setulae at base; halteres orange. **Legs**: black overall; tarsal claws yellow-orange with black tips, with burnt umber pulvilli shorter than length of tarsal claws; anterodorsal row of setae on hind tibia irregular and not fringelike, with several longer stronger setae at least 2X as long as others. **Abdomen**: large and flattened globose, black to dark burgundy ground color; tomentum absent from T1+2, light dusting of bronze tomentum on T3 with only very slight gold tomentum along anterior margin, gold tomentum along anterior 15% of surface of T4, bisected medially by an area devoid of tomentum, brilliant gold tomentose throughout 95% of T5 reaching with black on hind margin of tergite; ventral surfaces of T3–T5 extremely densely hirsute but with no distinct sex-patches present, with light gold tomentum throughout; middorsal depression on ST1+2 reaching to hind margin of tergite; ST1+2 with 1 pair of median marginal setae, pairs of median marginal setae present on T3, and complete rows of setae on T4 and T5.

**Male terminalia** (Fig. 85) : sternite 5 with a deeply excavated median cleft along posterior edge, roughly Y-shaped, with soft shoulders, margins covered in dense tomentum; posterior lobes rounded apically, with a group of 4–5 strong setulae surrounded by many shorter weaker setulae. Anterior plate of sternite 5 approximately 1/2 length of posterior lobes; unsclerotized "window" on anterior plate of sternite 5 translucent, rectangular, slightly arcuate. Cerci in posterior view short triangular, equal to length of surstyli; pointed at apex, medially to fused along basal 2/3 of their length. Cerci in lateral view, inflated along basal 2/3rds, sharply tapered with anterior curved at apical 1/3, giving it a shallow hooked appearance; cerci setose along basal 2/3rds, underside of cerci setose along basal 2/3 of length. Surstylus in lateral view, wide rounded apically, straight along inferior margin; surstylus appearing not fused with epandrium; when viewed posteriorly surstyli convergent. Pregonite broad, well-developed, apically squared off, and blunt, with 5–6 marginal setulae. Postgonite, narrow, 1/2 as wide as pregonite, sharply pointed and curved at apex, bladelike, postgonite subequal in length to pregonite. Distiphallus broadly cone-shaped, with a slender median longitudinal sclerotized reinforcement on its posterior surface and a broad, anterolateral, sclerotized acrophallus, bearing a slight anterior hook on anterior surface near apex, 1.3X length of basiphallus.

**Female** (Fig. 86) length: 14–15mm, overall morphology as in male differing in the following traits: **Head**: bearing 2 rows of frontal setae and 3–4 pairs of proclinate orbital setae in addition to single pair of reclinate orbital seta; gena 1/3 of head height, 2/5 of eye height. **Abdomen**: gold tomentum covering anterior 10% of surface of T4, bisected medially by an area devoid of tomentum, and all of T5, much denser than in males; T4 bearing a narrow median black stripe bisecting yellow band; slightly more globose than males.

#### Diagnosis

*Belvosiapetronariosae* **sp. n.** can be distinguished from all other *Belvosia* by the following combination of traits: yellow setulae on gena, orange basicosta, abdominal ground color orange, postocular margin of head gold tomentose.

#### Etymology

*Belvosiapetronariosae* **sp. n.**, is named in honor of Sra. Petrona Rios in recognition of her decades of being part of the Parataxonomist Program of Area de Conservación Guanacaste (http://www.acguanacaste.ac.cr) in northwestern Costa Rica (Janzen and Hallwachs 2011). Interim species-specific name included in previously circulating databases and publications, *Belvosia* Woodley16.

#### Distribution

Costa Rica, ACG, Guanacaste Province, 280–760m elevation.

#### Ecology

*Belvosiapetronariosae* **sp. n.** has been reared 79 times from one species of Lepidoptera in the family Saturniidae, *Arsenuraarianae* Brechlin & Meister, 2010 (N=79), in cloud forest, rain forest, dry forest, and dry-rain lowland intergrade.

### Belvosia ricardocaleroi

Fleming & Woodley sp. nov.

B3455F31-787D-57B5-B316-B8D72CDE8D4B

37F5F5AA-DA57-43E4-8AE1-08ACECF7A527

#### Materials

- **Type status:** Holotype. **Occurrence:** occurrenceDetails: http://janzen.sas.upenn.edu; catalogNumber: DHJPAR0001232; recordedBy: D.H. Janzen, W. Hallwachs & Harry Ramirez; individualID: DHJPAR0001232; individualCount: 1; sex: Male; lifeStage: adult; preparations: pinned; otherCatalogNumbers: HCIC166-05, 02-SRNP-8786,; occurrenceID: E87E8F3F-C710-5762-B8FD-D99A642237EC; **Taxon:** scientificName: Belvosiaricardocaleroi; phylum: Arthropoda; class: Insecta; order: Diptera; family: Tachinidae; genus: Belvosia; specificEpithet: ricardocaleroi; scientificNameAuthorship: Fleming & Woodley, 2023; **Location:** continent: Central America; country: Costa Rica; countryCode: CR; stateProvince: Guanacaste; county: Sector Cacao; locality: Area de Conservacion Guanacaste; verbatimLocality: Estacion Cacao; verbatimElevation: 1150; verbatimLatitude: 10.9269; verbatimLongitude: -85.4682; verbatimCoordinateSystem: Decimal; decimalLatitude: 10.9269; decimalLongitude: -85.4682; **Identification:** identifiedBy: AJ Fleming; dateIdentified: 2022; **Event:** samplingProtocol: Reared from the larvae of the Noctuidae, Mythimnia sequax; verbatimEventDate: 18-May-2002; **Record Level:** language: en; institutionCode: CNC; collectionCode: Insects; basisOfRecord: Pinned Specimen
- **Type status:** Paratype. **Occurrence:** occurrenceDetails: http://janzen.sas.upenn.edu; catalogNumber: DHJPAR0001237; recordedBy: D.H. Janzen, W. Hallwachs & gusaneros; individualID: DHJPAR0001237; individualCount: 1; sex: Female; lifeStage: adult; preparations: pinned; otherCatalogNumbers: HCIC109-05, 94-SRNP-3141,; occurrenceID: C1F527D0-673B-5E47-849C-CA2B592EEAA0; **Taxon:** scientificName: Belvosiaricardocaleroi; phylum: Arthropoda; class: Insecta; order: Diptera; family: Tachinidae; genus: Belvosia; specificEpithet: ricardocaleroi; scientificNameAuthorship: Fleming & Woodley, 2023; **Location:** continent: Central America; country: Costa Rica; countryCode: CR; stateProvince: Guanacaste; county: Sector Santa Rosa; locality: Area de Conservacion Guanacaste; verbatimLocality: Vado Cuajiniquil; verbatimElevation: 275; verbatimLatitude: 10.9404; verbatimLongitude: -85.6804; verbatimCoordinateSystem: Decimal; decimalLatitude: 10.9404; decimalLongitude: -85.6804; **Identification:** identifiedBy: AJ Fleming; dateIdentified: 2022; **Event:** samplingProtocol: Reared from the larvae of the Noctuidae, Mythimnia sequax; verbatimEventDate: 26-Jun-1994; **Record Level:** language: en; institutionCode: CNC; collectionCode: Insects; basisOfRecord: Pinned Specimen
- **Type status:** Paratype. **Occurrence:** occurrenceDetails: http://janzen.sas.upenn.edu; catalogNumber: DHJPAR0001230; recordedBy: D.H. Janzen, W. Hallwachs & Harry Ramirez; individualID: DHJPAR0001230; individualCount: 1; sex: Male; lifeStage: adult; preparations: pinned; otherCatalogNumbers: HCIC150-05, 02-SRNP-8747,; occurrenceID: 4375C6AB-8538-5619-858C-2D4A7A0BA4F9; **Taxon:** scientificName: Belvosiaricardocaleroi; phylum: Arthropoda; class: Insecta; order: Diptera; family: Tachinidae; genus: Belvosia; specificEpithet: ricardocaleroi; scientificNameAuthorship: Fleming & Woodley, 2023; **Location:** continent: Central America; country: Costa Rica; countryCode: CR; stateProvince: Guanacaste; county: Sector Cacao; locality: Area de Conservacion Guanacaste; verbatimLocality: Estacion Cacao; verbatimElevation: 1150; verbatimLatitude: 10.9269; verbatimLongitude: -85.4682; verbatimCoordinateSystem: Decimal; decimalLatitude: 10.9269; decimalLongitude: -85.4682; **Identification:** identifiedBy: AJ Fleming; dateIdentified: 2022; **Event:** samplingProtocol: Reared from the larvae of the Noctuidae, Mythimnia sequax; verbatimEventDate: 18-May-2002; **Record Level:** language: en; institutionCode: CNC; collectionCode: Insects; basisOfRecord: Pinned Specimen

#### Description

**Male** (Fig. 87), length: 9–11mm. **Head**: head wider than thorax; vertex 1/3 head width; gena 1/5 of head height, 1/3 of eye height. Fronto-orbital plate light gold tomentose to glabrous, with two rows of frontal setae, populated with short black hair-like setulae intermingled with setae; ocellar setae absent; 2 pairs of proclinate orbital setae and 1 pair of reclinate orbital setae present outside frontal row. Parafacial, densely covered in same gold tomentum as on fronto-orbital plate, entire surface reflective and brilliant appearance; bare along parafacial outside facial ridge, with a few black setulae intermingled with facial ridge setae and extending just below lowest frontal setae; facial ridge setose along 3/4 of its length; gena covered in yellow setulae. Antenna, pedicel orange; postpedicel dark brown almost black, 4–5X as long as pedicel; arista bare gradually tapering to a point at tip. Palps, dark yellow throughout and densely covered in short black setulae; clubbed. Vibrissa approximately 1/2 pedicel length from facial margin. **Thorax**: yellow ground color, with light pale-gray/gold tomentum dorsally, appearing dusty to the naked eye; scutellum ground color light yellow, slightly lighter than scutum, under microscope bronze tomentum throughout becomes visible; scutum with four narrow dorsal vittae, one outer pair, one inner pair, both broken at suture, inner pair extending only slightly beyond first post-sutural dorsocentral seta; lateral surface of thorax densely covered in long hair-like setulae, these setulae all yellow and whispy; chaetotaxy: 3 strong setae on postpronotum arranged in a line, acrostichal setae 3:3; dorsocentral setae 3:4; intra-alar setae 2:4; supra-alar setae 2:3; 4 katepisternal setae; scutellum, with 4–5 pairs of long marginal setae of subequal length; apical scutellar setae short erect, inserted slightly above plane of marginal setae; 1 complete row of scutellar discal setae just posterior to marginal setae. **Wing**: infuscate, slightly darkened brown at wing base, basicosta brilliant orange; both upper and lower calypters also white translucent; wing vein R_4+5_ setose, bearing only 2–3 setulae at base; halteres orange. **Legs**: black overall, lightly covered in shimmering bronze tomentum, posterior margin of coxa on midleg and hindleg covered in yellow setulae; tarsal claws yellow-orange with black tips, with burnt umber pulvilli shorter than length of tarsal claws; anterodorsal row of setae on hind tibia irregular and not fringelike, with several longer stronger setae at least 2X as long as others. **Abdomen**: small and elongate globose, orange ground color; T1+2 with a light dusting of gray-gold tomentum mid-dorsally along depression, T3–T4 with a light dusting of gray gold tomentum dorsally, darker on T4, and brilliant gold tomentose throughout all of T5; ventral surfaces of T3–T5 with no distinct sex-patches present, and light gold tomentum throughout; middorsal depression on ST1+2 reaching to hind margin of tergite; ST1+2 with 1 pair of median marginal setae, 1 pair of median marginal setae present on T3, and complete rows of setae on T4 and T5.

**Male terminalia** (Fig. 88): sternite 5 with a deeply excavated median cleft along posterior edge, roughly Y-shaped, with reduced shoulders, margins covered in dense tomentum; posterior lobes rounded apically, with a group of strong setulae surrounded by many shorter weaker setulae. Anterior plate of sternite 5 approximately 1/2 length of posterior lobes; unsclerotized "window" on anterior plate of sternite 5 translucent, elongate and rectangular. Cerci in posterior view short bulbous basally, with a strong shoulder at midway tapered to ovoid, shorter than surstyli; rounded at apex, medially fused along 1/2 of their length. Cerci in lateral view, inflated along basal 2/3rds, sharply tapered with a curve at apical 1/3, giving it a shallow hooked appearance, caudal edge of apex of cerci protruding; cerci setose along basal 2/3rds, underside of cerci setose along basal 1/2 of length. Surstylus in lateral view, equilateral along its length rounded apically, straight, digitiform; surstylus appearing to be fused with epandrium; when viewed posteriorly surstyli straight. Pregonite narrow, well-developed, apically pointed, devoid of setulae. Postgonite, slightly narrow, as wide as pregonite, sharply pointed and straight, bladelike, with one small setula, postgonite subequal in length to pregonite. Distiphallus broadly cone-shaped, with a slender median longitudinal sclerotized reinforcement on its posterior surface and a broad, anterolateral, sclerotized acrophallus, bearing a slight anterior hook on anterior surface near apex, 1.6X length of basiphallus.

**Female** (Fig. 89) length: 9–12mm, overall morphology as in male differing in the following traits: **Head**: with 2–3 pairs of proclinate orbital setae in addition to single pair of reclinate orbital seta. **Abdomen**: as in male except for in its terminalia.

#### Diagnosis

*Belvosiaricardocaleroi* **sp. n.** can be distinguished from all other *Belvosia* by the following combination of traits: yellow setulae on gena, orange basicosta, abdominal ground color orange, postocular margin of head gold tomentose.

#### Etymology

*Belvosiaricardocaleroi* **sp. n**, is named in honor of Sr. Ricardo Calero in recognition of his decades of being part of the Parataxonomist Program of Area de Conservación Guanacaste (http://www.acguanacaste.ac.cr) in northwestern Costa Rica (Janzen and Hallwachs 2011). Interim species-specific name included in previously circulating databases and publications, *Belvosia* Woodley17.

#### Distribution

Costa Rica, ACG, Guanacaste Province, 275–1150m elevation.

#### Ecology

*Belvosiaricardocaleroi* **sp. n.** has been reared 20 times from one species of Lepidoptera in the family Noctuidae, *Mythimnasequax* (Franclemont, 1951) (N=20), in cloud forest, and dry forest.

### Belvosia robertoespinozai

Fleming & Woodley sp. nov.

84AA1AC6-0ED3-5675-B4BA-123FA5215D8E

130CB826-AD90-432E-B7C1-7AF45EA52C25

#### Materials

- **Type status:** Holotype. **Occurrence:** occurrenceDetails: http://janzen.sas.upenn.edu; catalogNumber: DHJPAR0045539; recordedBy: D.H. Janzen, W. Hallwachs & Lucia Rios; individualID: DHJPAR0045539; individualCount: 1; sex: Male; lifeStage: adult; preparations: pinned; otherCatalogNumbers: ACGAZ728-11, 11-SRNP-21027, BOLD:AAF0099; occurrenceID: D2530FE5-CFE7-5F84-B4FA-1BB4391669D7; **Taxon:** scientificName: Belvosiarobertoespinozai; phylum: Arthropoda; class: Insecta; order: Diptera; family: Tachinidae; genus: Belvosia; specificEpithet: robertoespinozai; scientificNameAuthorship: Fleming & Woodley, 2023; **Location:** continent: Central America; country: Costa Rica; countryCode: CR; stateProvince: Guanacaste; county: Sector El Hacha; locality: Area de Conservacion Guanacaste; verbatimLocality: Estacion Los Almendros; verbatimElevation: 290; verbatimLatitude: 11.0323; verbatimLongitude: -85.5278; verbatimCoordinateSystem: Decimal; decimalLatitude: 11.0323; decimalLongitude: -85.5278; **Identification:** identifiedBy: AJ Fleming; dateIdentified: 2022; **Event:** samplingProtocol: Reared from the larvae of the Sphingidae, Xylophanestyndarus; verbatimEventDate: 23-Sep-2011; **Record Level:** language: en; institutionCode: CNC; collectionCode: Insects; basisOfRecord: Pinned Specimen
- **Type status:** Paratype. **Occurrence:** occurrenceDetails: http://janzen.sas.upenn.edu; catalogNumber: DHJPAR0016360; recordedBy: D.H. Janzen, W. Hallwachs & Harry Ramirez; individualID: DHJPAR0016360; individualCount: 1; sex: Male; lifeStage: adult; preparations: pinned; otherCatalogNumbers: ASTAP389-06, 06-SRNP-45620, BOLD:AAF0099; occurrenceID: 159389BB-93F9-55A1-8924-2C87C7E94327; **Taxon:** scientificName: Belvosiarobertoespinozai; phylum: Arthropoda; class: Insecta; order: Diptera; family: Tachinidae; genus: Belvosia; specificEpithet: robertoespinozai; scientificNameAuthorship: Fleming & Woodley, 2023; **Location:** continent: Central America; country: Costa Rica; countryCode: CR; stateProvince: Guanacaste; county: Sector Cacao; locality: Area de Conservacion Guanacaste; verbatimLocality: Quebrada Otilio; verbatimElevation: 550; verbatimLatitude: 10.89; verbatimLongitude: -85.4797; verbatimCoordinateSystem: Decimal; decimalLatitude: 10.89; decimalLongitude: -85.4797; **Identification:** identifiedBy: AJ Fleming; dateIdentified: 2022; **Event:** samplingProtocol: Reared from the larvae of the Sphingidae, Xylophanestyndarus; verbatimEventDate: 23-Aug-2006; **Record Level:** language: en; institutionCode: CNC; collectionCode: Insects; basisOfRecord: Pinned Specimen
- **Type status:** Paratype. **Occurrence:** occurrenceDetails: http://janzen.sas.upenn.edu; catalogNumber: DHJPAR0016364; recordedBy: D.H. Janzen, W. Hallwachs & Harry Ramirez; individualID: DHJPAR0016364; individualCount: 1; sex: Female; lifeStage: adult; preparations: pinned; otherCatalogNumbers: ASTAP393-06, 06-SRNP-45615, BOLD:AAF0099; occurrenceID: 11420998-8B50-54B5-BCDD-296D9500B8C9; **Taxon:** scientificName: Belvosiarobertoespinozai; phylum: Arthropoda; class: Insecta; order: Diptera; family: Tachinidae; genus: Belvosia; specificEpithet: robertoespinozai; scientificNameAuthorship: Fleming & Woodley, 2023; **Location:** continent: Central America; country: Costa Rica; countryCode: CR; stateProvince: Guanacaste; county: Sector Cacao; locality: Area de Conservacion Guanacaste; verbatimLocality: Quebrada Otilio; verbatimElevation: 550; verbatimLatitude: 10.89; verbatimLongitude: -85.4797; verbatimCoordinateSystem: Decimal; decimalLatitude: 10.89; decimalLongitude: -85.4797; **Identification:** identifiedBy: AJ Fleming; dateIdentified: 2022; **Event:** samplingProtocol: Reared from the larvae of the Sphingidae, Xylophanestyndarus; verbatimEventDate: 23-Oct-2006; **Record Level:** language: en; institutionCode: CNC; collectionCode: Insects; basisOfRecord: Pinned Specimen

#### Description

**Male** (Fig. 90), length: 13–14mm. **Head**: head wider than thorax; vertex 1/3 head width; gena 1/3 of head height, 1/2 of eye height. Fronto-orbital plate silver tomentose throughout, with two rows of frontal setae, populated with short black hair-like setulae intermingled with setae; ocellar setae absent; orbital setae absent. Parafacial, densely covered in same silver tomentum as on fronto-orbital plate, entire surface reflective and brilliant appearance; almost bare along parafacial outside facial ridge, with a few black setulae intermingled with facial ridge setae and extending just below lowest frontal setae; facial ridge setose along 2/3 of its length; gena covered in black setulae. Antenna, pedicel black, concolorous with postpedicel; postpedicel black, less than 2X as long as pedicel; arista bare gradually tapering to a point at tip. Palps, dark yellow throughout and densely covered in short black setulae; tapering to a sharp point apically, devoid of setulae medially. Vibrissa approximately 1–1.5X pedicel length from facial margin. **Thorax**: black ground color, with gray tomentum throughout, appearing dusty to the naked eye; scutellum ground color dark brown, distinctly lighter than scutum, under microscope bronze tomentum throughout becomes visible; scutum with four narrow dorsal vittae, one outer pair, one inner pair, both broken at suture, inner pair extending only slightly beyond first post-sutural dorsocentral seta; lateral surface of thorax densely covered in long hair-like setulae, these setulae all black; chaetotaxy: 3 strong setae on postpronotum arranged in a line, acrostichal setae 3:4; dorsocentral setae 2–3:4; intra-alar setae 3:3; supra-alar setae 2:3; 4–6 katepisternal setae; scutellum, with 4–5 pairs of long marginal setae of subequal length; apical scutellar setae short erect, inserted slightly above plane of marginal setae; 1 complete row of scutellar discal setae just posterior to marginal setae. **Wing**: infuscate, slightly darkened brown at wing base, basicosta mostly dark brown with only slight orange present along caudal margin; both upper and lower calypters also infuscate concolorous with remainder of wing; wing vein R_4+5_ setose, bearing only 2–3 setulae at base; halteres orange. **Legs**: black overall, lightly covered in shimmering bronze tomentum, posterior margin of coxa on midleg and hindleg covered in yellow setulae; tarsal claws yellow-orange with black tips, with burnt umber pulvilli shorter than length of tarsal claws; anterodorsal row of setae on hind tibia irregular and not fringelike, with several longer stronger setae at least 2X as long as others. **Abdomen**: large and flattened globose, black to dark burgundy ground color; tomentum absent from T1+2–T3 with only very slight gold tomentum along anterior 10% of margin of T4, bisected medially by an area devoid of tomentum, brilliant gold tomentose throughout 95% of T5 reaching with black on hind margin of tergite; ventral surfaces of T3–T5 hirsute but sex-patches present, with light gold tomentum throughout; middorsal depression on ST1+2 reaching to hind margin of tergite; ST1+2 with 1 pair of median marginal setae, 1 pair of median marginal setae present on T3, and complete rows of setae on T4 and T5.

**Male terminalia** (Fig. 91): sternite 5 with a deeply excavated median cleft along posterior edge, roughly Y-shaped, with soft shoulders, margins covered in dense tomentum; posterior lobes rounded apically, with a group of strong erect setulae surrounded by many shorter weaker setulae. Anterior plate of sternite 5 approximately 1/2 length of posterior lobes; unsclerotized "window" on anterior plate of sternite 5 translucent, rectangular. Cerci in posterior view elongate triangular, equal to length of surstyli; pointed at apex, medially to fused along 2/3 of their length. Cerci in lateral view, inflated along basal 2/3rds, sharply tapered with anterior curved at apical 1/3, giving it a shallow hooked appearance; cerci setose along basal 2/3rds, underside of cerci setose along basal 1/2 of length. Surstylus in lateral view, equilateral along its length rounded apically, digitiform; surstylus appearing to be fused with epandrium; when viewed posteriorly surstyli straight. Pregonite broad, well-developed, apically squared off, and blunt, devoid of setulae. Postgonite, slightly narrowed, 1/2 as wide as pregonite, sharply pointed and curved at apex, bladelike, postgonite subequal in length to pregonite. Distiphallus broadly cone-shaped, with a slender median longitudinal sclerotized reinforcement on its posterior surface and a broad, anterolateral, sclerotized acrophallus, bearing a slight anterior hook on anterior surface near apex, 1.2X length of basiphallus.

**Female** (Fig. 92) length: 13–16mm, overall morphology as in male differing in the following traits: **Head**: bearing 1–2 rows of frontal setae and 2–4 pairs of proclinate orbital setae in addition to single pair of reclinate orbital seta; gena 1/3 head height, 2/5 eye height. **Abdomen**: gold tomentum on anterior margin of T4 extending to 20% of surface of tergite; abdomen slightly more globose than males.

#### Diagnosis

*Belvosiarobertoespinozai* **sp. n.** can be distinguished from all other *Belvosia* by the following combination of traits: dark setulae on gena, black basicosta, lacking 4–6 setulae in front of postocular row, postpedicel 1.5X length of pedicel, and tergite 5 black apically. Differs from *B.duniagarciae* by the lack of setulae on postocular margin, and the length of the pedicel.

#### Etymology

*Belvosiarobertoespinozai* **sp. n**, is named in honor of Sr. Roberto Espinoza in recognition of his decades of being part of the Parataxonomist Program of Area de Conservación Guanacaste (http://www.acguanacaste.ac.cr) in northwestern Costa Rica (Janzen and Hallwachs 2011). Interim species-specific name included in previously circulating databases and publications, *Belvosia* Woodley18.

#### Distribution

Costa Rica, ACG, Guanacaste Province, 280–550m elevation.

#### Ecology

*Belvosiarobertoespinozai* **sp. n.** has been reared nine times from two species of Lepidoptera in the family Sphingidae, *Xylophanesjocasta* Druce, 1888 (N=2), and *Xylophanestyndarus* (Boisduval, 1875) (N=7) in rain forest, dry forest, and dry-rain lowland intergrade.

### Belvosia rostermoragai

Fleming & Woodley sp. nov.

4C52D27E-93B5-5115-8C1C-64E5BA4BD742

CDA90233-6078-40CA-B610-C384A0CFCFD8

#### Materials

- **Type status:** Holotype. **Occurrence:** occurrenceDetails: http://janzen.sas.upenn.edu; catalogNumber: DHJPAR0001243; recordedBy: D.H. Janzen, W. Hallwachs & Gloria Sihezar; individualID: DHJPAR0001243; individualCount: 1; sex: Male; lifeStage: adult; preparations: pinned; otherCatalogNumbers: HCIC157-05, 01-SRNP-1141, BOLD:AAF0104; occurrenceID: 7E02DC21-8567-5C86-807C-B61658AE4840; **Taxon:** scientificName: Belvosiarostermoragai; phylum: Arthropoda; class: Insecta; order: Diptera; family: Tachinidae; genus: Belvosia; specificEpithet: rostermoragai; scientificNameAuthorship: Fleming & Woodley, 2023; **Location:** continent: Central America; country: Costa Rica; countryCode: CR; stateProvince: Alajuela; county: Sector San Cristobal; locality: Area de Conservacion Guanacaste; verbatimLocality: Sendero Perdido; verbatimElevation: 620; verbatimLatitude: 10.8794; verbatimLongitude: -85.3861; verbatimCoordinateSystem: Decimal; decimalLatitude: 10.8794; decimalLongitude: -85.3861; **Identification:** identifiedBy: AJ Fleming; dateIdentified: 2022; **Event:** samplingProtocol: Reared from the larvae of the Saturniidae, Rothschildiatriloba; verbatimEventDate: 21-Nov-2001; **Record Level:** language: en; institutionCode: CNC; collectionCode: Insects; basisOfRecord: Pinned Specimen
- **Type status:** Paratype. **Occurrence:** occurrenceDetails: http://janzen.sas.upenn.edu; catalogNumber: DHJPAR0001245; recordedBy: D.H. Janzen, W. Hallwachs & Gloria Sihezar; individualID: DHJPAR0001245; individualCount: 1; sex: Male; lifeStage: adult; preparations: pinned; otherCatalogNumbers: HCIC173-05, 01-SRNP-1141, BOLD:AAF0104; occurrenceID: 873F767C-2288-504A-A987-E2E257ABC09D; **Taxon:** scientificName: Belvosiarostermoragai; phylum: Arthropoda; class: Insecta; order: Diptera; family: Tachinidae; genus: Belvosia; specificEpithet: rostermoragai; scientificNameAuthorship: Fleming & Woodley, 2023; **Location:** continent: Central America; country: Costa Rica; countryCode: CR; stateProvince: Alajuela; county: Sector San Cristobal; locality: Area de Conservacion Guanacaste; verbatimLocality: Sendero Perdido; verbatimElevation: 620; verbatimLatitude: 10.8794; verbatimLongitude: -85.3861; verbatimCoordinateSystem: Decimal; decimalLatitude: 10.8794; decimalLongitude: -85.3861; **Identification:** identifiedBy: AJ Fleming; dateIdentified: 2022; **Event:** samplingProtocol: Reared from the larvae of the Saturniidae, Rothschildiatriloba; verbatimEventDate: 21-Nov-2001; **Record Level:** language: en; institutionCode: CNC; collectionCode: Insects; basisOfRecord: Pinned Specimen

#### Description

**Male** (Fig. 93), length: 12–13mm. **Head**: head wider than thorax; vertex 1/3 head width; gena 1/3 of head height, 1/2 of eye height. Fronto-orbital plate silver tomentose throughout, darkening slightly apically in some cases appearing slightly glabrous apically, with one row of frontal setae, and a second broken row somewhat apparent, and 1 pair of slightly inwardly lateroclinate orbital setae present outside frontal row. Parafacial light yellow in ground color, densely covered in same silver tomentum as on fronto-orbital plate, entire surface reflective and brilliant appearance; almost bare along parafacial outside facial ridge, with several black setulae intermingled with facial ridge setae and extending just below lowest frontal setae; facial ridge setose along 2/3–4/5 of its length; gena covered in black setulae. Antenna, pedicel darkened appearing dark brown or black, overall concolorous with postpedicel covered in a silver sheen; postpedicel dark brown almost black, 2.5X as long as pedicel; arista bare gradually tapering to a point at tip. Palps, orange throughout and densely covered in short black setulae; tapering to a sharp point apically, devoid of setulae apically. Vibrissa approximately 1 pedicel length from facial margin. **Thorax**: black ground color throughout, with gray tomentum throughout; scutellum ground color light brown almost yellow, distinctly lighter than scutum, under microscope bronze tomentum throughout becomes visible; scutum with five dorsal vittae, one outer pair, one inner pair, both broken at suture, and one dorsocentral vitta appearing postsuturally; lateral surface of thorax densely covered in long hair-like setulae, these setulae all black; chaetotaxy: 3–4 strong setae on postpronotum arranged in a line, acrostichal setae 3:3–4; dorsocentral setae 3:4; intra-alar setae 3:3; supra-alar setae 2:3; 4 katepisternal setae; scutellum, with 4–5 pairs of long marginal setae of subequal length; apical scutellar setae short erect, inserted slightly above plane of marginal setae; 1 complete row of scutellar discal setae just posterior to marginal setae. **Wing**: infuscate, slightly darkened yellow/orange at wing base, basicosta dark brown with orange; both upper and lower calypters also infuscate concolorous with remainder of wing; wing vein R_4+5_ setose, bearing only 2–3 setulae at base; halteres orange stalk with dark black/brown capitulum. **Legs**: black overall, lightly covered in shimmering bronze tomentum, posterior margin of coxa on midleg and hindleg covered in yellow setulae; tarsal claws yellow-orange with black tips, with burnt umber pulvilli shorter than length of tarsal claws; anterodorsal row of setae on hind tibia regular and fringelike, with one longer stronger setae at least 2X as long as others. **Abdomen**: large and slightly flattened globose, black to dark burgundy ground color; tomentum absent from T1+2–T4, with a very light almost invisible dusting of bronze tomentum on T5 reaching to hind margin of tergite; ventral surfaces of T3–T5 extremely densely hirsute but with no distinct sex-patches present, with light gold tomentum throughout; middorsal depression on ST1+2 reaching to hind margin of tergite; ST1+2 with 2–4 pairs of median marginal setae, and complete rows of median marginal setae on T3–T5.

**Male terminalia** (Fig. 94): sternite 5 with a deeply excavated median cleft along posterior edge, smoothly U-shaped, margins covered in dense tomentum; posterior lobes rounded apically, with a group of strong setulae surrounded by many shorter weaker setulae. Anterior plate of sternite 5 subequal to length of posterior lobes; unsclerotized "window" on anterior plate of sternite 5 absent. Cerci in posterior view triangular, slightly shorter than surstyli; blunted apex, medially to fused along 1/2 of their length. Cerci in lateral view, anterior curved at apex, giving it a shallow hooked appearance; cerci densely setose along basal 2/3rds, underside of cerci setose along basal 1/2 of length. Surstylus in lateral view, almost broad and equilateral along its length widening slightly slightly at apex structure appear spatulate; surstylus appearing to be fused with epandrium; when viewed posteriorly surstyli slightly convergent. Pregonite broad, well-developed, apically squared off, and blunt, devoid of setulae. Postgonite, slightly narrowed, 1/2 as wide as pregonite, sharply pointed and curved at apex, bladelike, postgonite subequal in length to pregonite. Distiphallus broadly cone-shaped, with a slender median longitudinal sclerotized reinforcement on its posterior surface and a broad, anterolateral, sclerotized acrophallus, on anterior surface near apex, 1.5X length of basiphallus.

**Female**: unknown at this time.

#### Diagnosis

*Belvosiarostermoragai* **sp. n.** can be distinguished from all other *Belvosia* by the following combination of traits: dark setulae below lowest frontal setae, black basicosta, ST1+2 with 2–4 pairs of median marginal setae, and complete rows of median marginal setae on T3–T5, and very light gold tomentum on T5.

#### Etymology

*Belvosiarostermoragai* **sp. n**, is named in honor of Sr. Roster Moraga in recognition of his decades of being part of the Parataxonomist Program of Area de Conservación Guanacaste (http://www.acguanacaste.ac.cr) in northwestern Costa Rica (Janzen and Hallwachs 2011). Interim species-specific name included in previously circulating databases and publications, *Belvosia* Woodley19.

#### Distribution

Costa Rica, ACG, Alajuela Province, 620–700m elevation.

#### Ecology

*Belvosiarostermoragai* **sp. n.** has been reared three times from one species of Lepidoptera in the family Saturniidae, *Rothschildiatriloba* Rothschild, 1907 (N=3) in rain forest.

### Belvosia ruthfrancoae

Fleming & Woodley sp. nov.

4511B959-4621-56A0-89BF-E540A45B5611

E737B17A-B0AB-42F4-906E-87C061B9EE04

#### Materials

- **Type status:** Holotype. **Occurrence:** occurrenceDetails: http://janzen.sas.upenn.edu; catalogNumber: DHJPAR0001249; recordedBy: D.H. Janzen, W. Hallwachs & Jorge Hernandez; individualID: DHJPAR0001249; individualCount: 1; sex: Male; lifeStage: adult; preparations: pinned; otherCatalogNumbers: HCIC108-05, 04-SRNP-16036, BOLD:AAI8614; occurrenceID: BCFD5BDE-3F15-5910-AC1B-A05CC7C85397; **Taxon:** scientificName: Belvosiaruthfrancoae; phylum: Arthropoda; class: Insecta; order: Diptera; family: Tachinidae; genus: Belvosia; specificEpithet: ruthfrancoae; scientificNameAuthorship: Fleming & Woodley, 2023; **Location:** continent: Central America; country: Costa Rica; countryCode: CR; stateProvince: Guanacaste; county: Sector Santa Rosa; locality: Area de Conservacion Guanacaste; verbatimLocality: Area Administrativa; verbatimElevation: 295; verbatimLatitude: 10.8376; verbatimLongitude: -85.6187; verbatimCoordinateSystem: Decimal; decimalLatitude: 10.8376; decimalLongitude: -85.6187; **Identification:** identifiedBy: AJ Fleming; dateIdentified: 2022; **Event:** samplingProtocol: Reared from the larvae of the Notodontidae, Ianassa druceiDHJ04; verbatimEventDate: 01-Jan-2005; **Record Level:** language: en; institutionCode: CNC; collectionCode: Insects; basisOfRecord: Pinned Specimen
- **Type status:** Paratype. **Occurrence:** occurrenceDetails: http://janzen.sas.upenn.edu; catalogNumber: DHJPAR0001250; recordedBy: D.H. Janzen, W. Hallwachs & Ruth Franco; individualID: DHJPAR0001250; individualCount: 1; sex: Female; lifeStage: adult; preparations: pinned; otherCatalogNumbers: HCIC116-05, 04-SRNP-16010, BOLD:AAI8614; occurrenceID: 811FD8BF-F3E1-56E8-BF95-A255AABB6578; **Taxon:** scientificName: Belvosiaruthfrancoae; phylum: Arthropoda; class: Insecta; order: Diptera; family: Tachinidae; genus: Belvosia; specificEpithet: ruthfrancoae; scientificNameAuthorship: Fleming & Woodley, 2023; **Location:** continent: Central America; country: Costa Rica; countryCode: CR; stateProvince: Guanacaste; county: Sector Santa Rosa; locality: Area de Conservacion Guanacaste; verbatimLocality: Area Administrativa; verbatimElevation: 295; verbatimLatitude: 10.8376; verbatimLongitude: -85.6187; verbatimCoordinateSystem: Decimal; decimalLatitude: 10.8376; decimalLongitude: -85.6187; **Identification:** identifiedBy: AJ Fleming; dateIdentified: 2022; **Event:** samplingProtocol: Reared from the larvae of the Notodontidae, Ianassa druceiDHJ04; verbatimEventDate: 02-Jan-2005; **Record Level:** language: en; institutionCode: CNC; collectionCode: Insects; basisOfRecord: Pinned Specimen
- **Type status:** Paratype. **Occurrence:** occurrenceDetails: http://janzen.sas.upenn.edu; catalogNumber: DHJPAR0001248; recordedBy: D.H. Janzen, W. Hallwachs & Jorge Hernandez; individualID: DHJPAR0001248; individualCount: 1; sex: Male; lifeStage: adult; preparations: pinned; otherCatalogNumbers: HCIC197-05, 04-SRNP-16033, BOLD:AAI8614; occurrenceID: 0FFB656C-41C9-5146-B0FC-A331DB68C5D0; **Taxon:** scientificName: Belvosiaruthfrancoae; phylum: Arthropoda; class: Insecta; order: Diptera; family: Tachinidae; genus: Belvosia; specificEpithet: ruthfrancoae; scientificNameAuthorship: Fleming & Woodley, 2023; **Location:** continent: Central America; country: Costa Rica; countryCode: CR; stateProvince: Guanacaste; county: Sector Santa Rosa; locality: Area de Conservacion Guanacaste; verbatimLocality: Area Administrativa; verbatimElevation: 295; verbatimLatitude: 10.8376; verbatimLongitude: -85.6187; verbatimCoordinateSystem: Decimal; decimalLatitude: 10.8376; decimalLongitude: -85.6187; **Identification:** identifiedBy: AJ Fleming; dateIdentified: 2022; **Event:** samplingProtocol: Reared from the larvae of the Notodontidae, Ianassa druceiDHJ04; verbatimEventDate: 01-Jan-2005; **Record Level:** language: en; institutionCode: CNC; collectionCode: Insects; basisOfRecord: Pinned Specimen

#### Description

**Male** (Fig. 95), length: 10mm. **Head**: head slightly wider to thorax; vertex 1/3 head width; gena 1/3 of head height, 1/2 of eye height. Fronto-orbital plate silver tomentose throughout, darkening slightly apically in some cases appearing slightly glabrous apically, with 1–2 rows of frontal setae, and 1 pair of slightly inwardly lateroclinate orbital setae present outside frontal row. Parafacial light yellow in ground color, densely covered in same silver tomentum as on fronto-orbital plate, entire surface reflective and brilliant appearance; almost bare along parafacial outside facial ridge, with several black setulae intermingled with facial ridge setae and extending just below lowest frontal setae; facial ridge setose along 2/3–4/5 of its length; gena covered in black setulae. Antenna, pedicel bright orange appearing, overall in contrast with postpedicel; postpedicel dark brown almost black, 3.5X as long as pedicel; arista bare gradually tapering to a point at tip. Palps, orange throughout and densely covered in short black setulae; tapering to a sharp point apically, devoid of setulae apically. Vibrissa approximately 1 pedicel length from facial margin. **Thorax**: black ground color throughout, with gray tomentum throughout, colorshifting to gold tomentum on posterior half only evident when viewed from posterior angle; scutellum ground color light brown almost yellow, distinctly lighter than scutum, under microscope bronze tomentum throughout becomes visible; scutum with four distinct dorsal vittae, one outer pair, one inner pair, both broken at suture; lateral surface of thorax densely covered in long hair-like setulae, these setulae all black; chaetotaxy: 3–4 strong setae on postpronotum arranged in a line, acrostichal setae 3:3–4; dorsocentral setae 3:4; intra-alar setae 3:3; supra-alar setae 2:3; 4 katepisternal setae; scutellum, with 4–5 pairs of long marginal setae of subequal length; apical scutellar setae short erect, inserted slightly above plane of marginal setae; 1 complete row of scutellar discal setae just posterior to marginal setae. **Wing**: infuscate, slightly darkened gray at wing base, basicosta orange; both upper and lower calypters also infuscate concolorous with remainder of wing; wing vein R_4+5_ setose, bearing only 2–3 setulae at base; halteres orange stalk with dark black/brown capitulum. **Legs**: black overall, lightly covered in shimmering bronze tomentum, posterior margin of coxa on midleg and hindleg covered in yellow setulae; tarsal claws yellow-orange with black tips, with burnt umber pulvilli shorter than length of tarsal claws; anterodorsal row of setae on hind tibia not regular or fringelike, with several longer stronger setae at least 2X as long as others. **Abdomen**: small and rounded globose, black to dark burgundy ground color; tomentum absent from T1+2, with gold tomentum on over 60% of surfaces of both T3 and T4, both with a section of black tomentum along the midline of the tergite, appearing as a black gap between 4 gold patches, T5 entirely gold tomentose; ventral surfaces of T3–T5 extremely densely hirsute but with no distinct sex-patches present, with light gold almost silver tomentum throughout; middorsal depression on ST1+2 reaching to hind margin of tergite; ST1+2 and T3 lacking median marginal setae, and complete rows of median marginal setae on T4–T5.

**Male terminalia** (Fig. 96): sternite 5 with an excavated median cleft along posterior edge, smoothly U-shaped, margins covered in dense tomentum; posterior lobes squared off apically, with strong erect bristle-like setulae surrounded by many shorter weaker setulae. Anterior plate of sternite 5 approximately subequal to length of posterior lobes; unsclerotized "window" on anterior plate of sternite 5 translucent directly basal to posterior lobes, flat basally, with 3 indentations along anterior edge. Cerci in posterior view triangular, short subequal to length of surstyli; separate medially along apical 2/3s of its length. Cerci in lateral view. narrow and appearing rounded apically, straight along lower margin with only a very slight anterior projection, not appearing clubbed apically; cerci setose along basal 2/3rds, underside of cerci bare. Surstylus in lateral view, wide broadly rounded, spatulate or oarlike appearance; surstylus appearing fused with epandrium; when viewed posteriorly surstyli appearing slightly convergent or bearing inward curved apices but not strongly convergent. Pregonite short, not well-developed, apically flat, somewhat blunt, devoid of setulae. Postgonite, short slightly narrowed, 1/3 as wide as pregonite, hooked and sharp at apex. Distiphallus broadly cone-shaped and a broad, anterolateral, sclerotized acrophallus, on anterior surface near apex, 1.4X length of basiphallus.

**Female** (Fig. 97) length: 9–11mm, overall morphology as in male differing in the following traits: **Head**: bearing 1–2 rows of frontal setae and 3–4 pairs of proclinate orbital setae in addition to single pair of reclinate orbital seta. **Abdomen**: abdomen slightly more globose than males.

#### Diagnosis

*Belvosiaruthfrancoae* **sp. n.** can be distinguished from all other *Belvosia* by the following combination of traits: dark setulae below lowest frontal setae, and on gena, orange basicosta, calypters infuscate brown, and median marginal setae absent from ST1+2 and T3.

#### Etymology

*Belvosiaruthfrancoae* **sp. n**, is named in honor of Srta. Ruth Franco, in recognition of her decades of being part of the Parataxonomist Program of Area de Conservación Guanacaste (http://www.acguanacaste.ac.cr) in northwestern Costa Rica (Janzen and Hallwachs 2011). Interim species-specific name included in previously circulating databases and publications, *Belvosia* Woodley20.

#### Distribution

Costa Rica, ACG, Guanacaste Province, 295m elevation.

#### Ecology

*Belvosiaruthfrancoae* **sp. n.** has been reared four times from one species of Lepidoptera in the family Notodontidae, *Ianassadrucei*DHJ04 (N=4) in dry forest.

### Belvosia sergioriosi

Fleming & Woodley sp. nov.

04645514-16C0-51CC-82D7-4C481FF952DE

3A411E04-99BE-4717-AD4C-584EC3BD9575

#### Materials

- **Type status:** Holotype. **Occurrence:** occurrenceDetails: http://janzen.sas.upenn.edu; catalogNumber: DHJPAR0040811; recordedBy: D.H. Janzen, W. Hallwachs & Ricardo Calero; individualID: DHJPAR0040811; individualCount: 1; lifeStage: adult; preparations: pinned; otherCatalogNumbers: ASHYE2977-11, 10-SRNP-72992, BOLD:AAU1116; occurrenceID: 3D347685-8BDE-5E18-BA87-01179E3B1B85; **Taxon:** scientificName: Belvosiasergioriosi; phylum: Arthropoda; class: Insecta; order: Diptera; family: Tachinidae; genus: Belvosia; specificEpithet: sergioriosi; scientificNameAuthorship: Fleming & Woodley, 2023; **Location:** continent: Central America; country: Costa Rica; countryCode: CR; stateProvince: Guanacaste; county: Sector Pitilla; locality: Area de Conservacion Guanacaste; verbatimLocality: Medrano; verbatimElevation: 380; verbatimLatitude: 11.016; verbatimLongitude: -85.3805; verbatimCoordinateSystem: Decimal; decimalLatitude: 11.016; decimalLongitude: -85.3805; **Identification:** identifiedBy: AJ Fleming; dateIdentified: 2022; **Event:** samplingProtocol: Reared from the larvae of the Saturniidae, Pseudodirphiaregia; verbatimEventDate: 14-Jan-2011; **Record Level:** language: en; institutionCode: CNC; collectionCode: Insects; basisOfRecord: Pinned Specimen

#### Description

**Male** (Fig. 98), length: 12mm. **Head**: head slightly wider to thorax; vertex 1/3 head width; gena 1/4 of head height, 1/3 of eye height. Fronto-orbital plate silver tomentose throughout, darkening slightly apically, 2 rows of frontal setae, orbital setae absent. Parafacial light yellow in ground color, densely covered in same silver tomentum as on fronto-orbital plate, entire surface reflective and brilliant appearance; almost bare along parafacial outside facial ridge, with several black setulae intermingled with facial ridge setae and extending just below lowest frontal setae; facial ridge setose along 4/5 of its length; gena covered in black setulae. Antenna, pedicel bright orange appearing, overall in contrast with postpedicel; postpedicel dark brown almost black, 4–5X as long as pedicel; arista bare gradually tapering to a point at tip. Palps, orange throughout and densely covered in short black setulae; tapering to a sharp point apically, devoid of setulae apically. Vibrissa approximately 1 pedicel length from facial margin. **Thorax**: black ground color throughout, with gray tomentum throughout, tomentum receding along posterior edge, postallar callosity with a light vestiture of bronze tomentum only visible on certain angles; scutellum ground color dark reddish-brown, distinctly lighter than scutum, under microscope bronze tomentum throughout becomes visible; scutum with four distinct dorsal vittae, one outer pair, one inner pair, both broken at suture; lateral surface of thorax densely covered in long hair-like setulae, these setulae all black; chaetotaxy: 3–4 strong setae on postpronotum arranged in a line, acrostichal setae 3:3–4; dorsocentral setae 3:4; intra-alar setae 3:3; supra-alar setae 2:3; 4 katepisternal setae; scutellum, with 4–5 pairs of long marginal setae of subequal length; apical scutellar setae short erect, inserted slightly above plane of marginal setae; 1 complete row of scutellar discal setae just posterior to marginal setae, these setae 1/2–2/3 length of scutellar marginals. **Wing**: infuscate, slightly darkened gray at wing base, basicosta brilliant orange; both upper and lower calypters also infuscate concolorous with remainder of wing; wing vein R_4+5_ setose, bearing only 2–3 setulae at base; halteres orange stalk with dark black/brown capitulum. **Legs**: black overall, lightly covered in shimmering bronze tomentum, posterior margin of coxa on midleg and hindleg covered in yellow setulae; tarsal claws yellow-orange with black tips, with burnt umber pulvilli shorter than length of tarsal claws; anterodorsal row of setae on hind tibia regular, fringelike. **Abdomen**: small and rounded globose, orange-brown ground color; gold tomentum absent from T1+2–T4, but present on over 90% of surface of T5; ventral surfaces of T3–T5 extremely densely hirsute with distinct sex-patches present; middorsal depression on ST1+2 reaching to hind margin of tergite; ST1+2 and T3 with one pair of median marginal setae, and complete rows of median marginal setae on T4–T5.

**Male Terminalia**: (Fig. 99) Sternite 5 with a deeply excavated median cleft along posterior edge, smoothly U-shaped, margins with a slight shoulder, covered in dense tomentum; posterior lobes rounded apically, with long bristle-like setulae surrounded by many shorter weaker setulae. Anterior plate of sternite 5 approximately 1/2 length of posterior lobes; unsclerotized "window" on anterior plate of sternite 5 translucent, elongate spanning almost the entire width of the posterior lobes rectangular in shape. Cerci in posterior view triangular, subequal to length of surstyli; separate medially halfway along its length. Cerci in lateral view. wide and appearing rounded apically, straight along lower margin with only a very slight anterior projection, not appearing clubbed apically; cerci setose along basal 2/3rds. Surstylus in lateral view, broadly rounded along its posterior edge giving the structure a blade-like appearance; surstylus appearing fused with epandrium; when viewed posteriorly surstyli appearing straight with no apparent bias. Pregonite broad, well-developed, apically rounded, somewhat blunt, devoid of setulae. Postgonite, narrower than pregonite, rounded with a slight curve at apex. Distiphallus broadly cone-shaped, with a slender median longitudinal sclerotized reinforcement on its posterior surface and a broad, anterolateral, sclerotized acrophallus, on anterior surface near apex, ~1.9X as long as basiphallus.

**Female**: unknown at this time.

#### Diagnosis

*Belvosiasergioriosi* **sp. n.** can be distinguished from all other *Belvosia* by the following combination of traits: gena covered in black setulae, orange basicosta, median marginal setae present on ST1+2, and T4 lacking any gold tomentum. It can be differenciated from its closest congener *B.naccina* by the color of the arista, and the evenly infuscate wings.

#### Etymology

*Belvosiasergioriosi* **sp. n**, is named in honor of Sr. Sergio Rios in recognition of his decades of being part of the Parataxonomist Program of Area de Conservación Guanacaste (http://www.acguanacaste.ac.cr) in northwestern Costa Rica (Janzen and Hallwachs 2011). Interim species-specific name included in previously circulating databases and publications, *Belvosia* Woodley21.

#### Distribution

Costa Rica, ACG, Guanacaste Province, 380m elevation.

#### Ecology

*Belvosiasergioriosi* **sp. n.** has been reared once from one species of Lepidoptera in the family Saturniidae, *Pseudodirphiaregia* Draudt, 1930 (N=1) in rain forest.

## Identification Keys

### Key to the males of *Belvosia* Robineau-Desvoidy of North- and Meso-America

**Table:** 

1	Abdomen to naked eye appearing black when viewed from above, if tomentum present, bronze or brown but not gold, densely covered in strong abdominal setae, resembling *Leschenaultia* Robineau-Desvoidy in general appearance (Figs 76, 93); 3–5+ pairs of marginal setae on both ST1+2 and T3	2
–	Abdomen either black with contrasting tomentose bands more striking, “typical” *Belvosia*, or with a more ochraceous ground color; not entirely black. Setation of abdomen restricted to marginal setae (Figs 19, 61)	3
2	Tergite 5 when viewed under certain angles of light, completely dull golden tomentose; basicosta yellow; setulae of genal dilation and pleura mostly pale; species only known from 1000m elevation and above	*B.minorcarmonai* **sp. n.**
–	Tergite 5 black, shiny, with very inconspicuous and very sparse tomentum; basicosta mostly black; setulae of genal dilation and pleura black	*B.rostermoragai* **sp. n.**
3	Abdominal bands of T4 and T5 both with deep orange to brick red tomentum	4
–	Abdominal bands of T4 and T5, when present either with bronze-gold or white tomentum	5
4	Pollen of head brown except facial ridges and a spot on parafacial, where it is silvery	*B.vanderwulpi* Williston
–	Pollen of head silvery white, palpus dark brown almost black, facial ridge with setulae extending almost along entire length, pedicel and postpedicel concolorous black	*B.ferruginosa* Townsend
5	Basicosta brilliant yellow-red/orange; general appearance variable, but frequently not black with yellowish tomentose bands	6
–	Basicosta partly black/dark brown; often characterized as large black flies with yellow tomentose bands on abdomen	28
6	Abdomen with light ground color (yellow-orange), occasionally this orange only apparent when viewed laterally especially in photos; often male wth proclinate orbital setae present	7
–	Abdomen with dark ground color (can be dark yellow-orange appearing black to the naked eye), either with or without a narrow black median stripe or indistinct stripe created by tomentum; males without proclinate orbital setae	11
7	Dorsum of thorax gray tomentose	8
–	Dorsum of thorax ranging from entirely bronze to gold tomentose concolorous with T3–T5	10
8	Abdomen entirely light orange ground color, postocular margins gold tomentose	*B.ricardocaleroi* **sp. n.**
–	Abdomen light orange ground color, darkened dorsally; postocular margins silver–gray tomentose	9
9	Postpedicel orange concolorous with pedicel; wing orange infuscate basally; T3 with light dusting of gold tomentum medially bisected by a thick stripe extending through T4	*B.equinoctalis* (Townsend)
–	Postpedicel dark with orange basally, juxtaposed against a yellow pedicel; wing brown-yellow infuscate basally; T3 with gold tomentum only along anterior margin, stripe bisecting tomentosity almost indistinct	*B.obesula* (Wulp)
10	Four pairs of scutellar marginal setae; calypters orange translucent; middorsal abdominal stripe occluded by bronze tomentum of abdomen	*B.mira* Reinhard
–	Three pairs of scutellar marginal setae; calypters pale yellow translucent; middorsal abdominal stripe visible through gold tomentum of abdomen	*B.ochriventris* (Wulp)
11	Median marginal setae extremely weak–absent from Syntergite 1+2 (ST1+2)	12
–	Median marginal setae present on ST1+2	17
12	Both calypters white translucent	13
–	Both calypters heavily infuscate reddish/brown (From ACG, parasitizing Notodontidae exclusively)	14
13	Fronto-orbital plate, parafacial and thorax silver tomentose, calypters white translucent throughout	*B.omissa* Aldrich
–	Fronto-orbital plate, parafacial and thorax gold tomentose, calypters white on edges with brown centrally	*B.manuelriosi* **sp. n.**
14	Median marginal setae absent from tergite 3; fronto-orbital plate with setulae extending well below lower margin of pedicel	*B.ruthfrancoae* **sp. n.**
–	Median marginal setae present on tergite 3, at most 3–4 fronto-orbital setulae present below lower margin of pedicel	15
15	Dorsal surfaces of scutum tawny tomentose, transitioning to brown postsuturally; pedicel orange constrasting with postpedicel	*B.matamorosa* Reinhard
–	Dorsal surfaces of scutum entirely silver tomentose; pedicel brown concolorous with postpedicel	16
16	T4 with gold tomentum only on anterior 60% of tergite with a middorsal stripe bisecting tomentosity	*B.manuelpereirai* **sp. n.**
–	T4 and T5 entirely brilliant gold tomentose	*B.recticornis* Macquart
17	Pilosity of gena, anepisternum, katepisternum completely dark	18
–	Pilosity of gena, anepisternum, katepisternum partly pale, particularly posterior to row of major setae along posterior margin	23
18	Tergite 4 completely devoid of any gold tomentum	19
–	Tergite 4 bearing at least 10% gold/bronze tomentum	20
19	Arista orange, abdomen black ground color throughout, wings orange basally	*B.naccina* Reinhard
–	Arista black, abdomen orange-brown ground color appearing black on T3, wings evenly infuscate throughout	*B.sergioriosi* **sp. n.**
20	Median Marginal Setae (MMST) ST1+2 weak, but distinctly present ; T5 gold tomentose with a very sparse and randomly spaced vestiture of short black setulae present lateroventrally	*B.carolinacanoae* **sp. n.**
–	MMST ST1+2, distinctly present and strong; T5 gold tomentose with a randomly spaced vestiture of short black setulae present on all surfaces	21
21	Anterior margin of T3 devoid of gold tomentum; gold tomentum on T4 90% coverage of tergite, only part not tomentose is a narrow band adjancent to marginal setae, tergal band complete with no longitudinal stripe, or if stripe apparent then only as a slight darkening less than thickness of one median marginal seta	*B.ansata* Reinhard
–	Anterior margin of T3 bearing some minor gold tomentum <10%; gold tomentum on T4 ranging from 20–40% coverage of tergite, tergal band bissected medially by a dark longitudinal stripe	22
22	Gold tomentum on T5 covering entire tergite inclusive of tergal margin; epandrium orange	*B.ciliata* Aldrich
–	Gold tomentum on T5 interrupted along dorsomedial apex, replaced with a small but presente darkened spot; epandrium black,	*B.anacarballoae* **sp. n.**
23	Fronto-orbital plate, appearing glabrous yellow; both calypters pale white translucent	24
–	Fronto-orbital plate, not as above, ranging from brilliant gold to dull gray tomentose	25
24	Gena concolorous with fronto-orbital plate usually appearing glabrous yellow; calypters white overall; abdominal tergite 5 completely orangish yellow with similarly colored tomentum that contrasts with tomentum of other segments; majority of hair-like setulae on genal dilation black; wings only slightly infuscate	*B.unifasciata* (Robineau-Desvoidy)
–	Gena brilliant silver tomentose with fronto-orbital plate usually appearing glabrous yellow; calypters white with yellow-orange fringe; abdominal tergite 5 black in ground color, with pale tomentum that is similar to tomentum of other segments; tomentum sparse to absent on posterior margin so that it appears black; majority of hair-like setulae on genal dilation pale; wings dark brown infuscate	*B.slossonae* Coquillett
25	Katepisternum, meron, and anepimeron bearing only long pale setulae; in males fronto-orbital plate and up to 50% of parafacial gold tomentose; females dull gray tomentum on fronto-orbital plate, and silver parafacial; female palpus apically clubbed and covered in a sparse vestiture of setulae	*B.brigittevilchezae* **sp. n.**
–	Katepisternum, meron, and anepimeron with mostly black-brown setulae with few long pale setulae interspersed; both males and females without gold tomentum on either fronto-orbital plate or parafacial (sometimes tomentum can be thin so as to make the head appear yellow but distinctly not gold tomentose)	26
26	T4 almost devoid of gold tomentum with a narrow fringe of bronze-gold tomentum apparent only along anterior margin of tergite	*B.angelhernandezi* **sp. n.**
–	T4 with gold tomentum covering over 50% of tergite, broken medially by narrow black band	27
27	Setulae below lowest frontal seta dark; four postsutural acrostichals	*B.adrianguadamuzi* **sp. n.**
–	Setulae below lowest frontal seta pale yellow; three postsutural acrostichals	*B.calixtomoragai* **sp. n.**
28	Abdominal tergite 5 entirely gold or white tomentose, sometimes with at most a vague dark stripe dorsally	29
–	Abdominal tergite 5 with tomentum posterior to large marginal setae absent, thus appearing black apically	32
29	Abdomial tergite 4 entirely black, devoid of gold tomentum	*B.desita* (Walker)
–	Abdomial tergite 4 gold tomentose	30
30	Gold tomentum on T4 covering entire tergite, not bisected medially by a dark strip so that both T4 and T5 are uninterrupted gold tomentose	*B.semiflava* Aldrich
–	Gold tomentum on T4 covering at most 50% of tergite, bisected medially by a dark strip so that two distinct tomentose patches on T4 appear separated from T5	31
31	Abdomen slightly flattened more like the 'regular' Belvosia, T5 slightly open vaguely exposing the genital capsule; T5 entirely gold with a slight blackening around median pair of marginal setae; fronto-orbital plate with a pale gold bronze tomentum	*B.canalis* Aldrich
–	Abdomen rounded globose, T5 with only a slit operculum enclosing the genital capsule; T5 entirely gold lacking any black around median pair of marginal setae; fronto-orbital plate with silver-gray	*B.osvaldoespinozai* **sp. n.**
32	Both calypters appearing whitish with a pale fringe of setulae	33
–	At least upper calypter, and frequently both, dark, often with a dark fringe	35
33	Anterodorsal row of setae on hind tibia fringelike, formed by a very regular row of uniformly sized setae separated from each other by less than the width of their sockets; antennae short, postpedicel about 2X as long as pedicel; all major abdominal setae rather strongly appressed and directed posteriorly; MMST absent from ST1+2 and reduced frequently absent on ST3	*B.gloriasihezarae* **sp. n.**
–	Anterodorsal row of setae on hind tibia irregular and not fringelike, usually with several median setae that are distinctly longer than others; antennae, postpedicel greater than 2X as long as pedicel; all major abdominal setae not strongly appressed; often MMST present both ST1+2 and present on ST3	34
34	Setae on facial ridge weak, each seta less than length of antennal pedicel; anepimeron with some obviously pale setulae, especially below and posterior to major anipemeral setae; tomentum on male fronto-orbital plate yellow-gold	*B.townsendi* Aldrich
–	Setae on facial ridge strong, each seta as long as or longer than length of antennal pedicel; anepimeron with entirely dark setulae, rarely with a few pale setulae; tomentum on male fronto-orbital plate silvery-white	*B.argentifrons* Aldrich
35	Lower calypter pale in color, contrasting to the upper calypter strongly infuscate	36
–	Lower calypter dark, concolorous with upper calypter	37
36	Golden tomentum reaching hind margin of abdominal tergite 4 laterally and ventrally, so the hind margin does not appear to have a uniformly dark band; surstylus nearly straight, not distinctly arcuate	*B.canadensis* Curran
–	Golden tomentum not reaching hind margin of abdominal tergite 4 laterally and ventrally, so the hind margin appears to have a uniformly dark band; surstylus distinctly arcuate anteriorly	*B.bifasciata* (Fabricius)
37	Post sutural surface of scutum displaying mostly brassy brown tomentum, concolorous with tomentosity of scutellum (visible under certain angles of light), or glabrous devoid of tomentosity	38
–	Post sutural surface of scutum displaying mostly silver tomentum, contrasting with tomentosity of scutellum (visible under certain angles of light), if any brassy tomentum present then this confined to postalar callus	40
38	Thorax appearing glabrous and devoid of tomentum	*B.splendens* Curran
–	Thorax tomentose	38
39	Palpus apically orange, darkened basally	*B.eldaarayae* **sp. n.**
–	Palpus brown throughout	*B.auratilis* Reinhard.
40	With at most most a narrow fringe of gold on T4, occupying less than 10% of tergite, males most often lacking gold tomentum on T4 entirely; postpedicel only up to maximum 2X as long as pedicel	41
–	T4 with gold tomentum covering at least 10% or more of tergite either as a solid unbroken band or bisected medially by darker brassy-brown tomentum; postpedicel variable length	44
41	Median marginal setae on ST1+2 reduced, if female then median marginal setae absent from tergite; anterodorsal setae on hind tibia regular and comblike, typically at most 1.25X as long as width of supporting tibia, each seta separated from the other with regular spacing no more than the width of the base of the preceding seta	*B.freddyquesadai* **sp. n.**
–	Both males and females with strong median marginal setae on ST1+2; anterodorsal setae on hind tibia irregular, not comblike, many seta exceeding 1.25X as long as width of supporting tibia, setae irregularly spaced	42
42	Pedicel orange to reddish brown, contrasting the dark blackened color of the postpedicel; postpedicel greater than 2X length of pedicel	*B.petronariosae* **sp. n.**
–	Pedicel black to dark brown, not contrasting the dark blackened color of the postpedicel; postpedicel at most 2X length of pedicel	43
43	Gena 1/3 length of eye; postpedicel 2X length of pedicel; inner row of 5-10 setae anterior to postocular setae; T5 with a sparse vestiture of setulae on dorsal and ventral surfaces	*B.duniagarciae* **sp. n.**
–	Gena 1/2 length of eye; postpedicel 1.5X length of pedicel; lacking an inner row of 5-10 setae anterior to postocular setae; T5 devoid of any setulae along dorsal surface outside of those surrounding tergal marginal setae	*B.robertoespinozai* **sp. n.**
44	Parafacial setulae yellow	*B.pabloumanai* **sp. n.**
–	Parafacial setulae dark	45
45	Anterodorsal setae on hind tibia irregular, not comblike, many seta exceeding 1.25X as long as width of supporting tibia, setae irregularly spaced	46
–	Anterodorsal setae on hind tibia regular and comblike, typically at most 1.25X as long as width of supporting tibia, each seta separated from the other with regular spacing no more than the width of the base of the preceding seta	53
46	Palpus dark umber brown throughout	*B.borealis* Aldrich
–	Palpus with at least partial yellow-orange	47
47	Gold tomentosity on T4 occupying over 60% of tergite, typically with only a narrow margin of bare tergite visible surrounding the marginal setae	48
–	Gold tomentosity on T4 reduced, occupying up to maximum 50% of tergite, often males with T4 mostly black	51
48	Postpedicel short, less than 1.5 times length of pedicel (typically almost equal to length of pedicel)	*B.bicincta* Robineau-Desvoidy
–	Postpedicel longer, more than 2X times length of pedicel (typically 3–4X)	49
49	Cercus narrow and parallel sided, apically beaked with a small indentation apically, slight swelling directly adjacent to this indentation, surstylus narrow, parallel sided, digitiform (Fig. 41a); females with rounded end to palpus with setulae extending apically; specialists reared only from Sphingidae	*B.eliethcantillanoae* **sp. n.**
–	Cercus not as descirbed above, surstylus oar-shaped, either with a slight pinch medially along ventral edge or a straight (Figs 67a, 70a); females: palpus more sharply pointed and bare apically; specialist feeding only on a variety of Saturniidae; specialist reared only from Saturniidae	50
50	Fronto-orbital plate silver with varying tonality of gold tomentum (ranging from very yellow-gold to silver with brassy tones), darkening slightly apically in some cases appearing glabrous or void of tomentum apically, in females uniformly silver gray with darkened area much larger and shinier; surstylus shorter than cercus, inversely tapered, spatulate, apically rounded with more curvature along upper edge; female antenna short of facial margin by 1.5X length of pedicel, underside of T3 silver tomentose along anterior margin; specialist feeding only on Saturniidae (Eacles sp.)	*B.keinoraragoni* **sp. n.**
–	Fronto-orbital plate silver tomentose, darkening apically in some cases appearing glabrous or void of tomentum apically, in females darkened area much larger and shinier; surstylus subequal in length to cercus, parallel-sided, apically rounded; female antenna short of facial margin by 1X length of pedicel, entire surface of T3 uniformly lightly brown rusty tomentose including underside (apparent under certain angles of light); specialist feeding only on Saturniidae (Citheronia sp.)	*B.luciariosae* **sp. n.**
51	Setae at base of scutum strong and irregularly spaced not appearing as a regularly formed marginal row; males with postpedicel short 2/5X as long as pedicel; abdominal tergite T3 with devoid of gold tomentum directly adjacent to ST1+2	*B.duvalierbricenoi* **sp. n.**
–	Setae at base of scutum strong and regularly spaced appearing as a regularly formed marginal row; males with postpedicel long 1/4–1/3 as long as pedicel;abdominal tergite T3 with traces of gold tomentum directly adjacent to ST1+2	52
52	Male, fronto-orbital plate gold; at most 1–2 small setae anterior to postocular row; gold wrapping around T4 extending to underside; cerci when viewed dorsally, regularly tapered with no distinct triangle apically, when viewed laterally only vaguely hirsute, surstylus slightly widened basally with a slight curvature; lobes of ST5 2.4x as long as basal section; female, with at most a row of 3–5 small setae anterior to postocular row	*B.diniamartinezae* **sp. n.**
–	Male, fronto-orbital plate mostly gray only with hints of gold present; row of 5–7 small setae anterior to postocular row; gold wrapping around T4 extending 3/4 around tergite not reaching underside; cerci when viewed dorsally, basal 3/5 widened, apical 2/5 equilaterally triangular and pointed, when viewed laterally strongly hirsute basally, surstylus equilaterally sided and straight with no distict curvature; lobes of ST5 1.75x as long as basal section; female, with row of 5–8 small setae anterior to postocular row	*B.ciriloumanai* **sp. n.**
53	Unsclerotized "window" at base of ST5 vaguely rectangular/ovoid with no distinct curvature at apices as in Figs 59c, 65c; when viewed laterally surstylus thickening at midpoint, basally thickened	54
–	Unsclerotized "window" at base of ST5 vaguely curved at apices	55
54	When viewed posteriorly cerci form a sharply pointed wide base triangle, with sides curving slightly inward, length to tips 1.3X basal width	*B.joseperezi* **sp. n.**
–	When viewed posteriorly cerci form a sharply pointed triangle with a narrow base, sides straight, length to tips 1.8X basal width	*B.jorgehernandezi* **sp. n.**
55	Surstylus when viewed laterally rounded at apices	56
–	Surstylus when viewed laterally pointed at apices	57
56	Epandrium and hypandrium not densely hirsute; when viewed laterally surstylus subequal in legth to cerci; surstylus apically rounded with a slight anterior curve along inferior edge, giving the process a digitiform appearance	*B.guillermopererai* **sp. n.**
–	Epandrium and hypandrium densely hirsute; when viewed laterally surstylus distinctly shorter than cerci; cerci apically rounded flat along inferior edge, giving the process a dull blade like appearance	*B.harryramirezi* **sp. n.**
57	Cerci when viewed laterally with posterior edge evidently straight ending in a curved tip; surstylus flat along anterior edge, posterior edge curved, as in an inverted straight back blade shape	*B.hazelcambroneroae* **sp. n.**
–	Cerci when viewed laterally with posterior edge rounded ending in a curved tip; surstylus angled upwards from anterior edge, upward edge curved, giving it a spear type point	*B.josecortezi* **sp. n.**

This key was written based on characters present in both males and females, in some rare cases where sexual dimorphism is present the species have been divided into parts and the sexes indicated. The geographic boundaries include Canada, south to the Panama border with Colombia. This key illustrates the identifying characters states for 33 new species from Area de Conservación Guanacaste along with 25 previously described species from North- and Meso-America.

## Discussion

**Figure 100.**
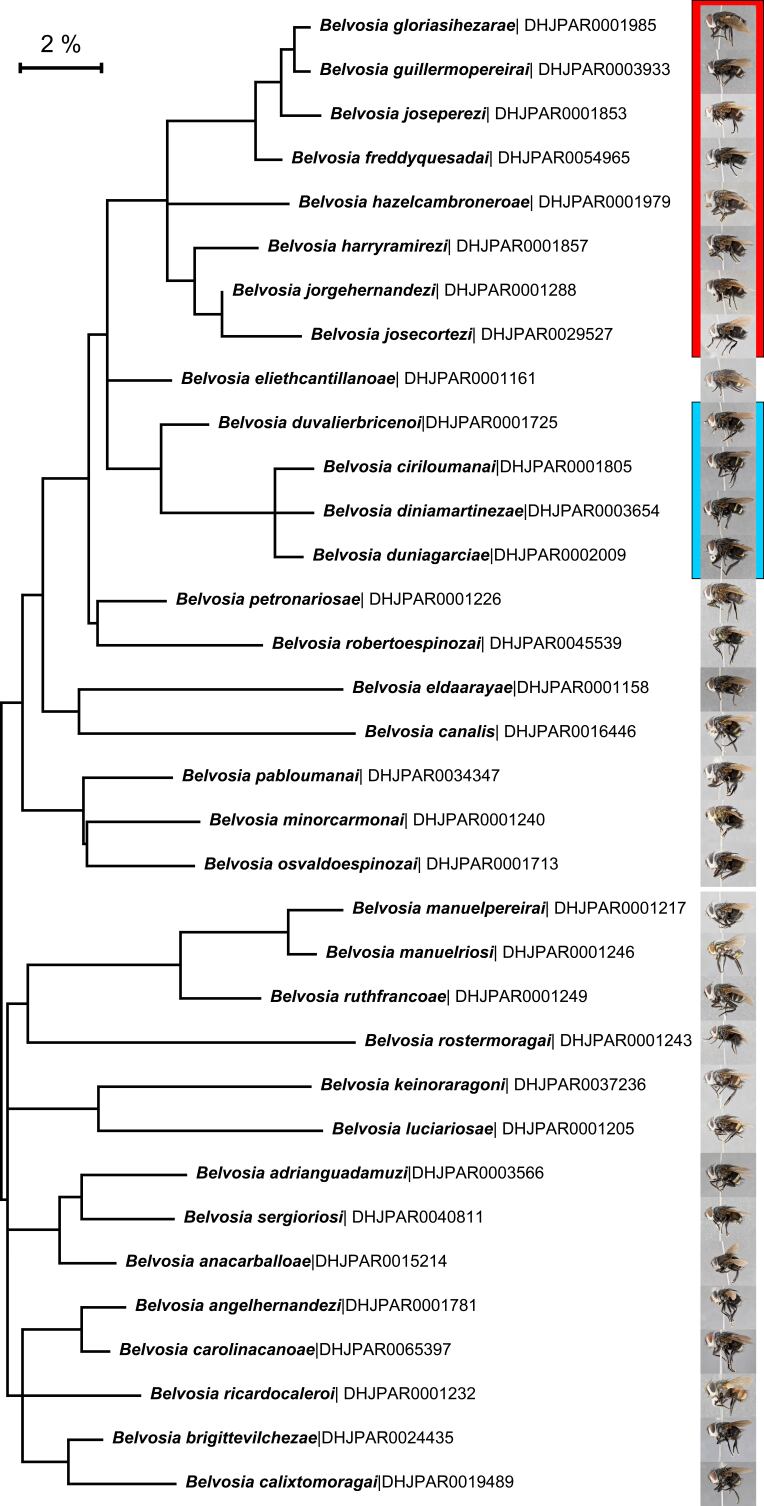
An unrooted phylogenetic tree for 34 species of ACG *Belvosia* inferred by using the Maximum Likelihood (ML) method based on the General Time Reversible model (Nei and Kumar 2000) conducted in MEGA X (Kumar et al. 2018). Tip labels are species names and the DHJPAR accession for the holotype and are associated with a lateral image of the holotype (except *B.canalis*). The red box contains the species discussed in the text of the *B.freddyquesadai*species complex,"Woodley07 complex" cited in Smith et al. (2006) while the blue box contains the species included in the Woodley04 complex cited in Smith et al. (2006).

A phylogenetic tree based on DNA barcodes was used to visually demonstrate the variation within and between species, and is presented in Fig. 100. Interested readers can consult the Barcode of Life Data System (BOLD) for all information associated with each sequence (including GenBank accession numbers), derived from each individual specimen using the persistent DOI:

## Supplementary Material

XML Treatment for Belvosia

XML Treatment for Belvosia adrianguadamuzi

XML Treatment for Belvosia anacarballoae

XML Treatment for Belvosia angelhernandezi

XML Treatment for Belvosia brigittevilchezae

XML Treatment for Belvosia calixtomoragai

XML Treatment for Belvosia canalis

XML Treatment for Belvosia carolinacanoae

XML Treatment for Belvosia ciriloumanai

XML Treatment for Belvosia diniamartinezae

XML Treatment for Belvosia duniagarciae

XML Treatment for Belvosia duvalierbricenoi

XML Treatment for Belvosia eldaarayae

XML Treatment for Belvosia eliethcantillanoae

XML Treatment for Belvosia freddyquesadai

XML Treatment for Belvosia gloriasihezarae

XML Treatment for Belvosia guillermopereirai

XML Treatment for Belvosia harryramirezi

XML Treatment for Belvosia hazelcambroneroae

XML Treatment for Belvosia jorgehernandezi

XML Treatment for Belvosia josecortezi

XML Treatment for Belvosia joseperezi

XML Treatment for Belvosia keinoraragoni

XML Treatment for Belvosia luciariosae

XML Treatment for Belvosia manuelpereirai

XML Treatment for Belvosia manuelriosi

XML Treatment for Belvosia minorcarmonai

XML Treatment for Belvosia osvaldoespinozai

XML Treatment for Belvosia pabloumanai

XML Treatment for Belvosia petronariosae

XML Treatment for Belvosia ricardocaleroi

XML Treatment for Belvosia robertoespinozai

XML Treatment for Belvosia rostermoragai

XML Treatment for Belvosia ruthfrancoae

XML Treatment for Belvosia sergioriosi

5C848049-5527-5B62-AEDE-FD00CE2685E9 10.3897/BDJ.11.e103667.suppl1 Supplementary material 1 All belvosia occurences ACG Data type occurences Brief description Due to the overwhelming size the cumulative dataset of all the records collected for the present work was published separately through GBIF (DOI) as well as attached herein as a supplement. File: oo_807707.xls https://binary.pensoft.net/file/807707 AJ Fleming

## Figures and Tables

**Figure 1a.**
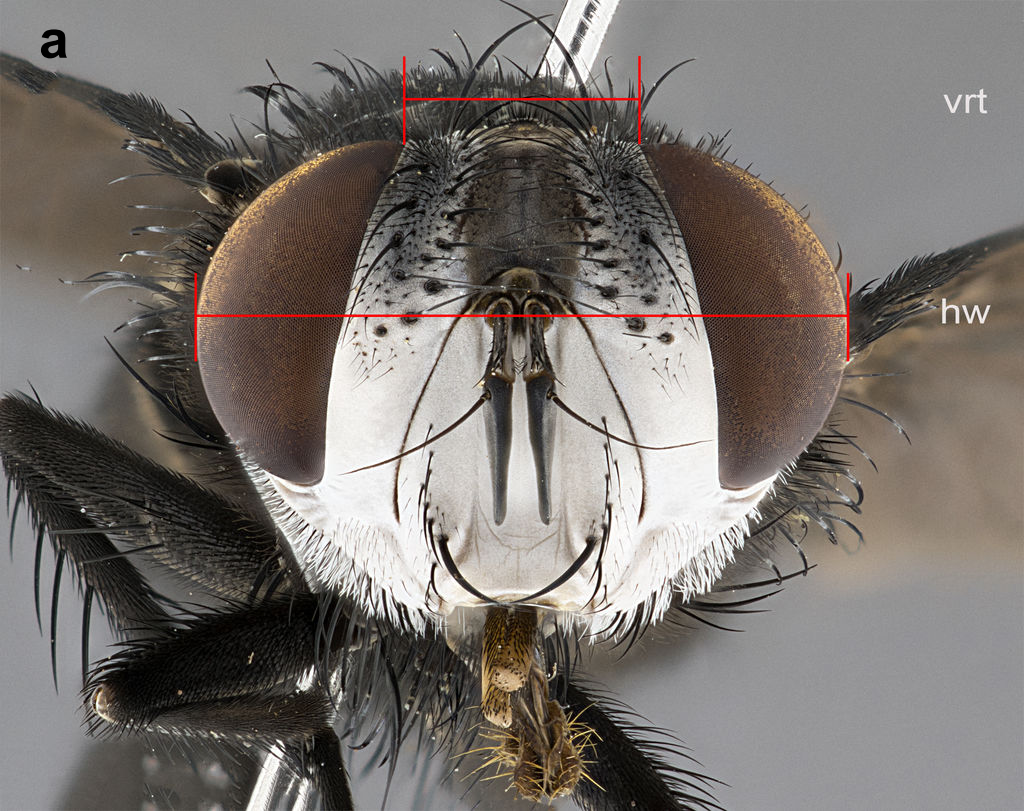
measured areas from front of head of female paratype of *Belvosiaciriloumanai* **sp. n.**, adtitionally of note is the contrasting dark setulae on the gena. Abbreviations: vrt, vertex; hw, head width.

**Figure 1b.**
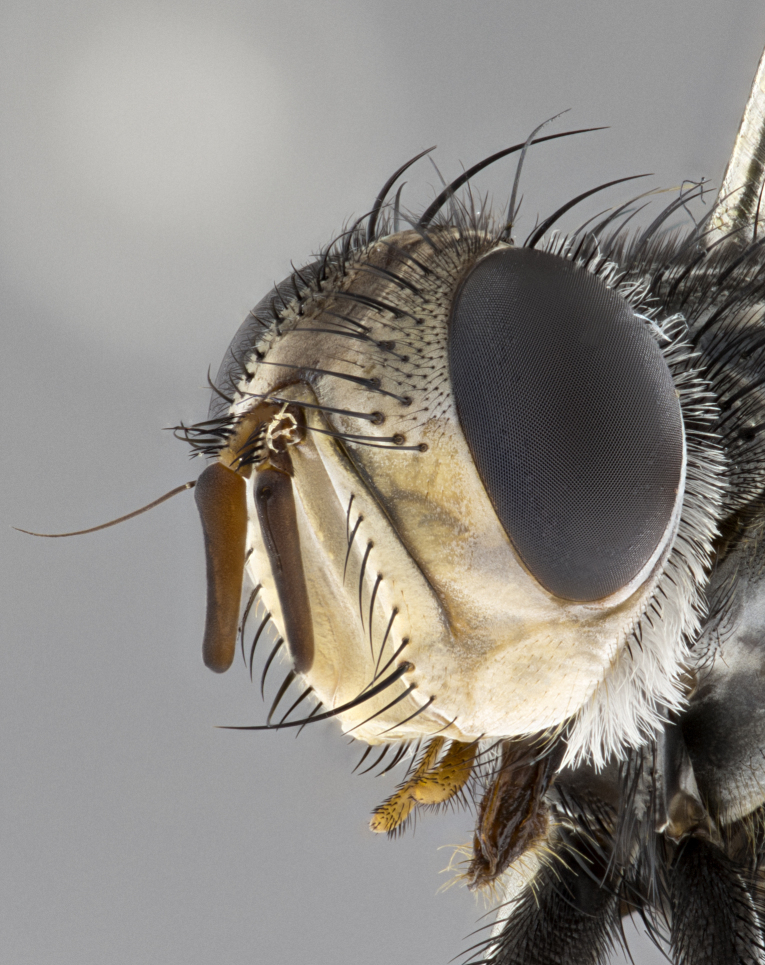
3/4 view of the head of male *Belvosiaangelhernandezi* **sp. n.**, note the light colored setulae of the gena

**Figure 1c.**
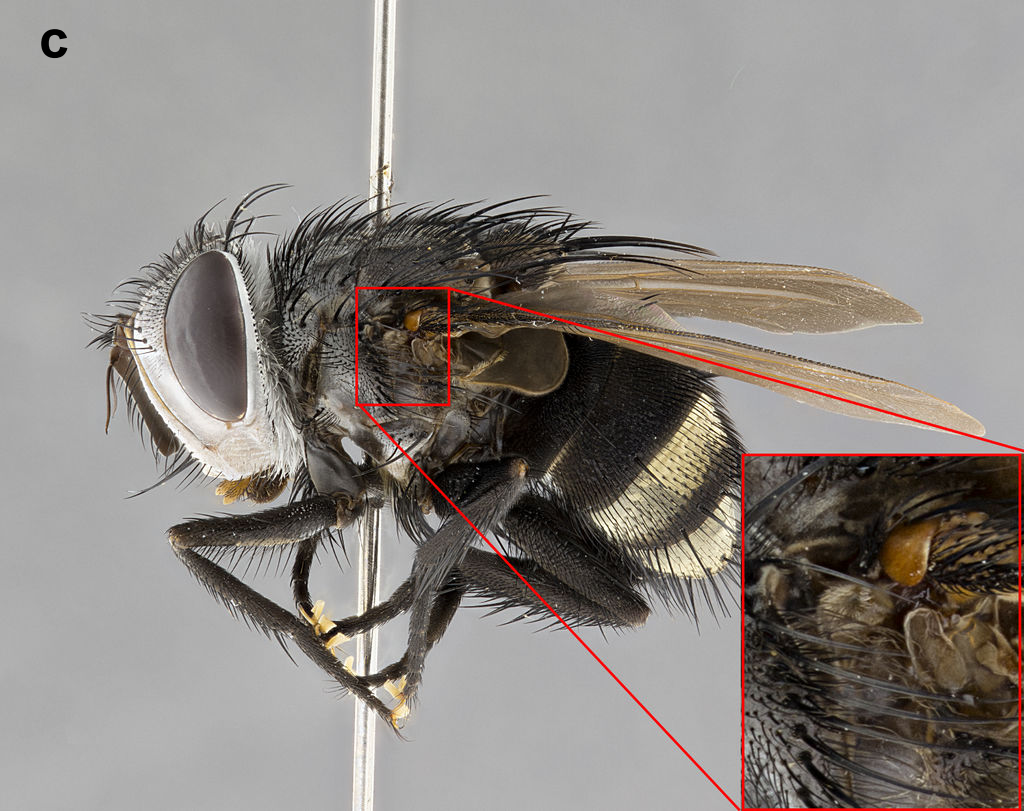
lateral habitus of male holotype *Belvosiaadrianguadamuzi* **sp. n.**, inset detailing the orange basicosta

**Figure 1d.**
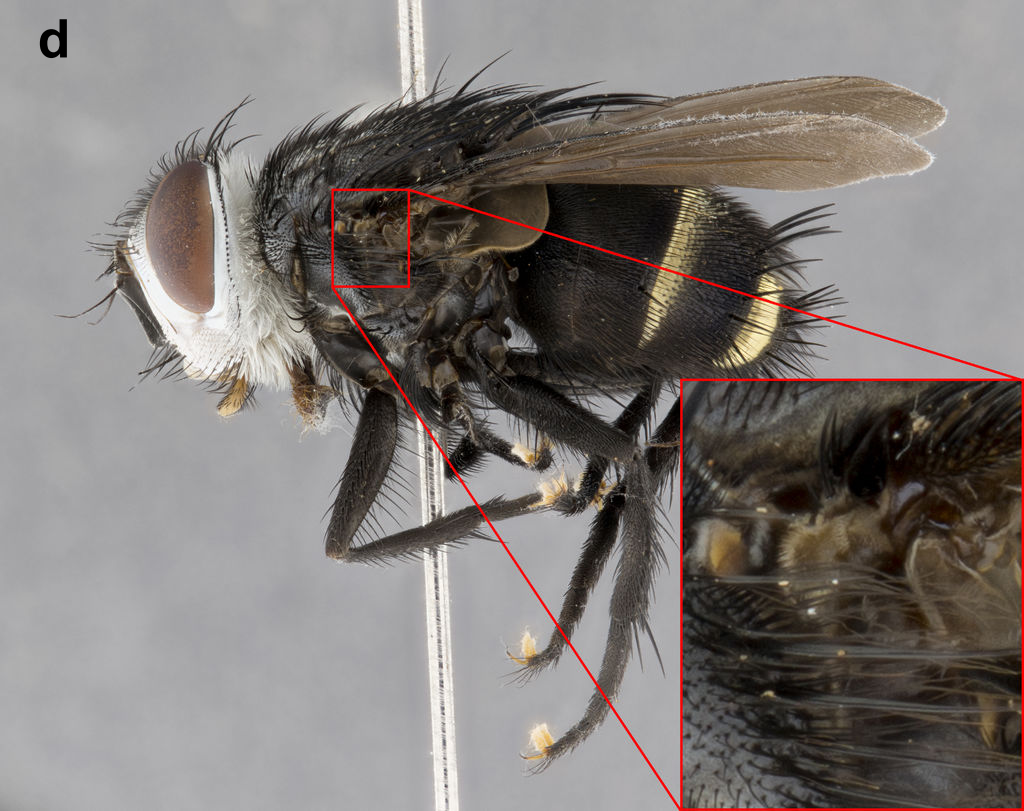
lateral habitus of male holotype *Belvosiaciriloumanai* **sp. n.**, inset detailing the black basicosta

**Figure 2a.**
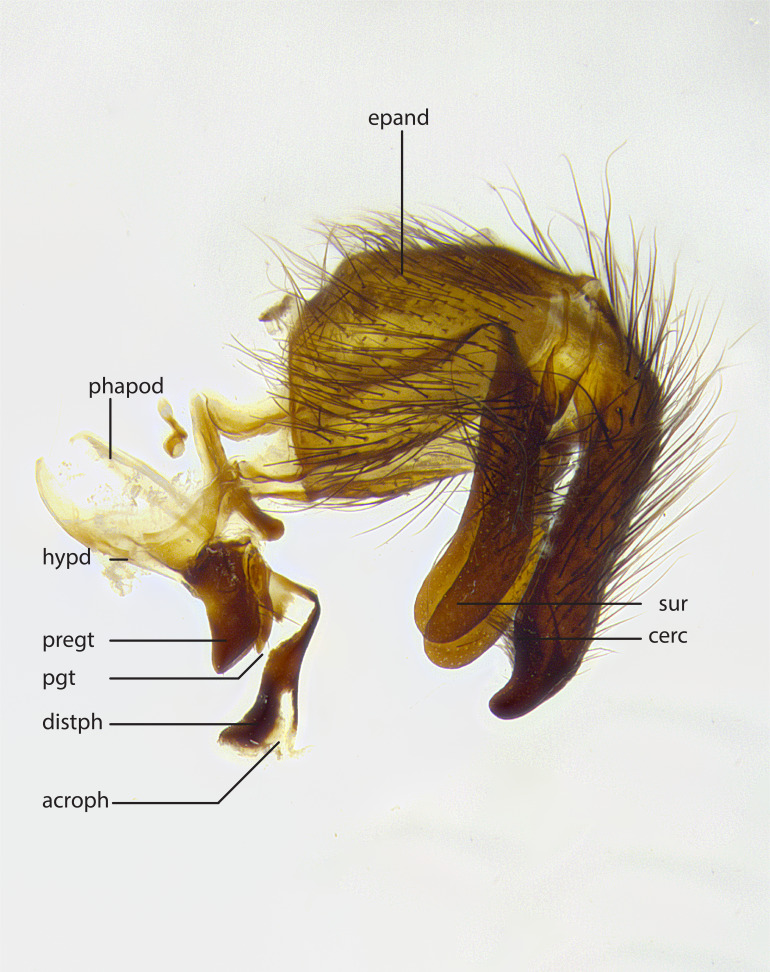
abbreviations: acroph = acrophallus; basiph = basiphallus; cerc = cercus; distph = distiphallus; epand = epandrium; hypd = hypandrium; phapod = phalloapodeme; pgt = postgonite; pregt = pregonite; sur = surstylus.

**Figure 2b.**
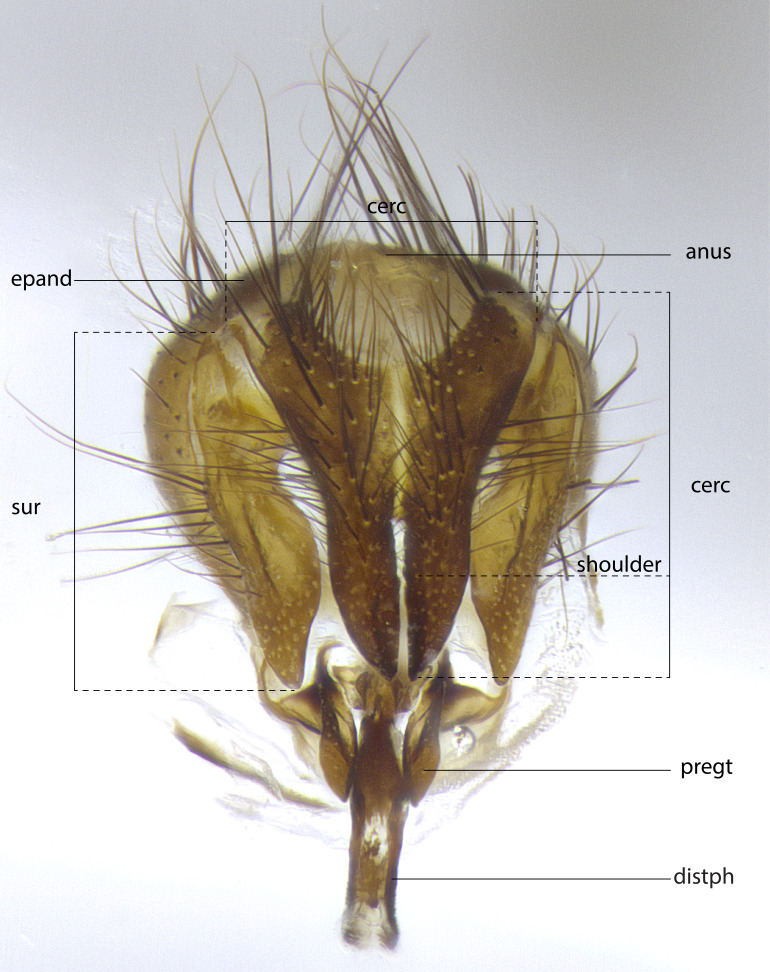
abbreviations: anus = anal operculum; distph = distiphallus; epand = epandrium; shoulder = shoulder point on cercus; pregt = pregonite; sur = surstylus.

**Figure 2c.**
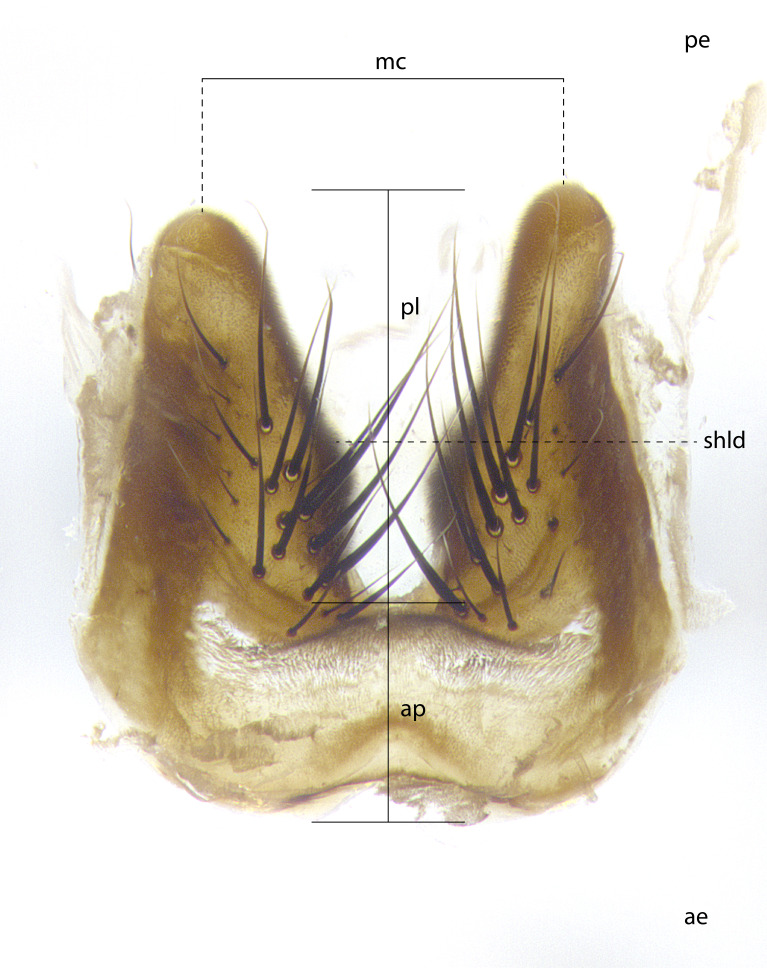
abbreviations: ae = anterior edge; ap = anterior plate; mc = median cleft; shld = shoulder of posterior lobes; pe = posterior edge; pl = posterior lobes.

**Figure 4a.**
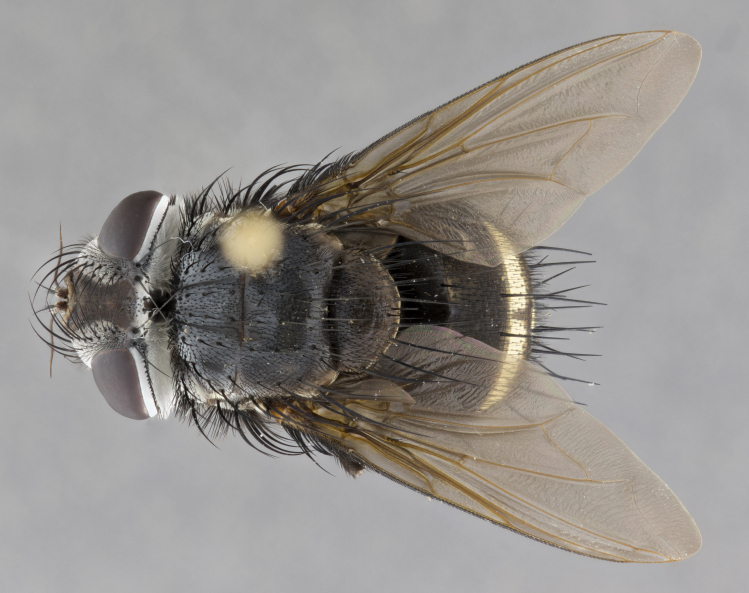
dorsal view

**Figure 4b.**
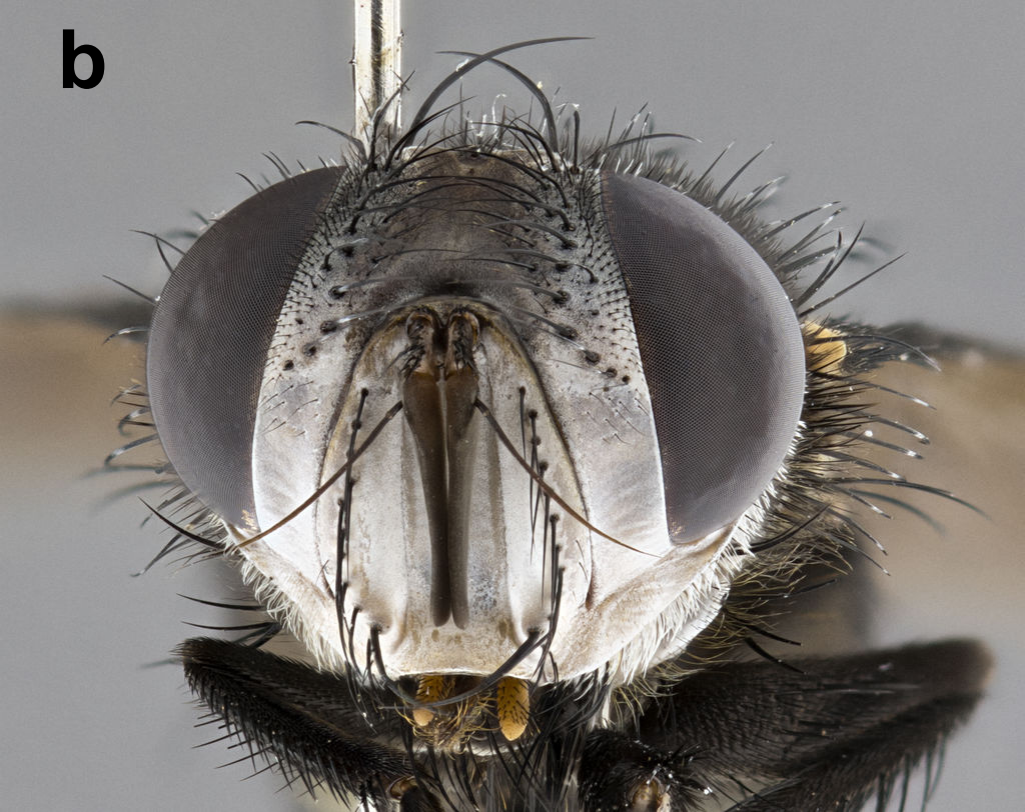
frontal view

**Figure 4c.**
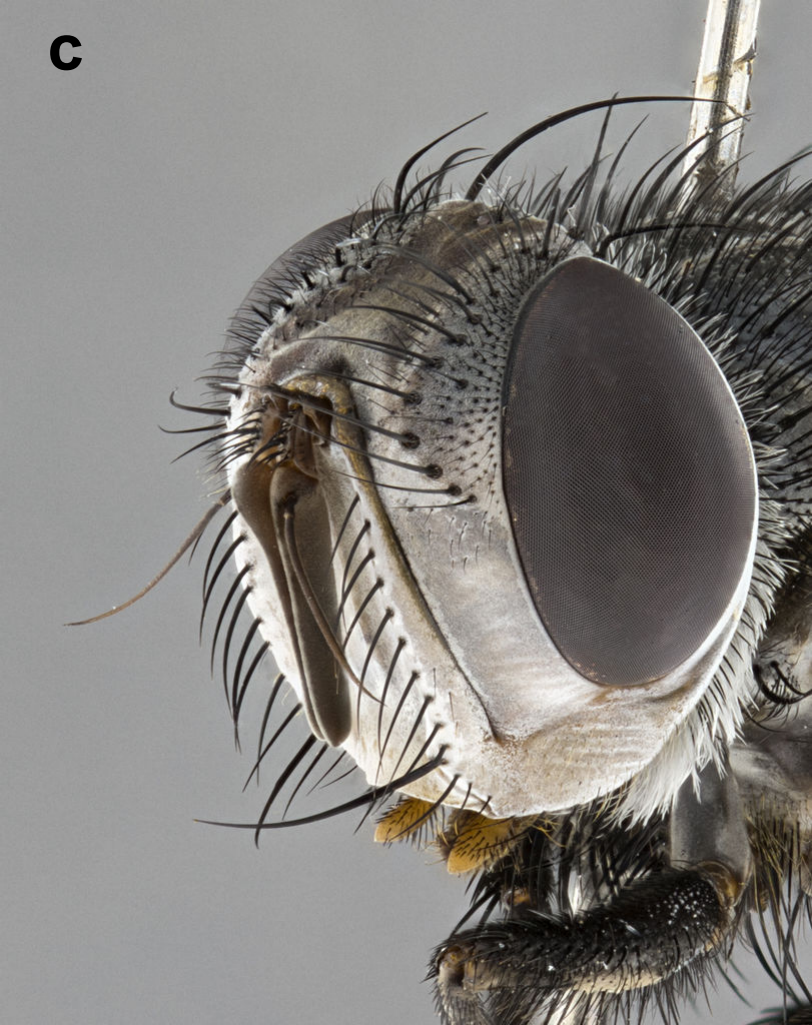
three quarters view

**Figure 4d.**
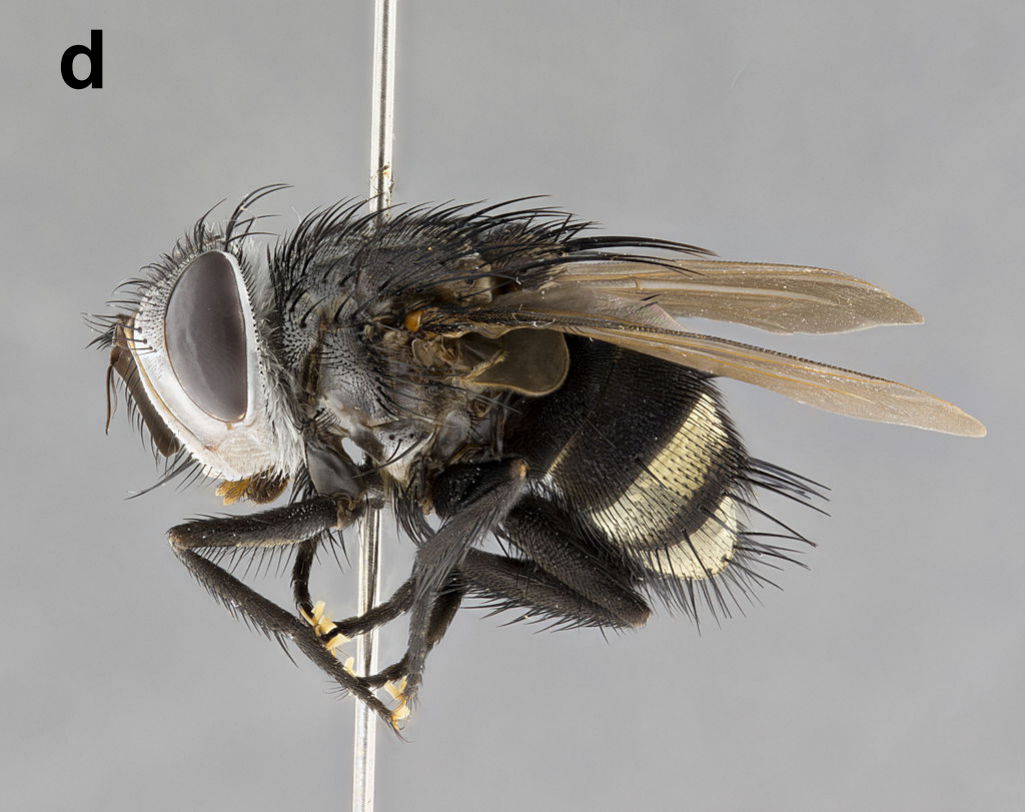
lateral view

**Figure 5a.**
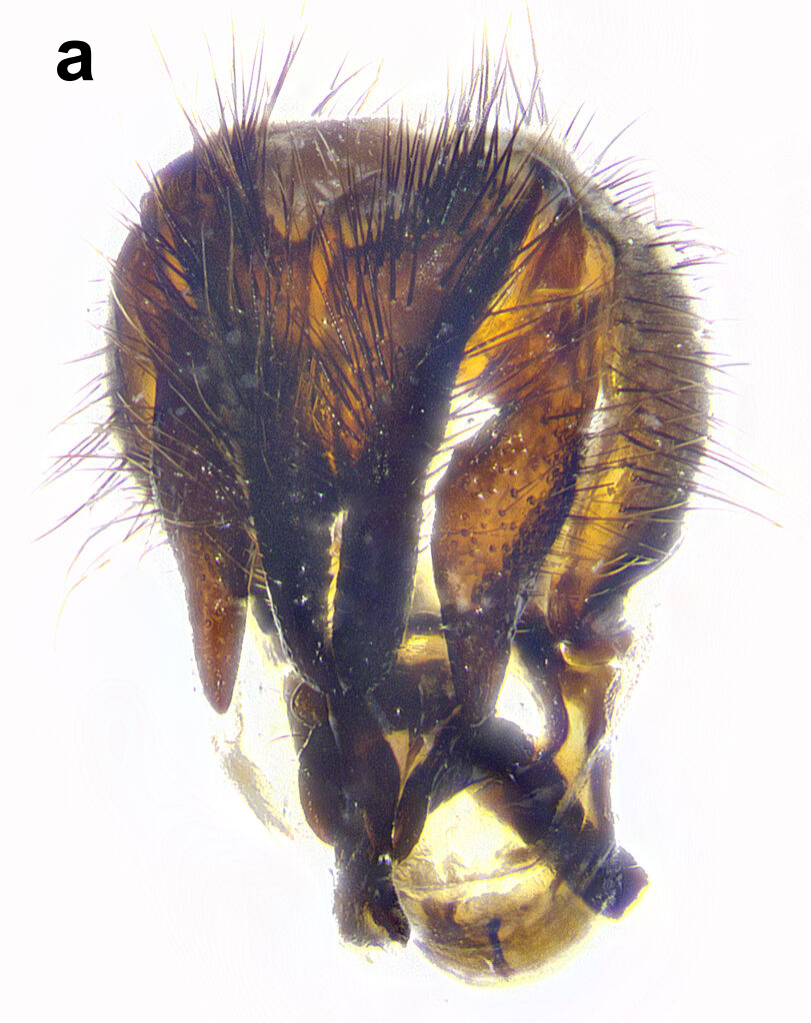
caudal view

**Figure 5b.**
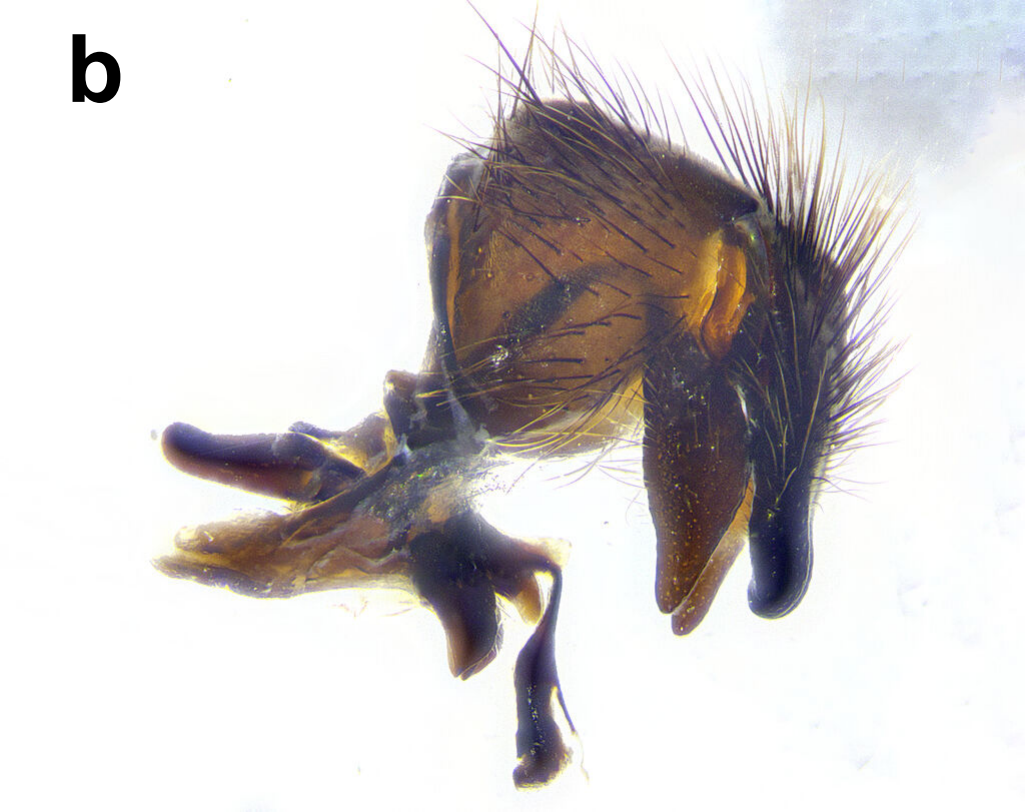
lateral view

**Figure 5c.**
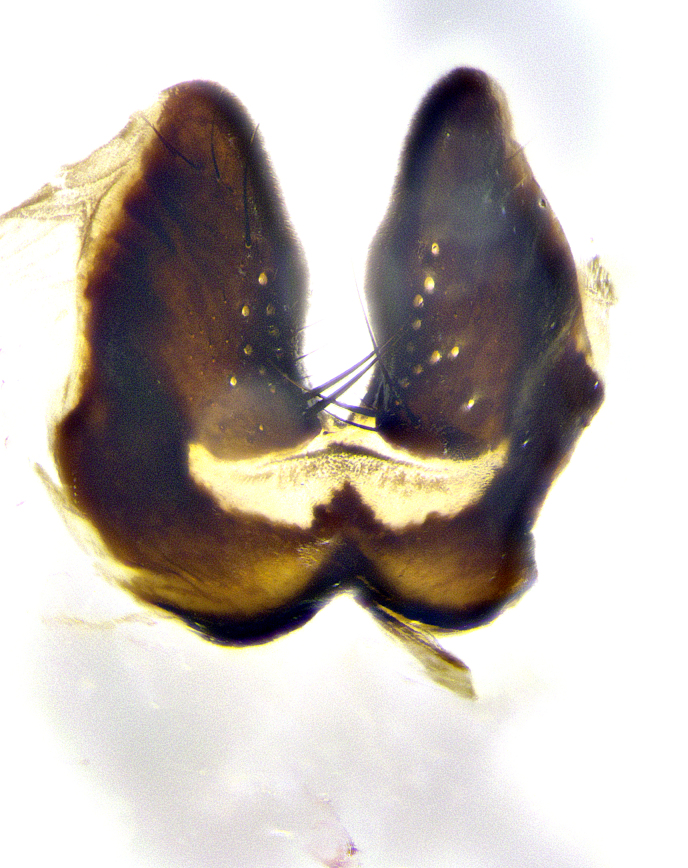
sternite 5, ventral view

**Figure 6a.**
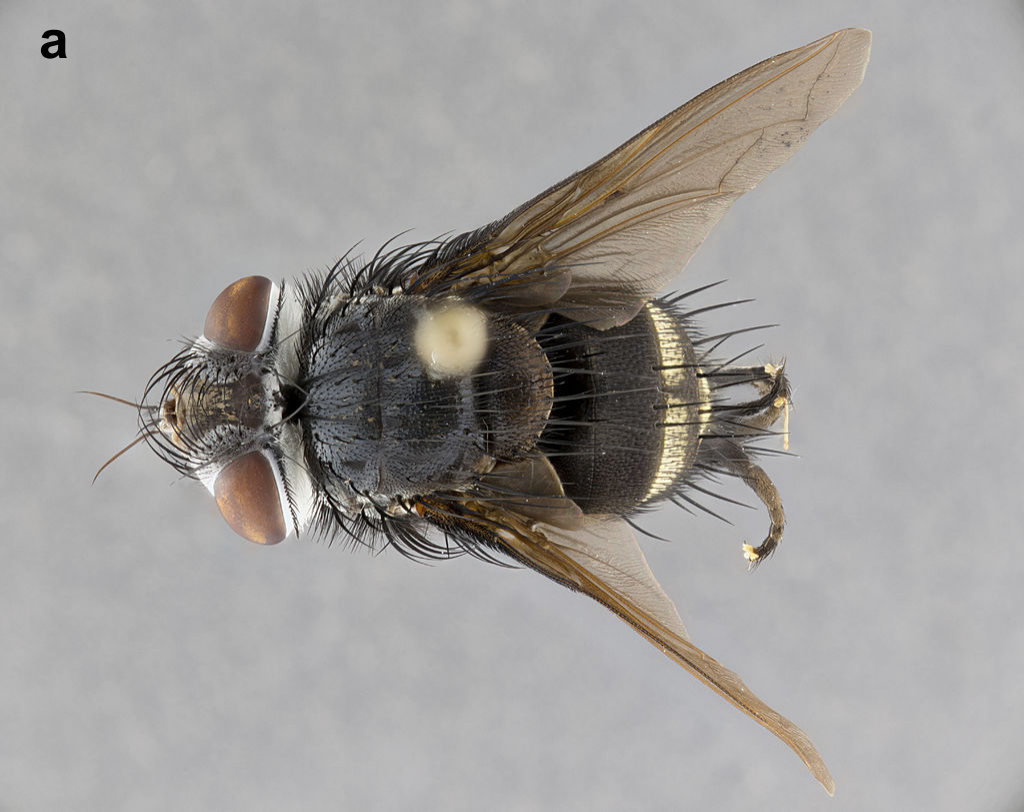
dorsal view

**Figure 6b.**
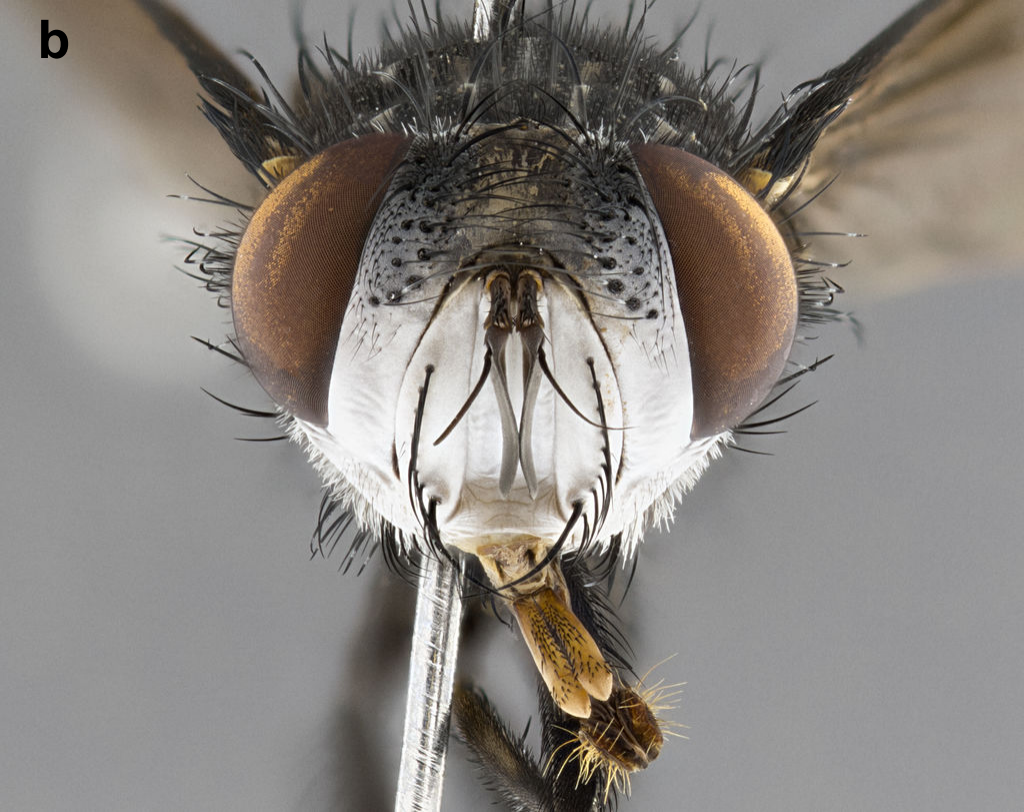
frontal view

**Figure 6c.**
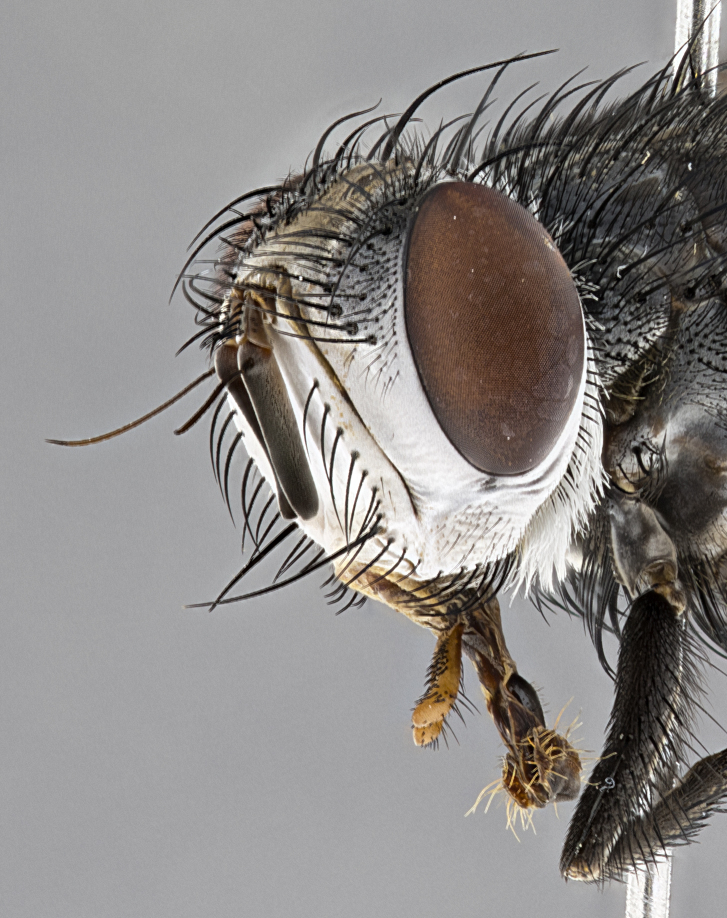
three quarters view

**Figure 6d.**
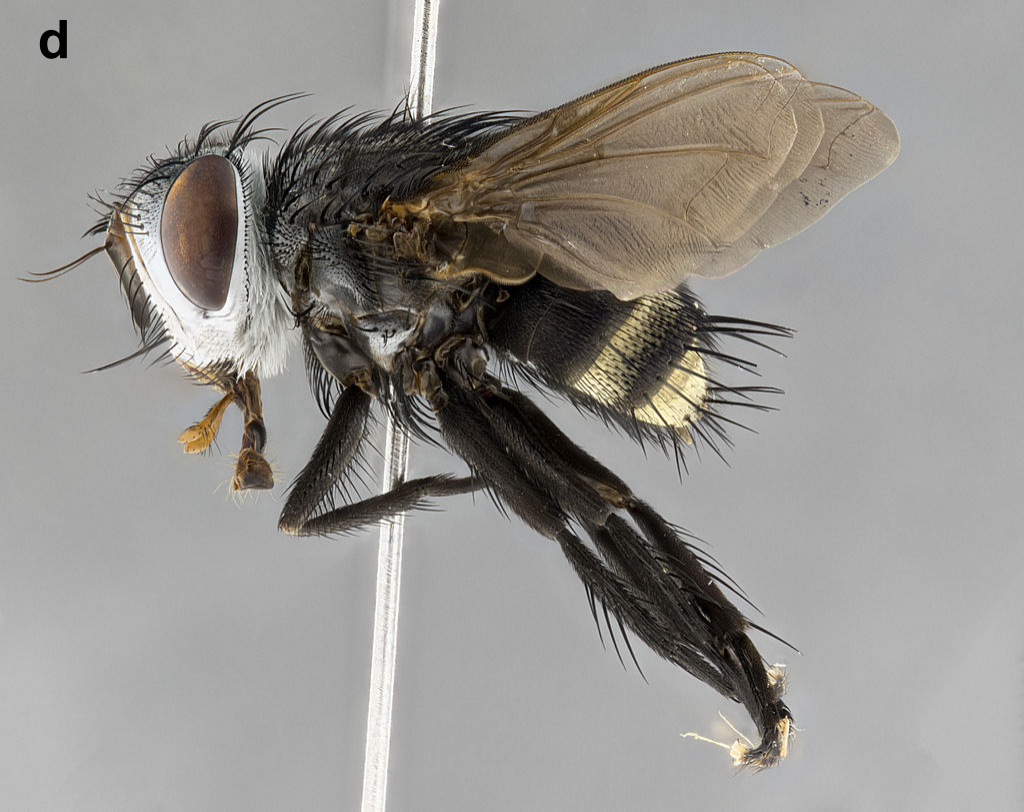
lateral view

**Figure 7a.**
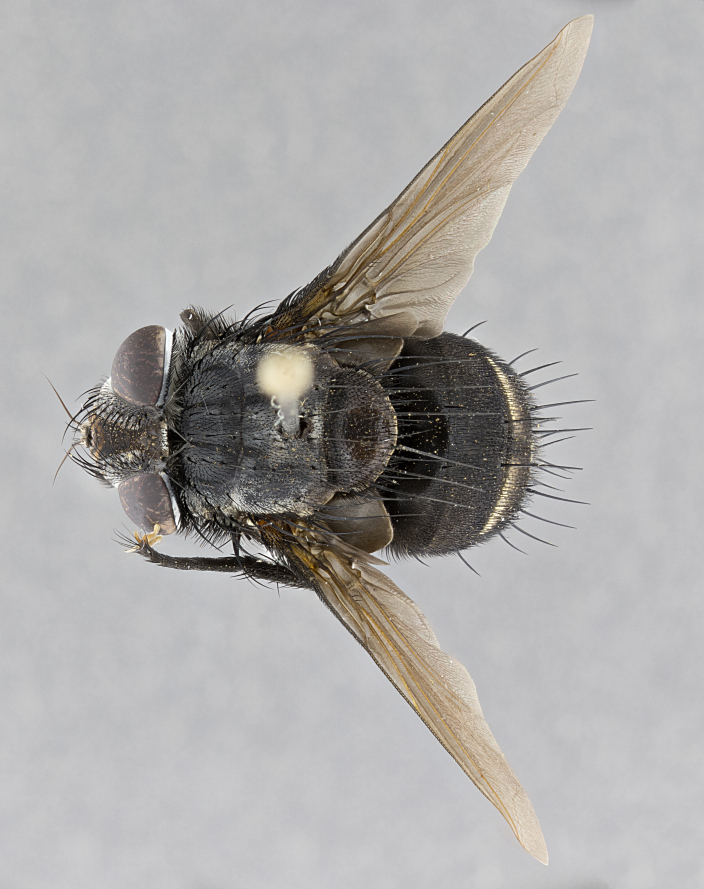
dorsal view

**Figure 7b.**
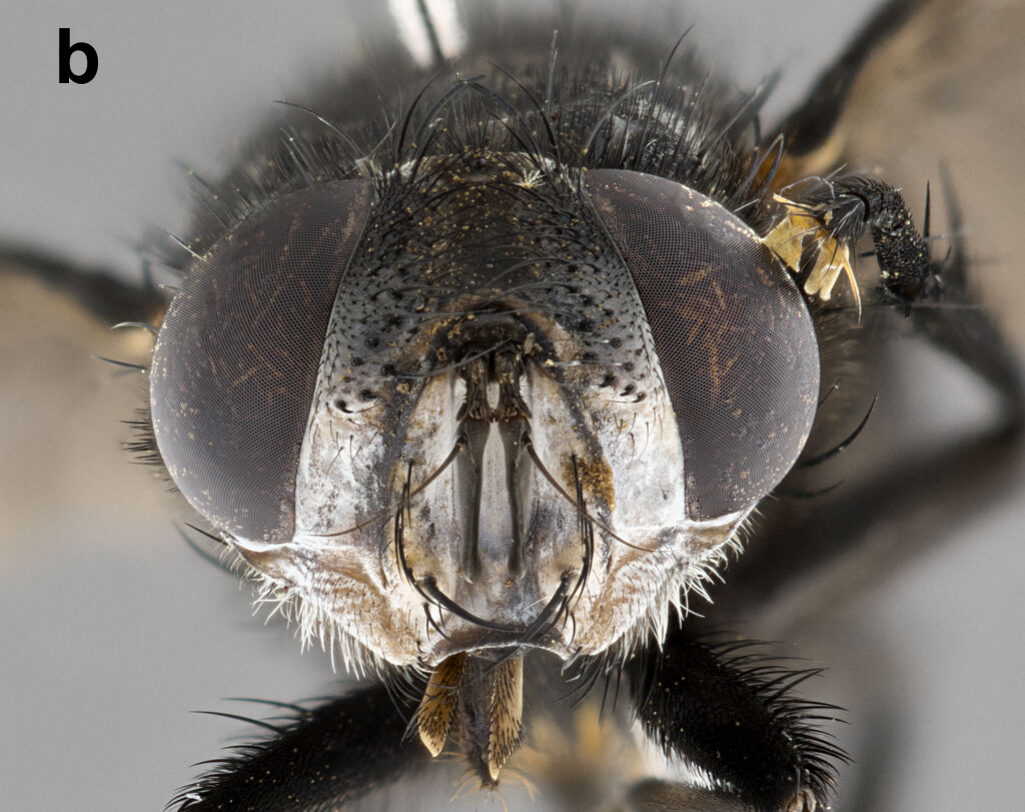
frontal view

**Figure 7c.**
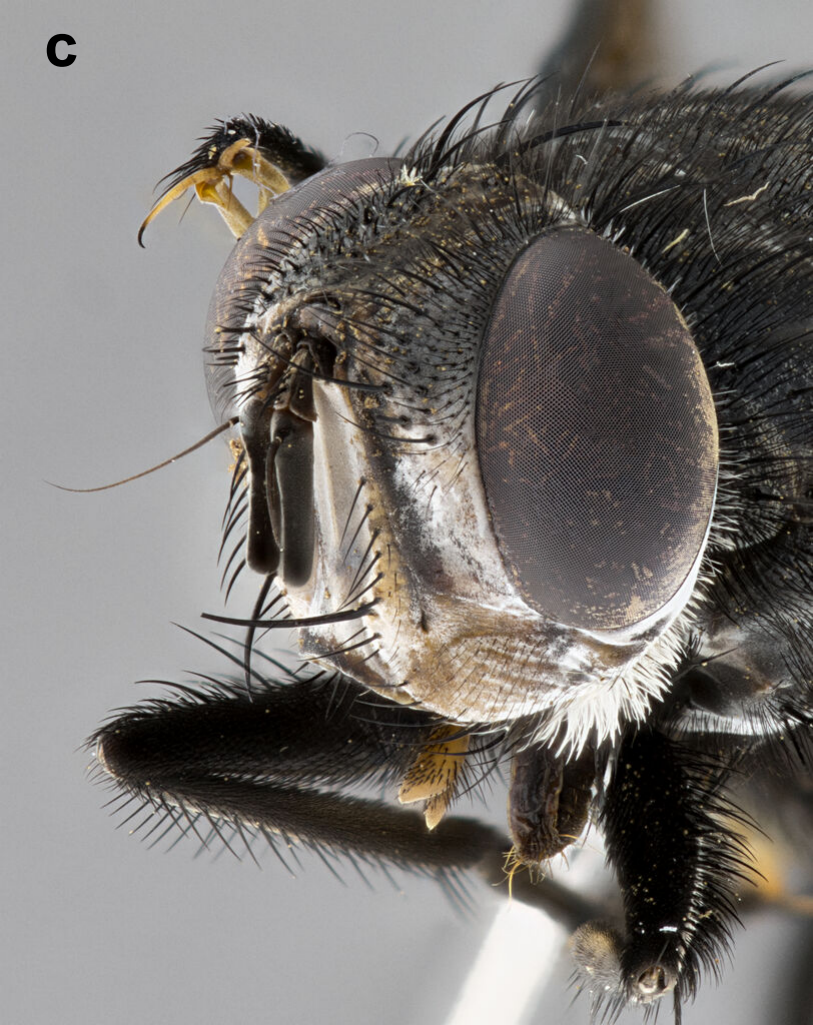
three quarters view

**Figure 7d.**
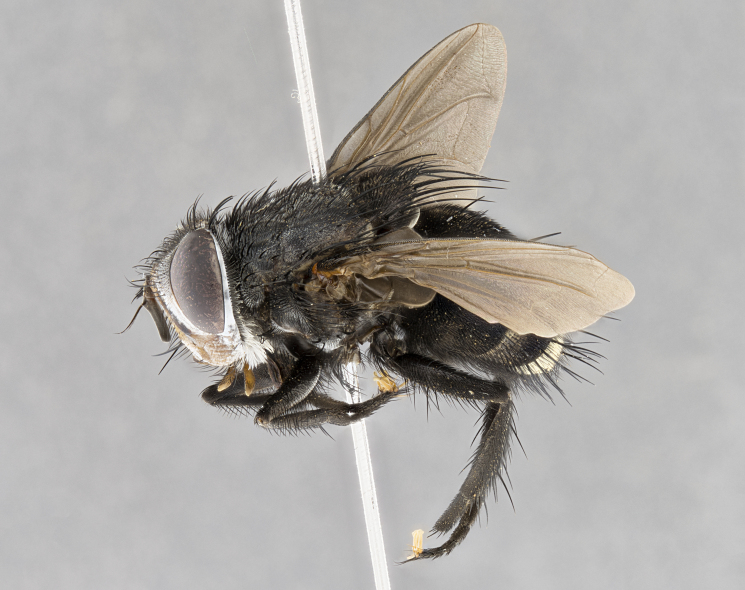
lateral view

**Figure 8a.**
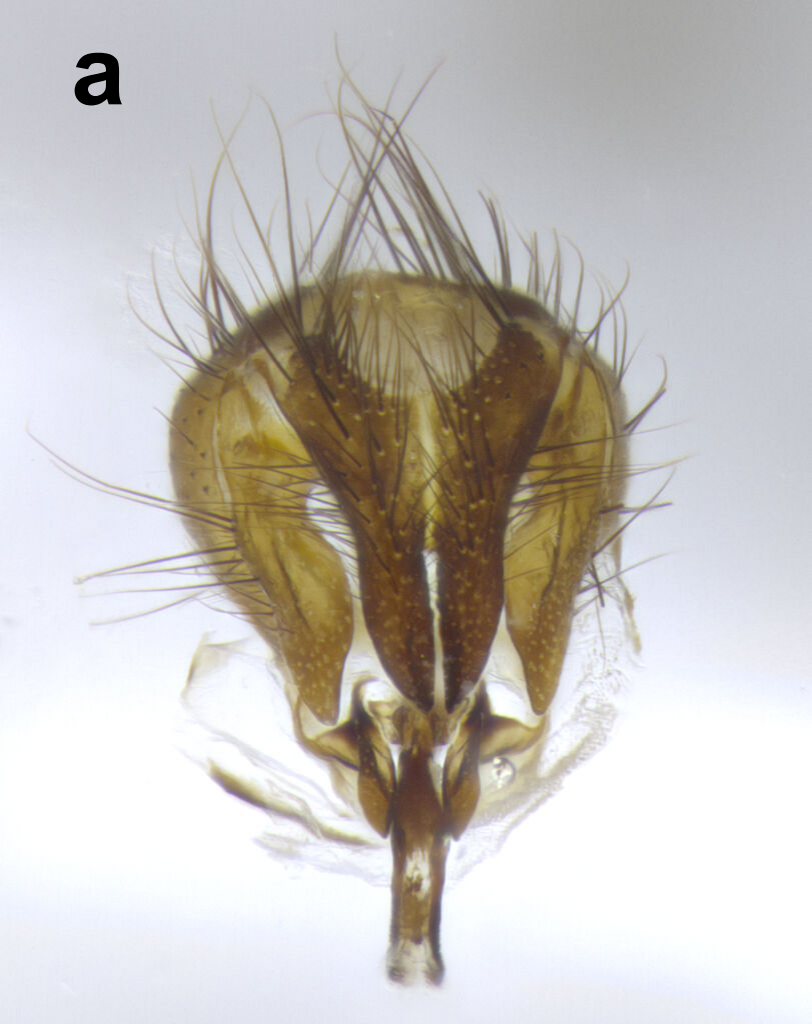
caudal view

**Figure 8b.**
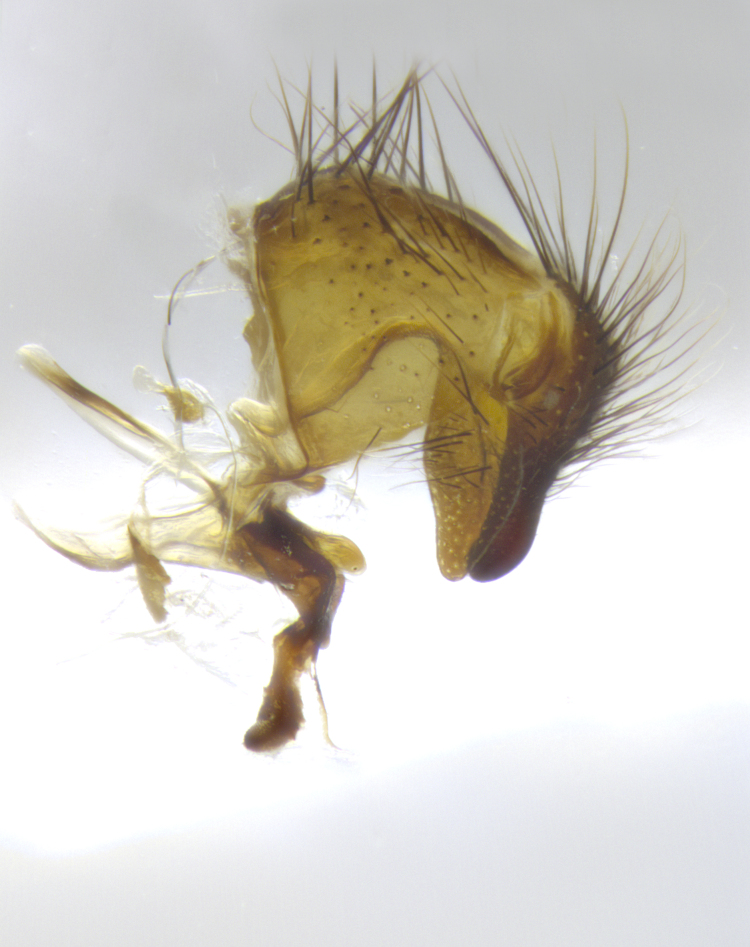
lateral view

**Figure 8c.**
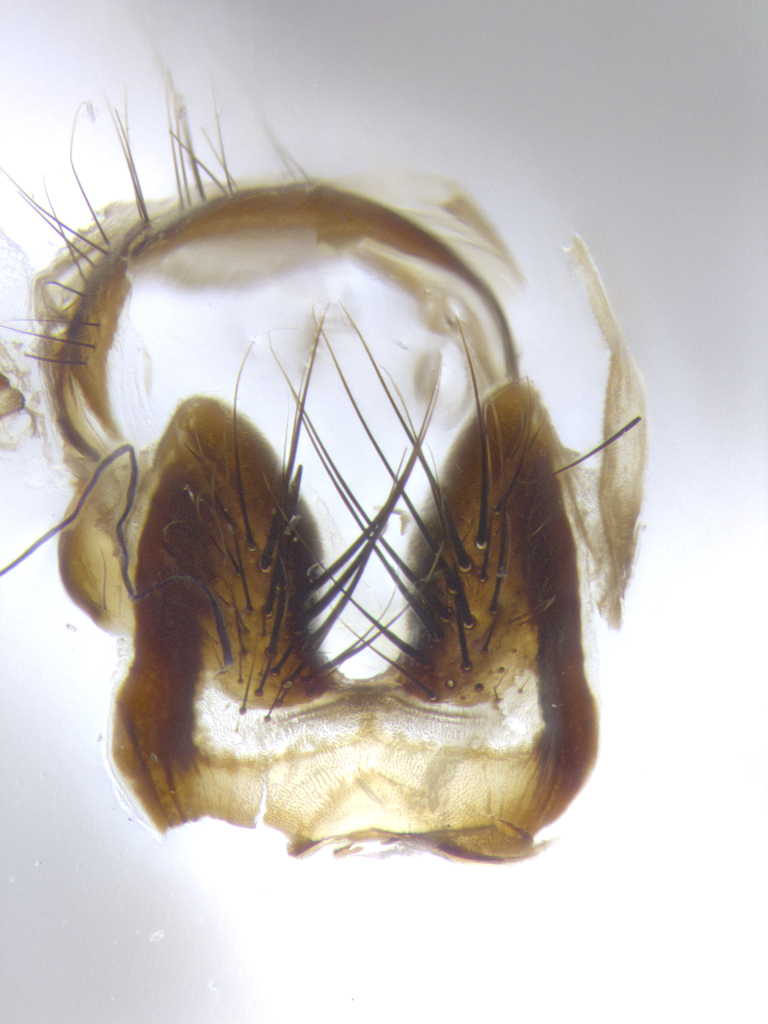
sternite 5, ventral view

**Figure 9a.**
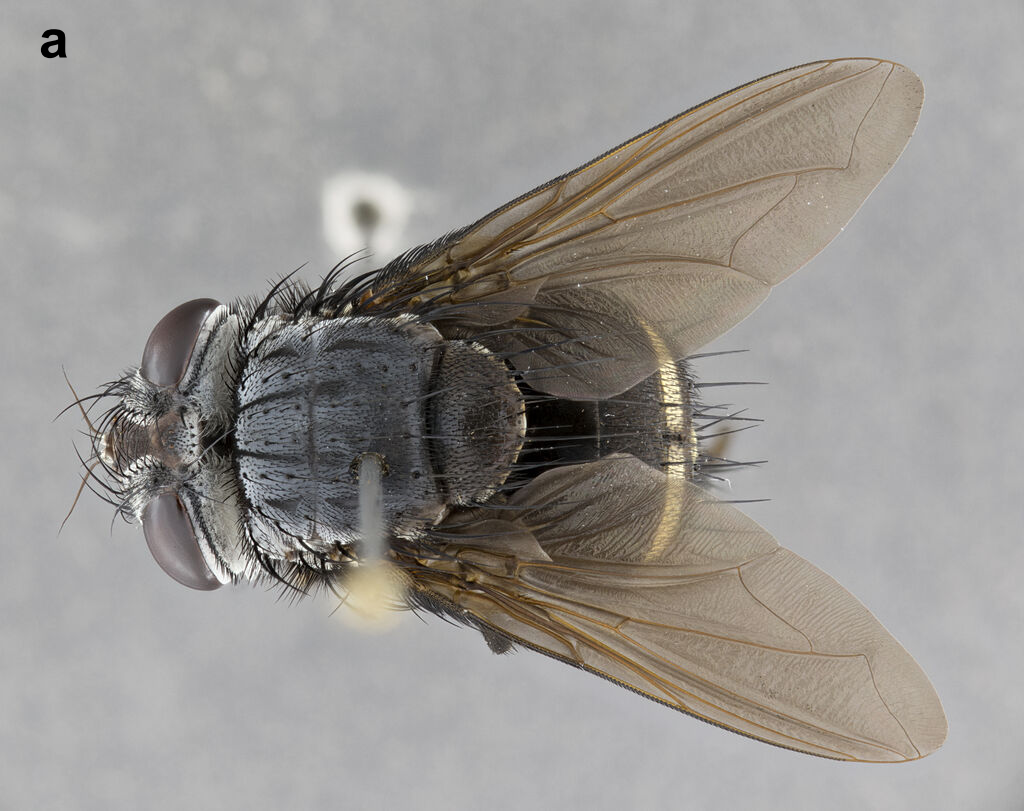
dorsal view

**Figure 9b.**
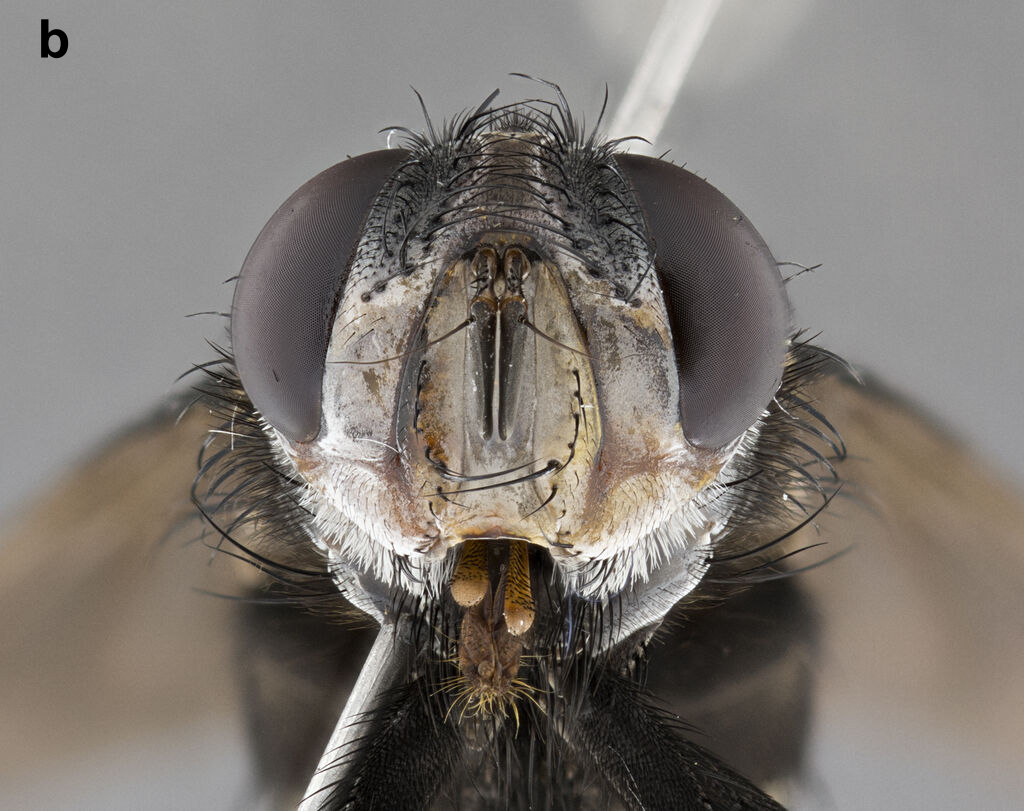
frontal view

**Figure 9c.**
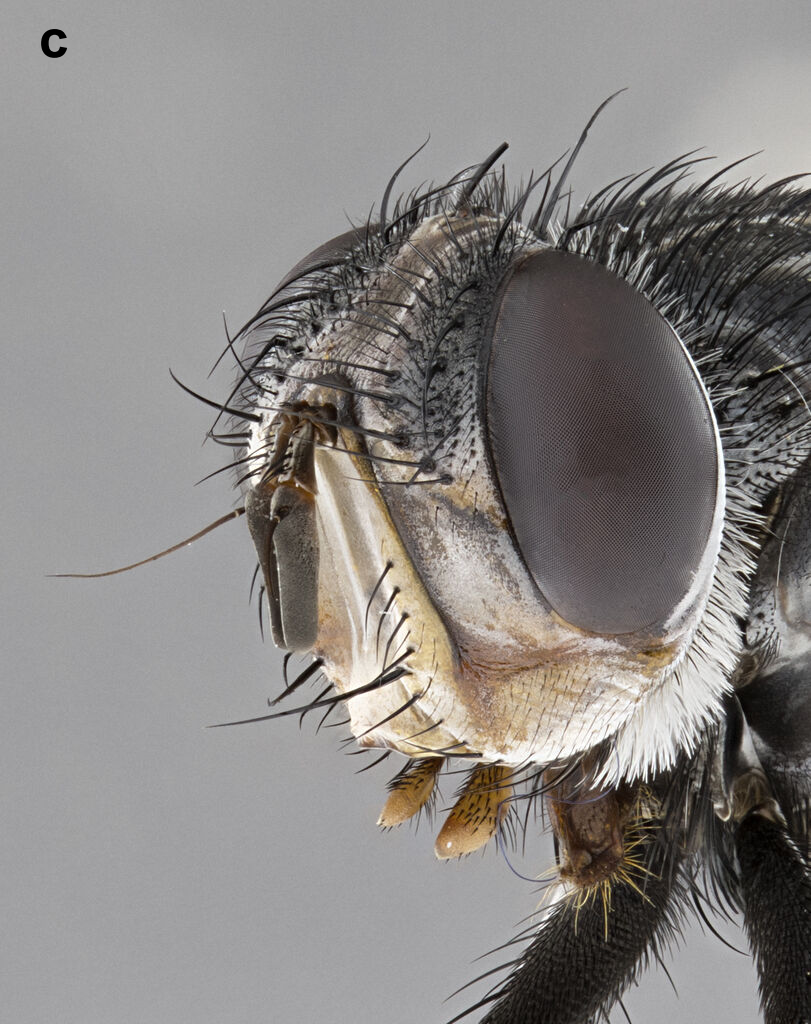
three quarters view

**Figure 9d.**
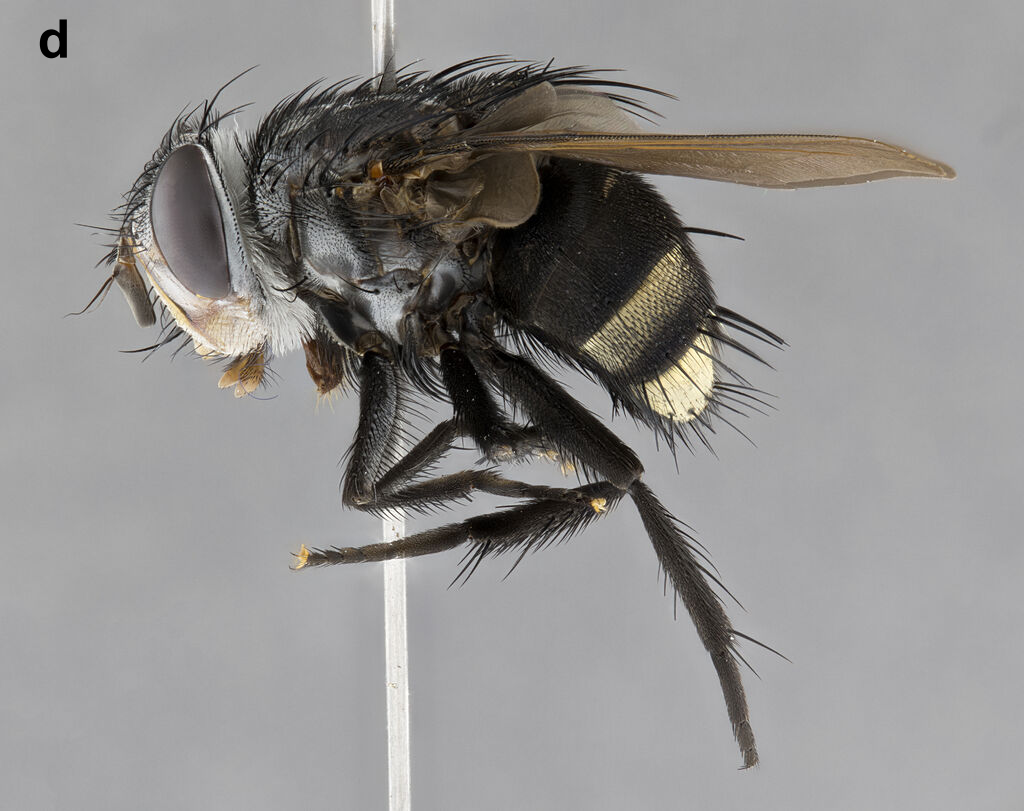
lateral view

**Figure 10a.**
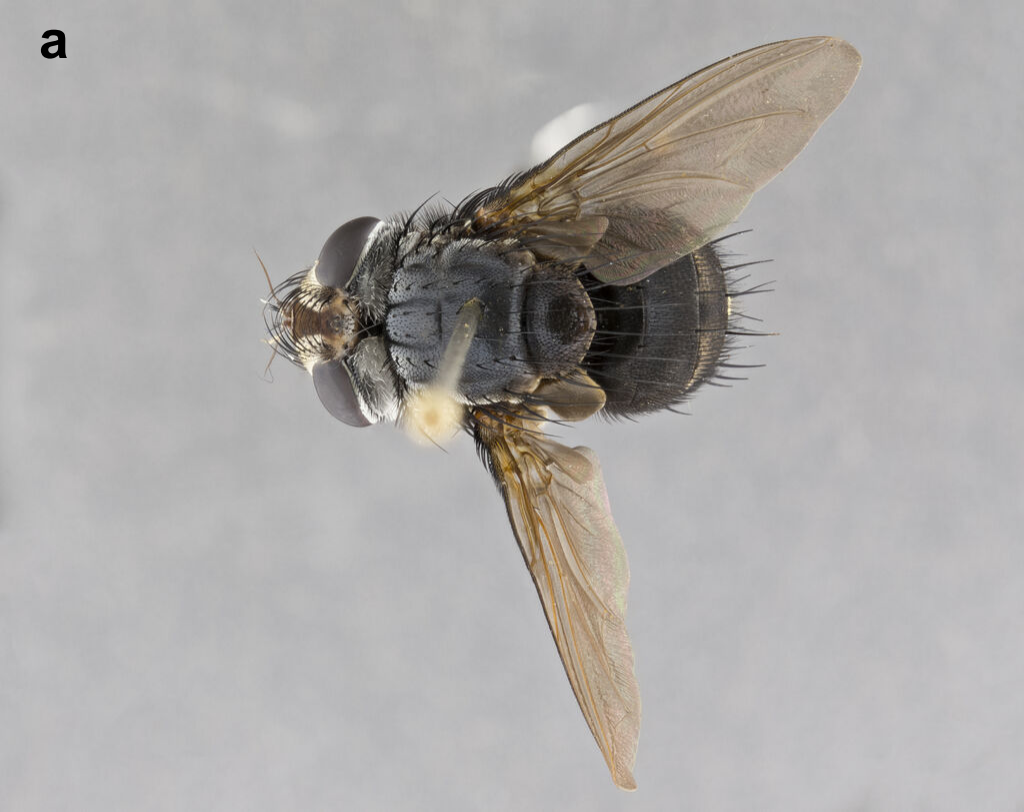
dorsal view

**Figure 10b.**
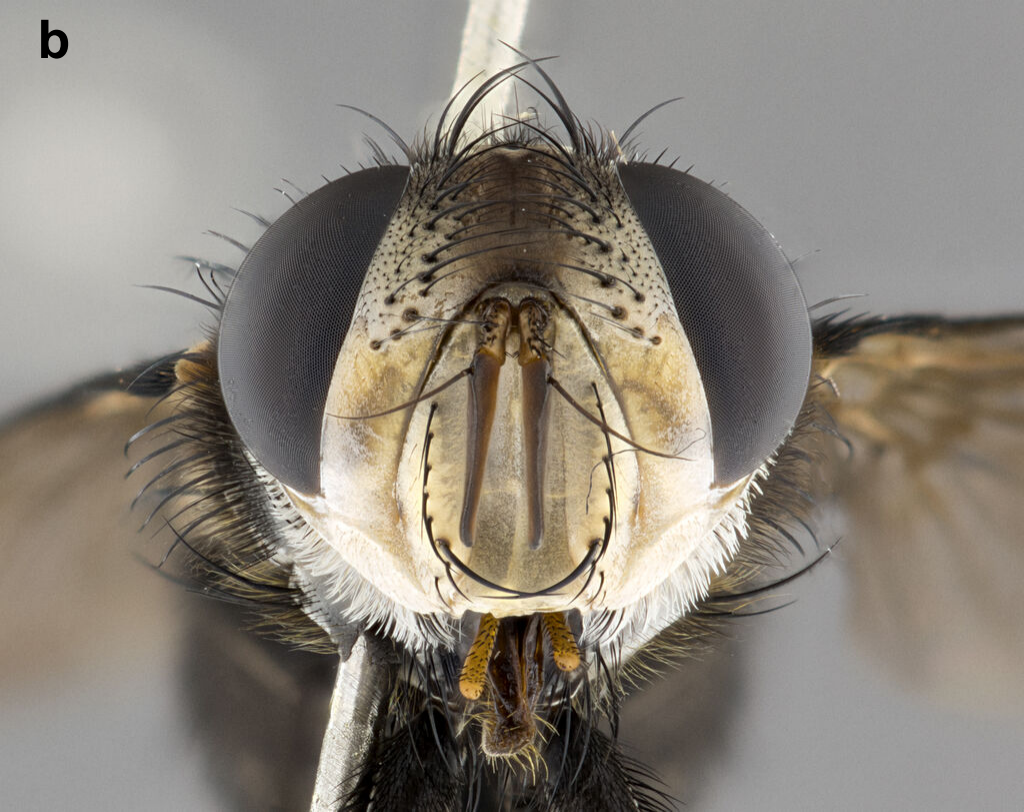
frontal view

**Figure 10c.**
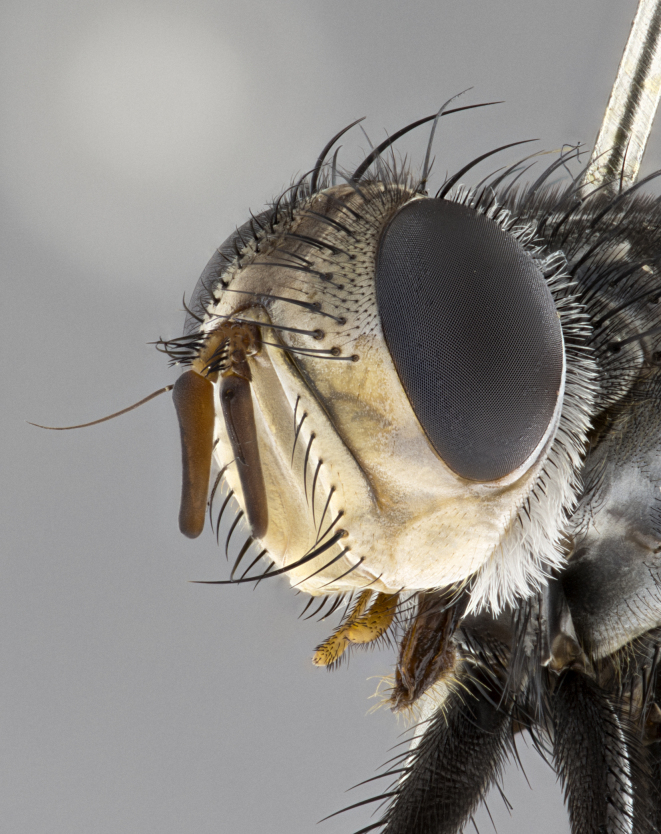
three quarters view

**Figure 10d.**
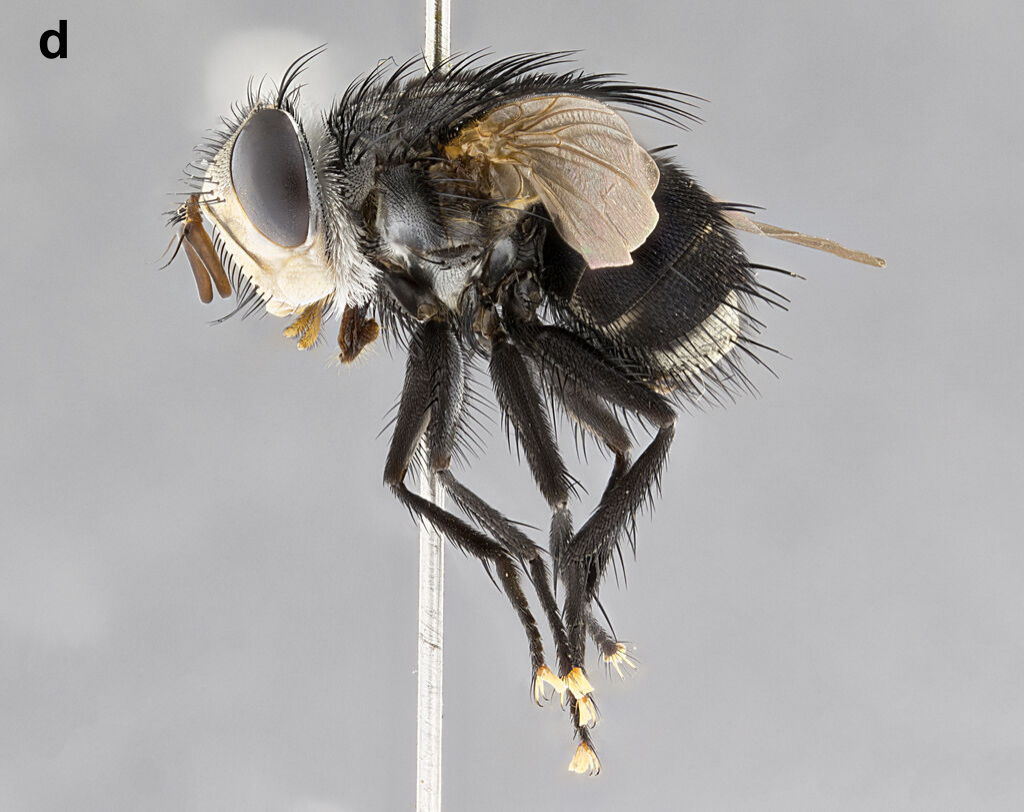
lateral view

**Figure 11a.**
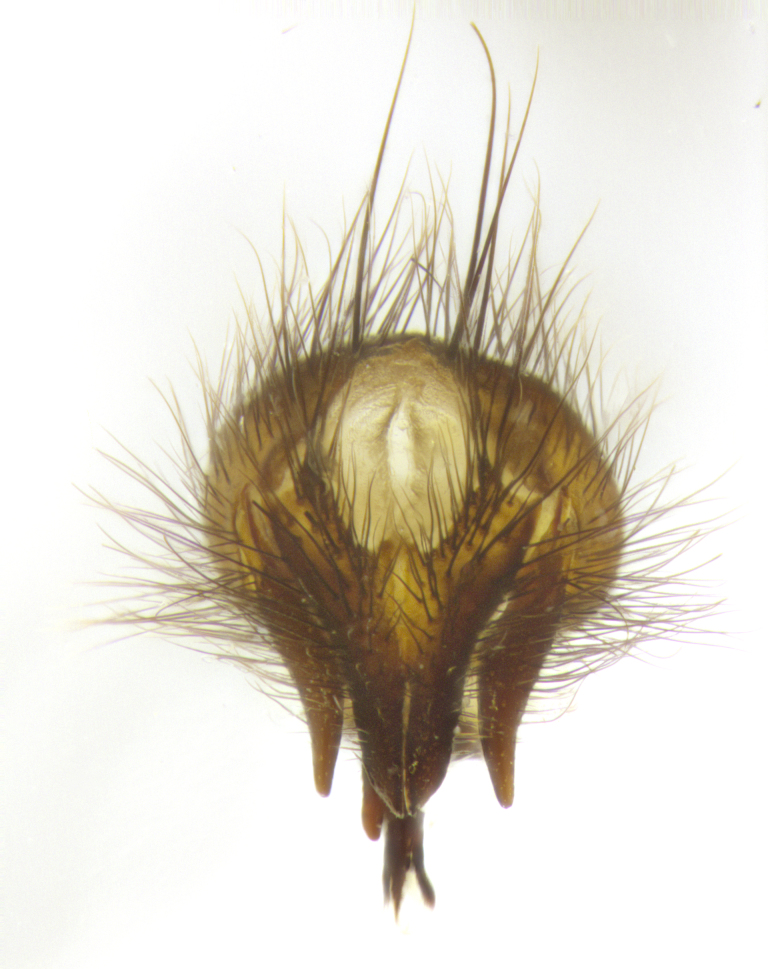
caudal view

**Figure 11b.**
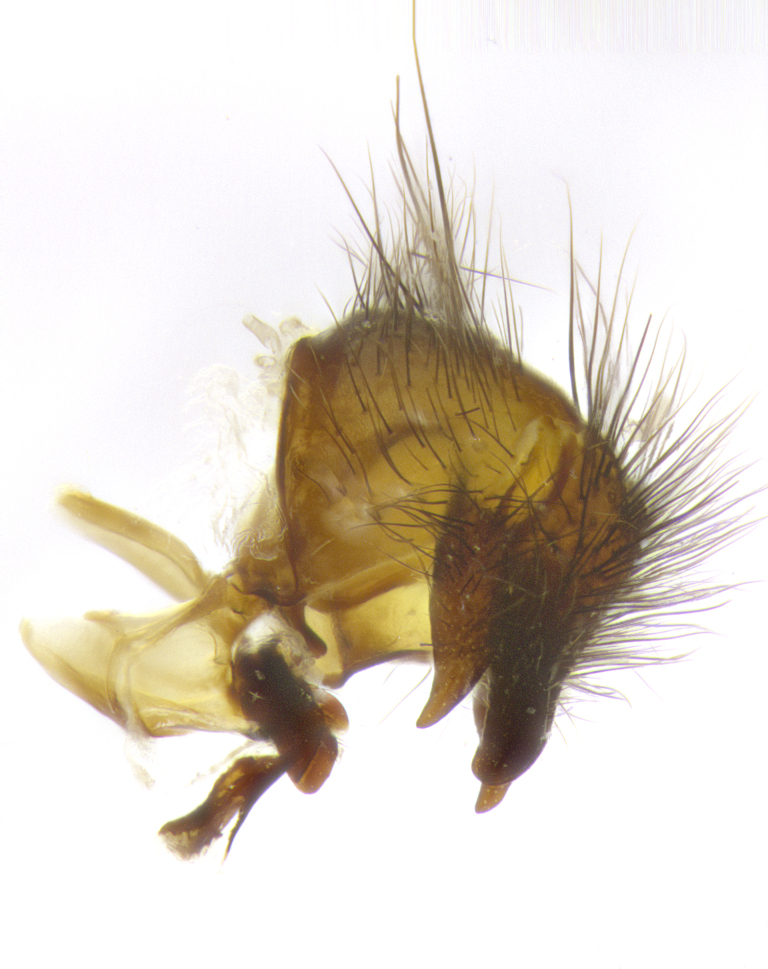
lateral view

**Figure 11c.**
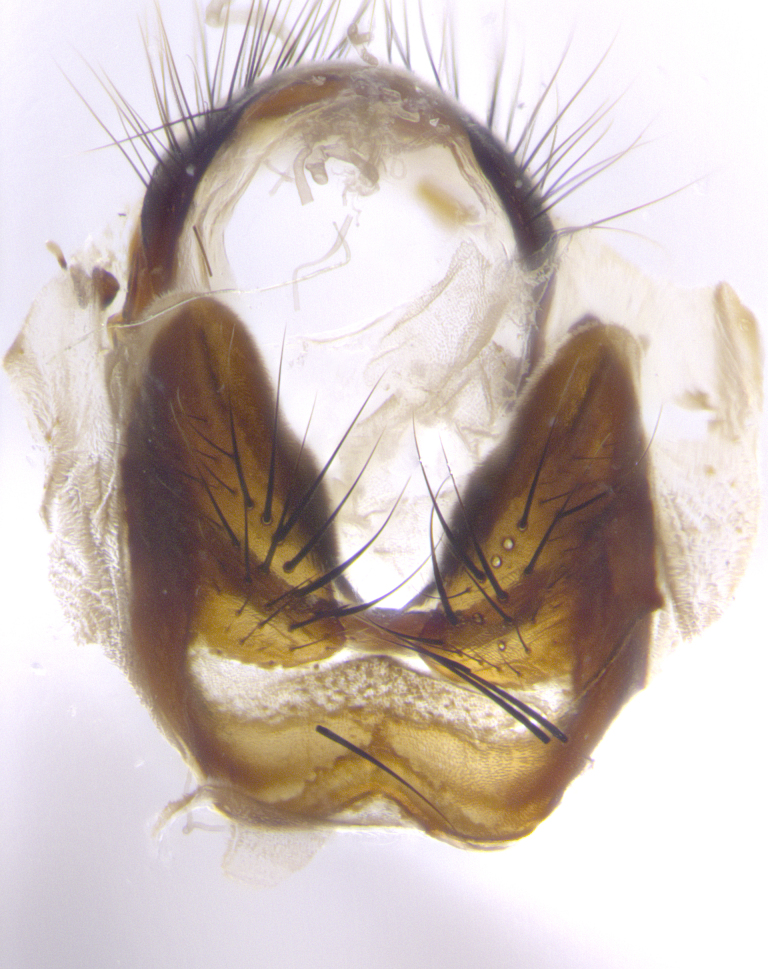
sternite 5, ventral view

**Figure 12a.**
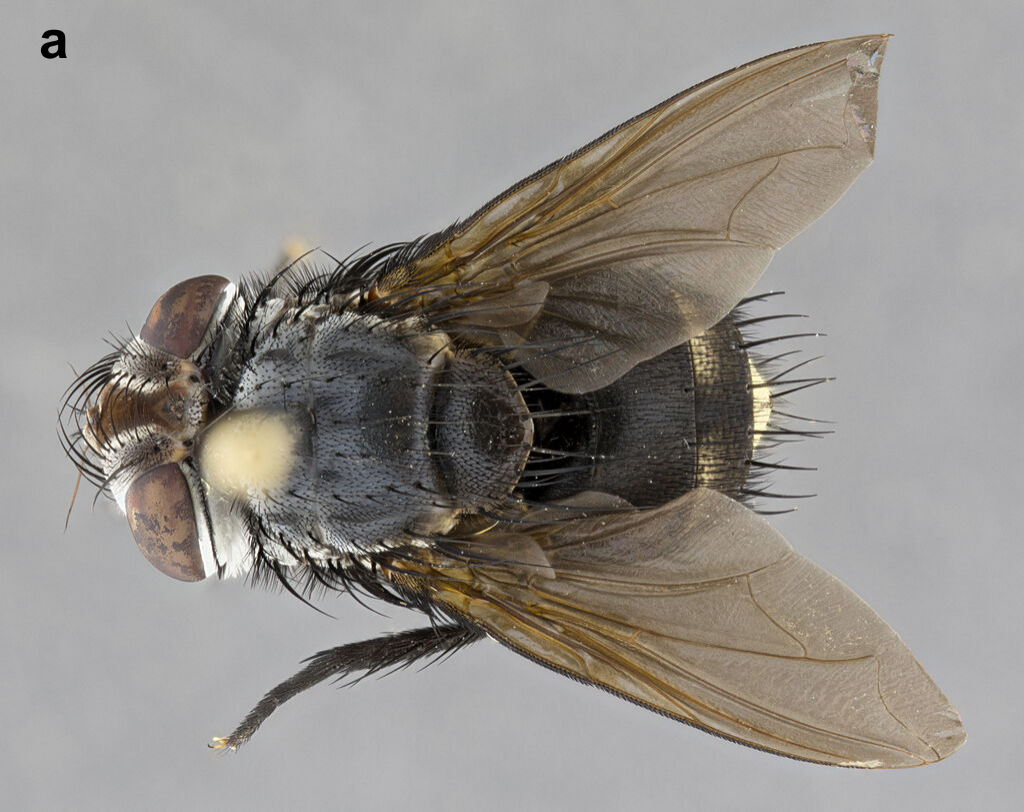
dorsal view

**Figure 12b.**
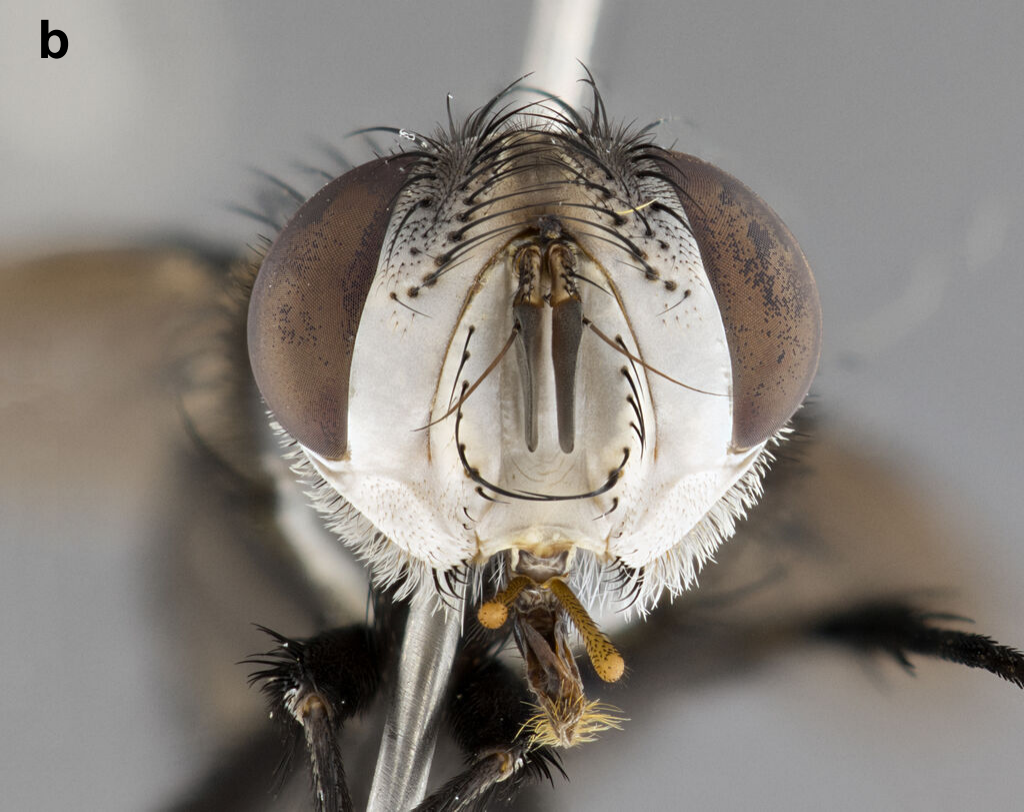
frontal view

**Figure 12c.**
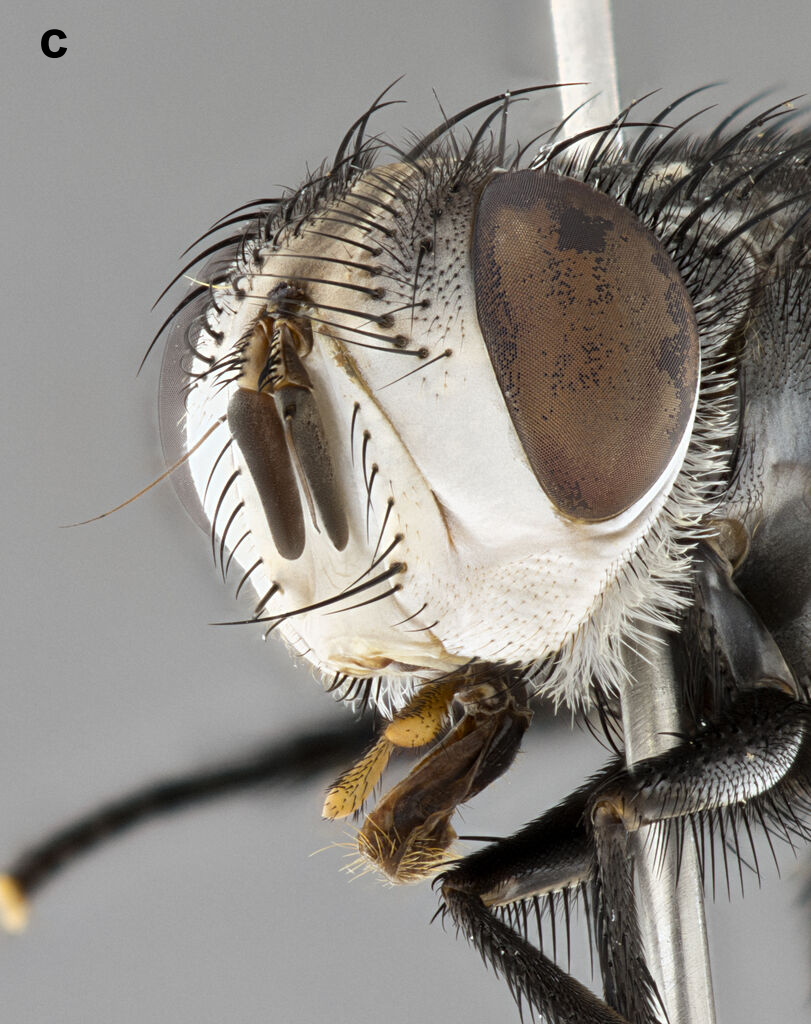
three quarters view

**Figure 12d.**
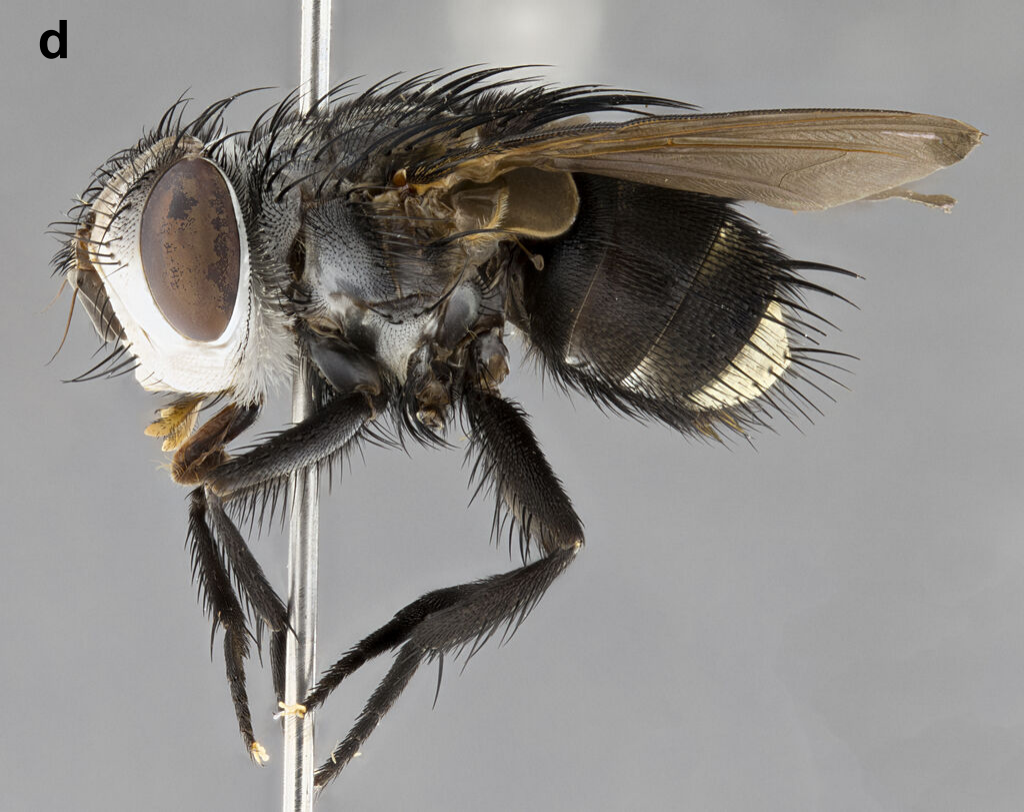
lateral view

**Figure 13a.**
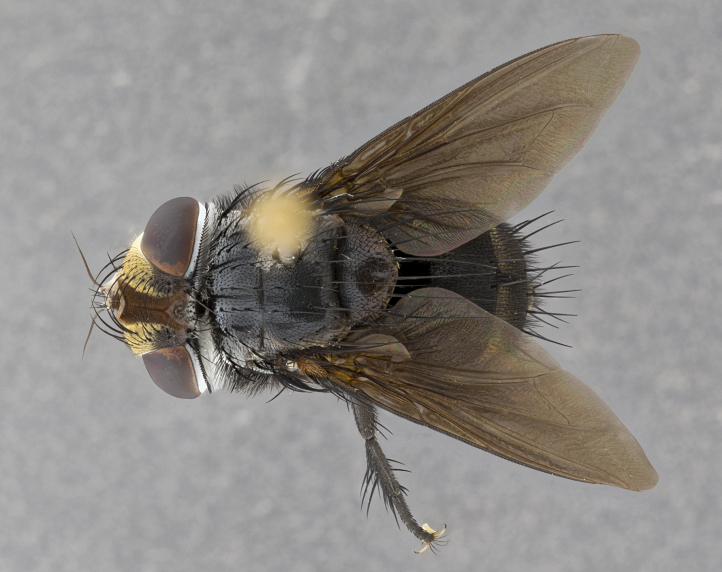
dorsal view

**Figure 13b.**
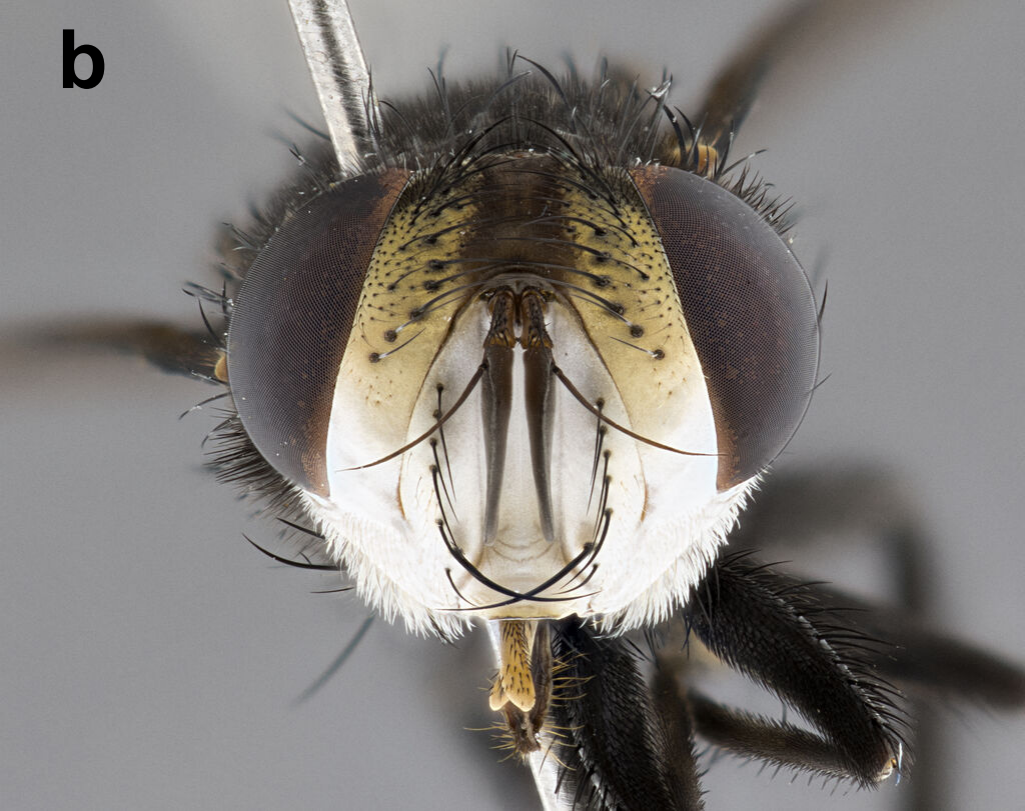
frontal view

**Figure 13c.**
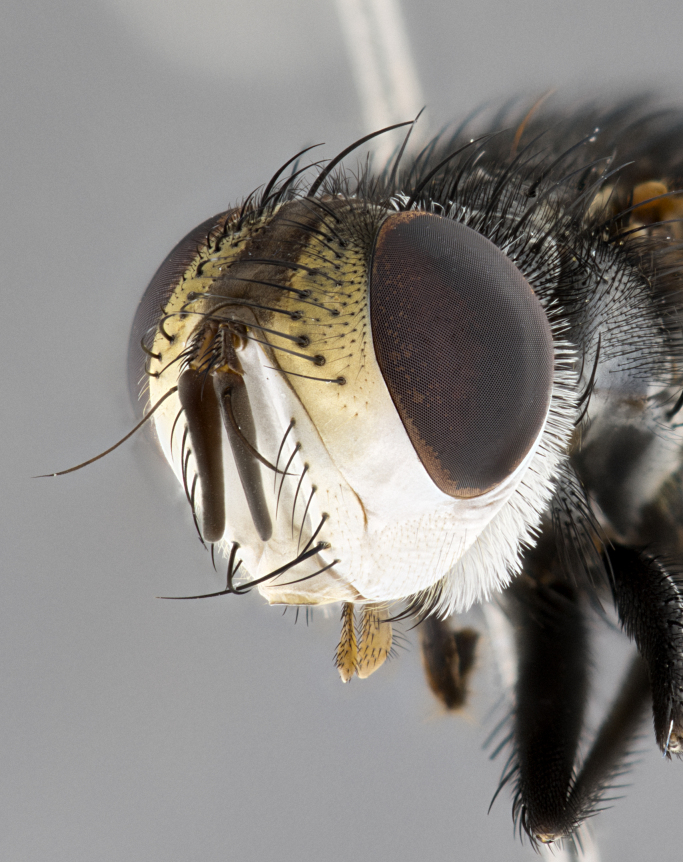
three quarters view

**Figure 13d.**
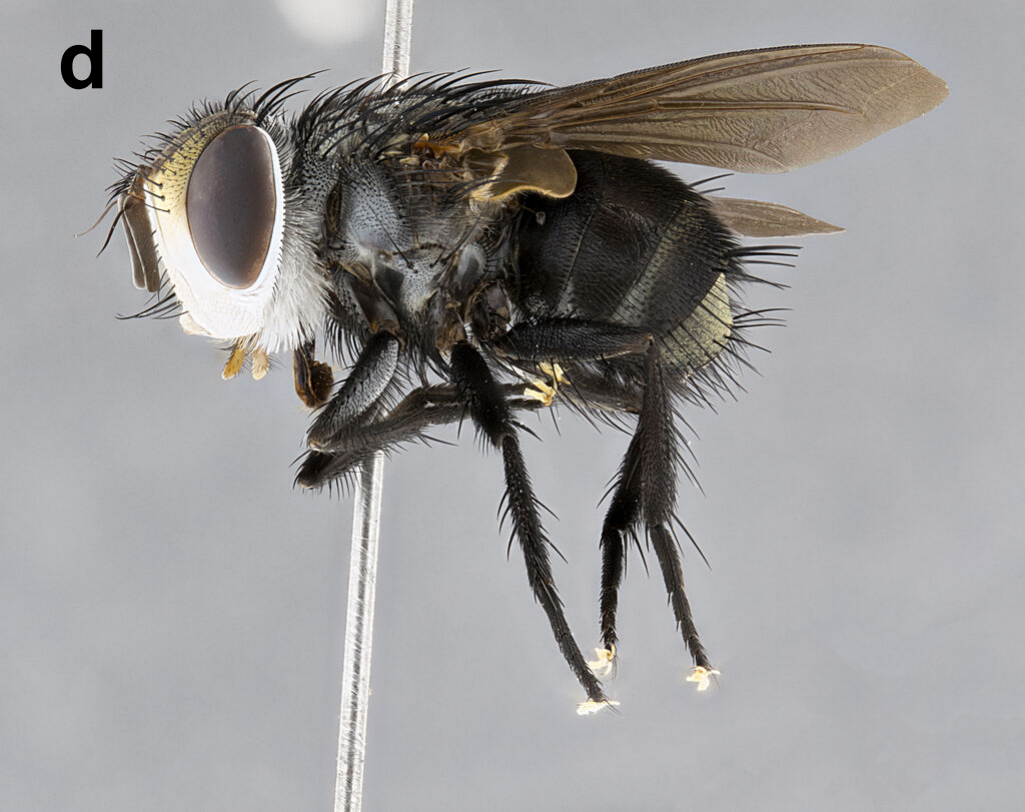
lateral view

**Figure 14a.**
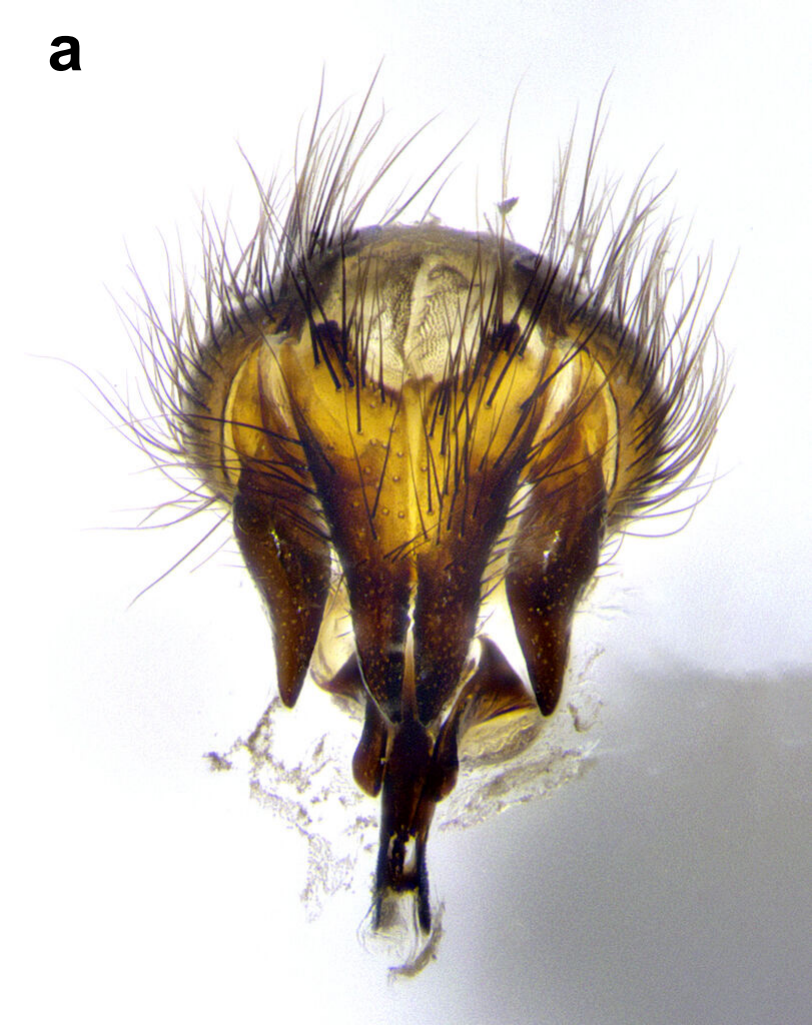
caudal view

**Figure 14b.**
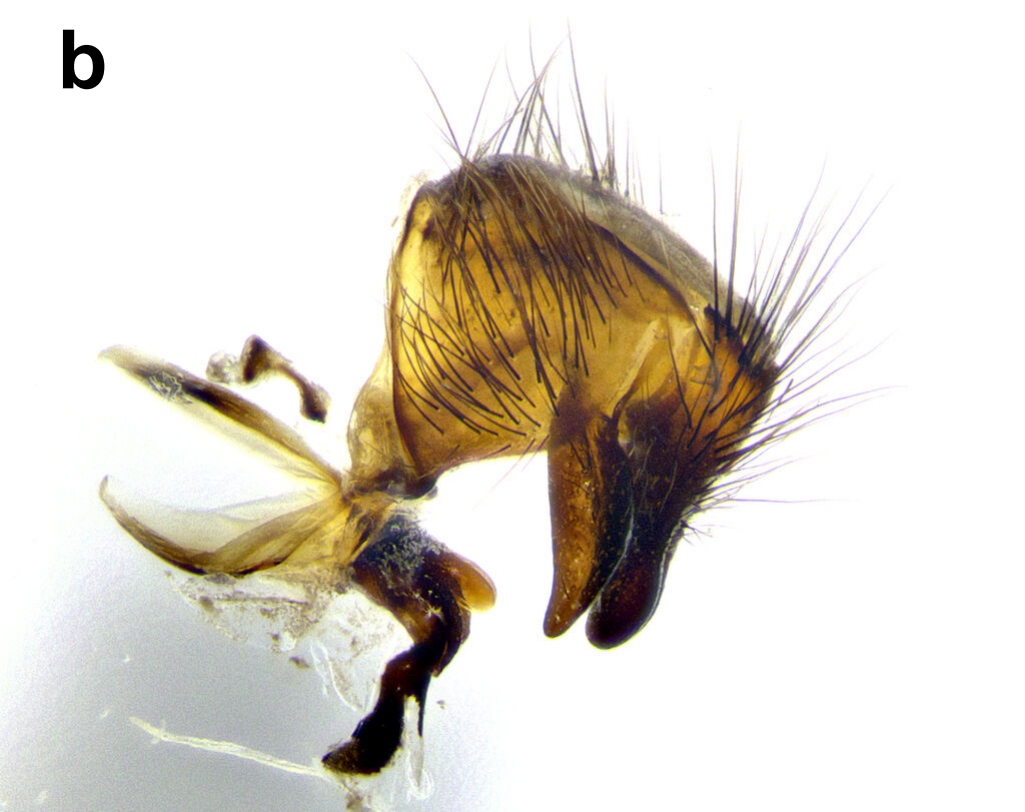
ventral view

**Figure 14c.**
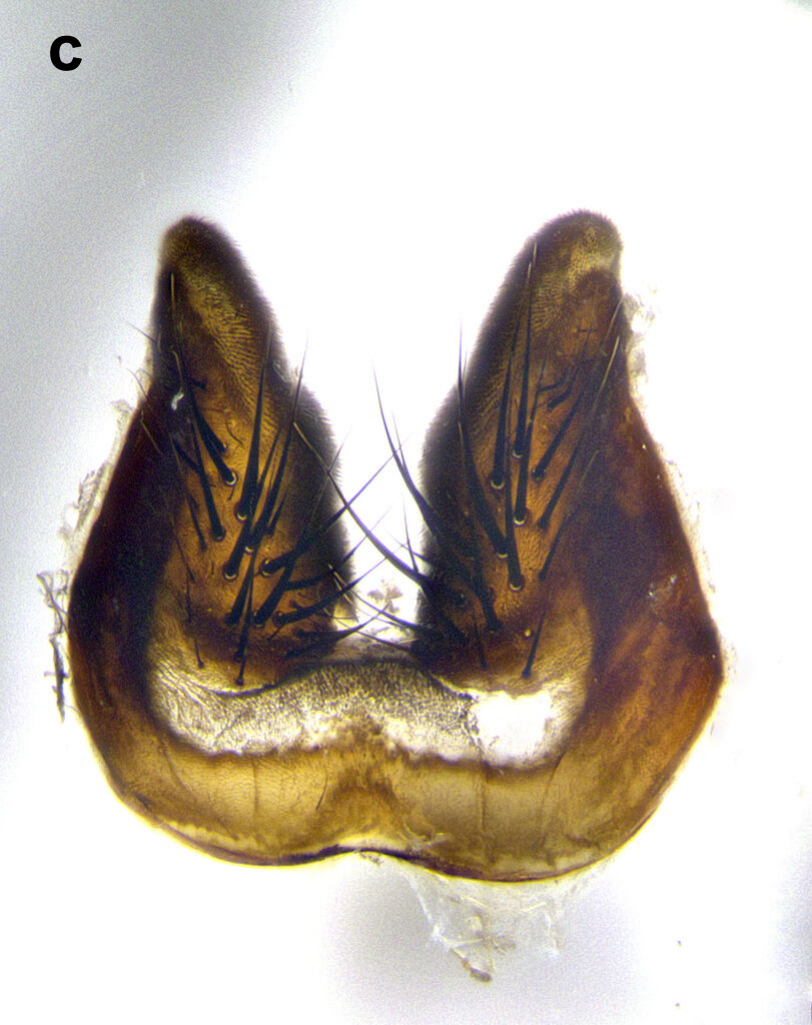
sternite 5, ventral view

**Figure 15a.**
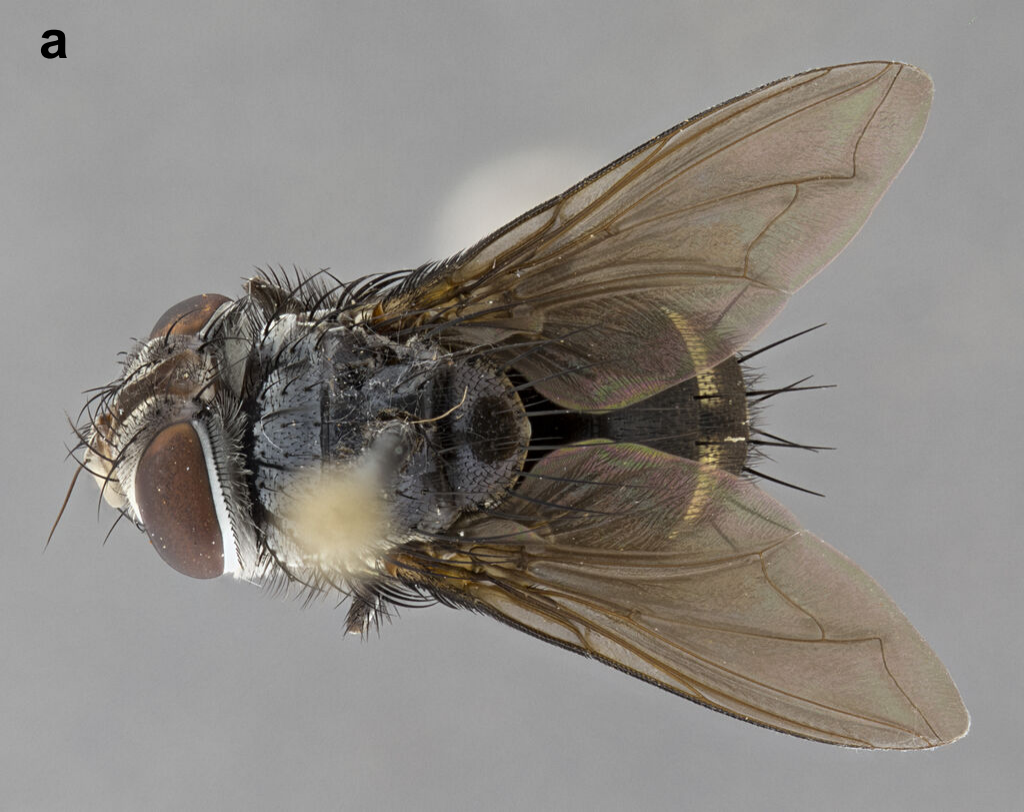
dorsal view

**Figure 15b.**
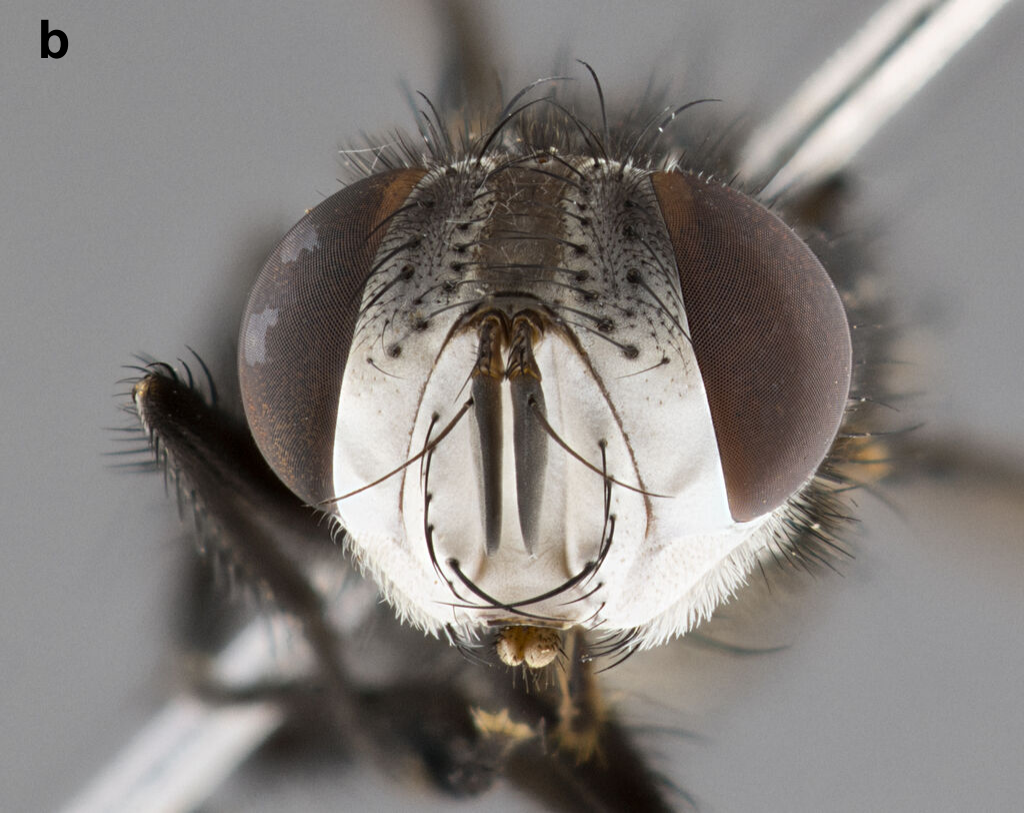
frontal view

**Figure 15c.**
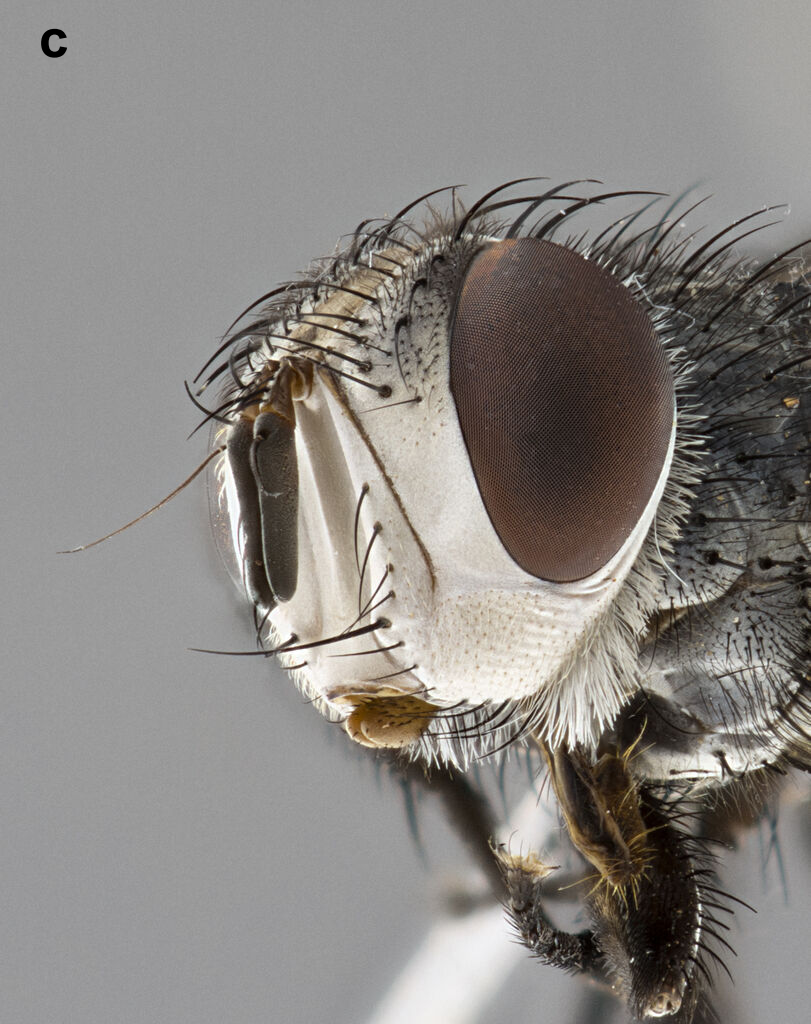
three quarters view

**Figure 15d.**
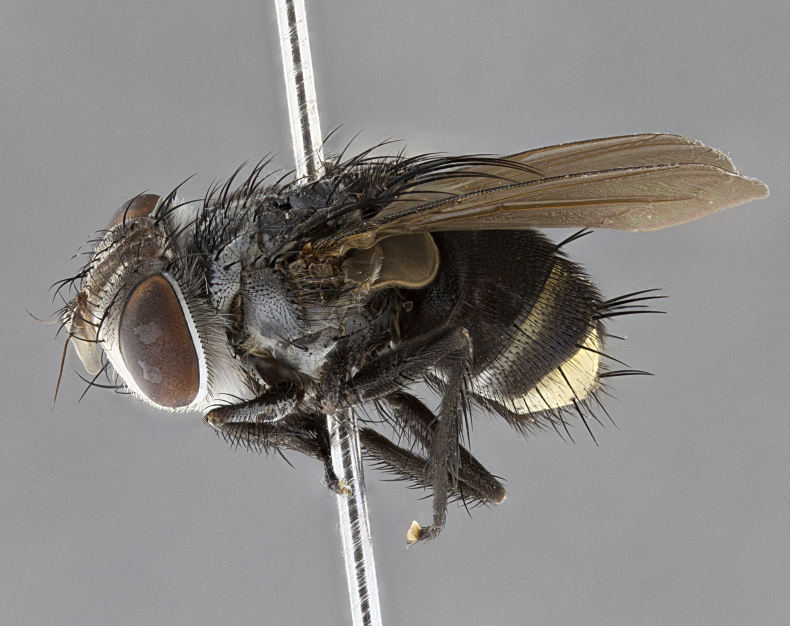
lateral view

**Figure 16a.**
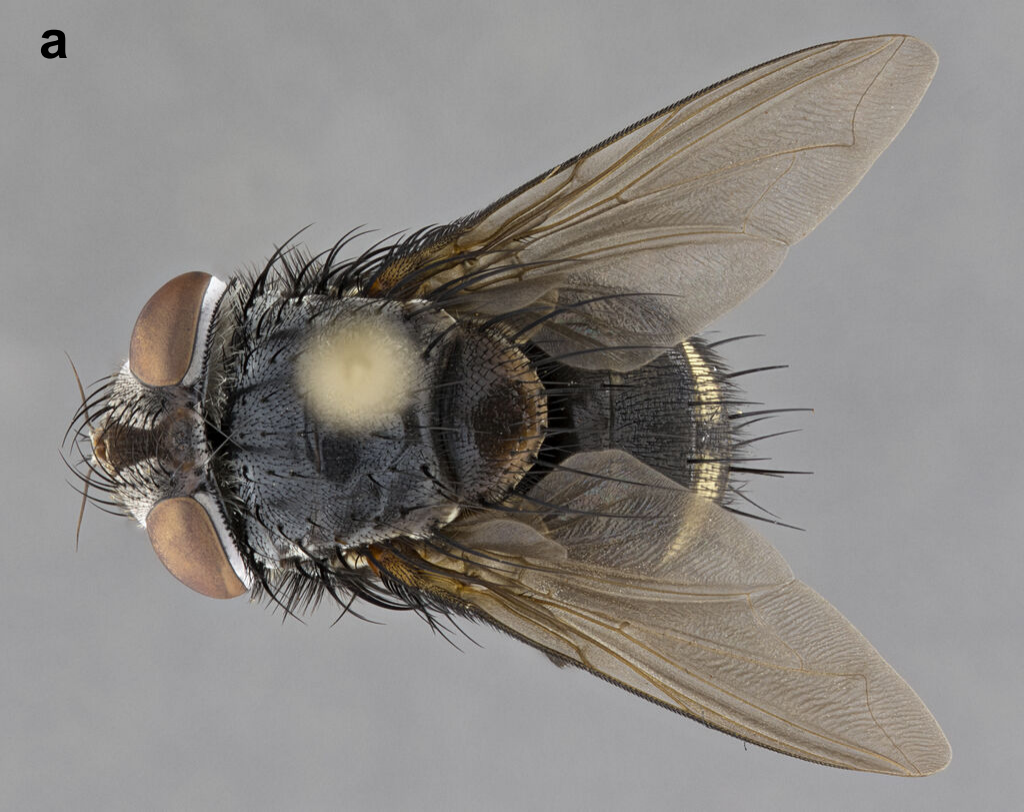
dorsal view

**Figure 16b.**
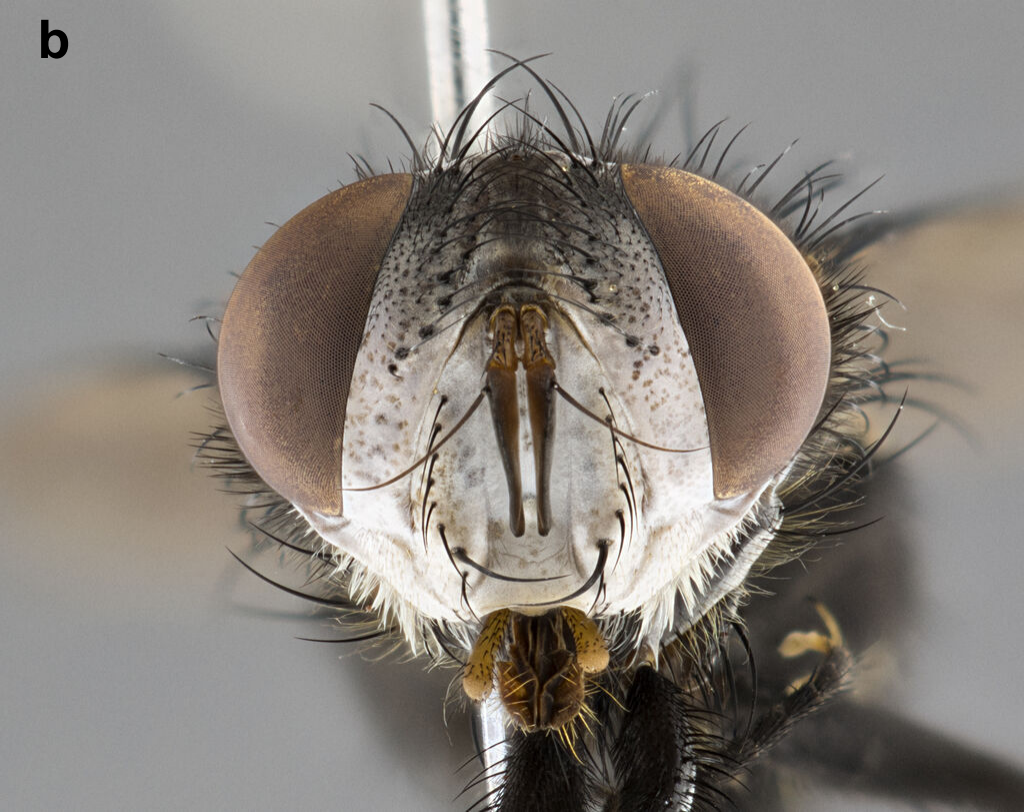
frontal view

**Figure 16c.**
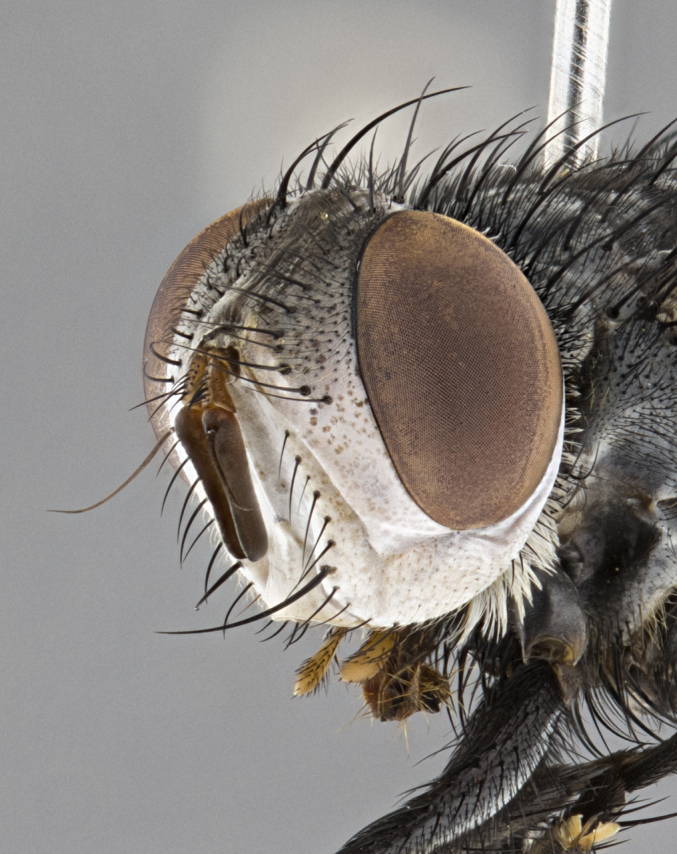
three quarters view

**Figure 16d.**
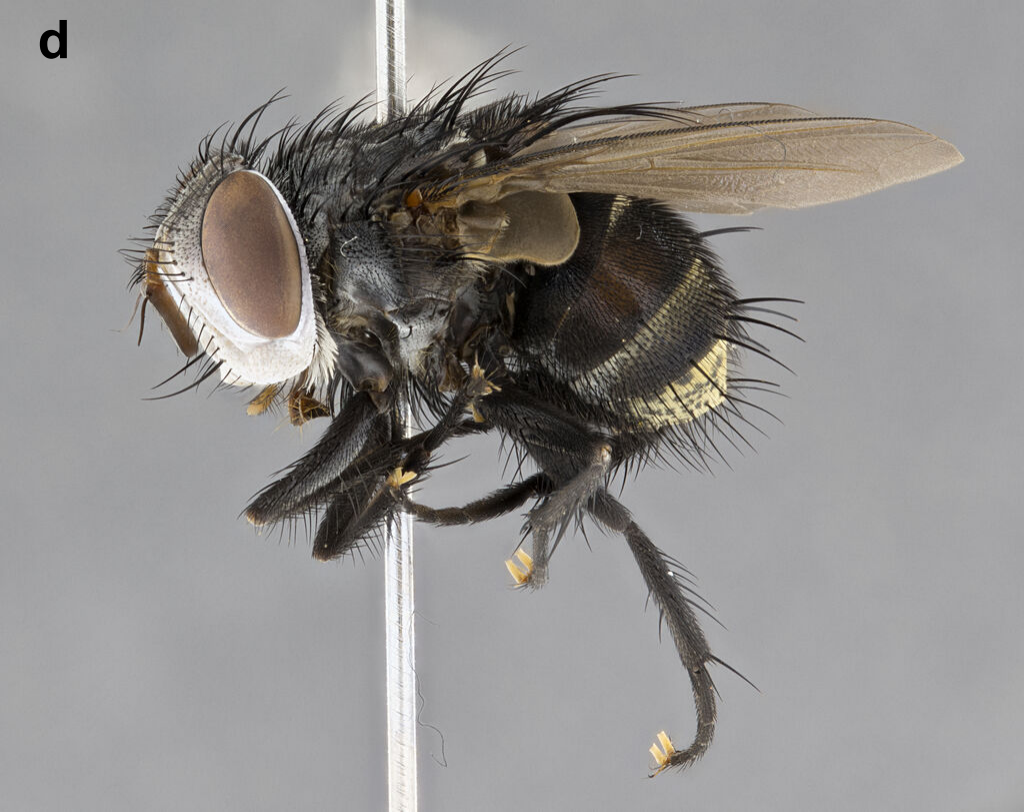
lateral view

**Figure 17a.**
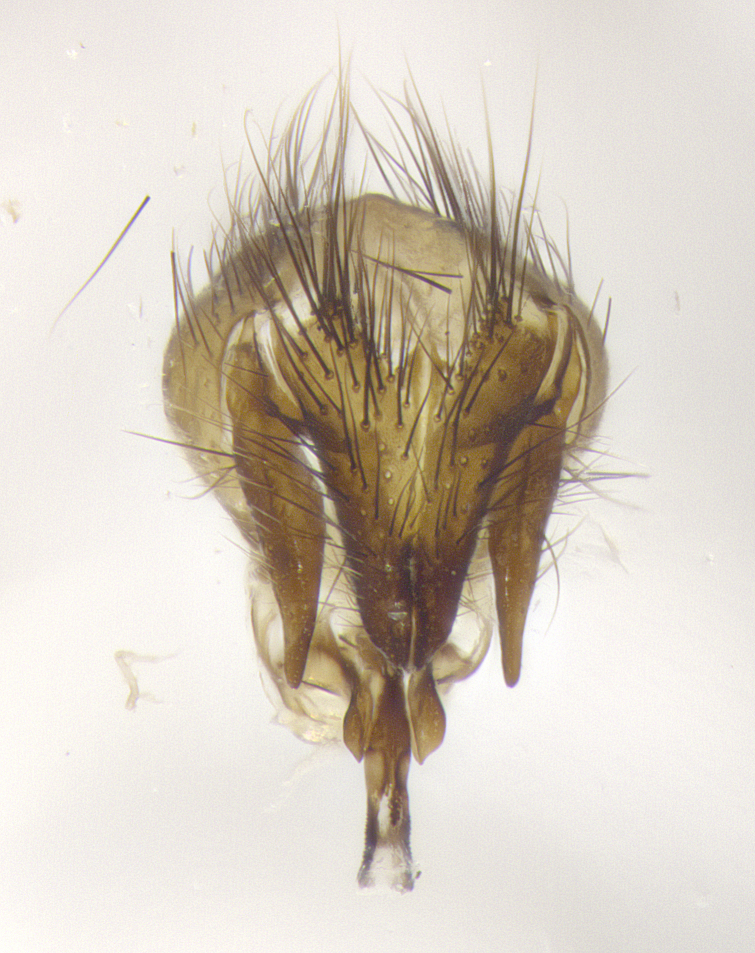
caudal view

**Figure 17b.**
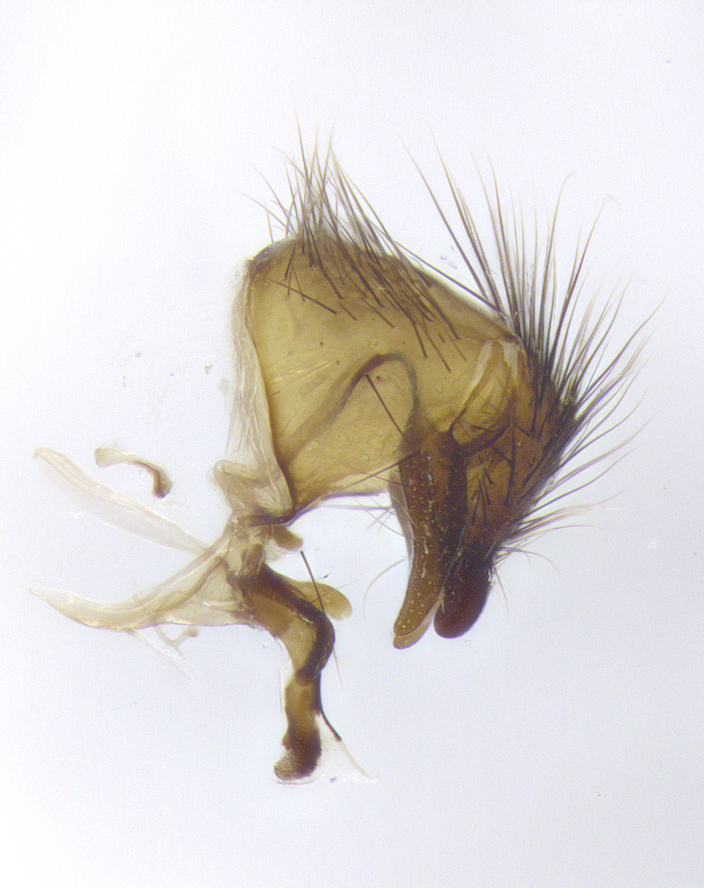
lateral view

**Figure 17c.**
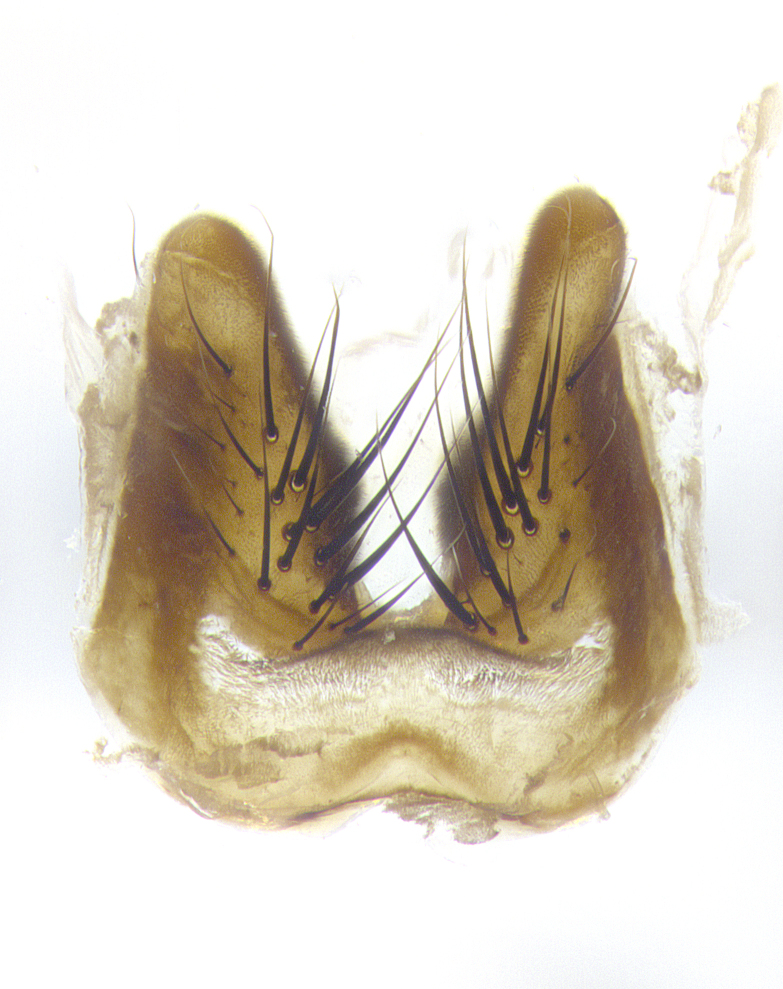
sternite 5, ventral view

**Figure 18a.**
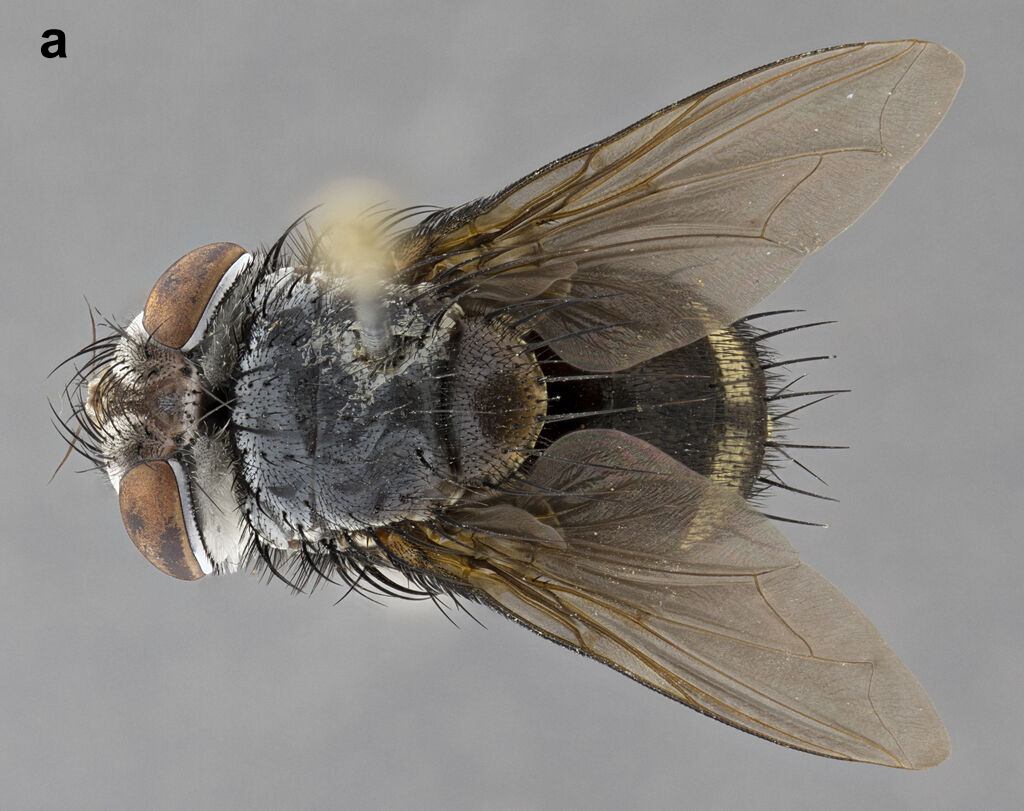
dorsal view

**Figure 18b.**
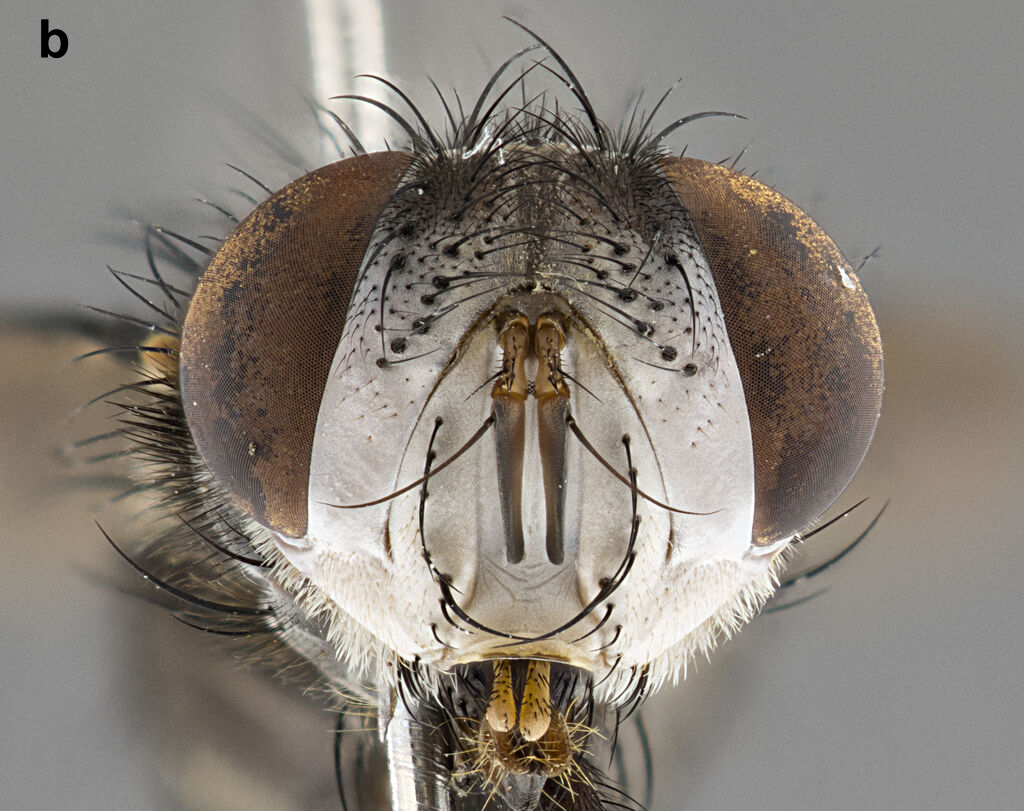
frontal view

**Figure 18c.**
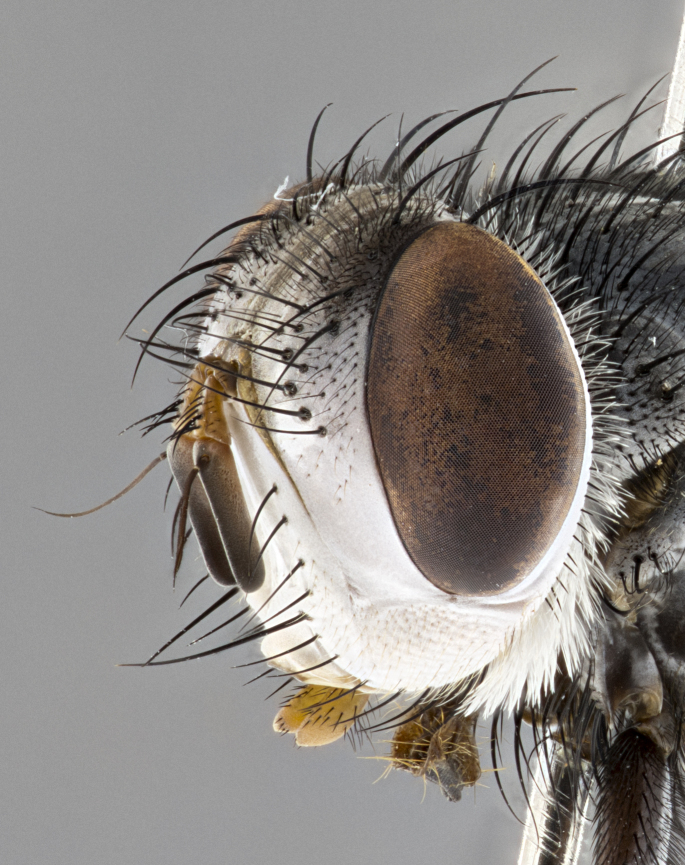
three quarters view

**Figure 18d.**
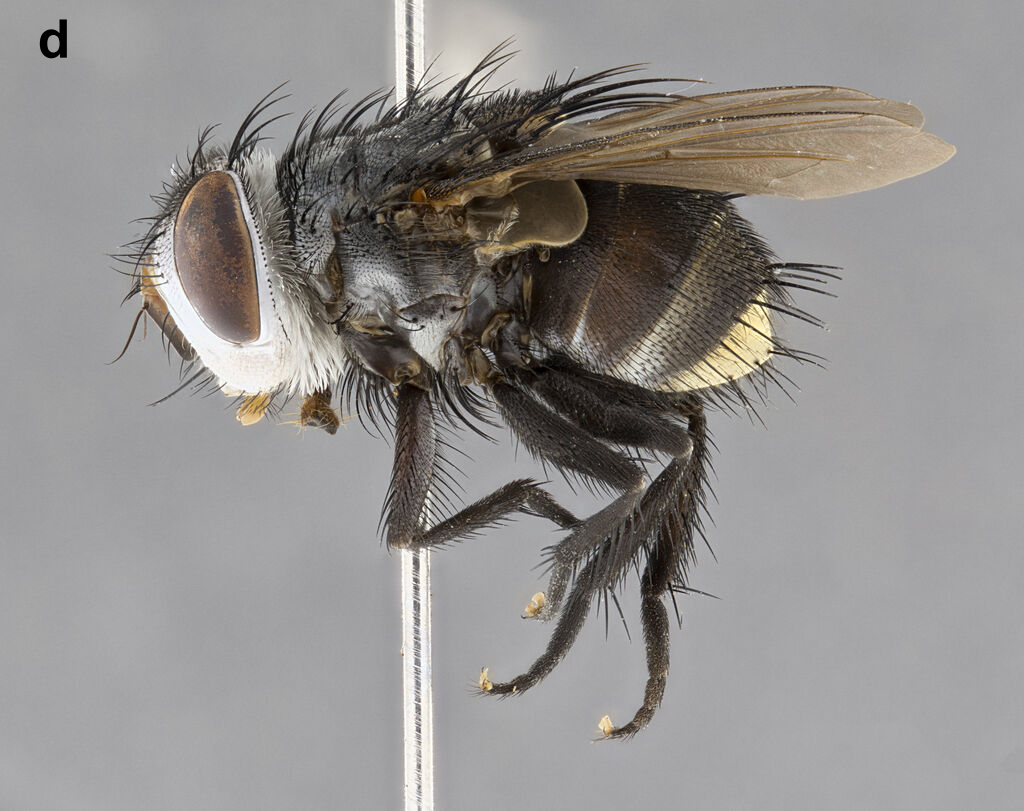
lateral view

**Figure 19a.**
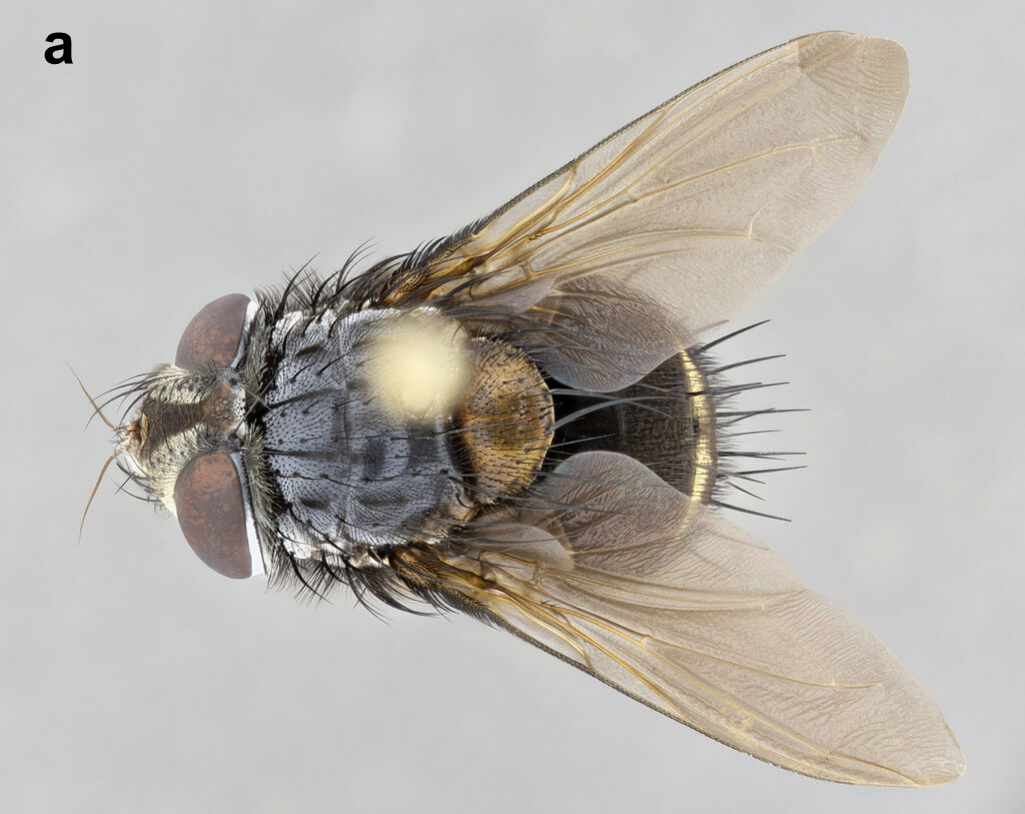
dorsal view

**Figure 19b.**
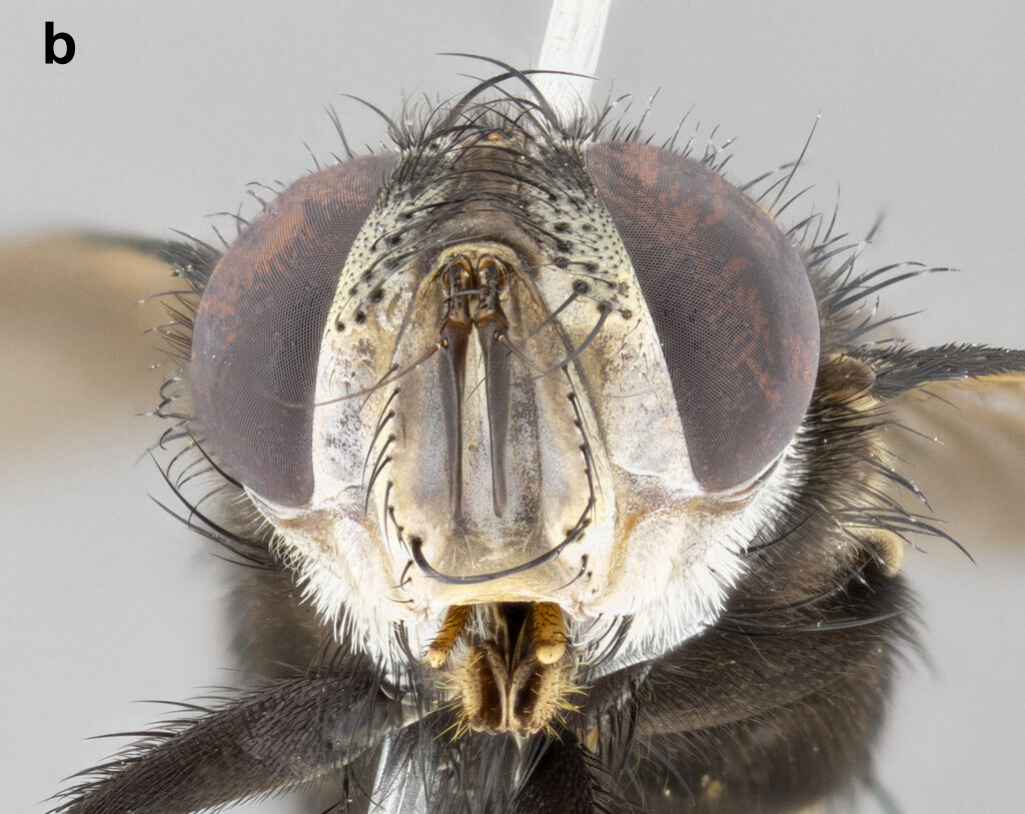
frontal view

**Figure 19c.**
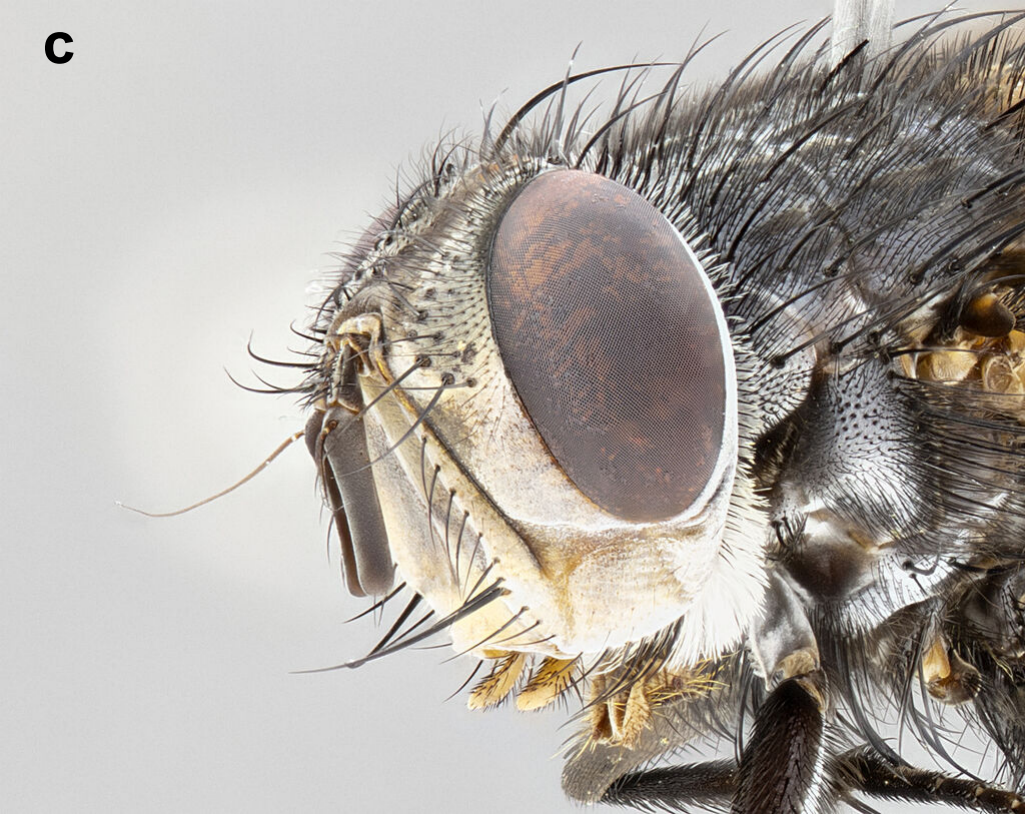
three quarters view

**Figure 19d.**
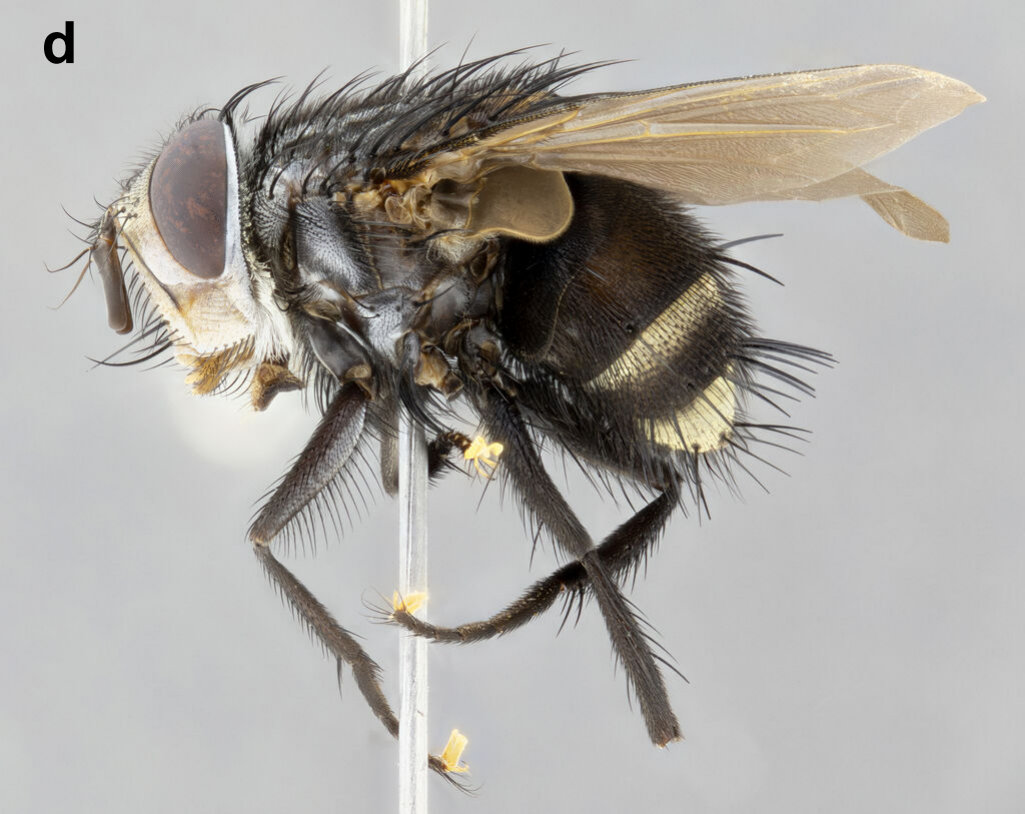
lateral view

**Figure 20a.**
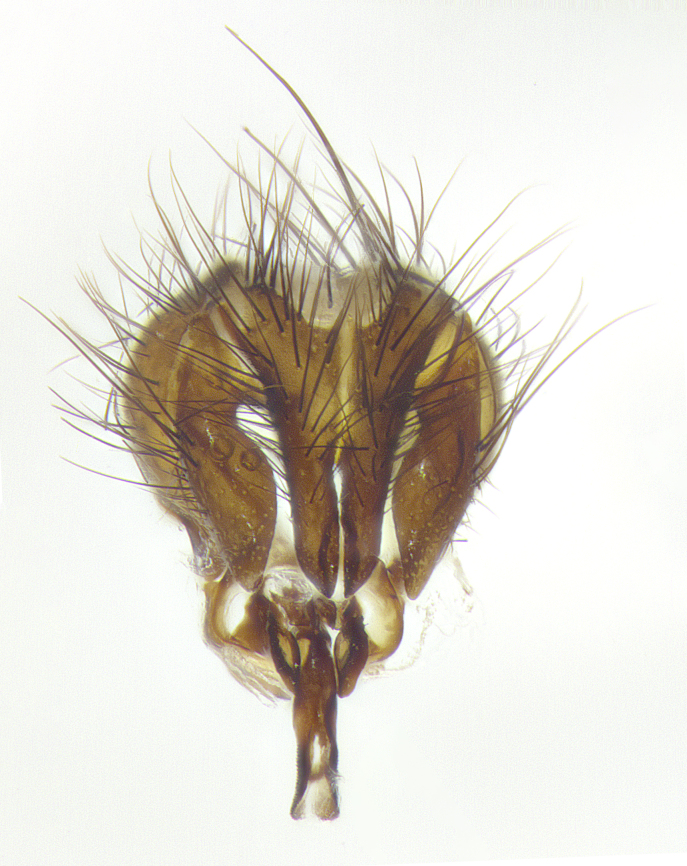
caudal view

**Figure 20b.**
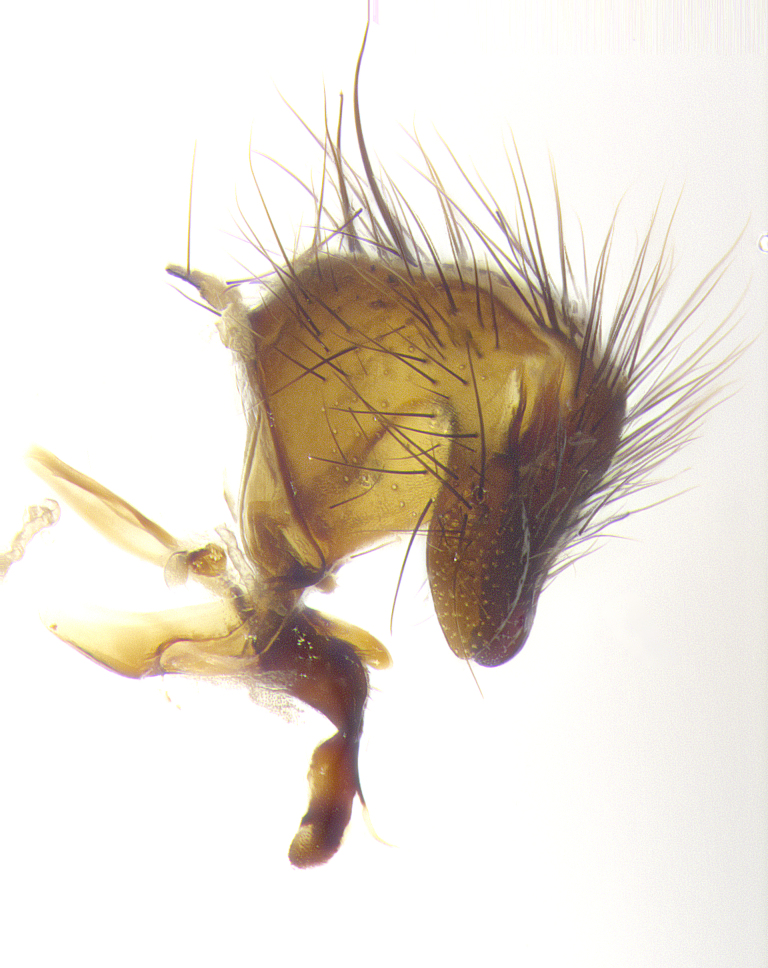
lateral view

**Figure 20c.**
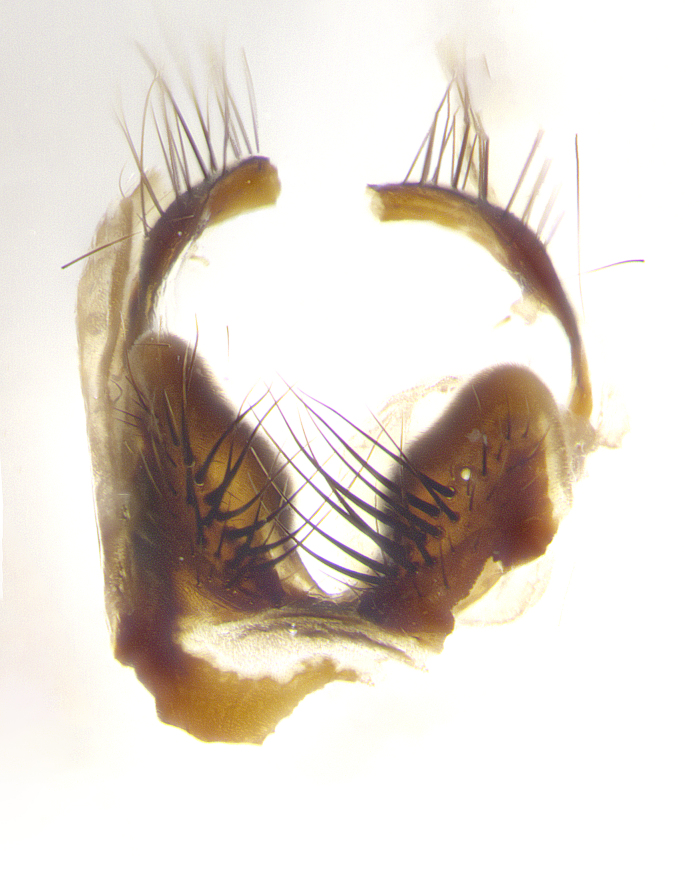
sternite 5, ventral view

**Figure 21a.**
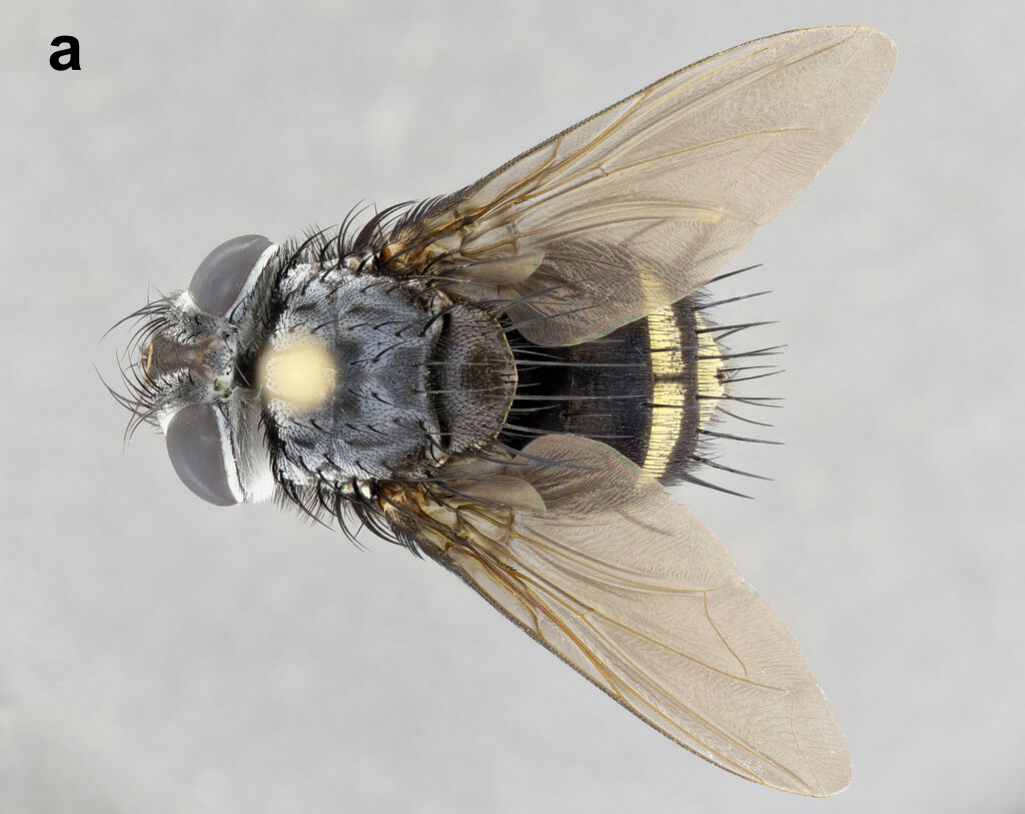
dorsal view

**Figure 21b.**
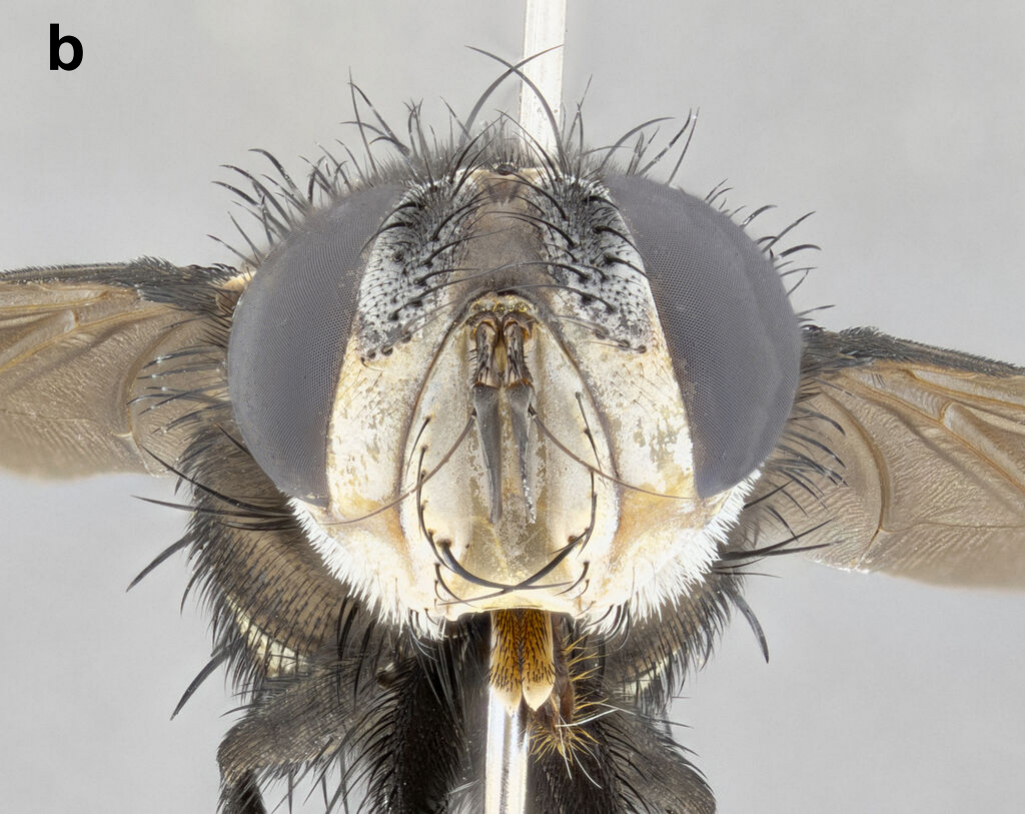
frontal view

**Figure 21c.**
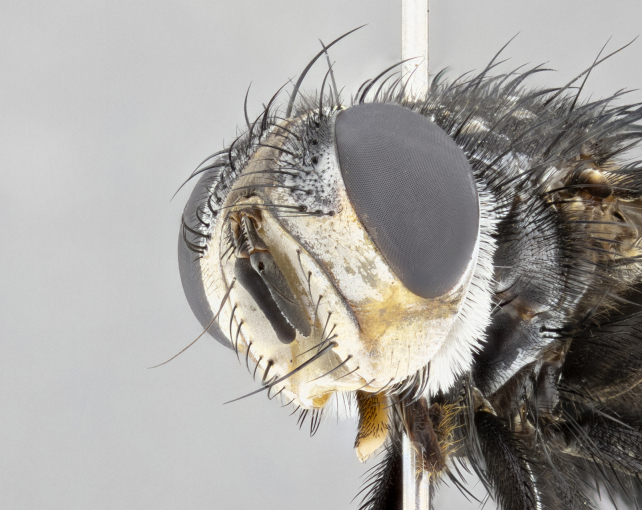
three quarters view

**Figure 21d.**
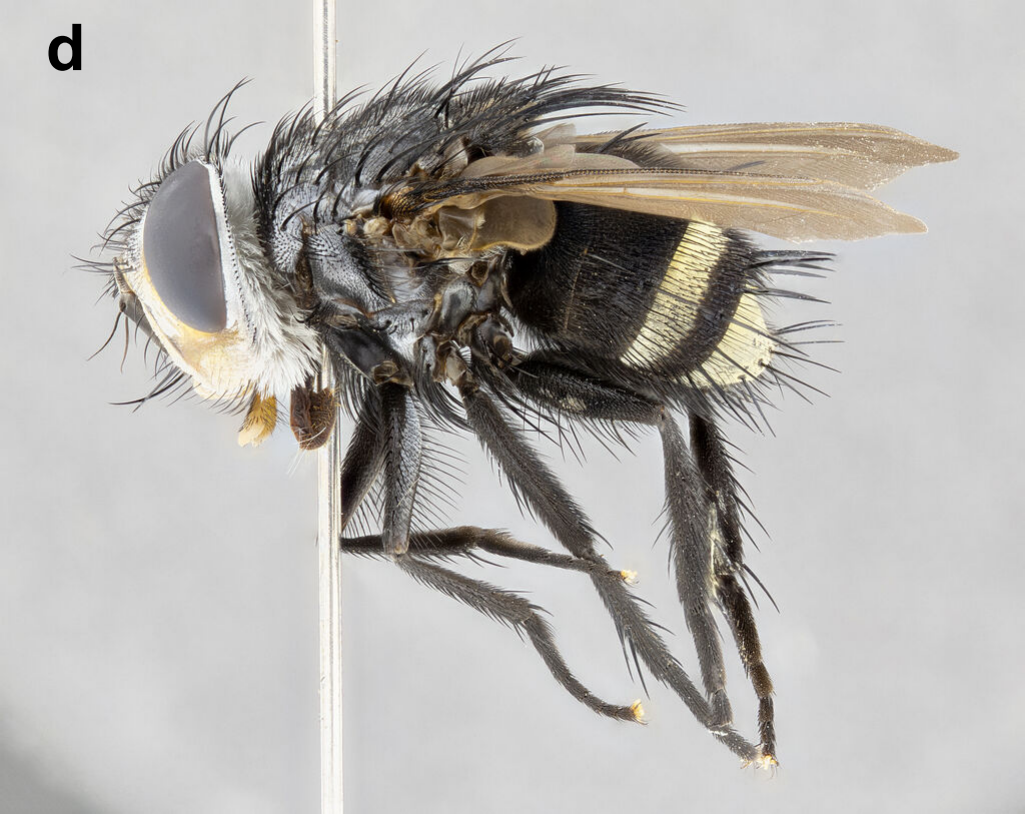
lateral view

**Figure 22a.**
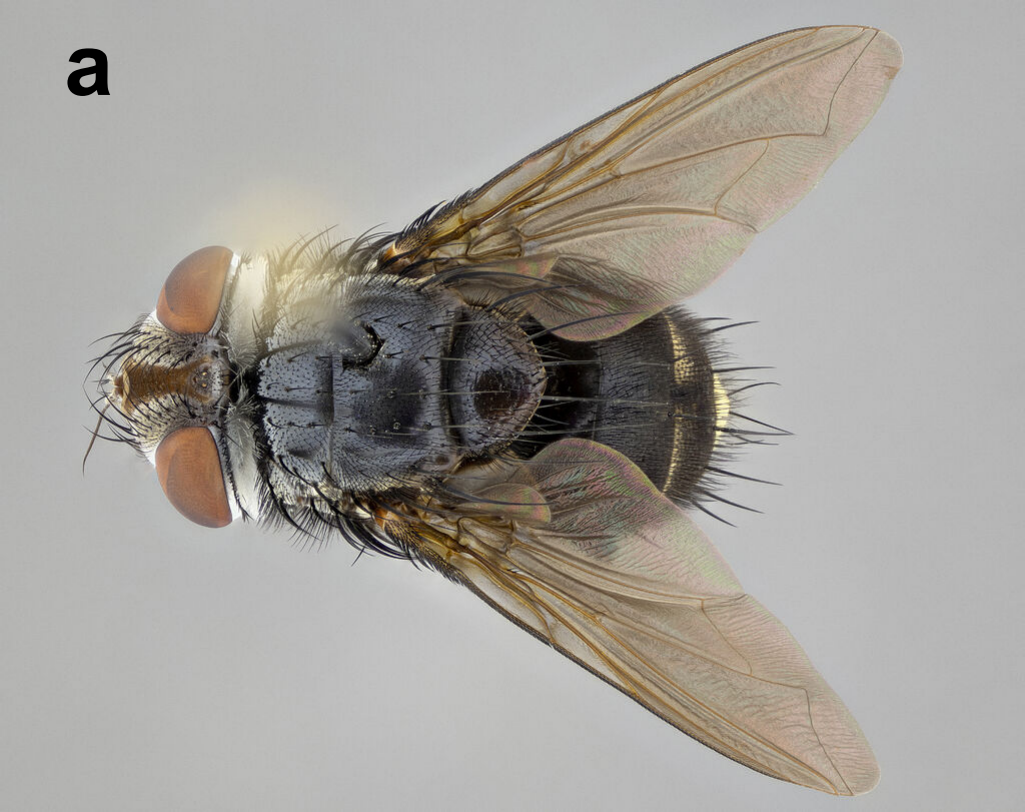
dorsal view

**Figure 22b.**
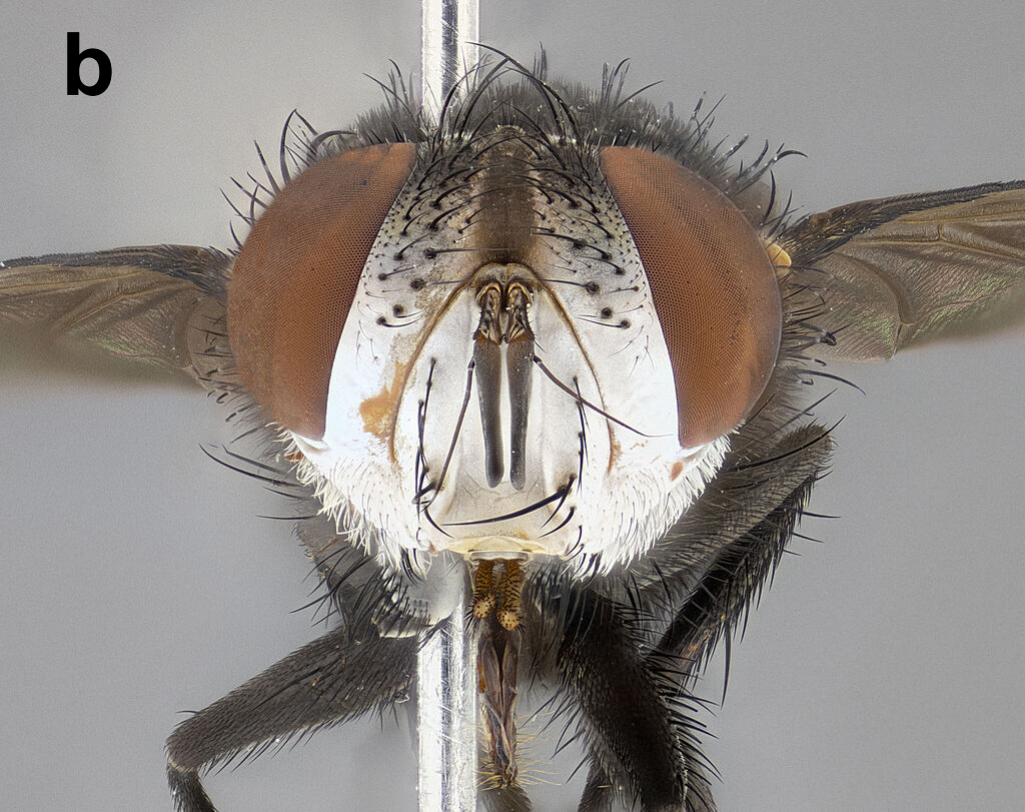
frontal view

**Figure 22c.**
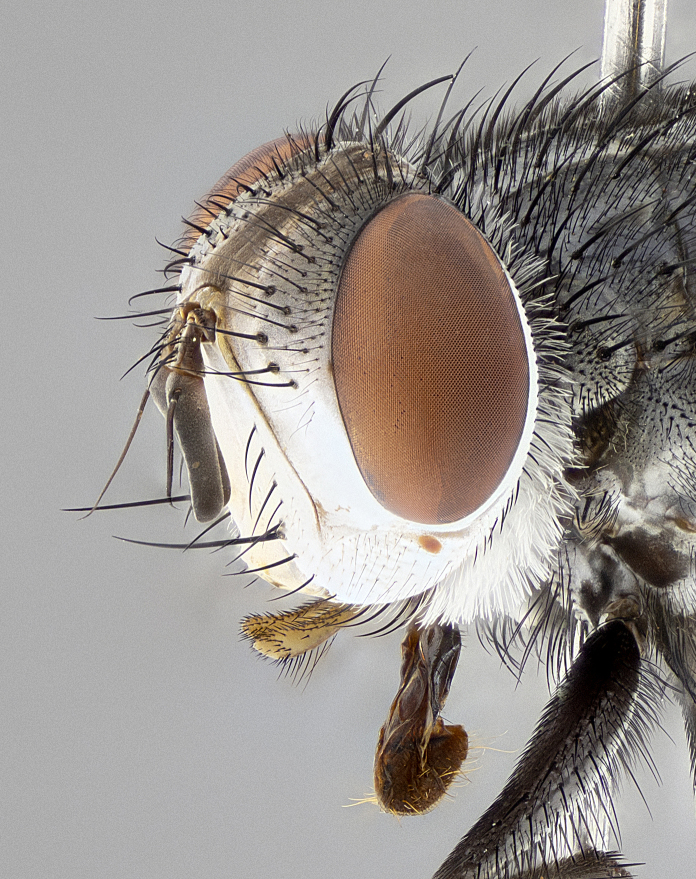
three quarters view

**Figure 22d.**
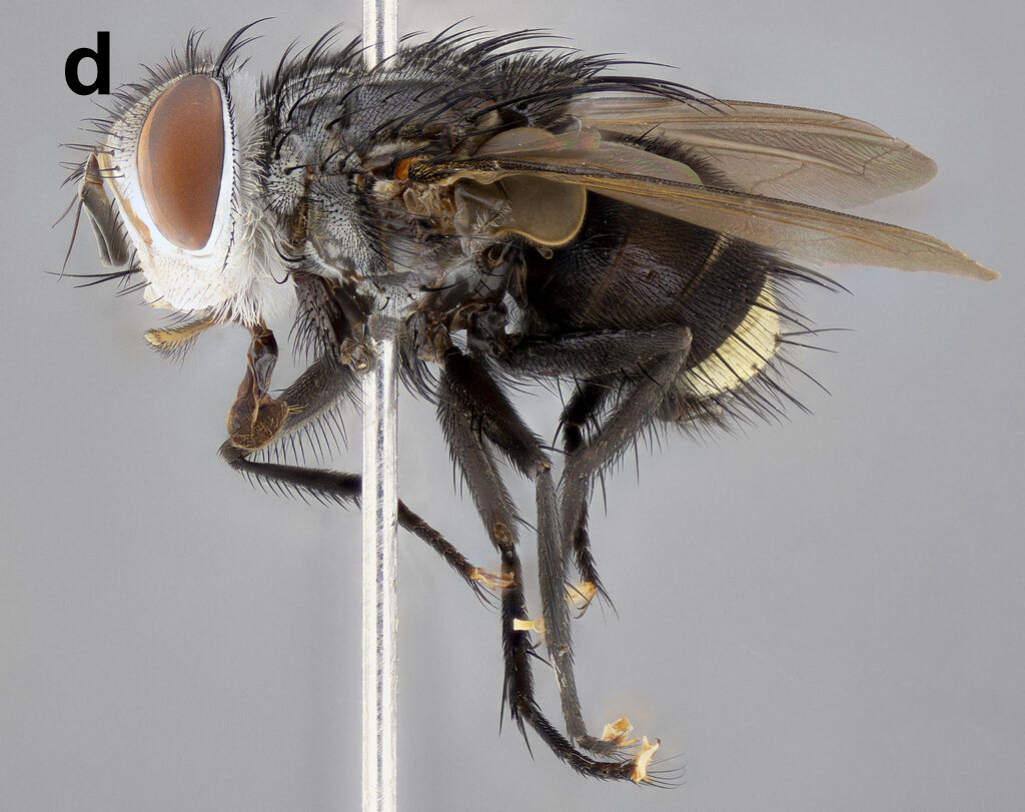
lateral view

**Figure 23a.**
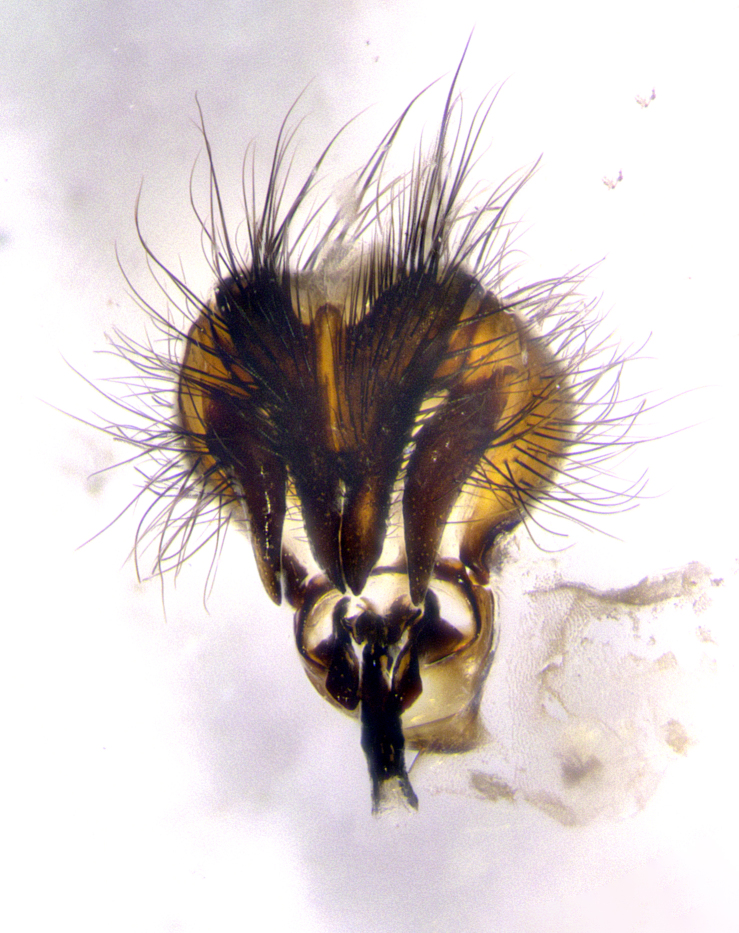
caudal view

**Figure 23b.**
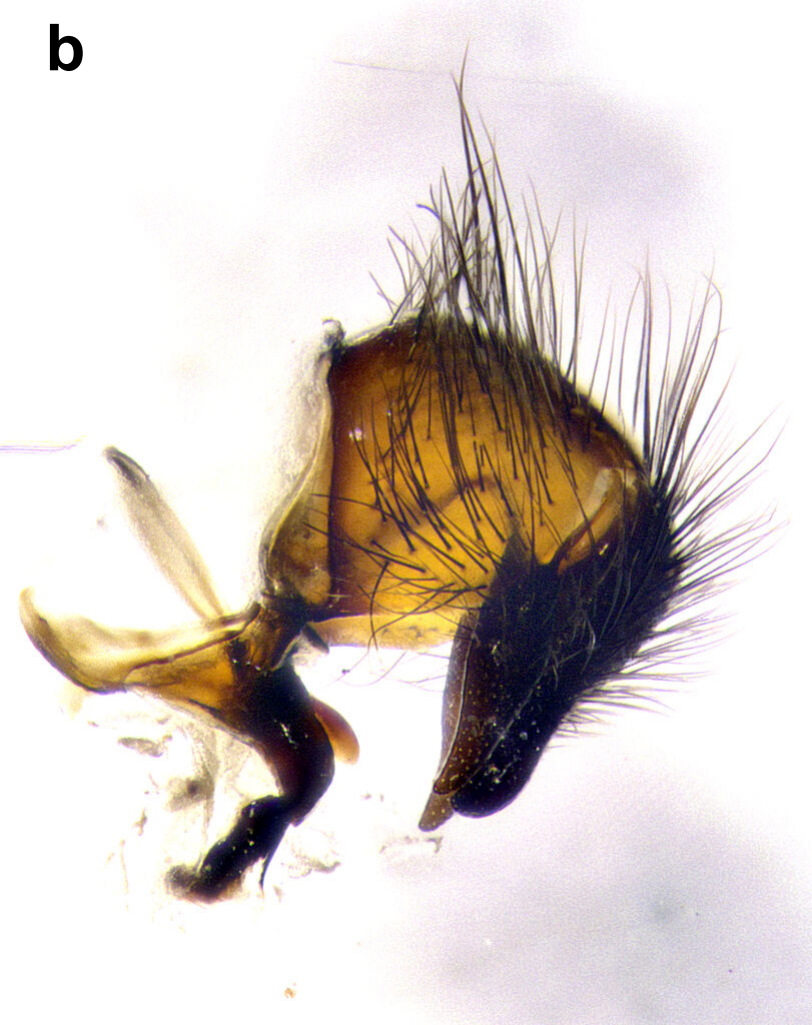
lateral view

**Figure 23c.**
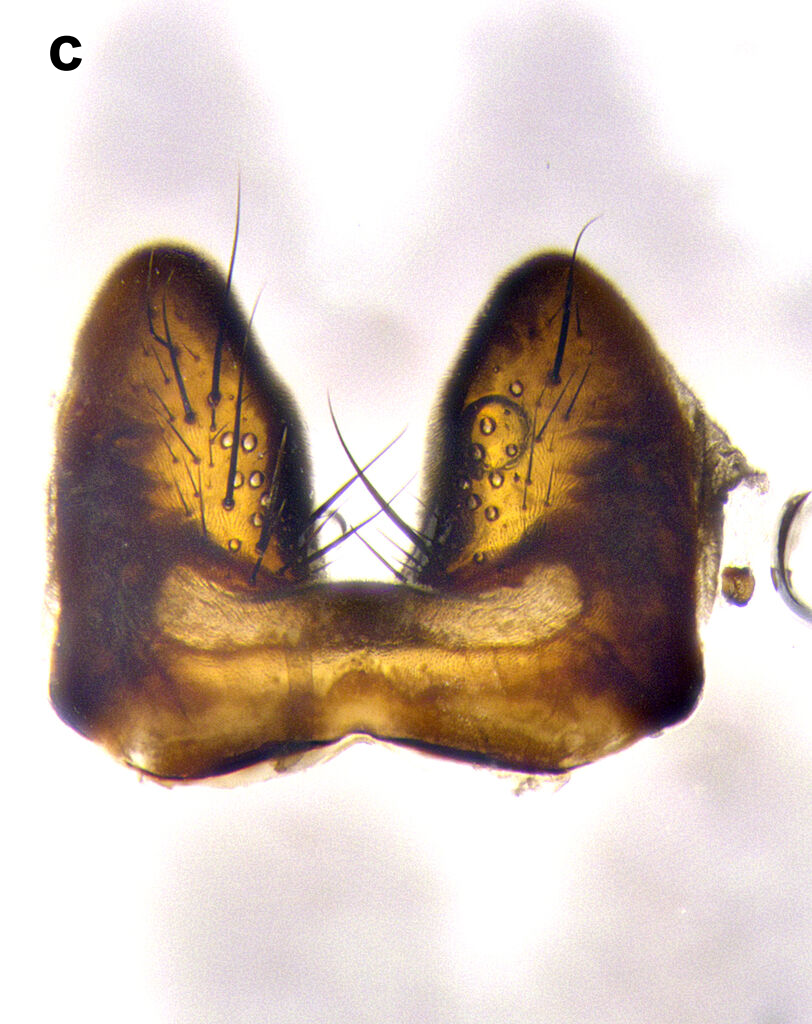
sternite 5, ventral view

**Figure 24a.**
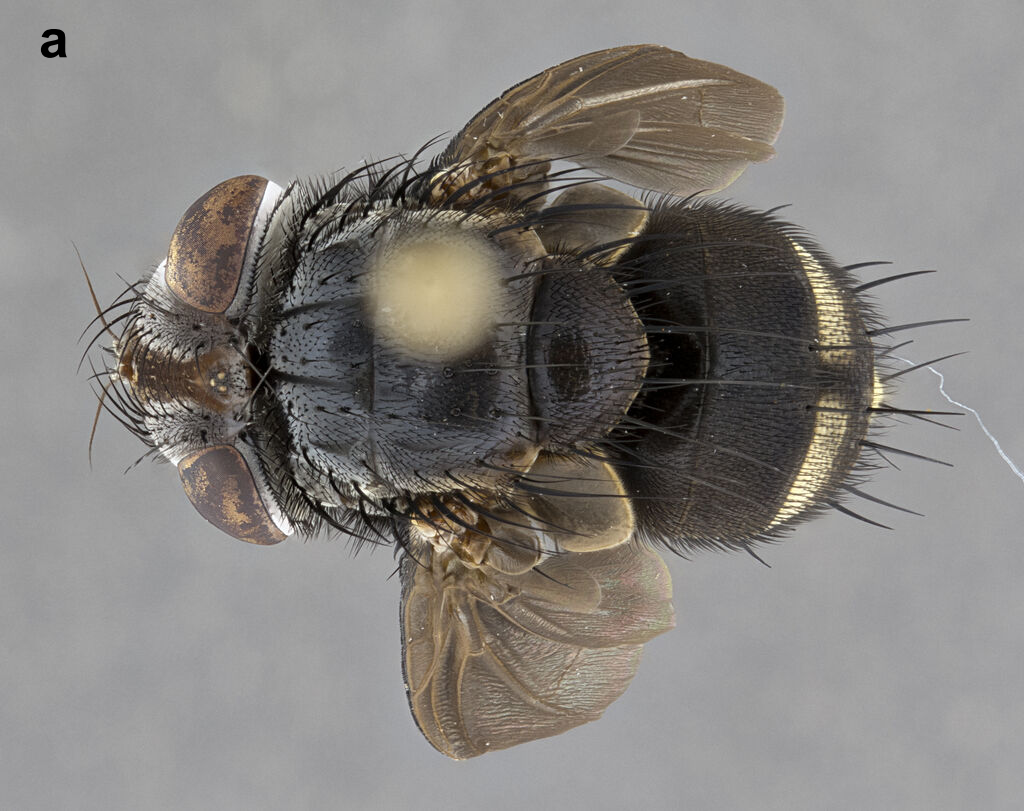
dorsal view

**Figure 24b.**
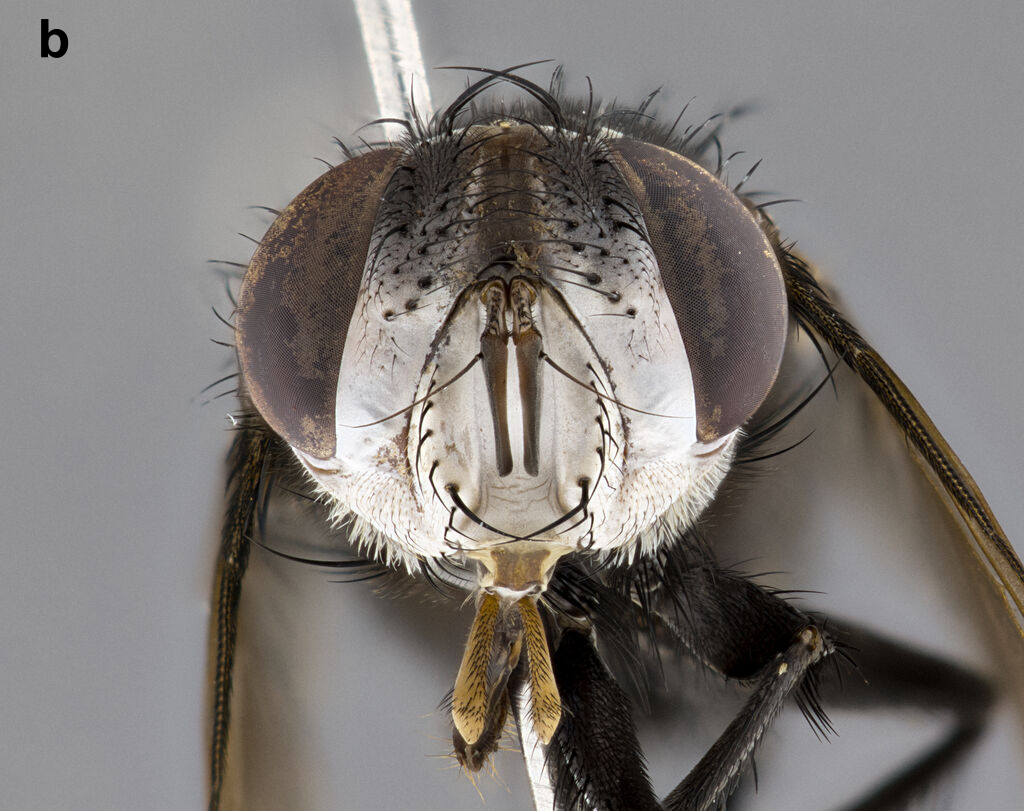
frontal view

**Figure 24c.**
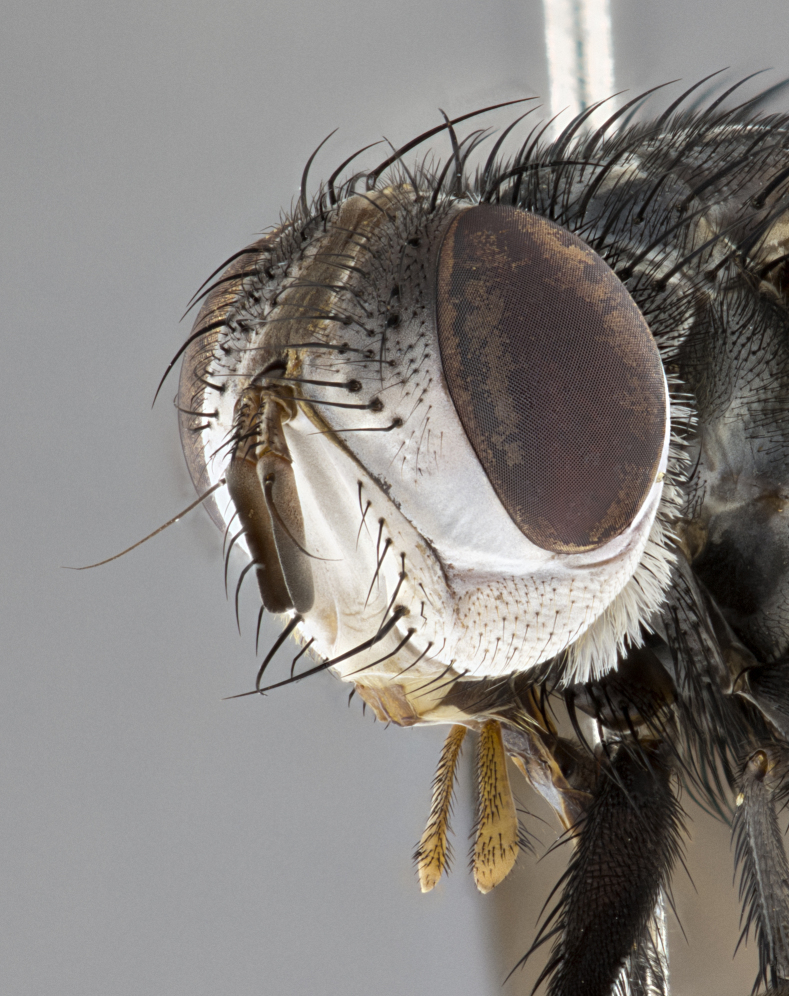
three quarters view

**Figure 24d.**
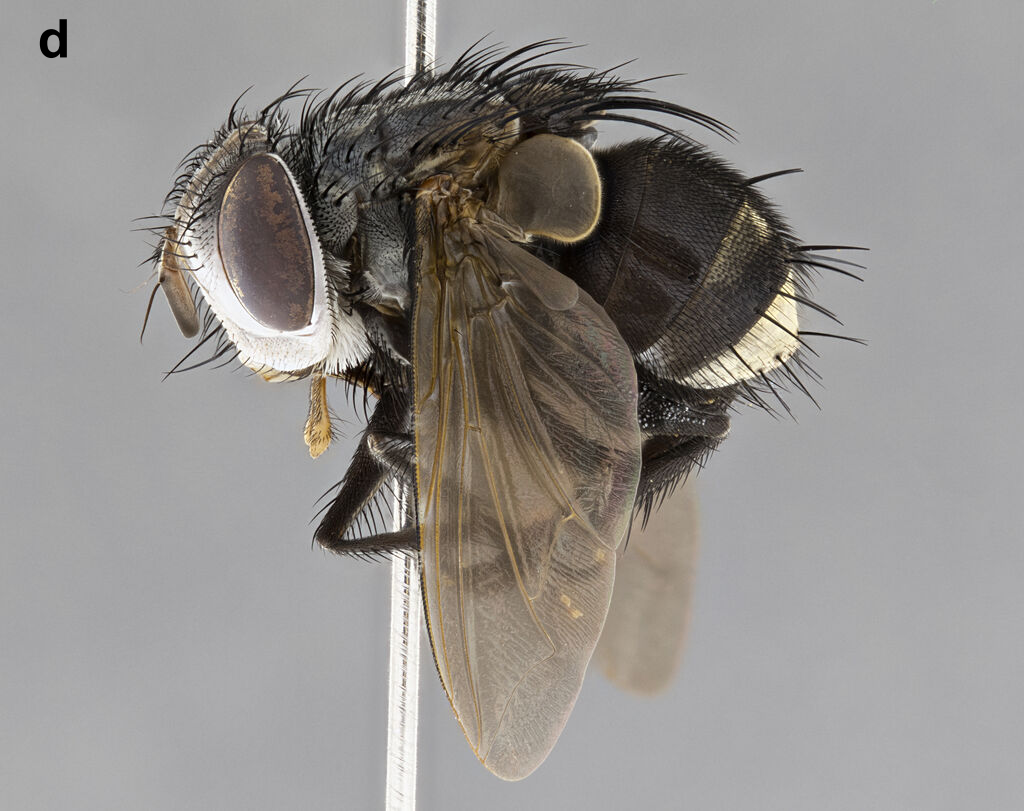
lateral view

**Figure 25a.**
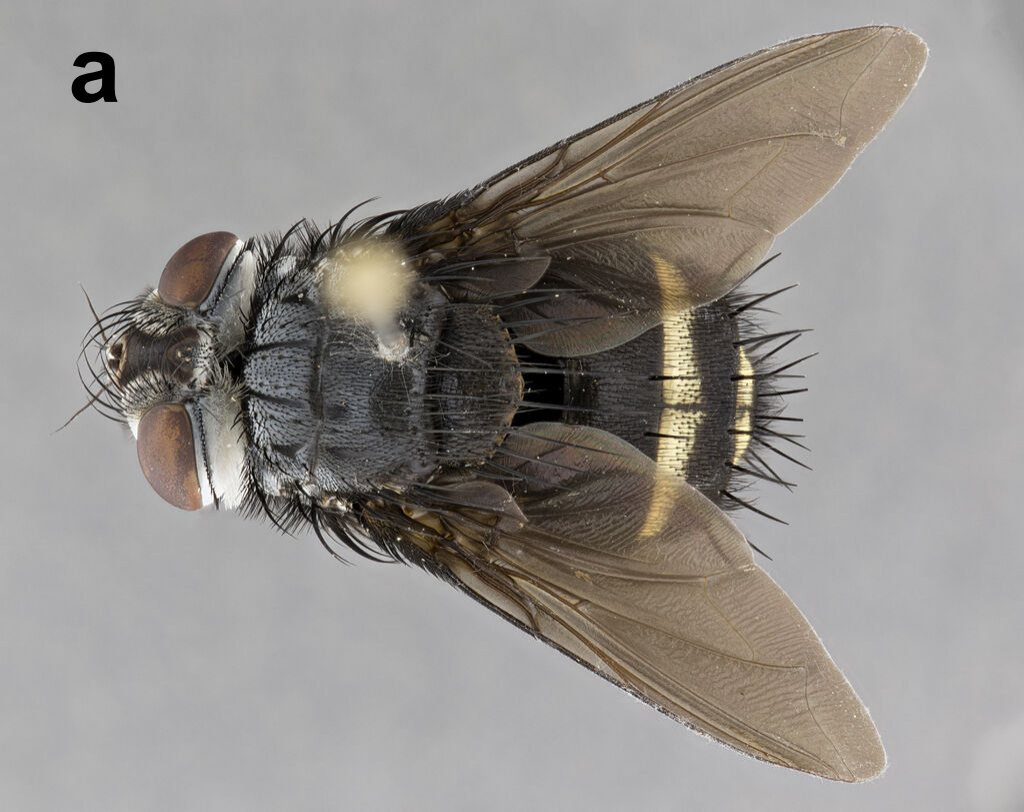
dorsal view

**Figure 25b.**
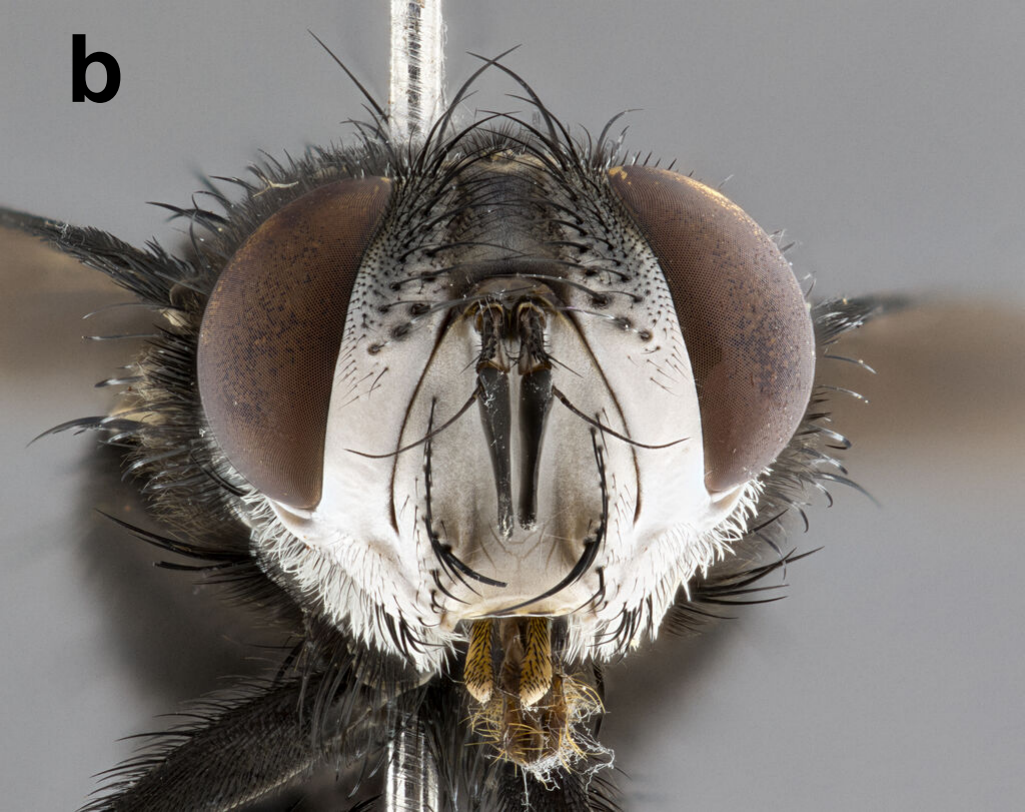
frontal view

**Figure 25c.**
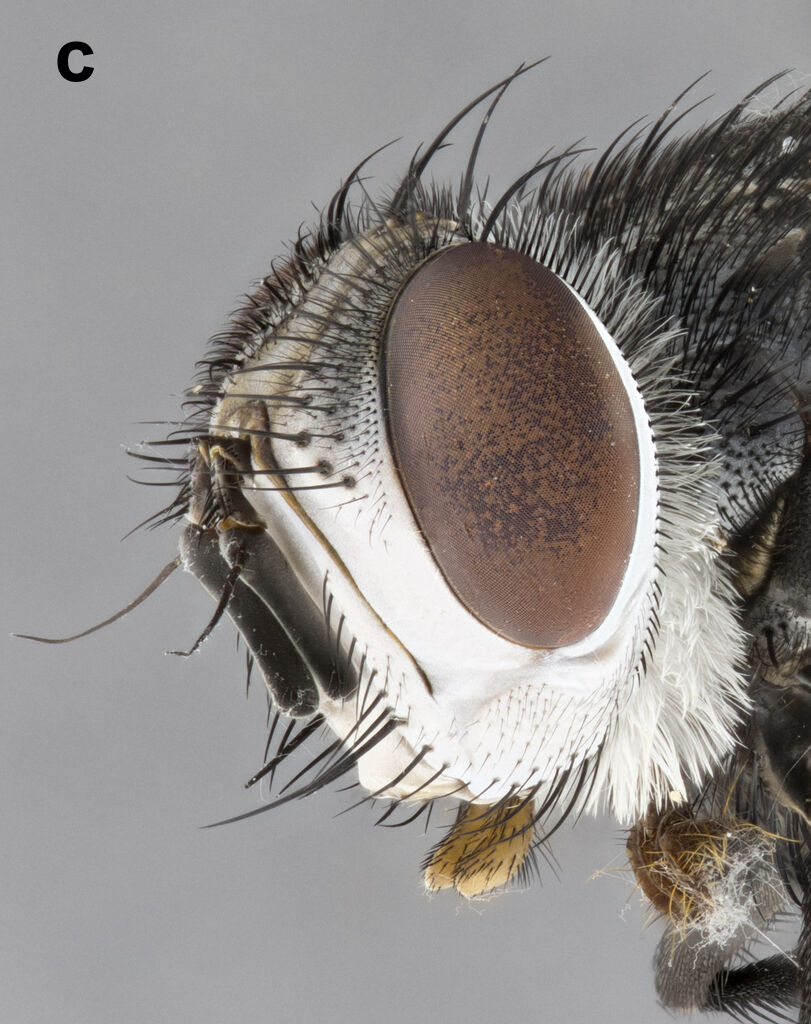
three quarters view

**Figure 25d.**
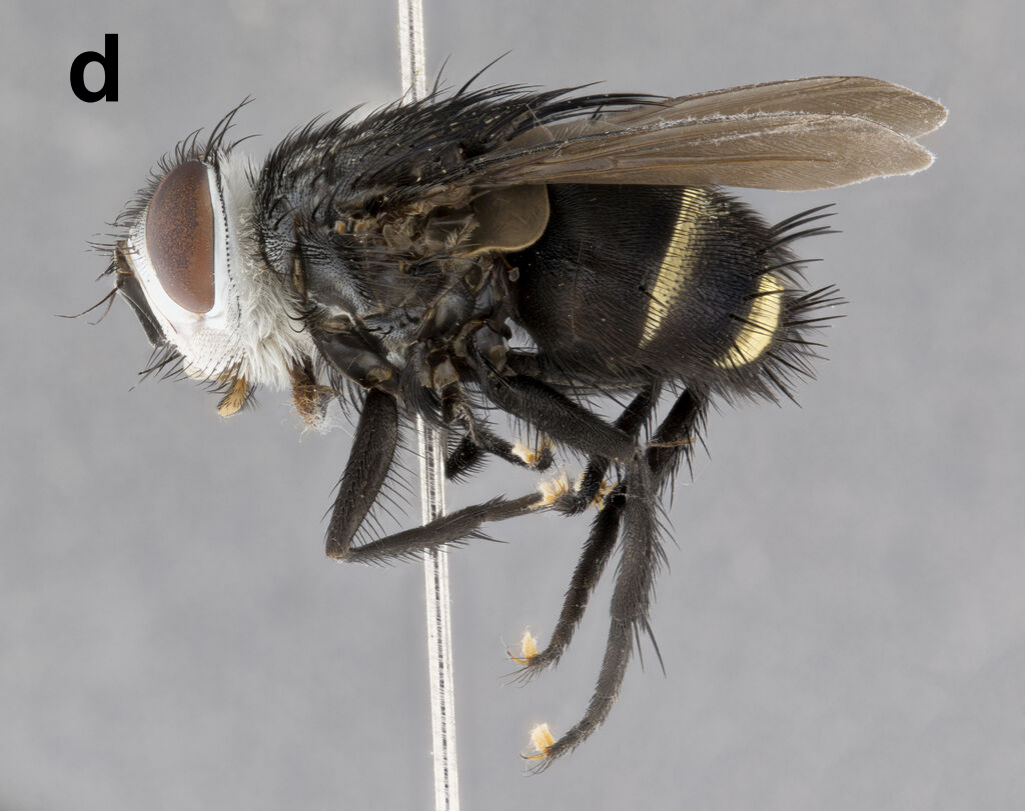
lateral view

**Figure 26a.**
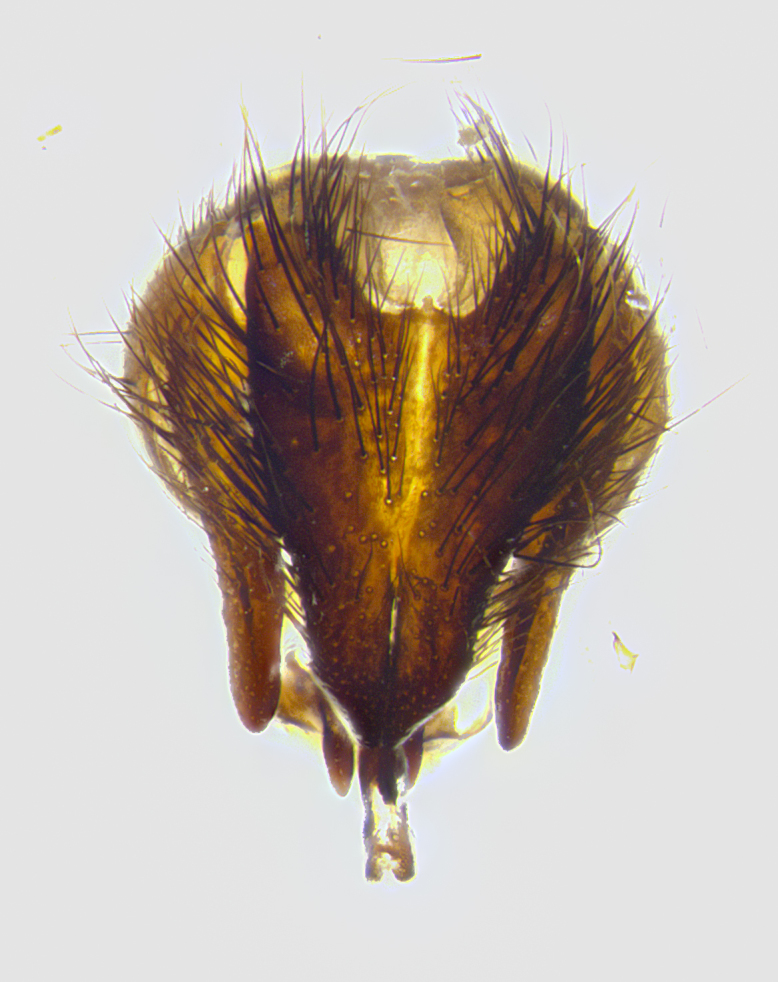
caudal view

**Figure 26b.**
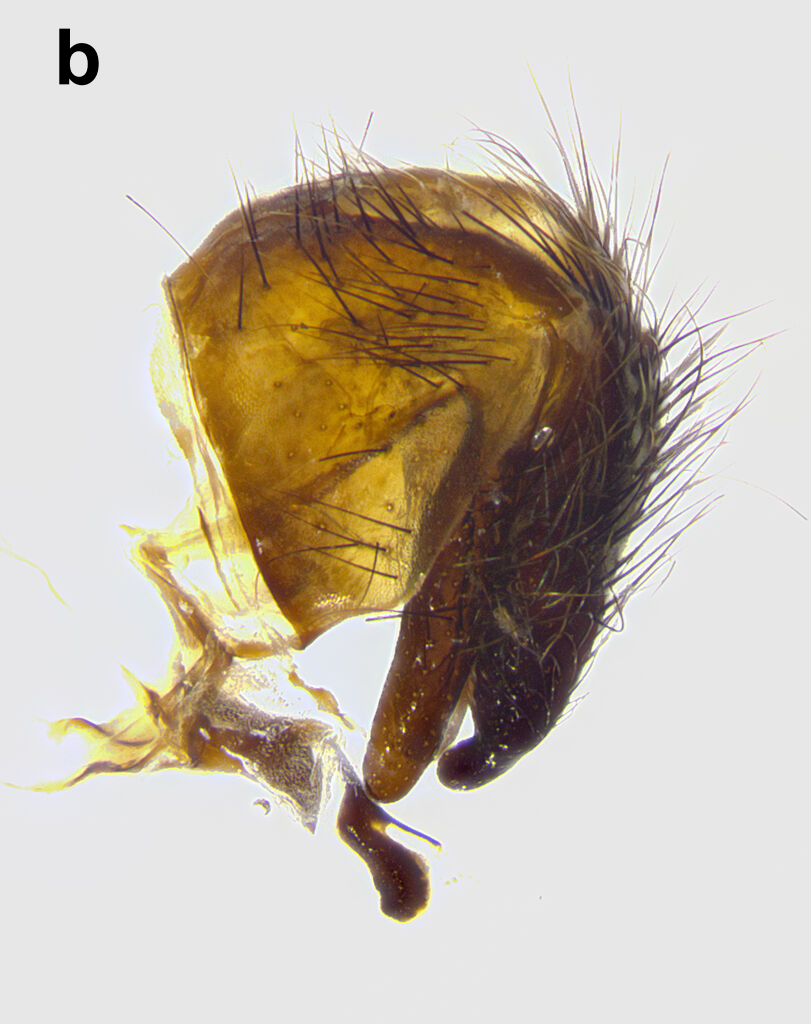
lateral view

**Figure 26c.**
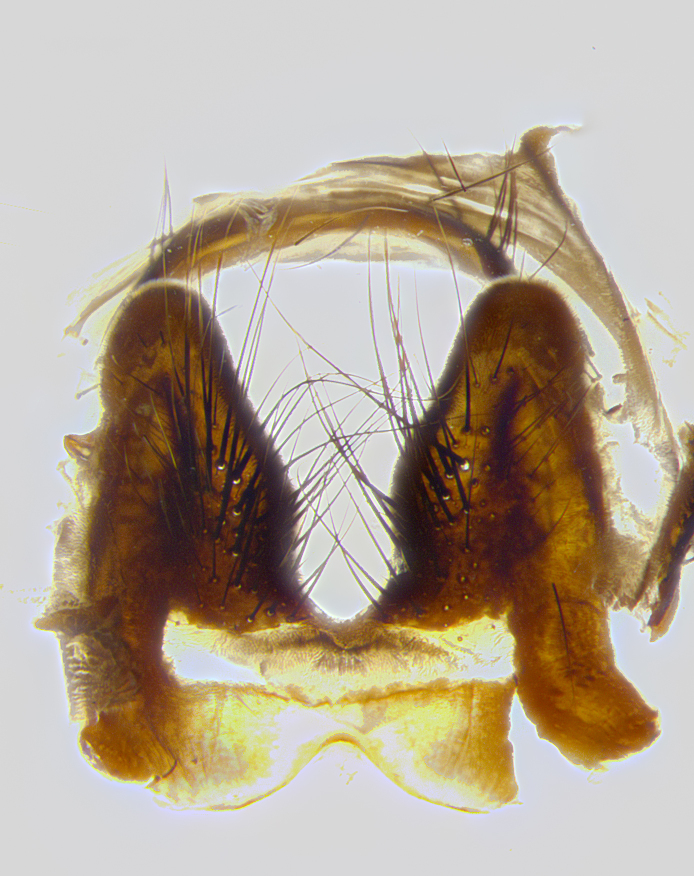
sternite 5, ventral view

**Figure 27a.**
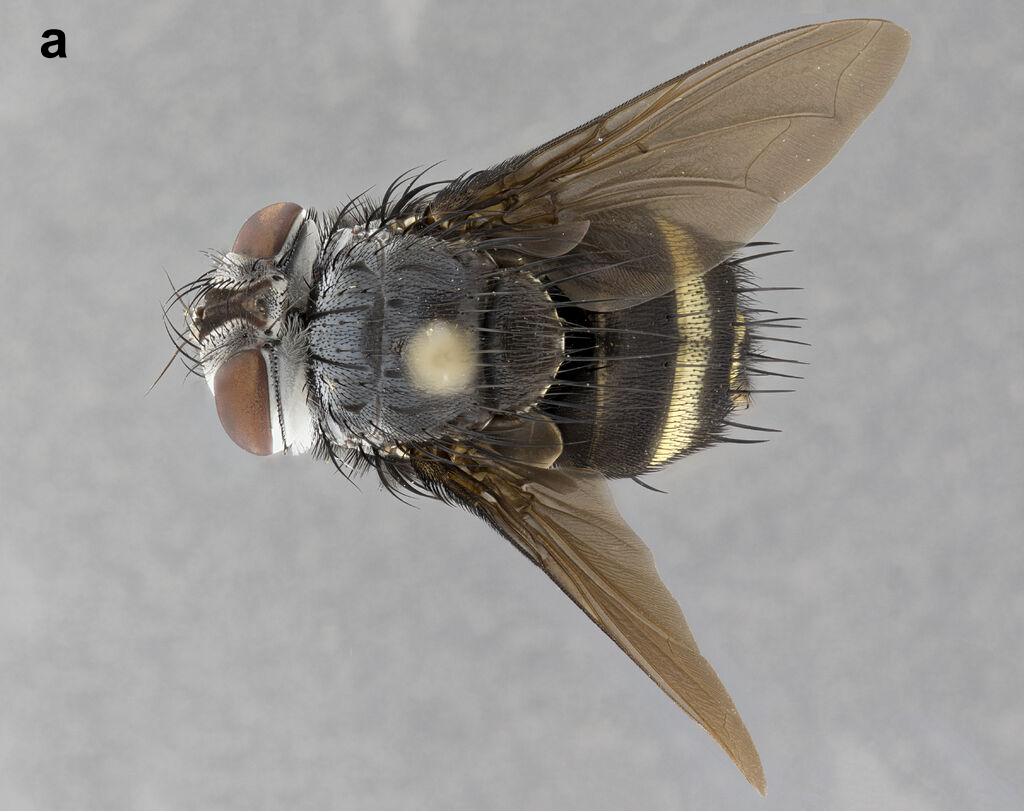
dorsal view

**Figure 27b.**
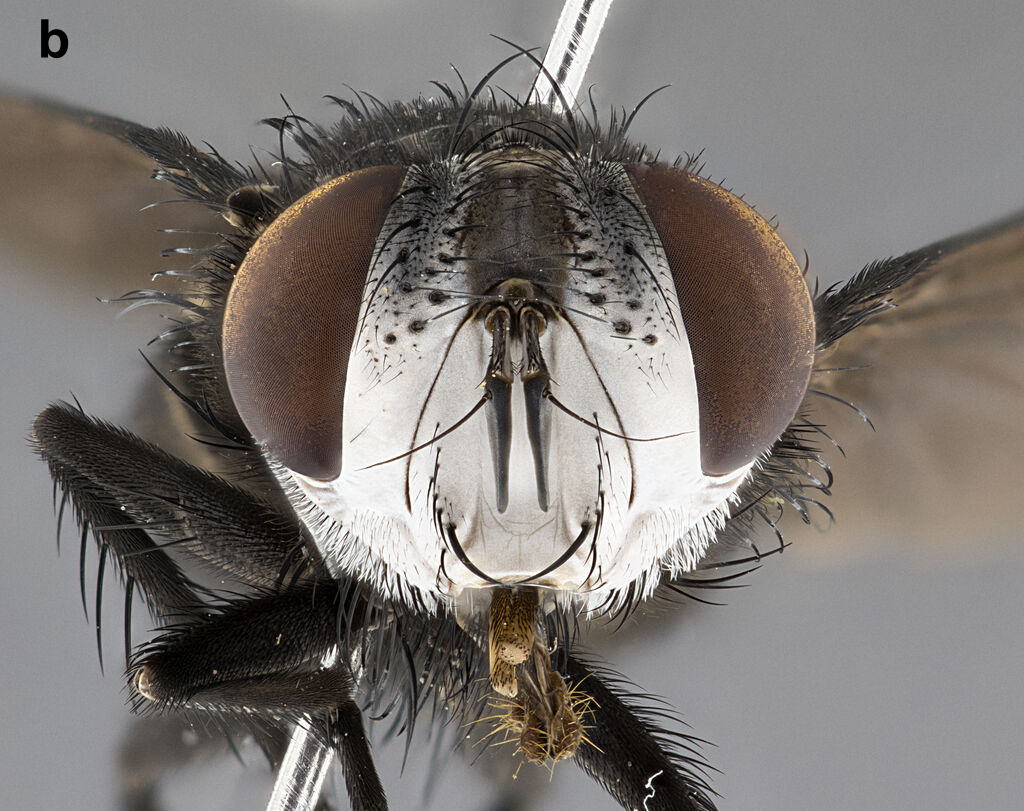
frontal view

**Figure 27c.**
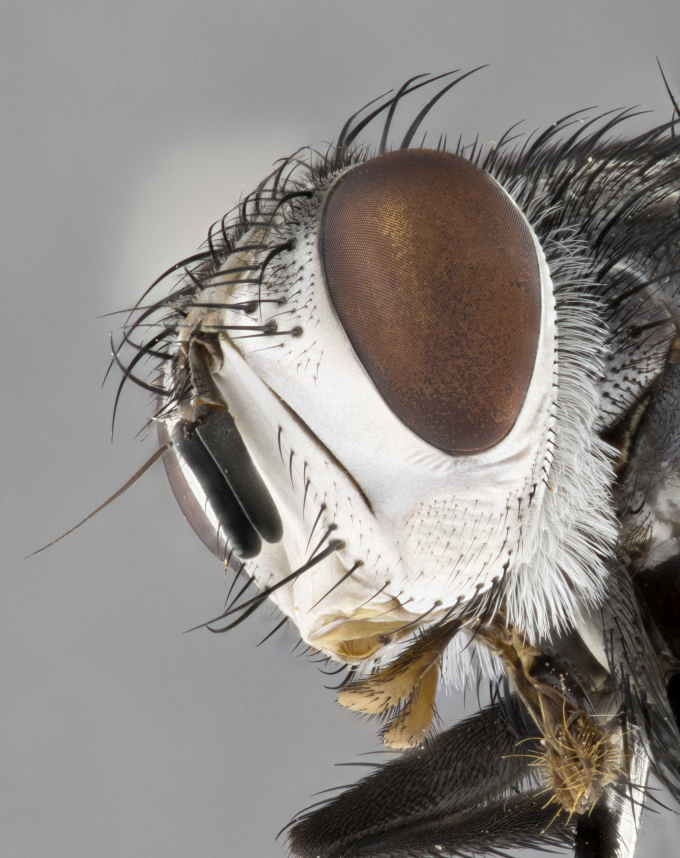
three quarters view

**Figure 27d.**
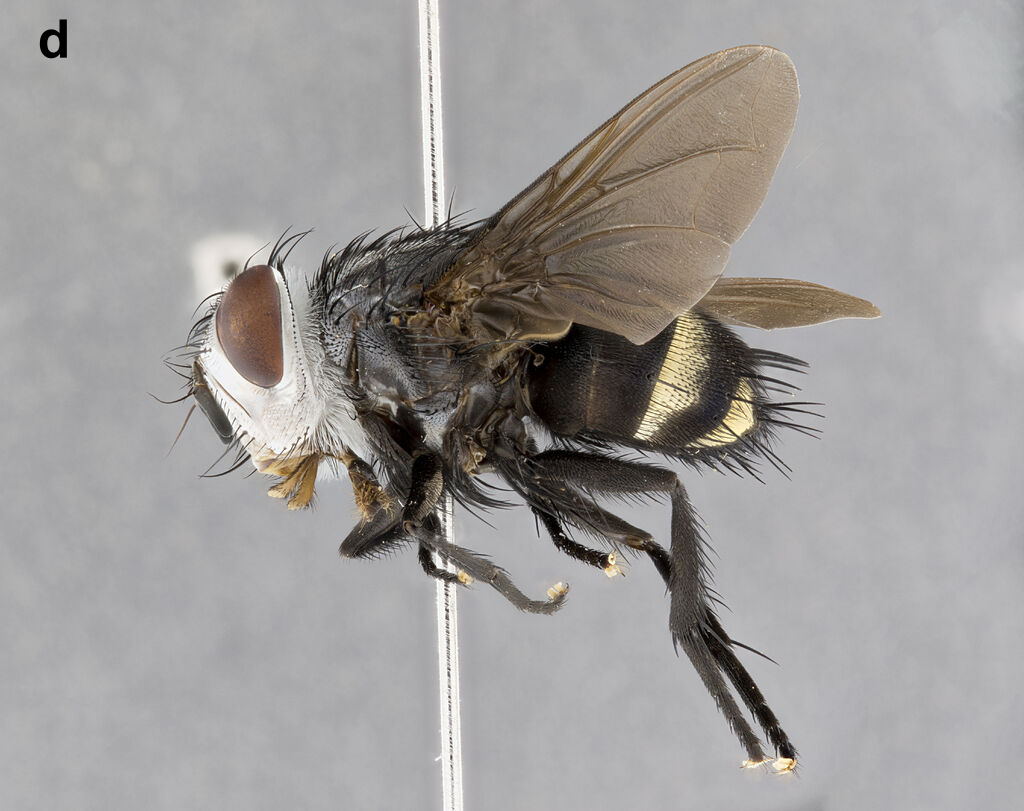
lateral view

**Figure 28a.**
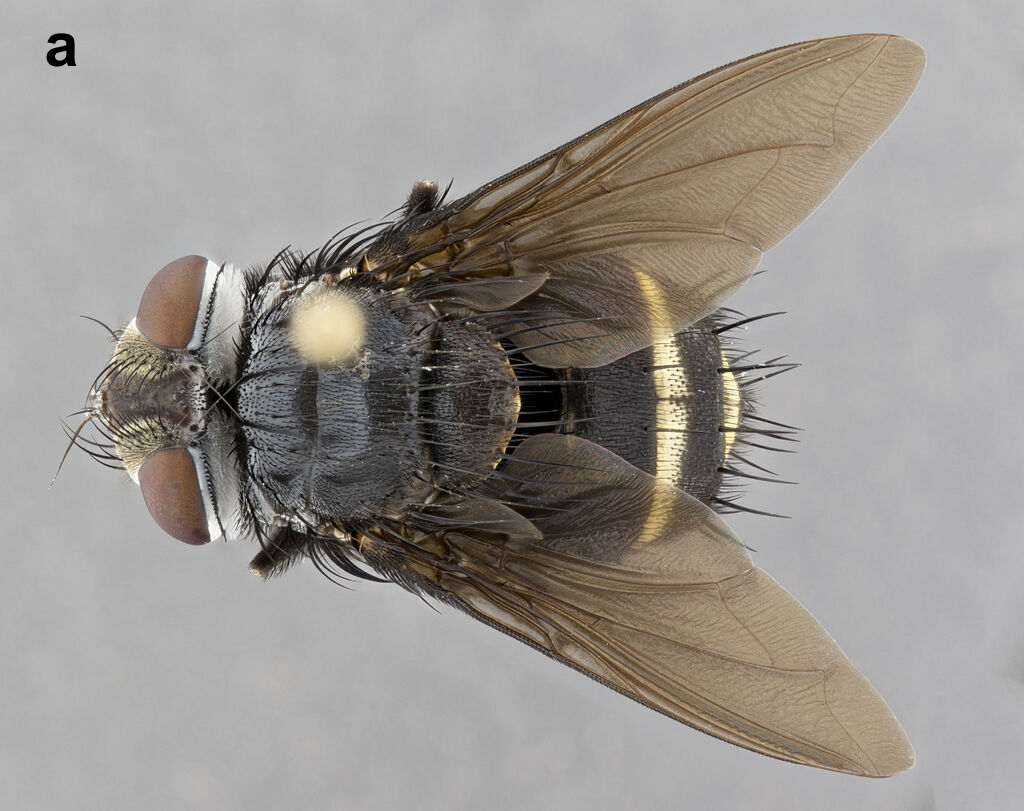
dorsal view

**Figure 28b.**
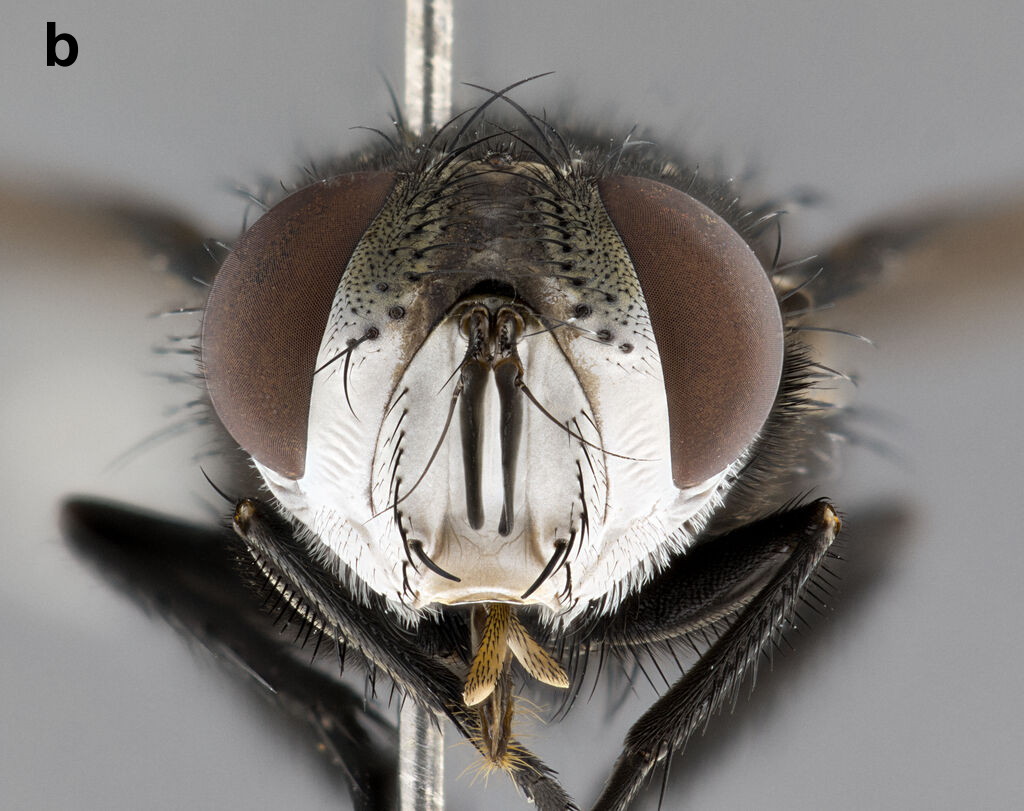
frontal view

**Figure 28c.**
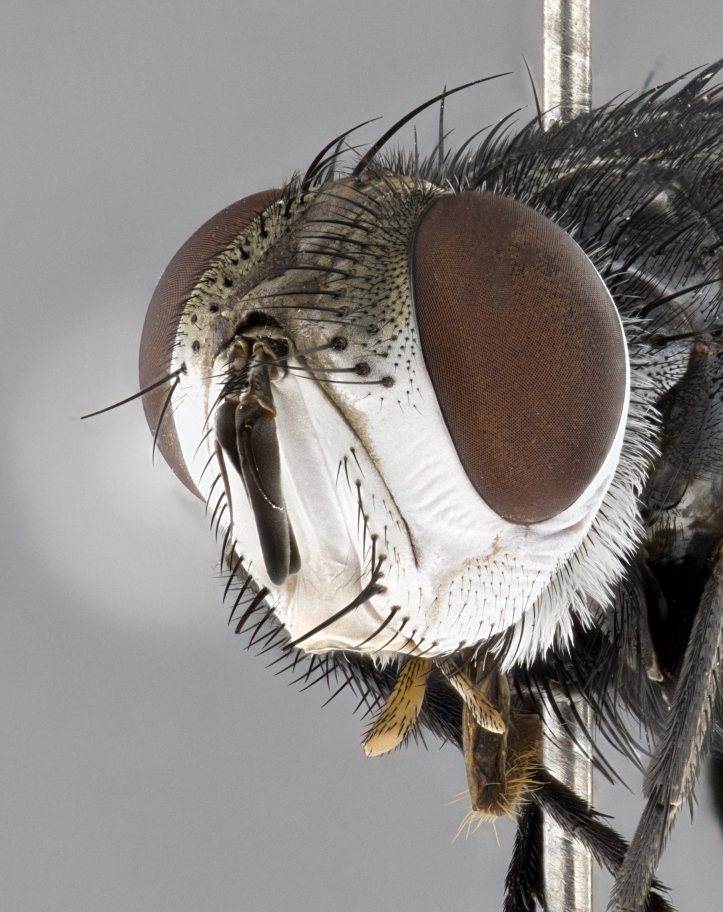
three quarters view

**Figure 28d.**
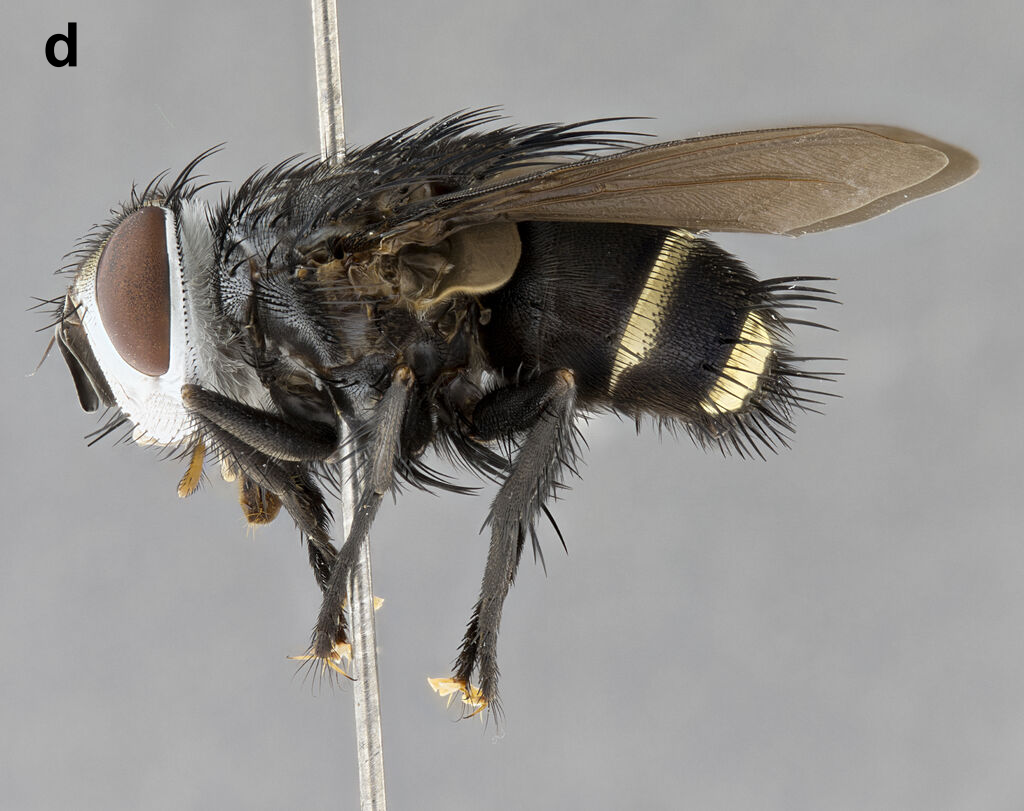
lateral view

**Figure 29a.**
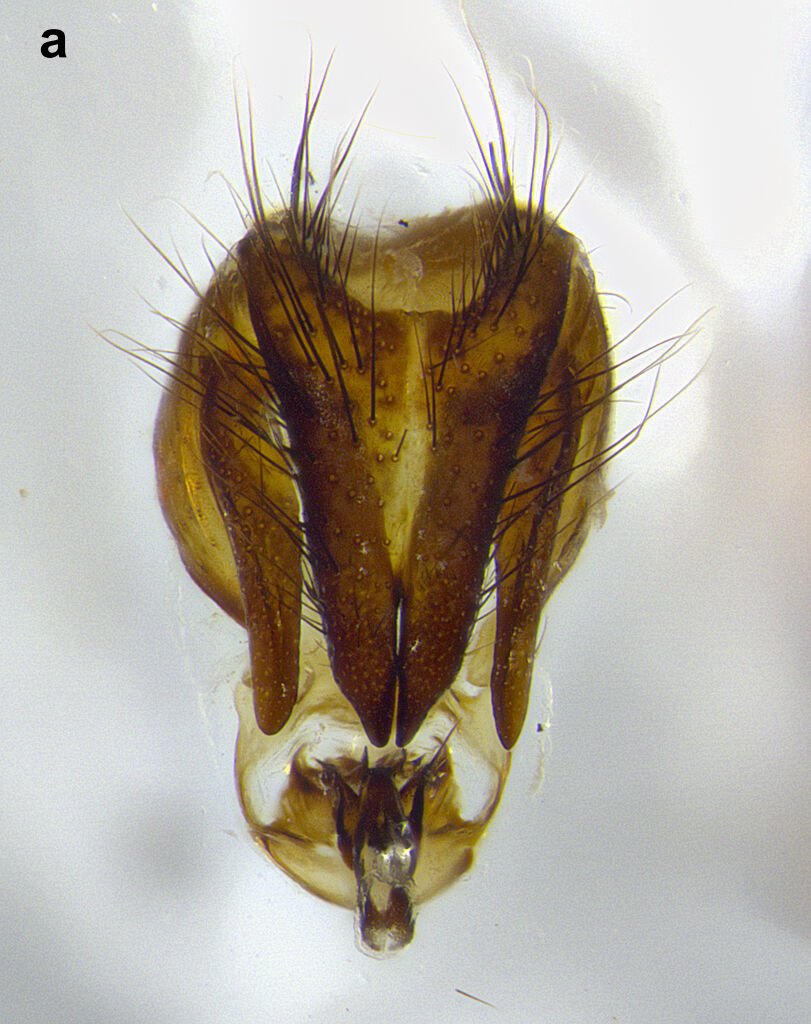
caudal view

**Figure 29b.**
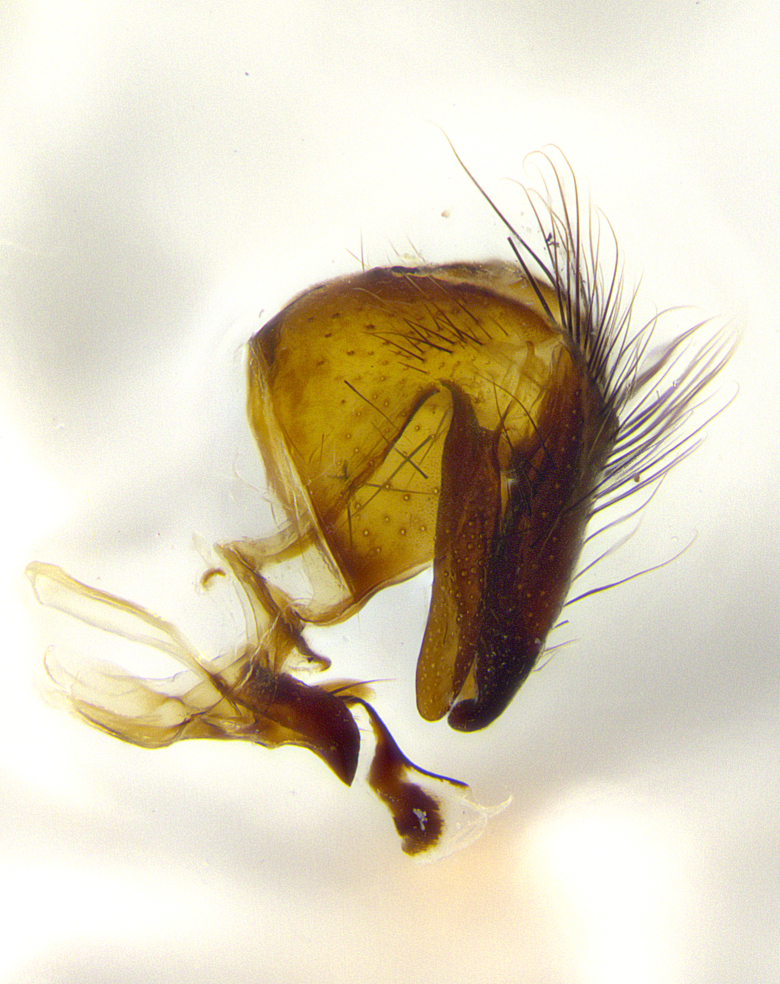
lateral view

**Figure 29c.**
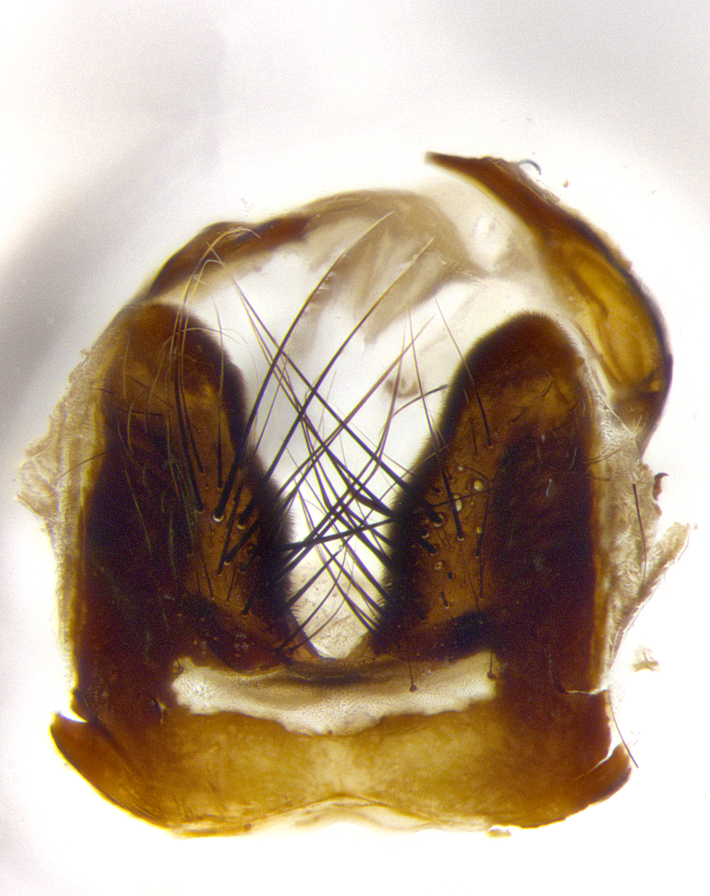
sternite 5, ventral view

**Figure 30a.**
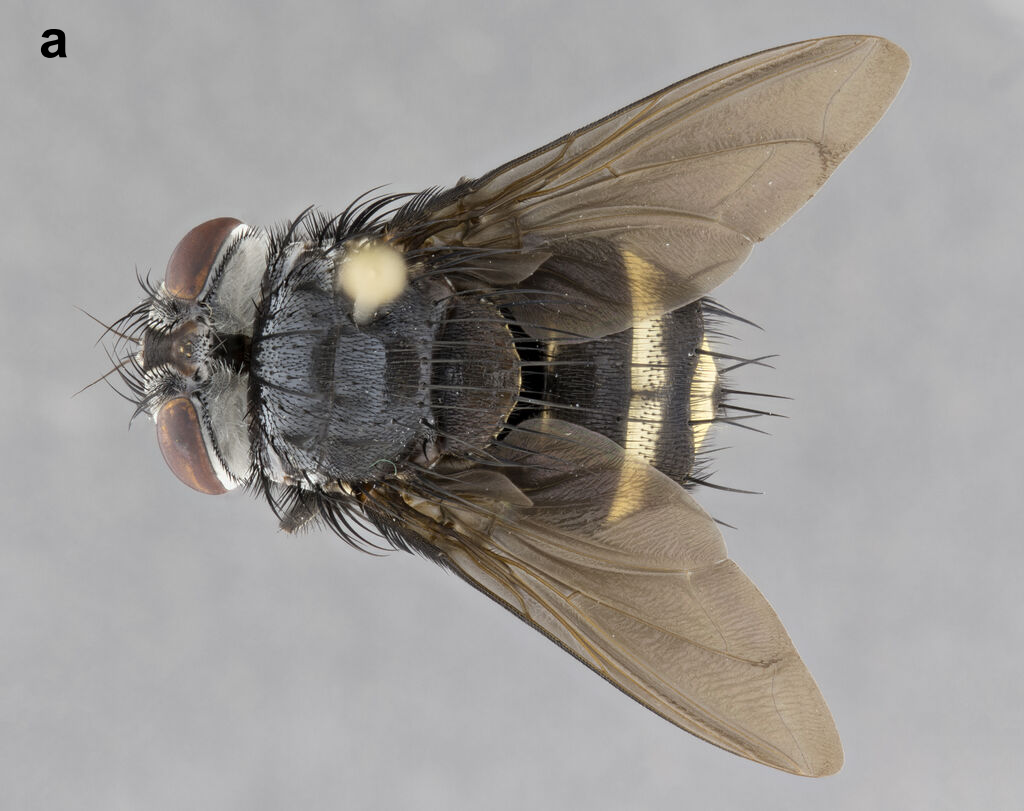
dorsal view

**Figure 30b.**
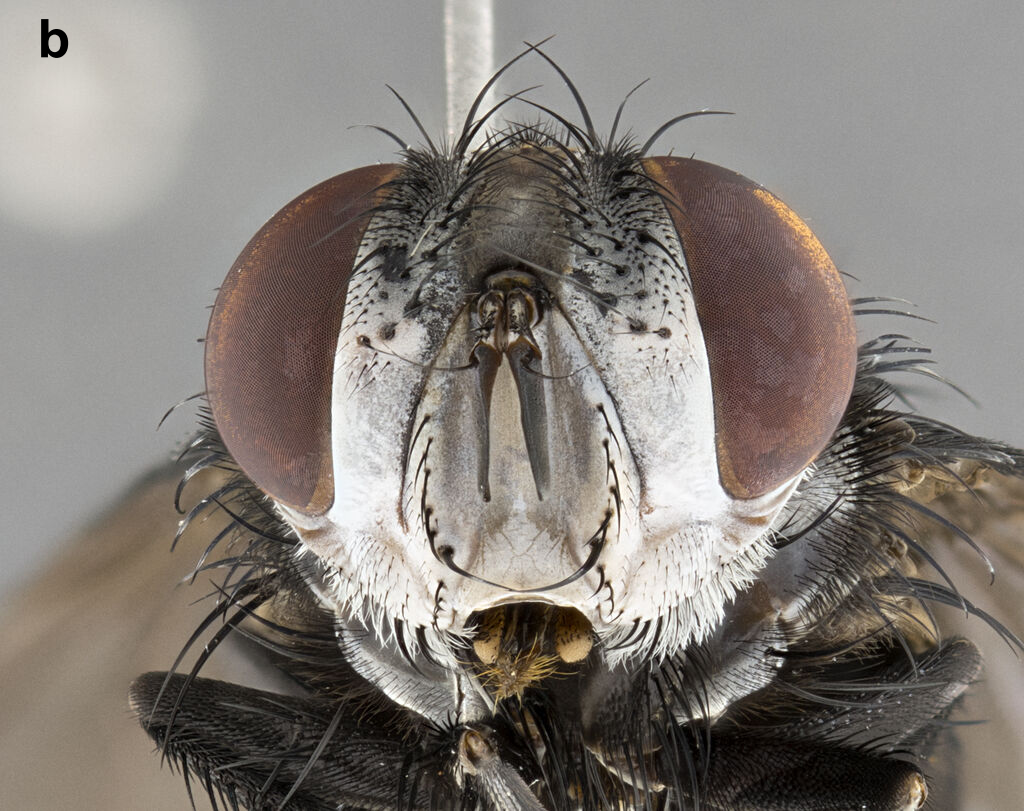
frontal view

**Figure 30c.**
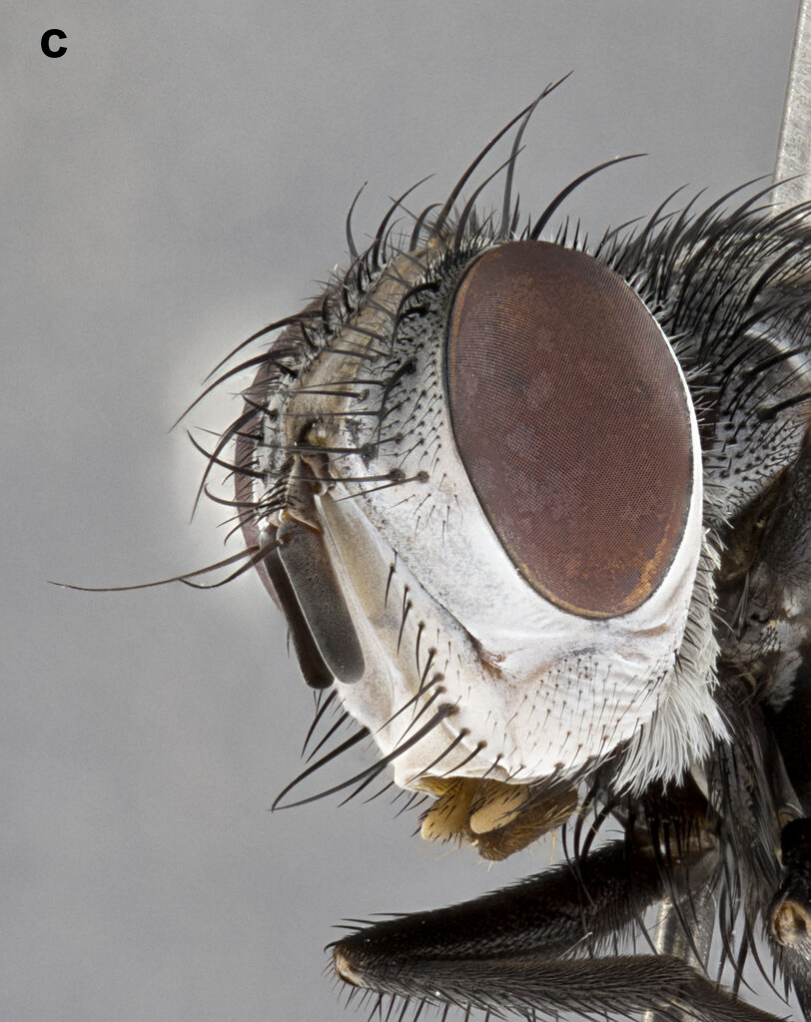
three quarters view

**Figure 30d.**
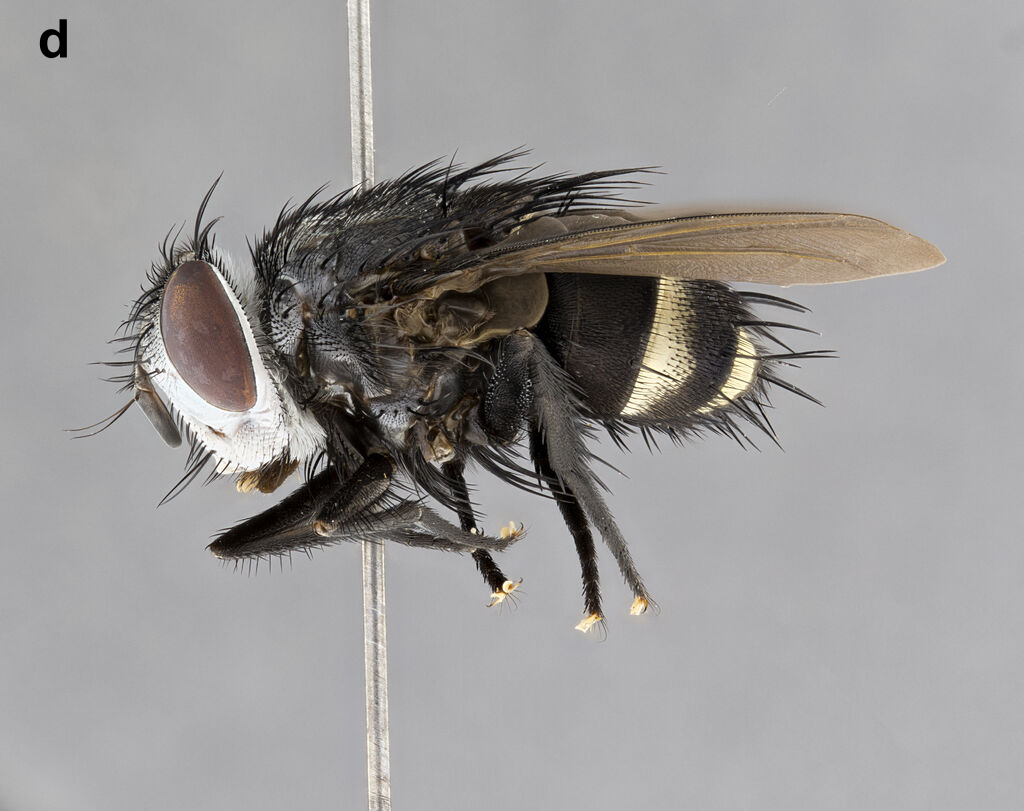
lateral view

**Figure 31a.**
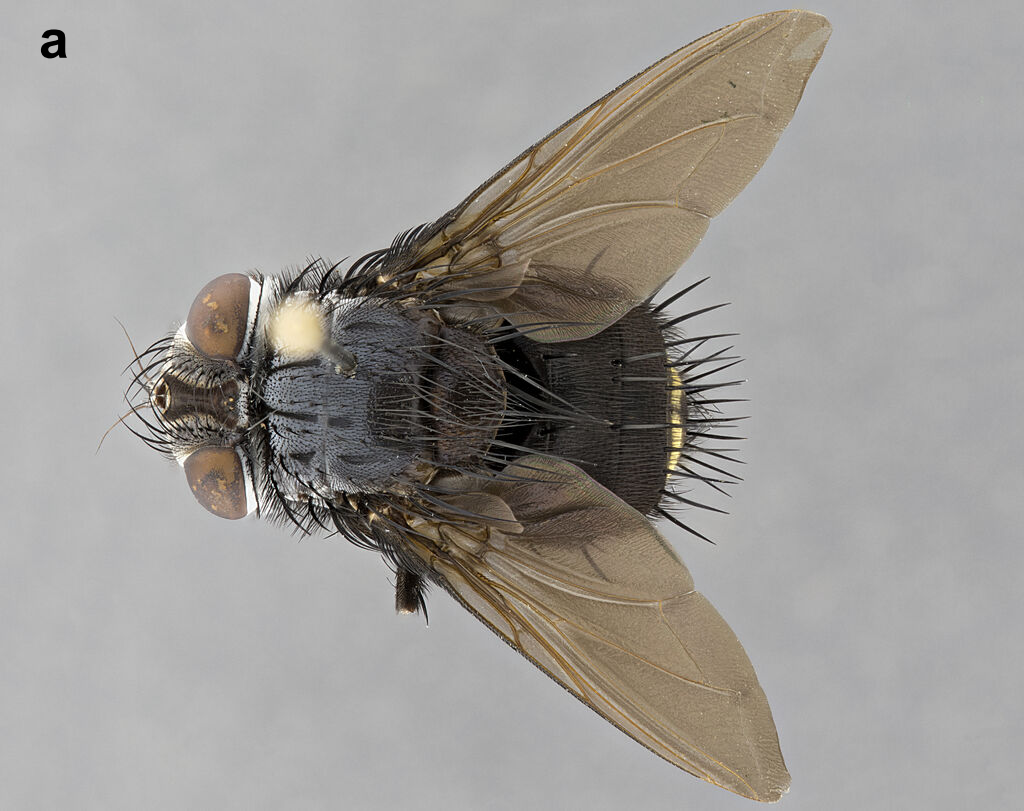
dorsal view

**Figure 31b.**
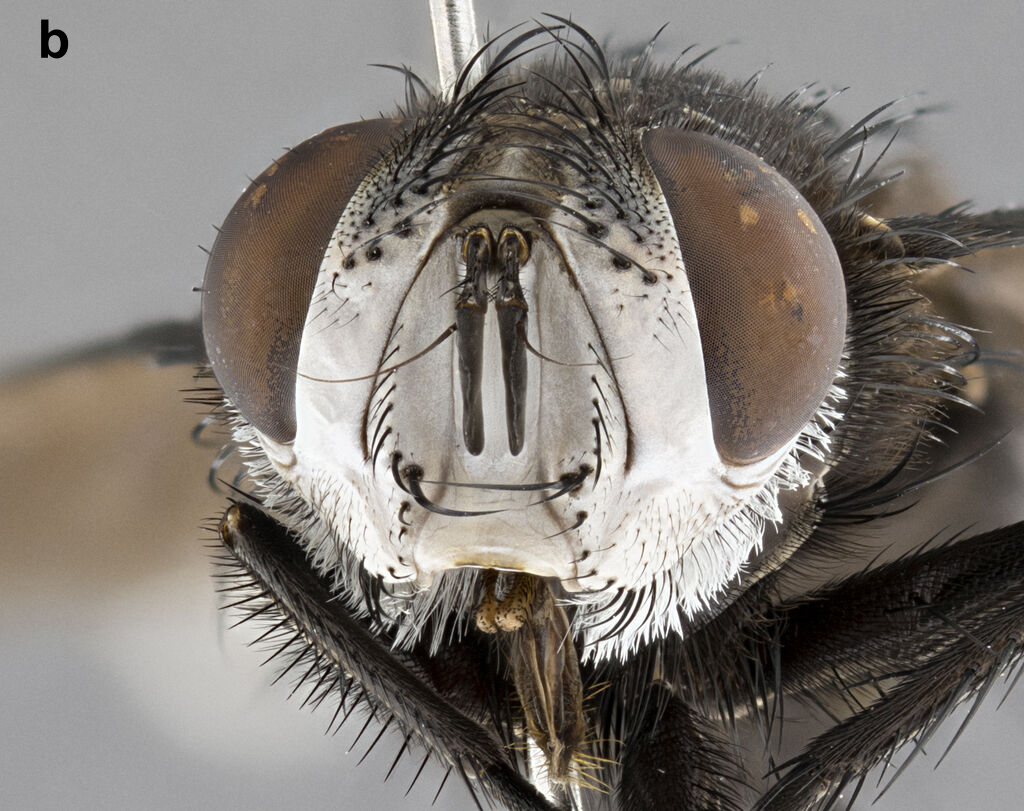
frontal view

**Figure 31c.**
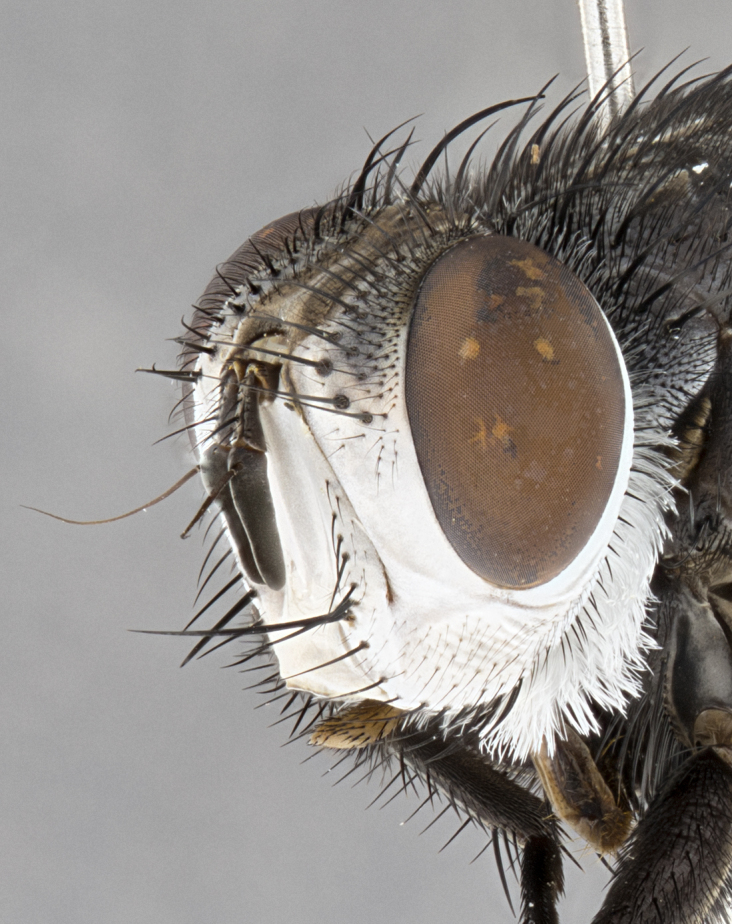
three quarters view

**Figure 31d.**
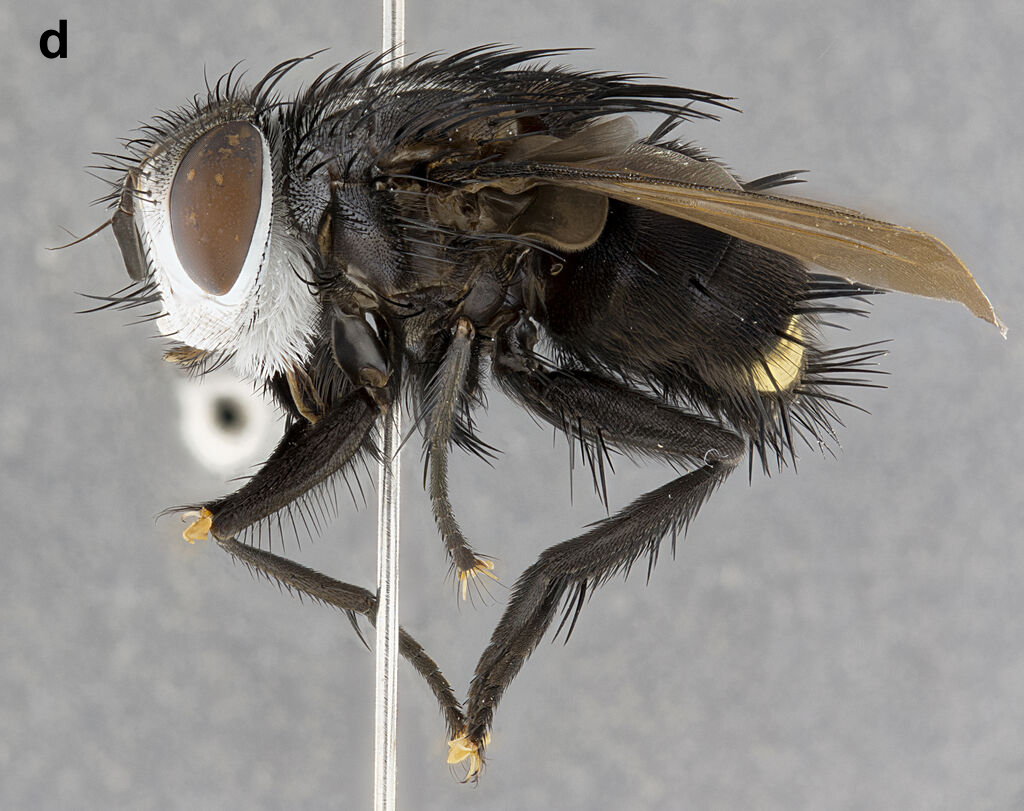
lateral view

**Figure 32a.**
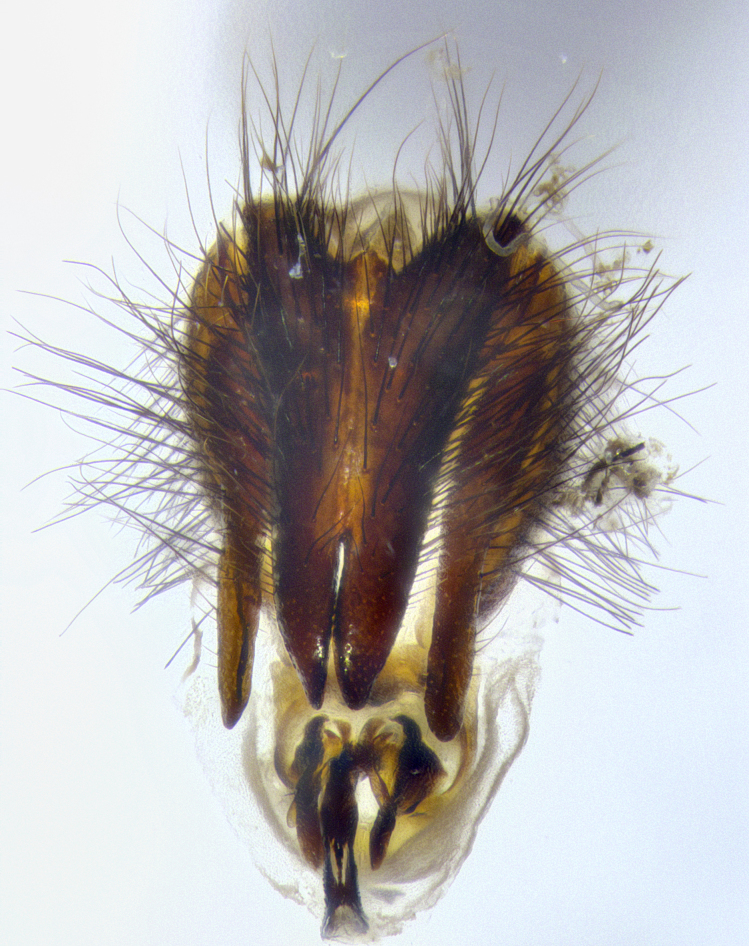
caudal view

**Figure 32b.**
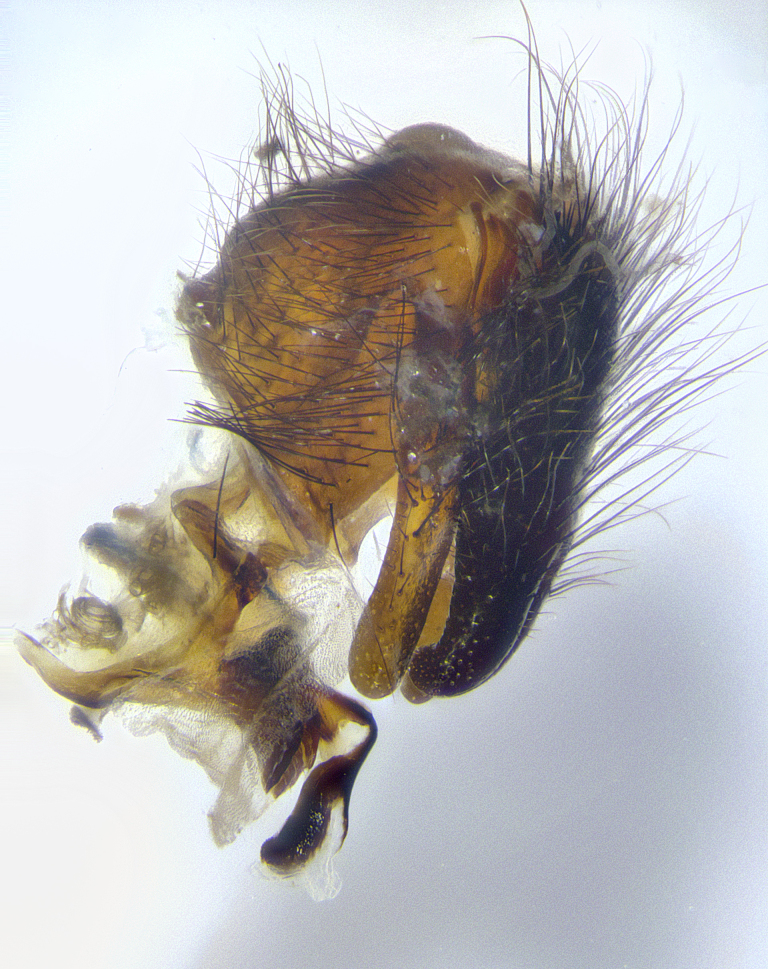
lateral view

**Figure 32c.**
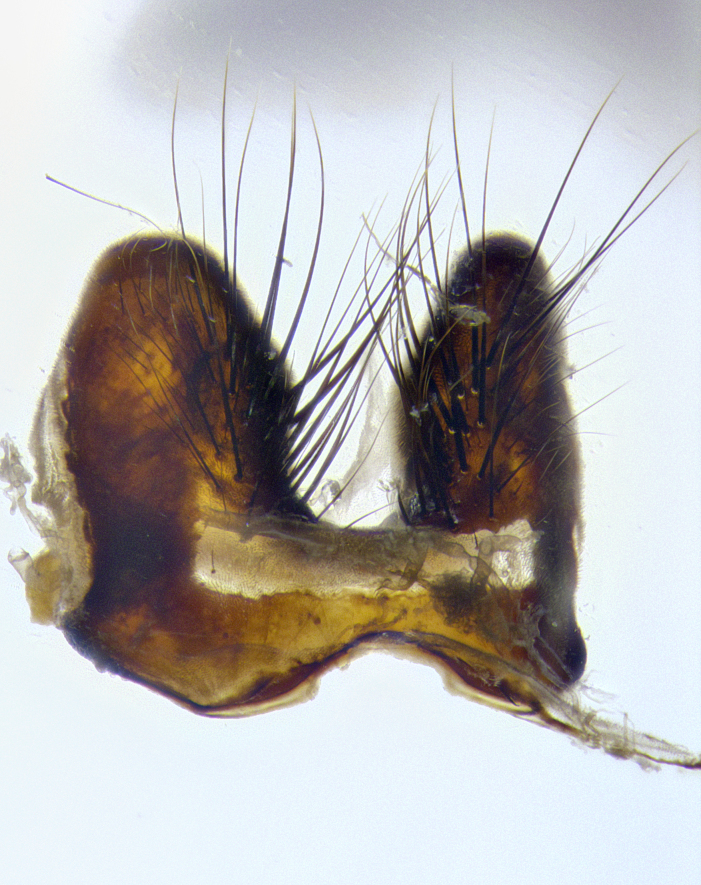
sternite 5, ventral view

**Figure 33a.**
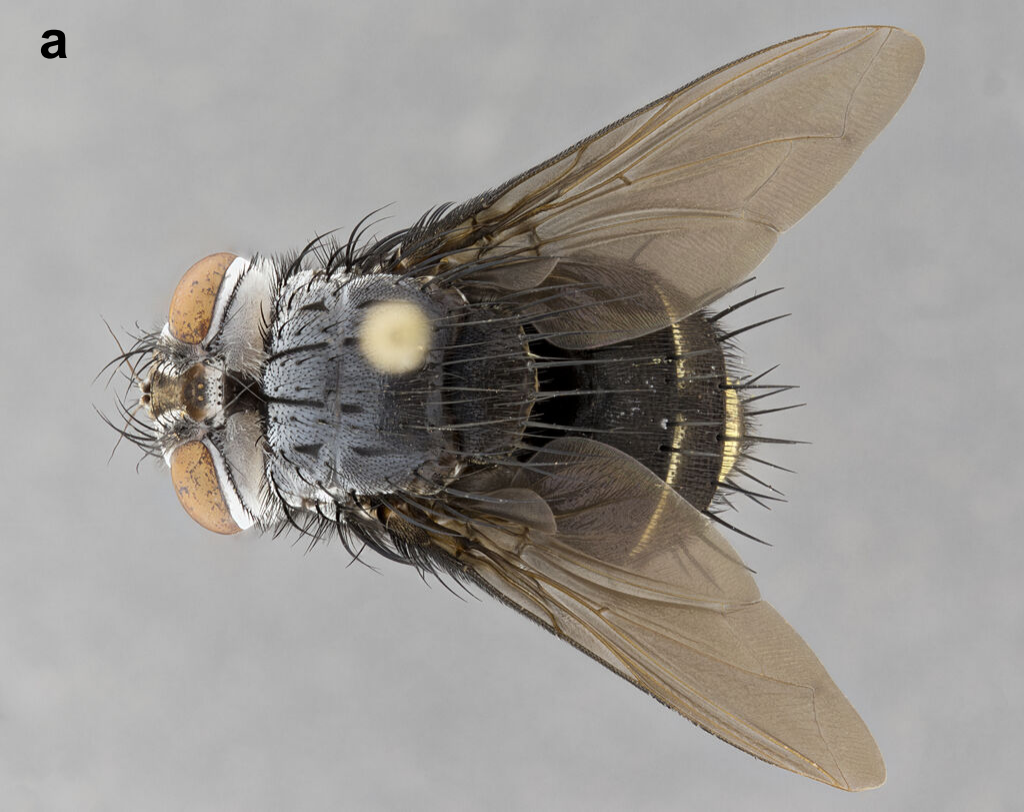
dorsal view

**Figure 33b.**
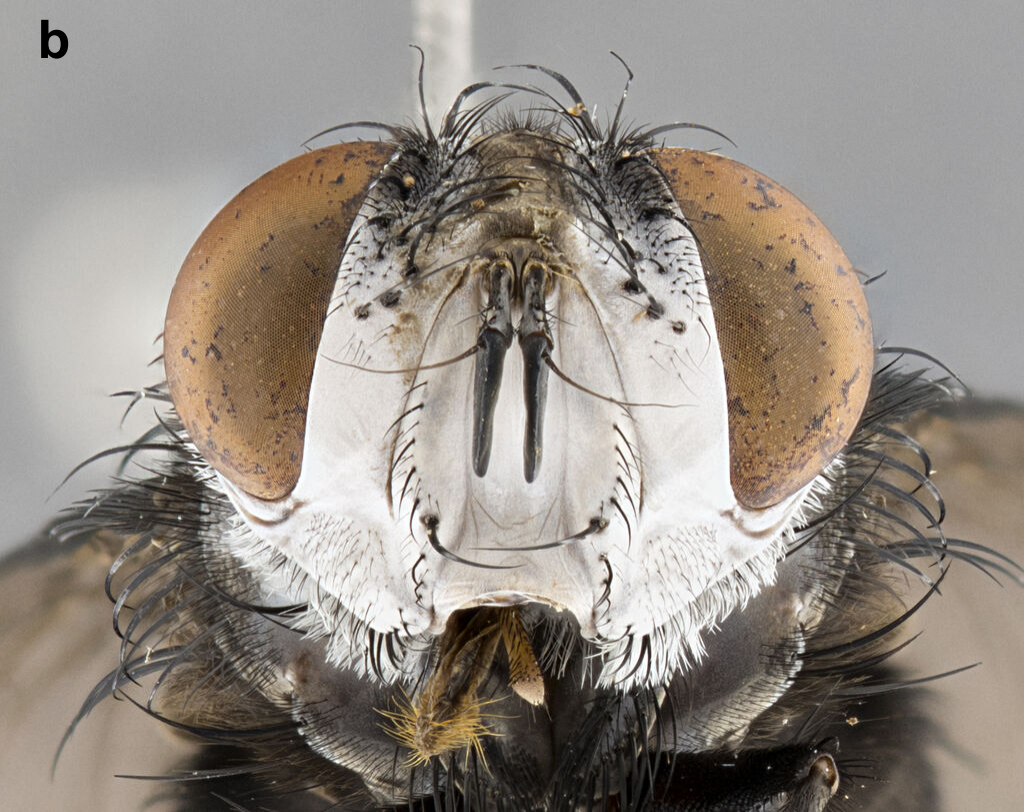
frontal view

**Figure 33c.**
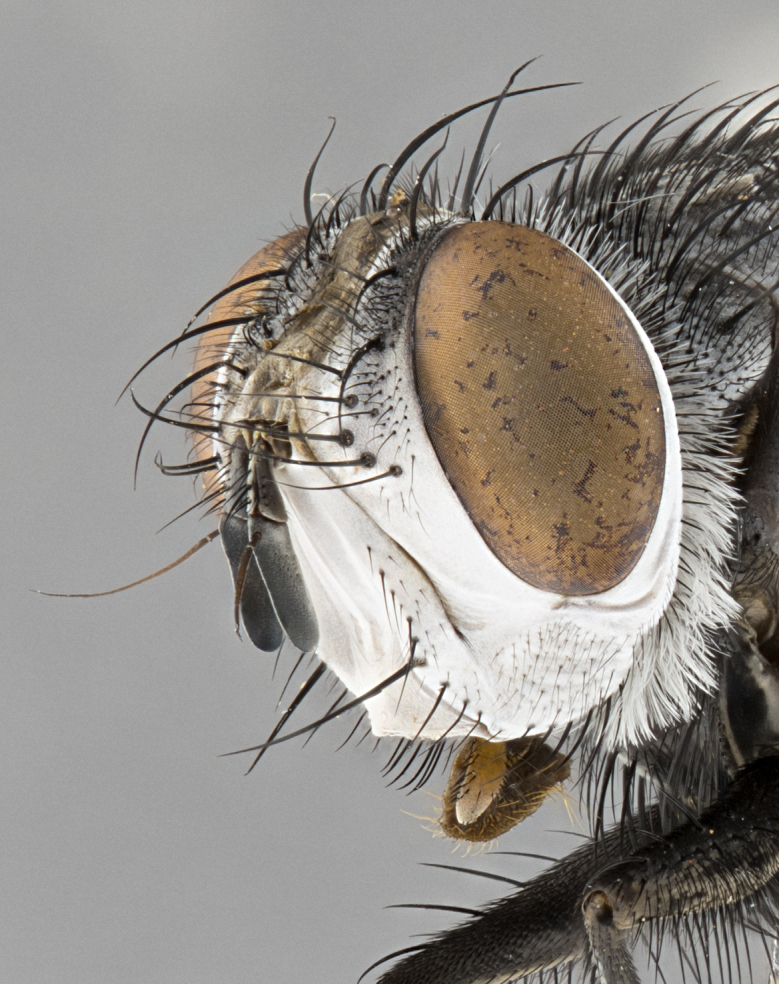
three quarters view

**Figure 33d.**
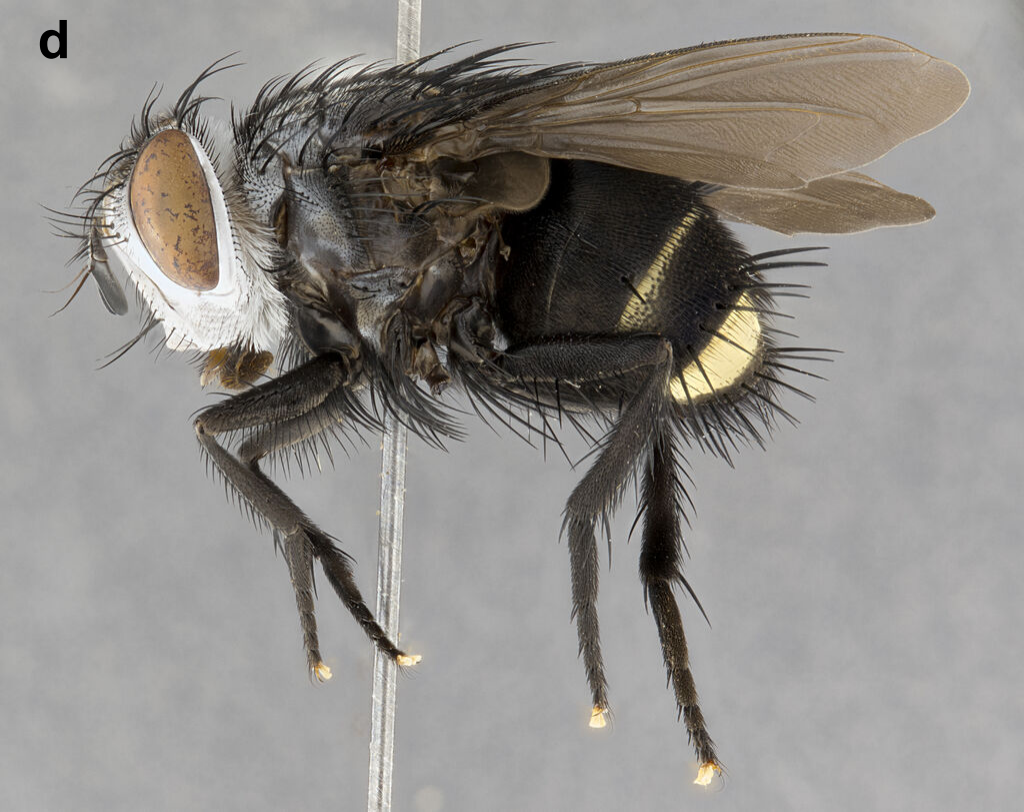
lateral view

**Figure 34a.**
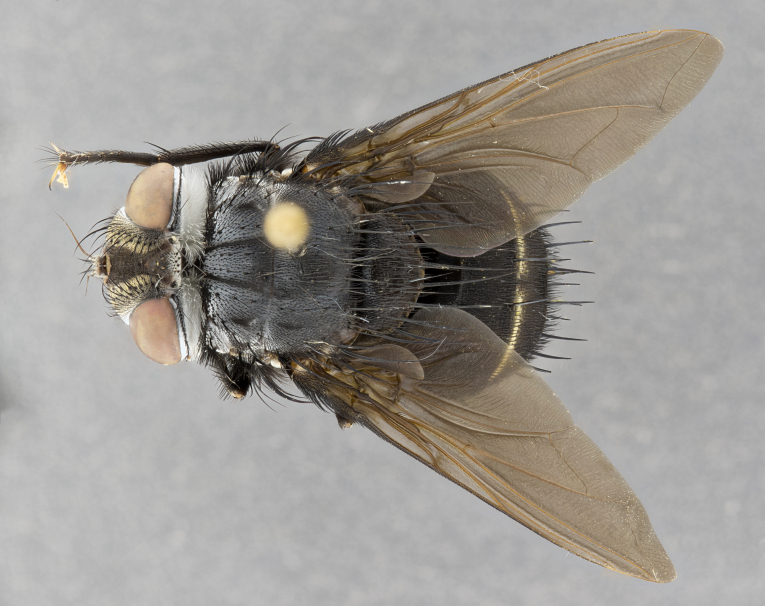
dorsal view

**Figure 34b.**
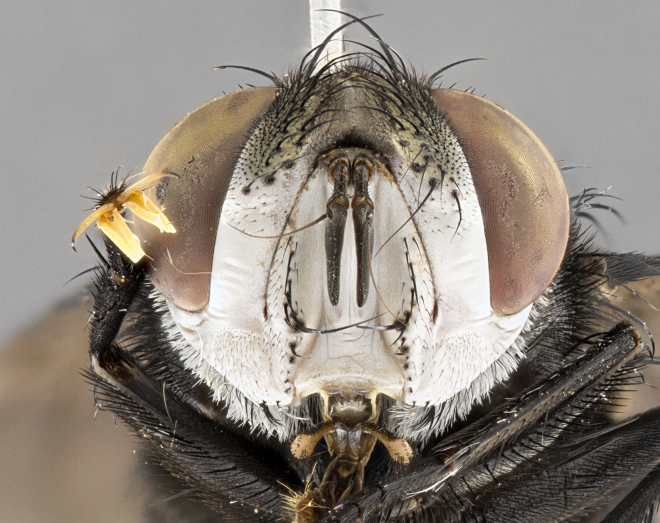
frontal view

**Figure 34c.**
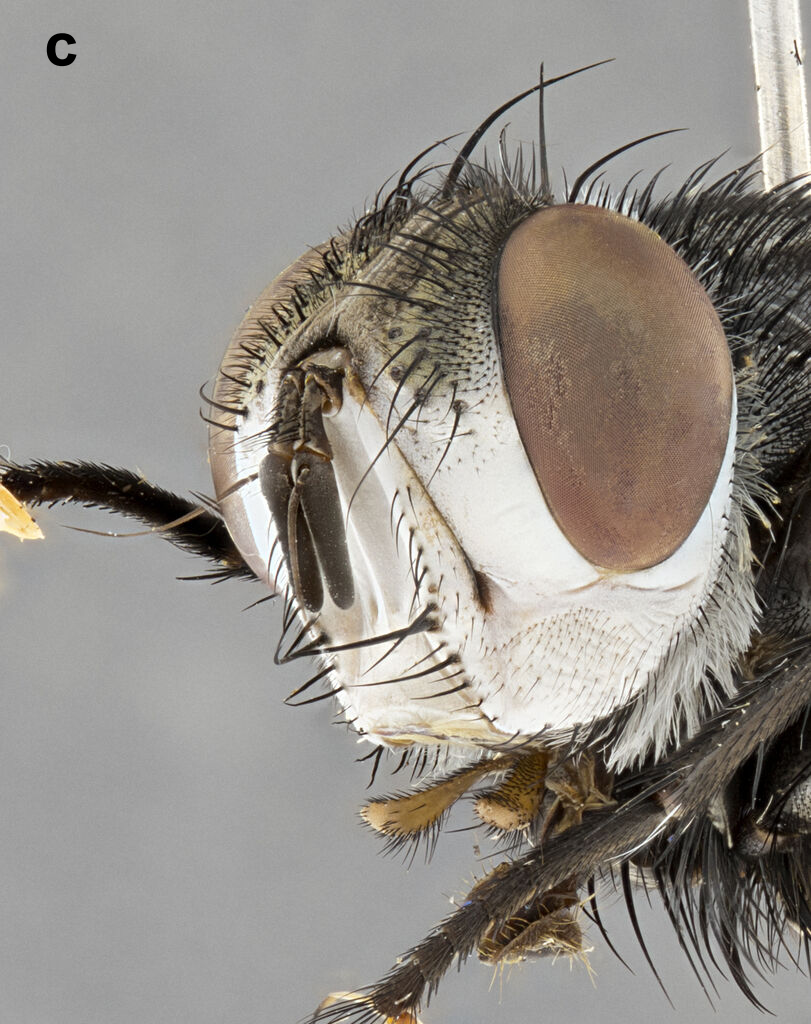
three quarters view

**Figure 34d.**
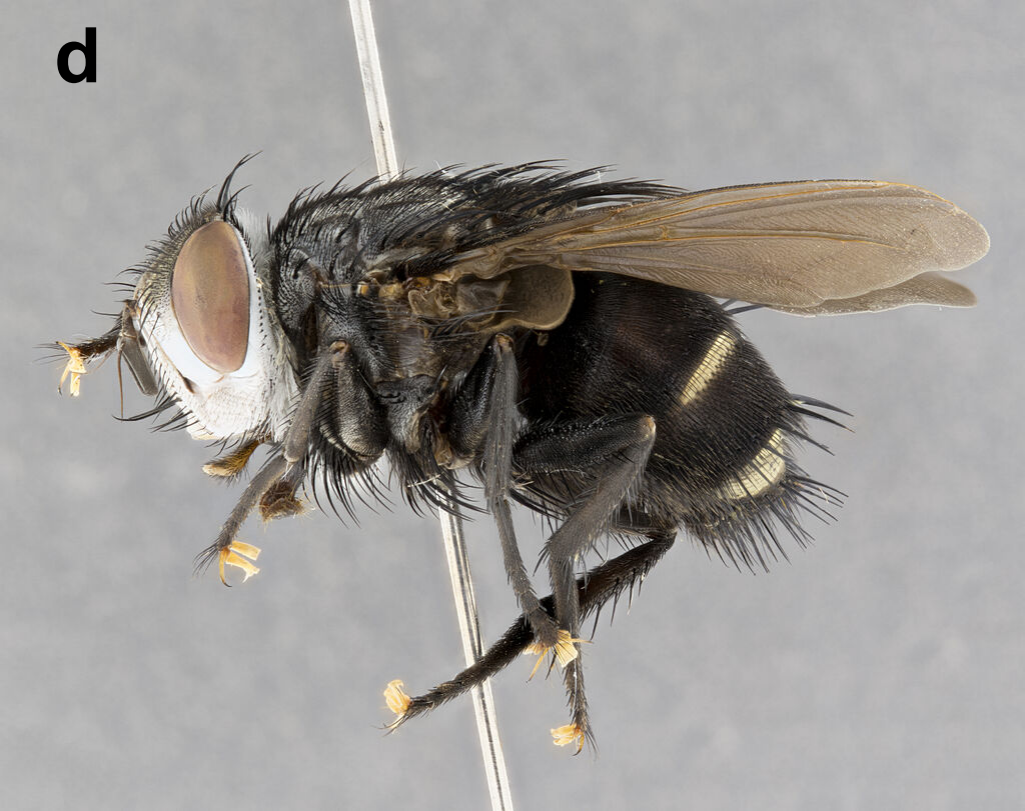
lateral view

**Figure 35a.**
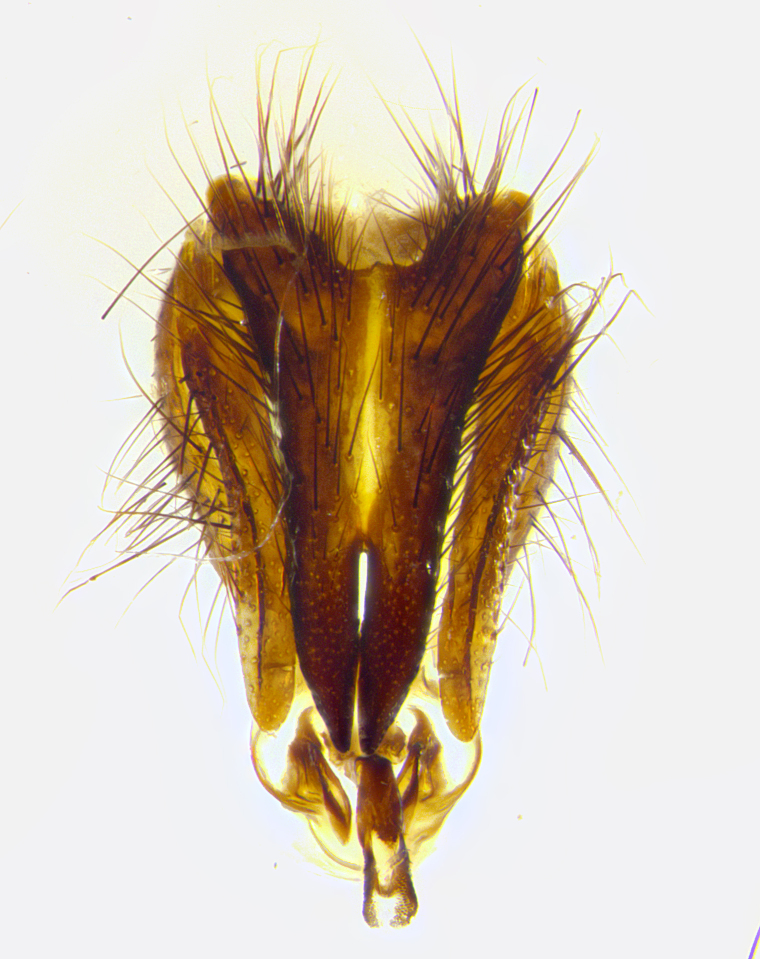
caudal view

**Figure 35b.**
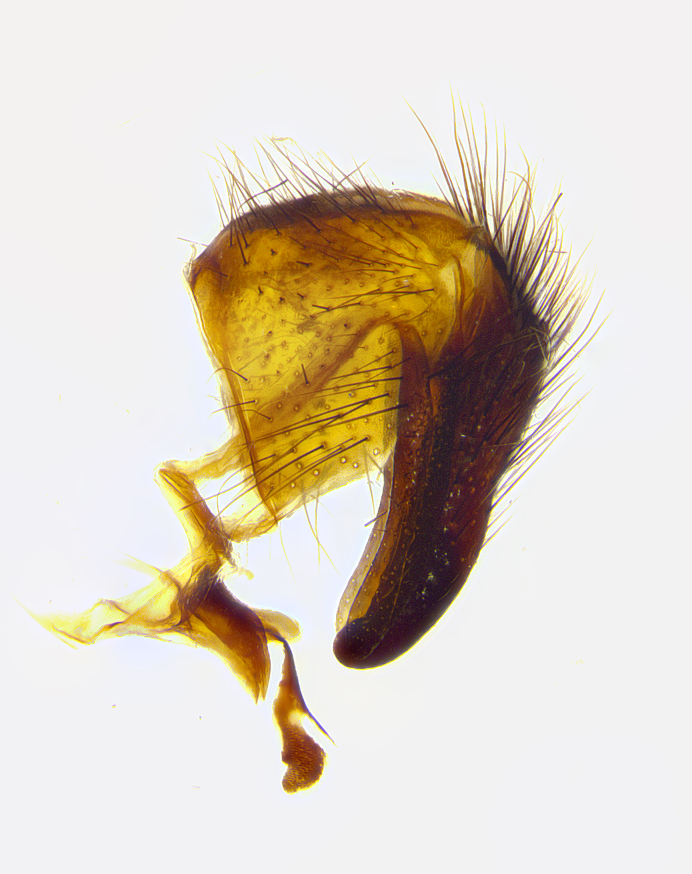
lateral view

**Figure 35c.**
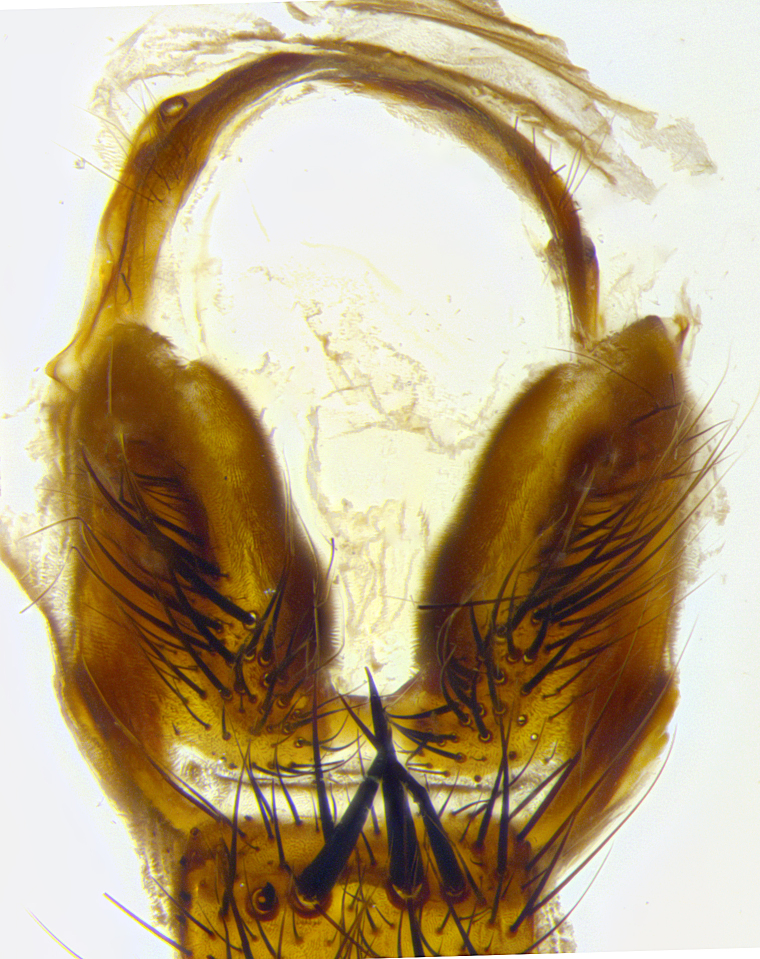
sternite 5, ventral view

**Figure 36a.**
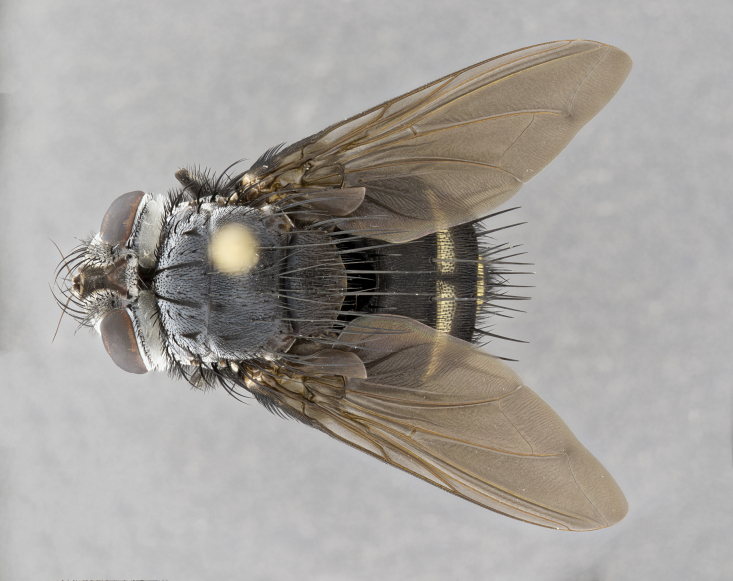
dorsal view

**Figure 36b.**
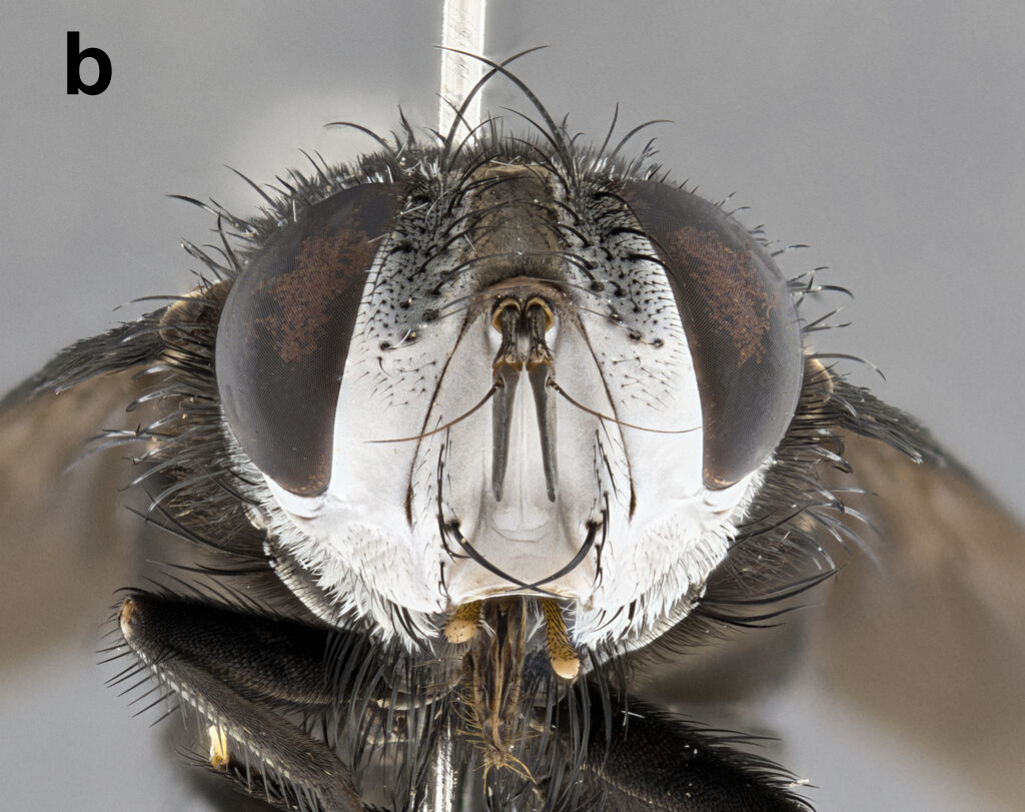
frontal view

**Figure 36c.**
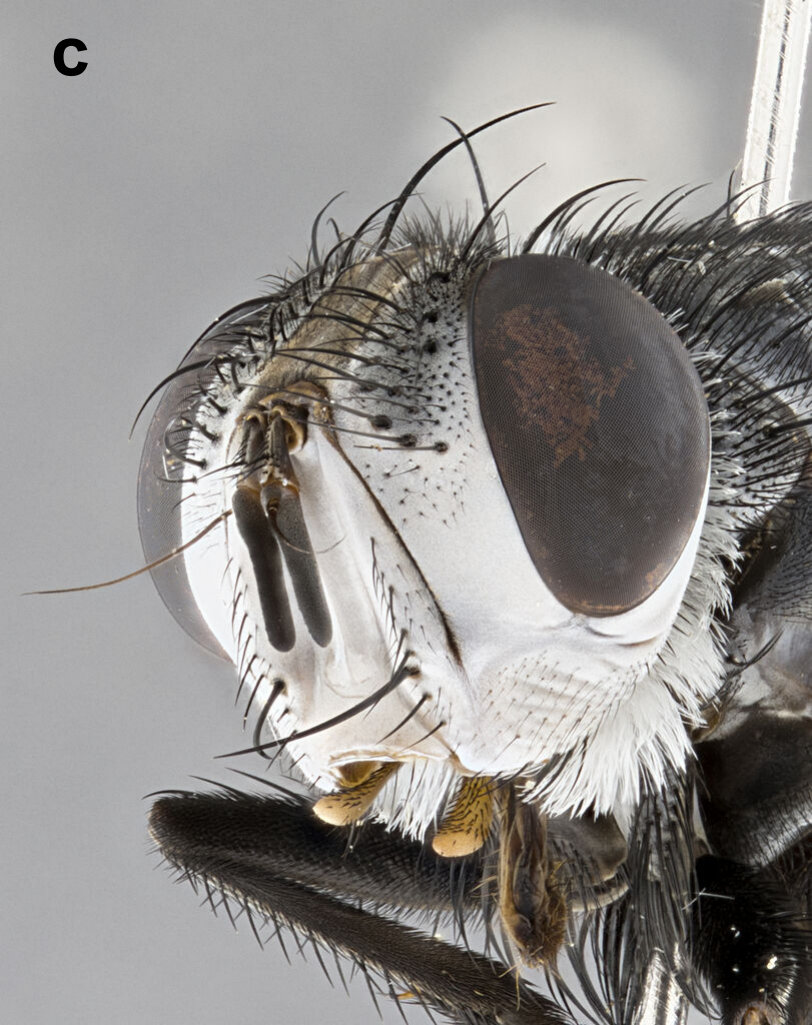
three quarters view

**Figure 36d.**
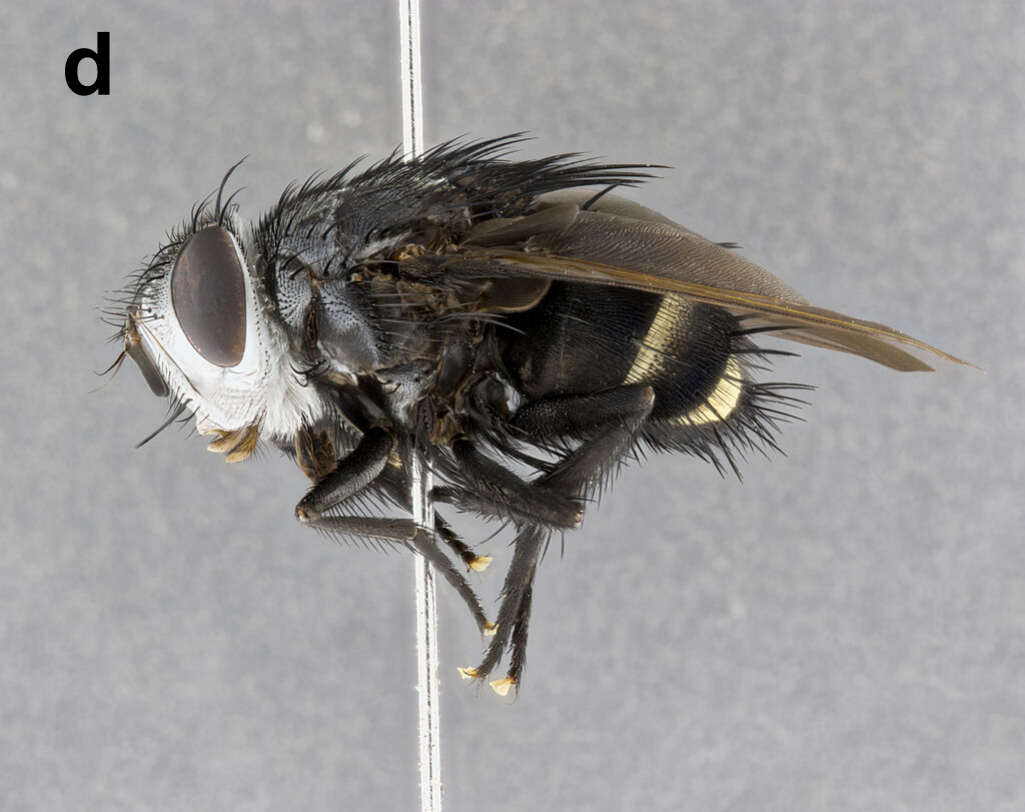
lateral view

**Figure 37a.**
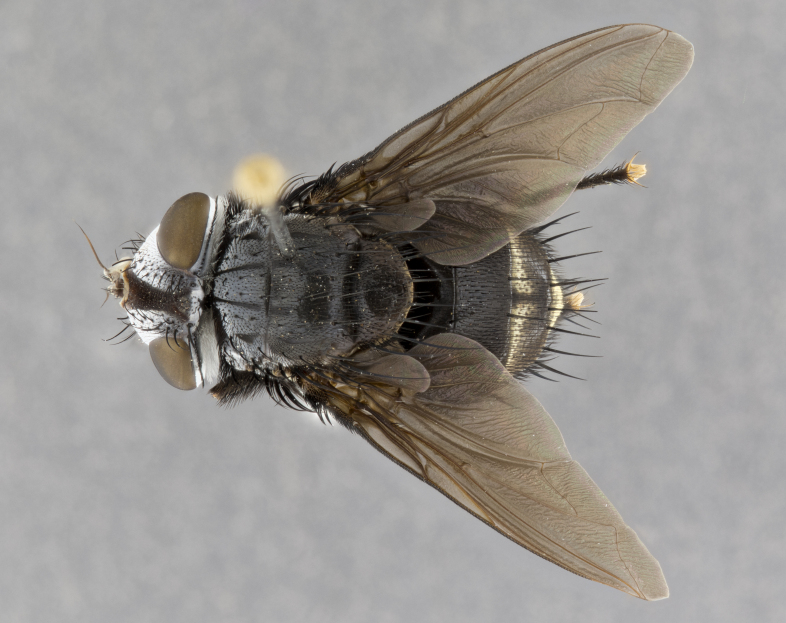
dorsal view

**Figure 37b.**
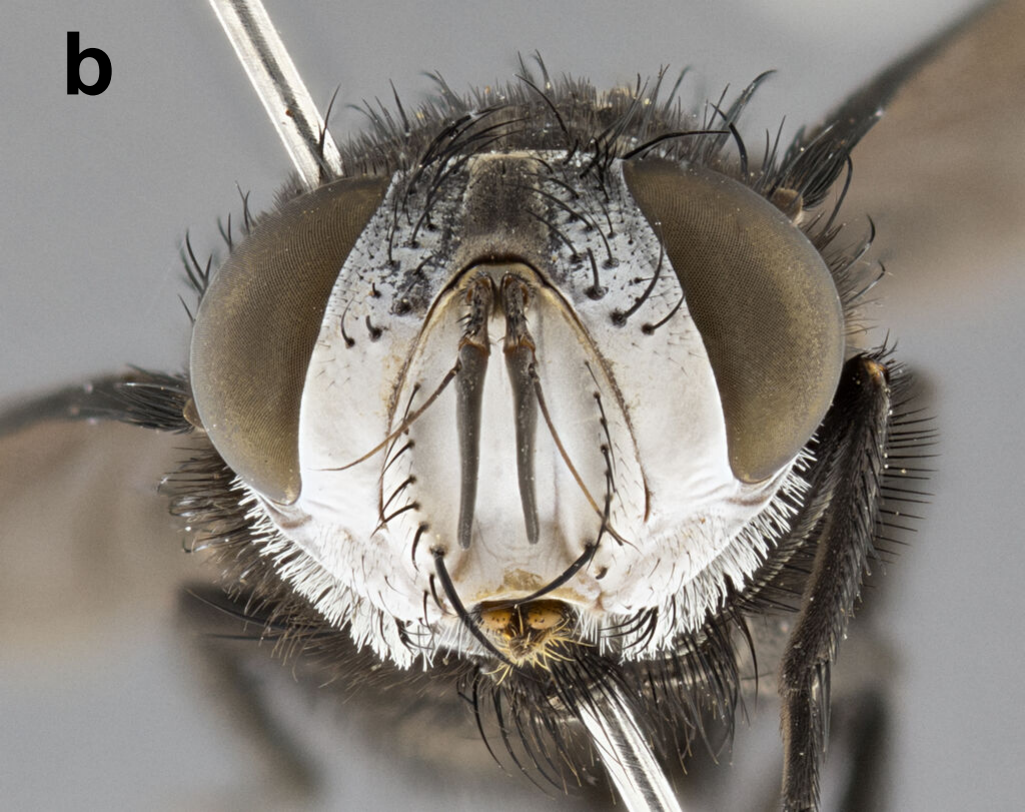
frontal view

**Figure 37c.**
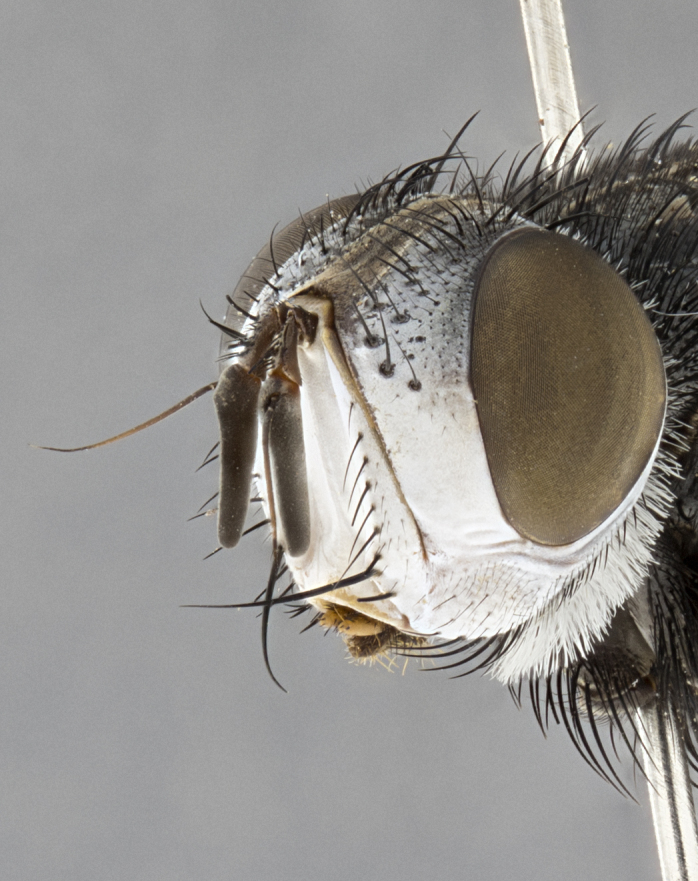
three quarters view

**Figure 37d.**
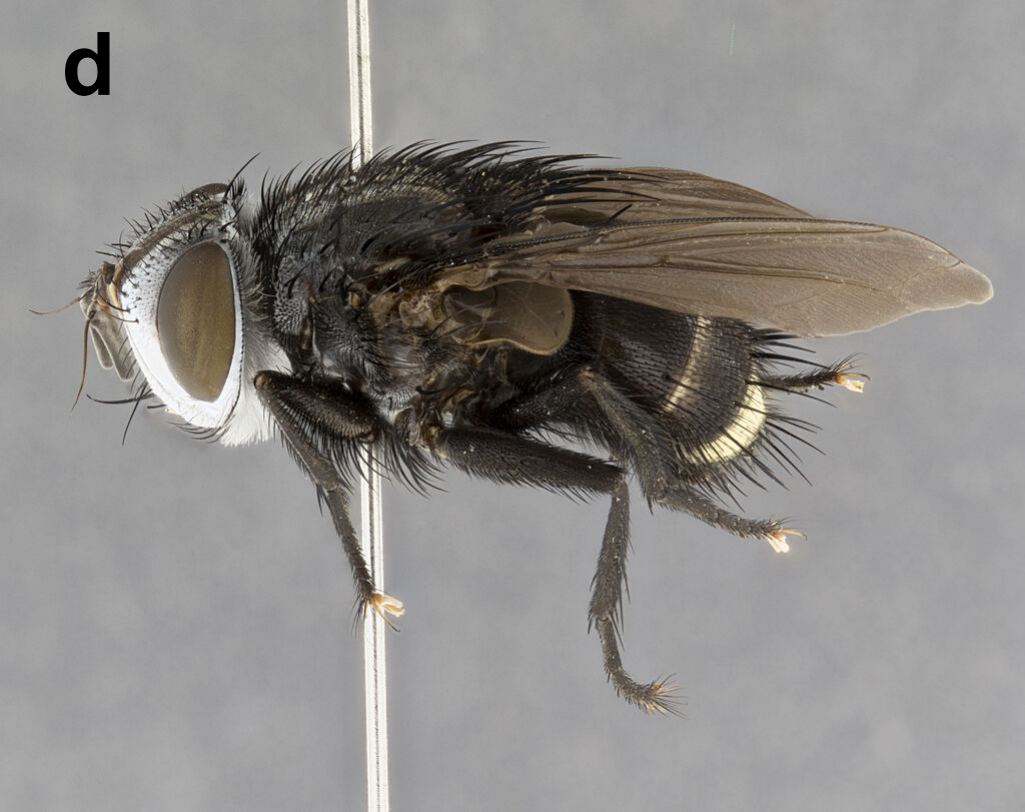
lateral view

**Figure 38a.**
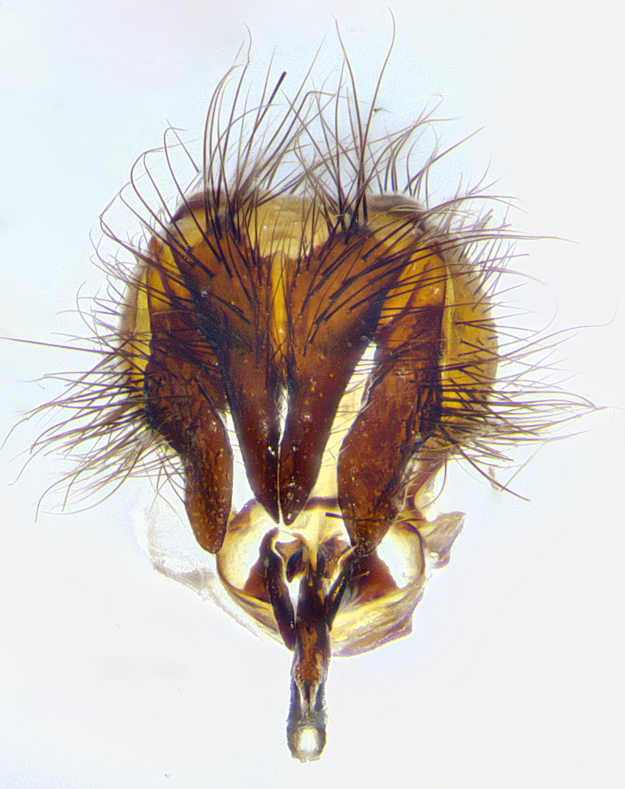
caudal view

**Figure 38b.**
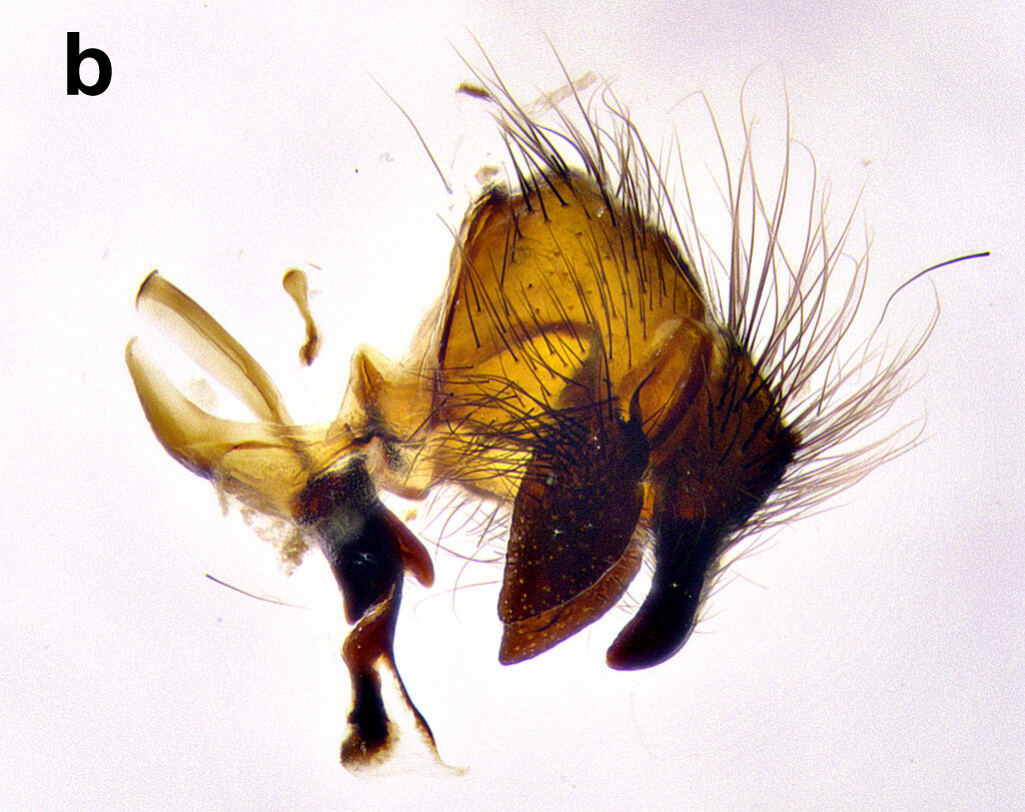
lateral view

**Figure 38c.**
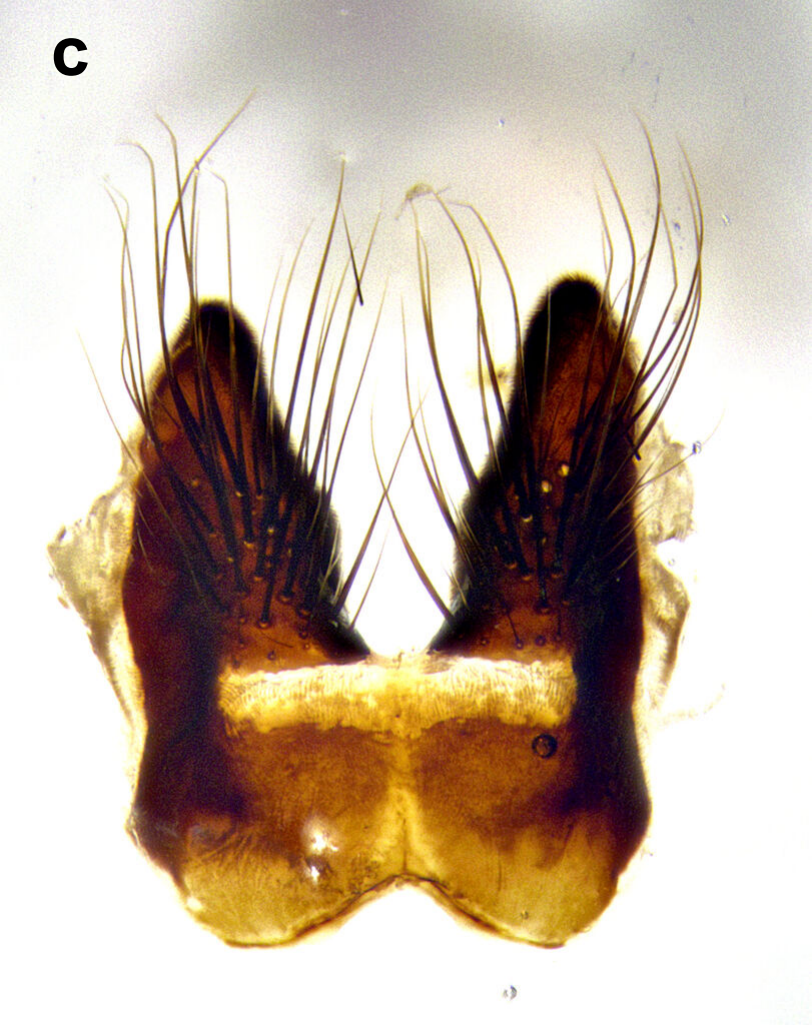
sternite 5, ventral view

**Figure 39a.**
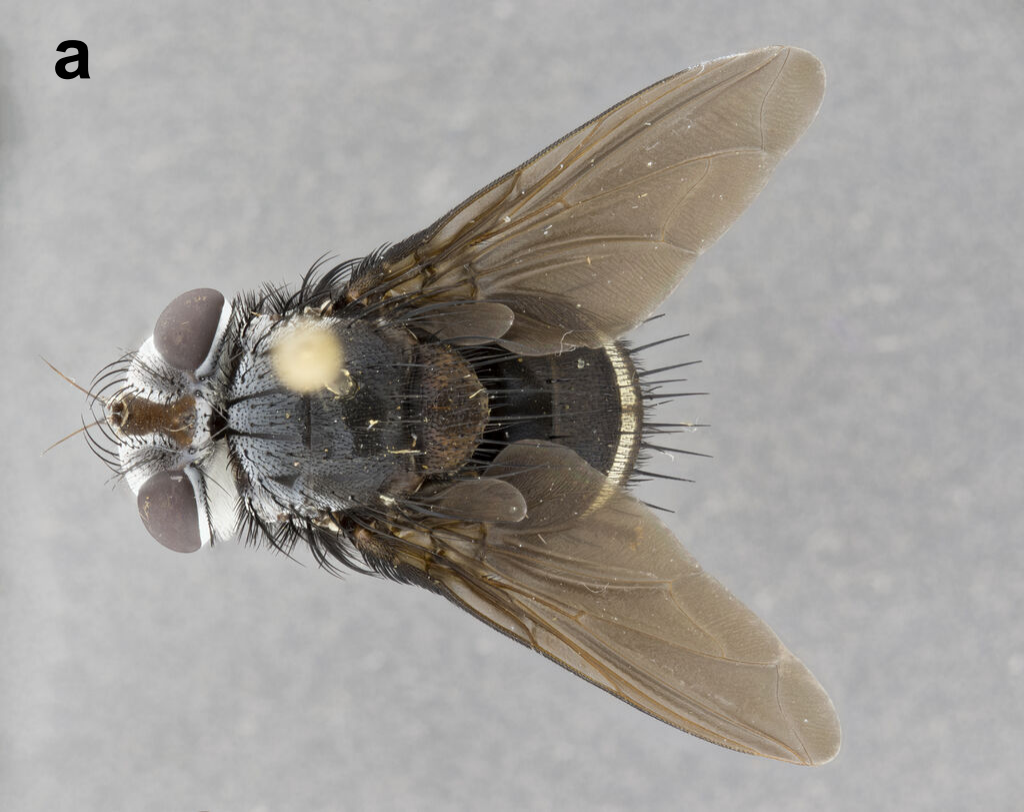
dorsal view

**Figure 39b.**
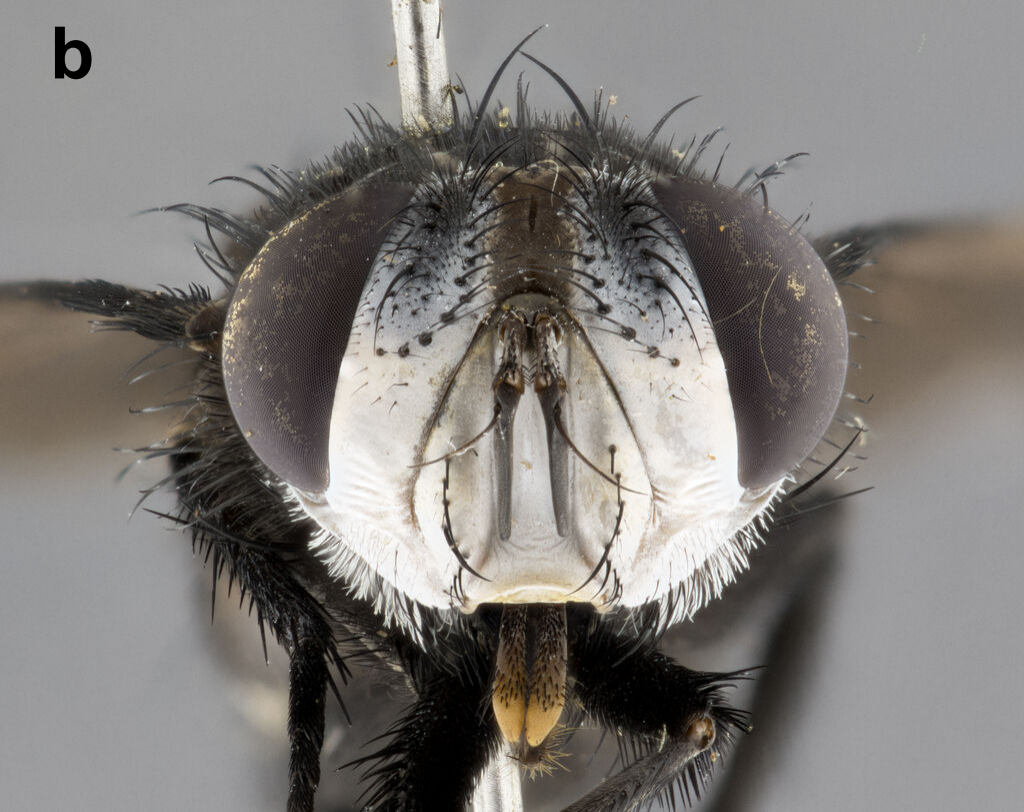
frontal view

**Figure 39c.**
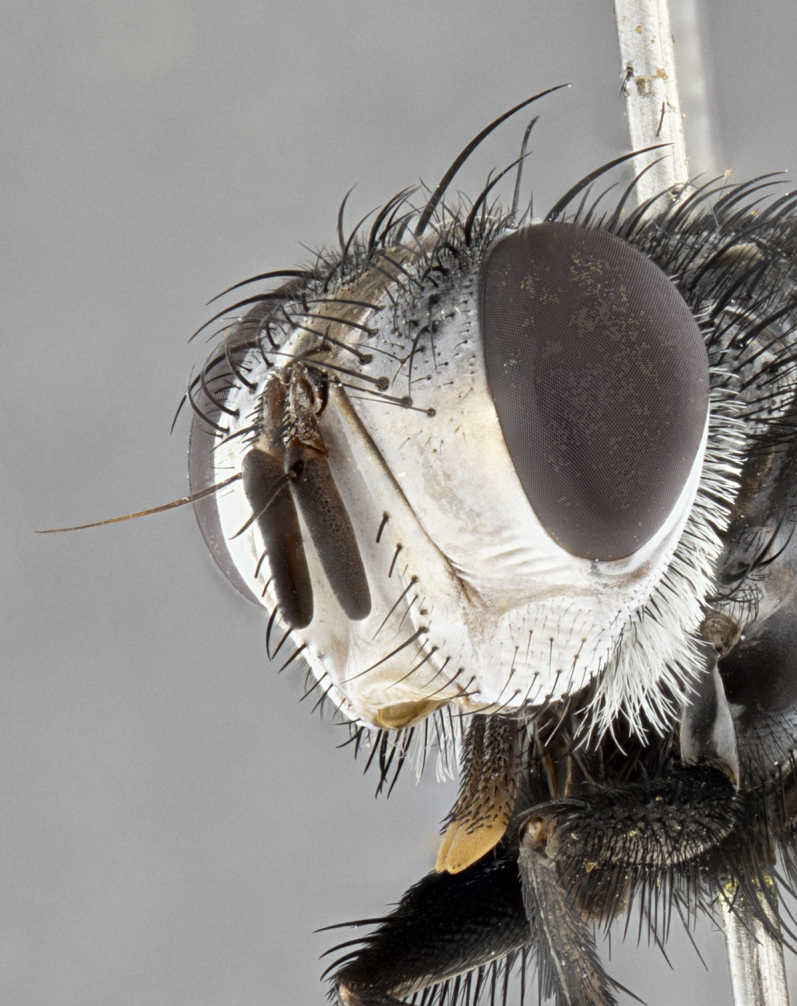
three quarters view

**Figure 39d.**
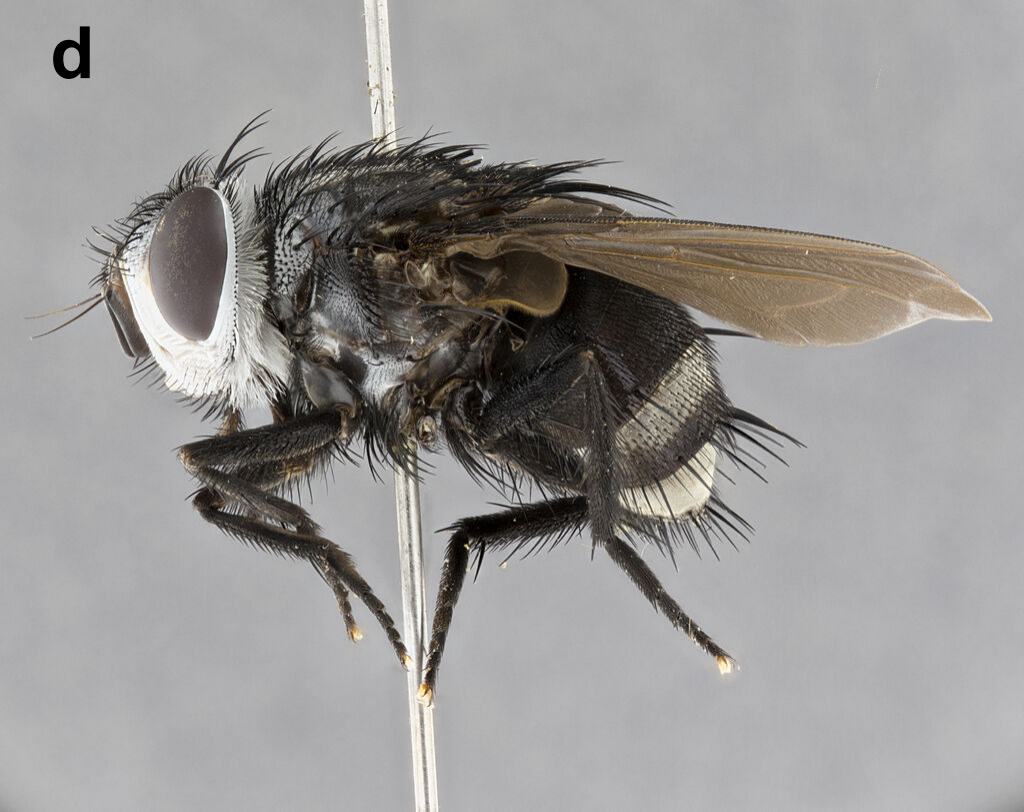
lateral view

**Figure 40a.**
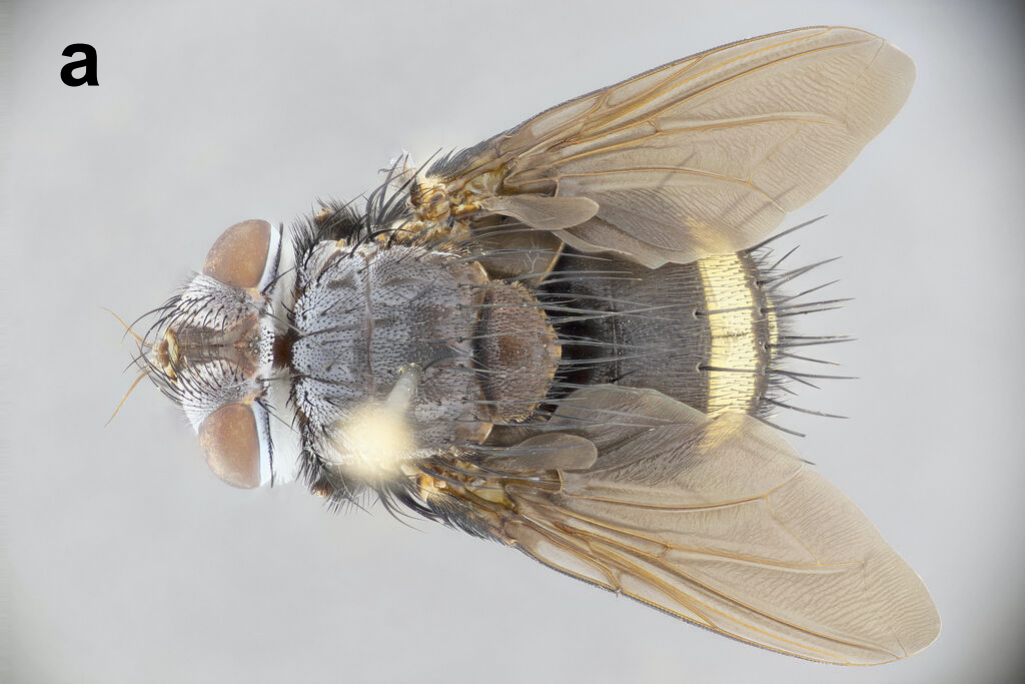
dorsal view

**Figure 40b.**
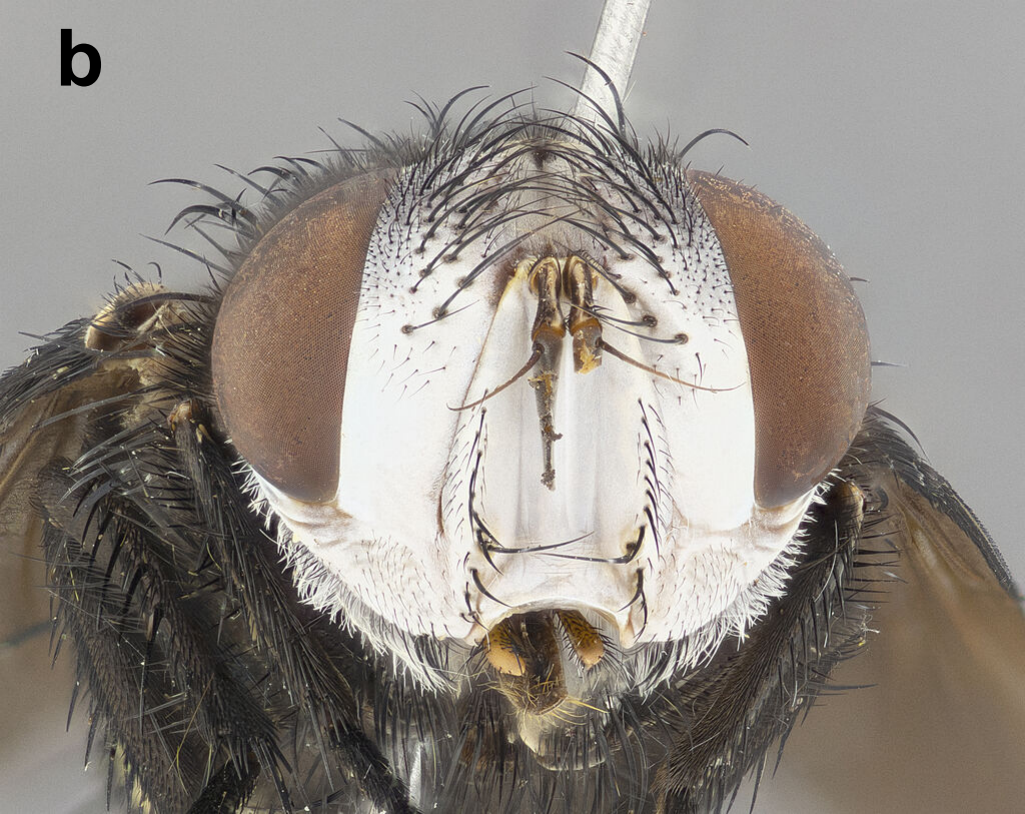
head view

**Figure 40c.**
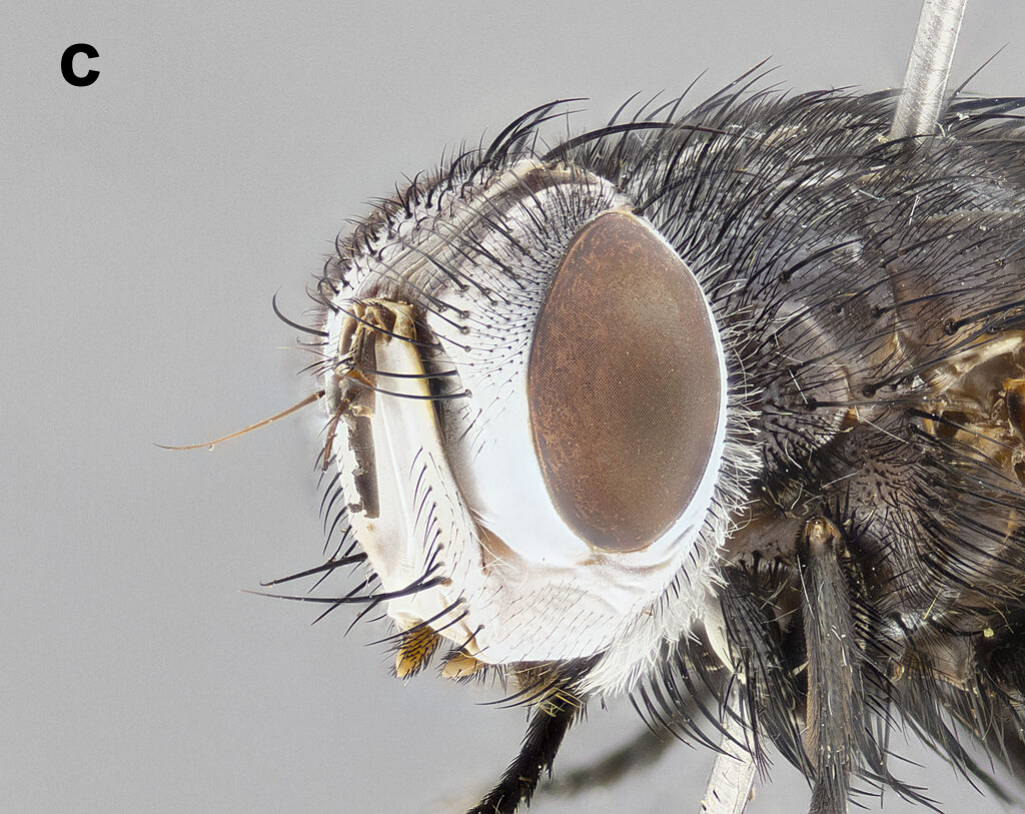
three quarters view

**Figure 40d.**
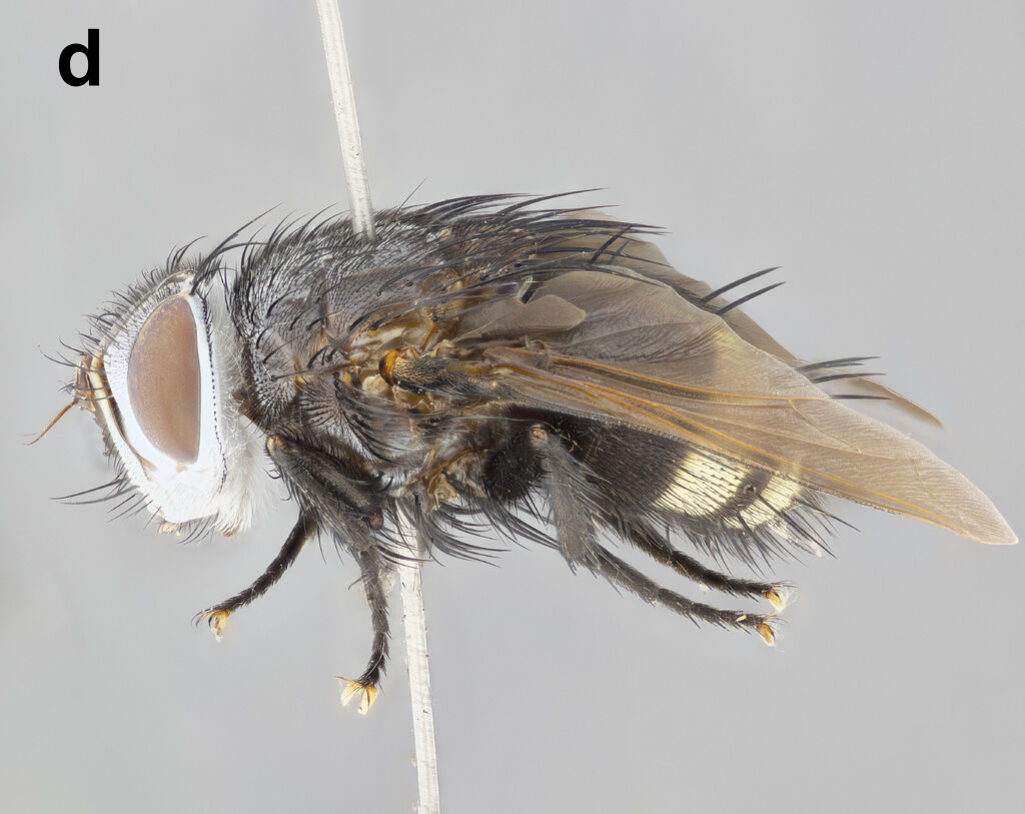
lateral view

**Figure 41a.**
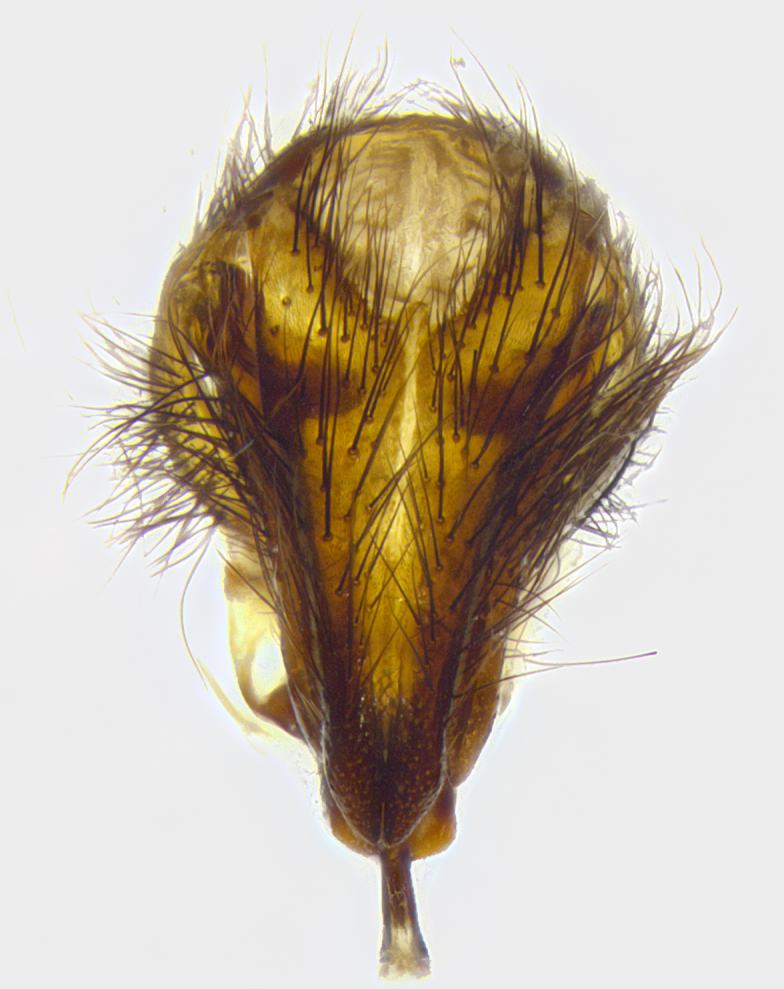
caudal view

**Figure 41b.**
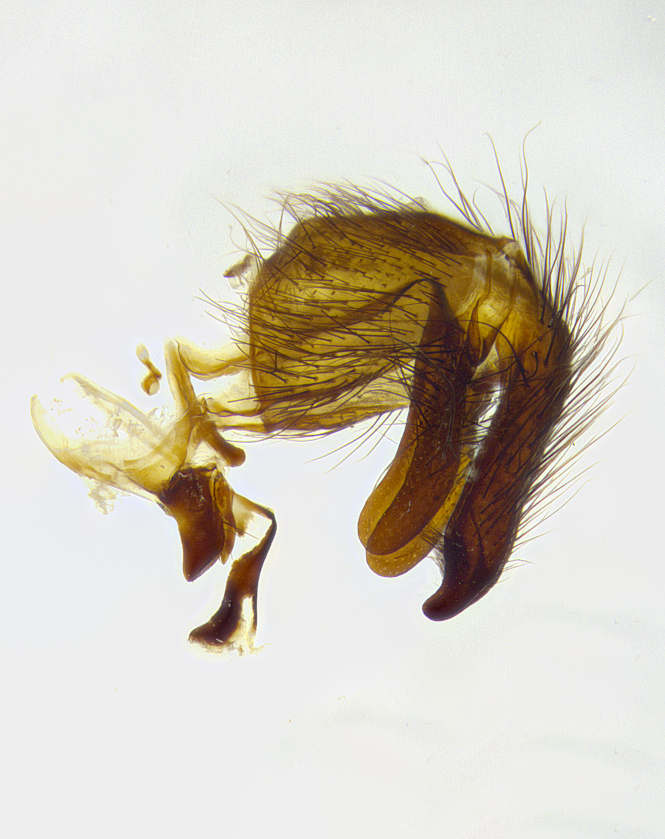
lateral view

**Figure 41c.**
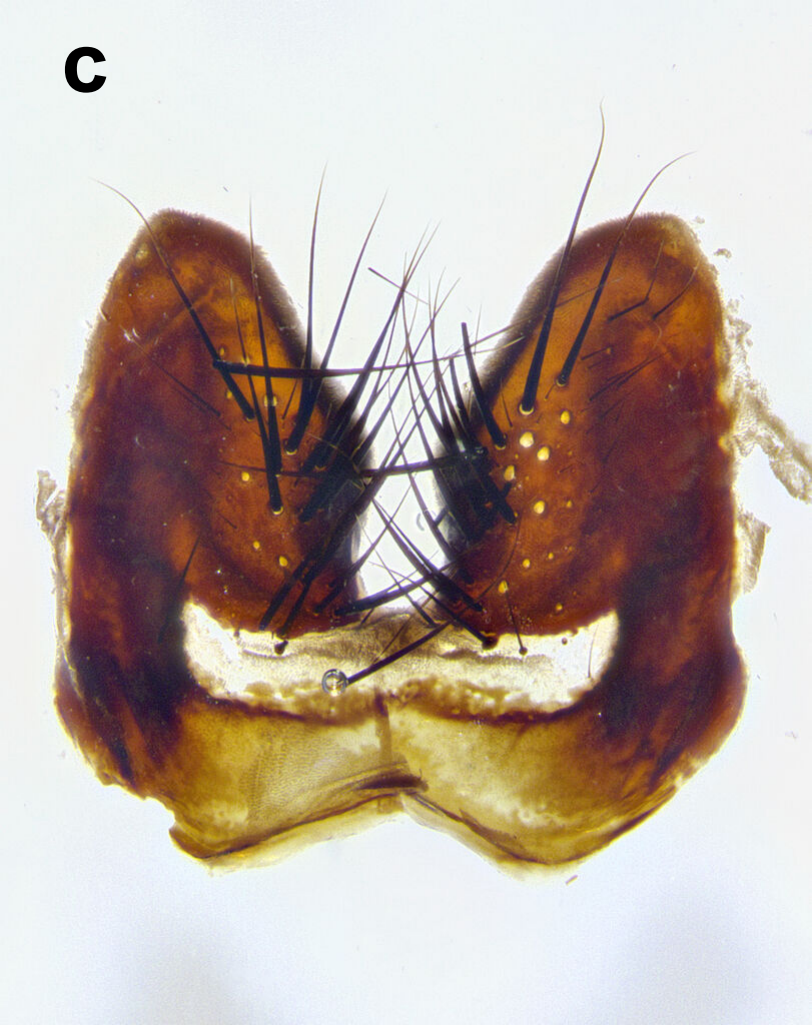
sternite 5, ventral view

**Figure 42a.**
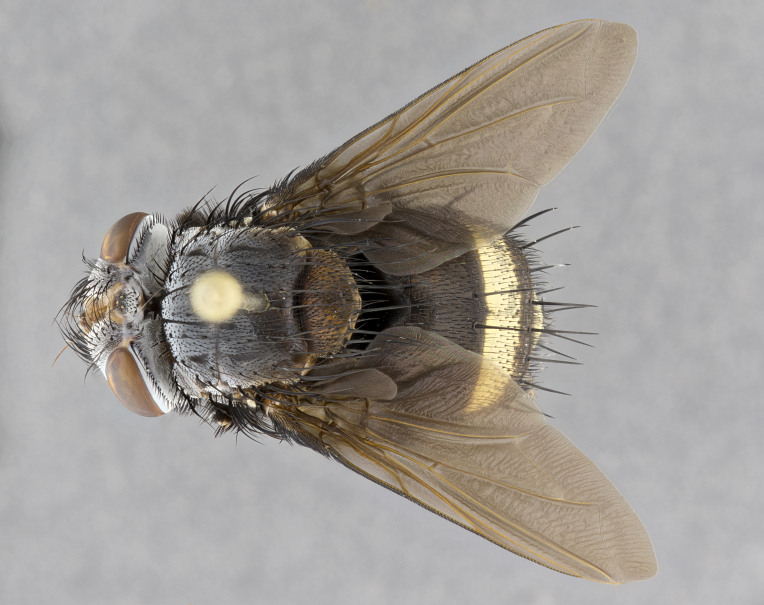
dorsal view

**Figure 42b.**
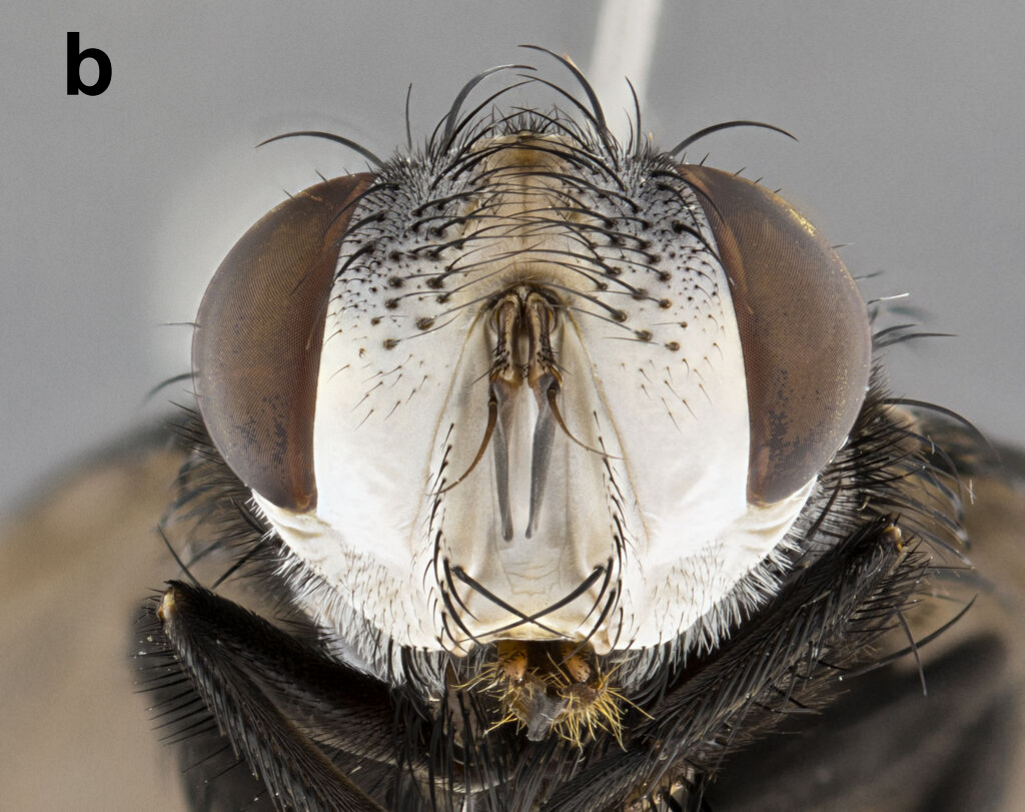
frontal view

**Figure 42c.**
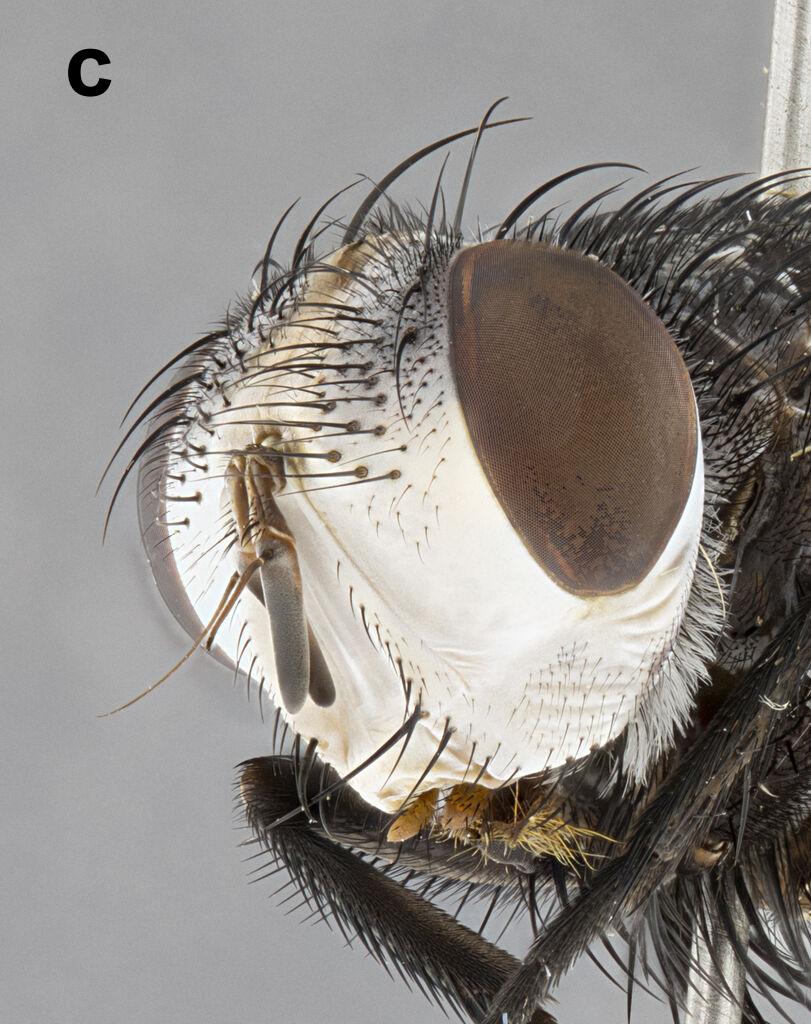
three quarters view

**Figure 42d.**
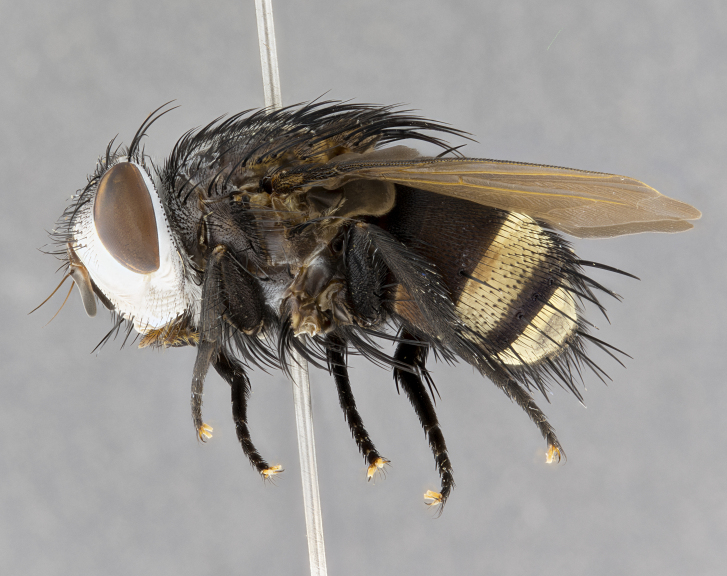
lateral view

**Figure 43a.**
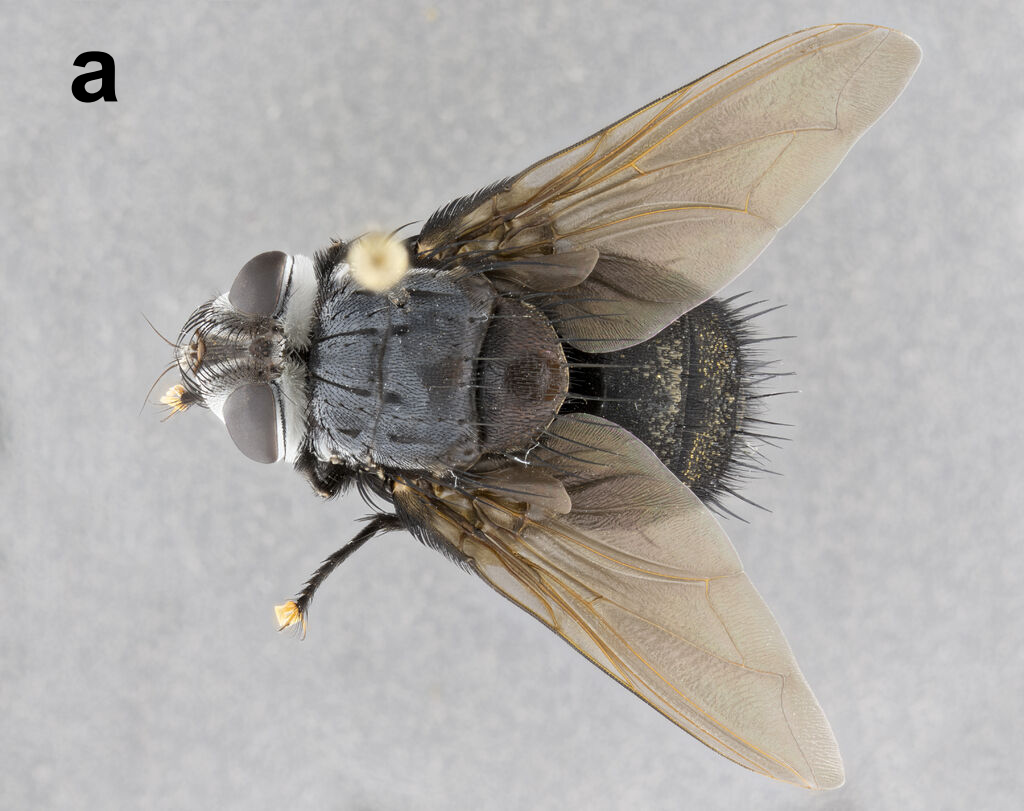
dorsal view

**Figure 43b.**
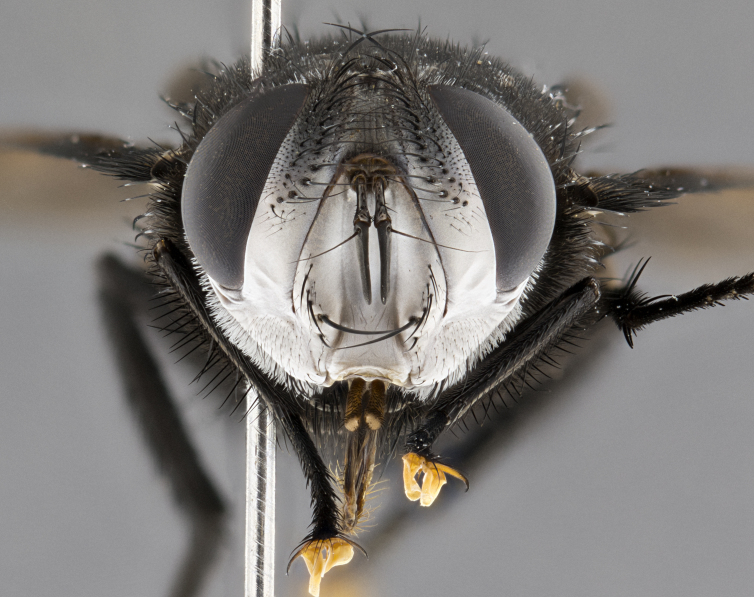
frontal view

**Figure 43c.**
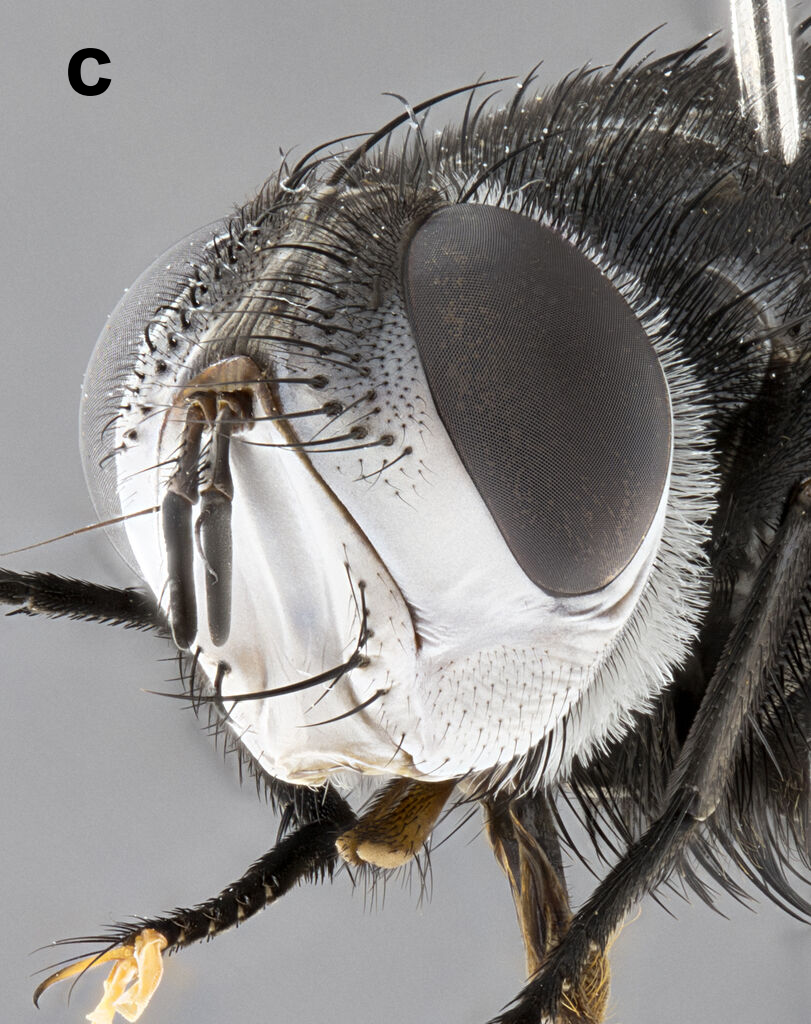
three quarters view

**Figure 43d.**
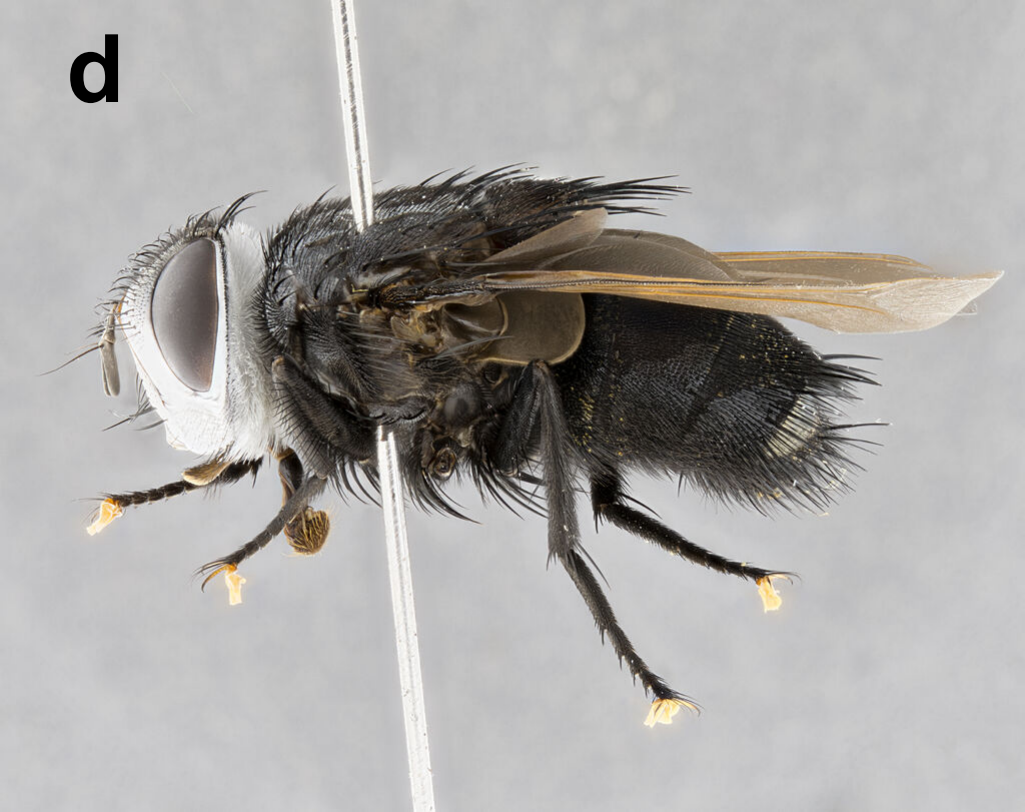
lateral view

**Figure 44a.**
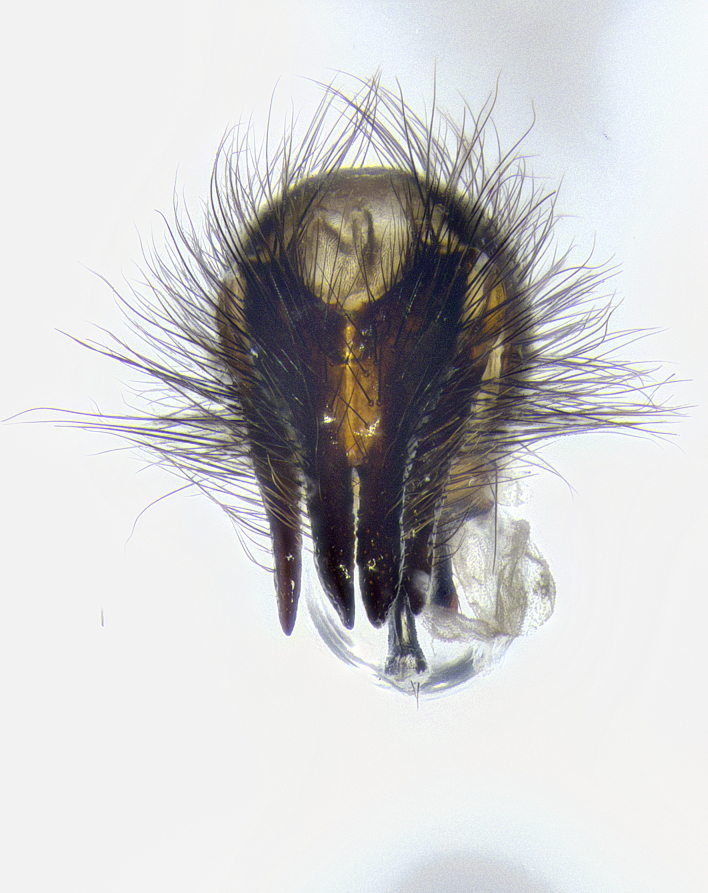
dorsal

**Figure 44b.**
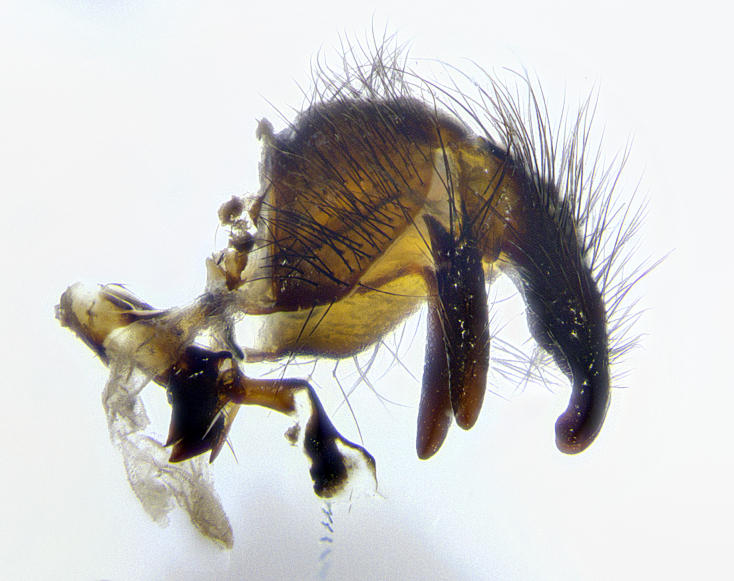
lateral

**Figure 44c.**
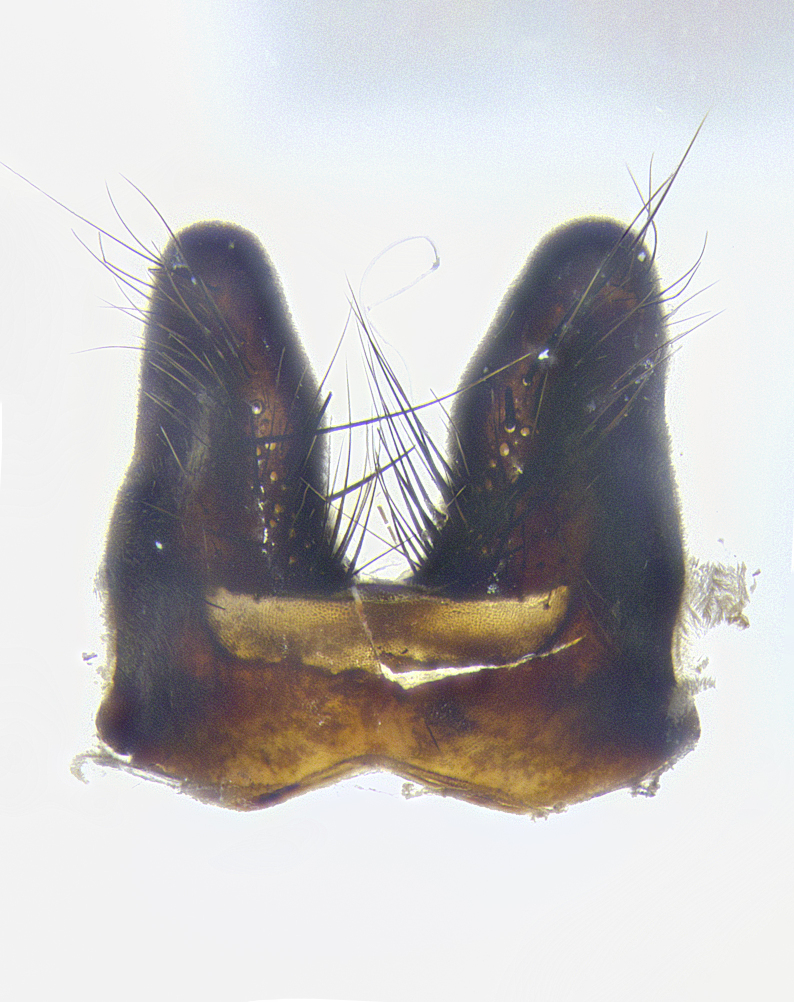
ST5

**Figure 45a.**
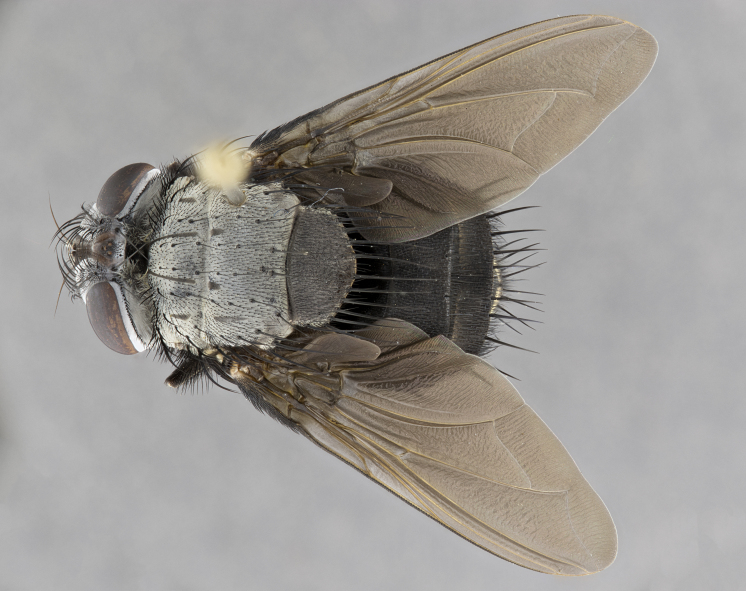
dorsal view

**Figure 45b.**
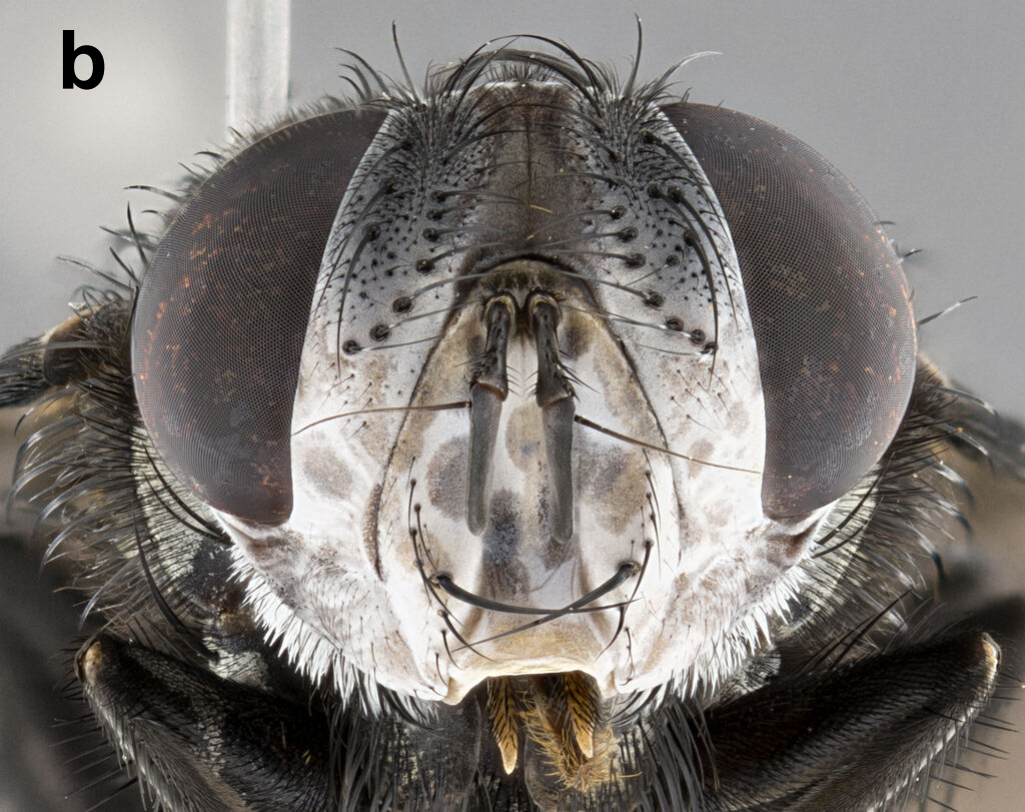
frontal view

**Figure 45c.**
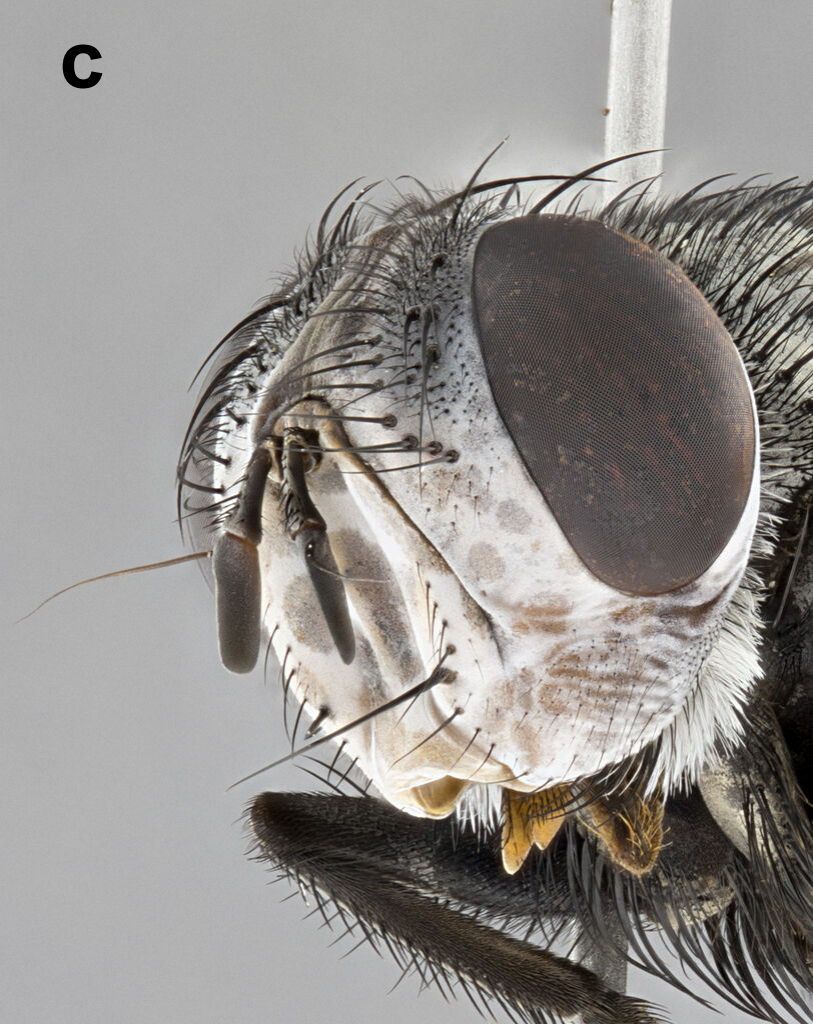
three quarters view

**Figure 45d.**
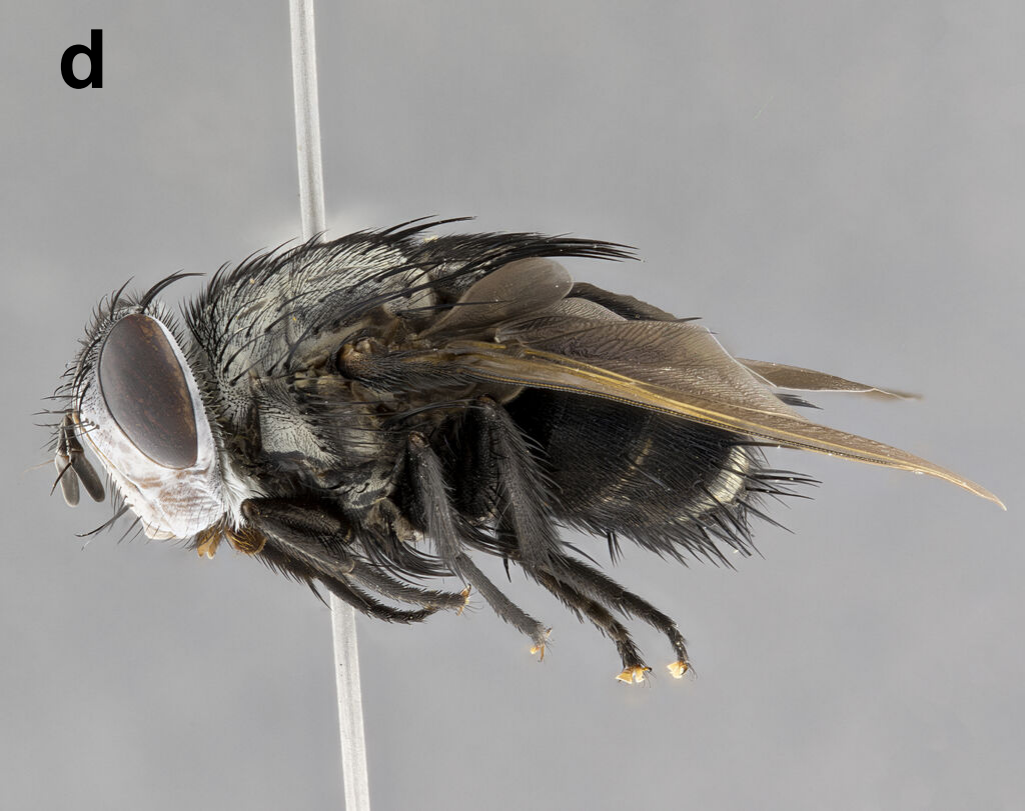
lateral view

**Figure 46a.**
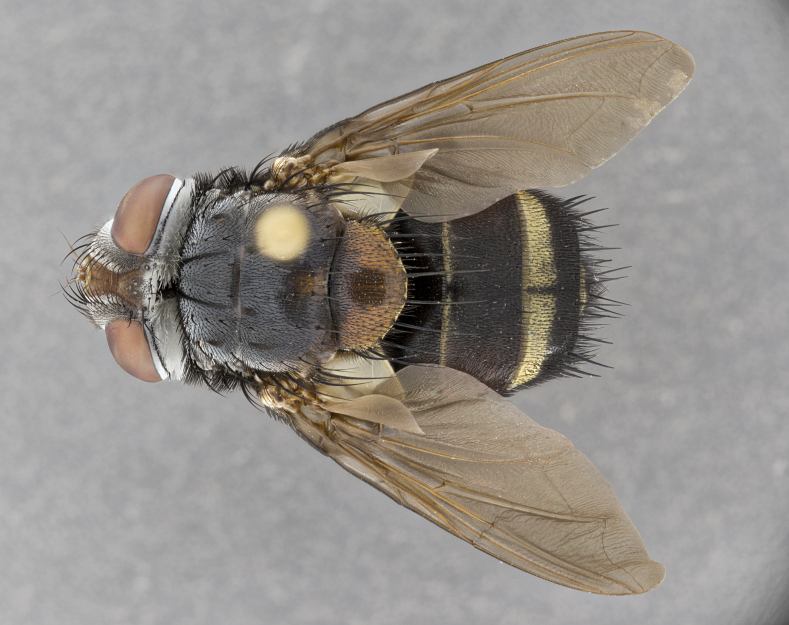
dorsal view

**Figure 46b.**
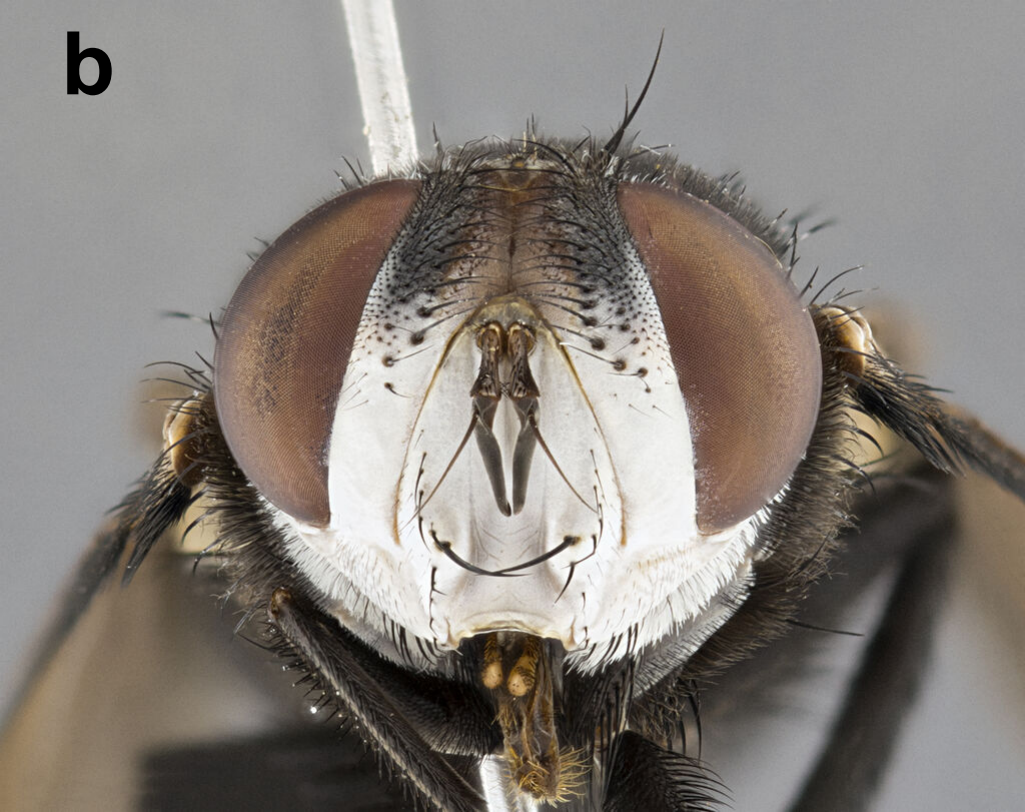
frontal view

**Figure 46c.**
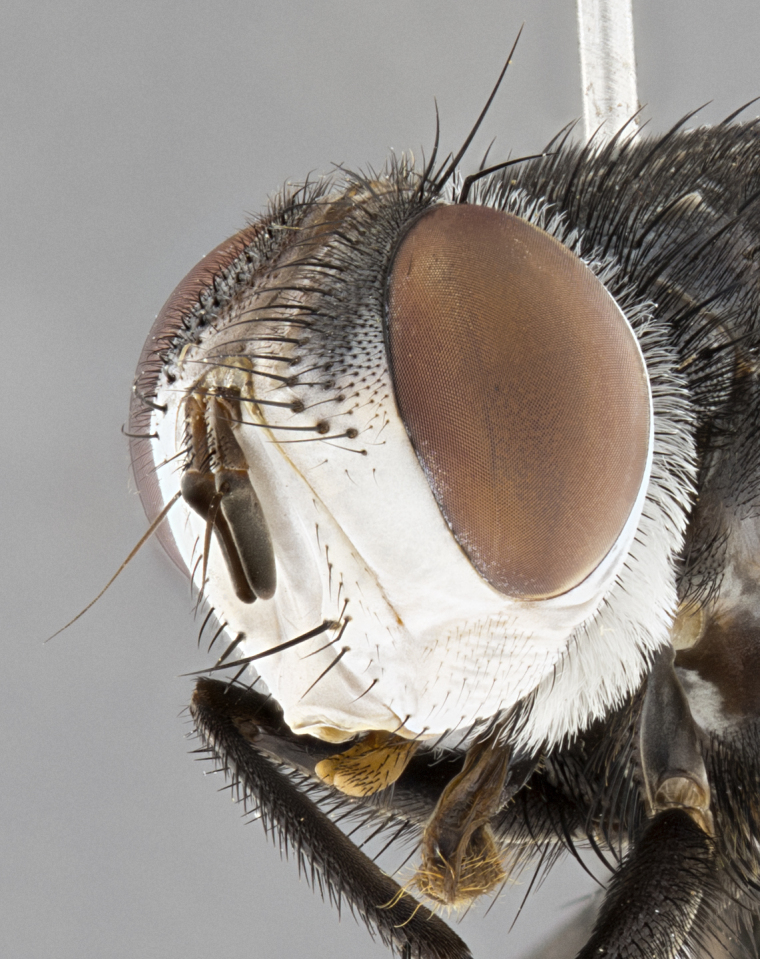
three quarters view

**Figure 46d.**
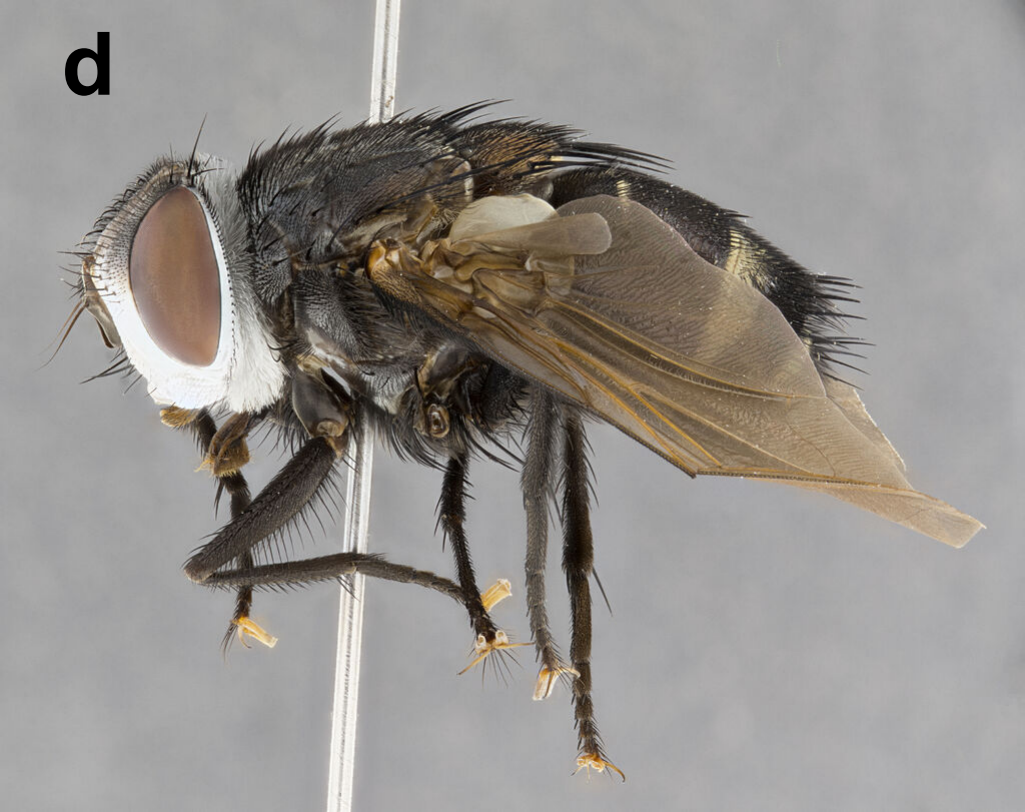
lateral view

**Figure 47a.**
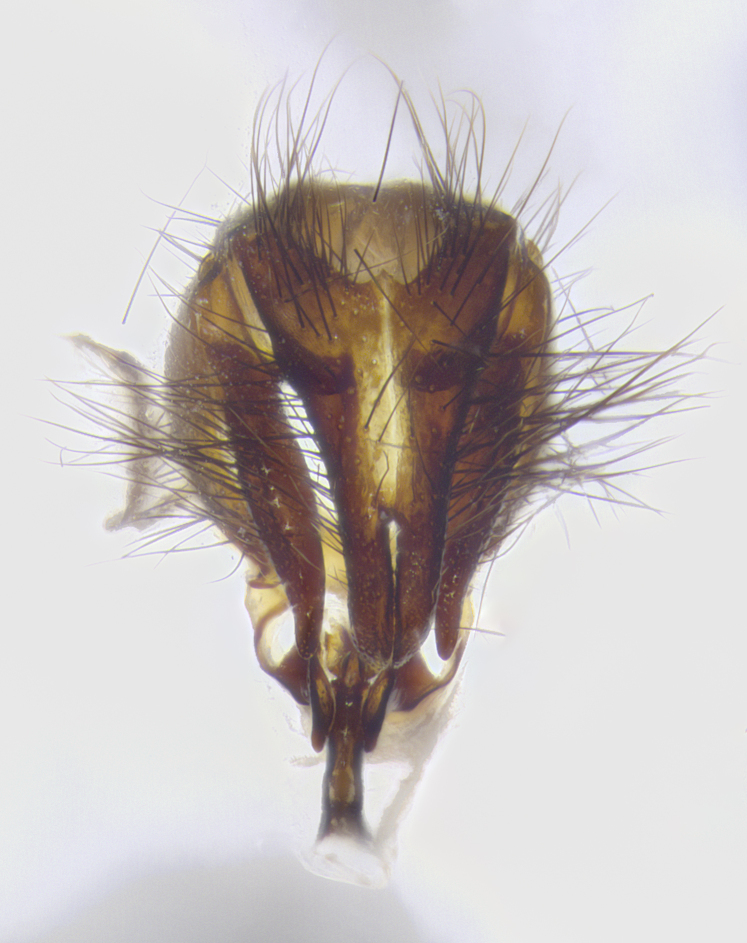
caudal view

**Figure 47b.**
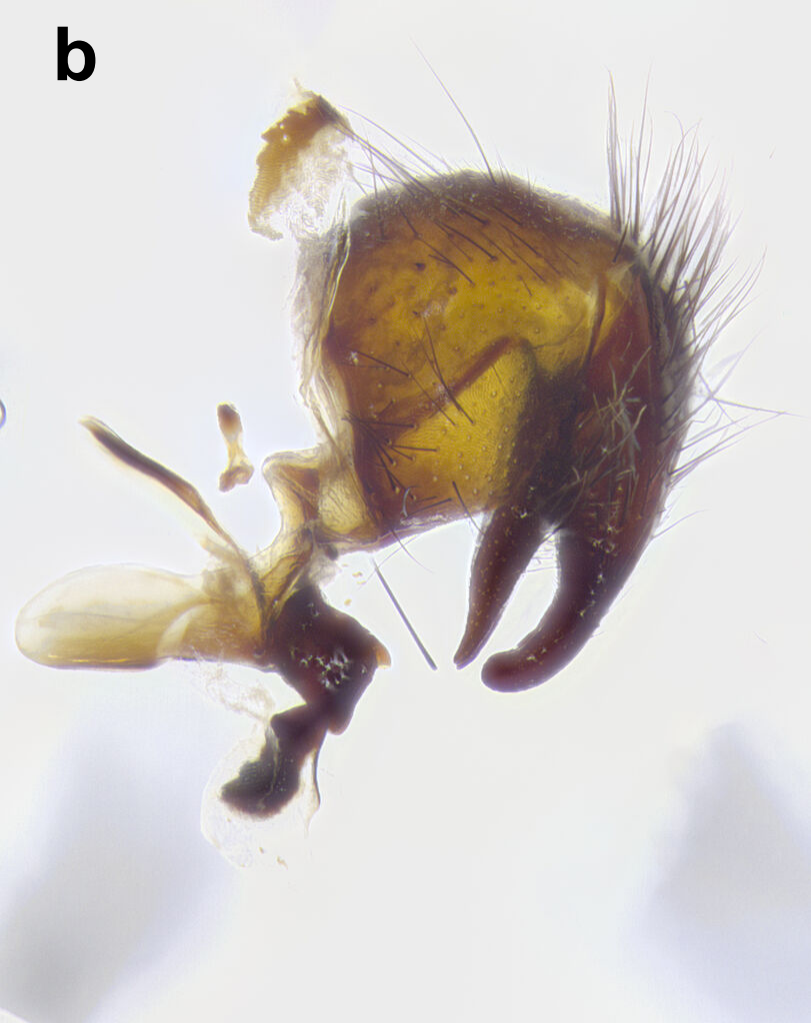
lateral view

**Figure 47c.**
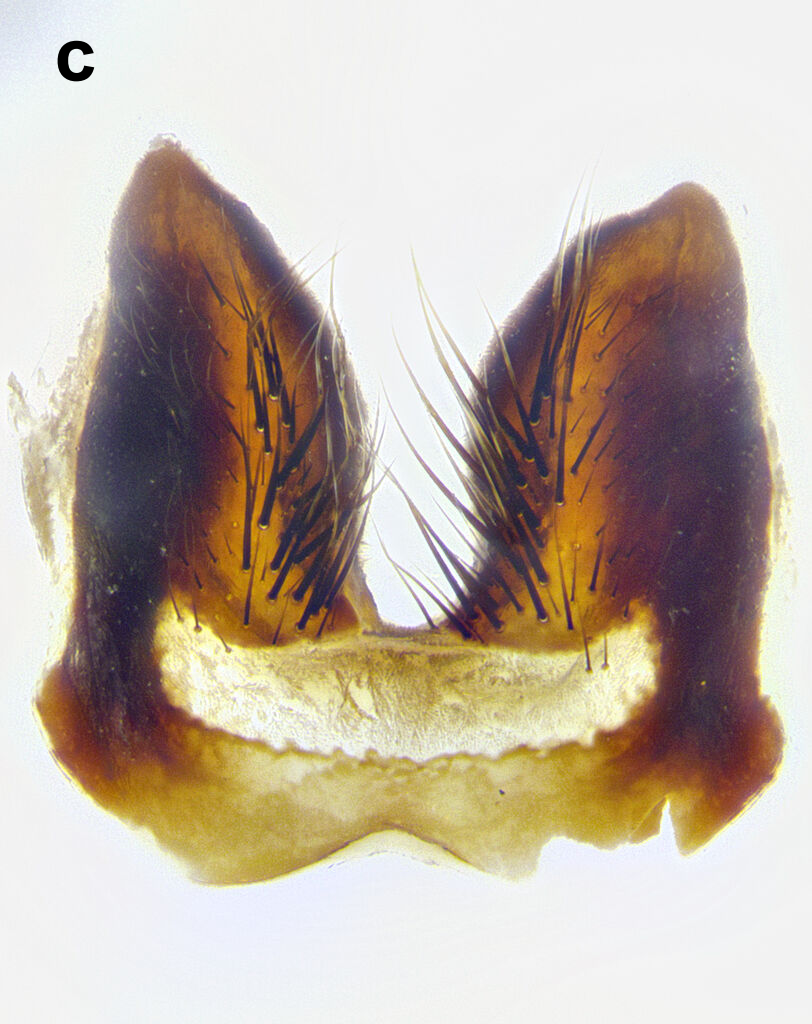
sternite 5, ventral view

**Figure 48a.**
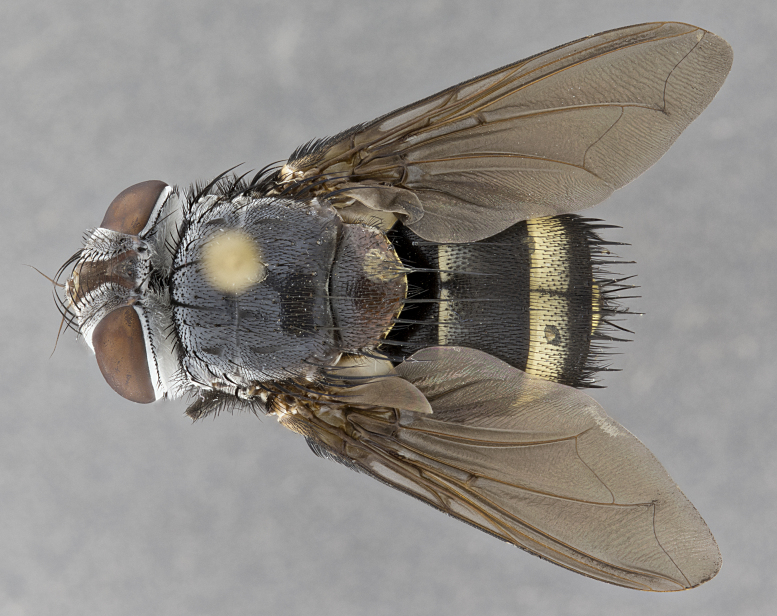
dorsal view

**Figure 48b.**
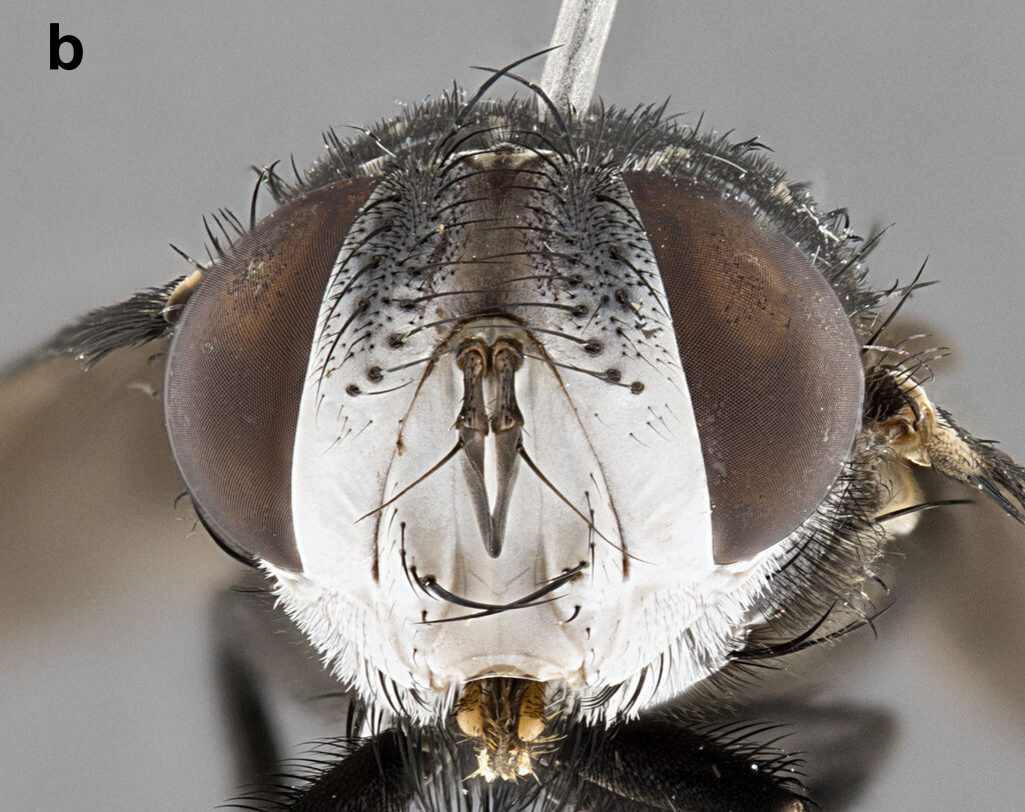
frontal view

**Figure 48c.**
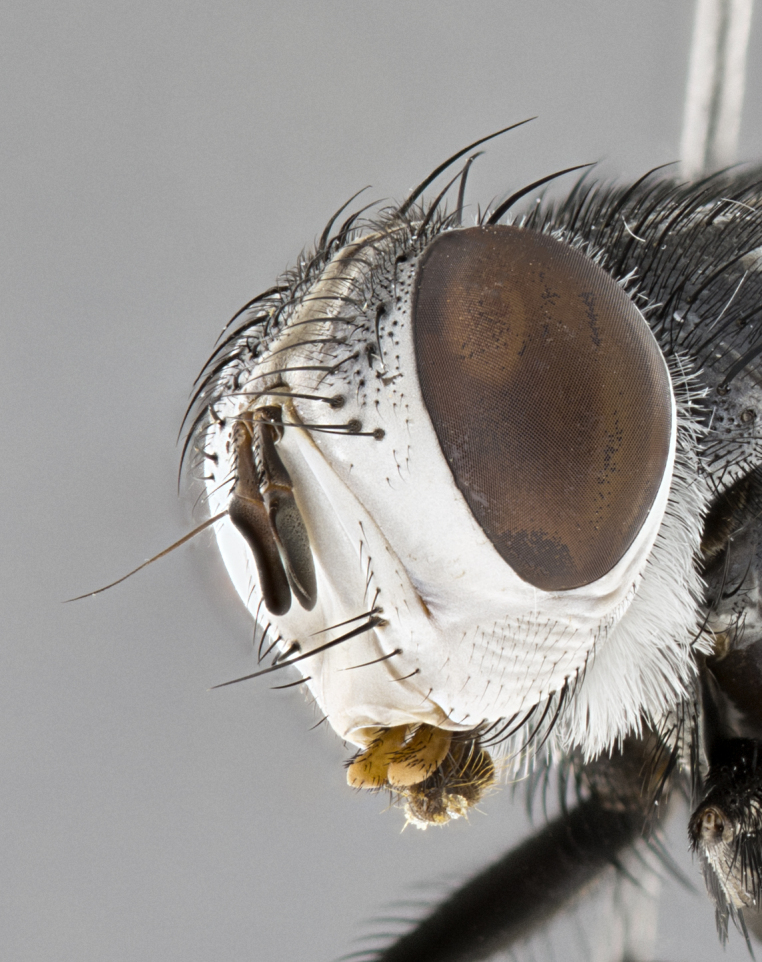
three quarters view

**Figure 48d.**
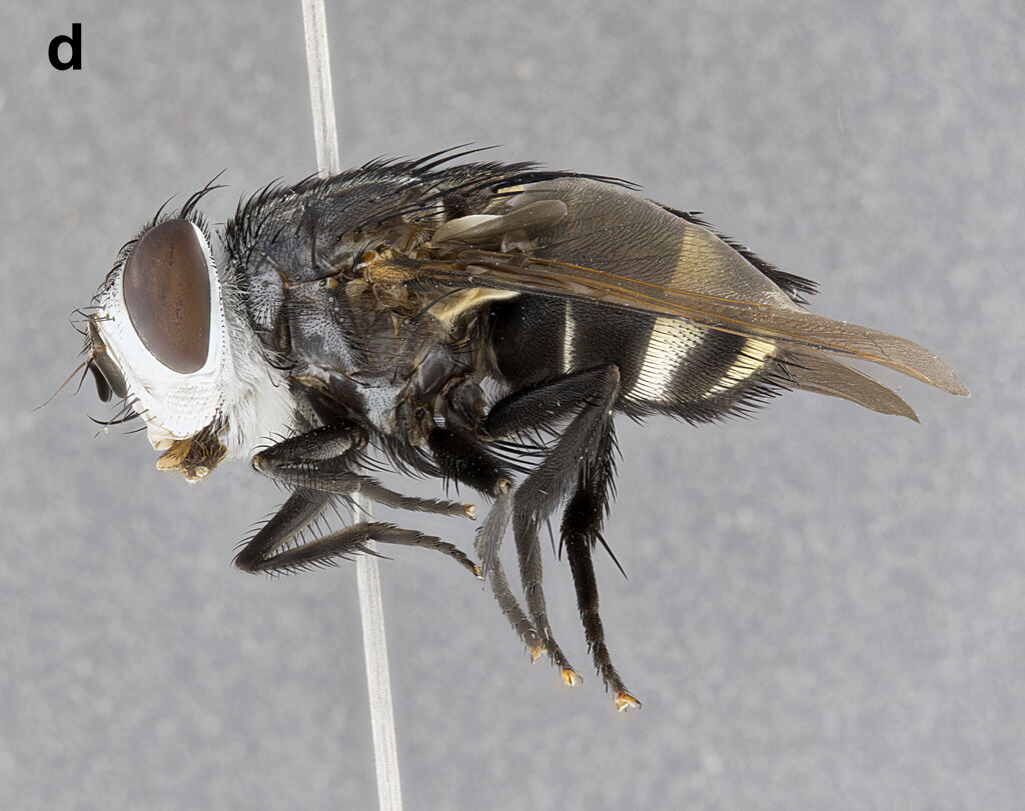
lateral view

**Figure 49a.**
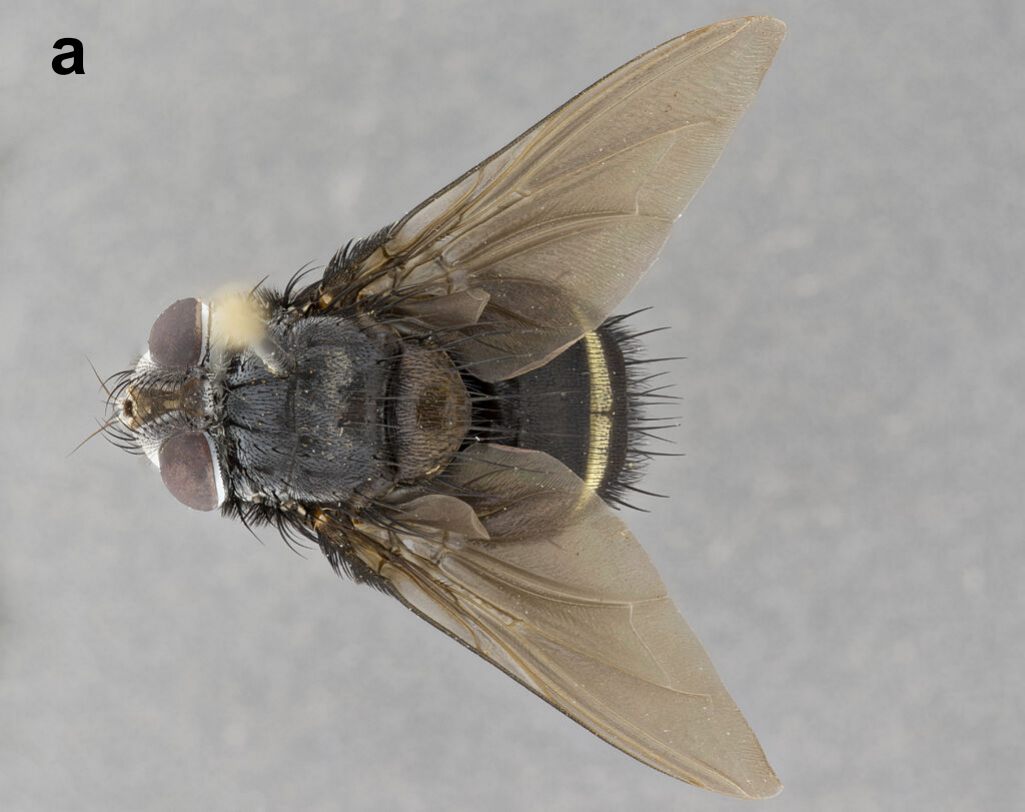
dorsal view

**Figure 49b.**
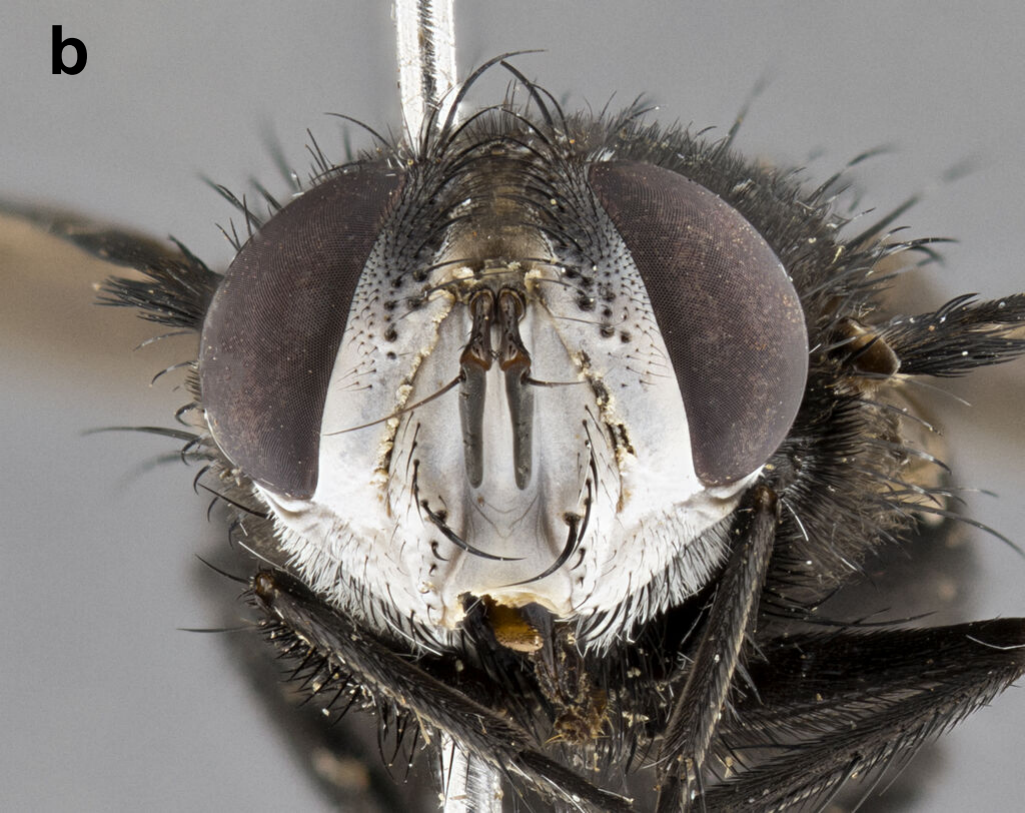
frontal view

**Figure 49c.**
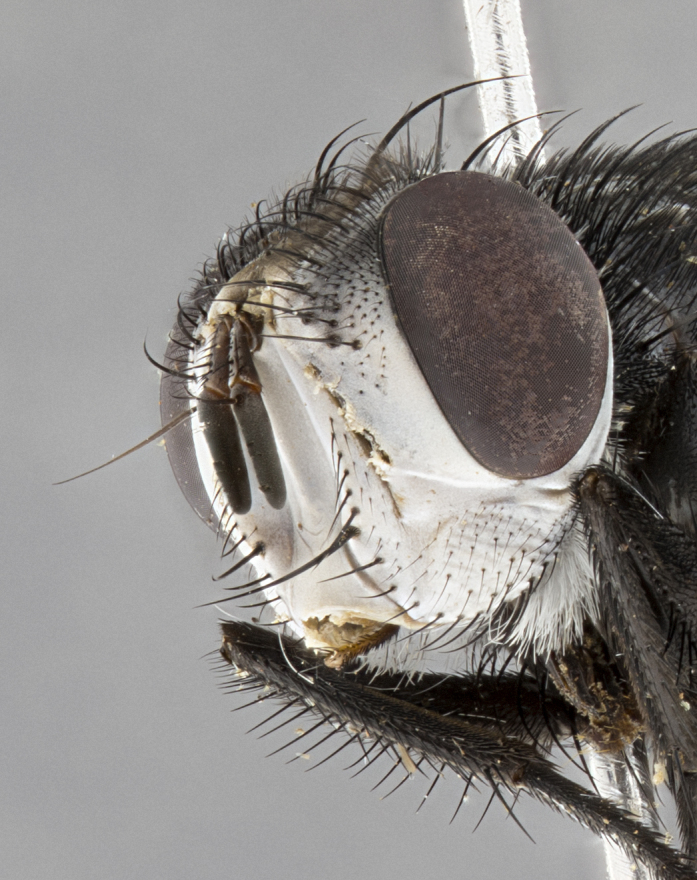
three quarters view

**Figure 49d.**
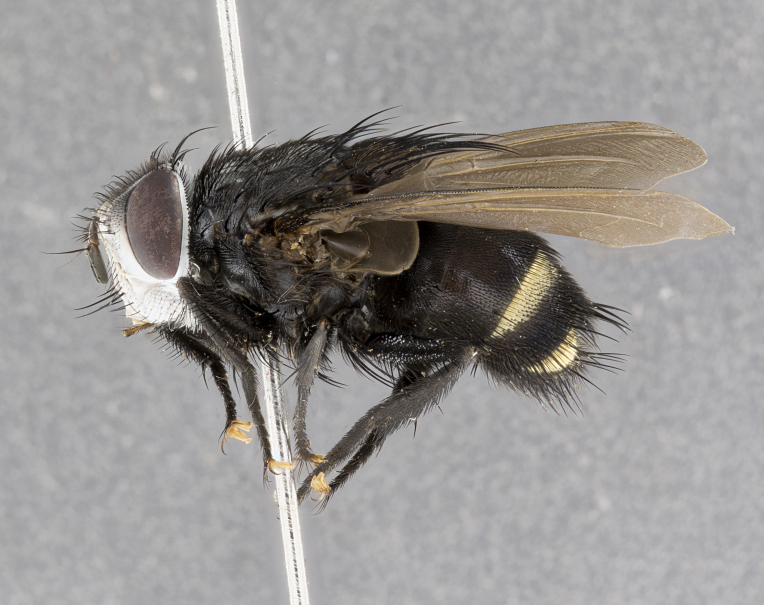
lateral view

**Figure 50a.**
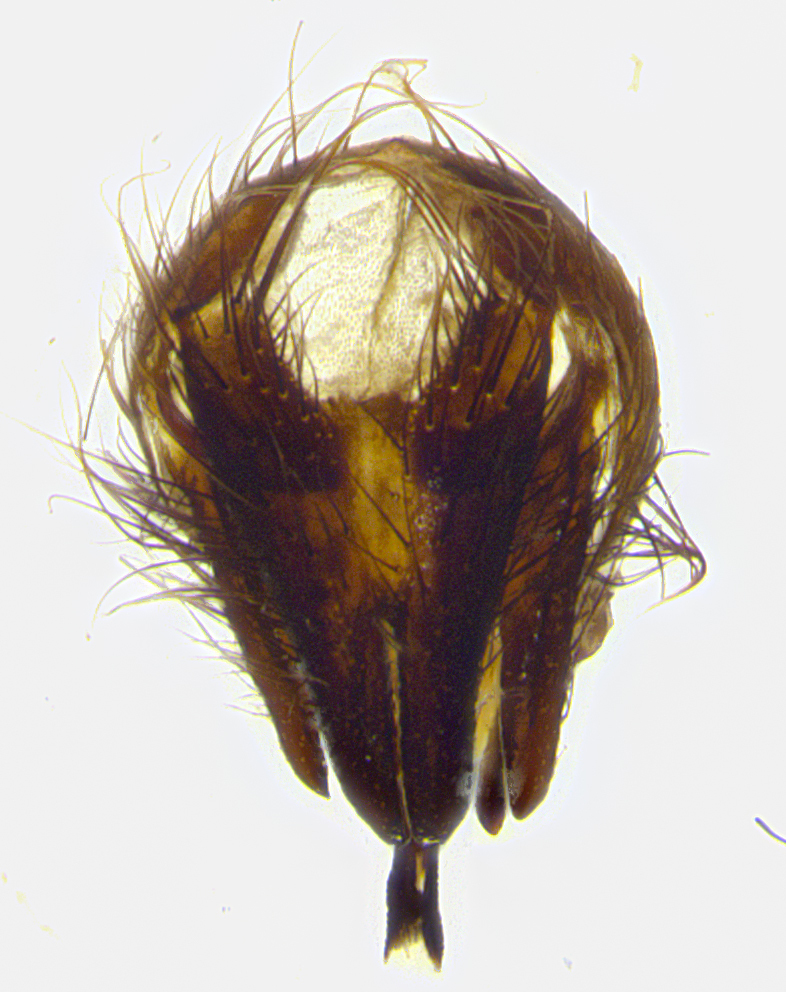
caudal view

**Figure 50b.**
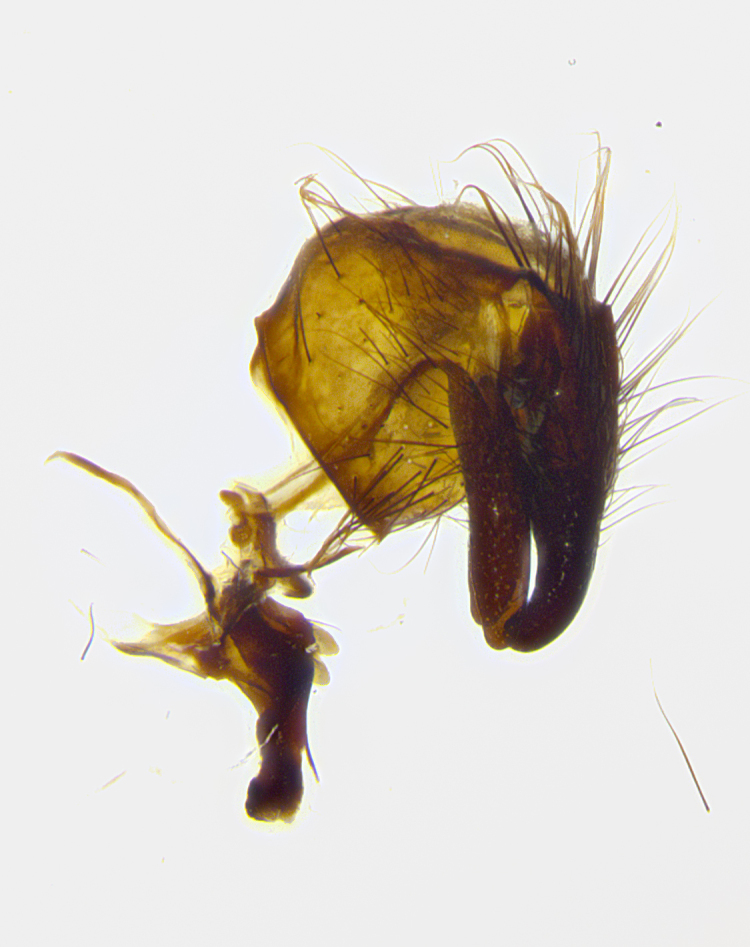
lateral view

**Figure 50c.**
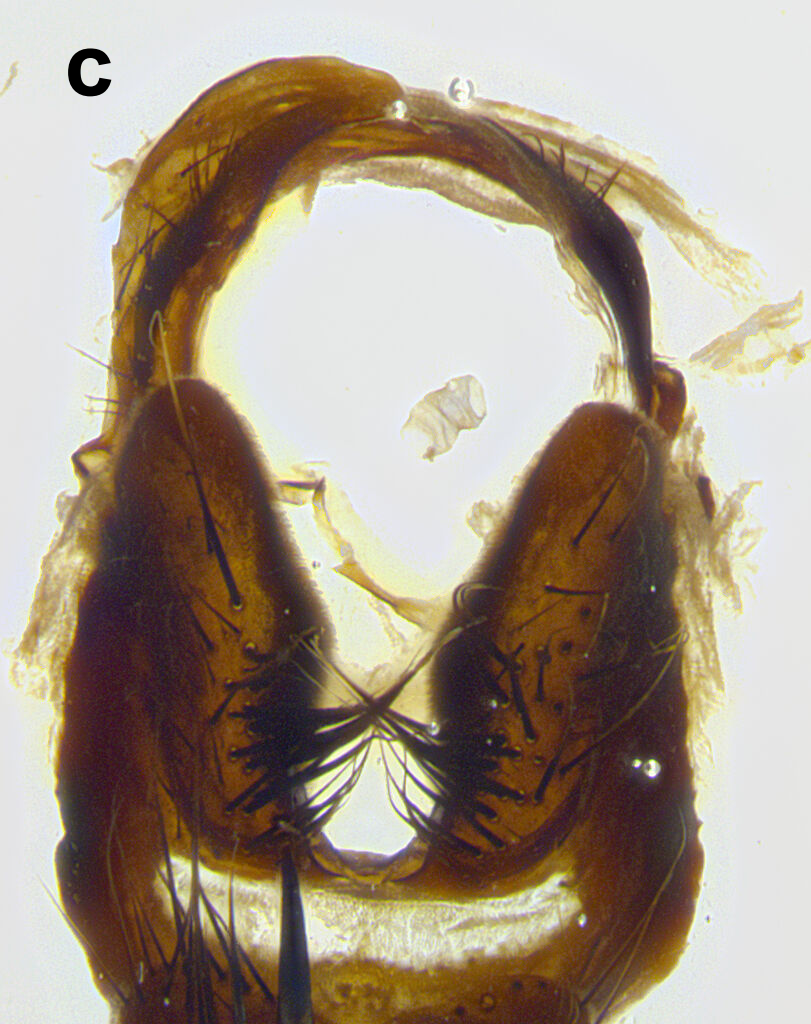
sternite 5, ventral view

**Figure 51a.**
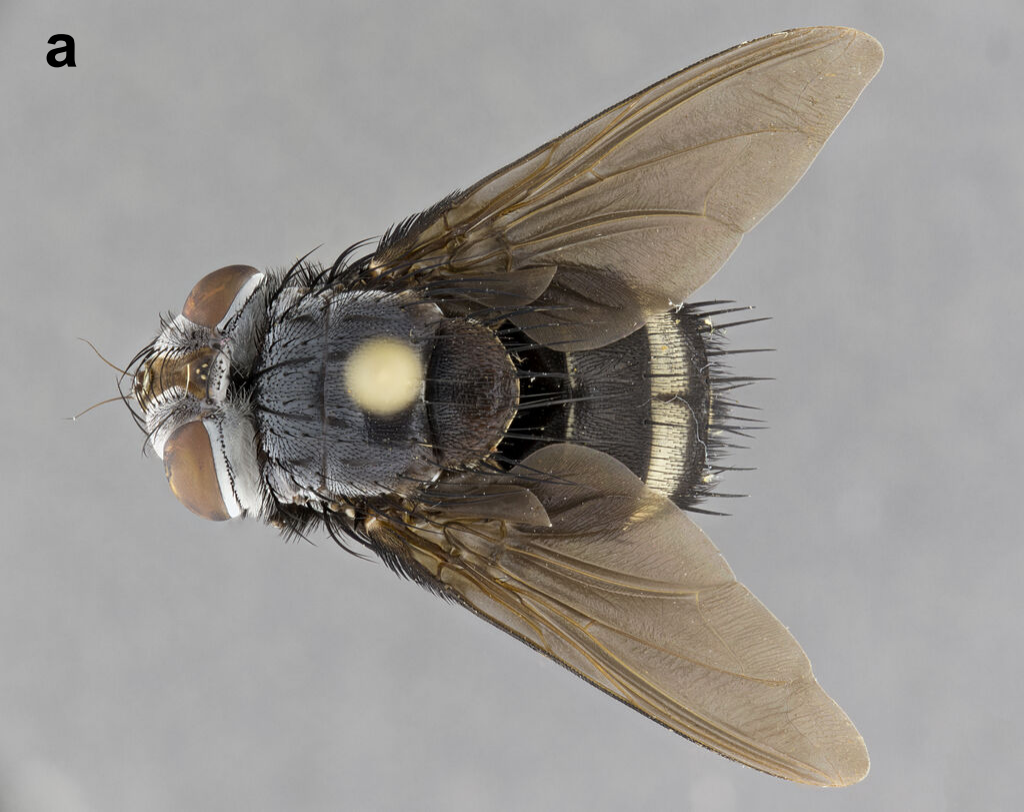
dorsal view

**Figure 51b.**
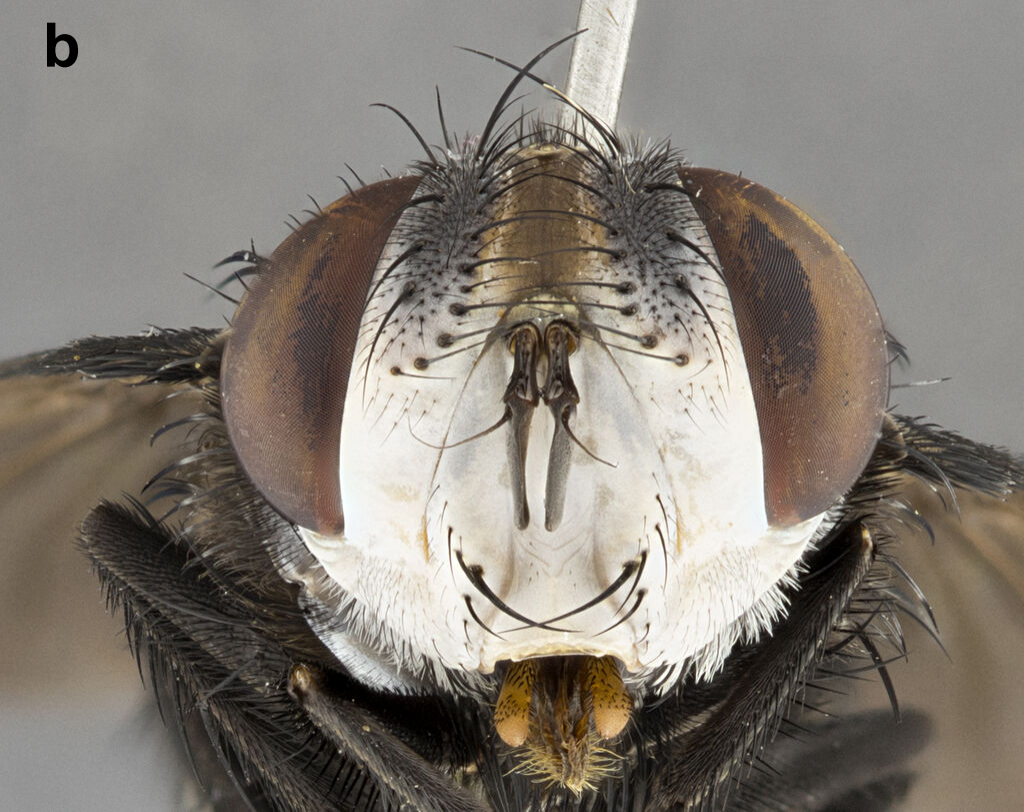
frontal view

**Figure 51c.**
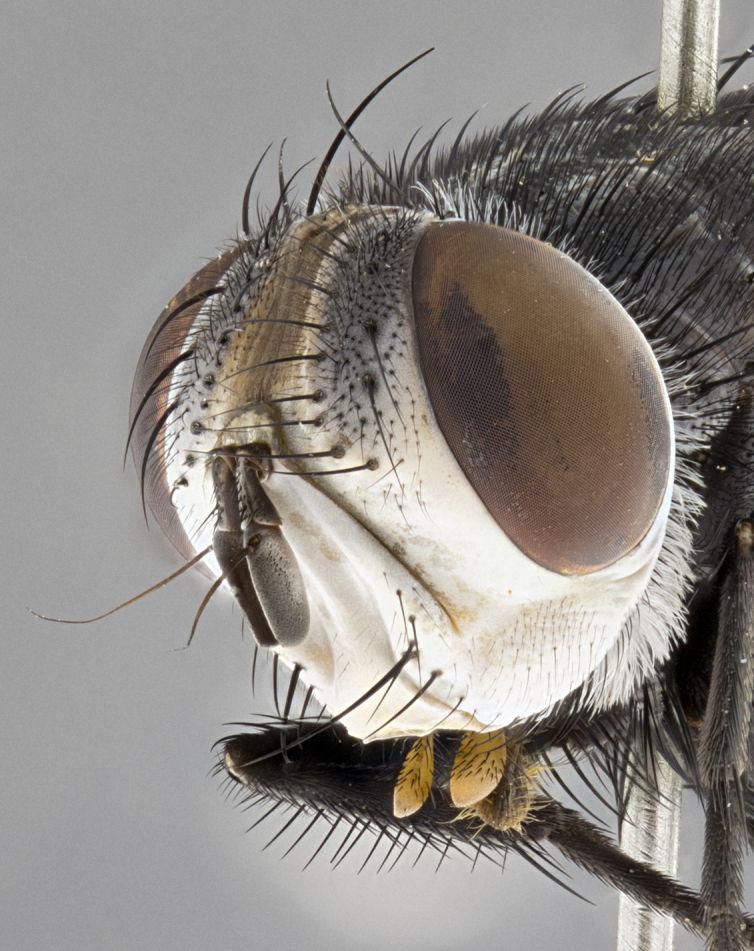
three quarters view

**Figure 51d.**
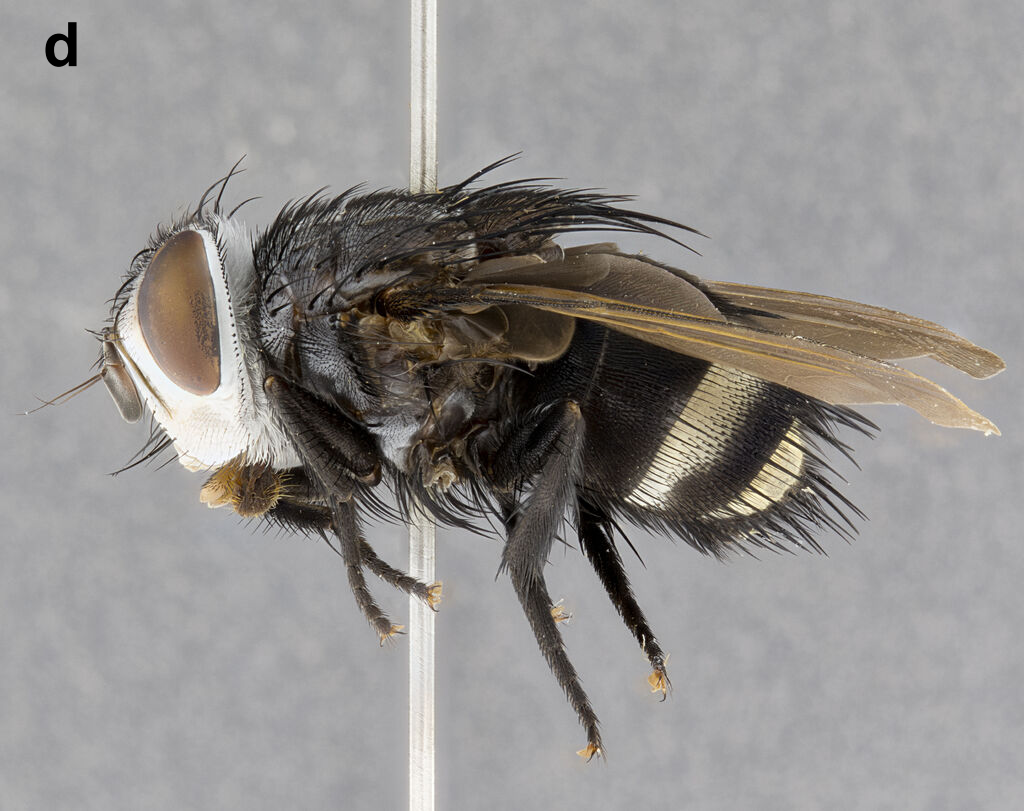
lateral view

**Figure 52a.**
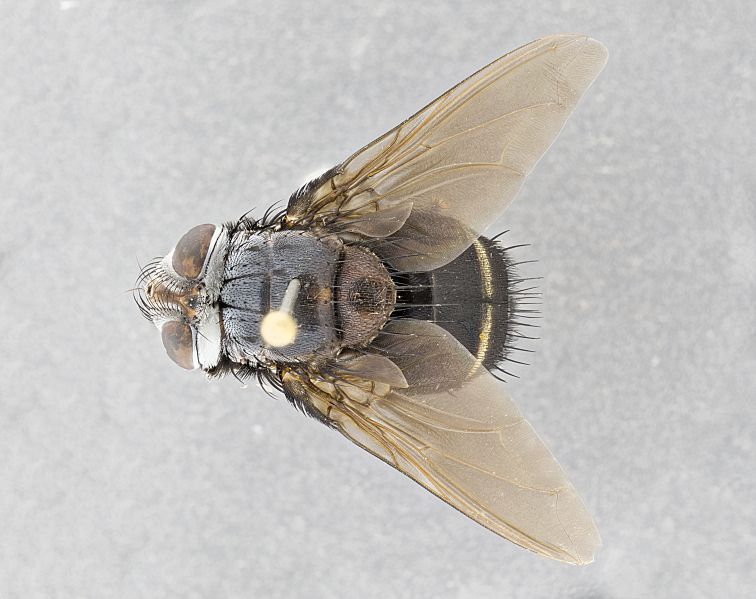
dorsal view

**Figure 52b.**
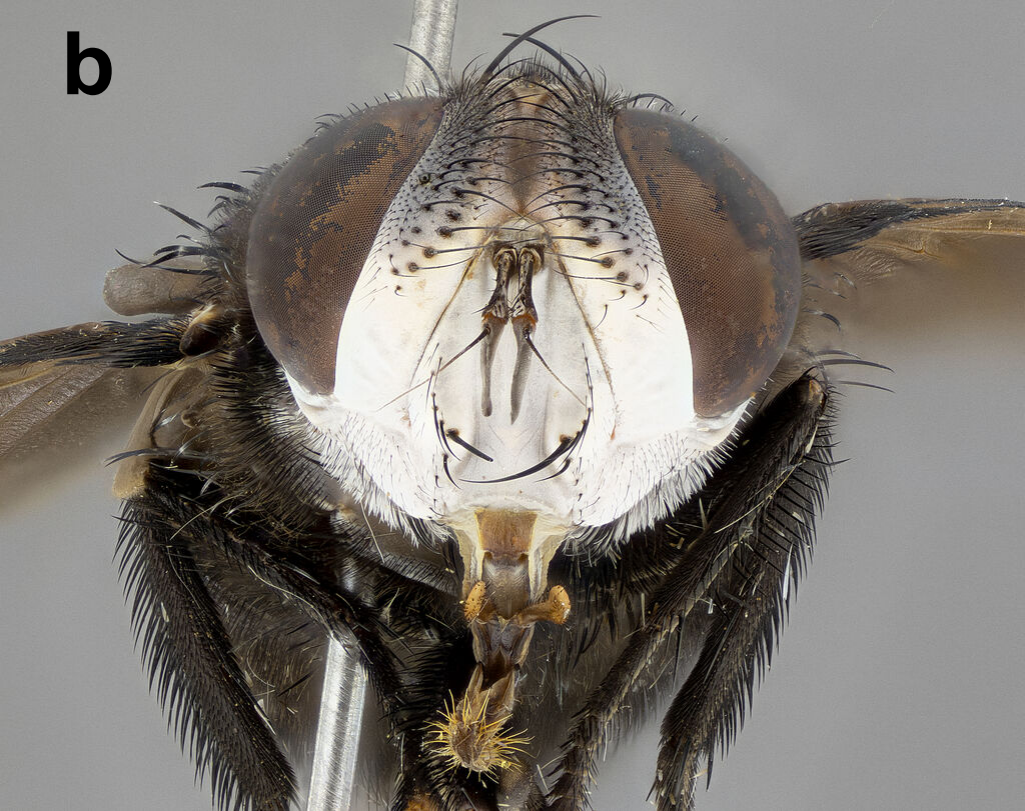
frontal view

**Figure 52c.**
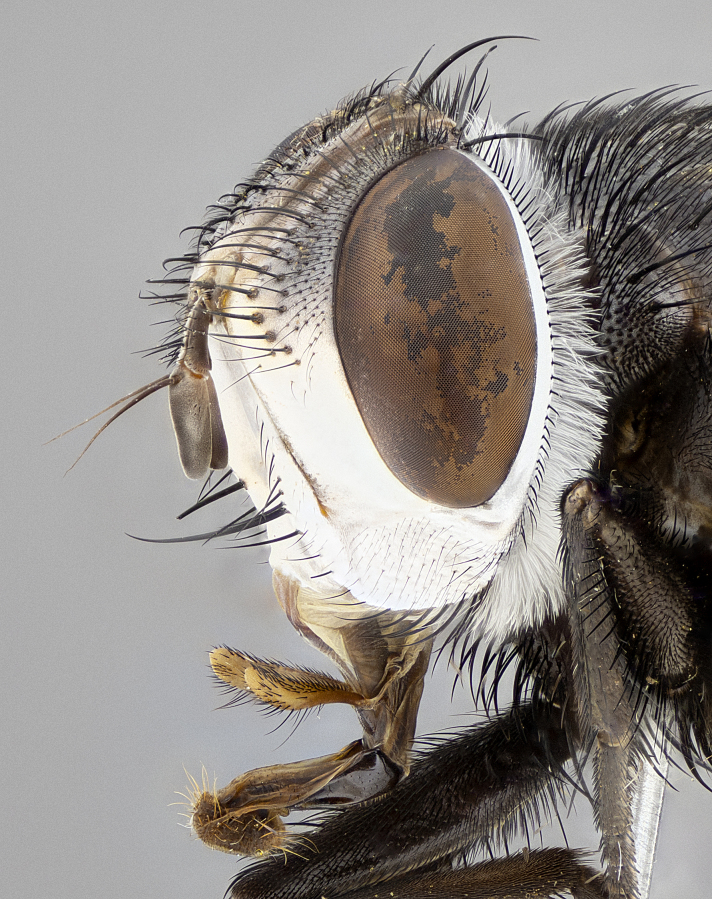
three quarters view

**Figure 52d.**
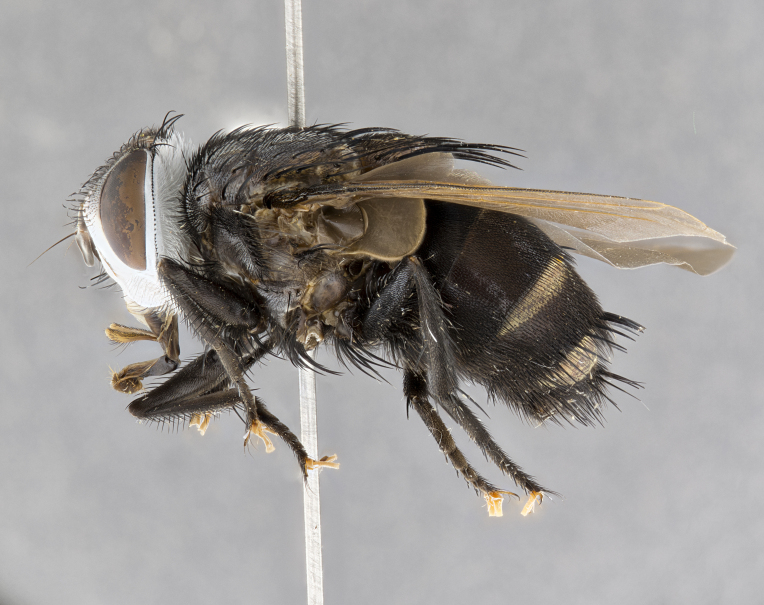
lateral view

**Figure 53a.**
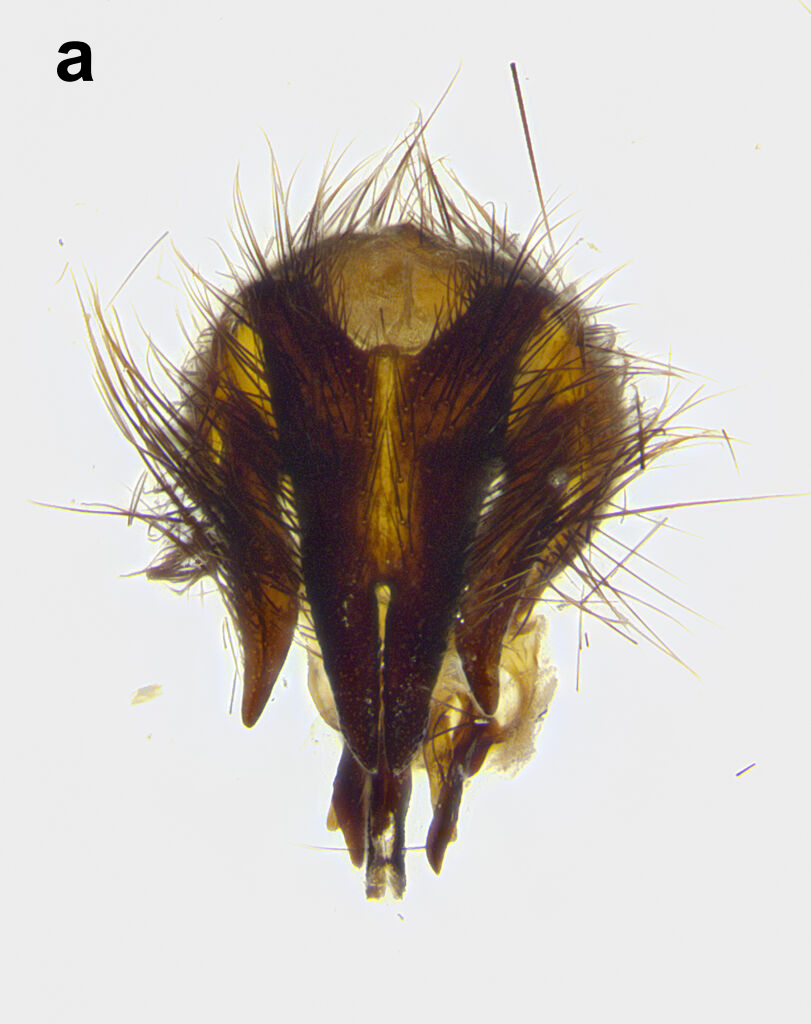
caudal view

**Figure 53b.**
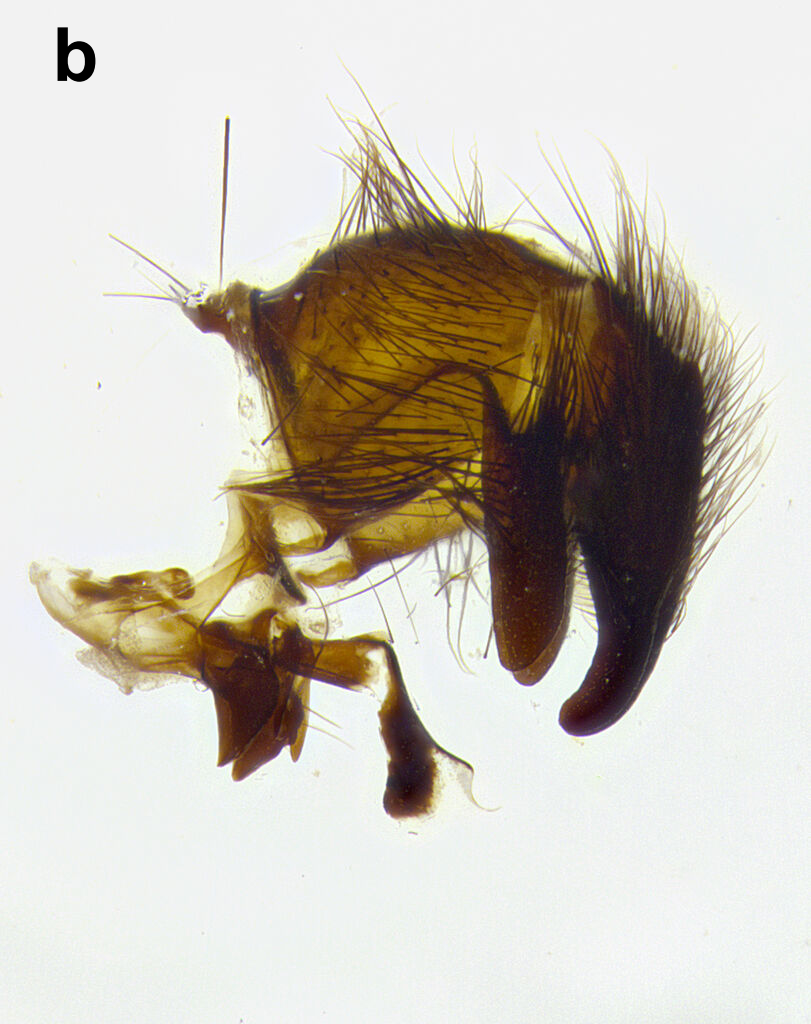
lateral view

**Figure 53c.**
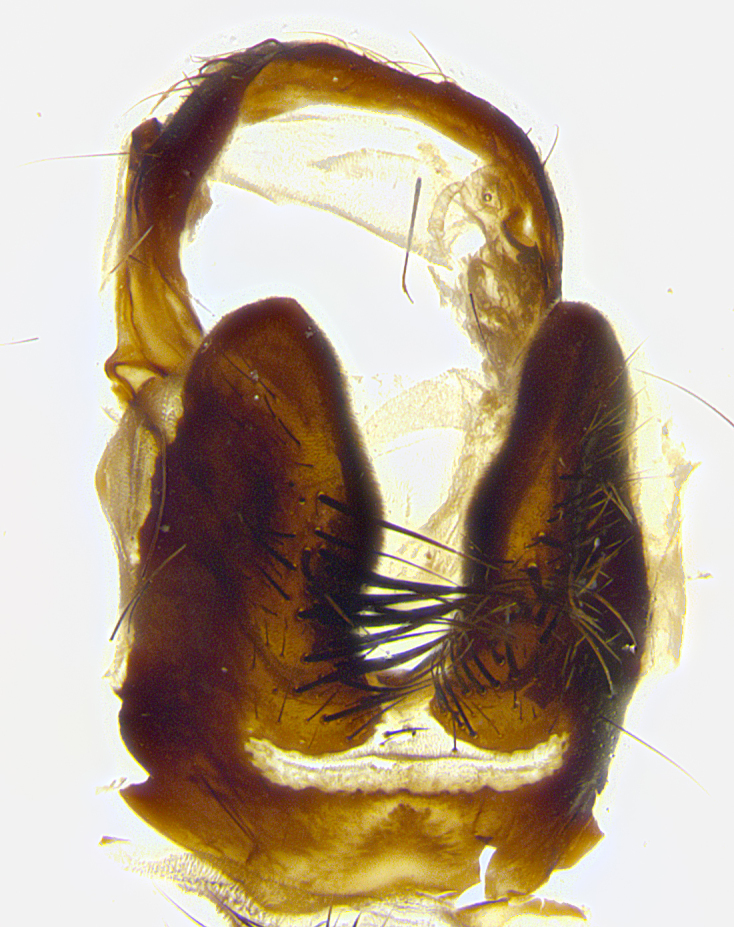
sternite 5, ventral view

**Figure 54a.**
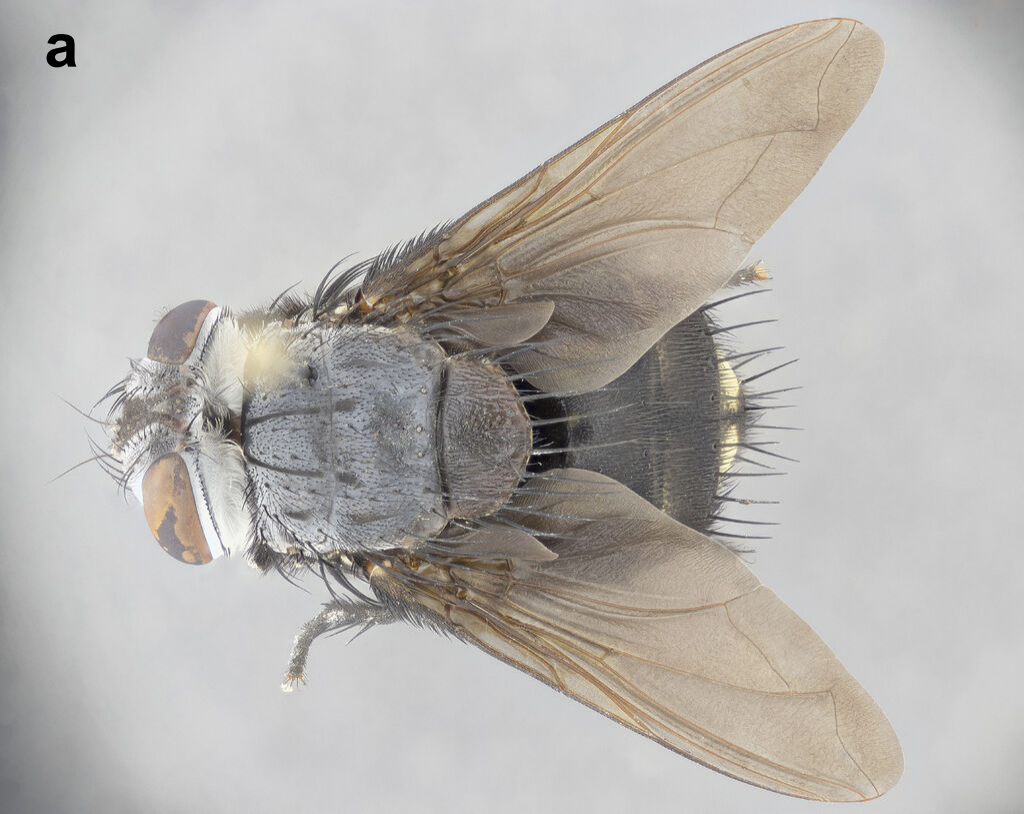
dorsal view

**Figure 54b.**
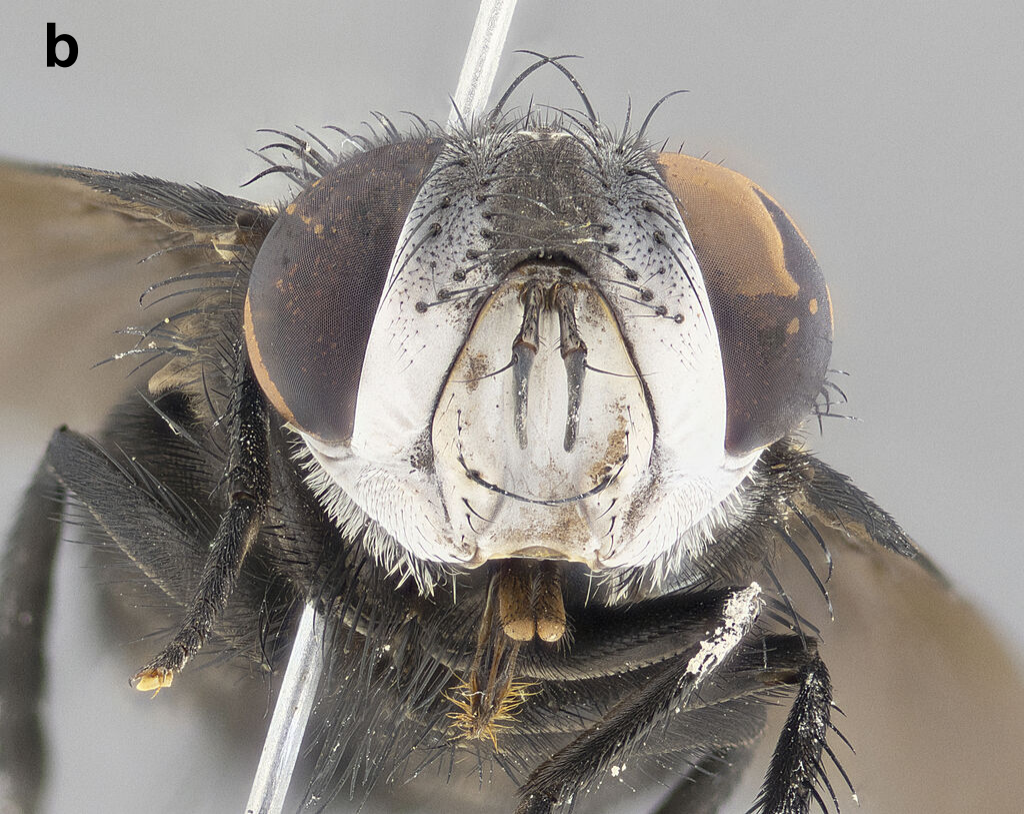
frontal view

**Figure 54c.**
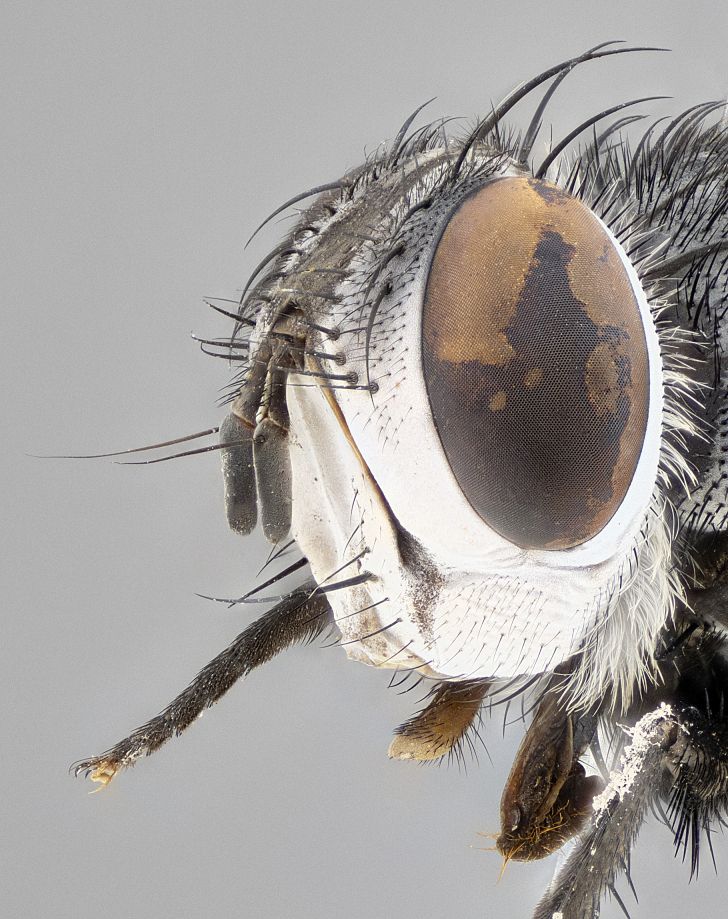
three quarters view

**Figure 54d.**
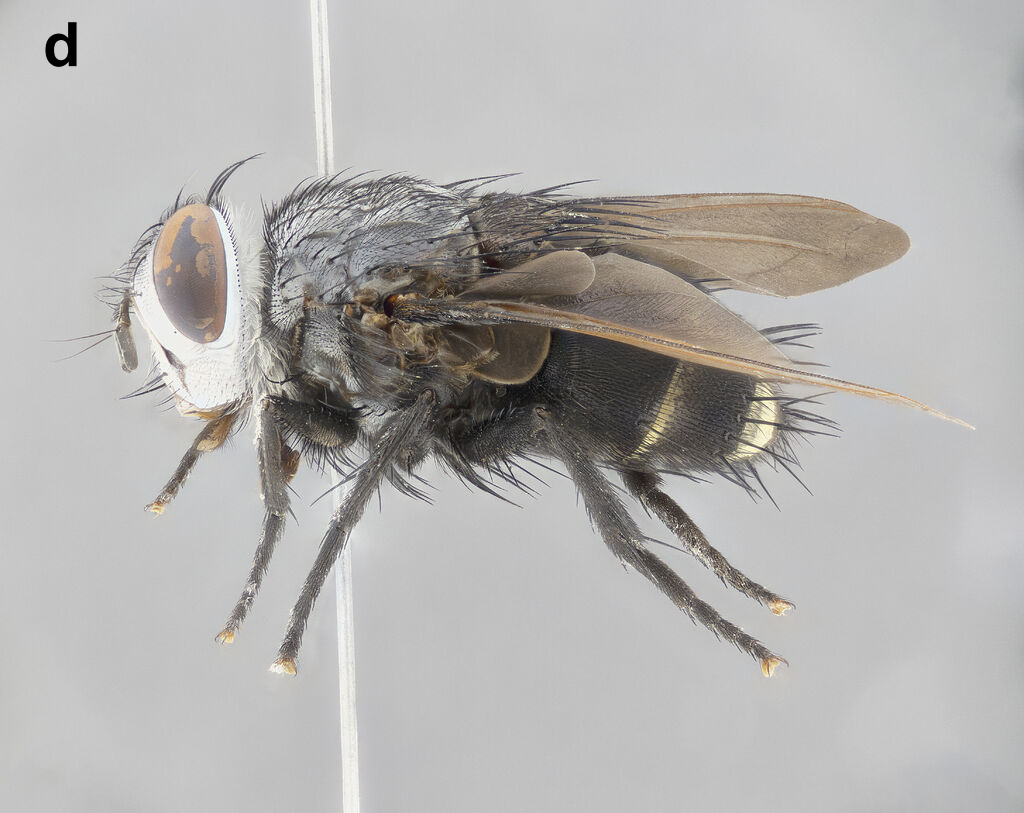
lateral view

**Figure 55a.**
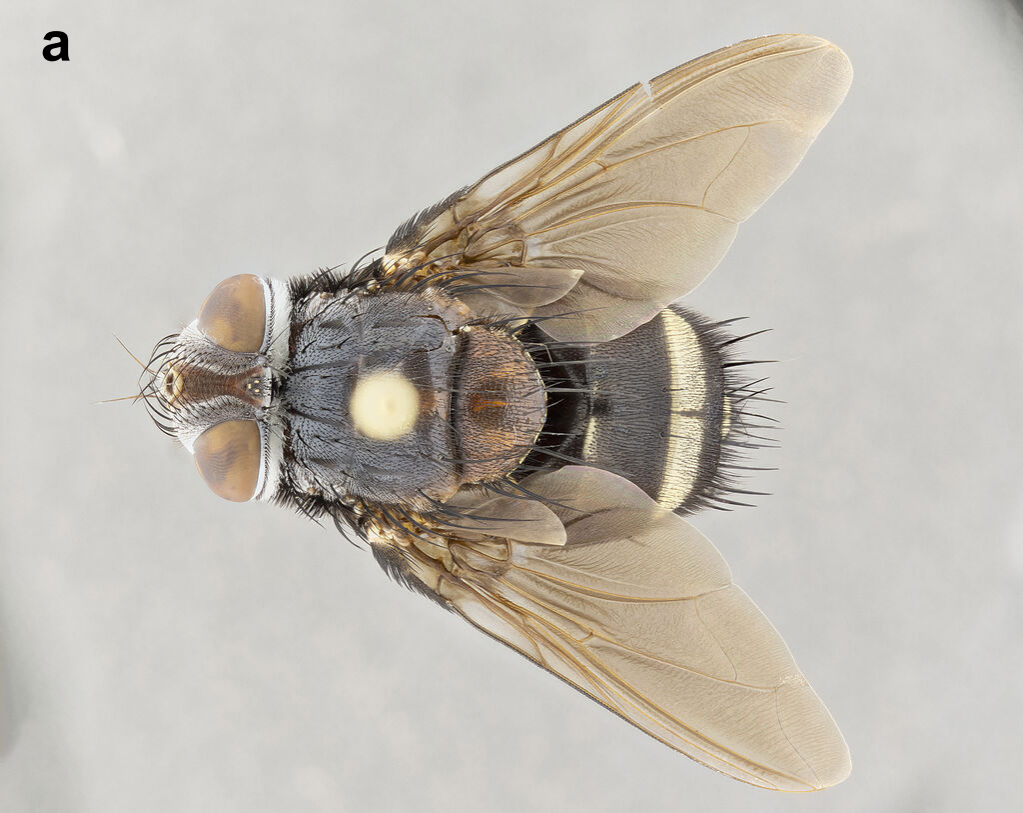
dorsal view

**Figure 55b.**
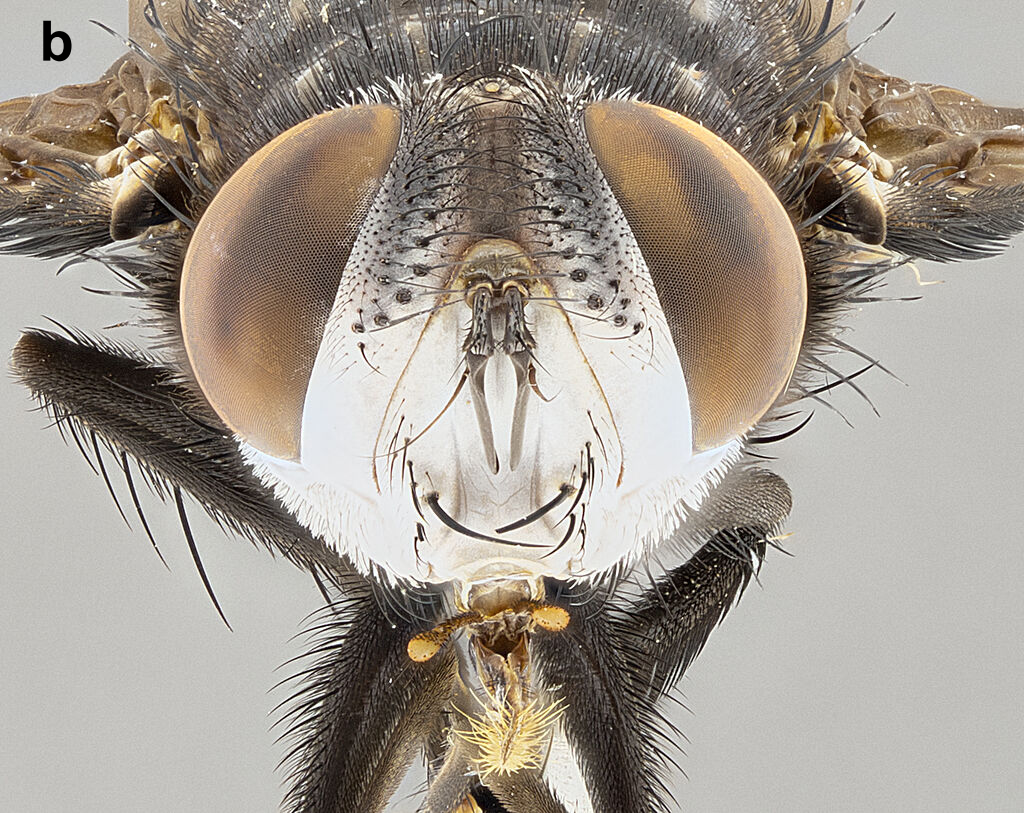
frontal view

**Figure 55c.**
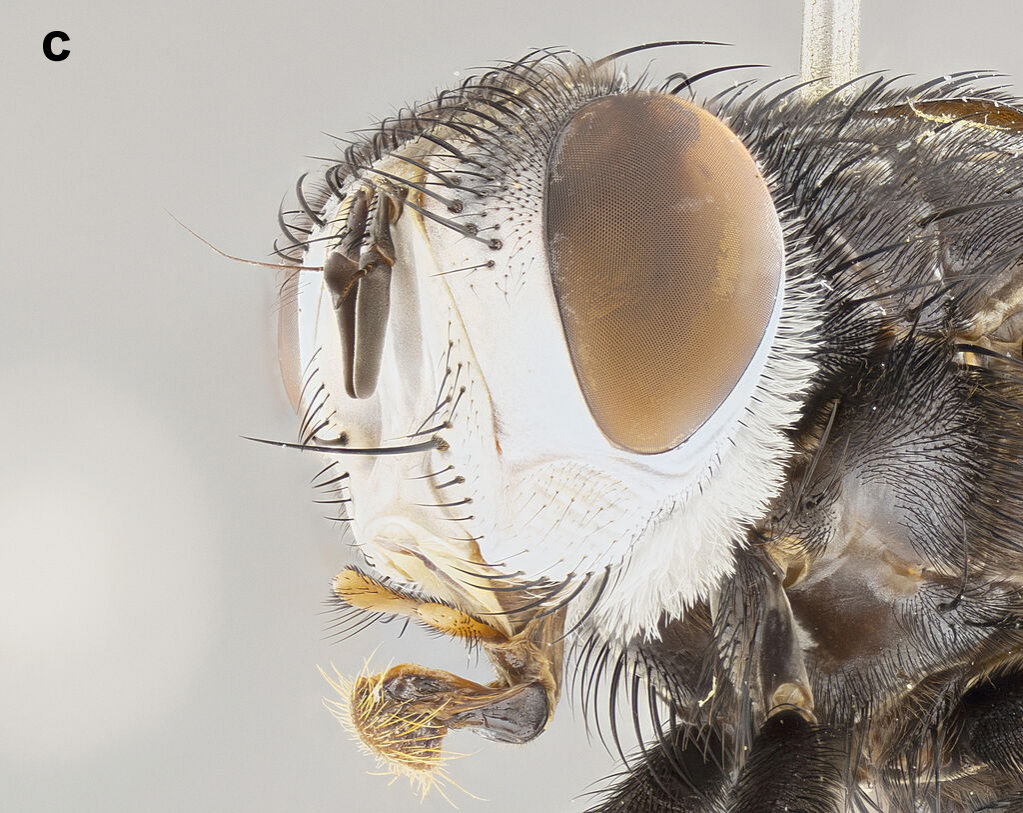
three quarters view

**Figure 55d.**
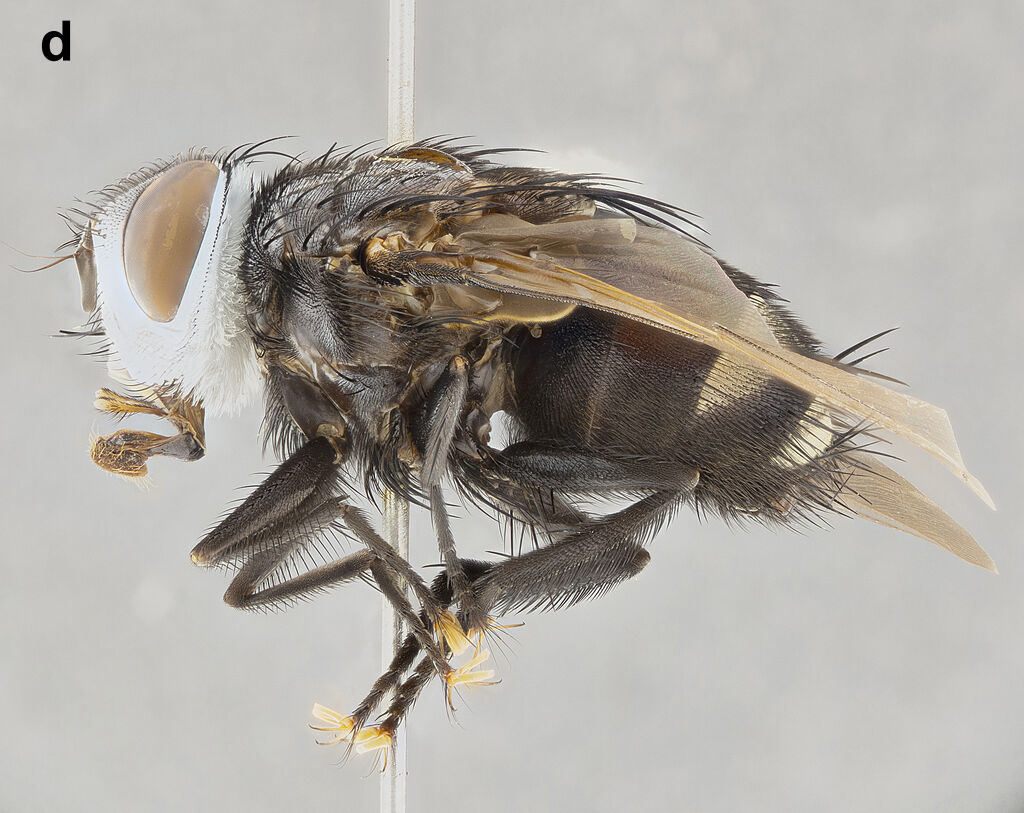
lateral view

**Figure 56a.**
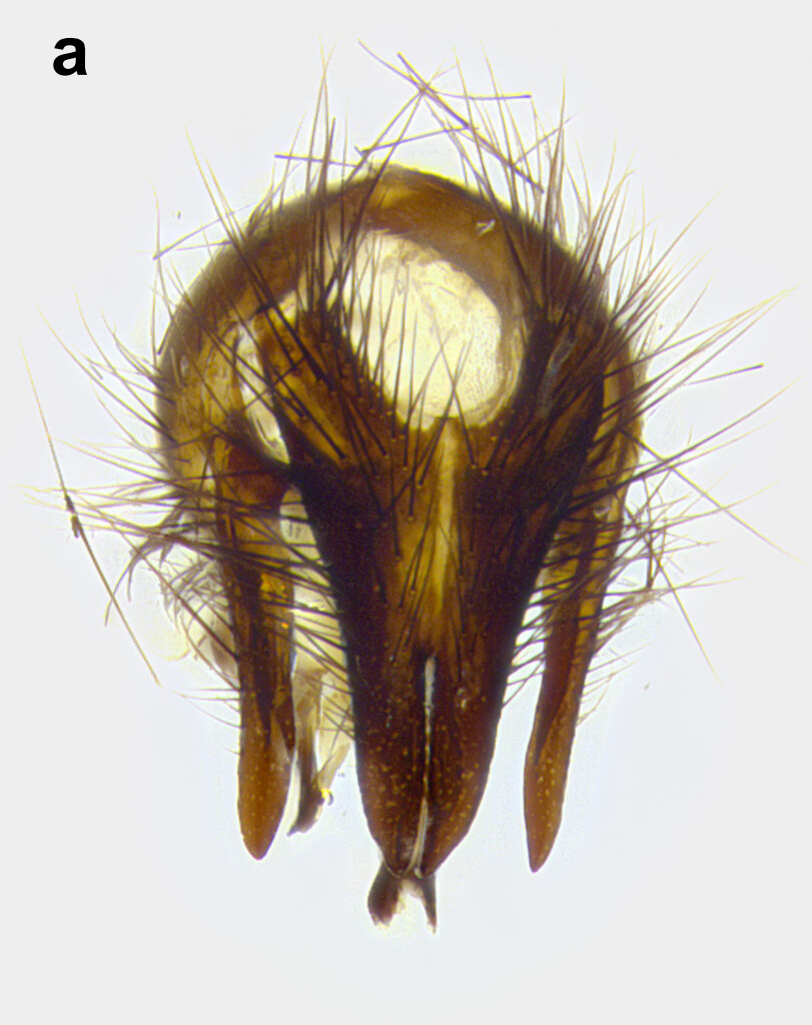
caudal view

**Figure 56b.**
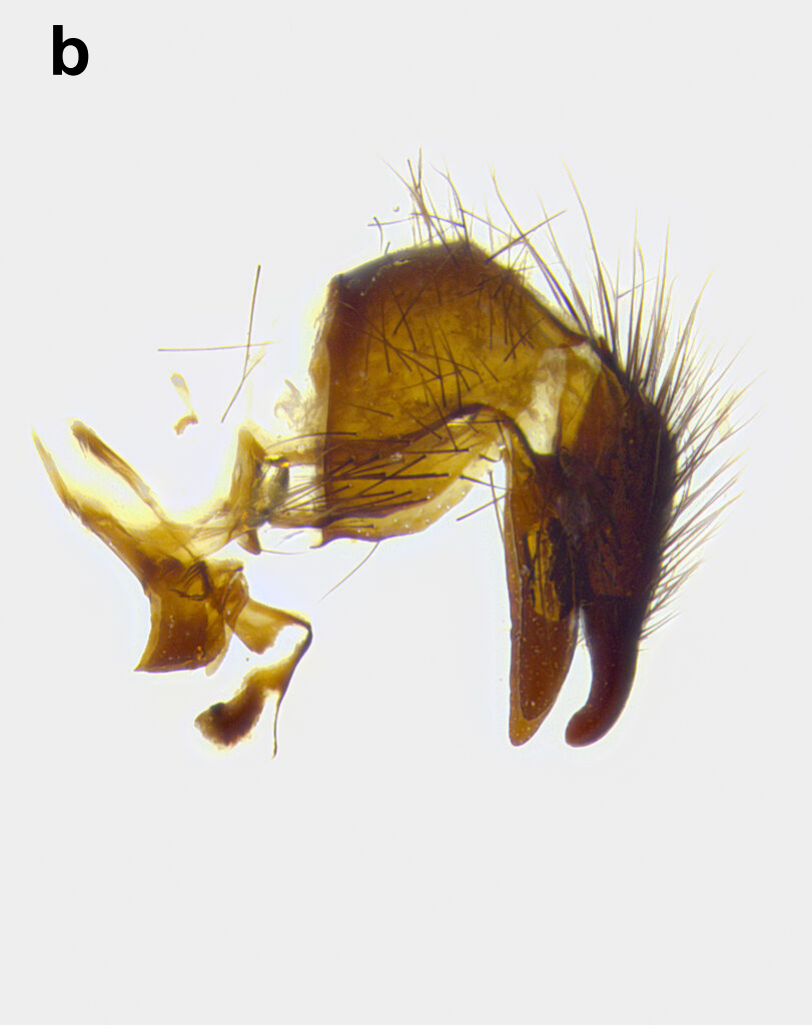
lateral view

**Figure 56c.**
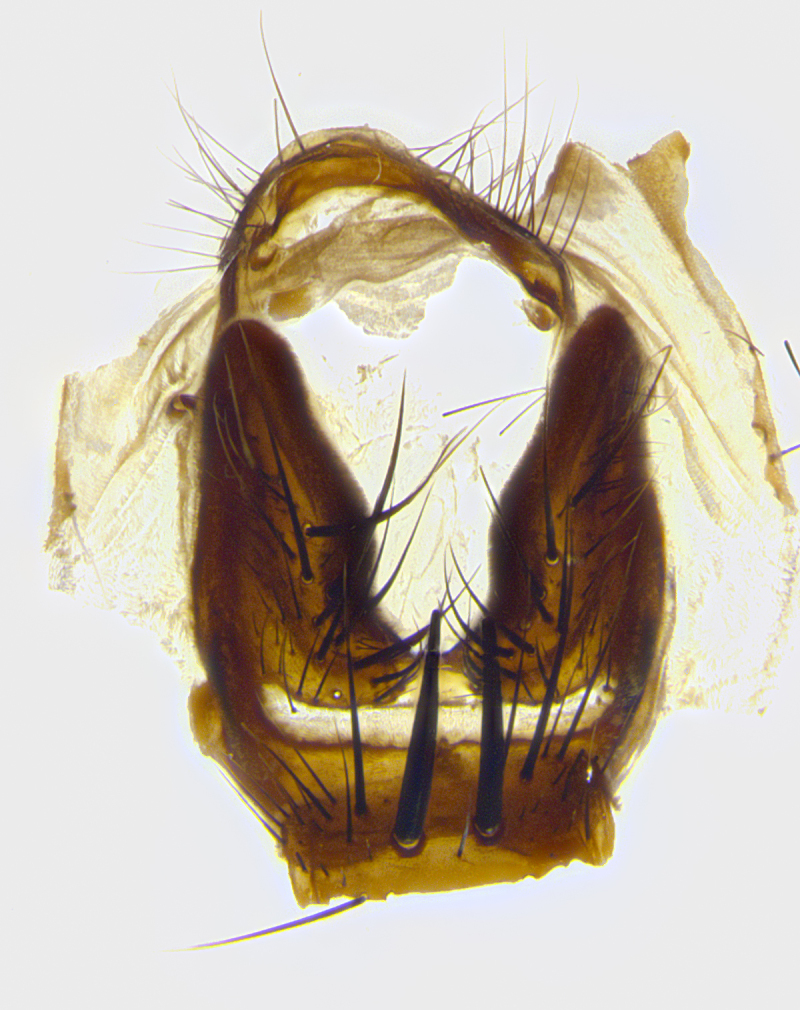
sternite 5, ventral view

**Figure 57a.**
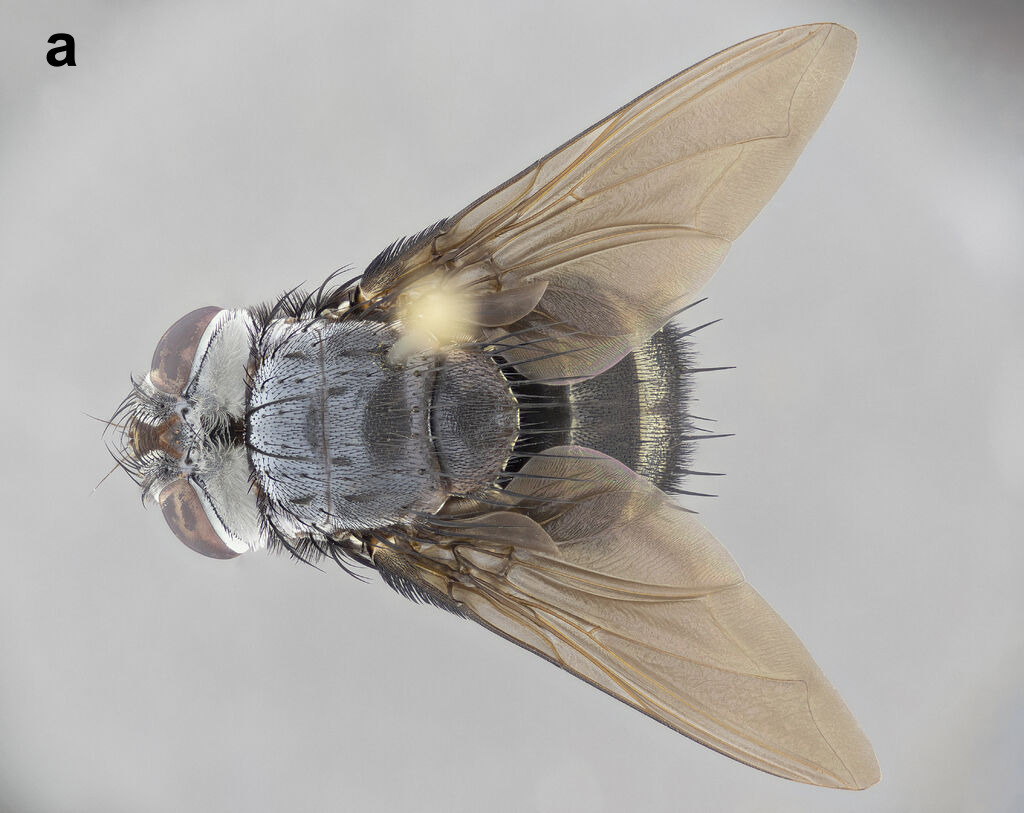
dorsal view

**Figure 57b.**
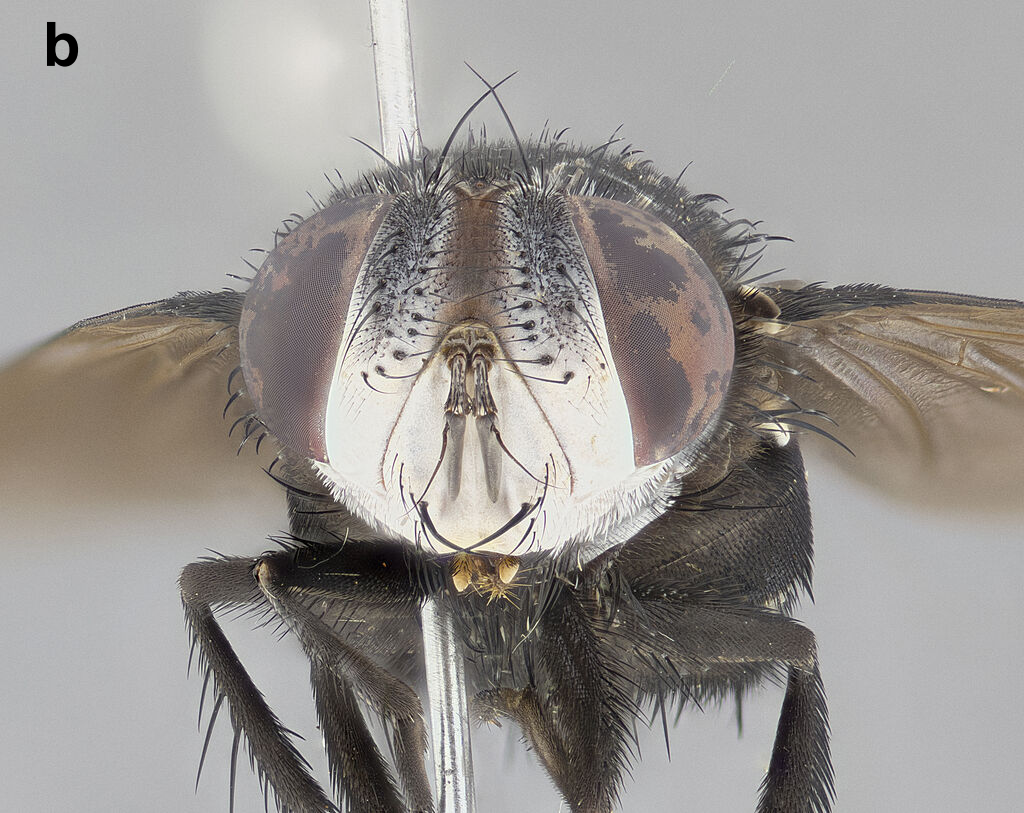
frontal view

**Figure 57c.**
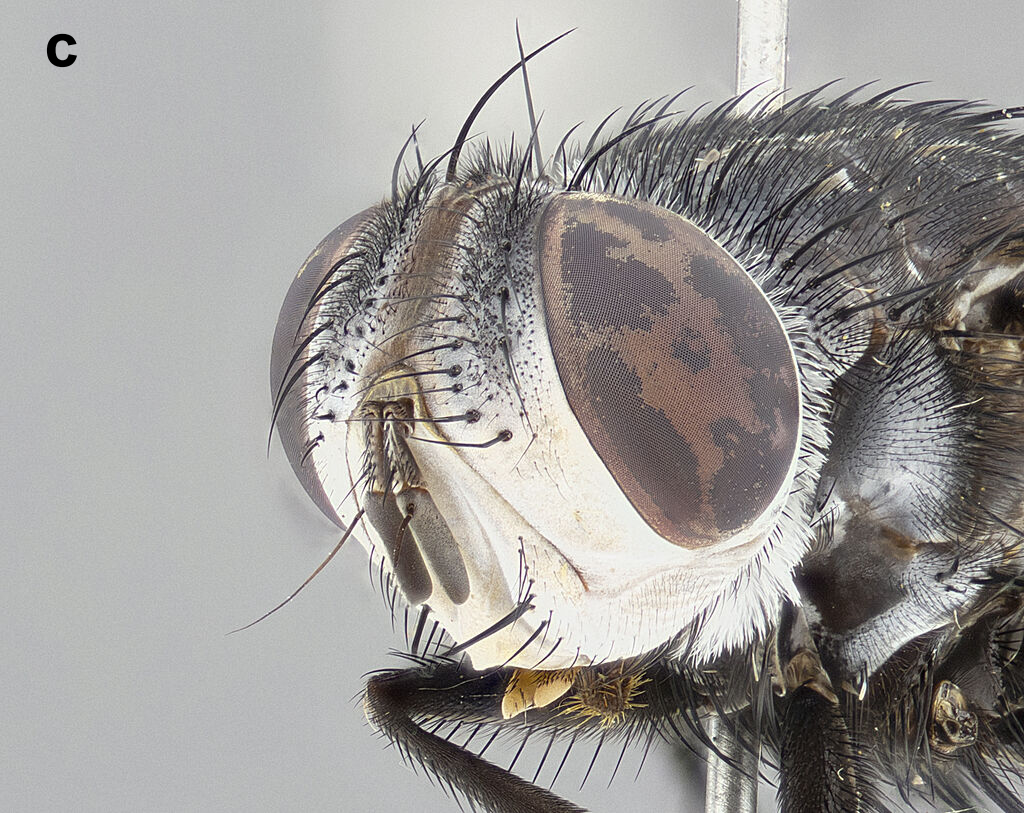
three quarters view

**Figure 57d.**
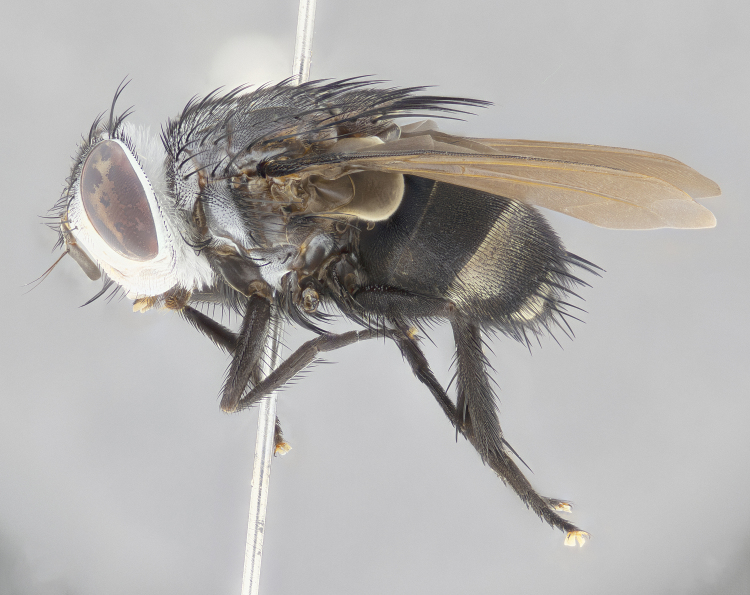
lateral view

**Figure 58a.**
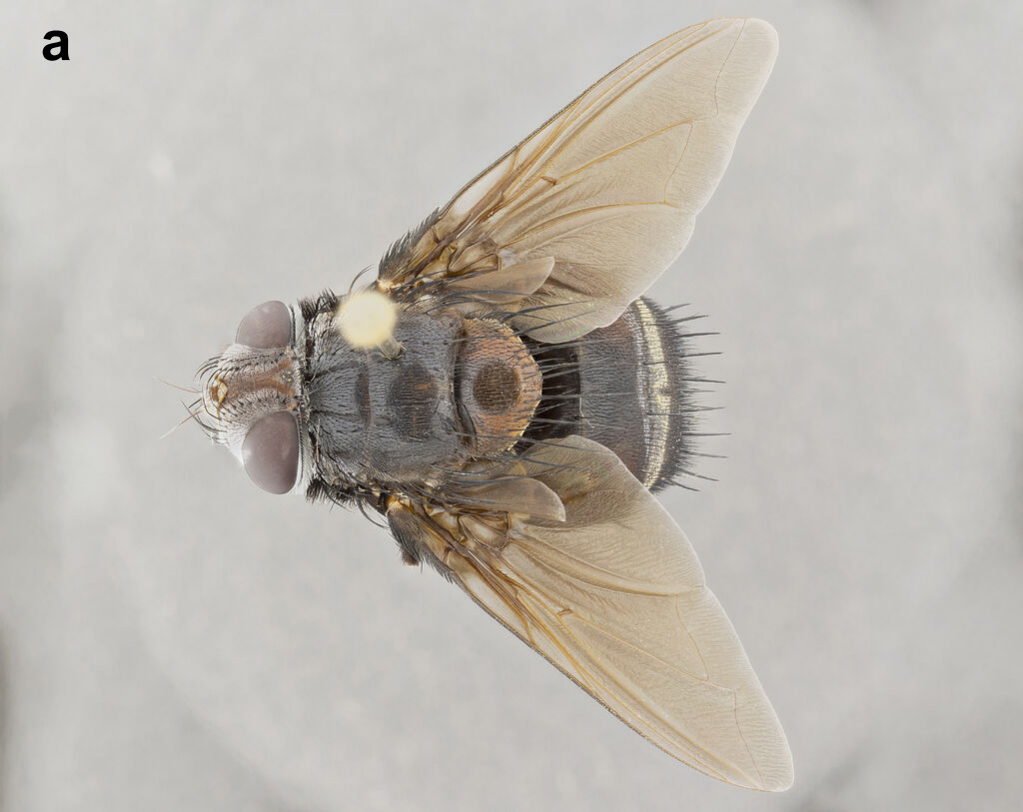
dorsal view

**Figure 58b.**
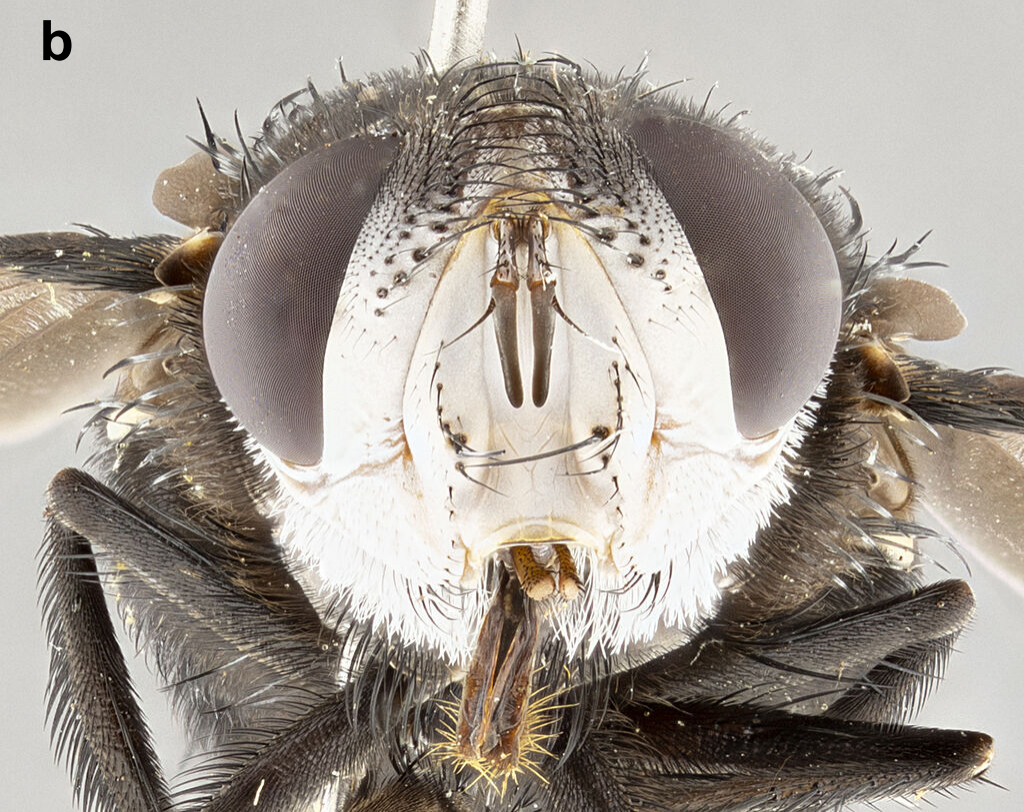
frontal view

**Figure 58c.**
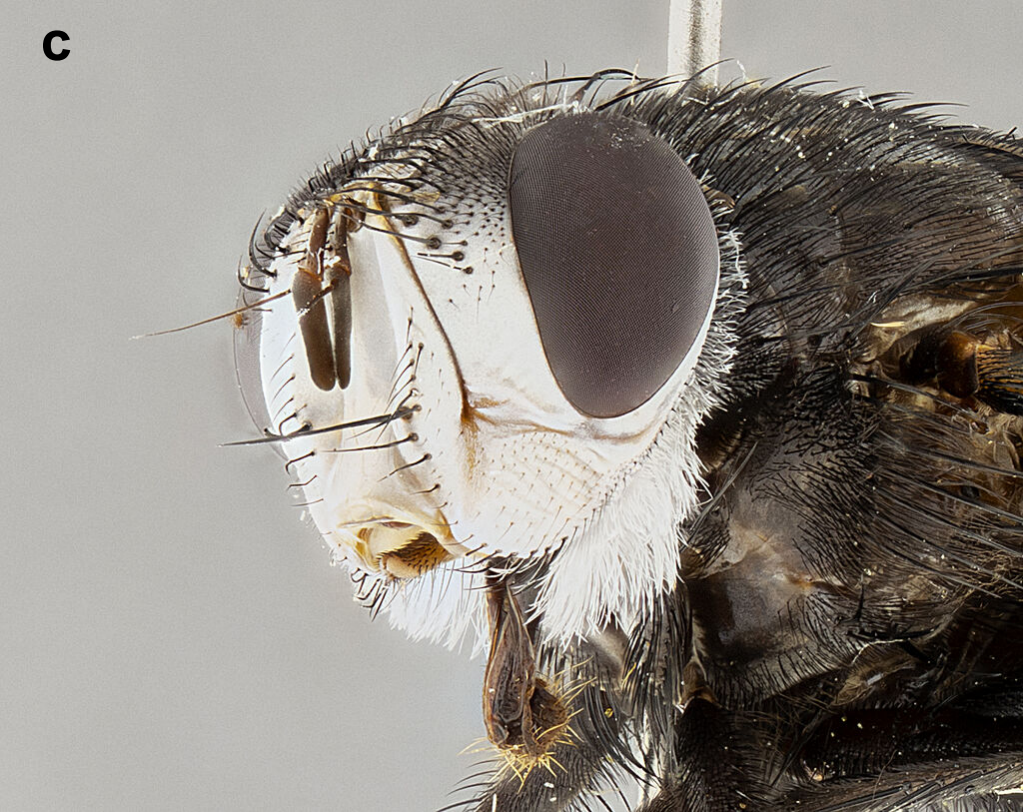
three quarters view

**Figure 58d.**
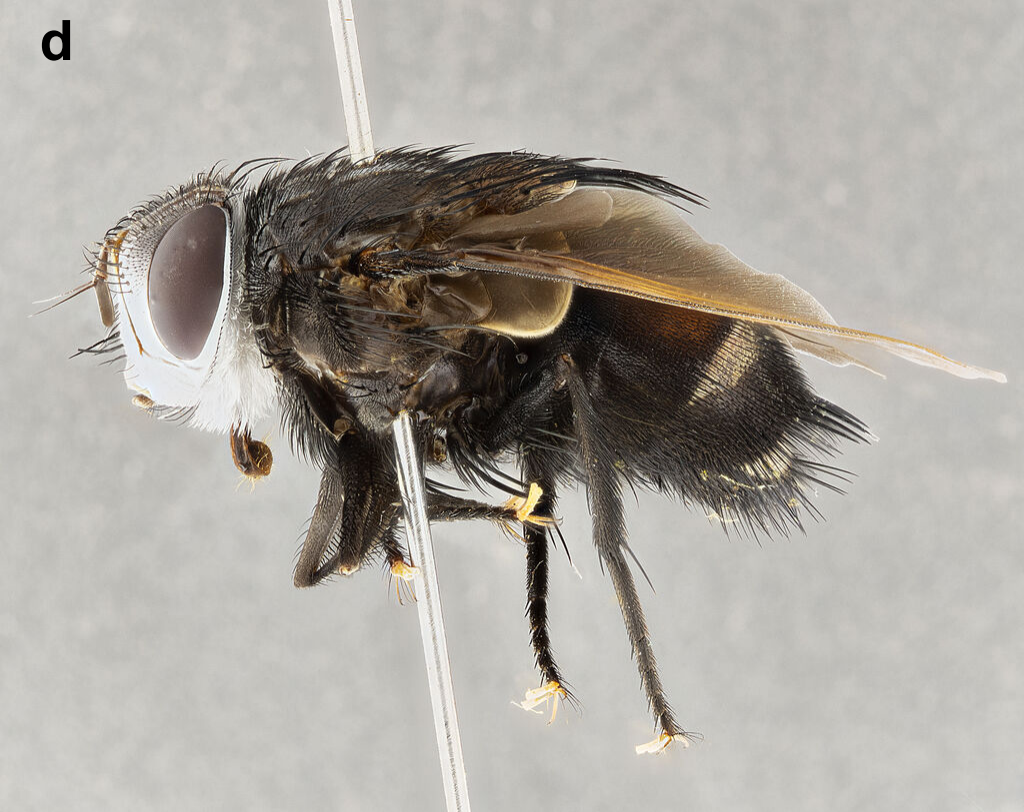
lateral view

**Figure 59a.**
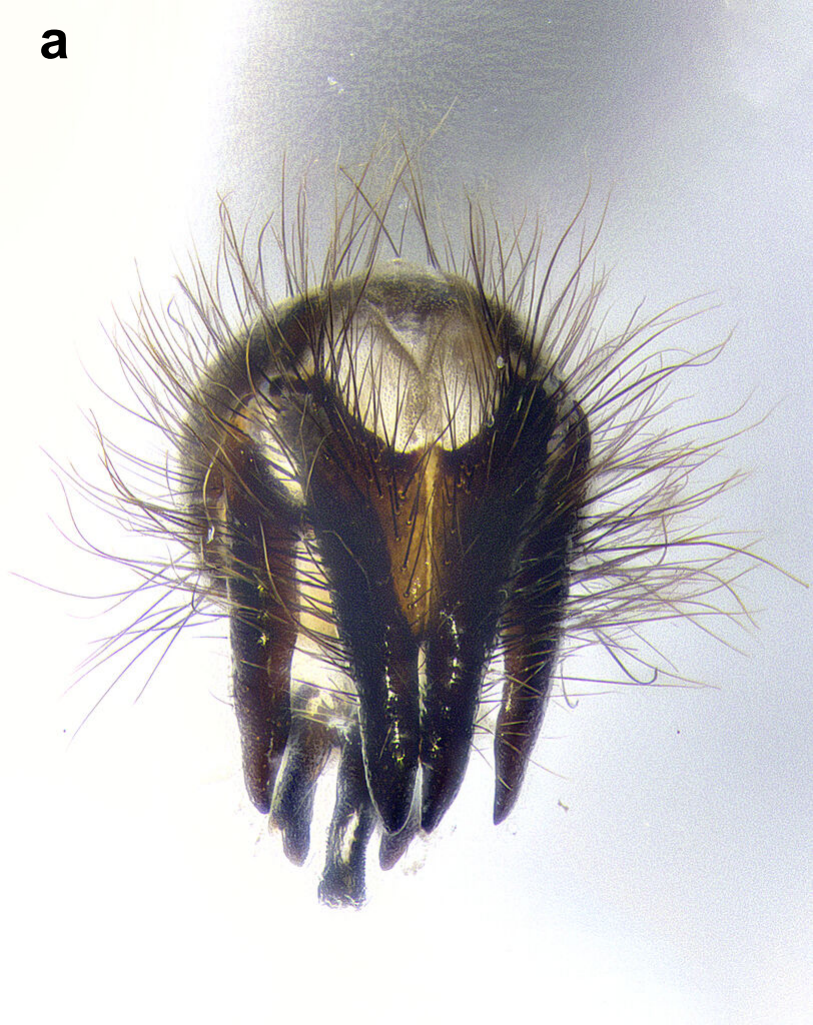
caudal view

**Figure 59b.**
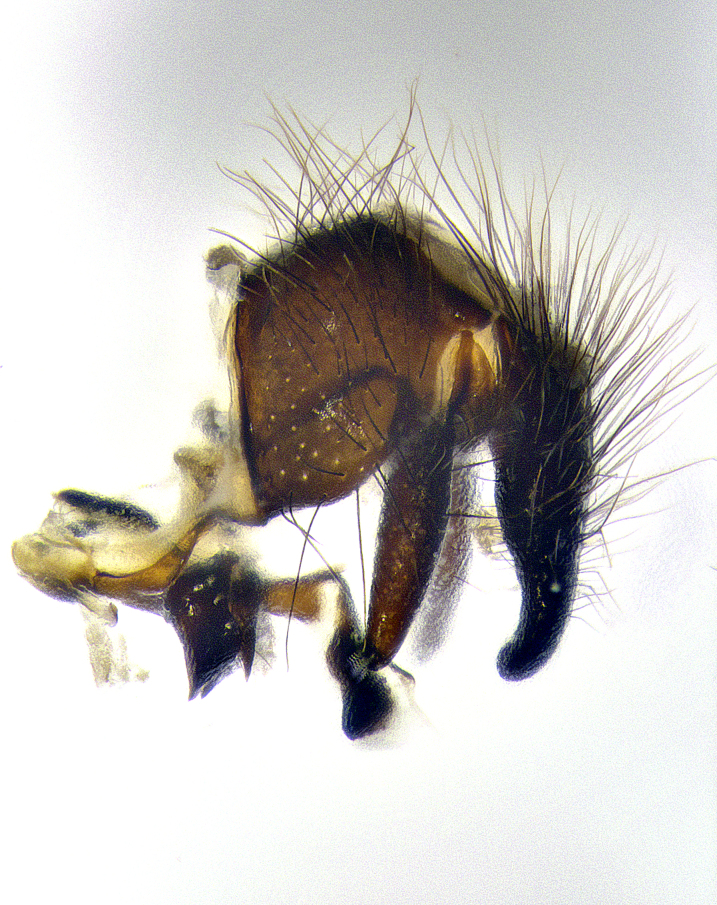
lateral view

**Figure 59c.**
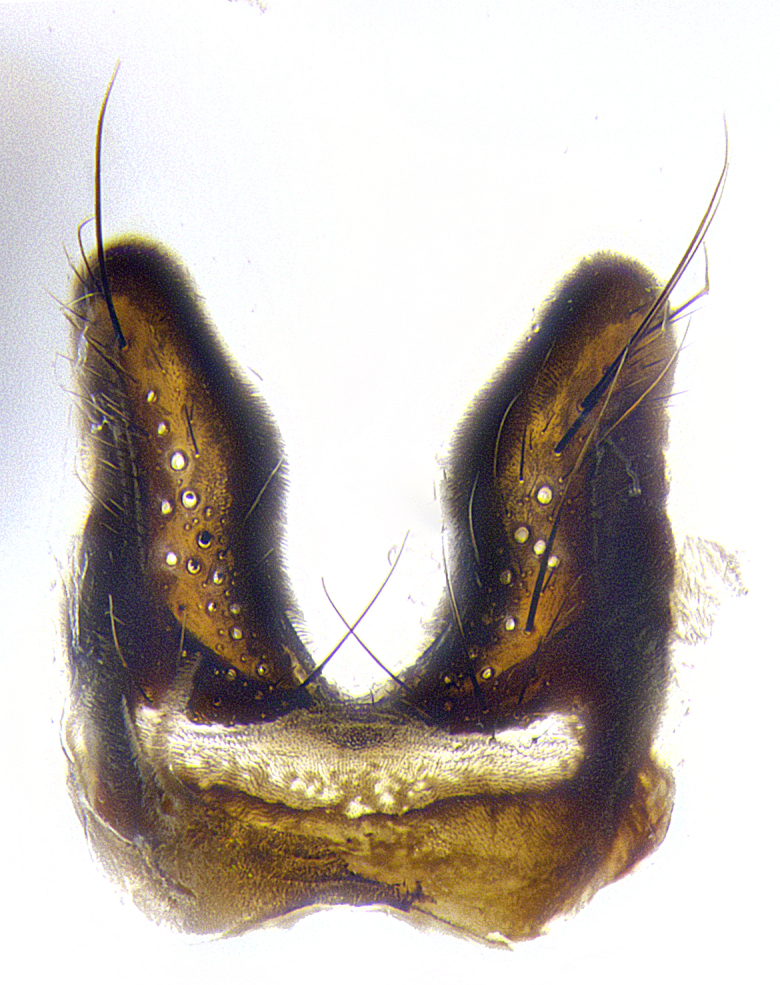
sternite 5, ventral view

**Figure 60a.**
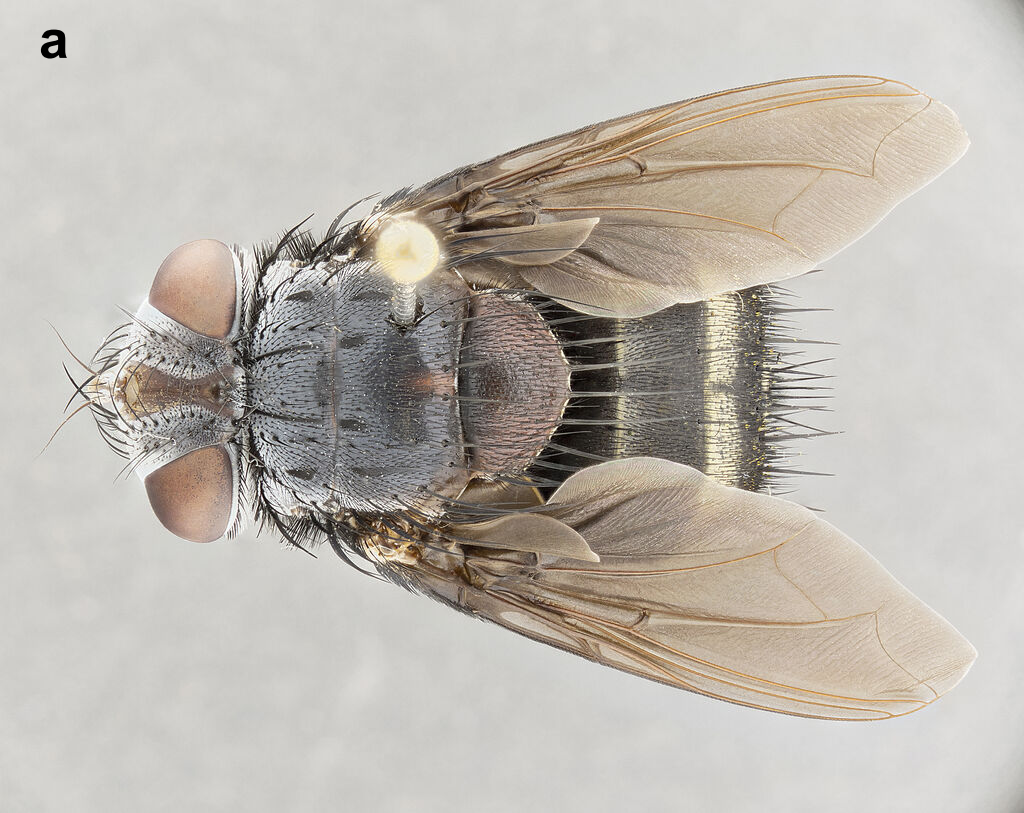
dorsal view

**Figure 60b.**
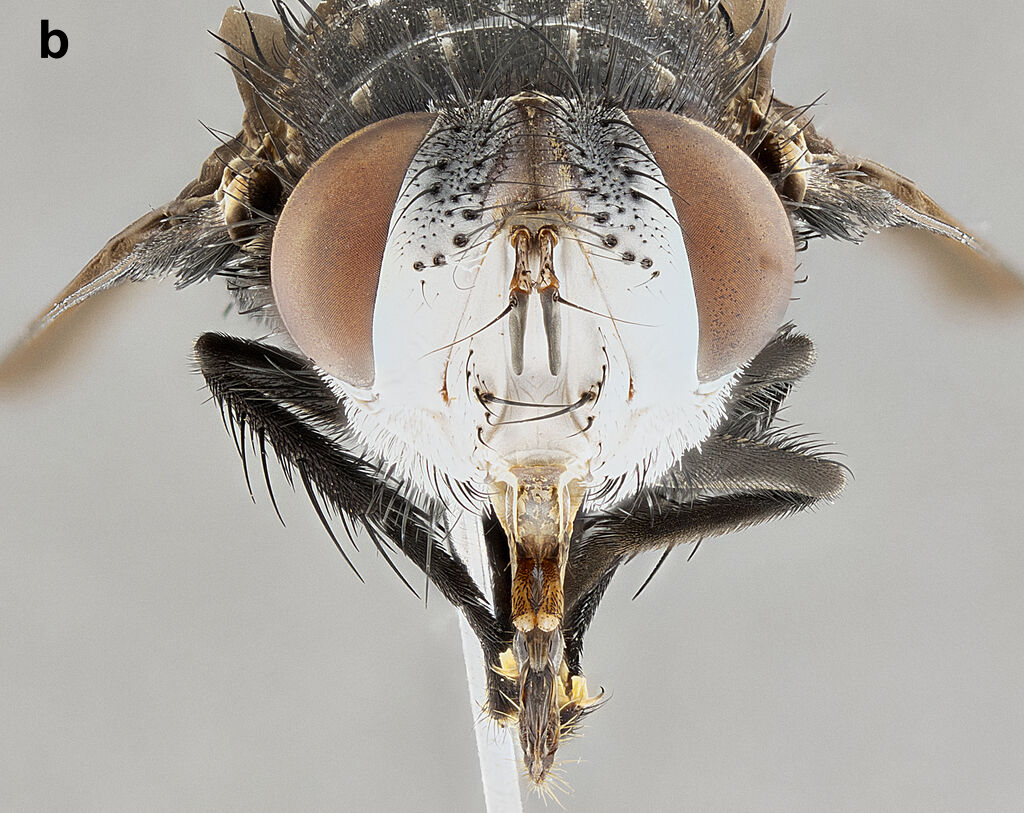
frontal view

**Figure 60c.**
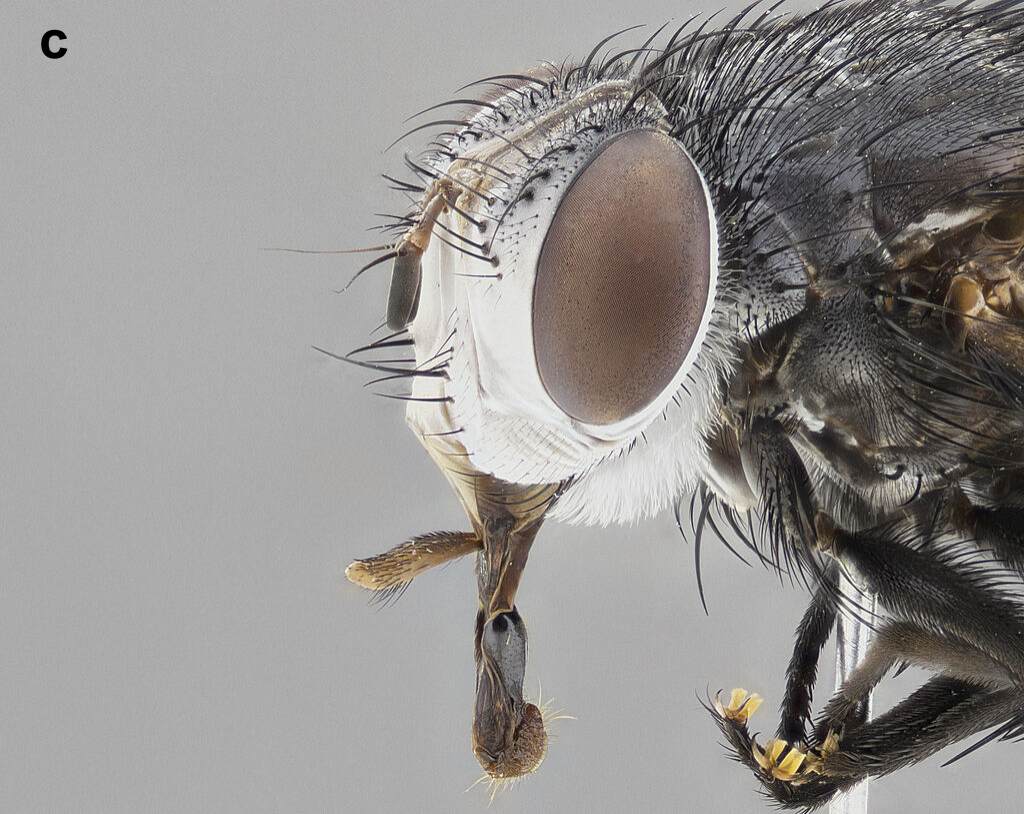
three quarters view

**Figure 60d.**
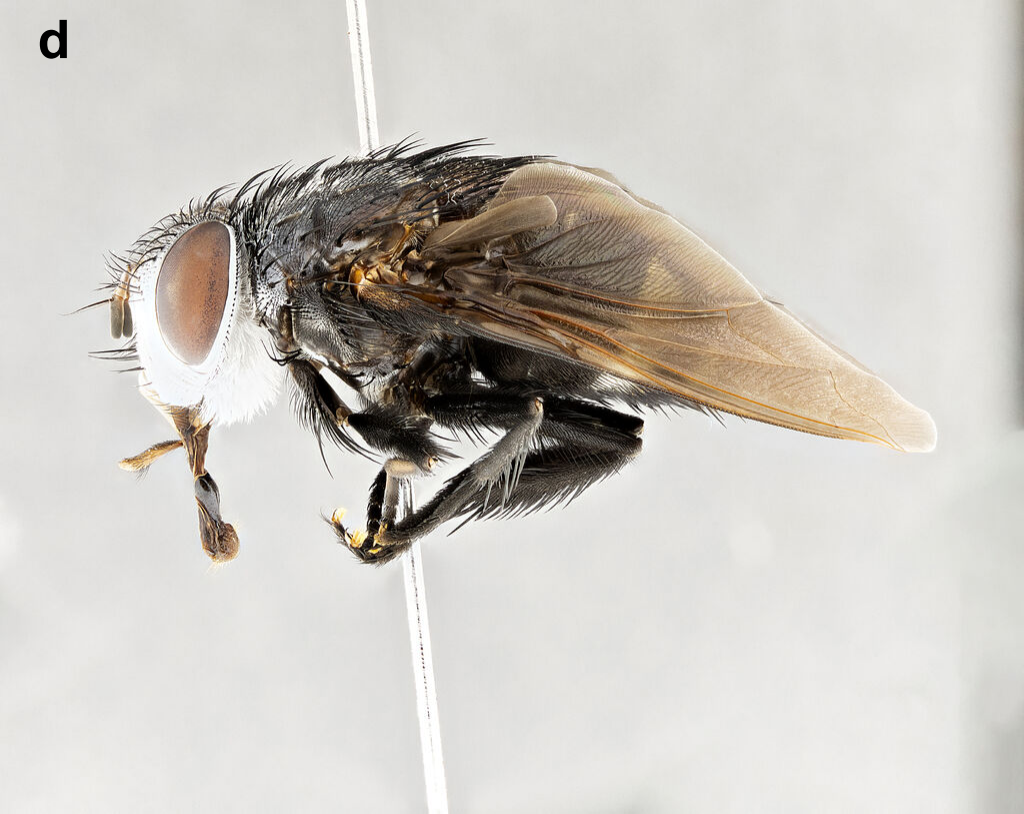
lateral view

**Figure 61a.**
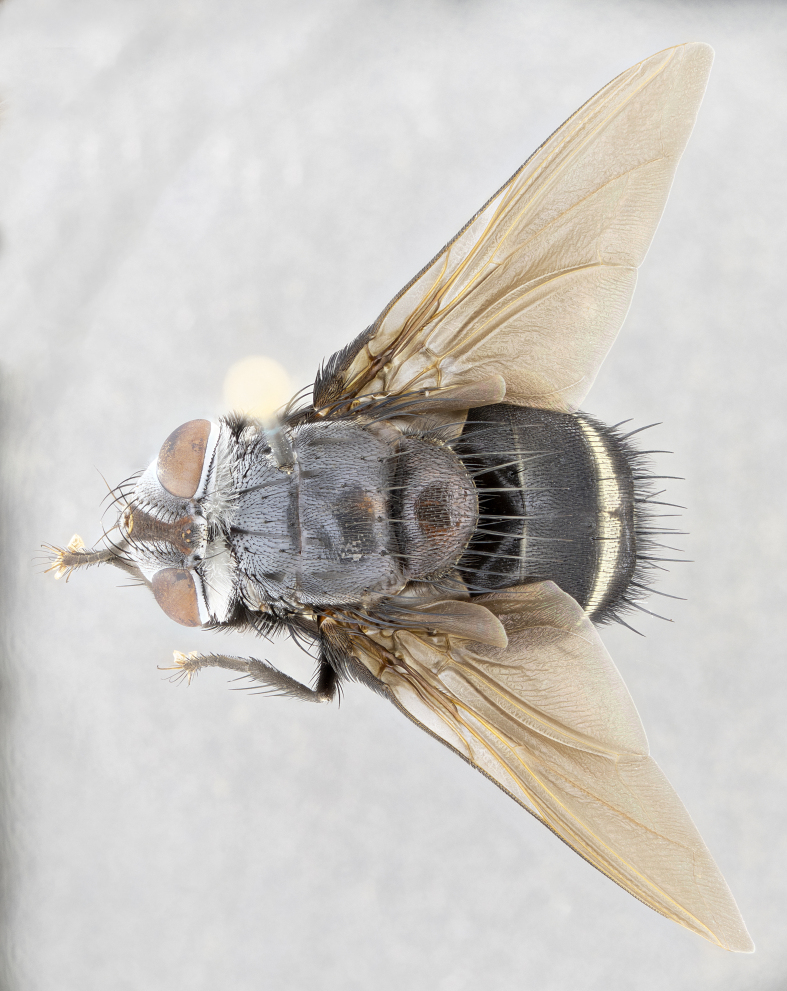
dorsal view

**Figure 61b.**
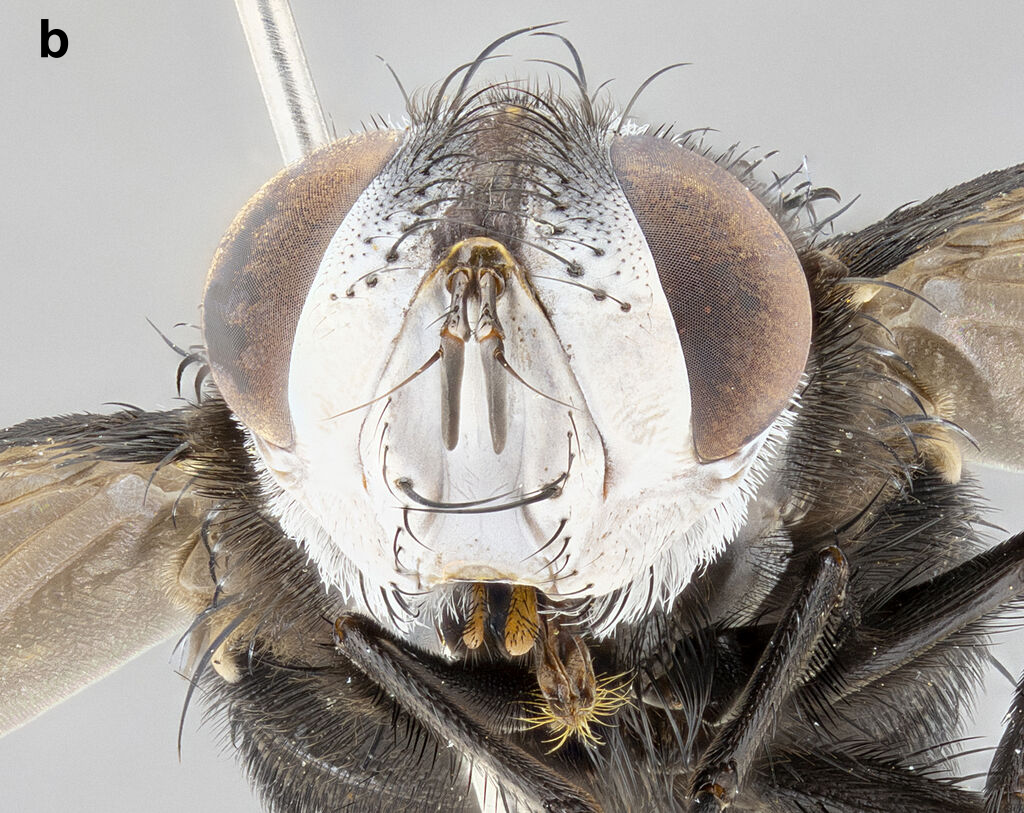
frontal view

**Figure 61c.**
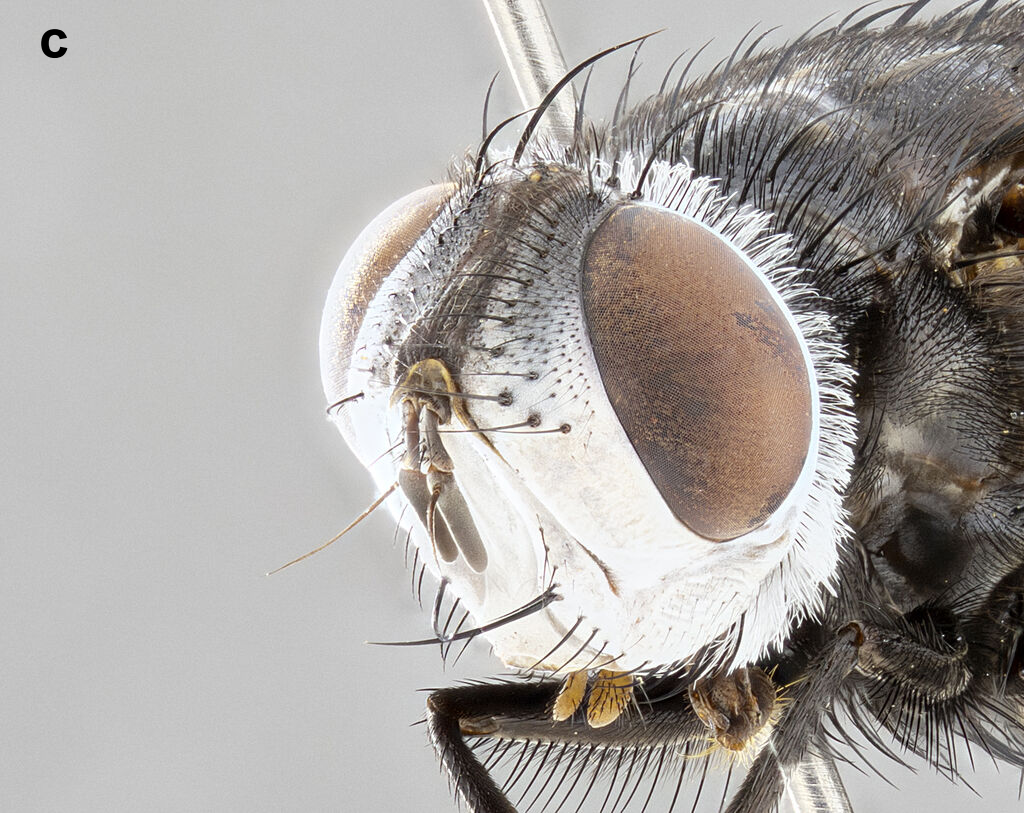
three quarters view

**Figure 61d.**
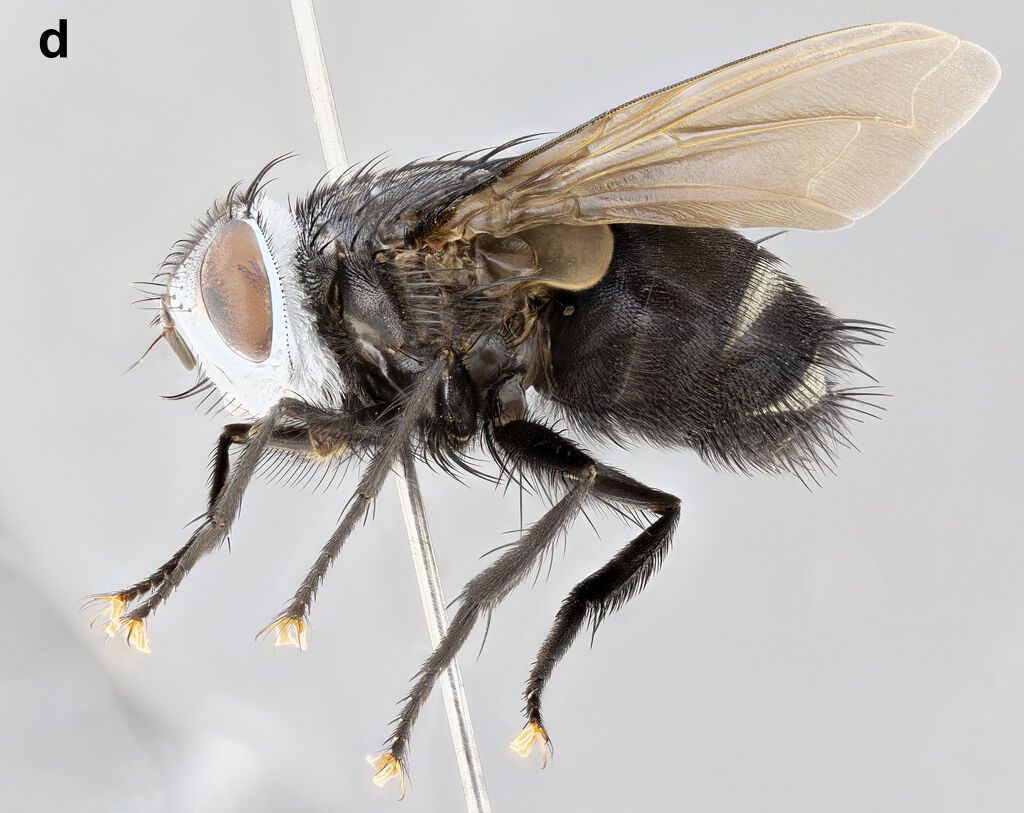
lateral view

**Figure 62a.**
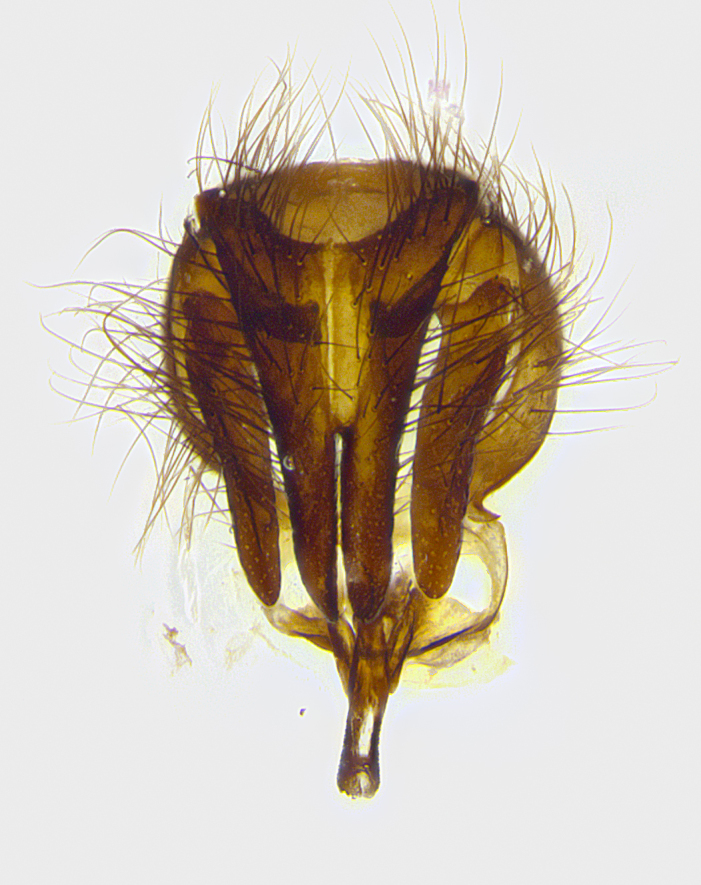
caudal view

**Figure 62b.**
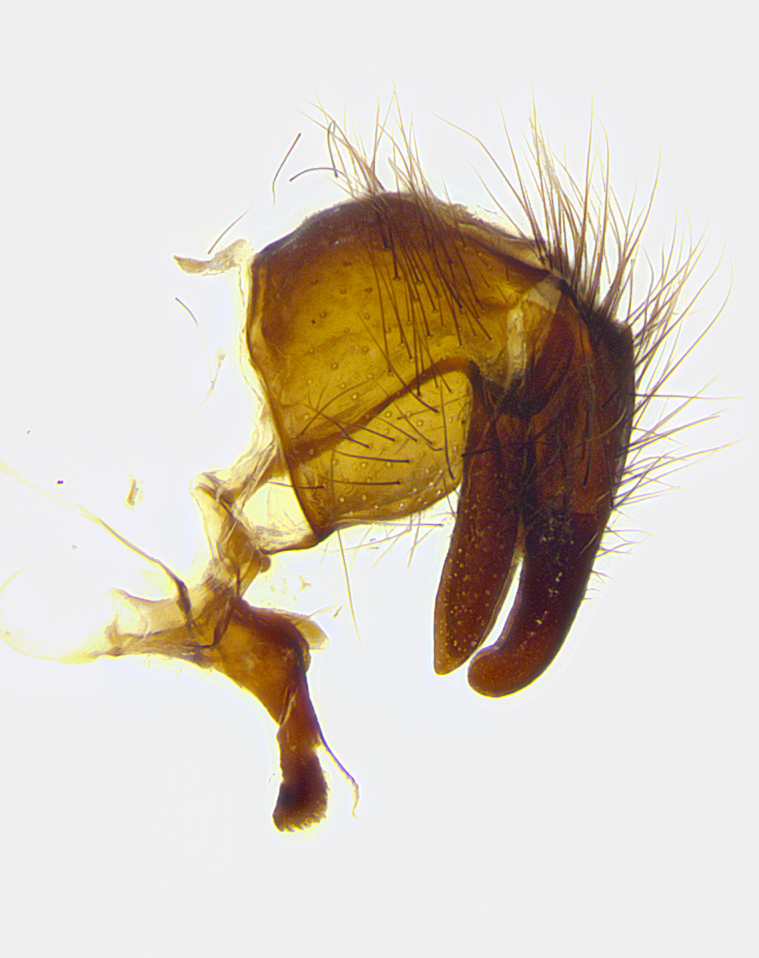
lateral view

**Figure 62c.**
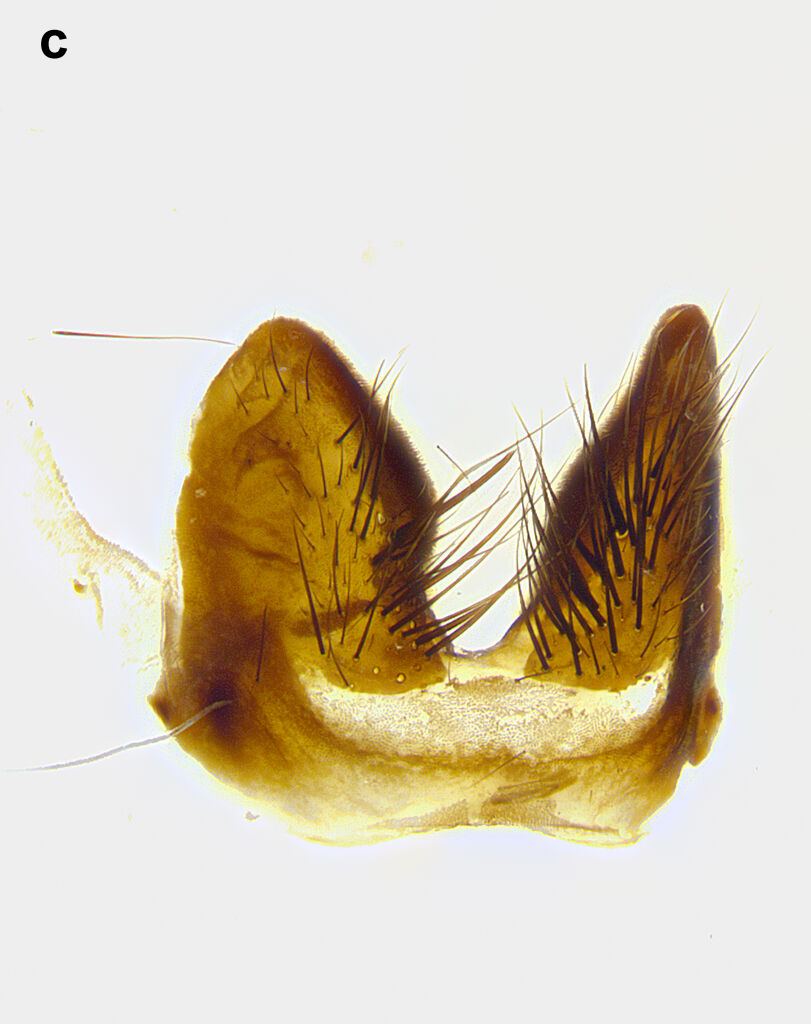
sternite 5, ventral view

**Figure 63a.**
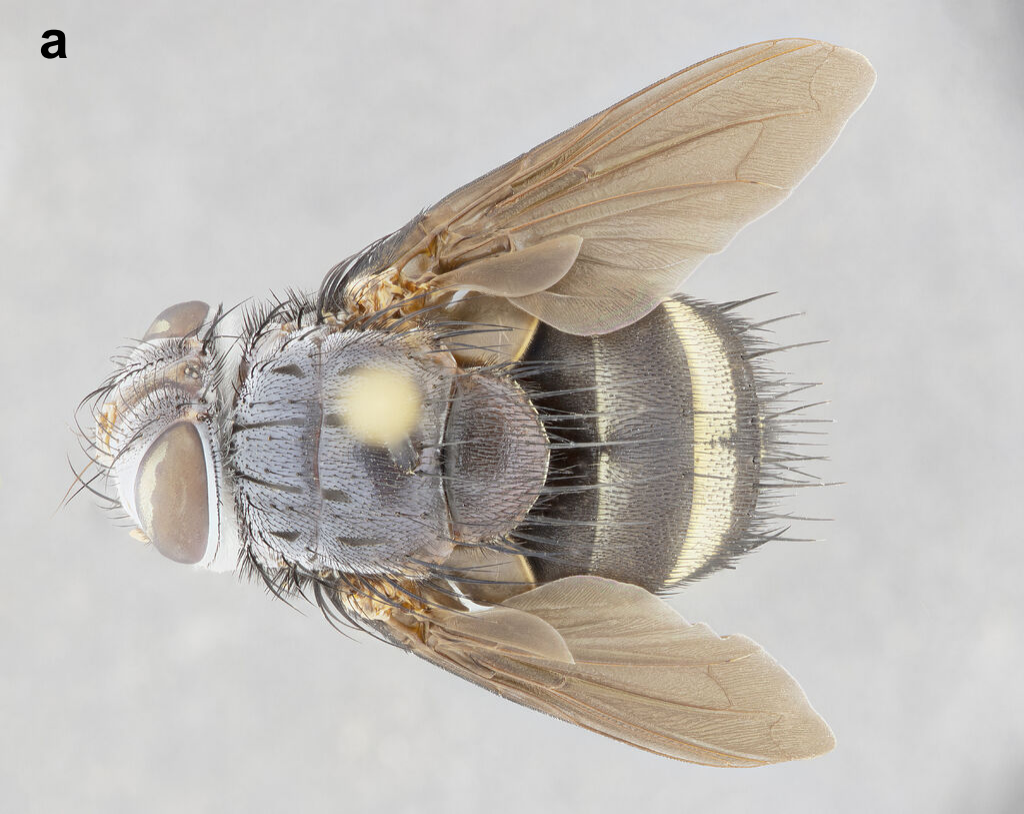
dorsal view

**Figure 63b.**
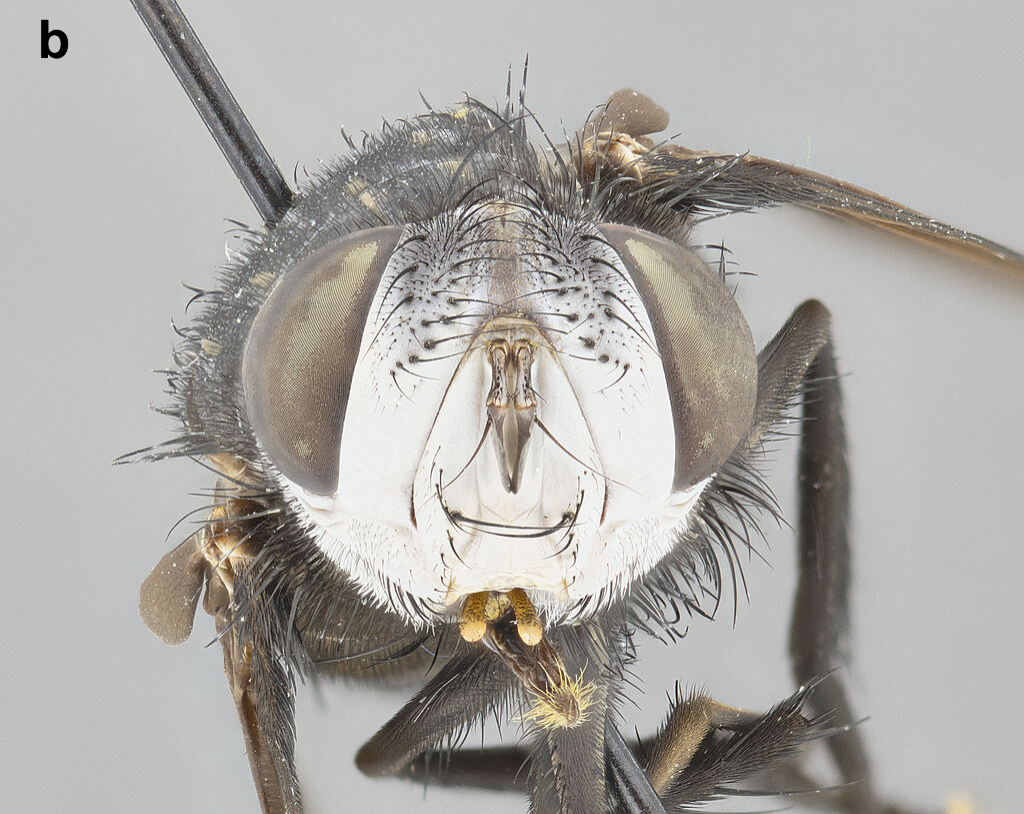
frontal view

**Figure 63c.**
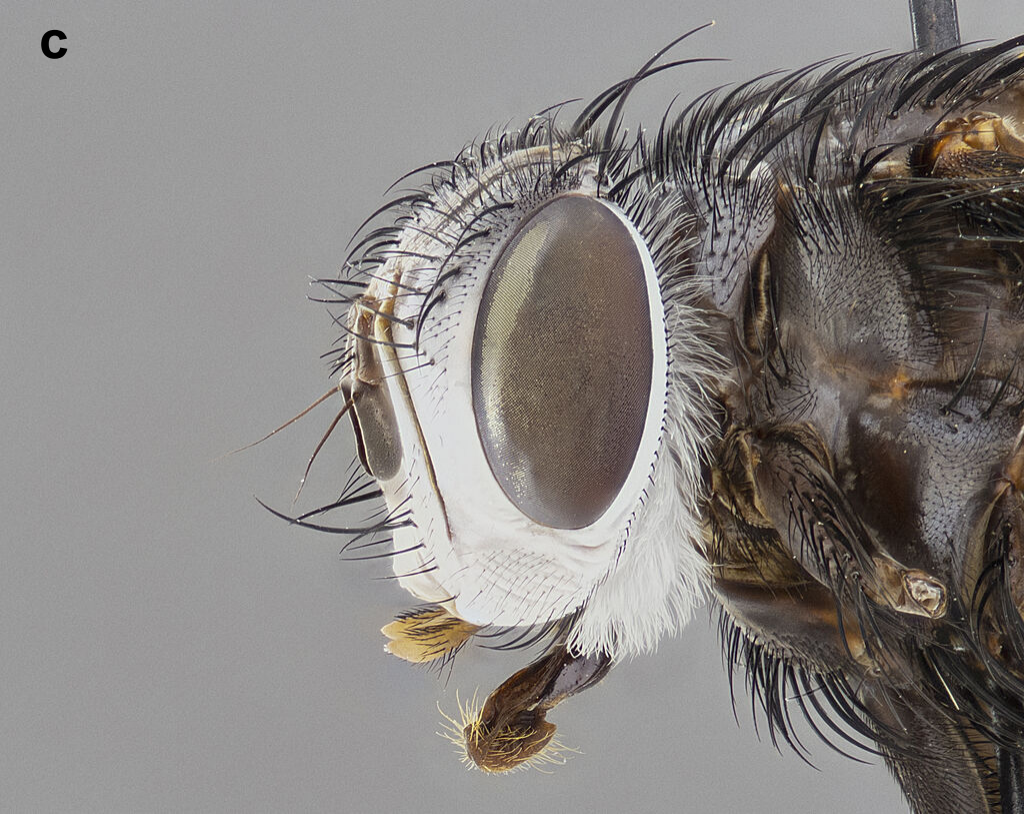
three quarters view

**Figure 63d.**
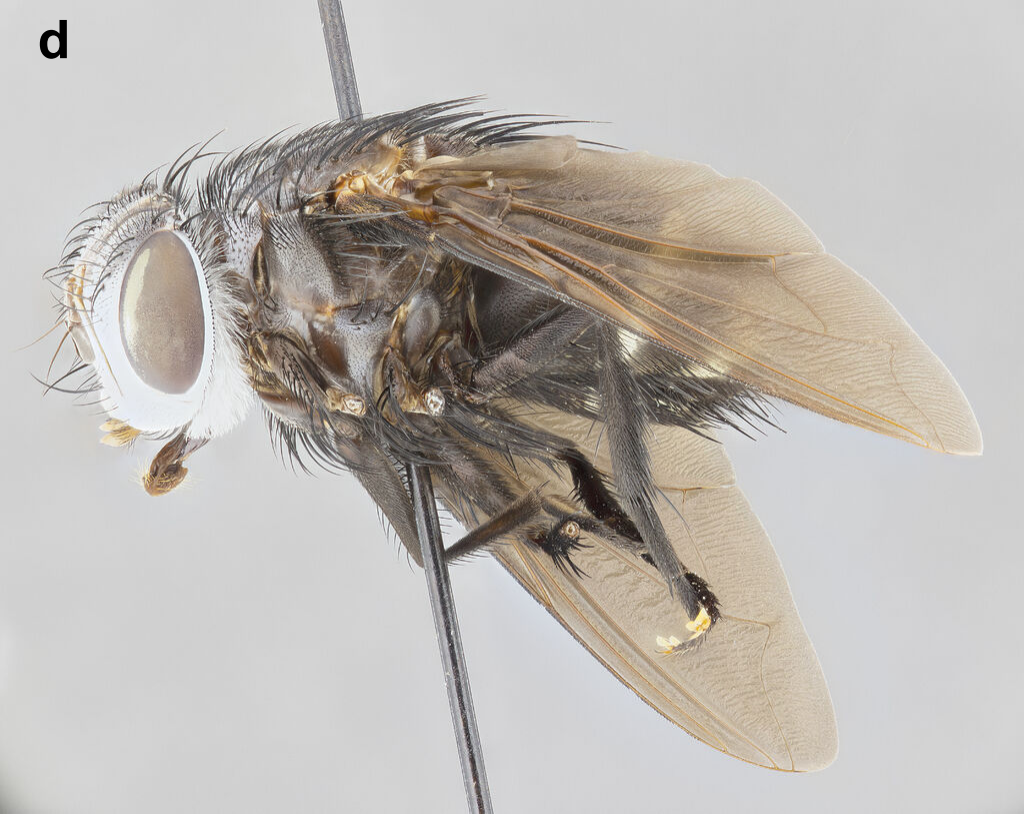
lateral view

**Figure 64a.**
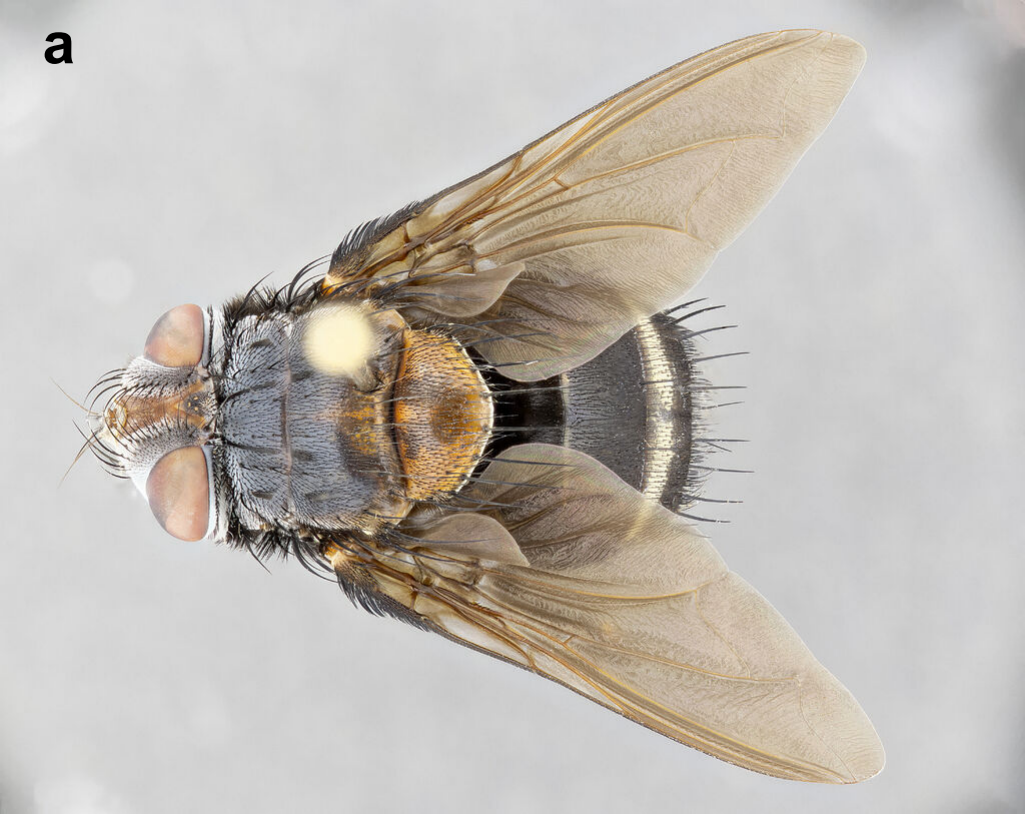
dorsal view

**Figure 64b.**
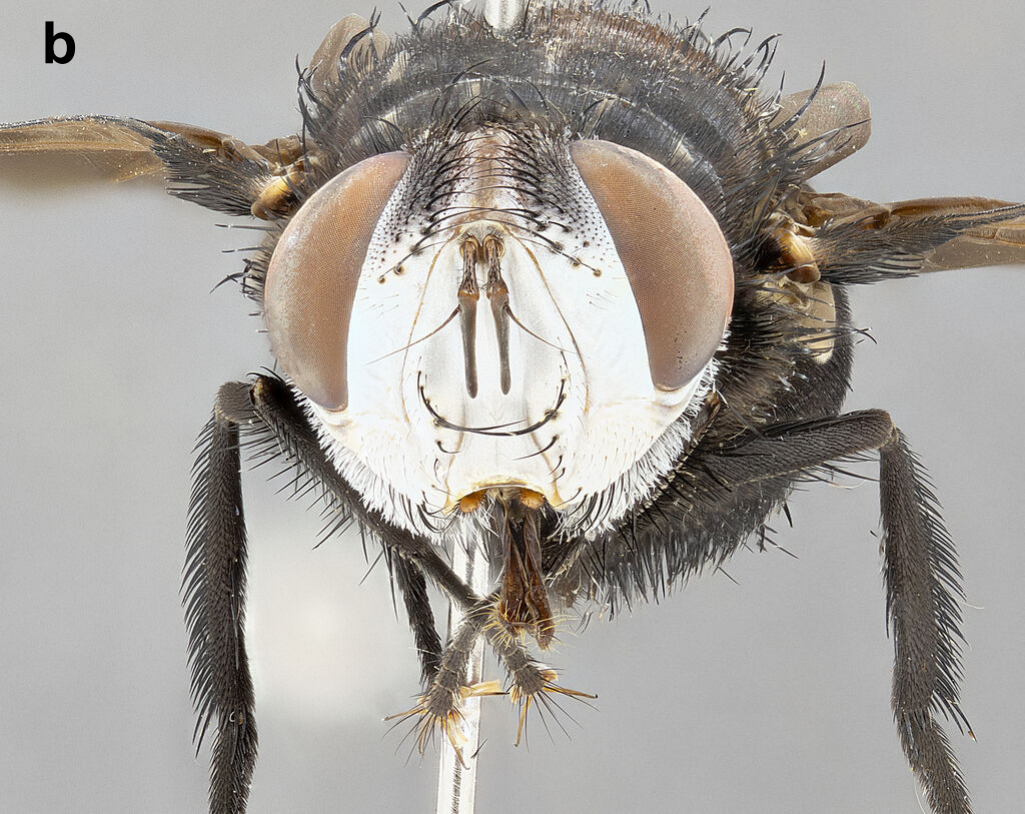
frontal view

**Figure 64c.**
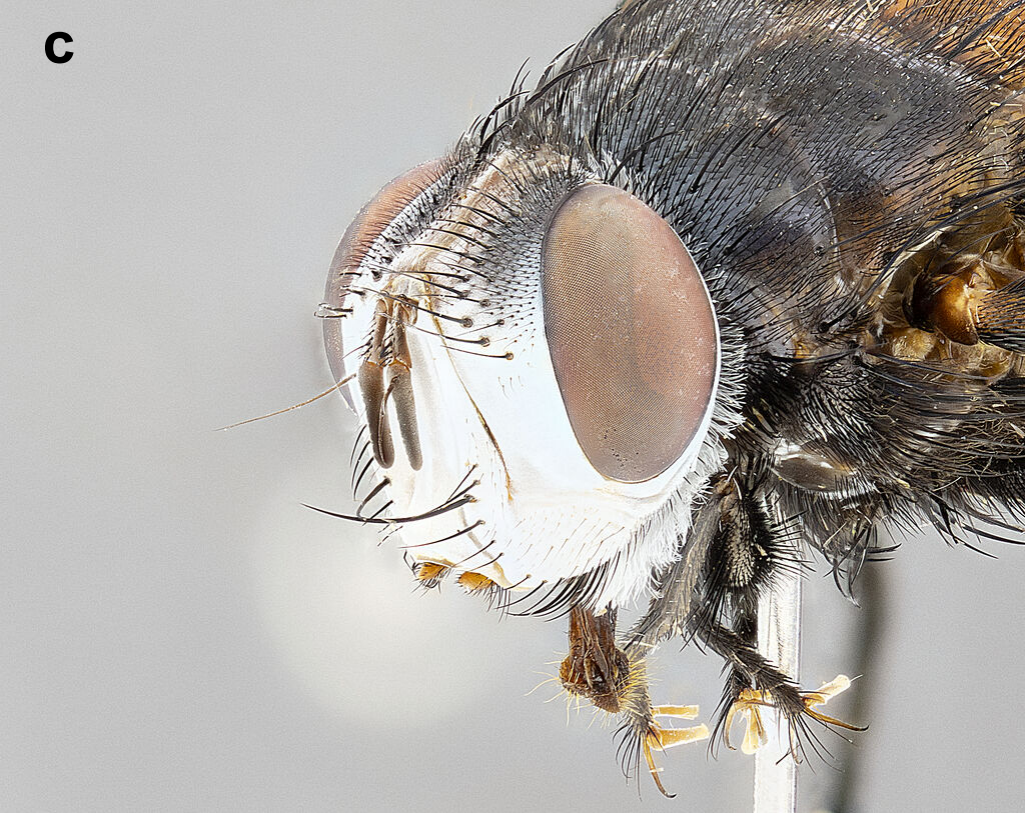
three quarters view

**Figure 64d.**
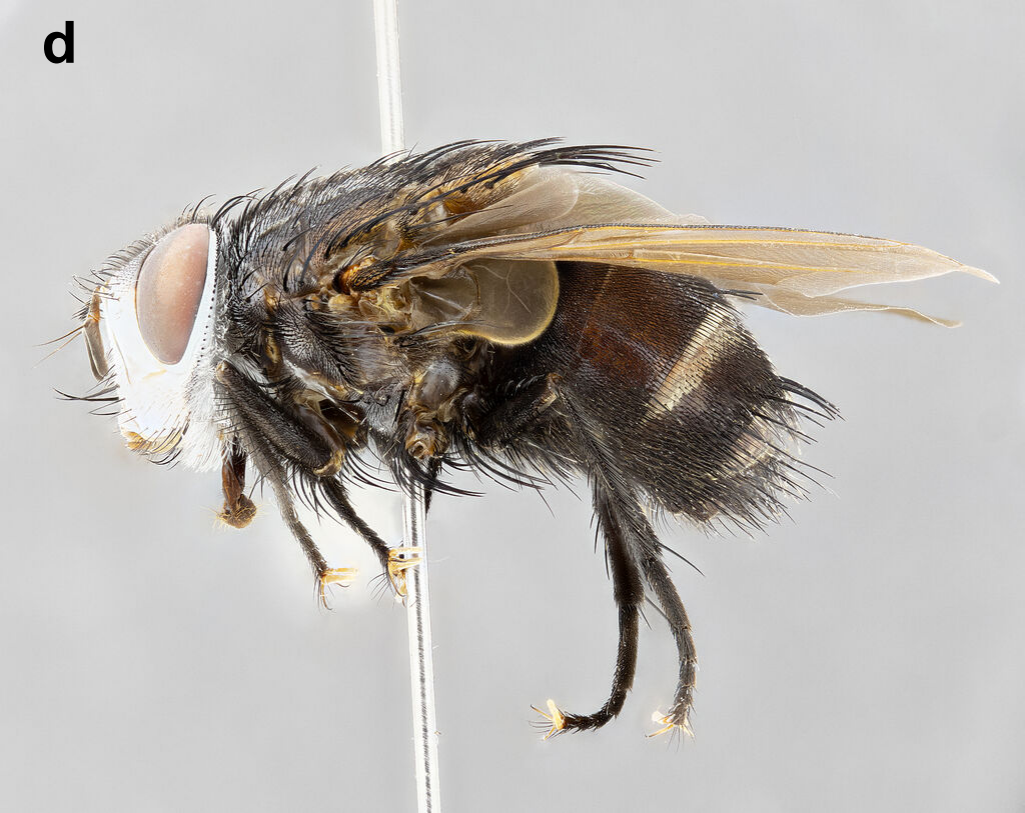
lateral view

**Figure 65a.**
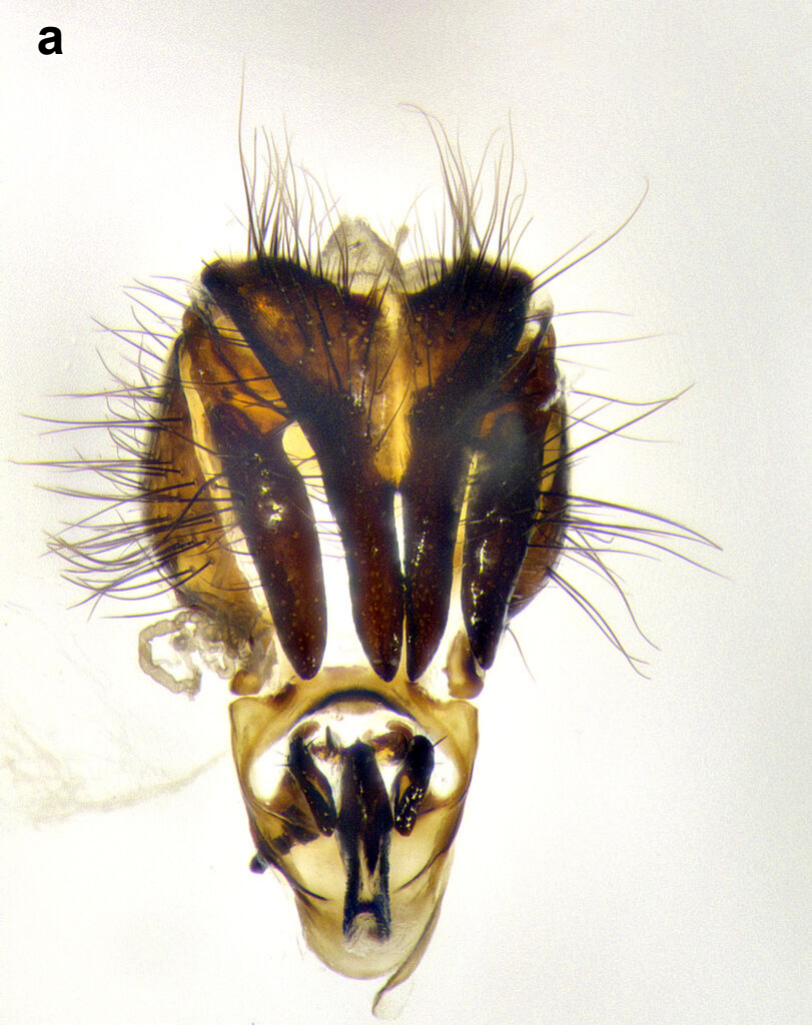
caudal view

**Figure 65b.**
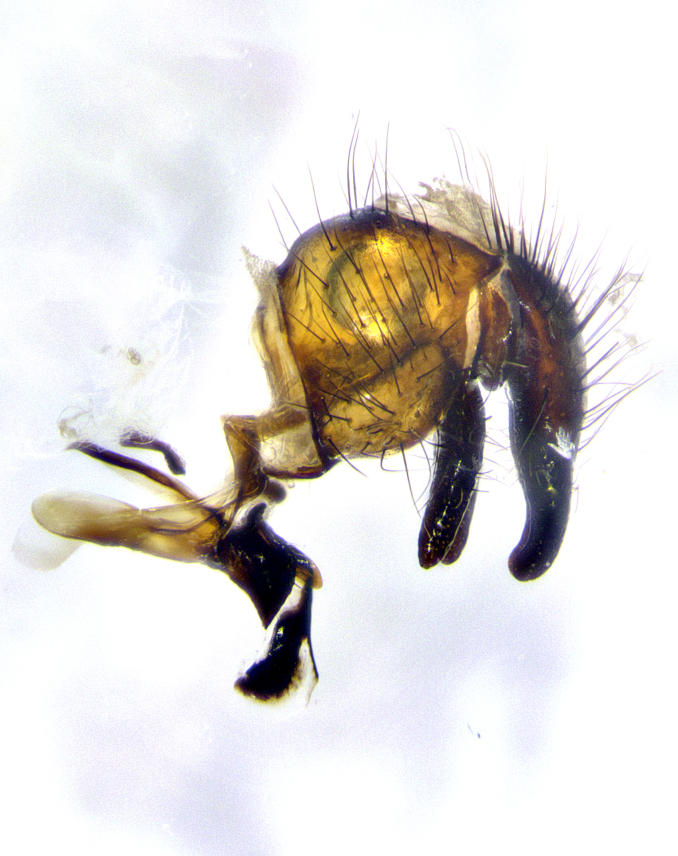
lateral view

**Figure 65c.**
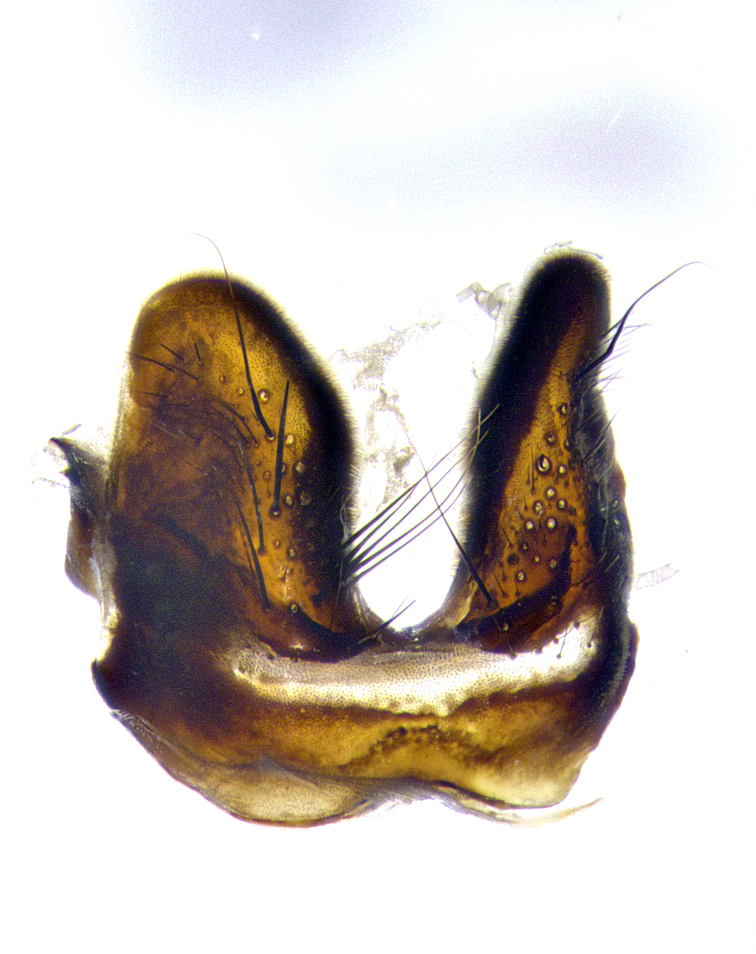
sternite 5, ventral view

**Figure 66a.**
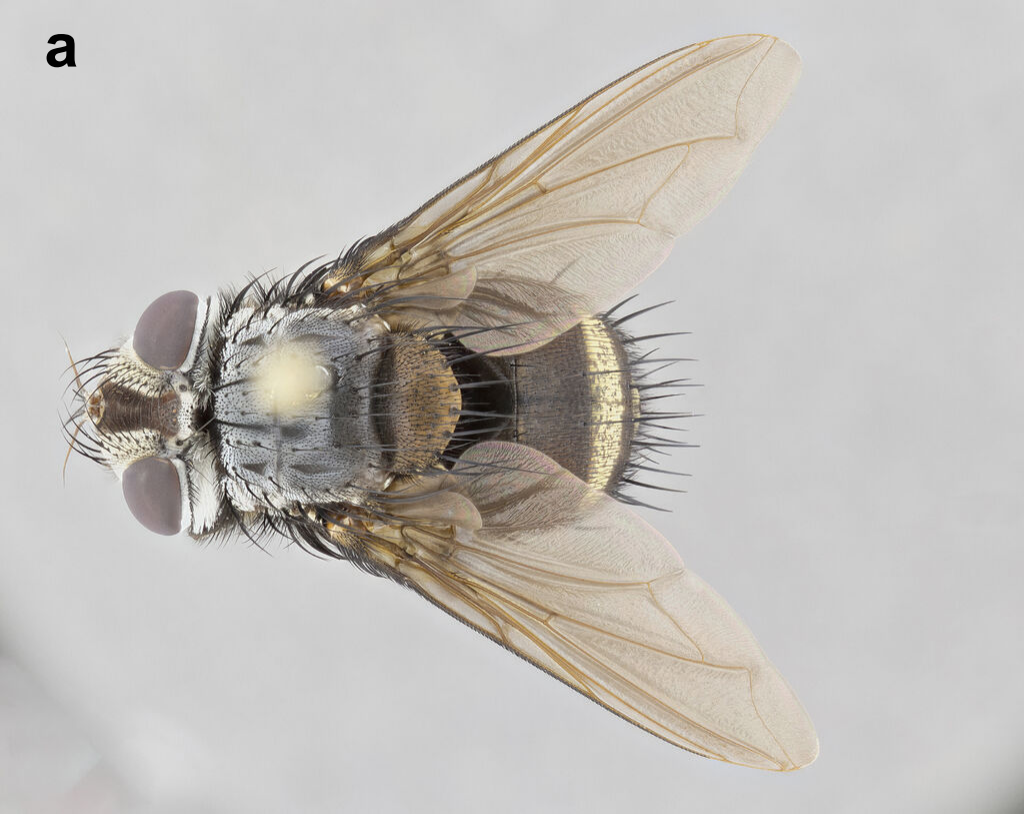
dorsal view

**Figure 66b.**
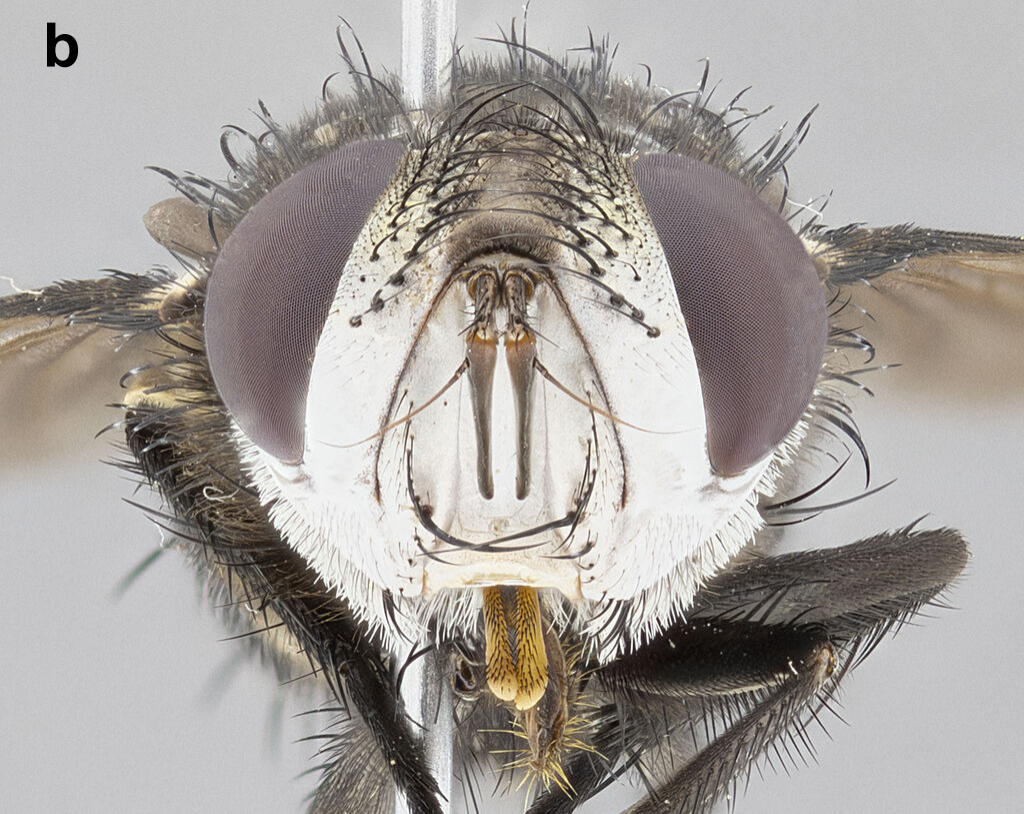
frontal view

**Figure 66c.**
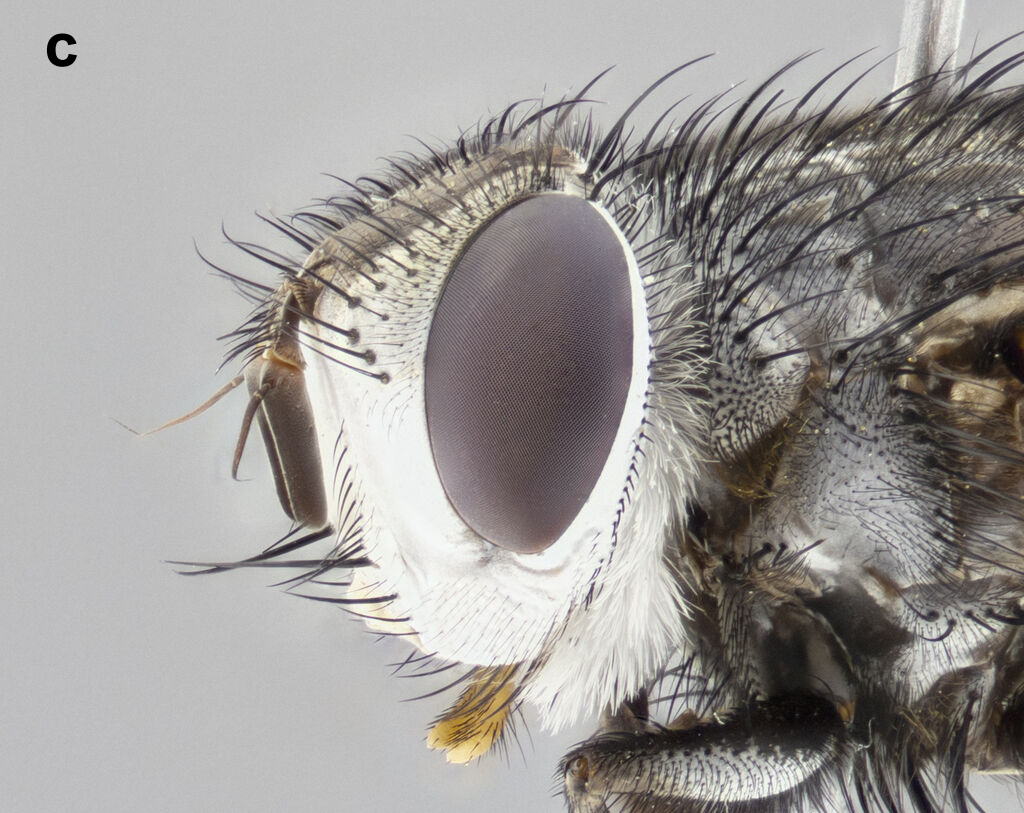
three quarters view

**Figure 66d.**
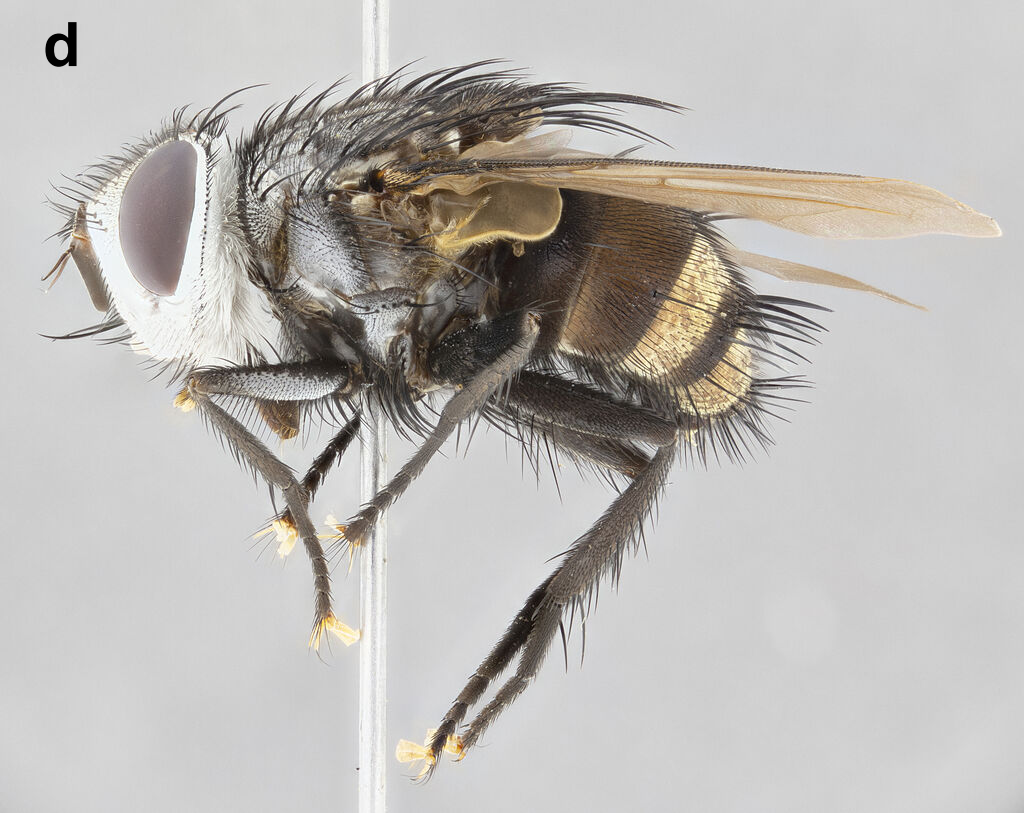
lateral view

**Figure 67a.**
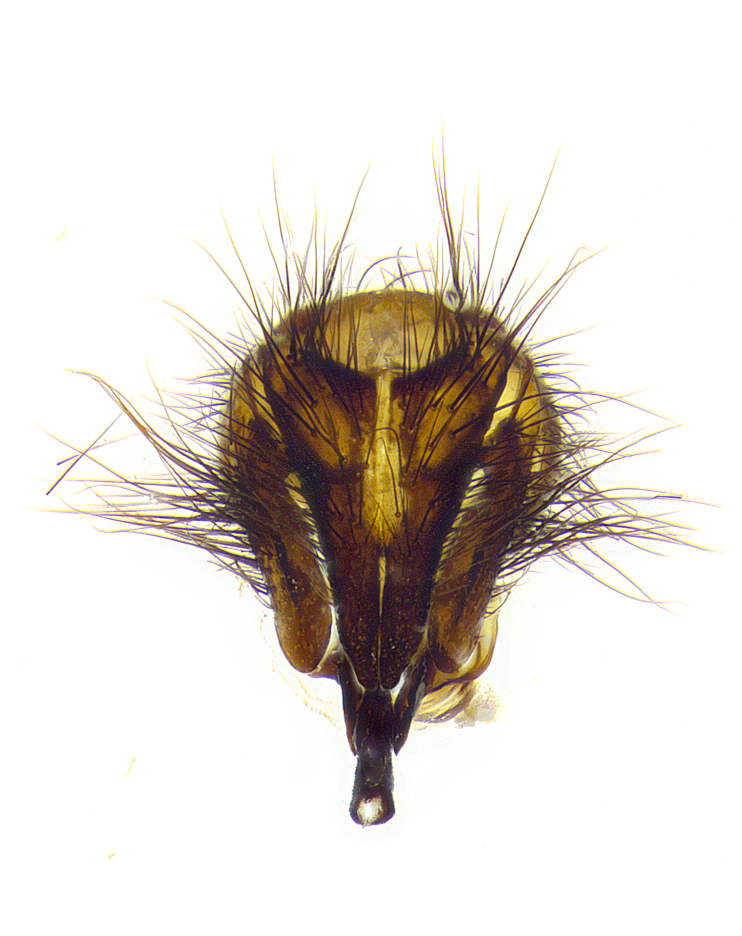
caudal view

**Figure 67b.**
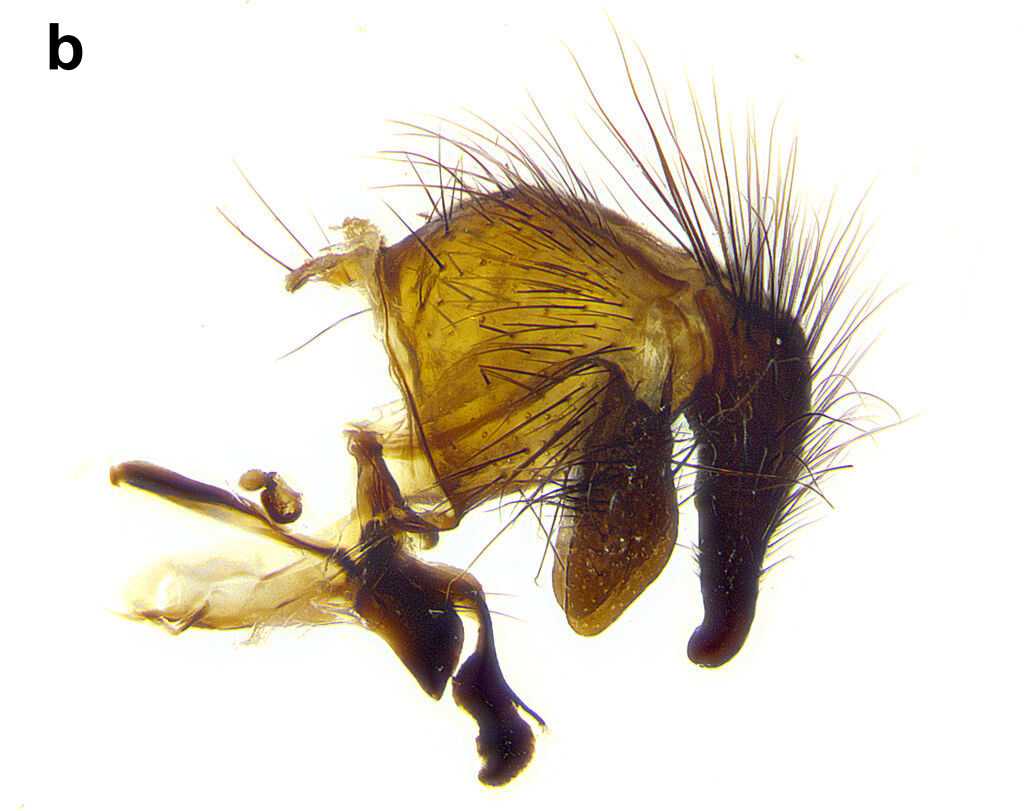
lateral view

**Figure 67c.**
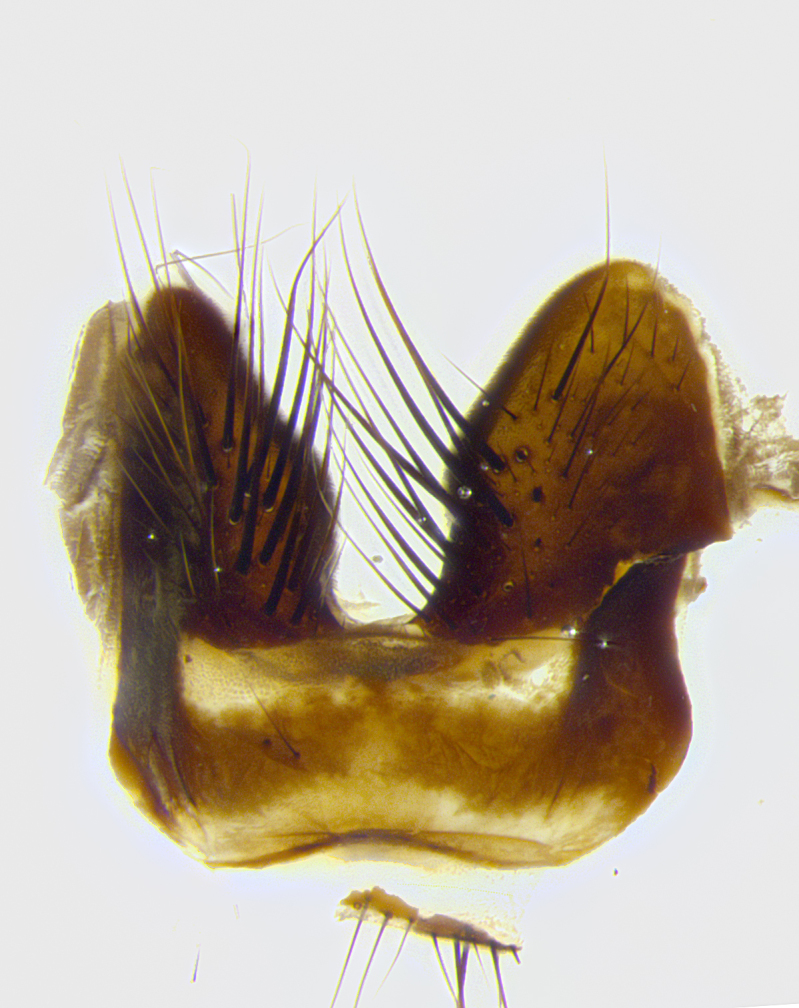
sternite 5, ventral view

**Figure 68a.**
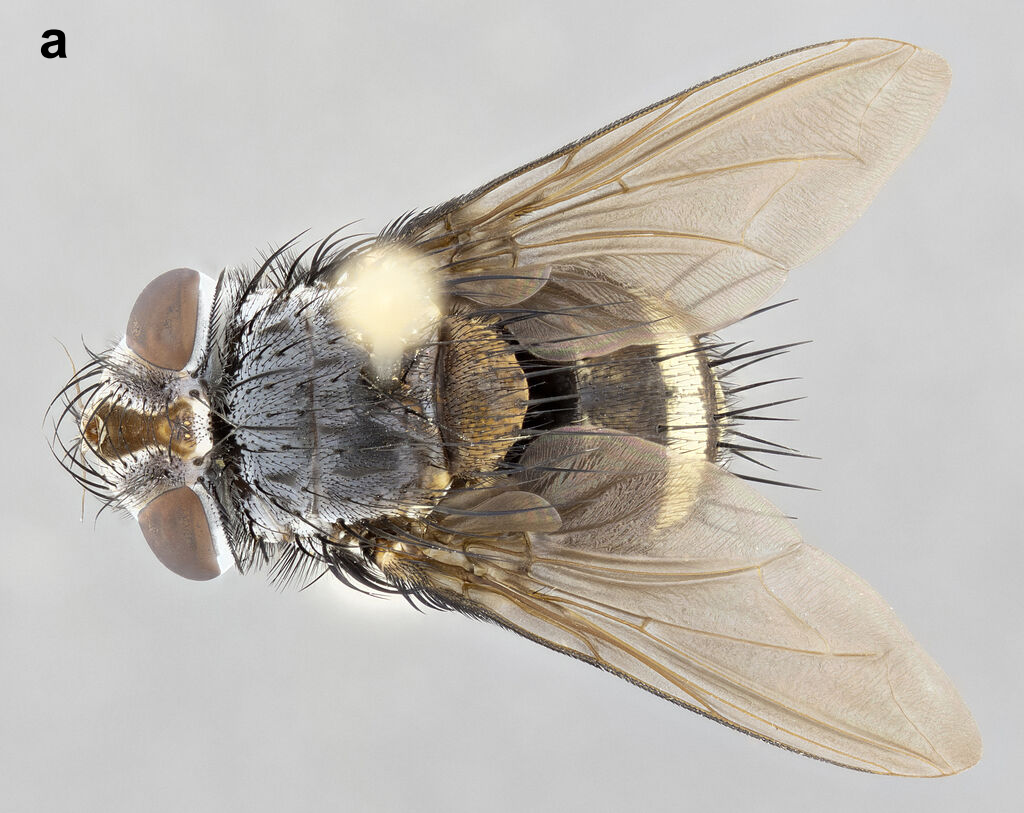
dorsal view

**Figure 68b.**
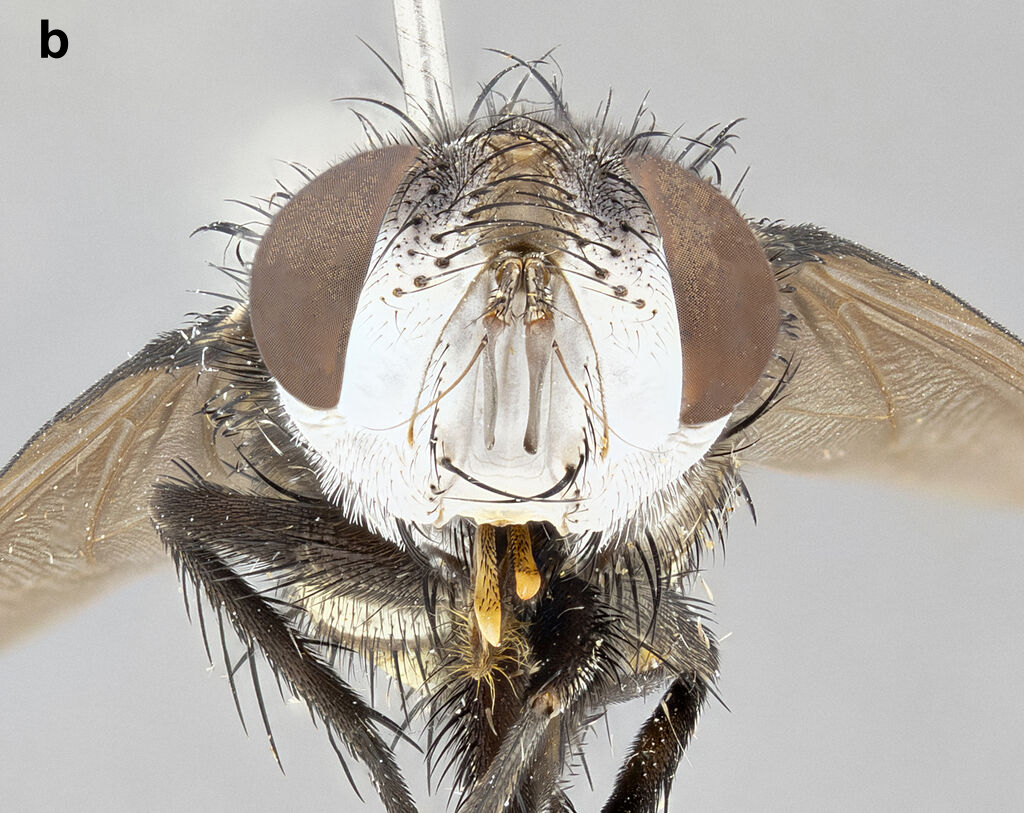
frontal view

**Figure 68c.**
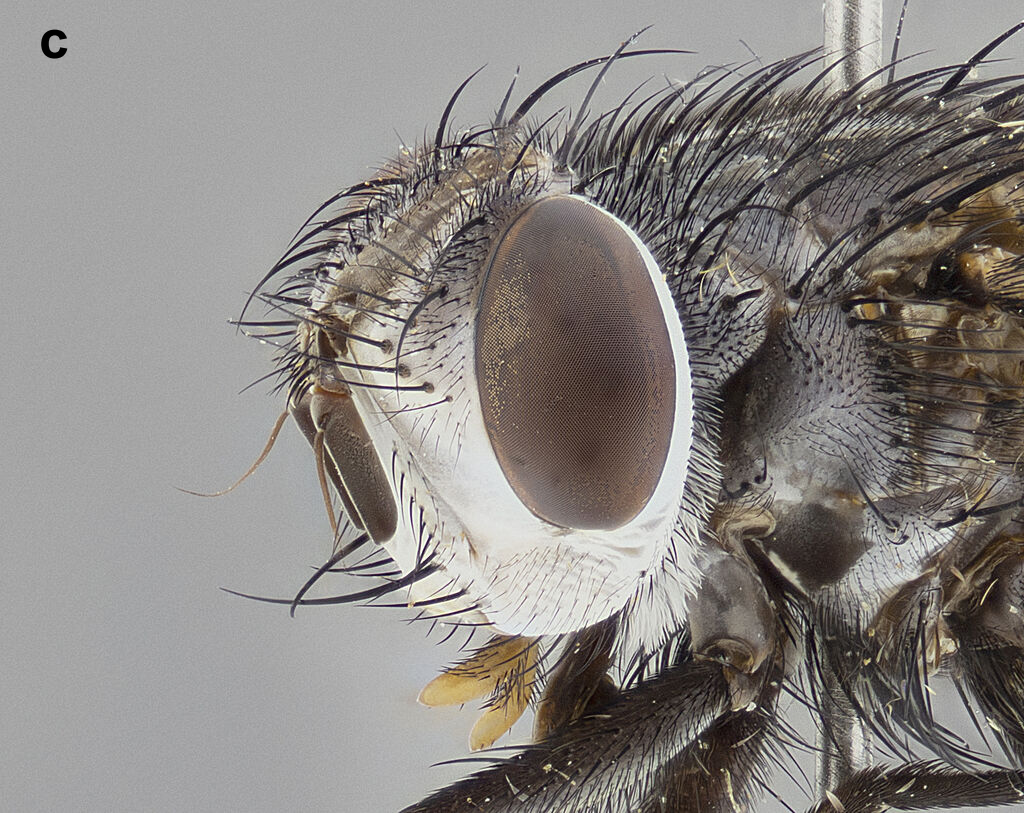
three quarters view

**Figure 68d.**
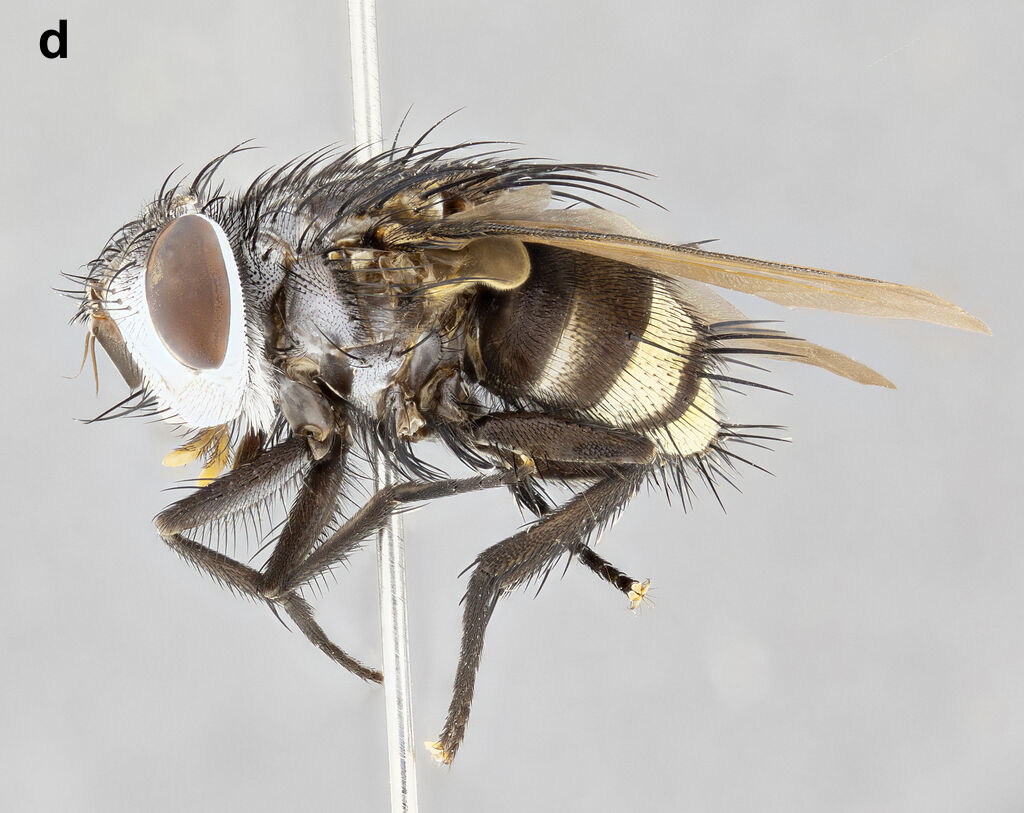
lateral view

**Figure 69a.**
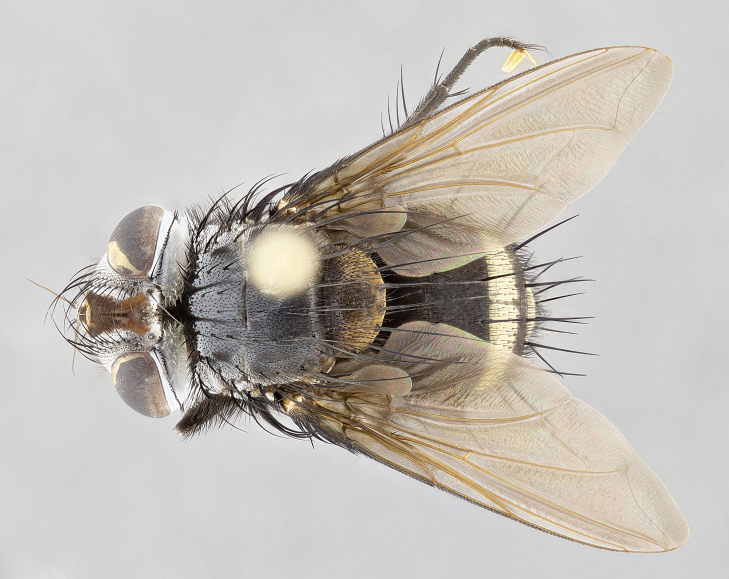
dorsal view

**Figure 69b.**
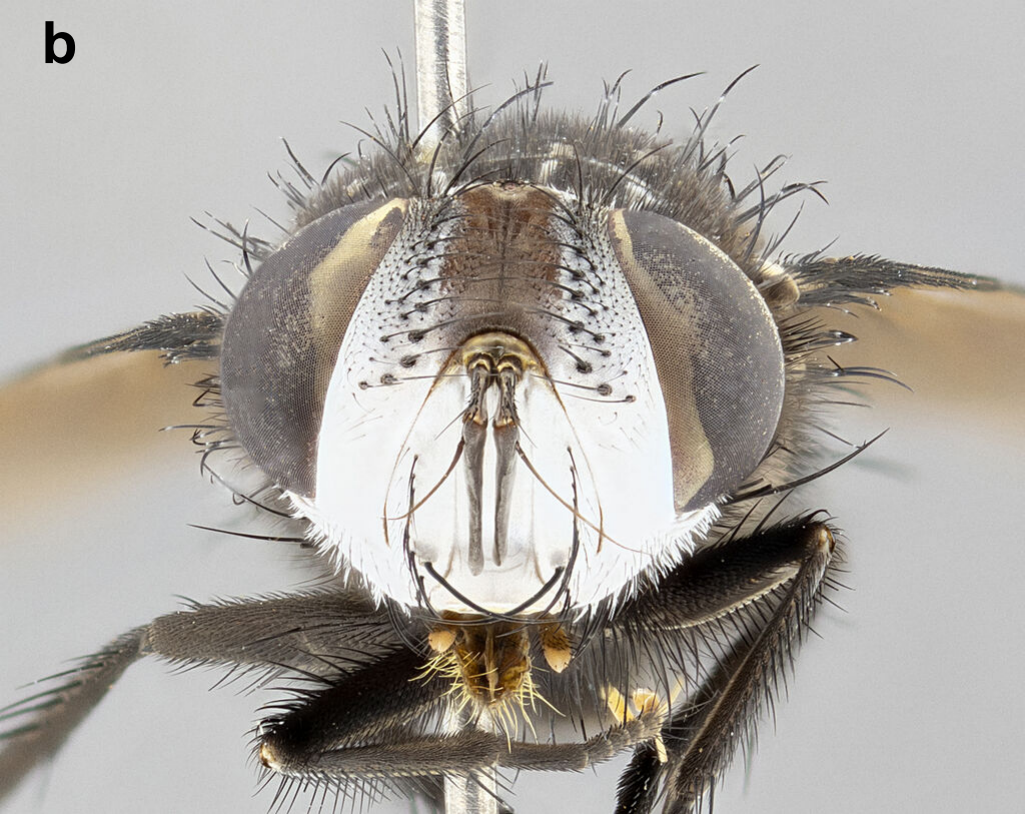
frontal view

**Figure 69c.**
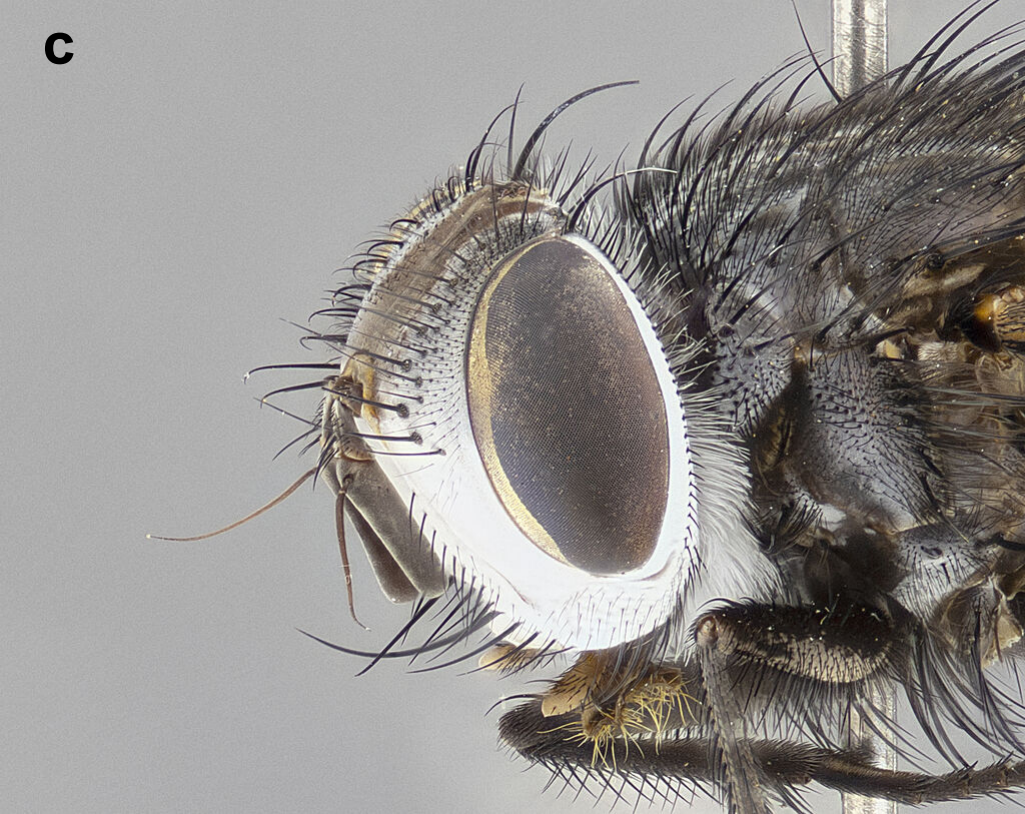
three quarters view

**Figure 69d.**
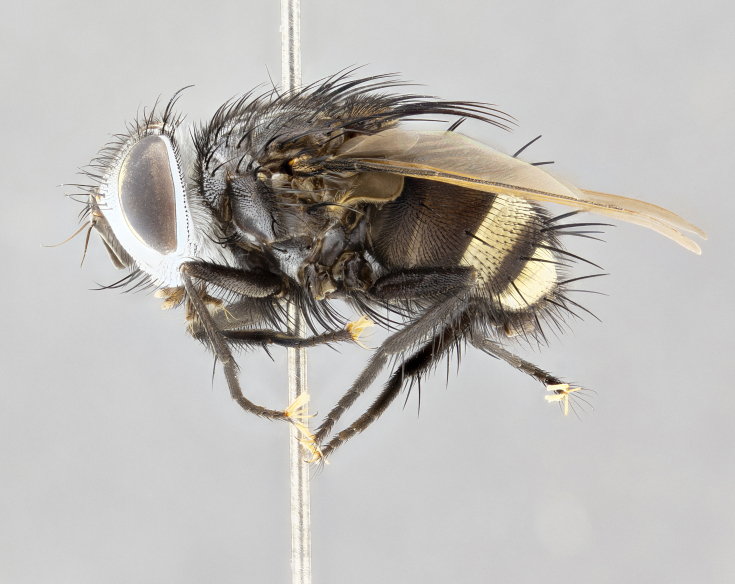
lateral view

**Figure 70a.**
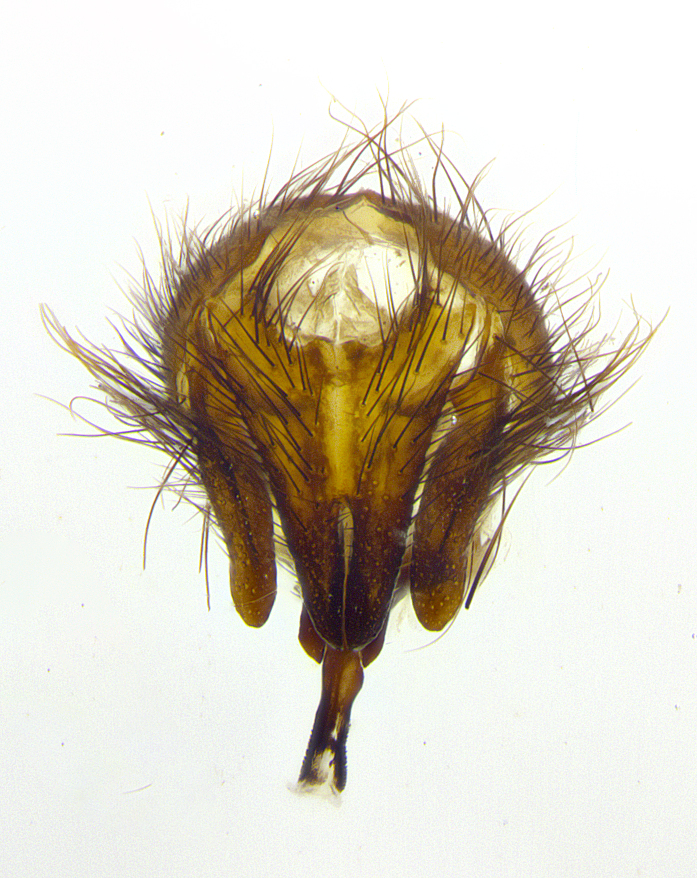
caudal view

**Figure 70b.**
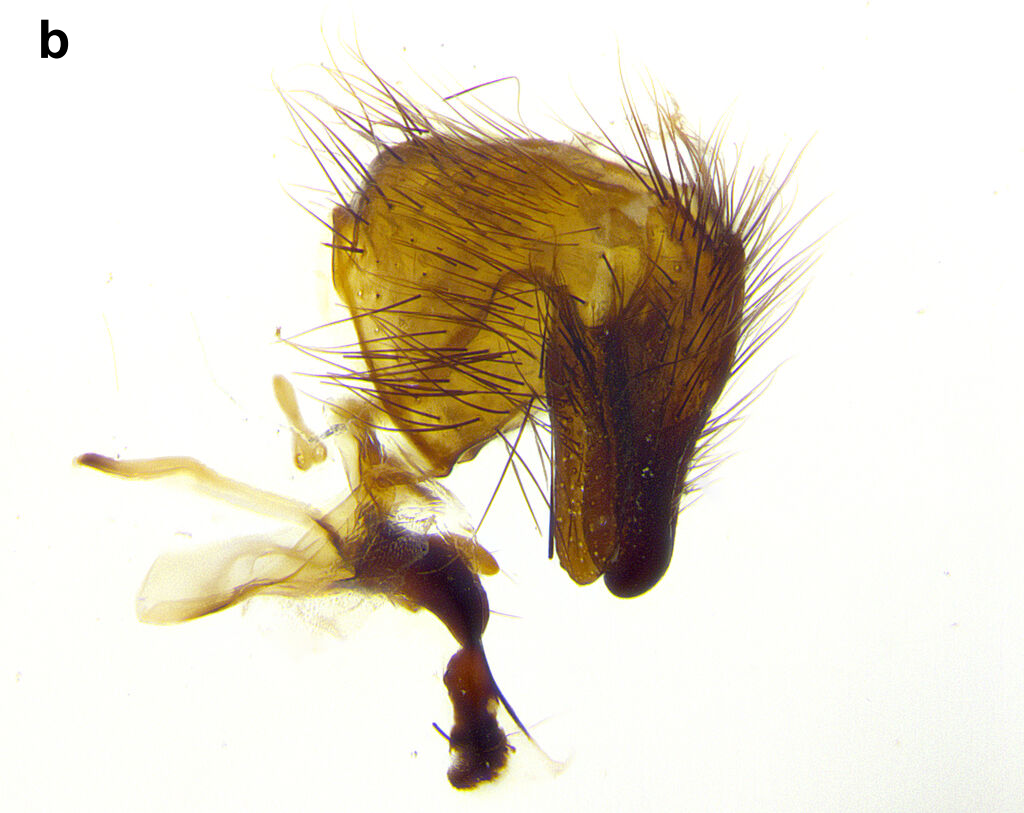
lateral view

**Figure 70c.**
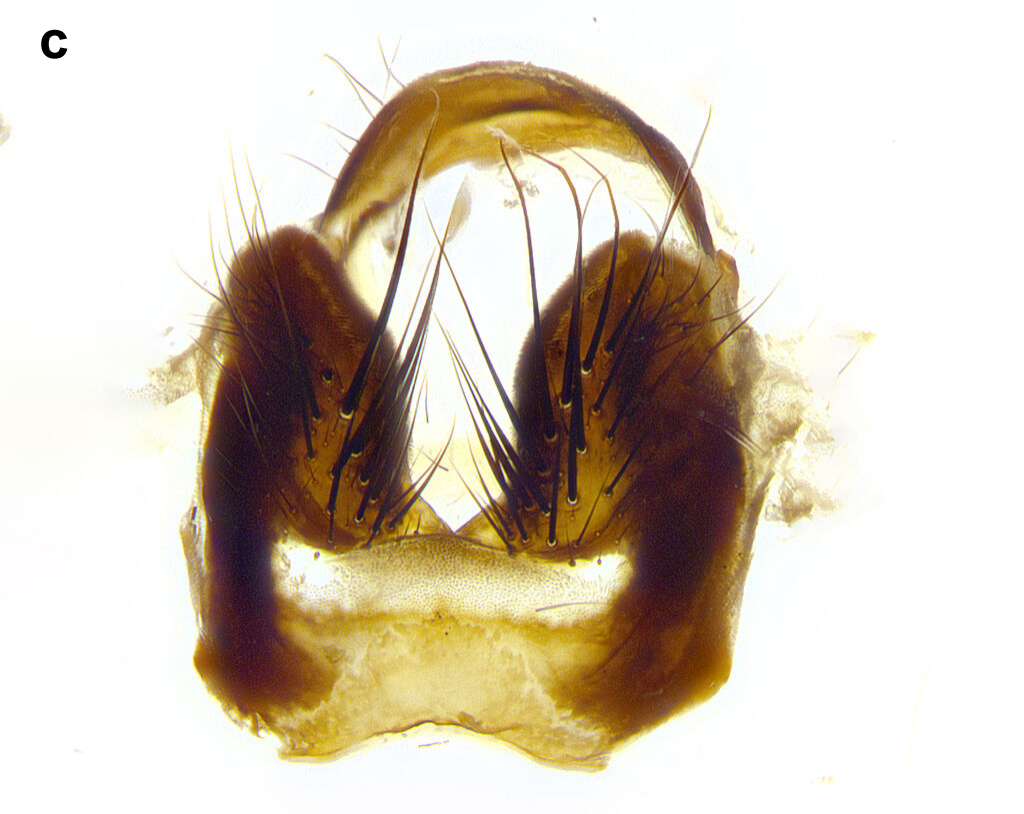
sternite 5, ventral view

**Figure 71a.**
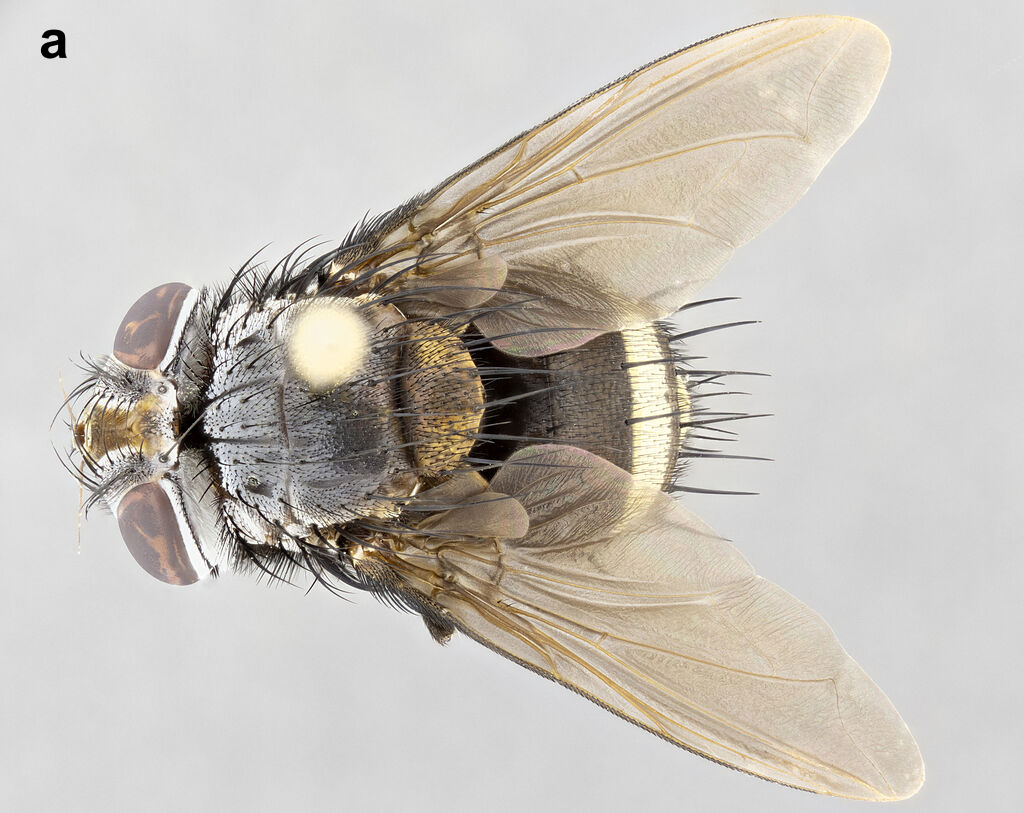
dorsal view

**Figure 71b.**
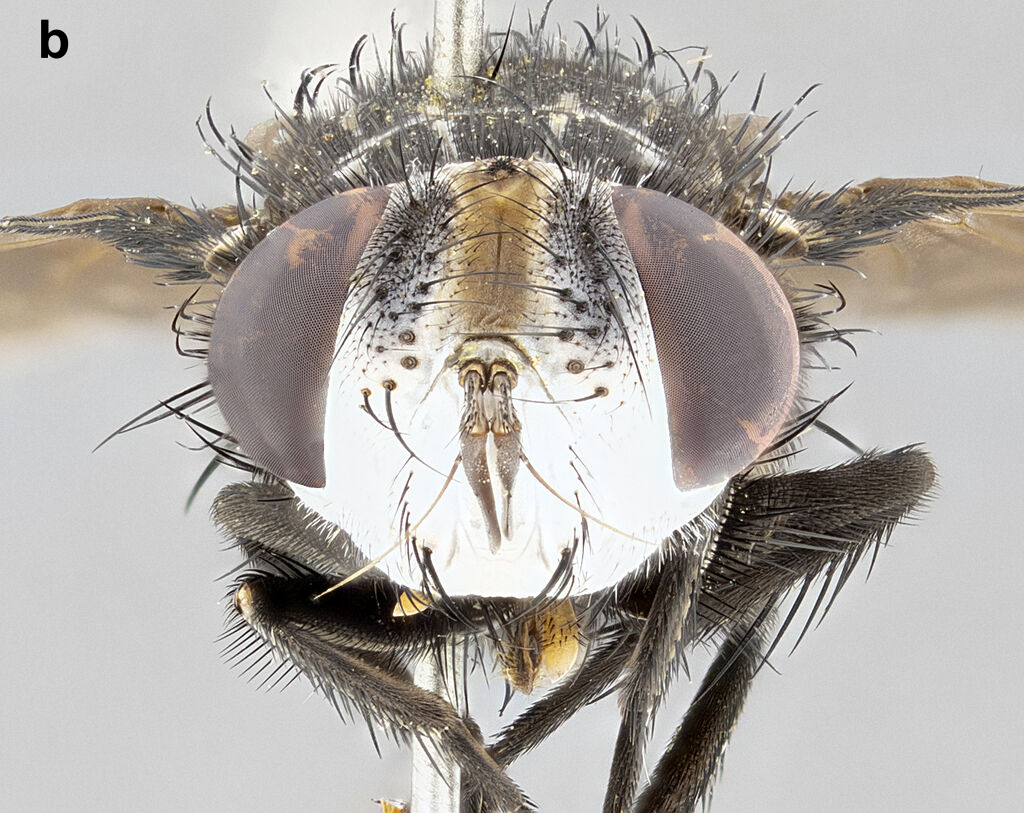
frontal view

**Figure 71c.**
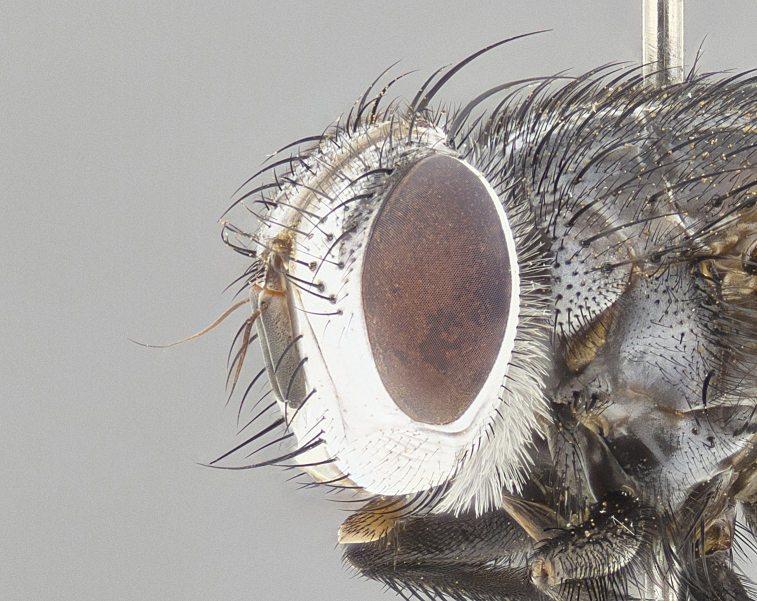
three quarters view

**Figure 71d.**
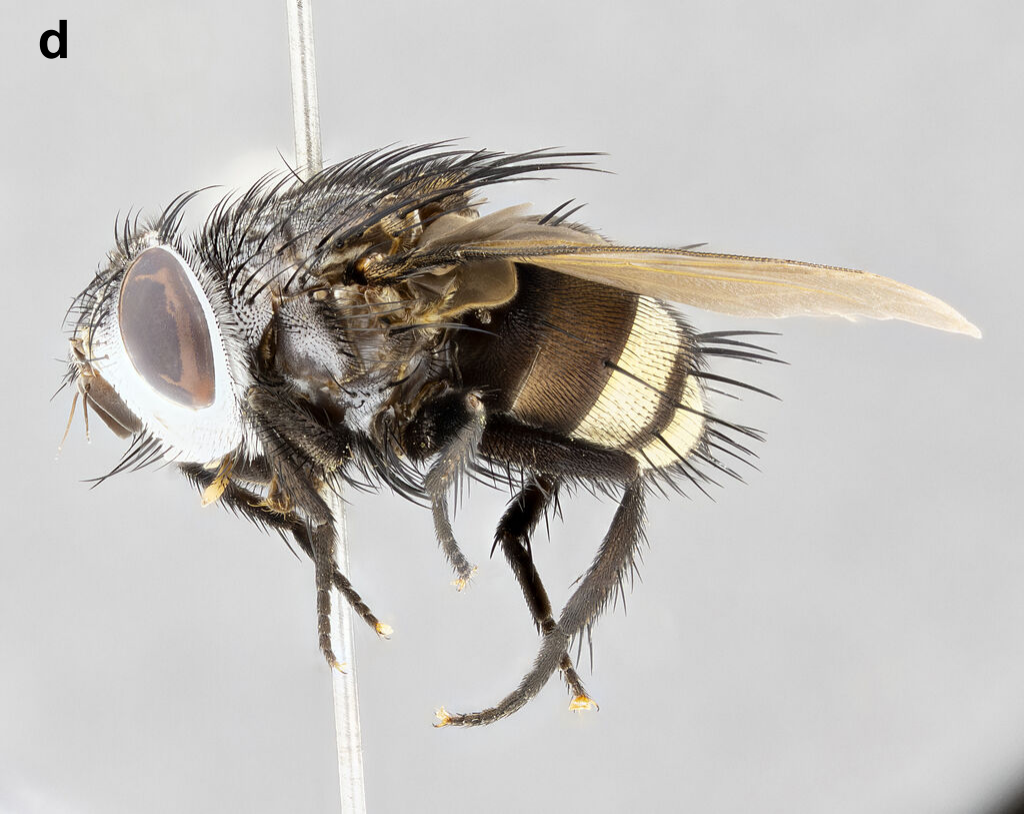
lateral view

**Figure 72a.**
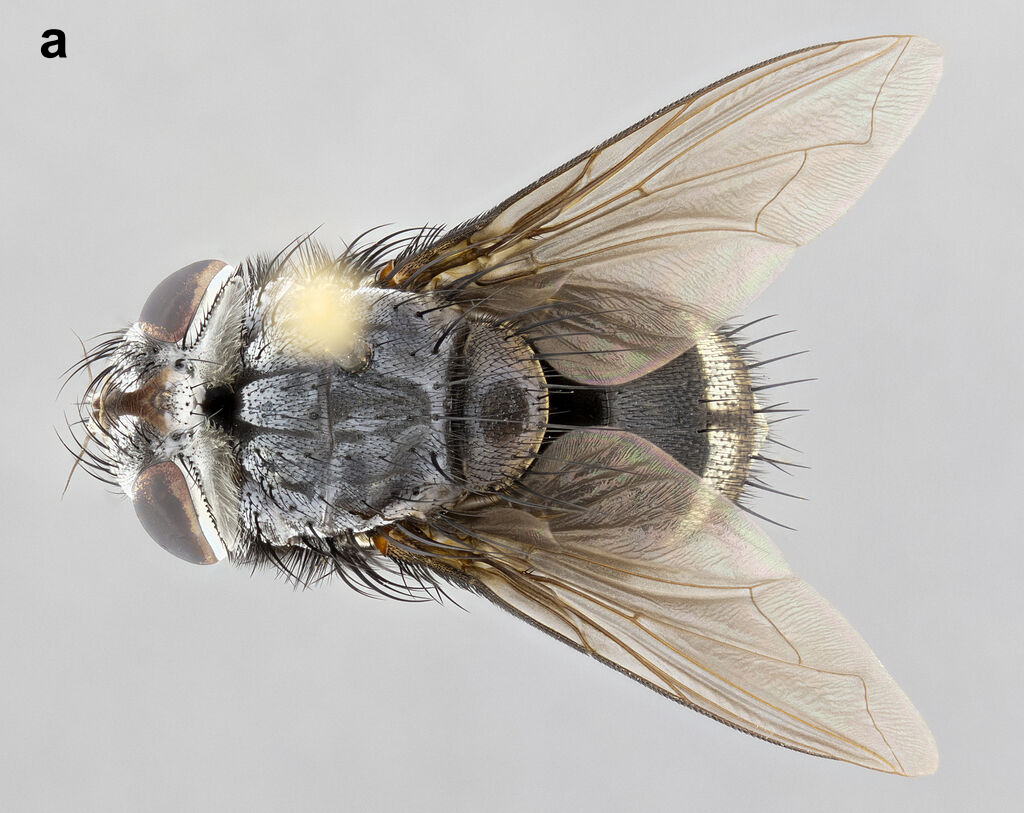
dorsal view

**Figure 72b.**
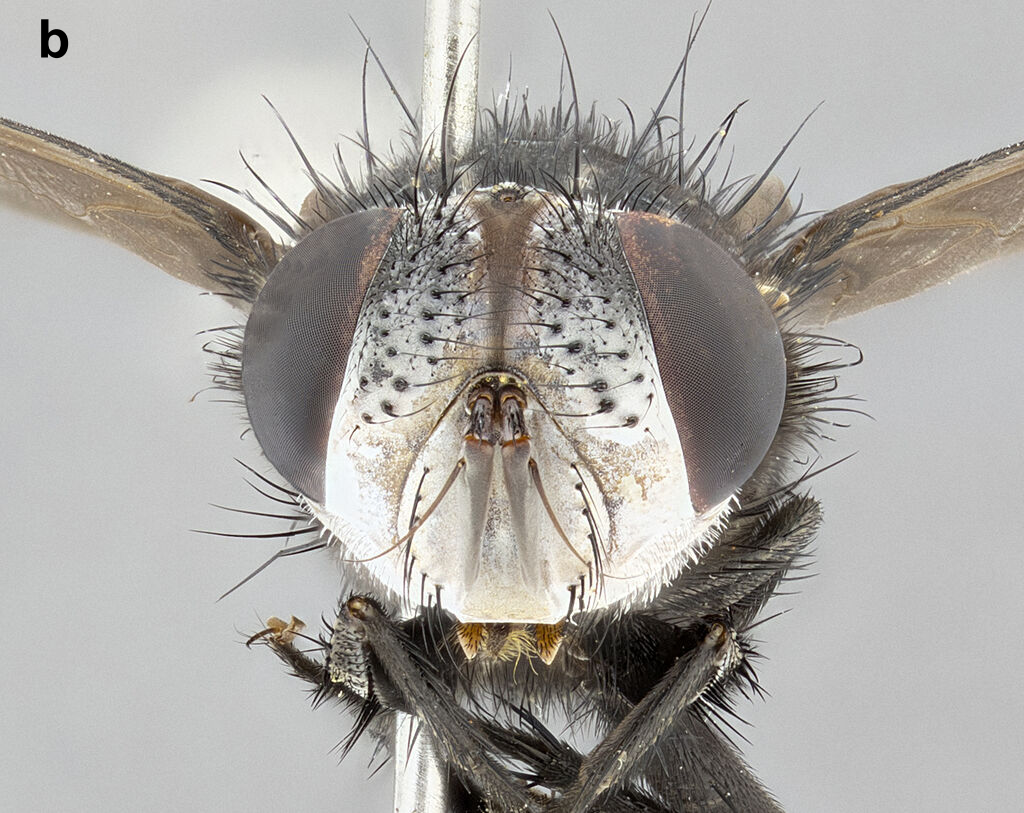
frontal view

**Figure 72c.**
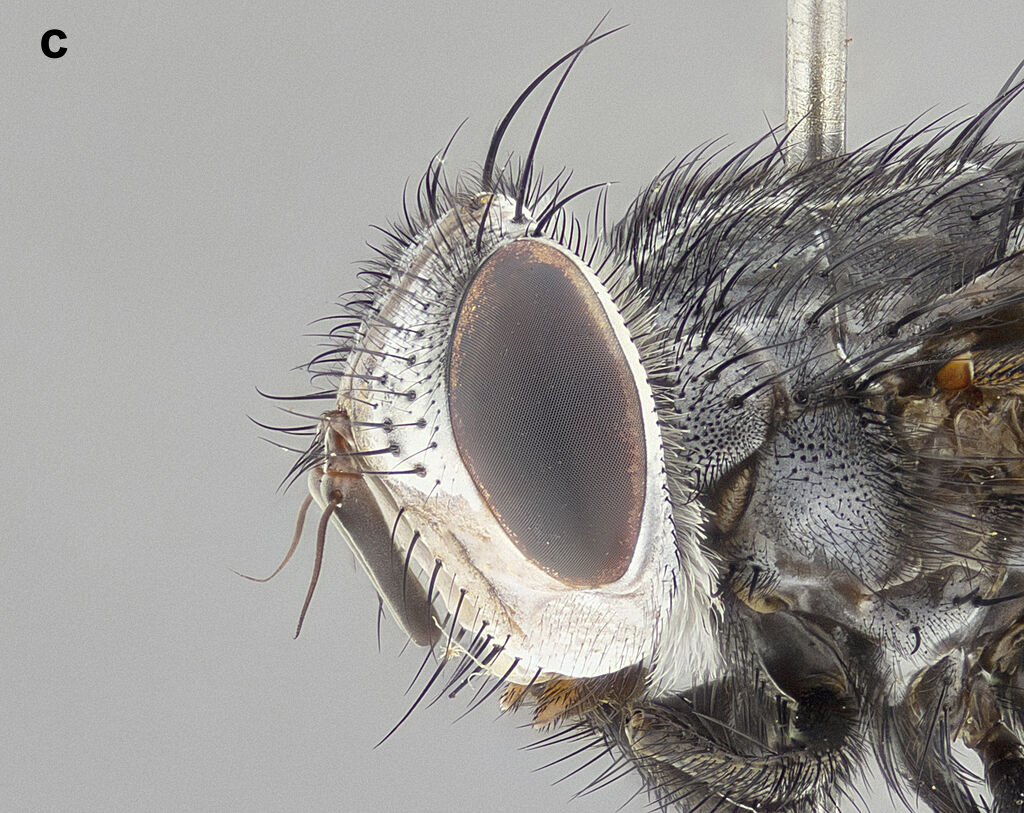
three quarters view

**Figure 72d.**
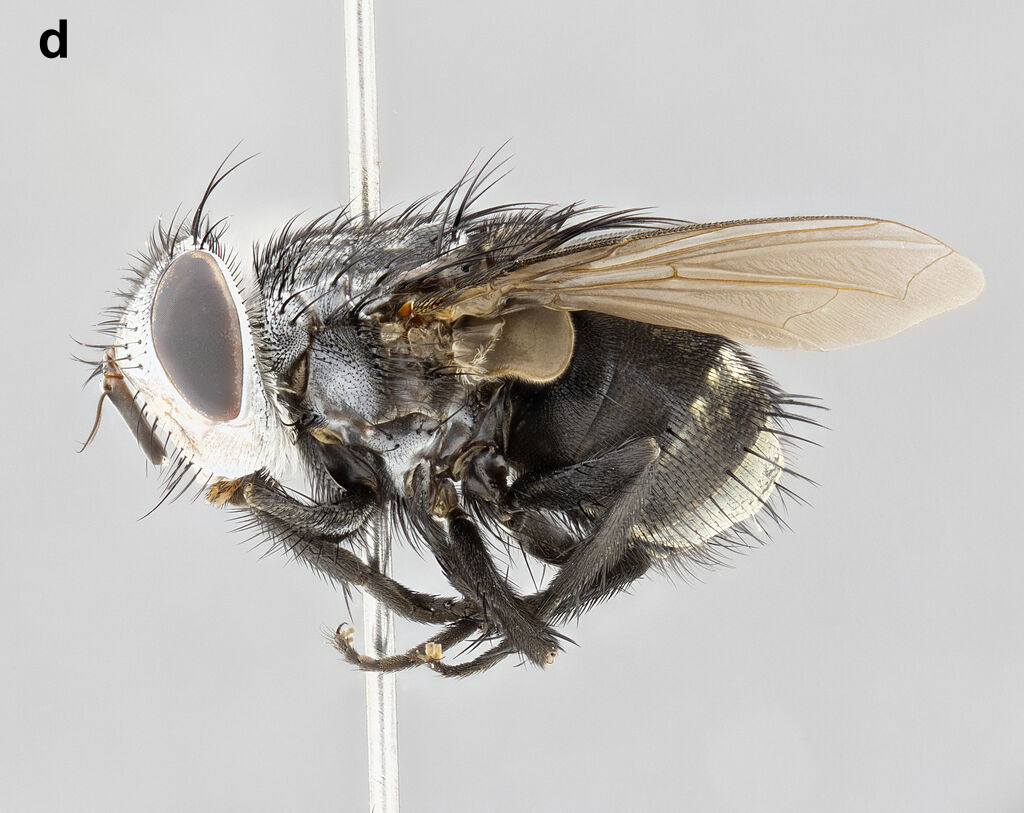
lateral view

**Figure 73a.**
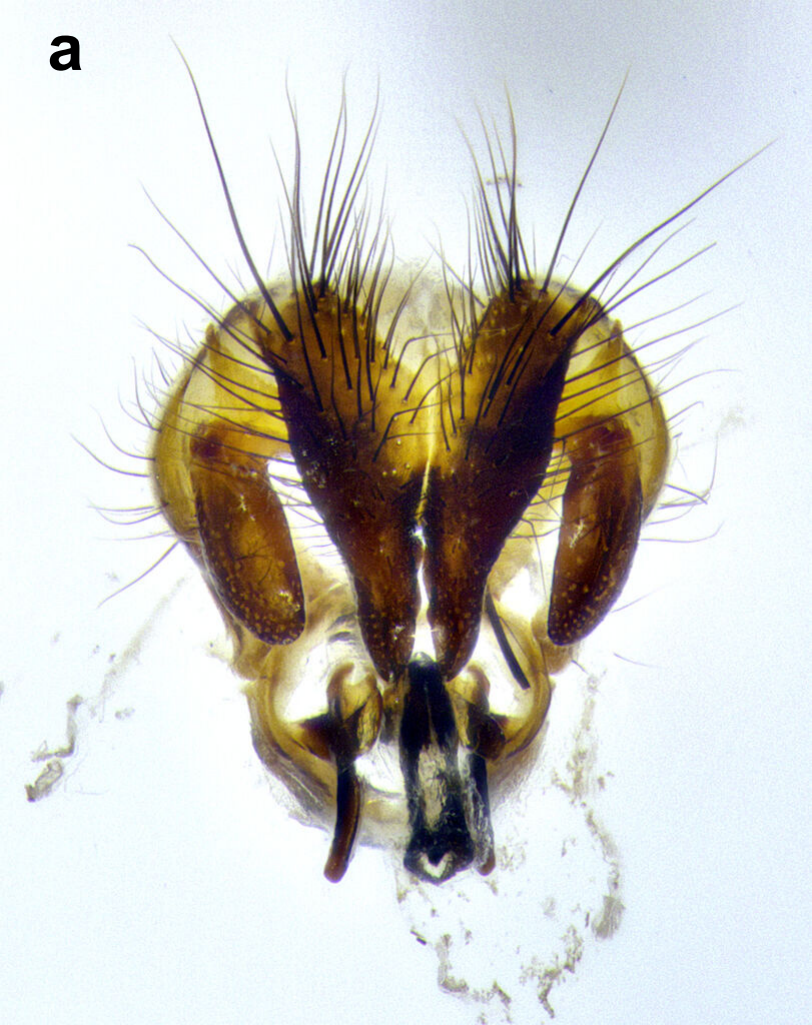
caudal view

**Figure 73b.**
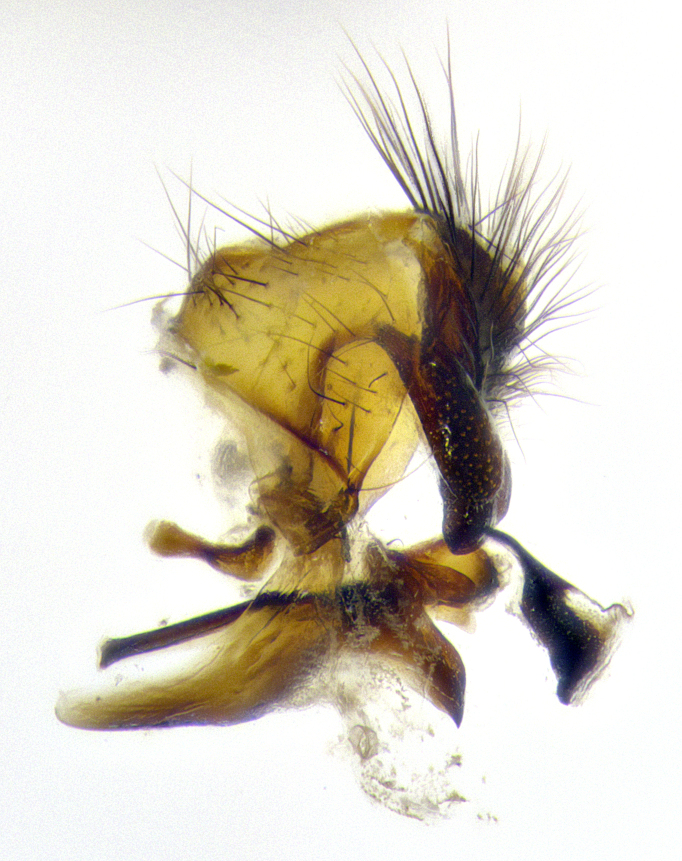
lateral view

**Figure 73c.**
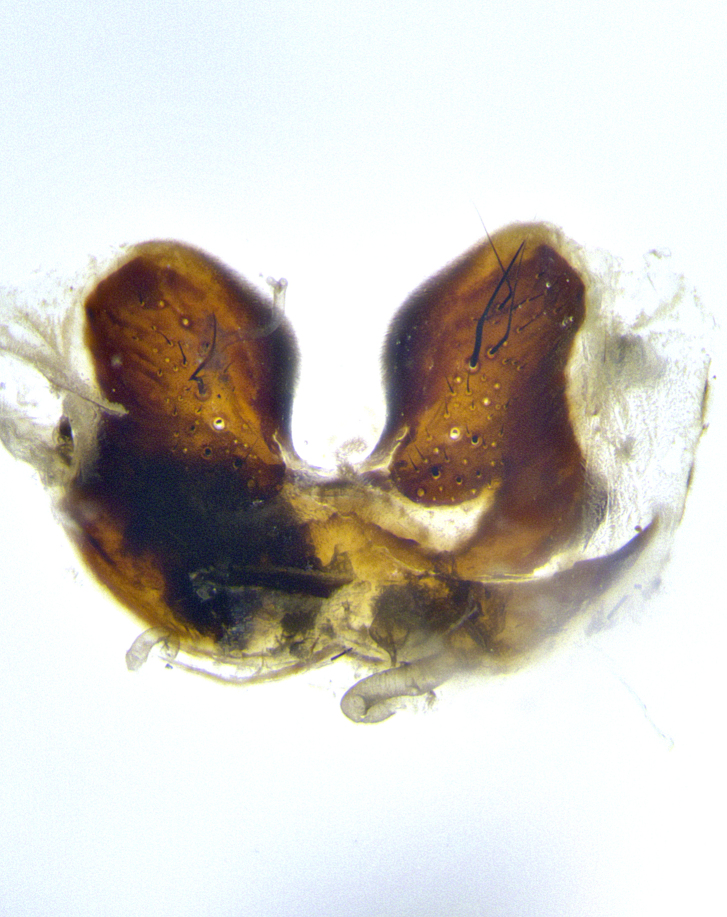
sternite 5, ventral view

**Figure 74a.**
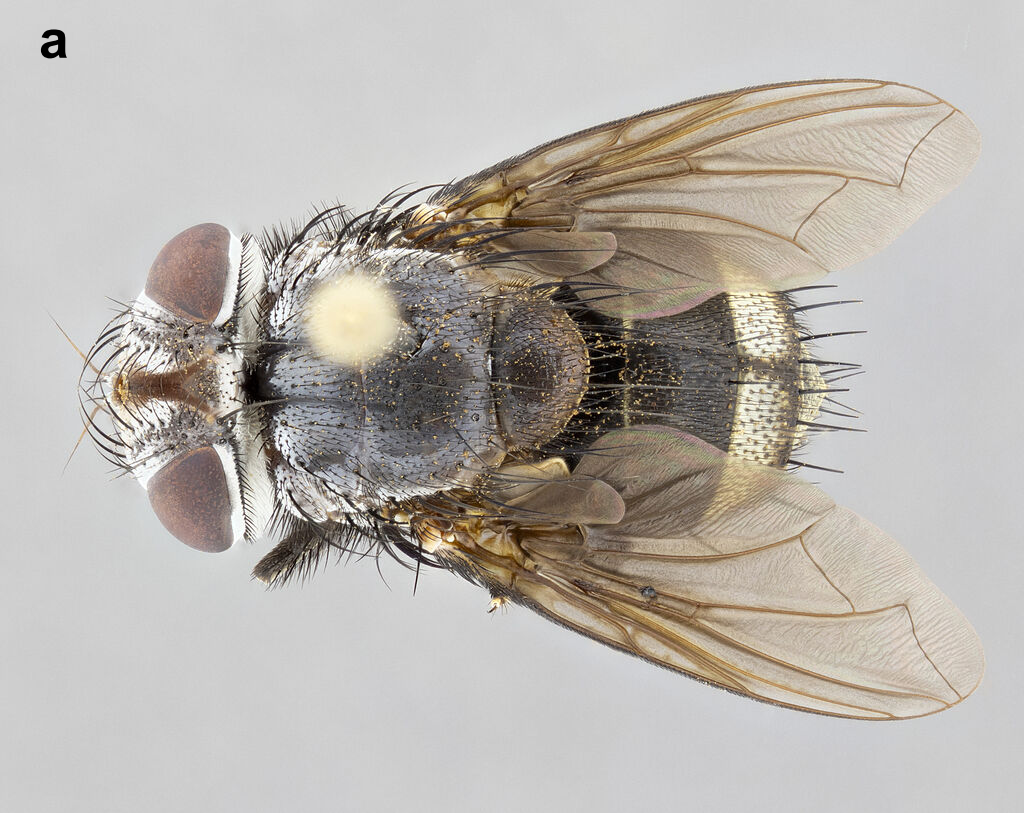
dorsal view

**Figure 74b.**
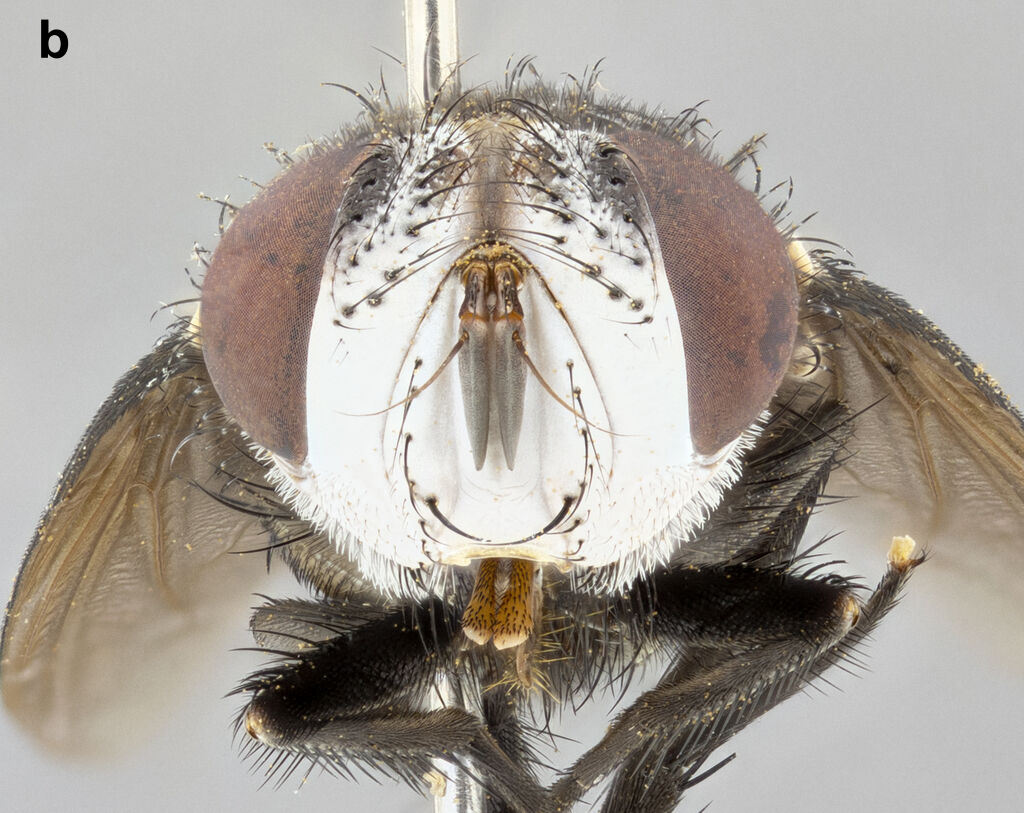
frontal view

**Figure 74c.**
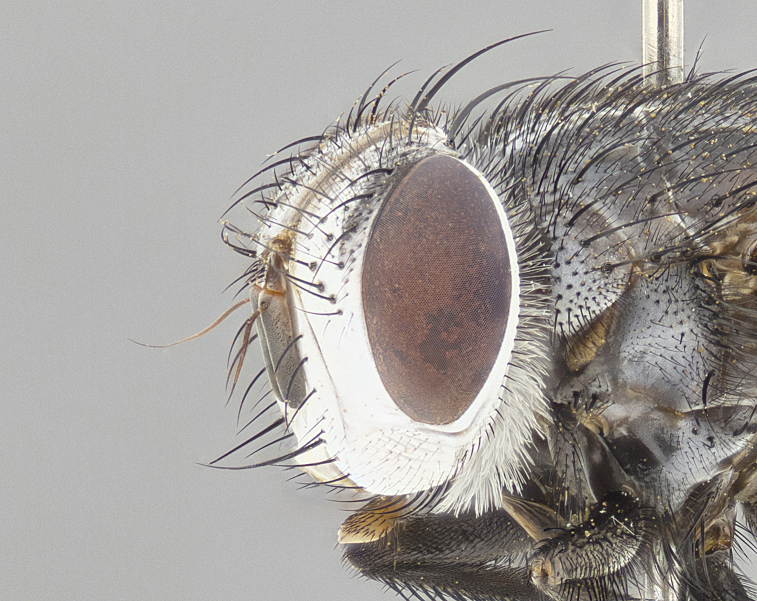
three quarters view

**Figure 74d.**
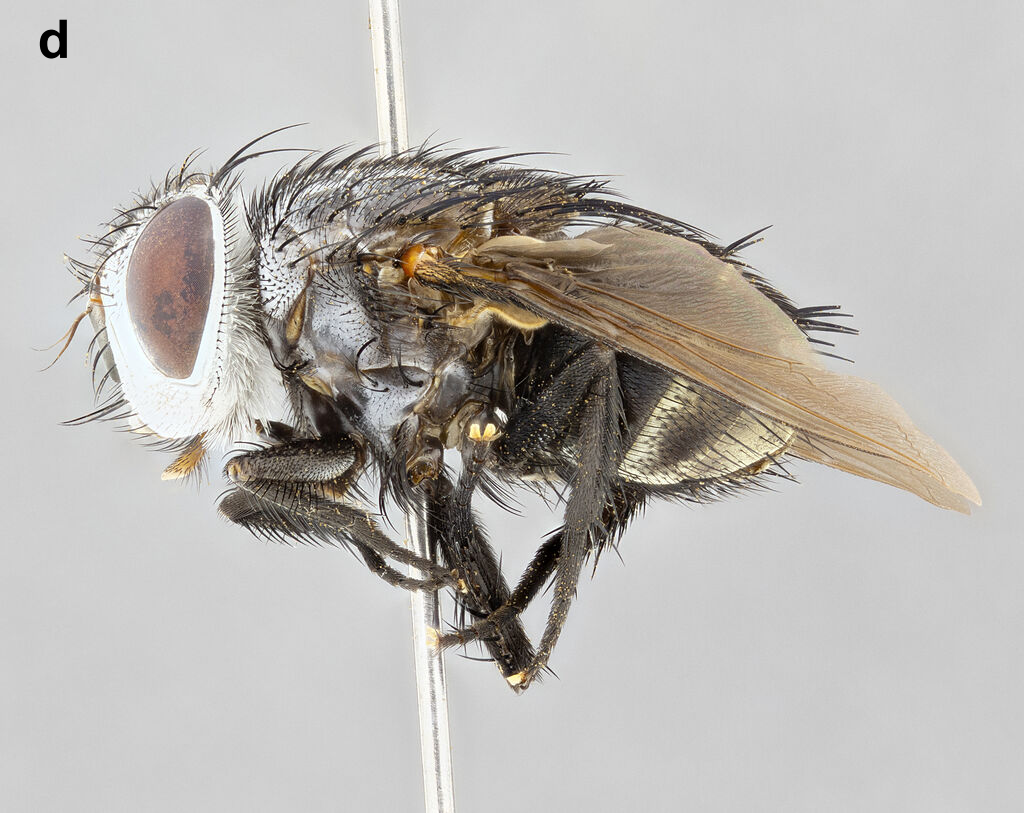
lateral view

**Figure 75a.**
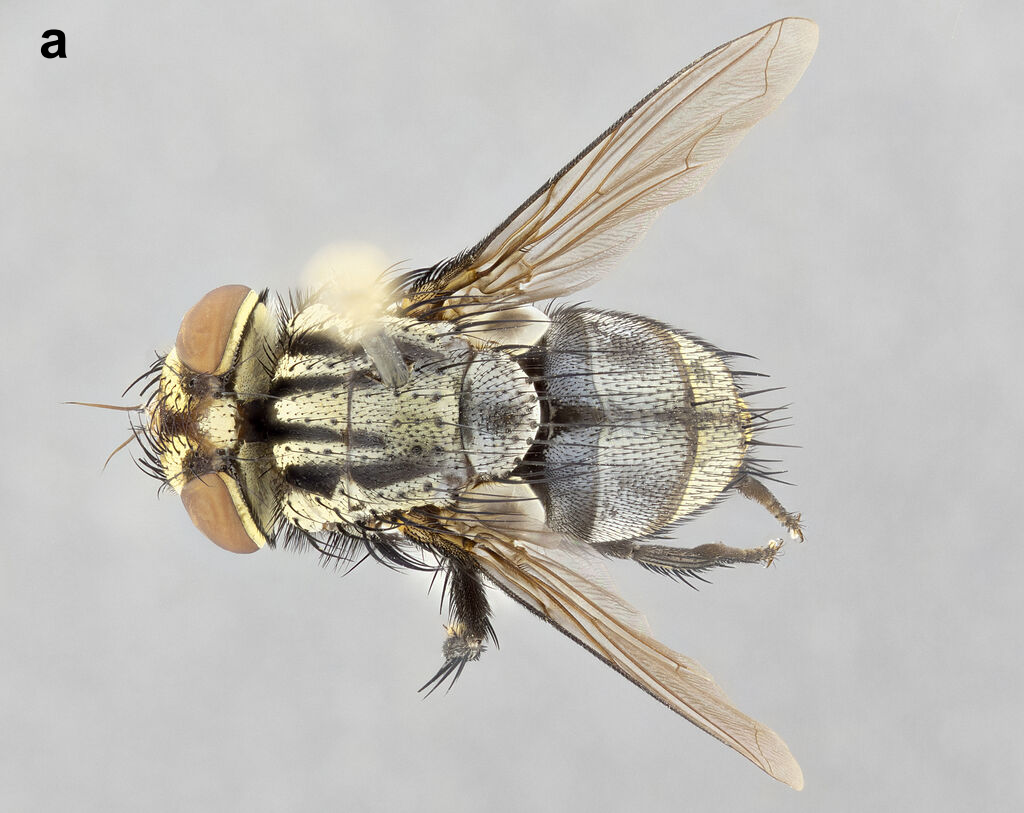
dorsal view

**Figure 75b.**
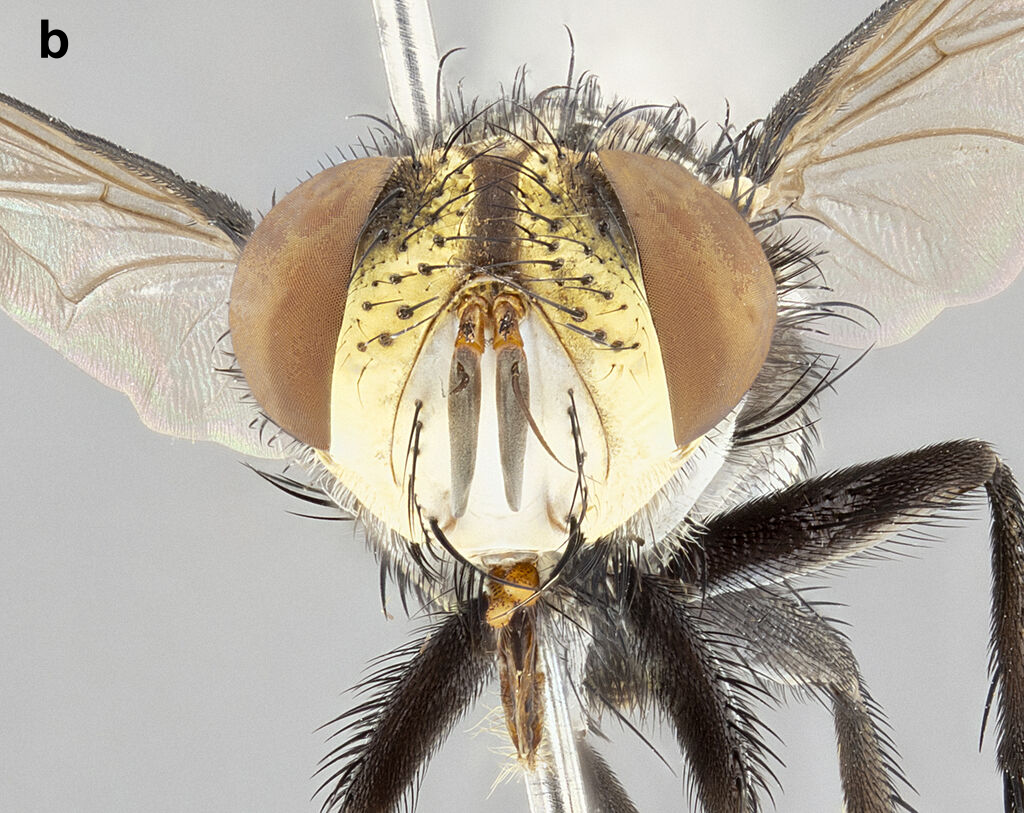
frontal view

**Figure 75c.**
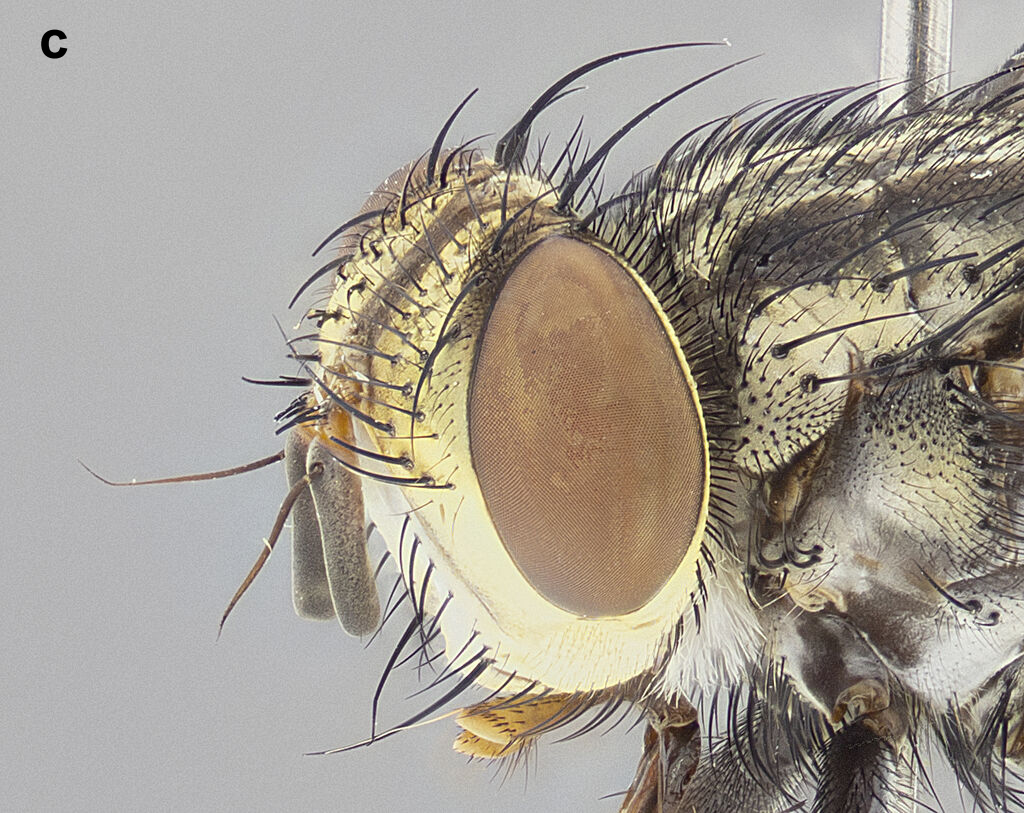
three quarters view

**Figure 75d.**
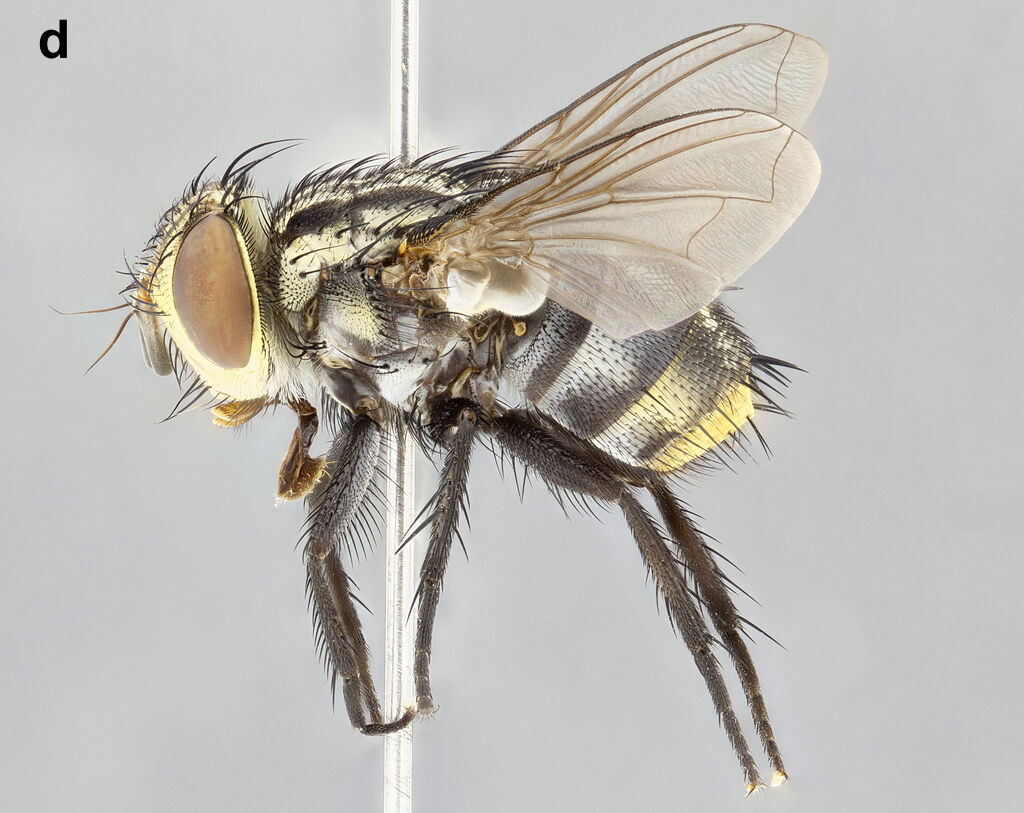
lateral view

**Figure 76a.**
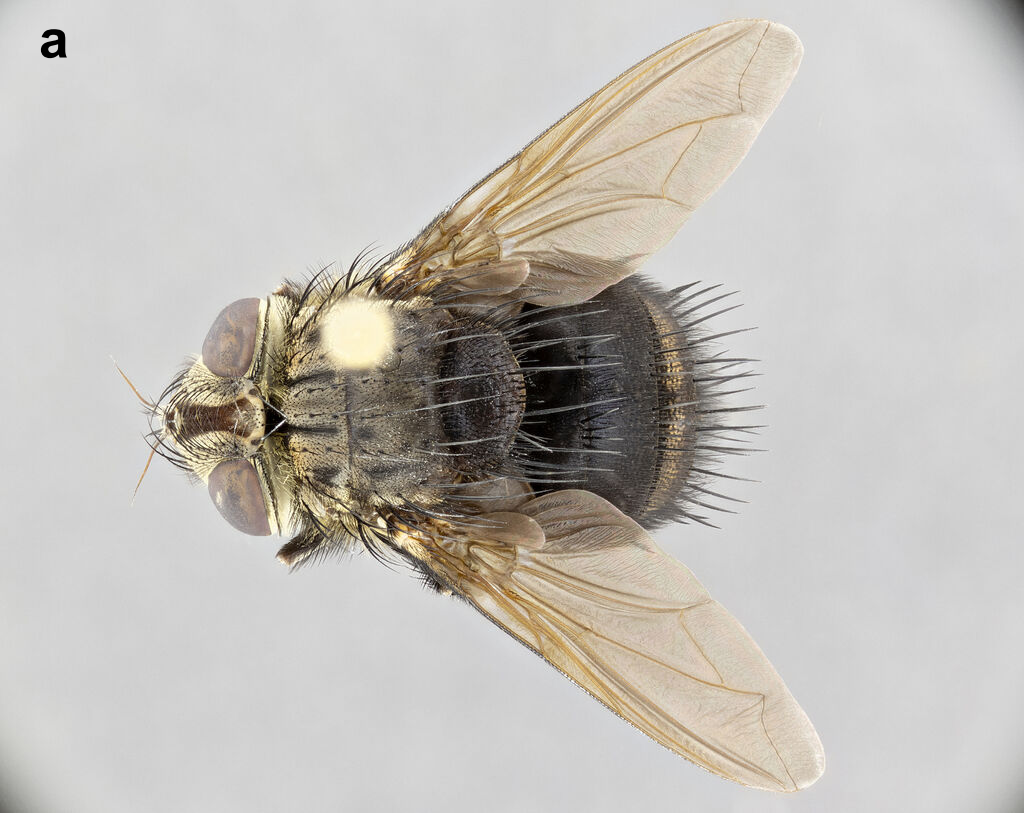
dorsal view

**Figure 76b.**
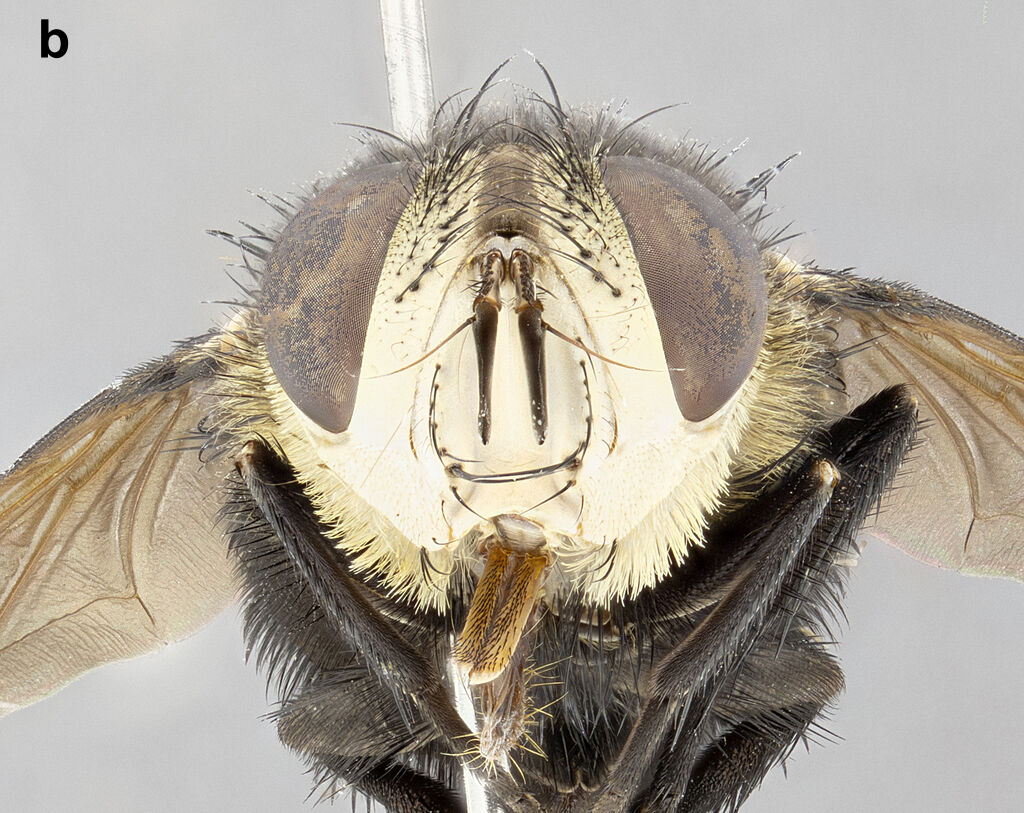
frontal view

**Figure 76c.**
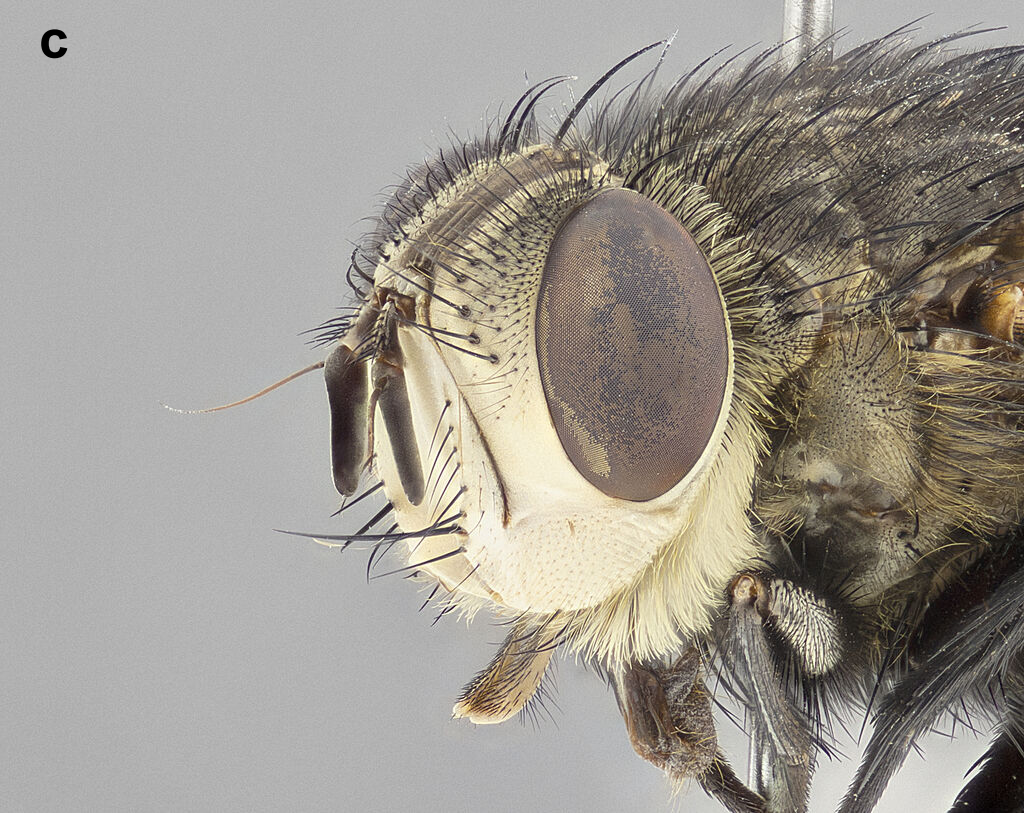
three quarters view

**Figure 76d.**
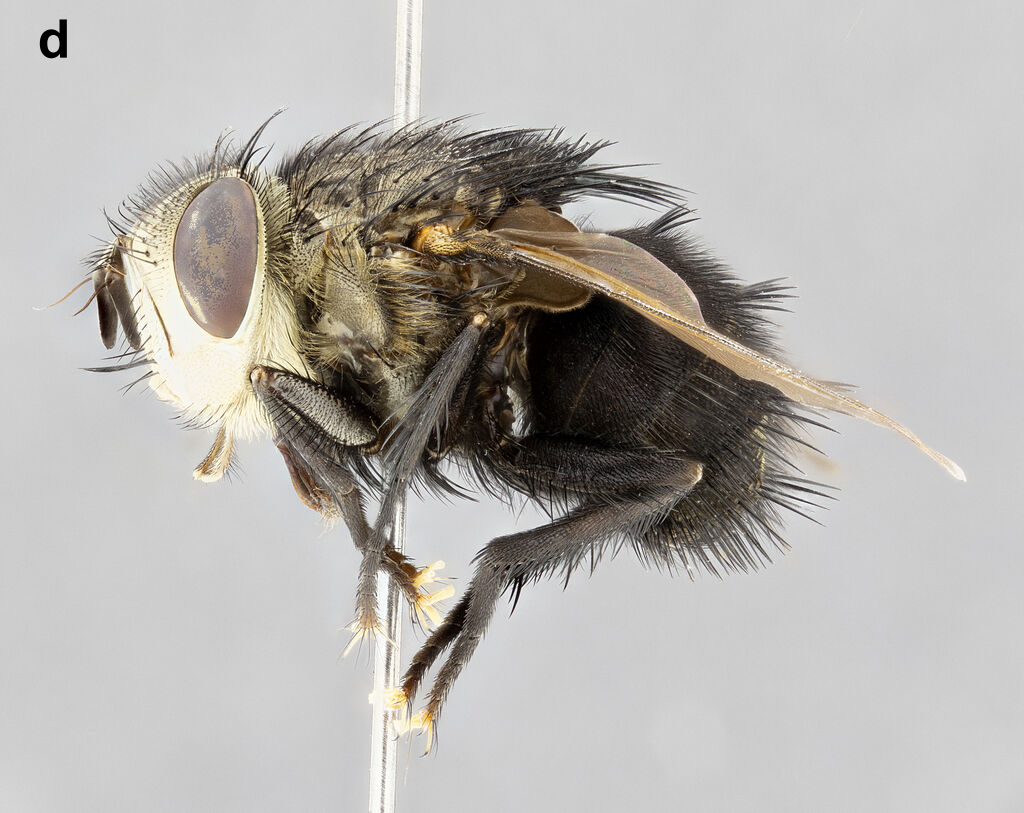
lateral view

**Figure 77a.**
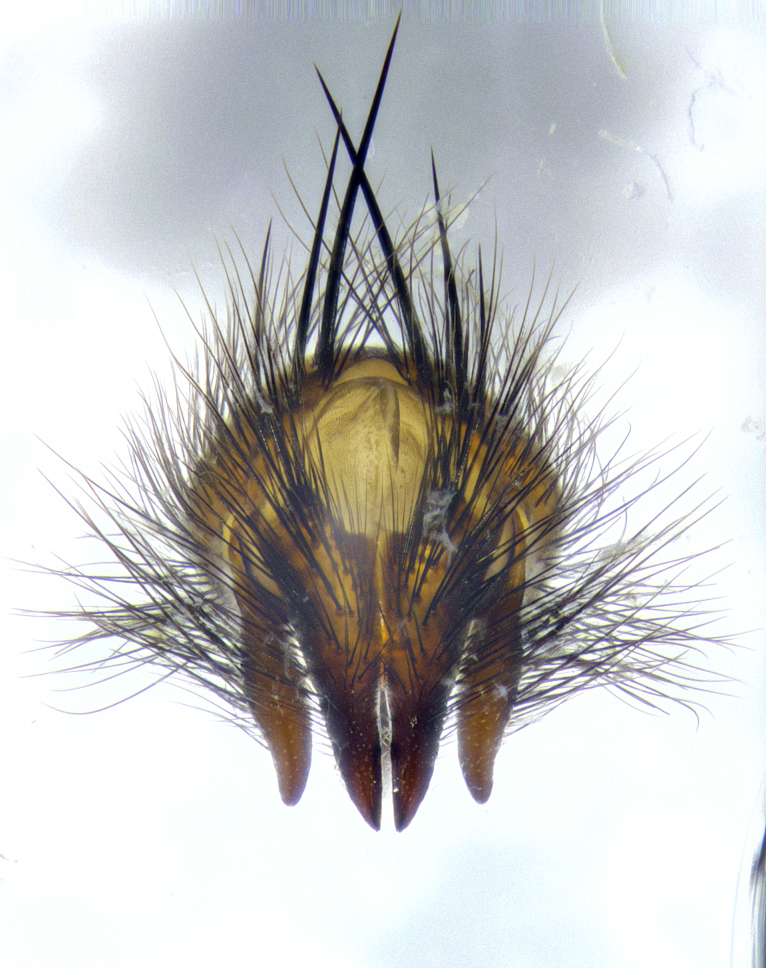
caudal view

**Figure 77b.**
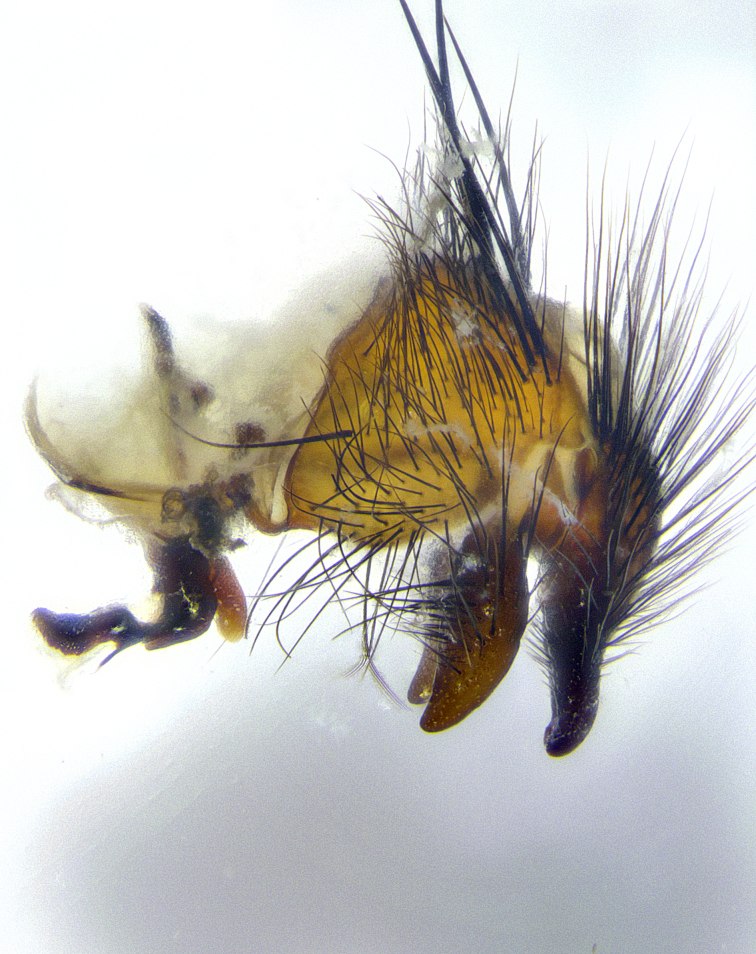
lateral view

**Figure 77c.**
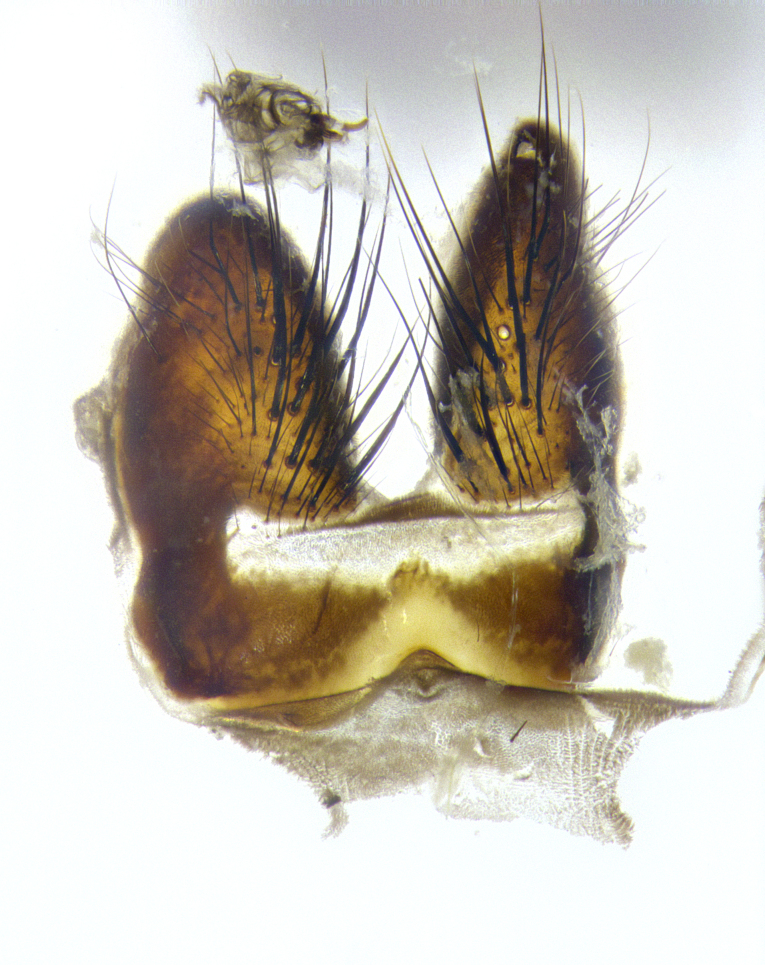
sternite 5, ventral view

**Figure 78a.**
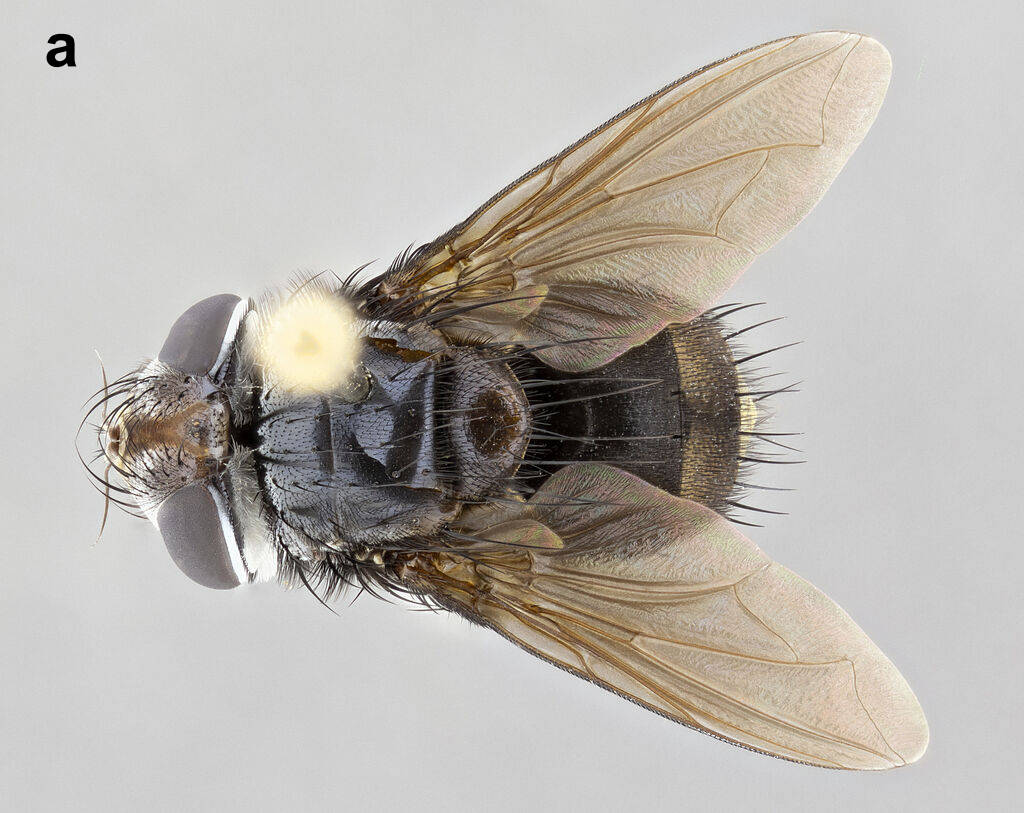
dorsal view

**Figure 78b.**
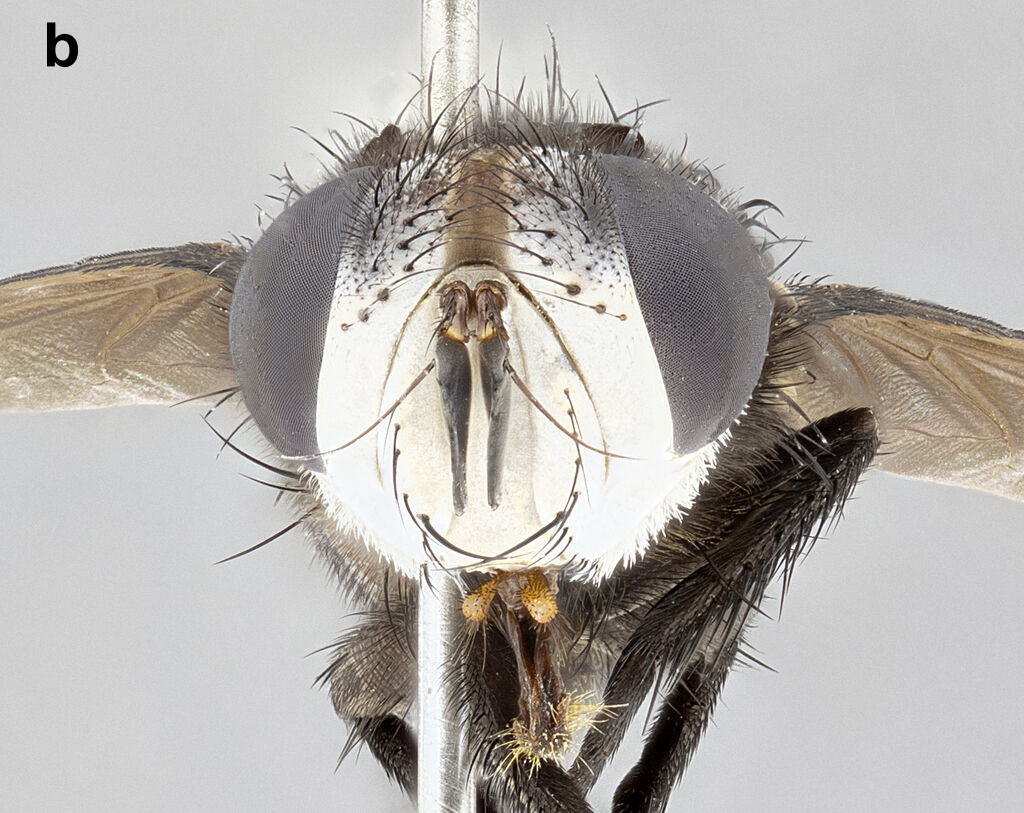
frontal view

**Figure 78c.**
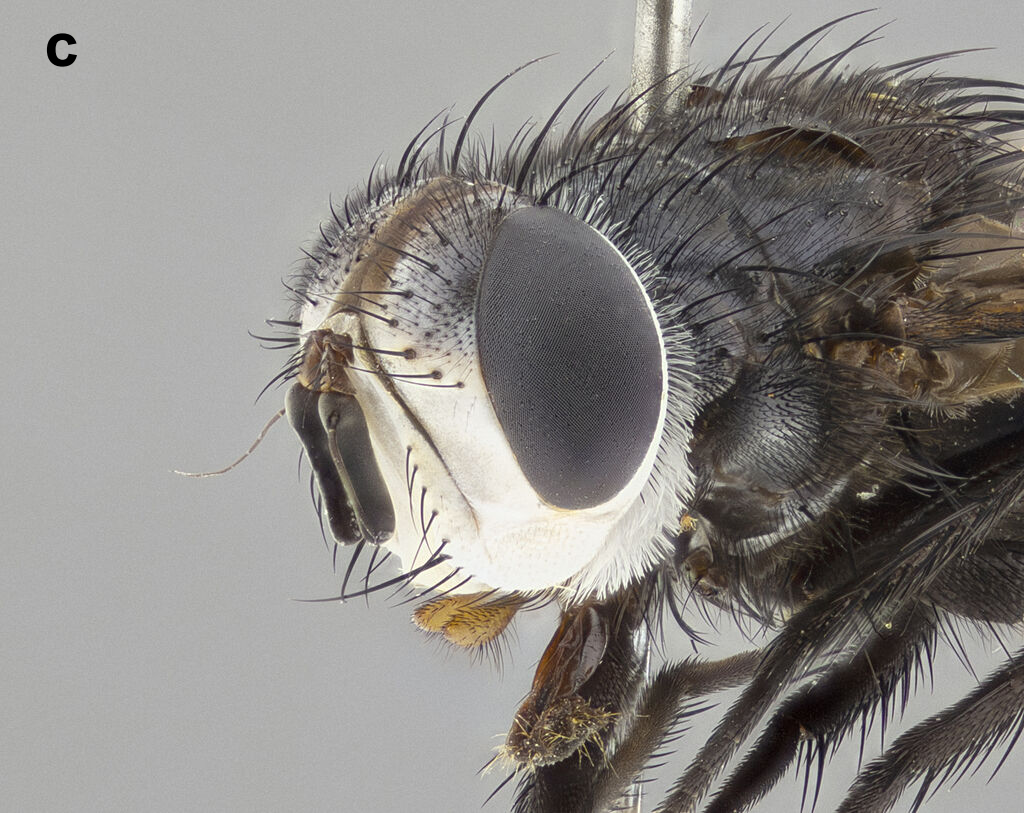
three quarters view

**Figure 78d.**
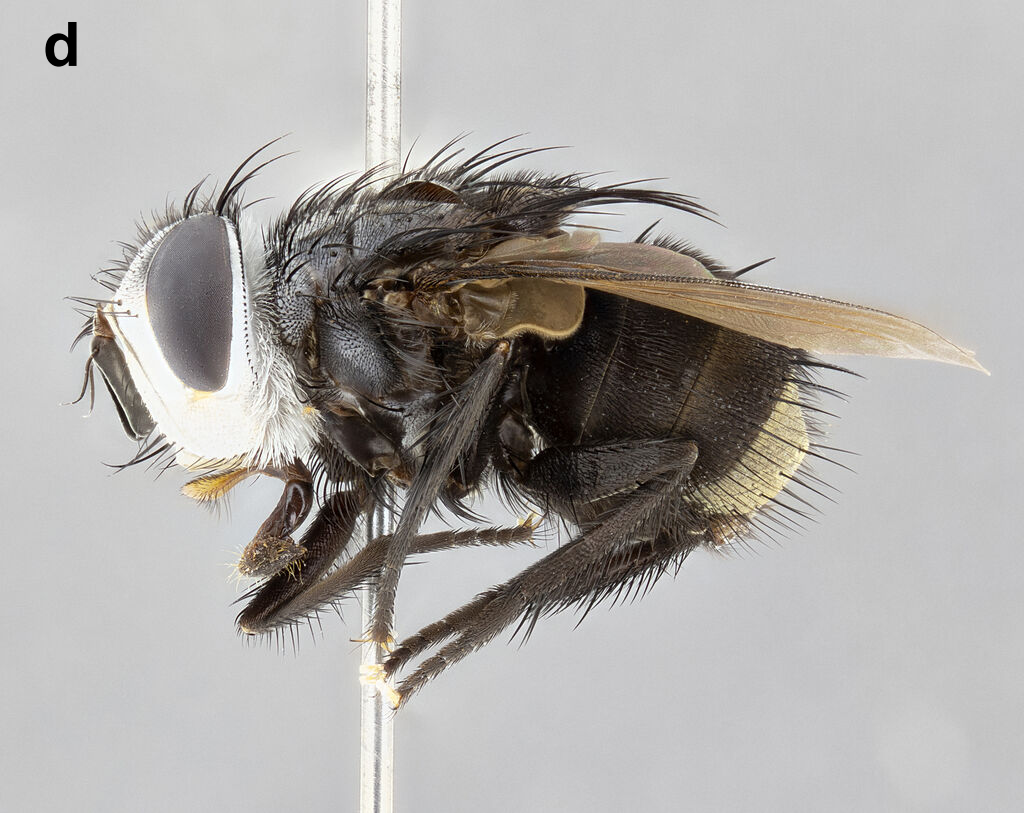
lateral view

**Figure 79a.**
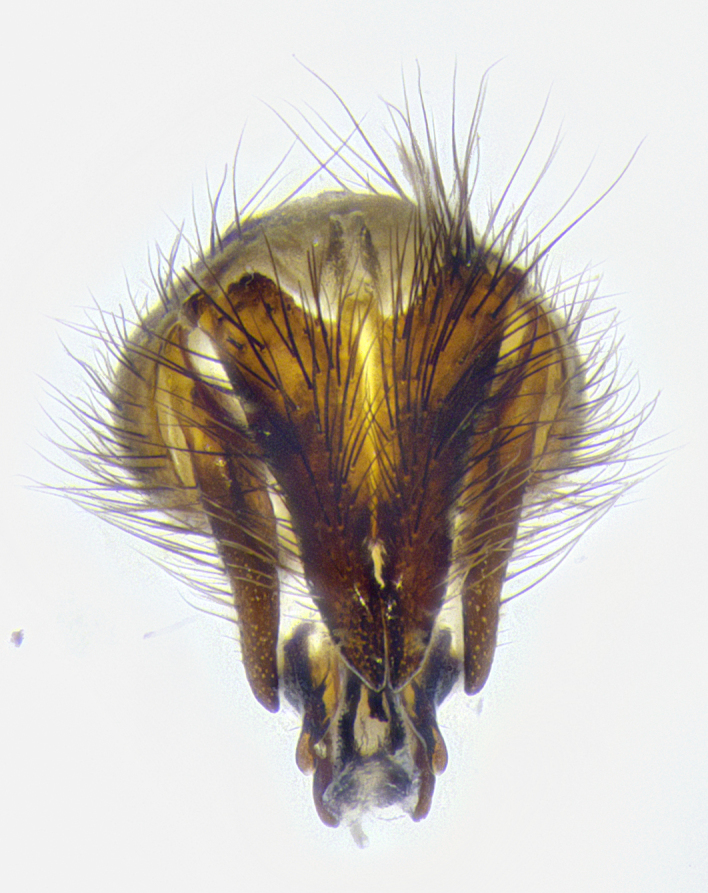
caudal view

**Figure 79b.**
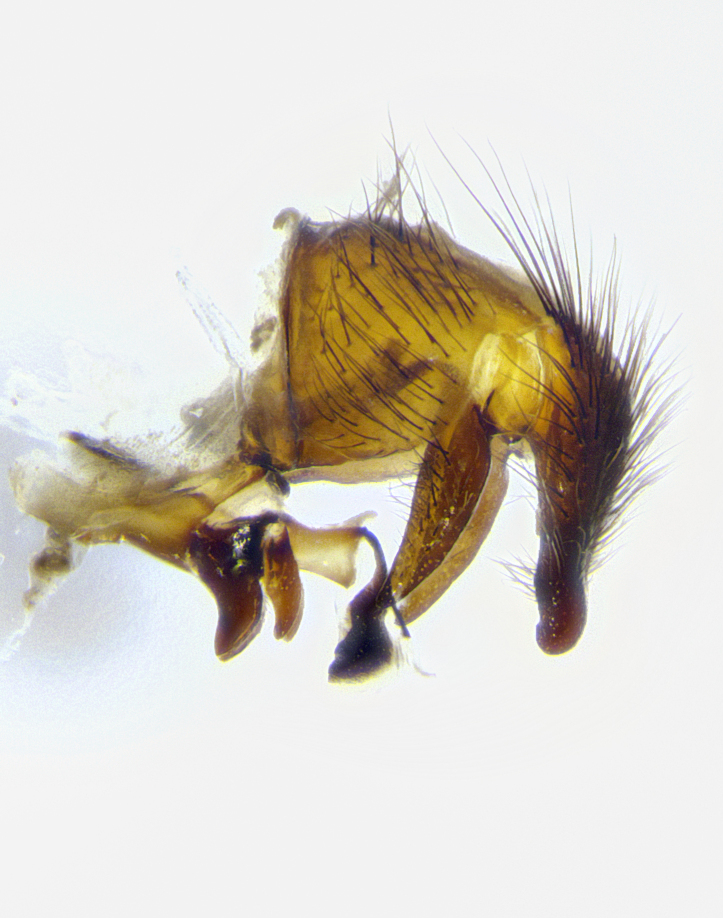
lateral view

**Figure 79c.**
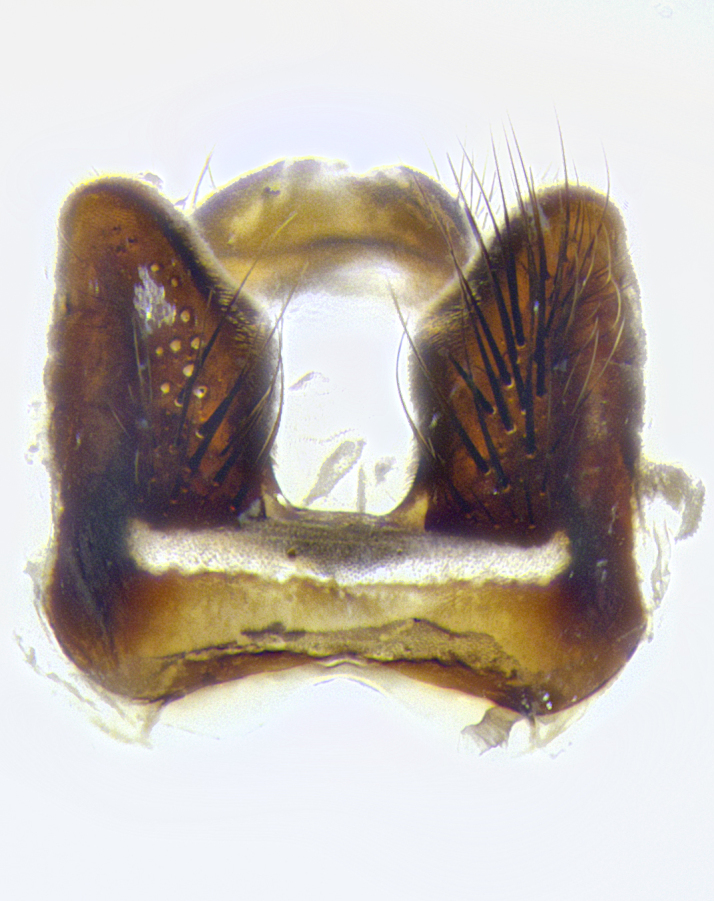
sternite 5, ventral view

**Figure 80a.**
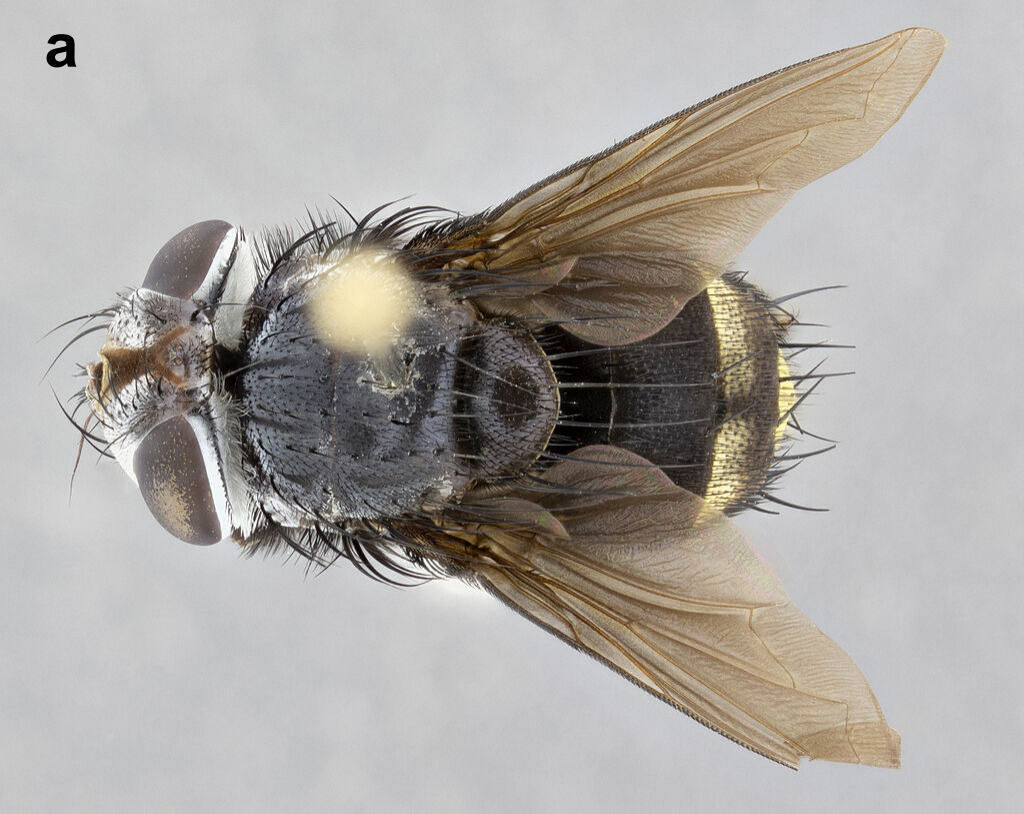
dorsal view

**Figure 80b.**
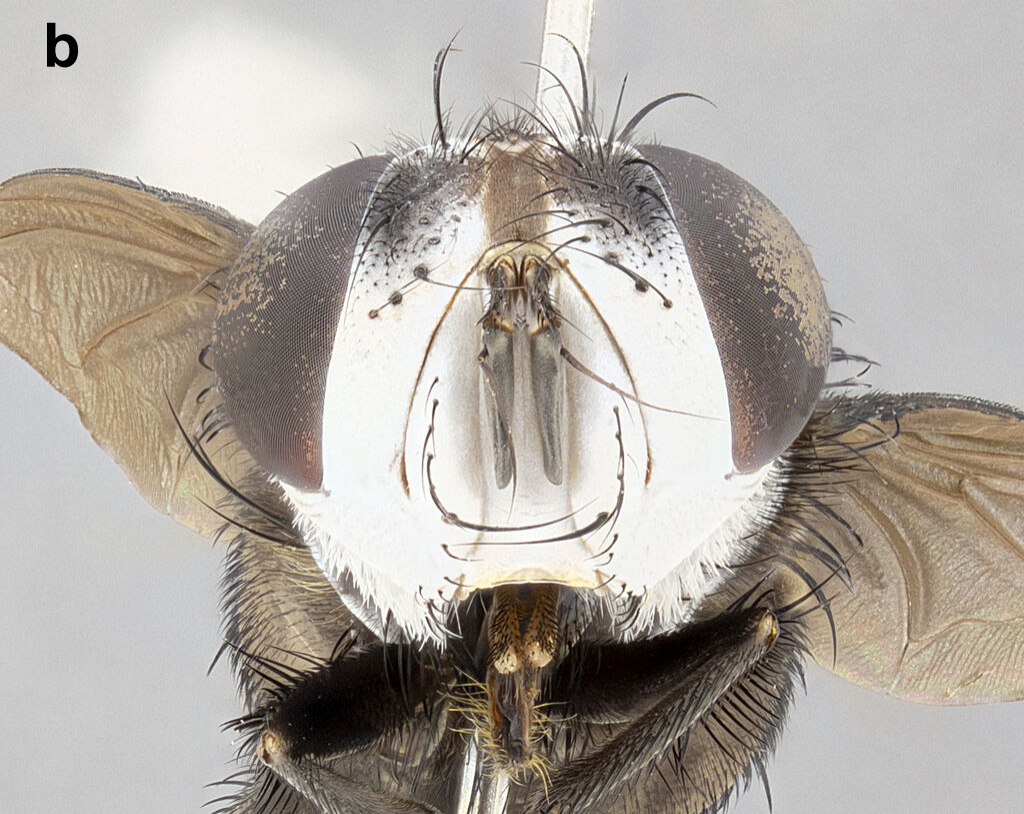
frontal view

**Figure 80c.**
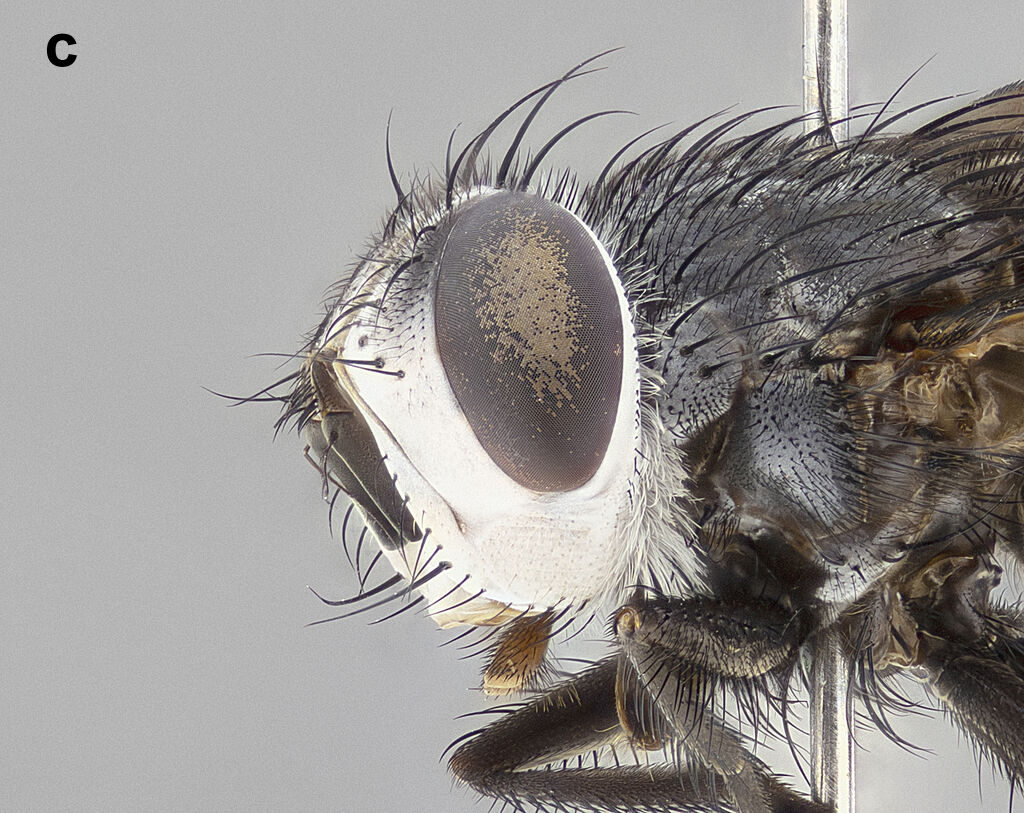
three quarters view

**Figure 80d.**
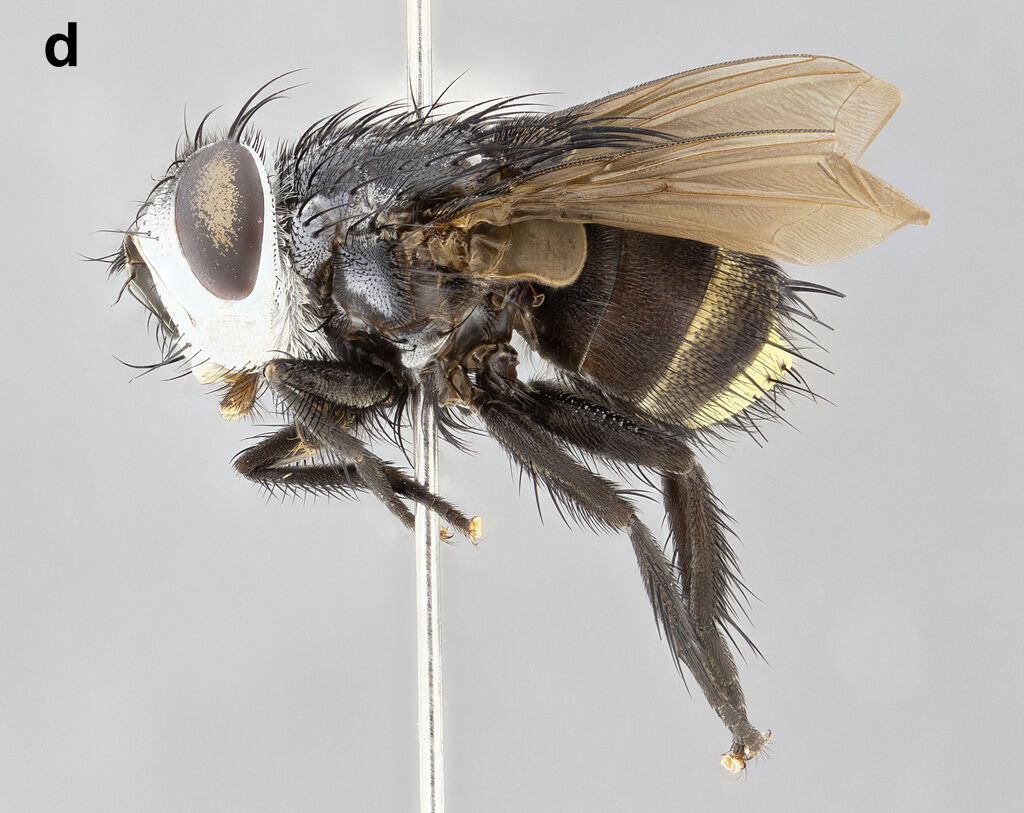
lateral view

**Figure 81a.**
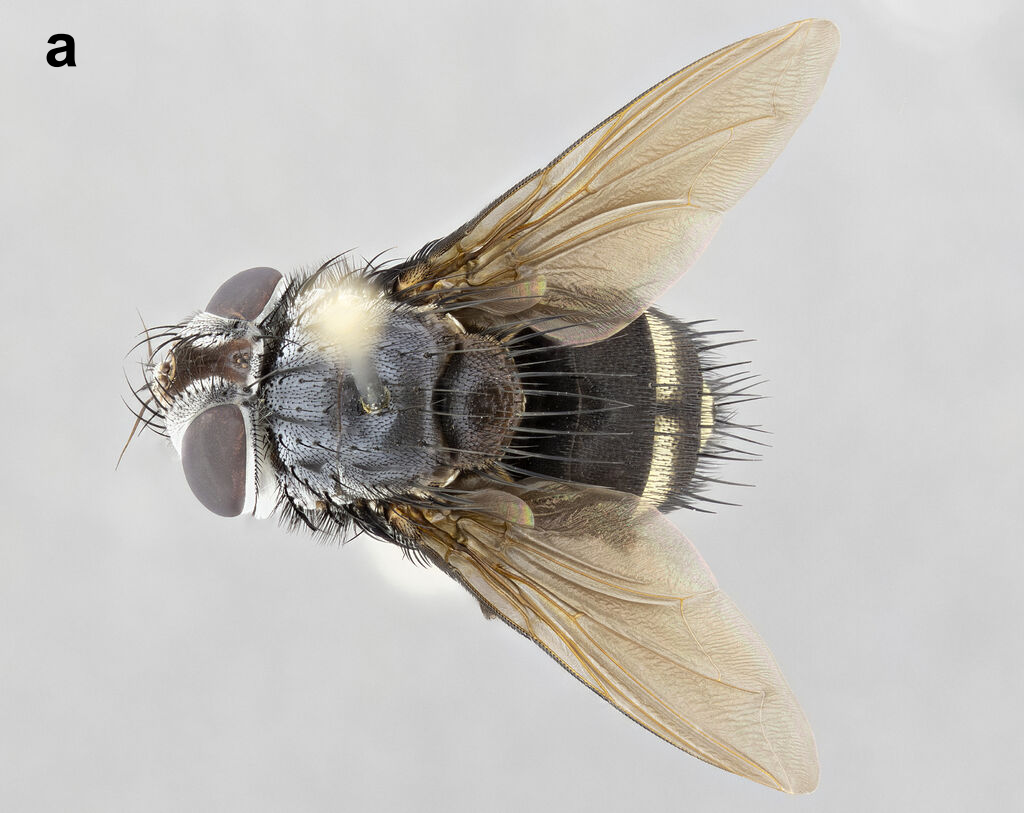
dorsal view

**Figure 81b.**
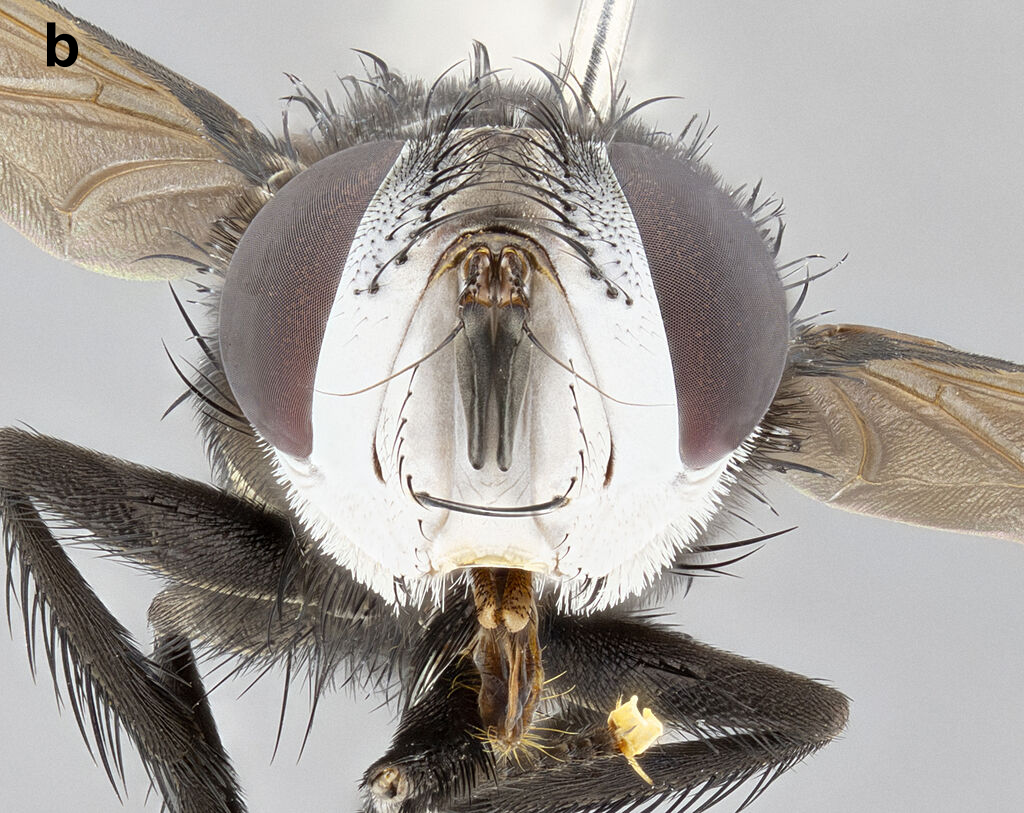
frontal view

**Figure 81c.**
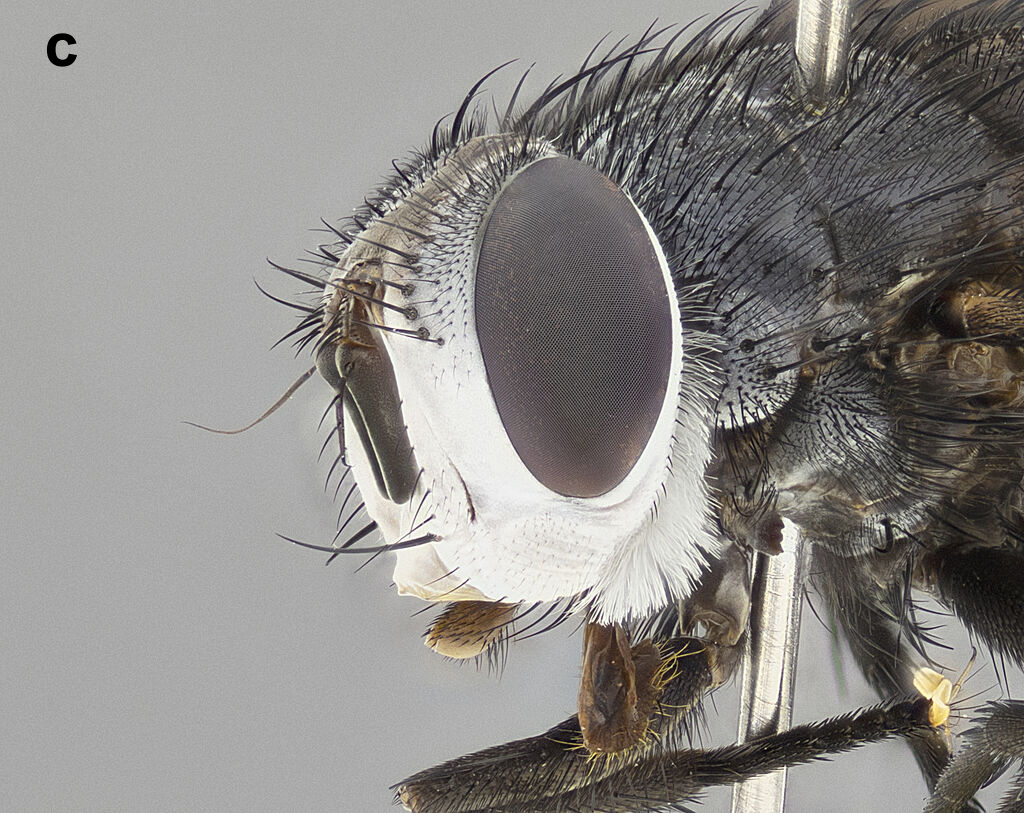
three quarters view

**Figure 81d.**
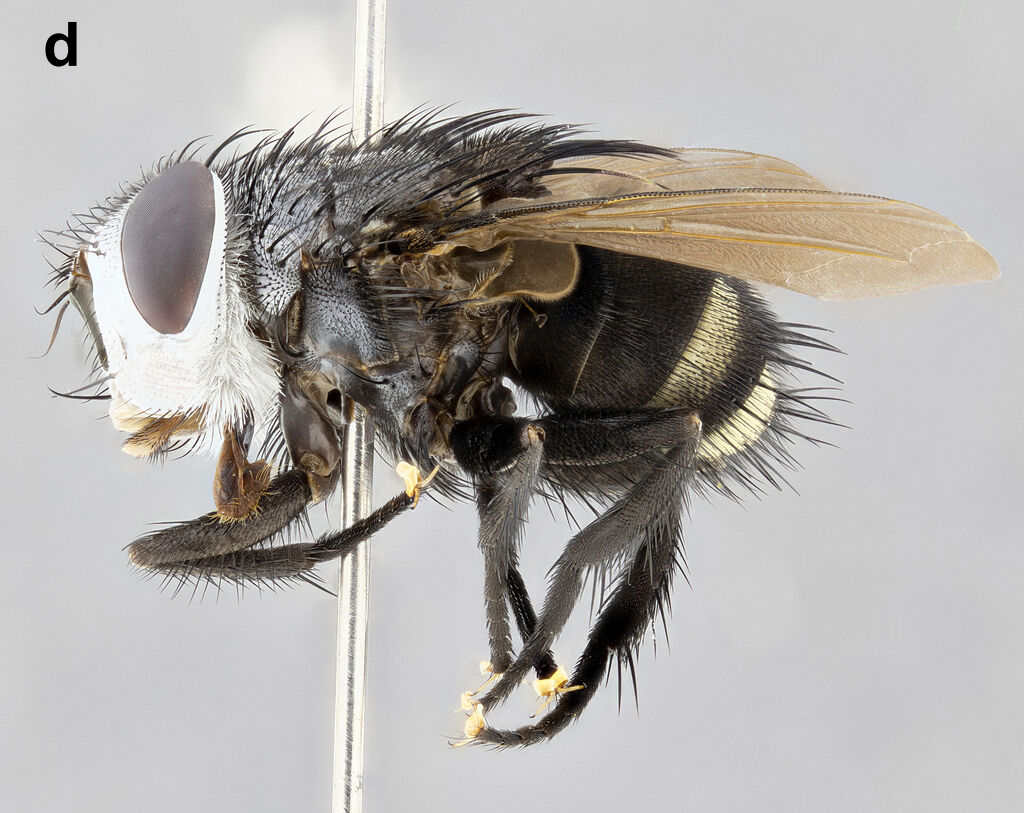
lateral view

**Figure 82a.**
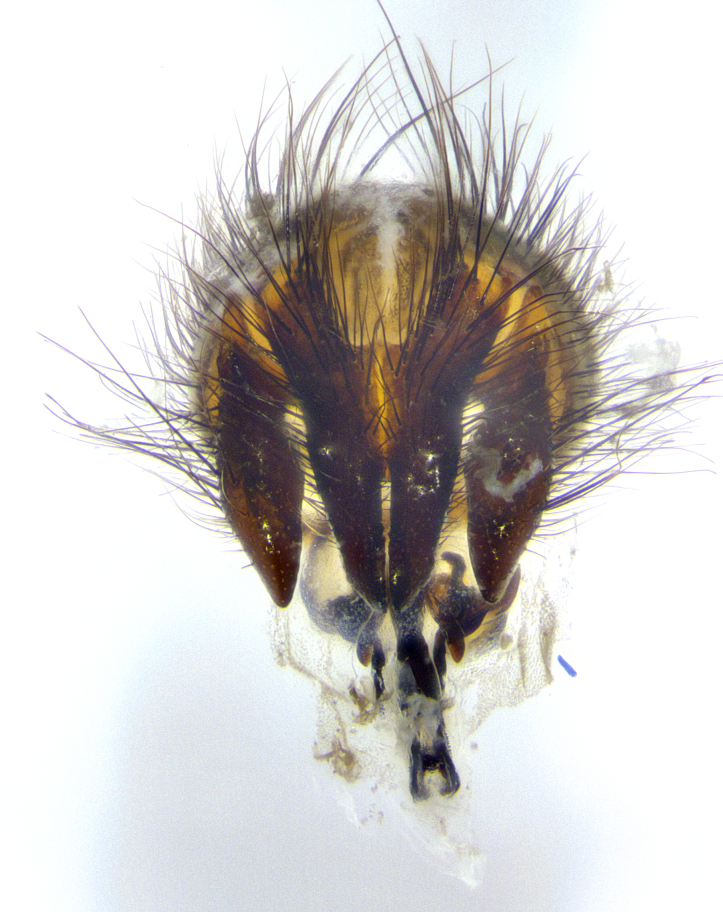
caudal view

**Figure 82b.**
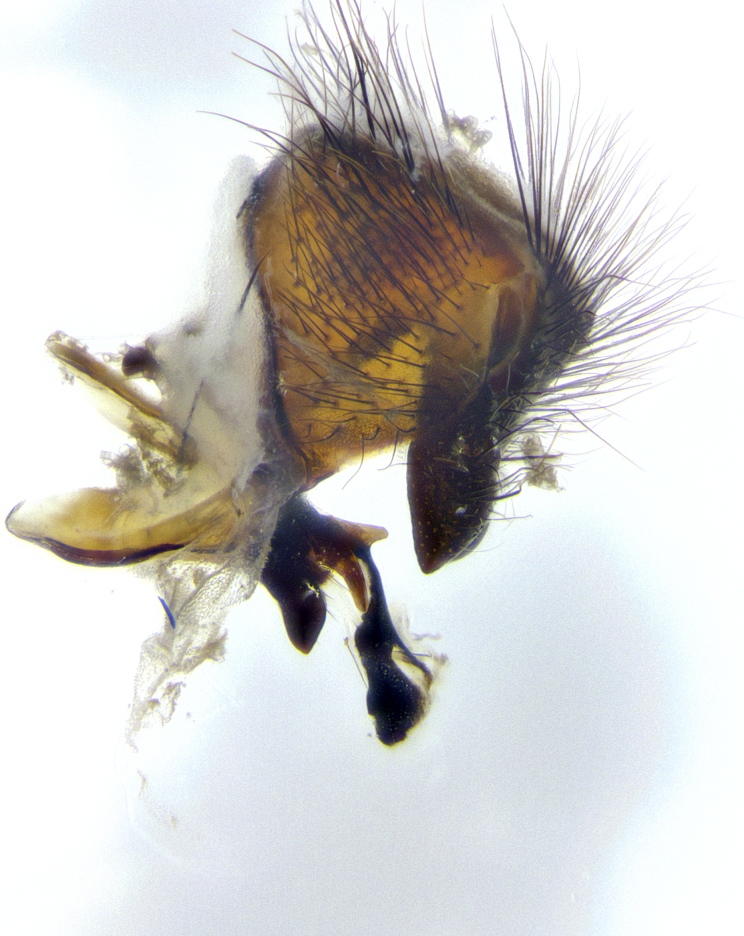
lateral view

**Figure 82c.**
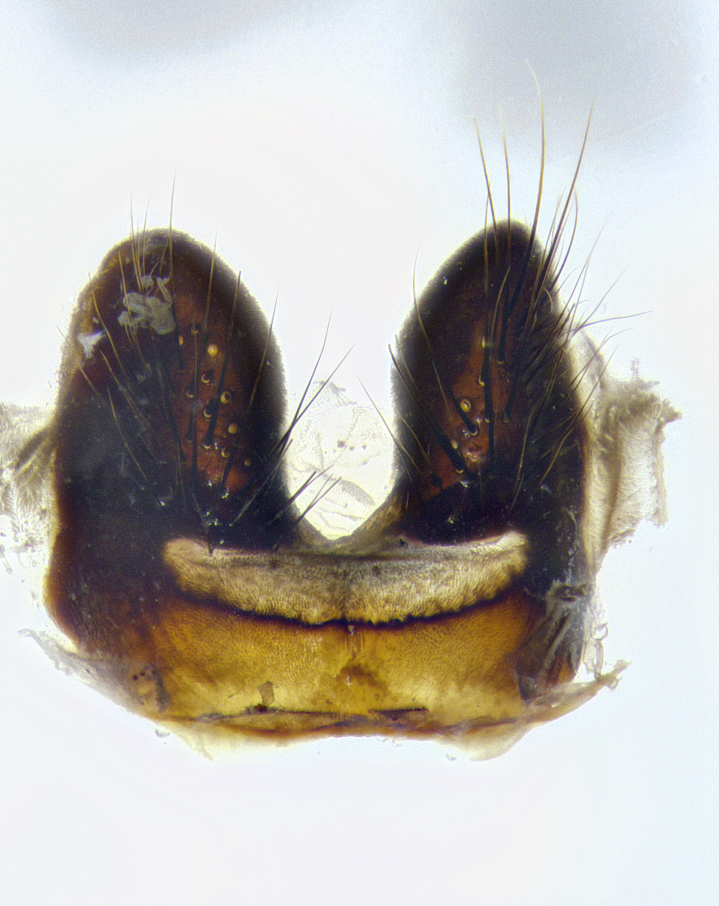
sternite 5, ventral view

**Figure 83a.**
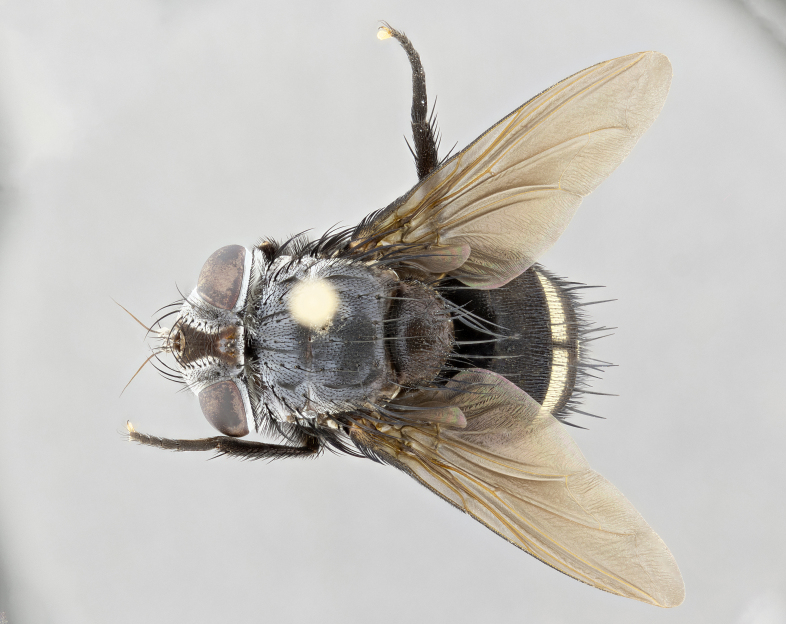
dorsal view

**Figure 83b.**
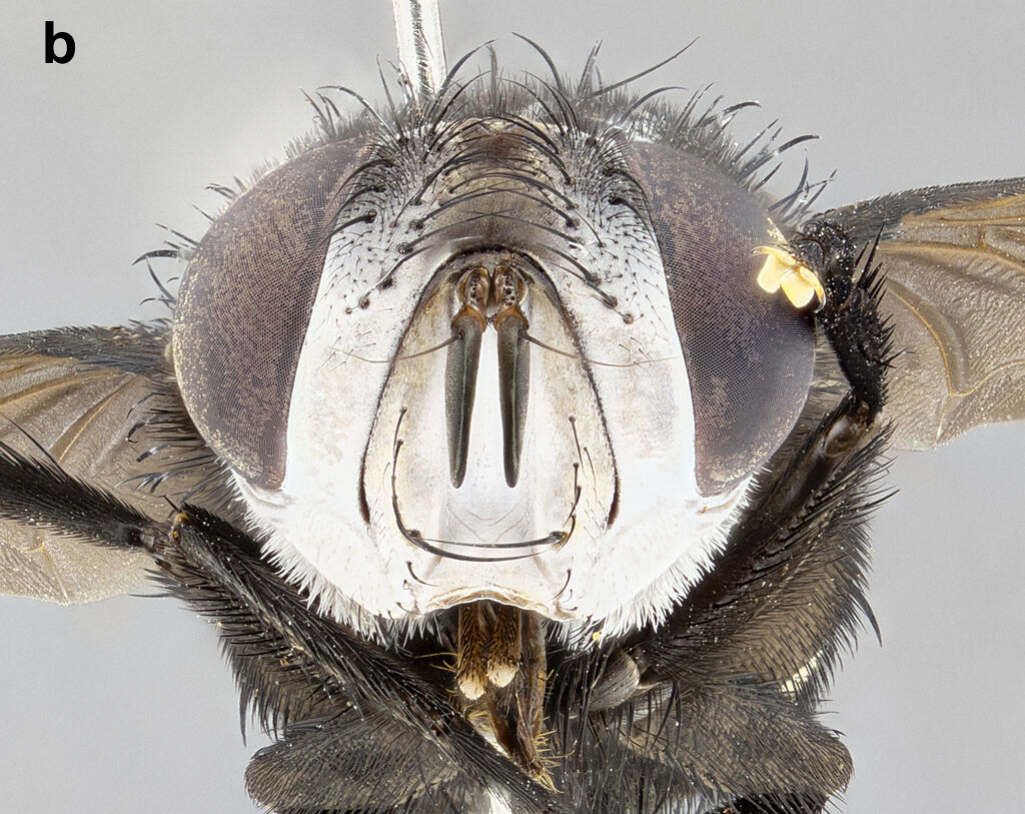
frontal view

**Figure 83c.**
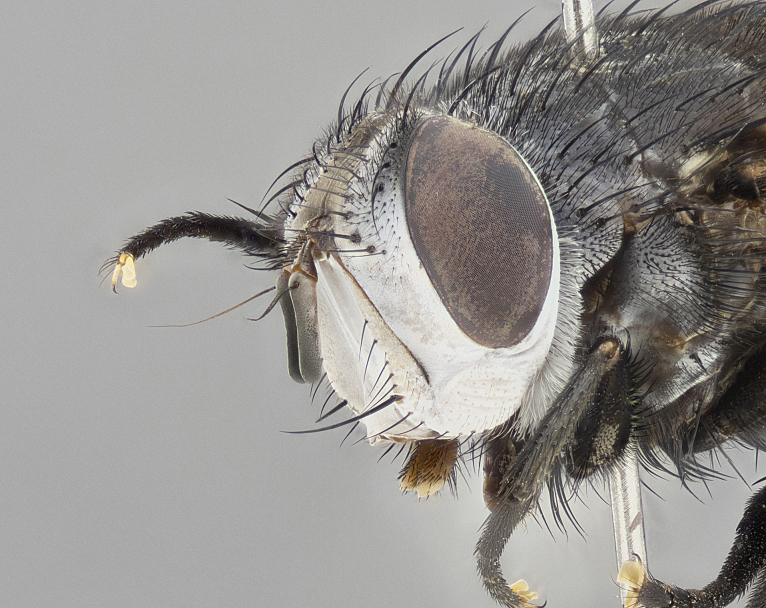
three quarters view

**Figure 83d.**
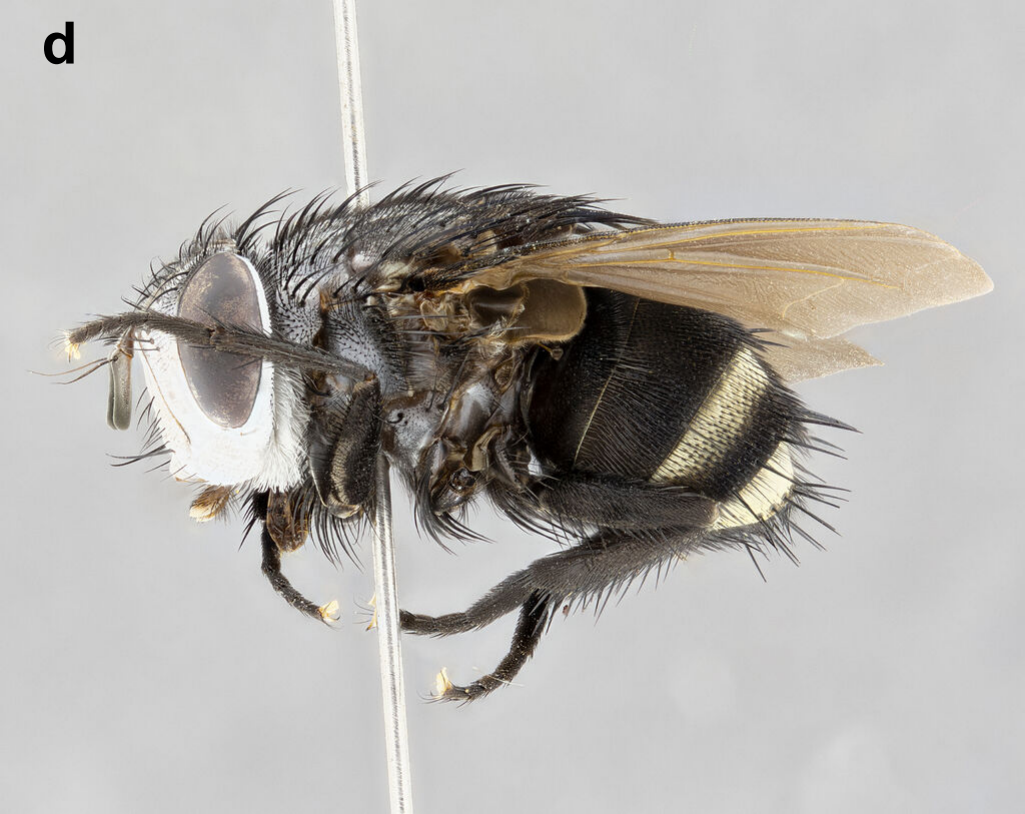
lateral view

**Figure 84a.**
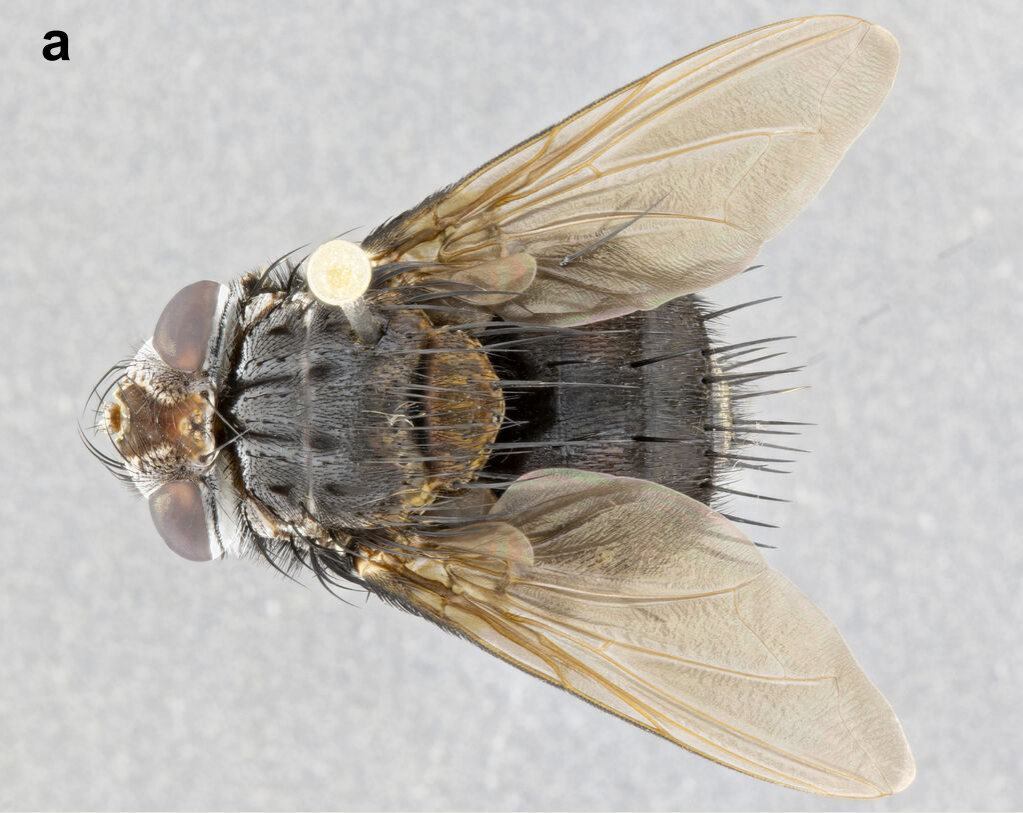
dorsal view

**Figure 84b.**
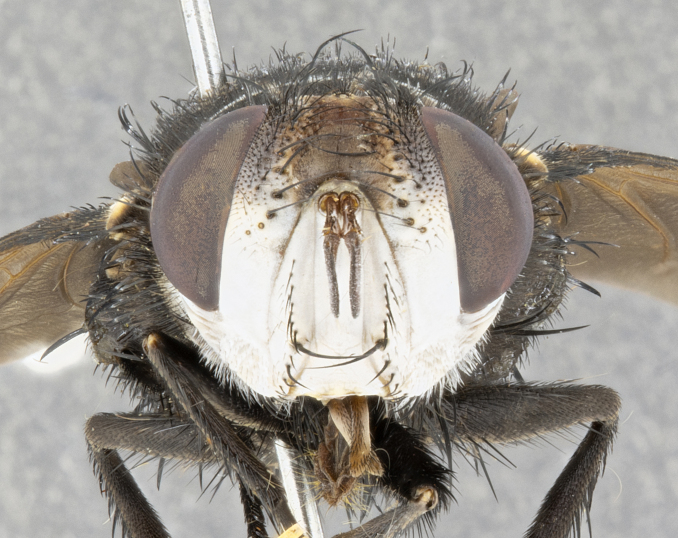
frontal view

**Figure 84c.**
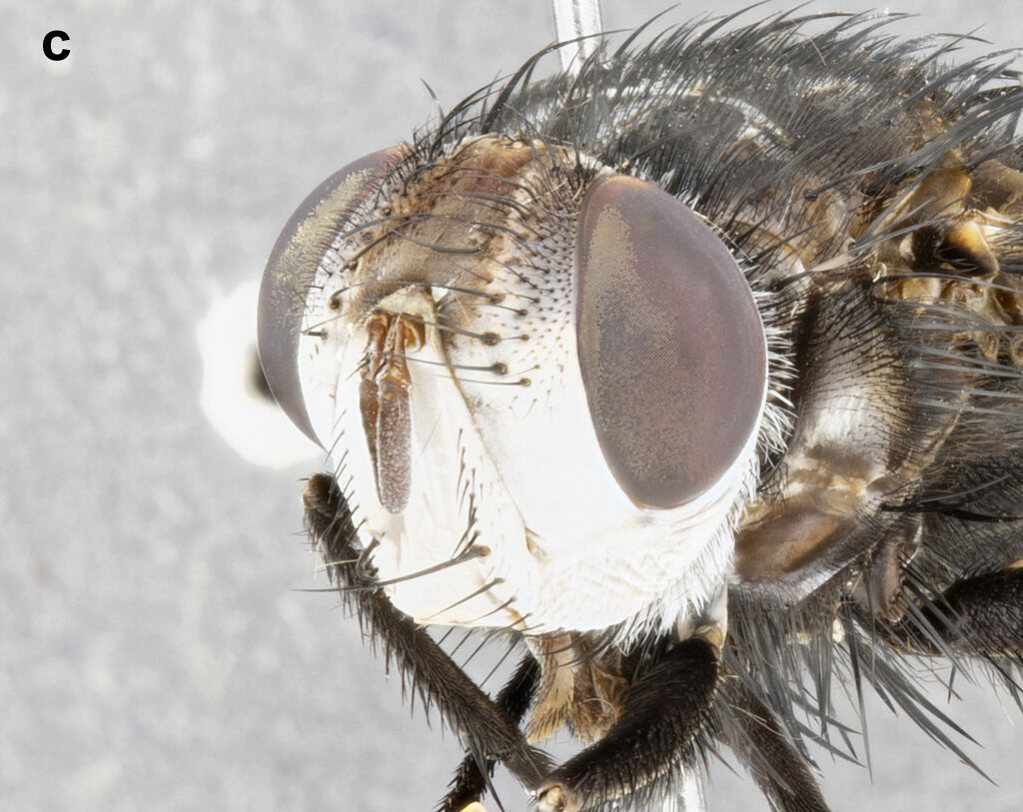
three quarters view

**Figure 84d.**
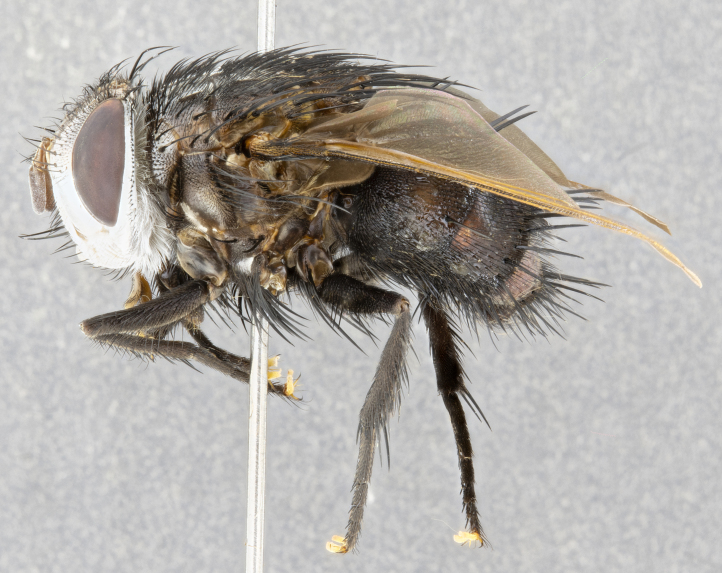
lateral view

**Figure 85a.**
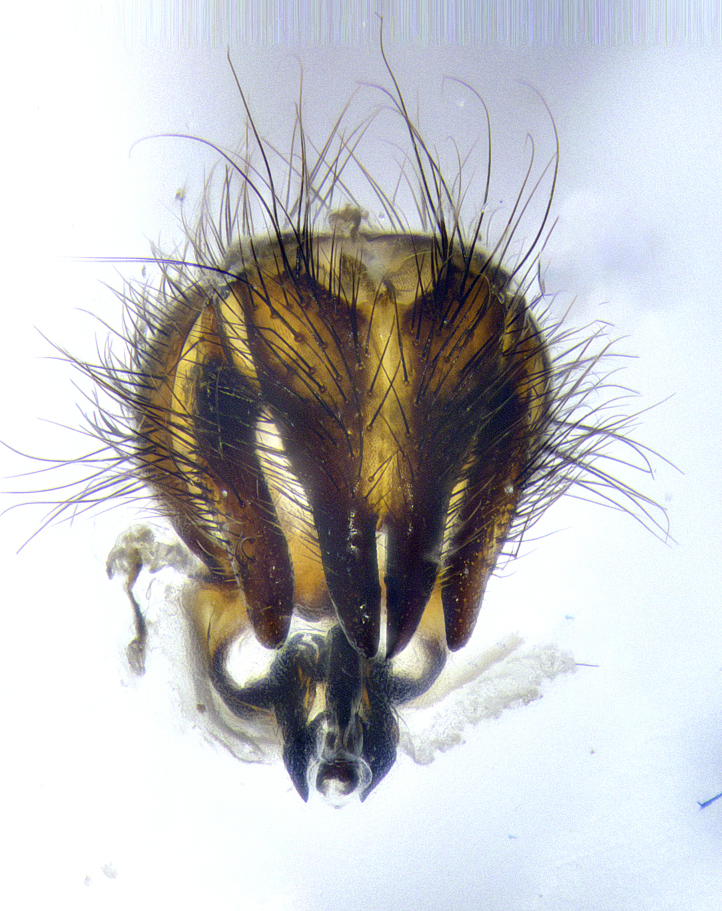
caudal view

**Figure 85b.**
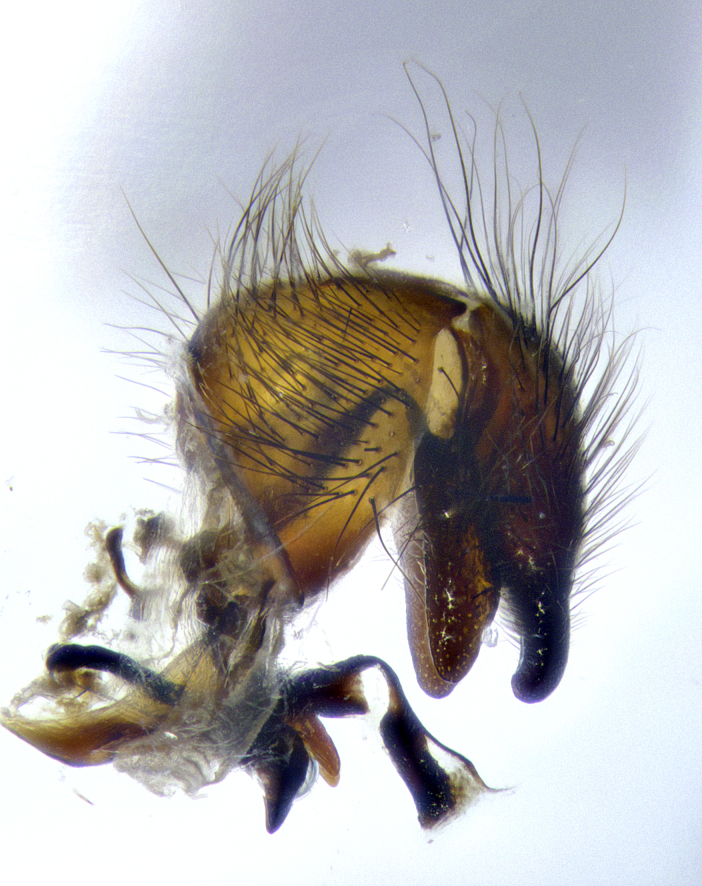
lateral view

**Figure 85c.**
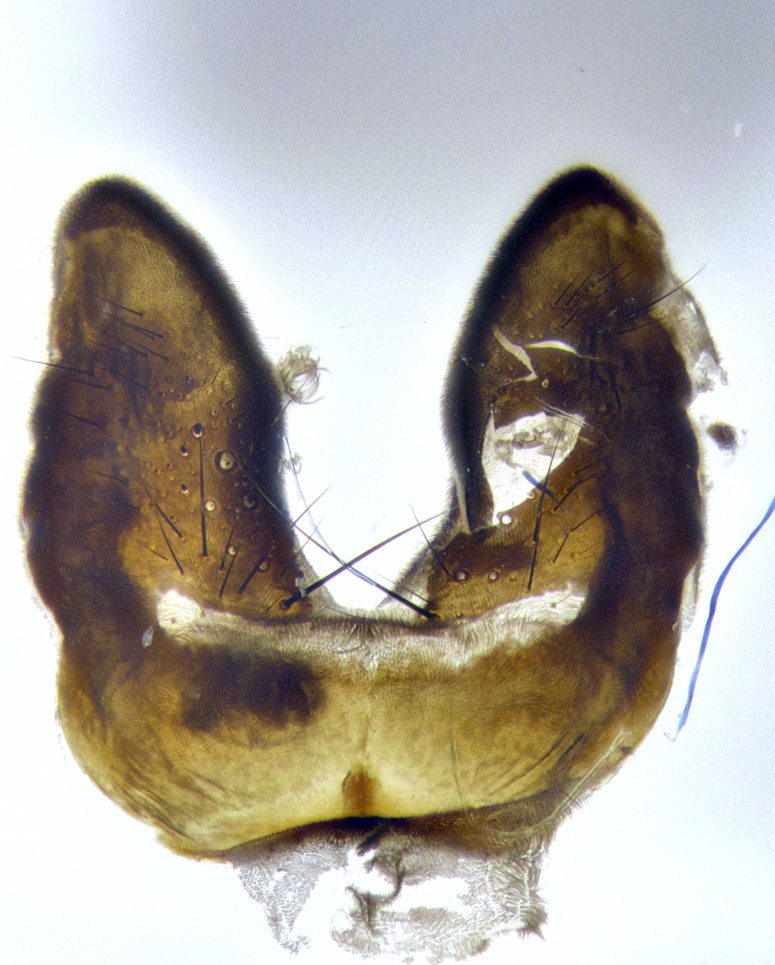
sternite 5, ventral view

**Figure 86a.**
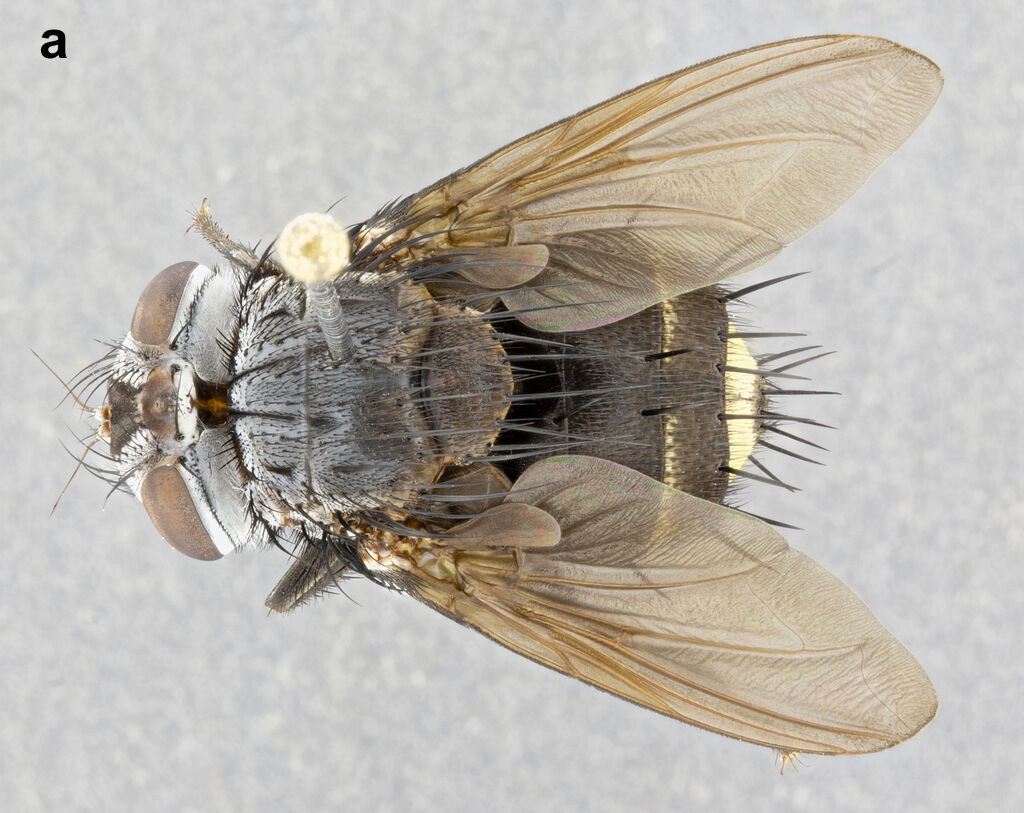
dorsal view

**Figure 86b.**
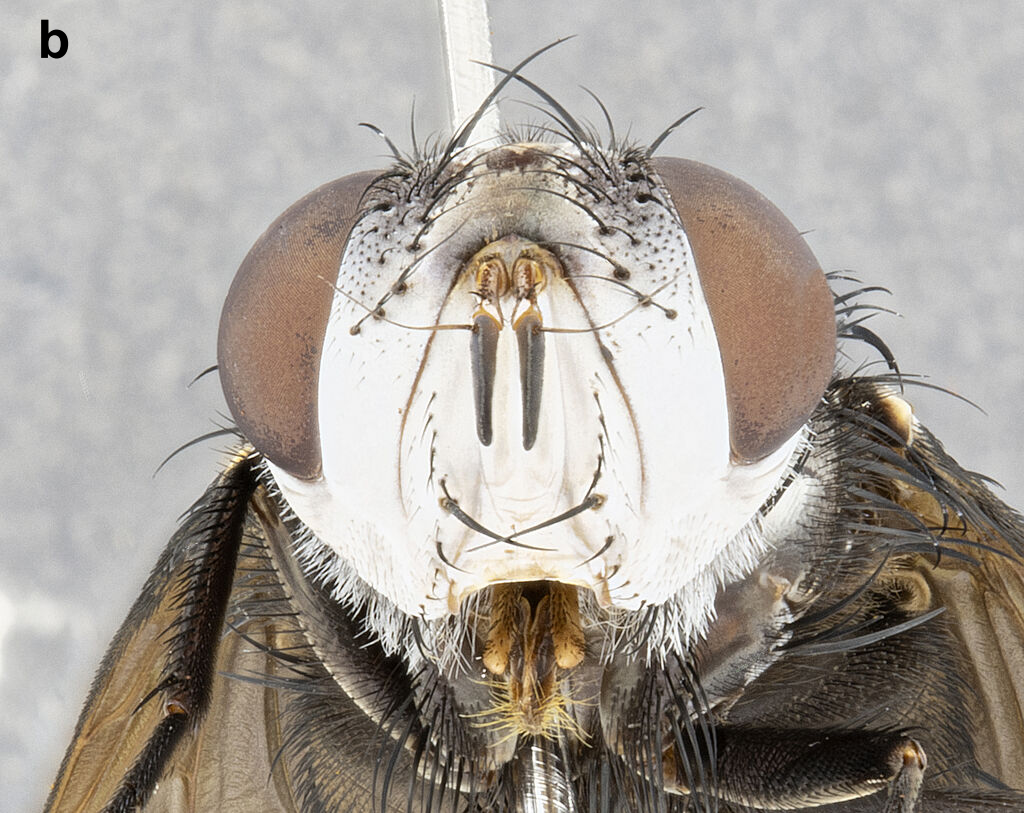
frontal view

**Figure 86c.**
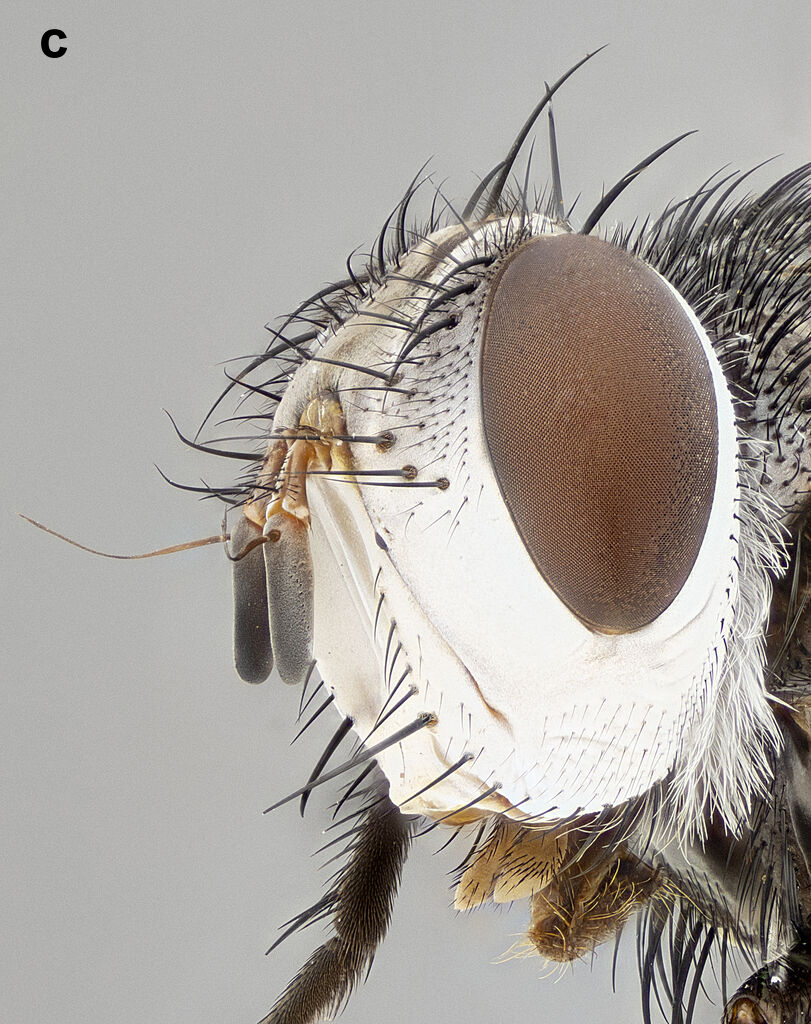
three quarters view

**Figure 86d.**
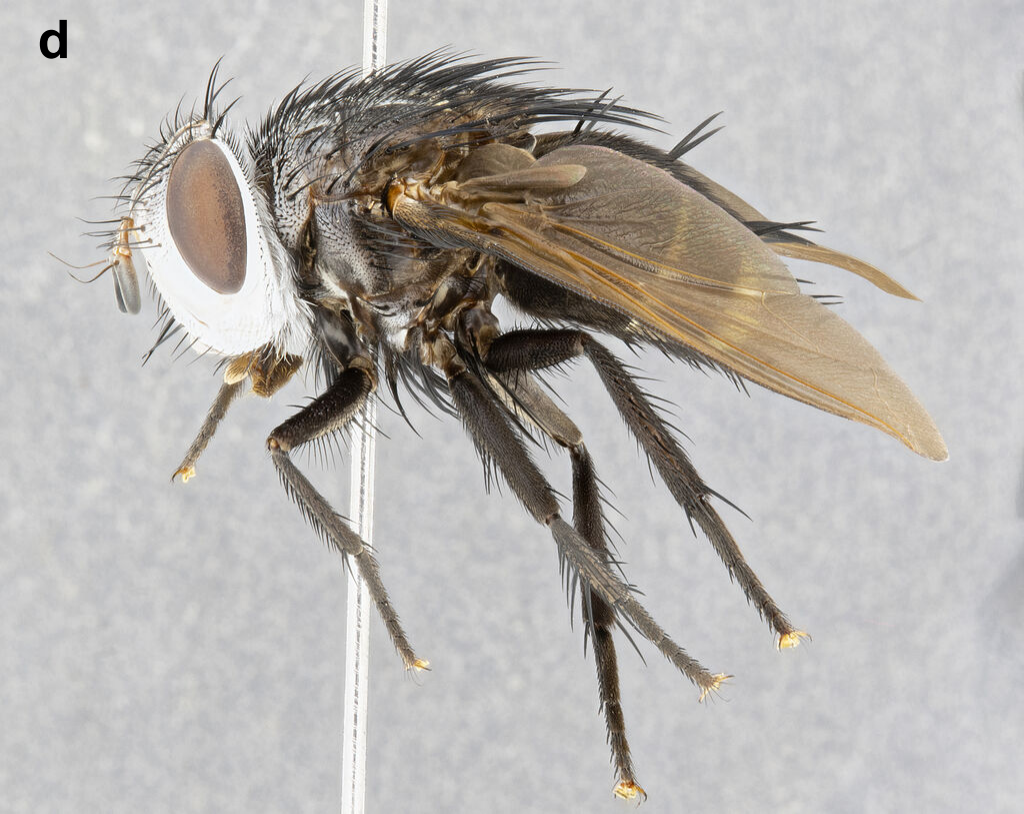
lateral view

**Figure 87a.**
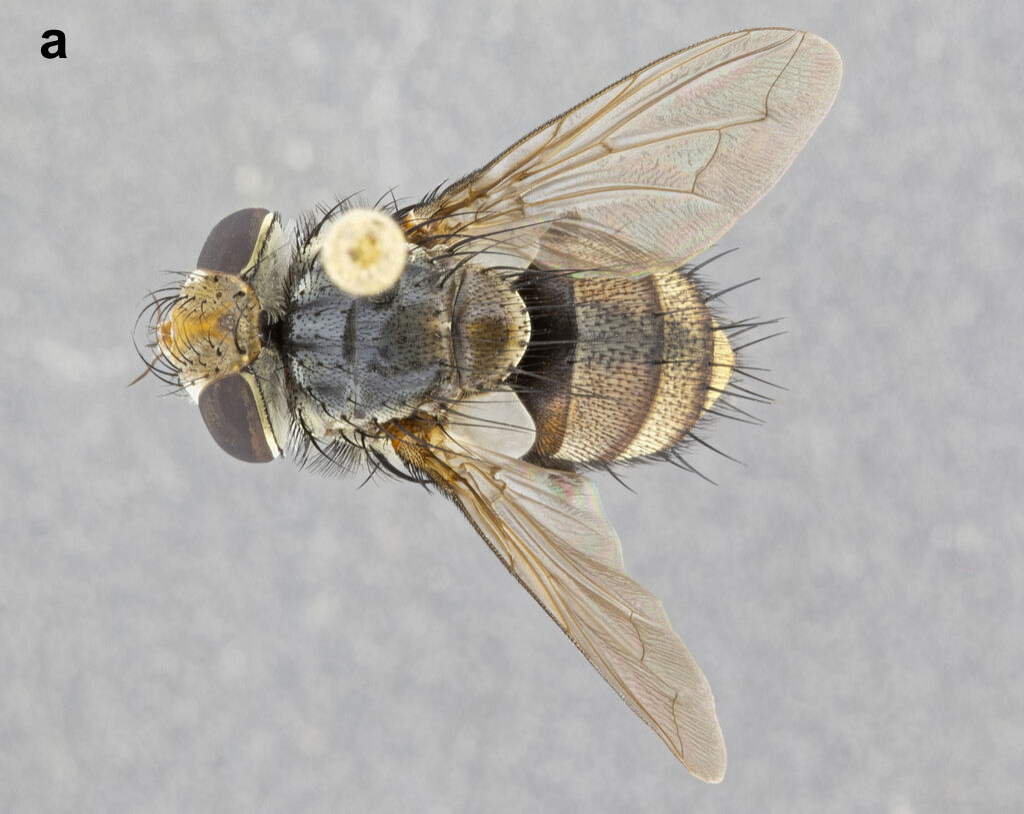
dorsal view

**Figure 87b.**
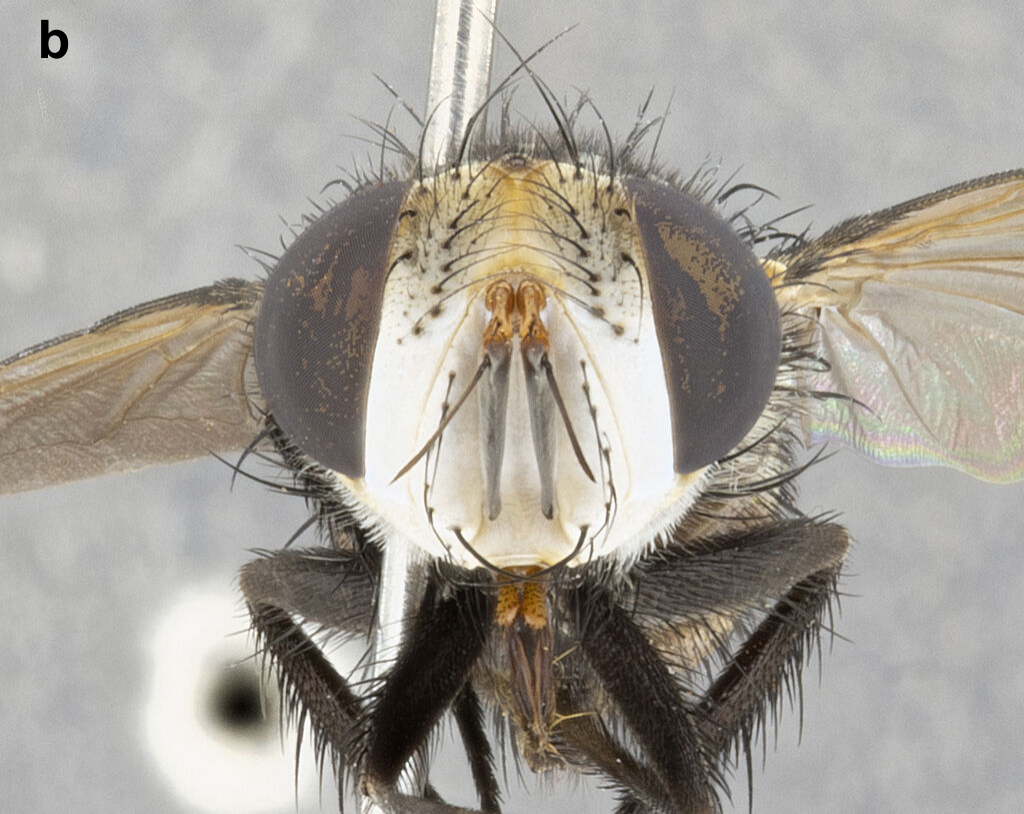
frontal view

**Figure 87c.**
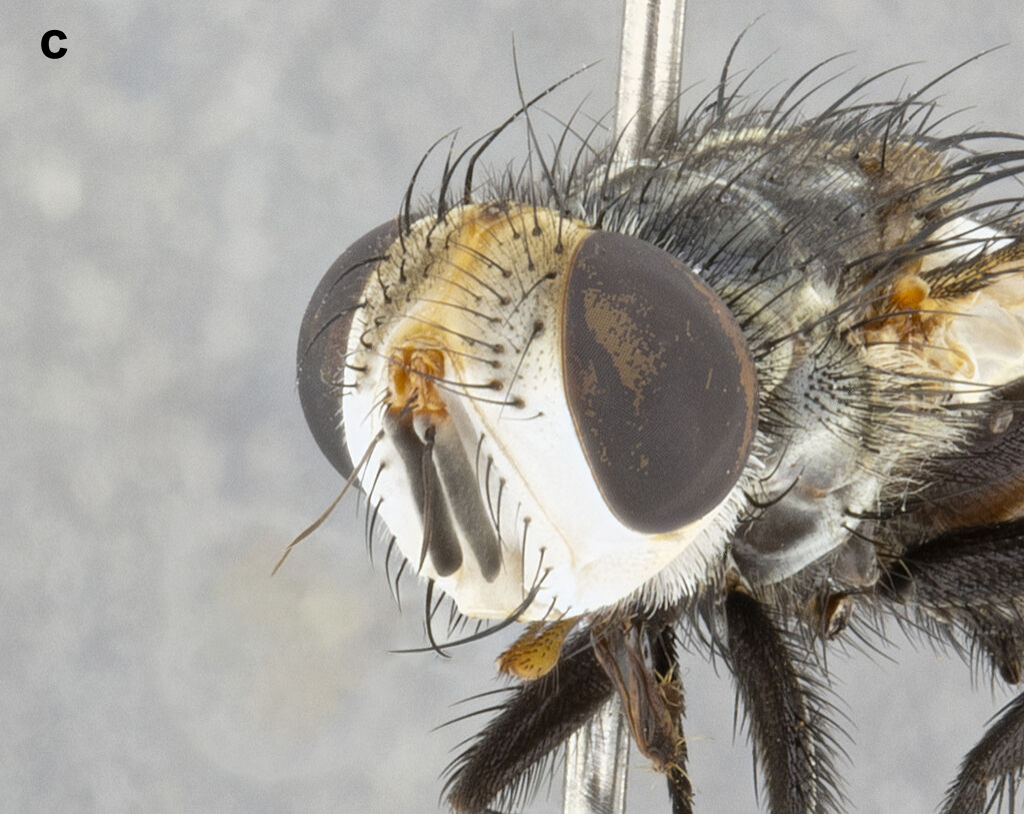
three quarters view

**Figure 87d.**
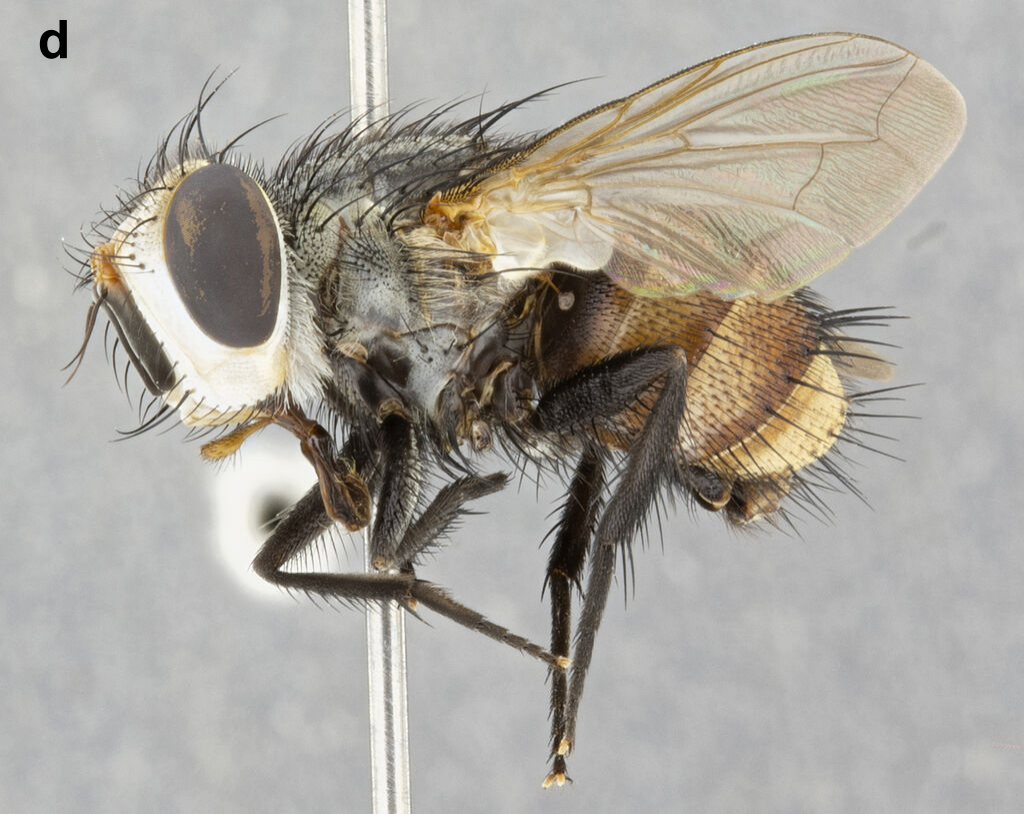
lateral view

**Figure 88a.**
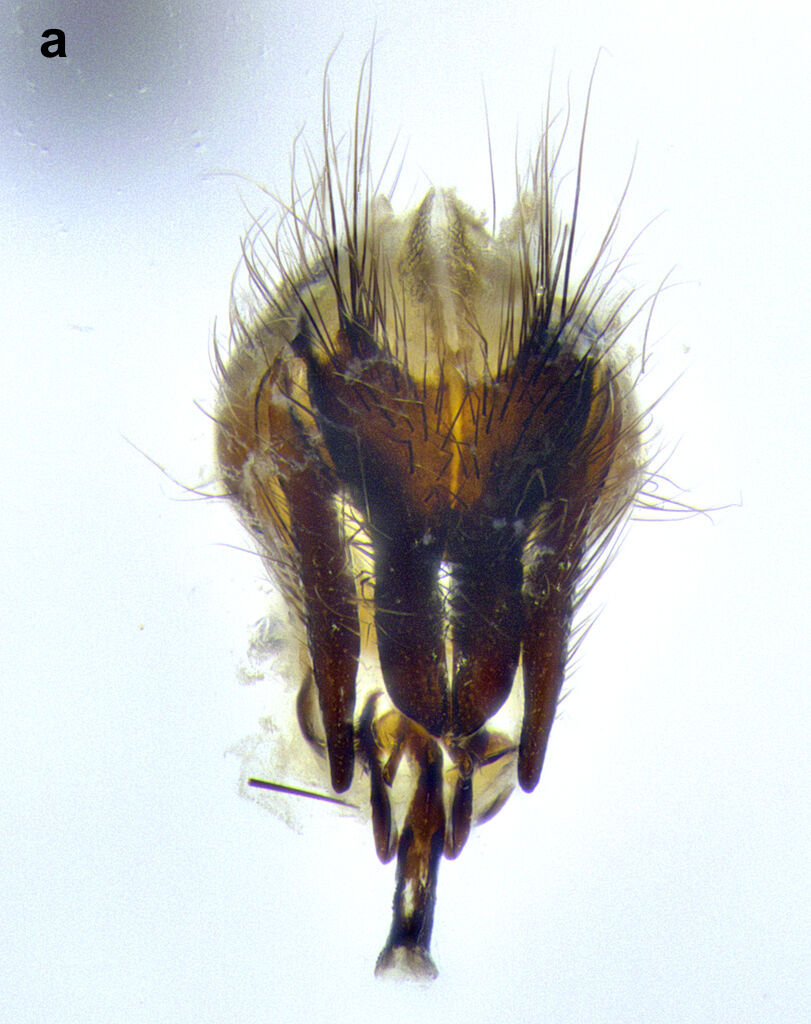
caudal view

**Figure 88b.**
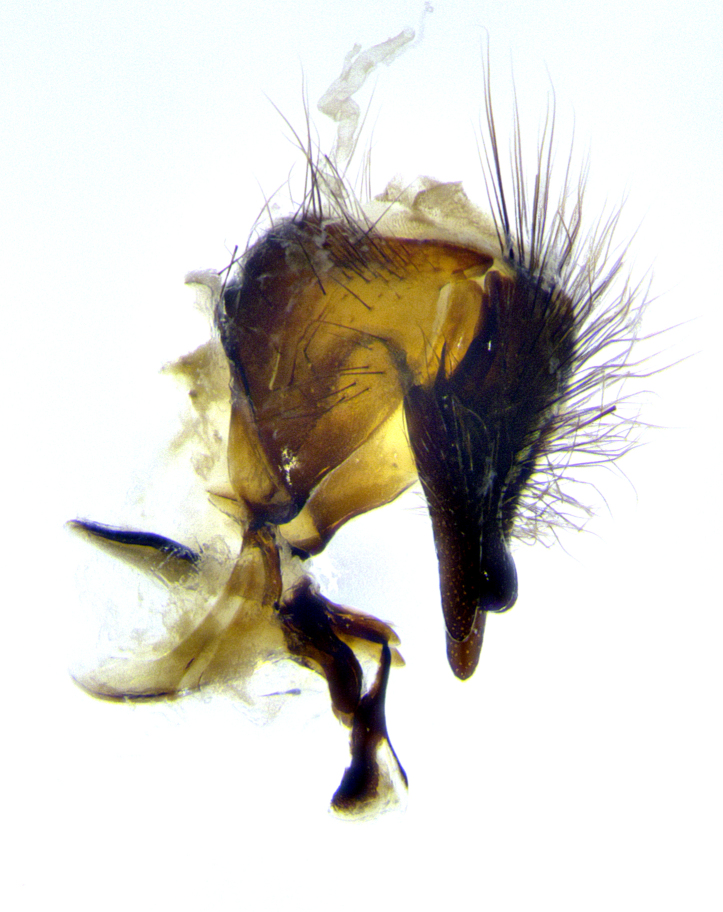
lateral view

**Figure 88c.**
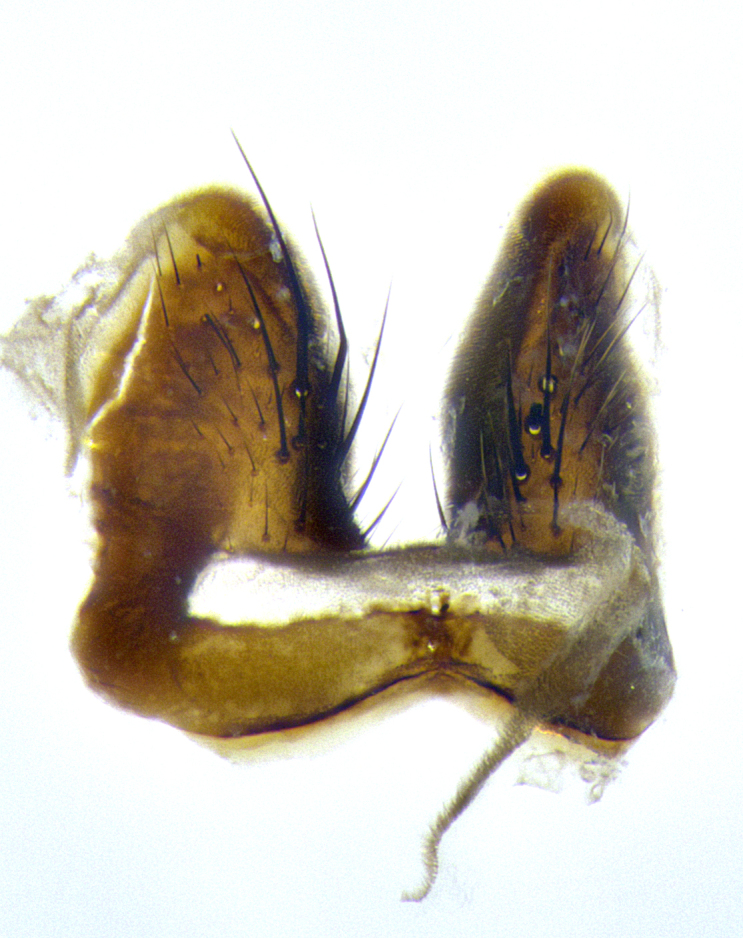
sternite 5, ventral view

**Figure 89a.**
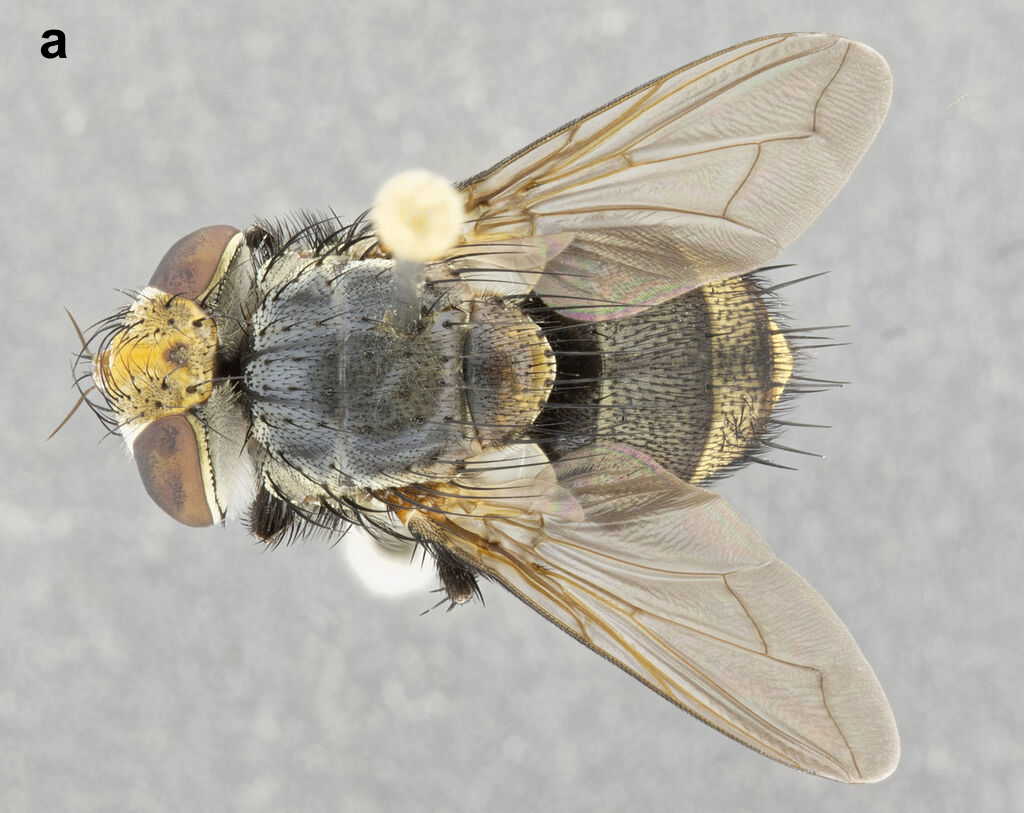
dorsal view

**Figure 89b.**
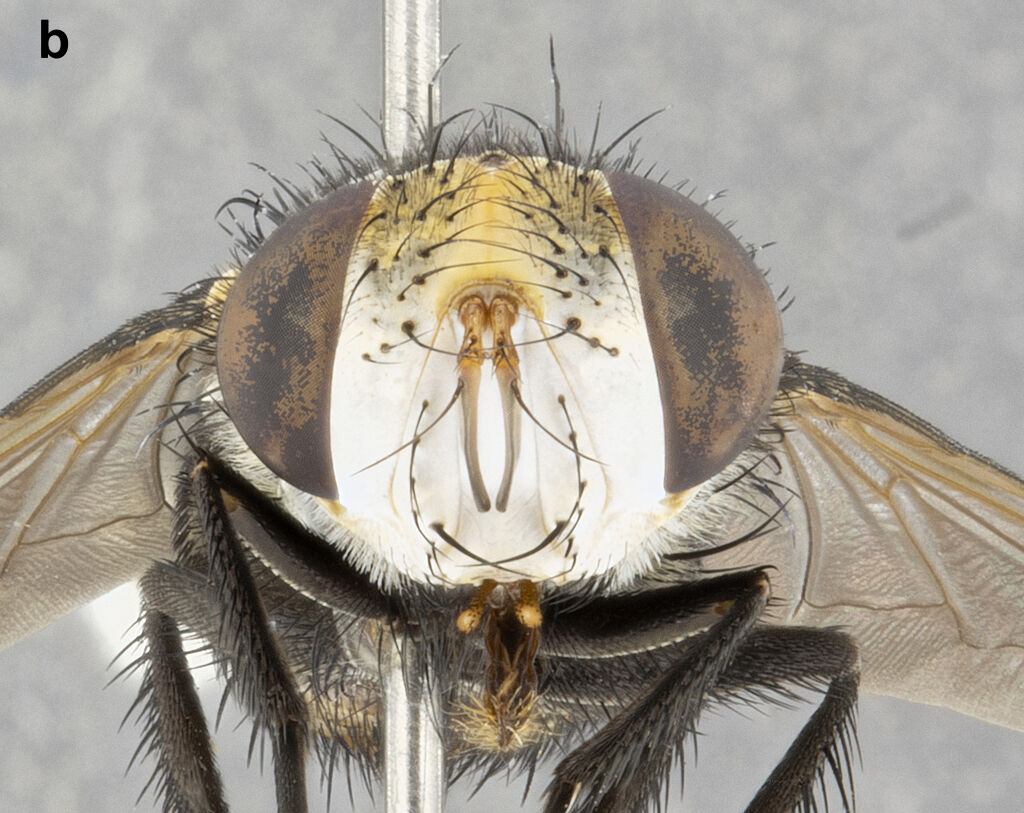
frontal view

**Figure 89c.**
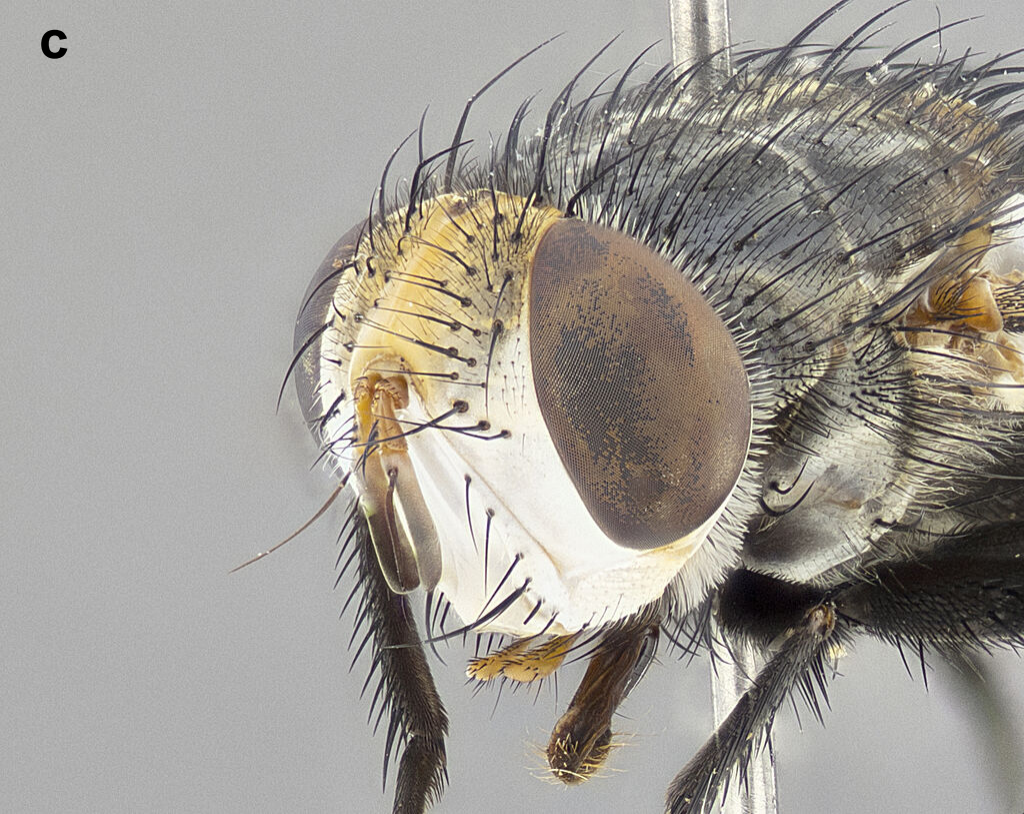
three quarters view

**Figure 89d.**
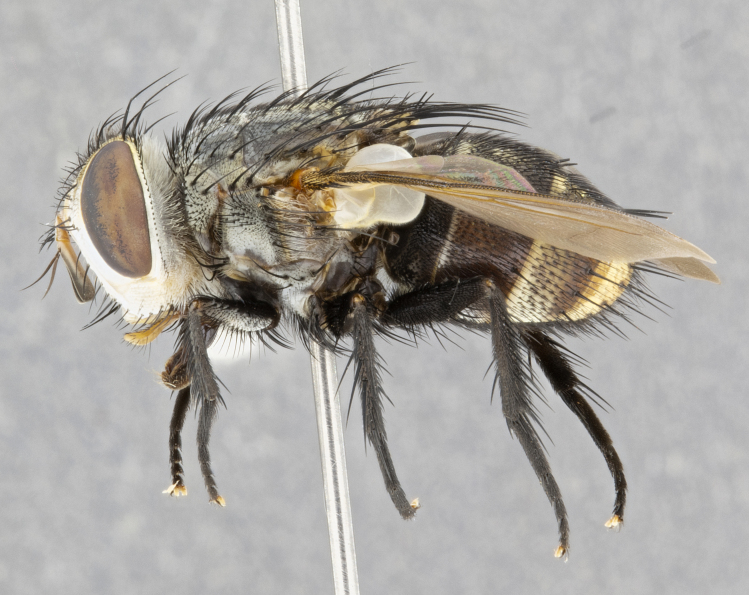
lateral view

**Figure 90a.**
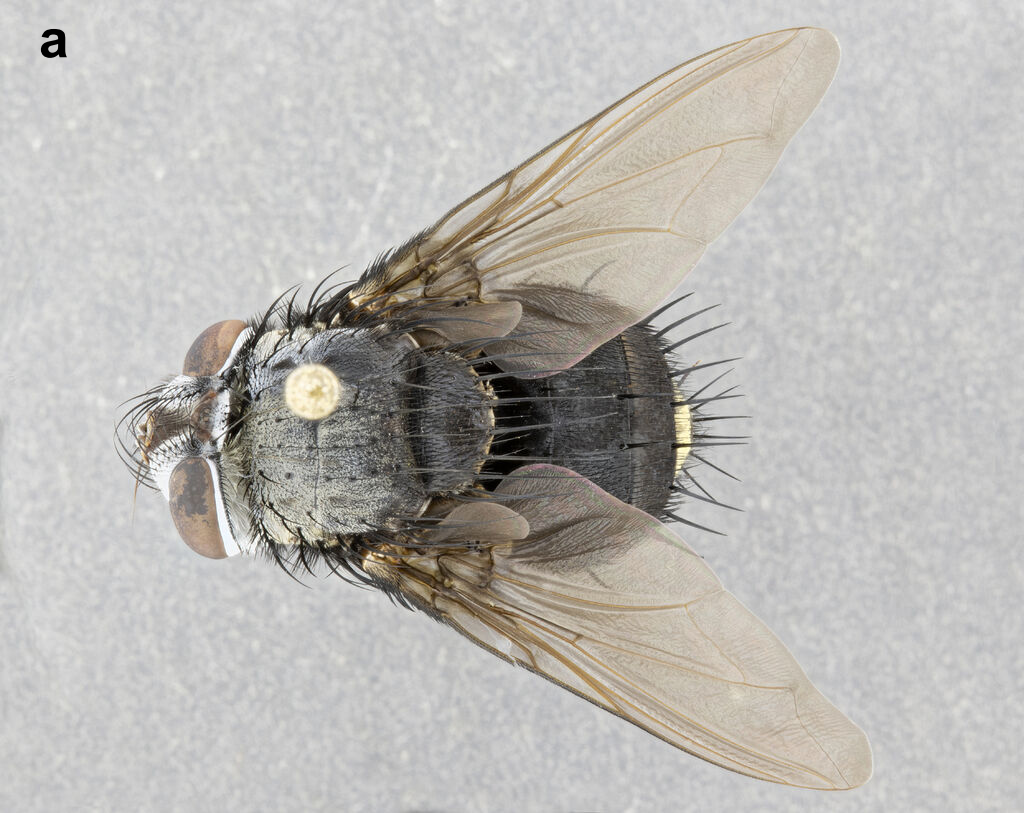
dorsal view

**Figure 90b.**
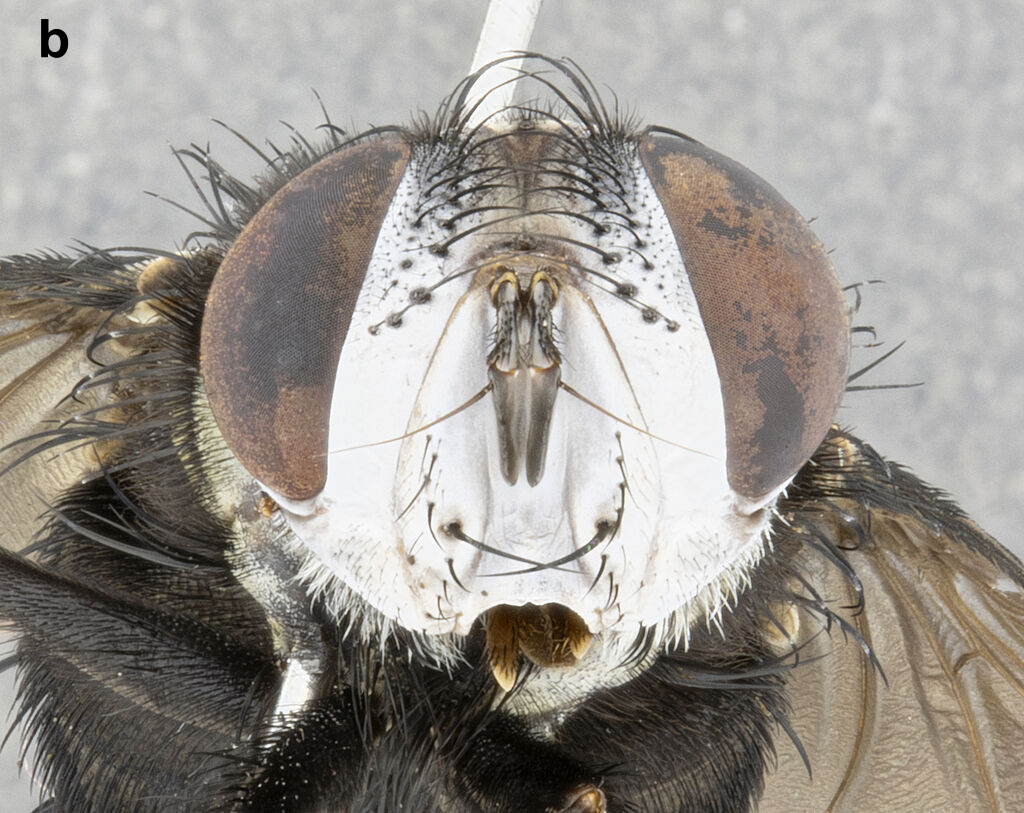
frontal view

**Figure 90c.**
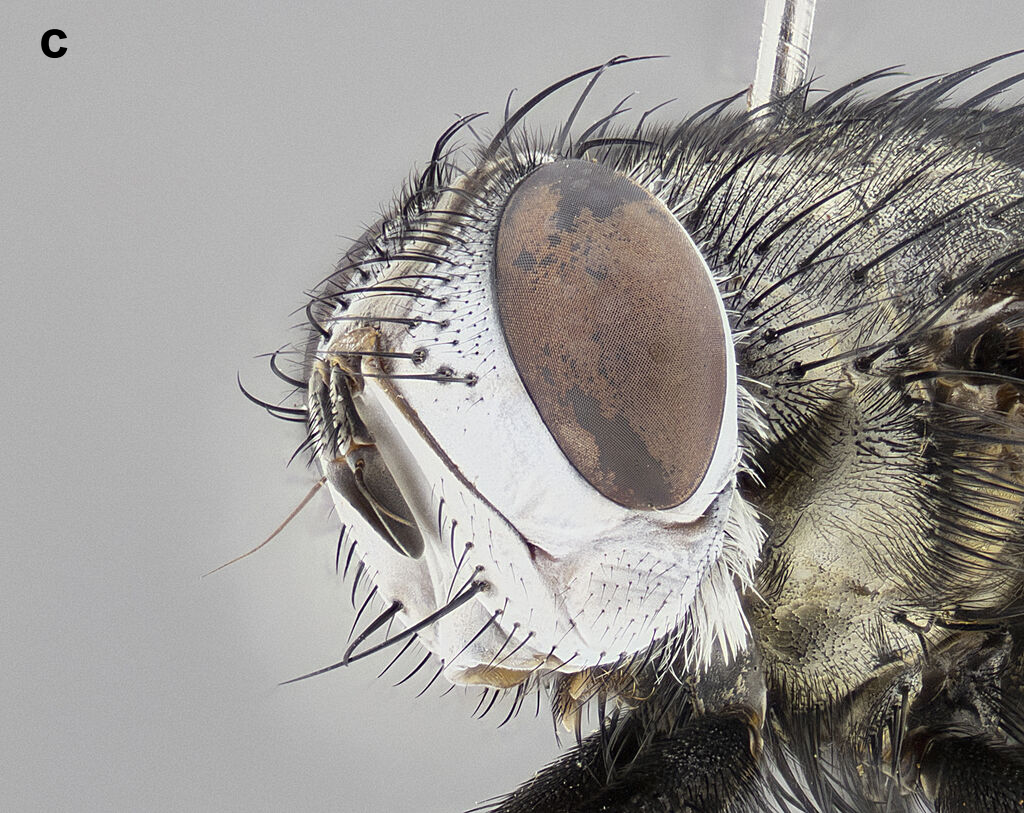
three quarters view

**Figure 90d.**
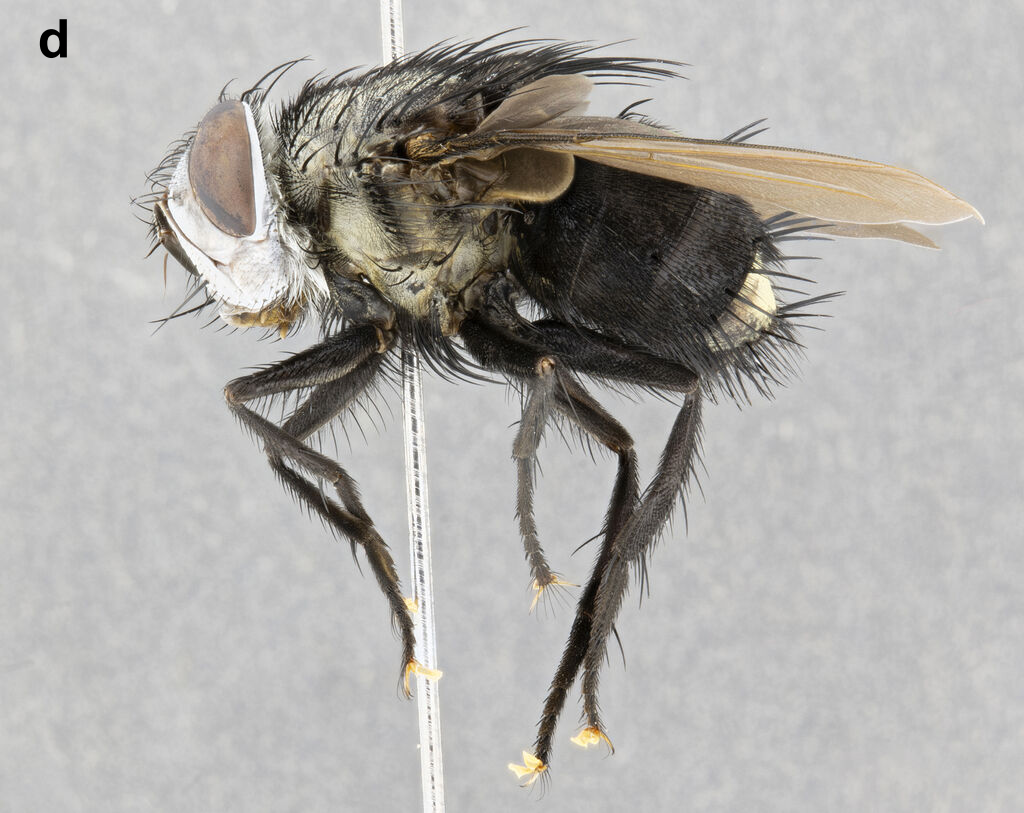
lateral view

**Figure 91a.**
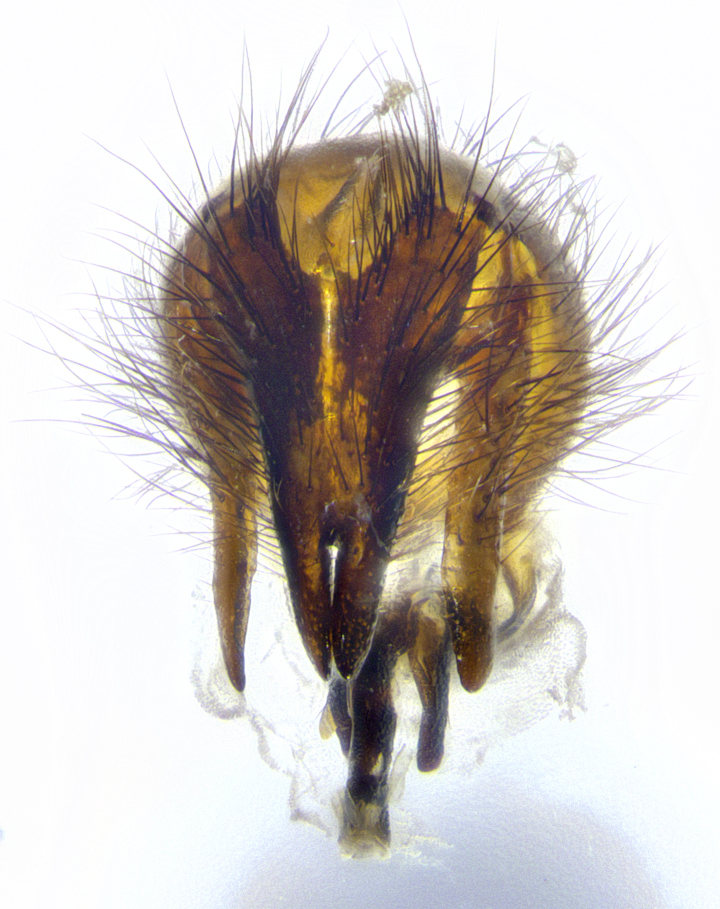
caudal view

**Figure 91b.**
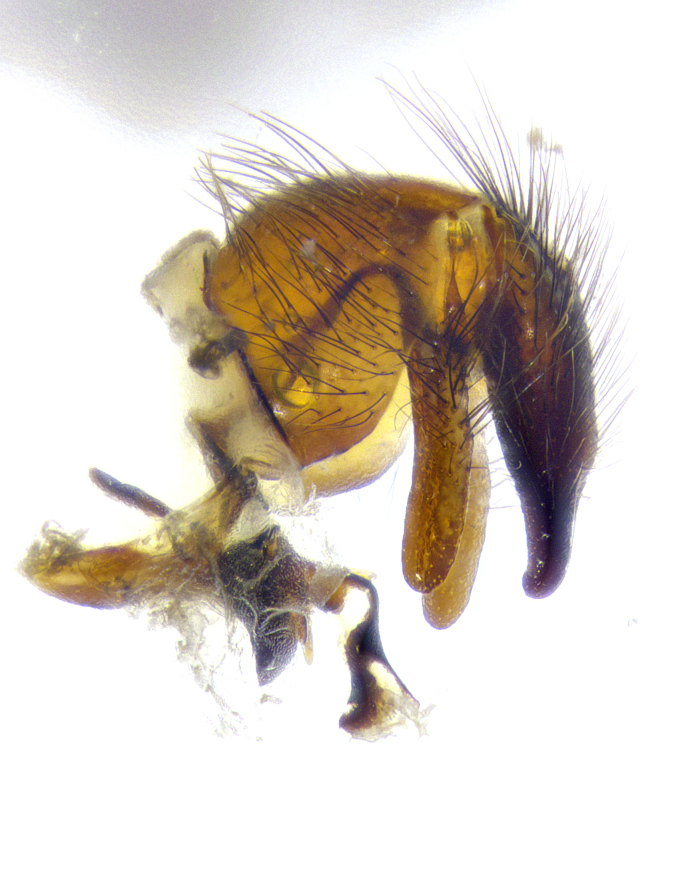
lateral view

**Figure 91c.**
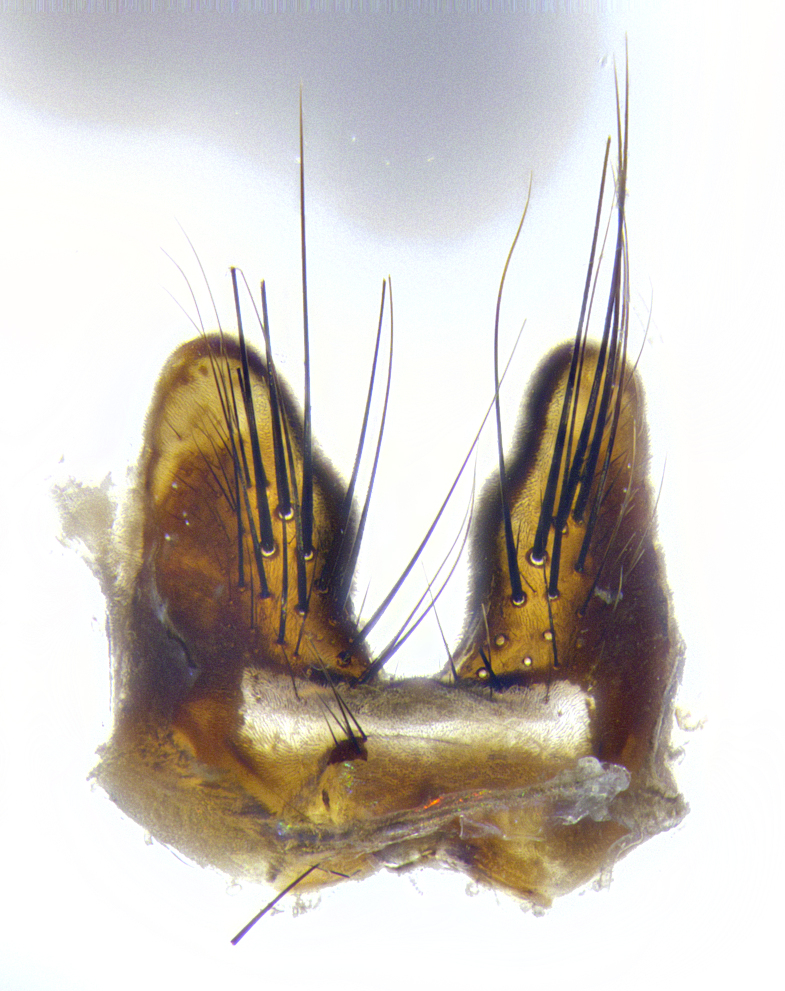
sternite 5, ventral view

**Figure 92a.**
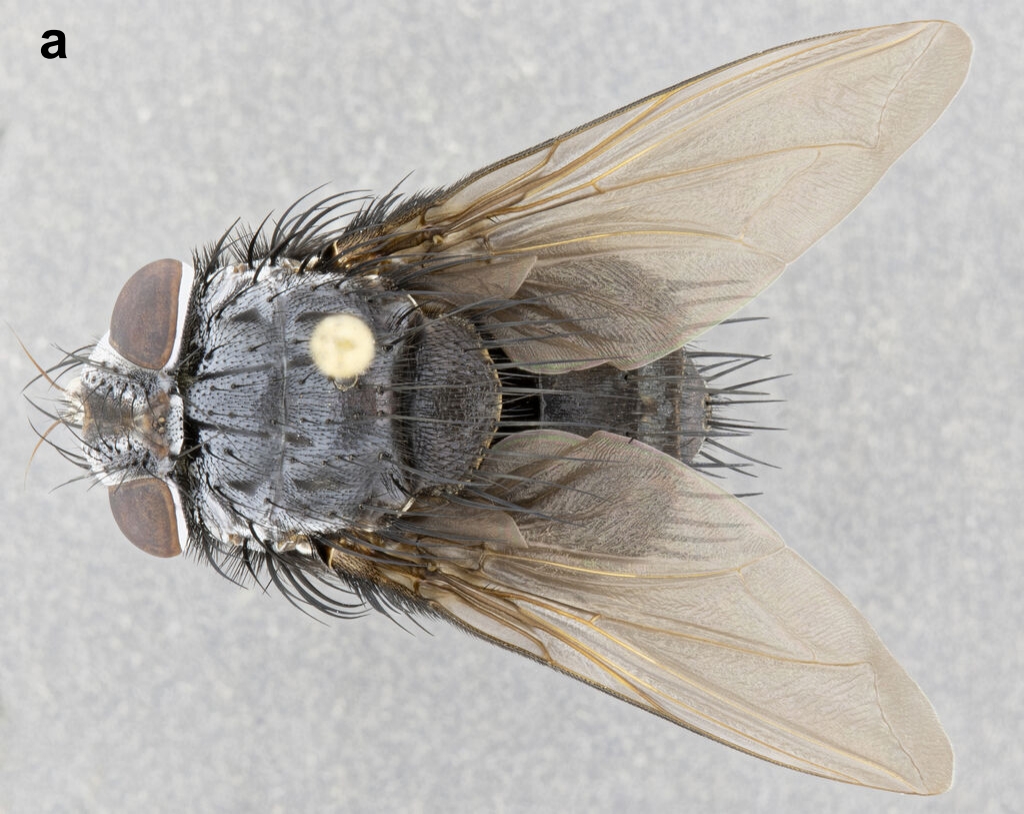
dorsal view

**Figure 92b.**
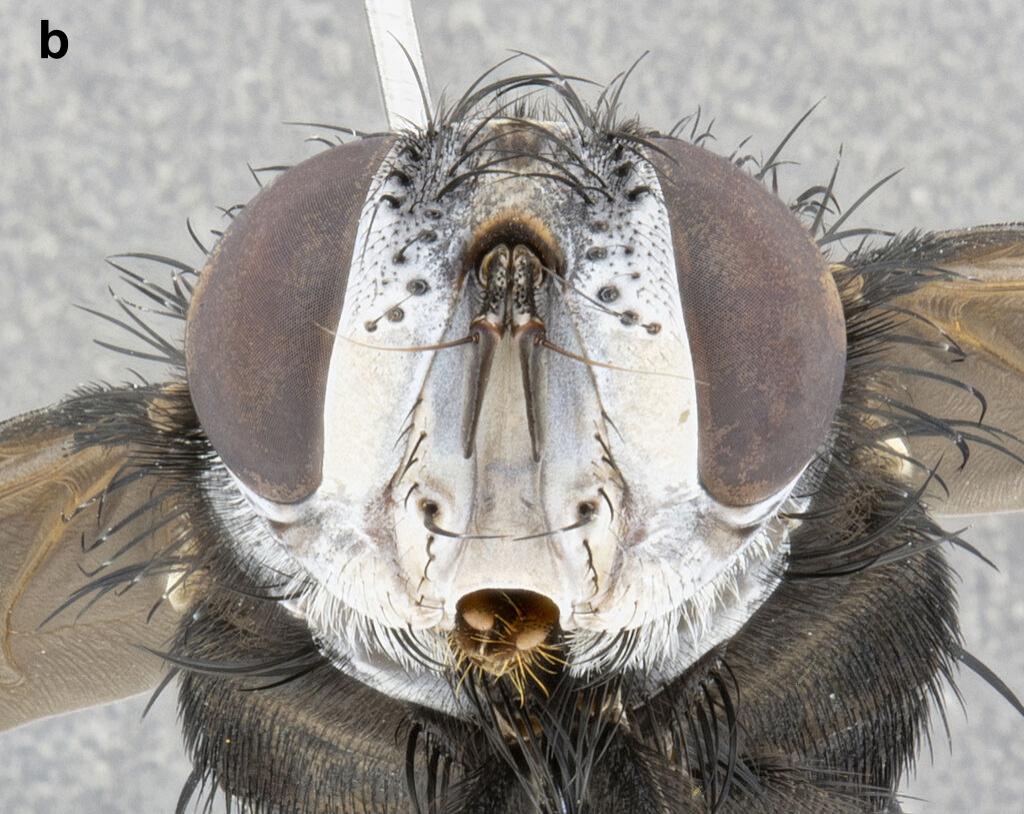
frontal view

**Figure 92c.**
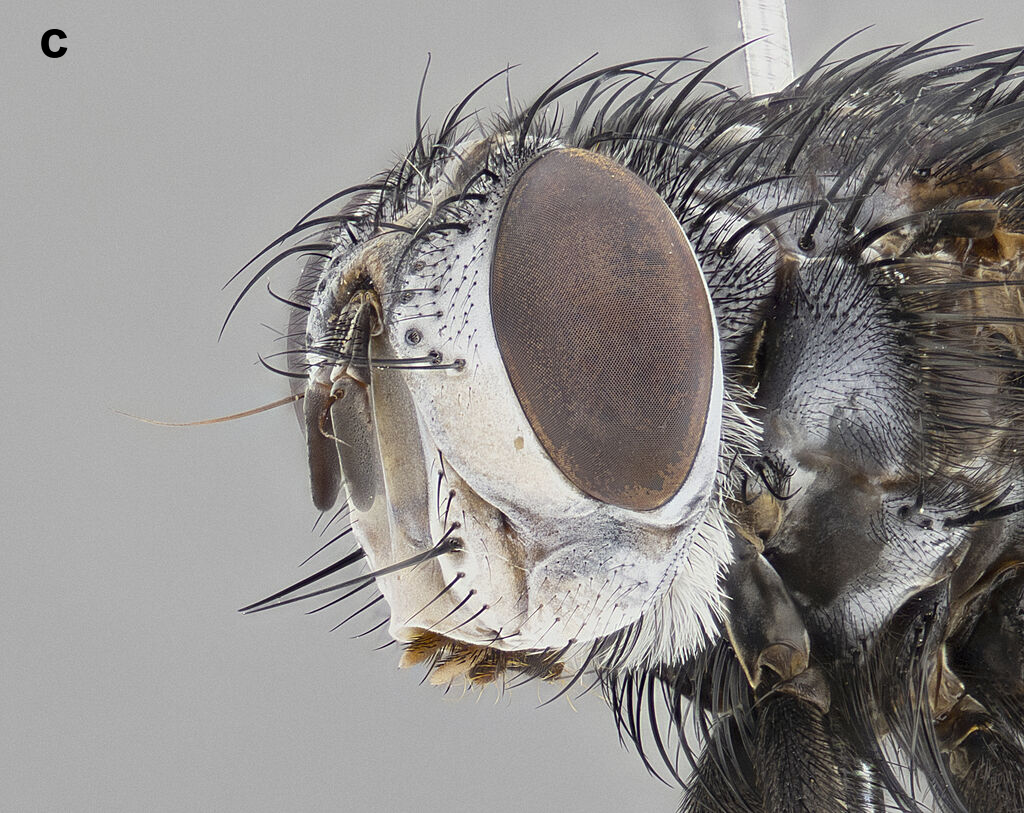
three quarters view

**Figure 92d.**
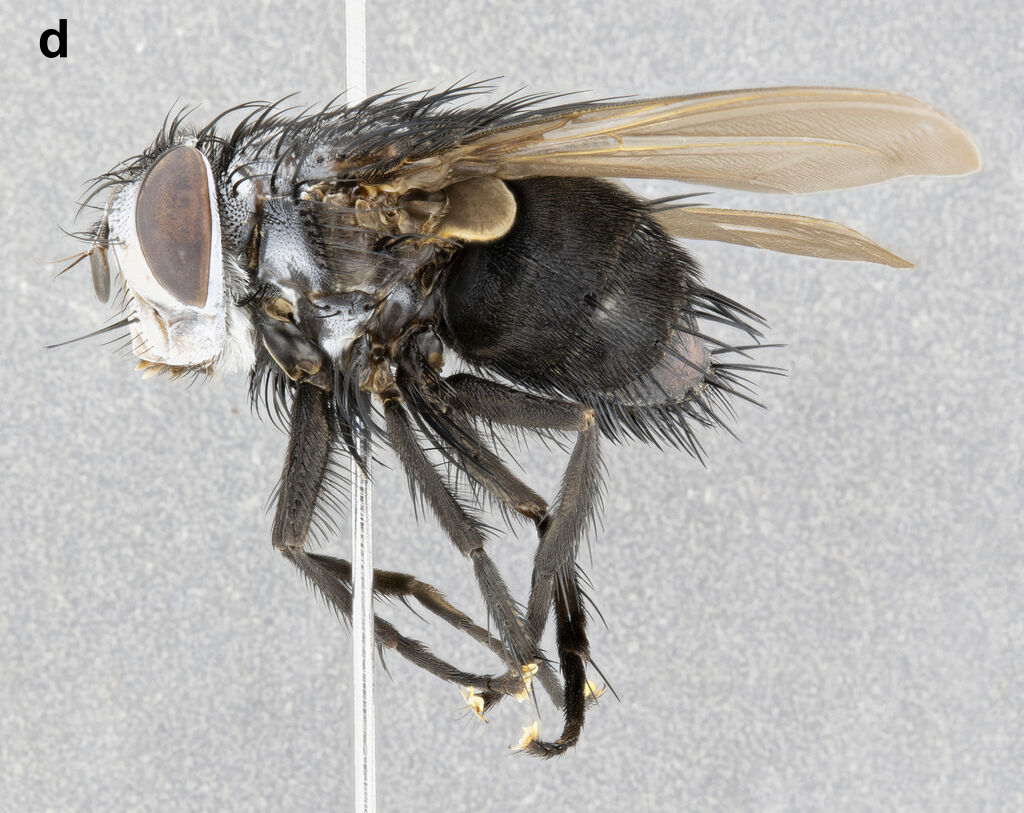
lateral view

**Figure 93a.**
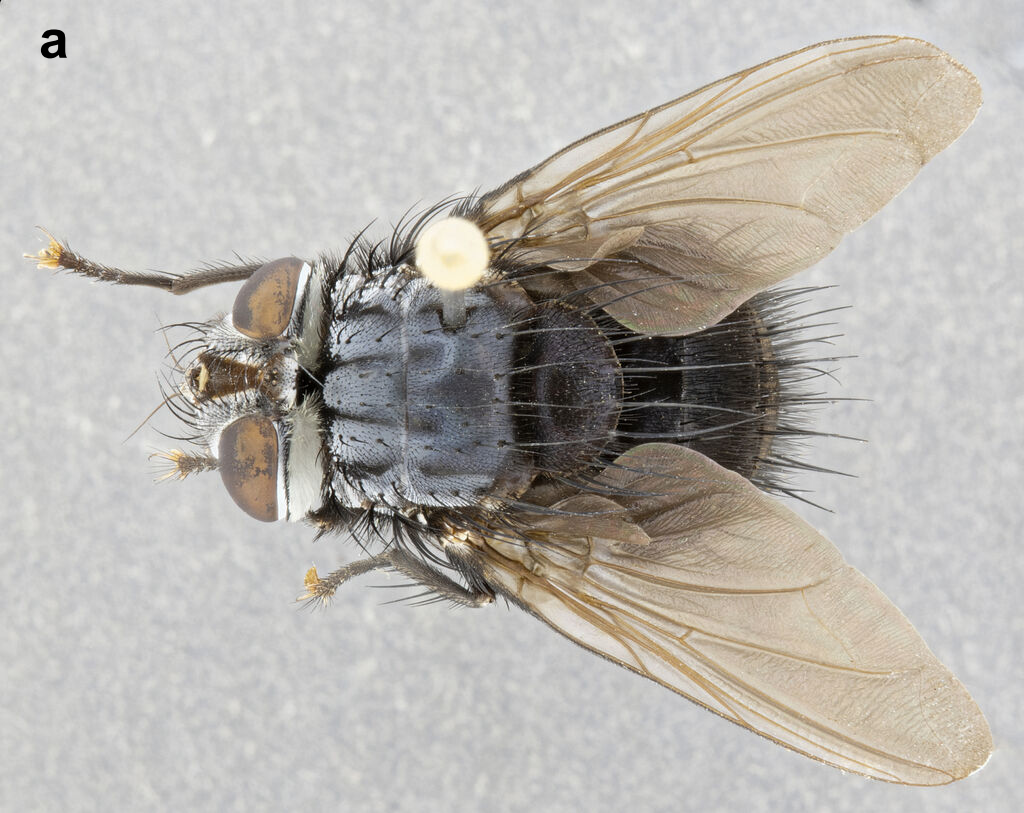
dorsal view

**Figure 93b.**
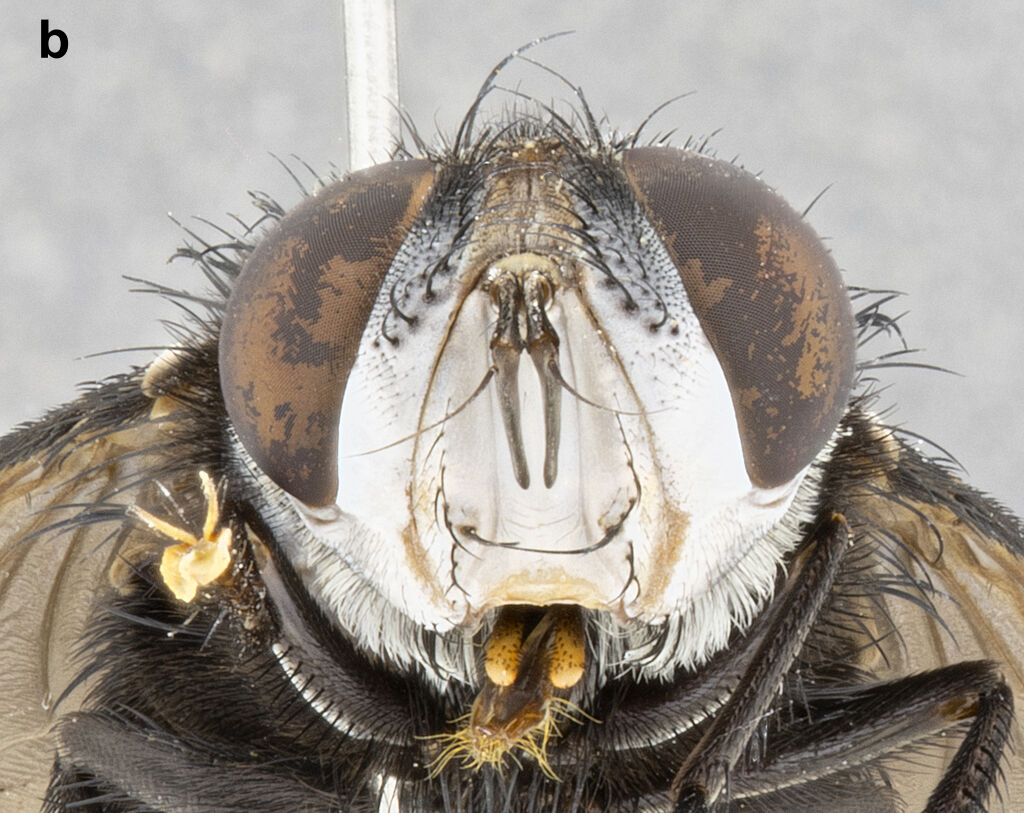
frontal view

**Figure 93c.**
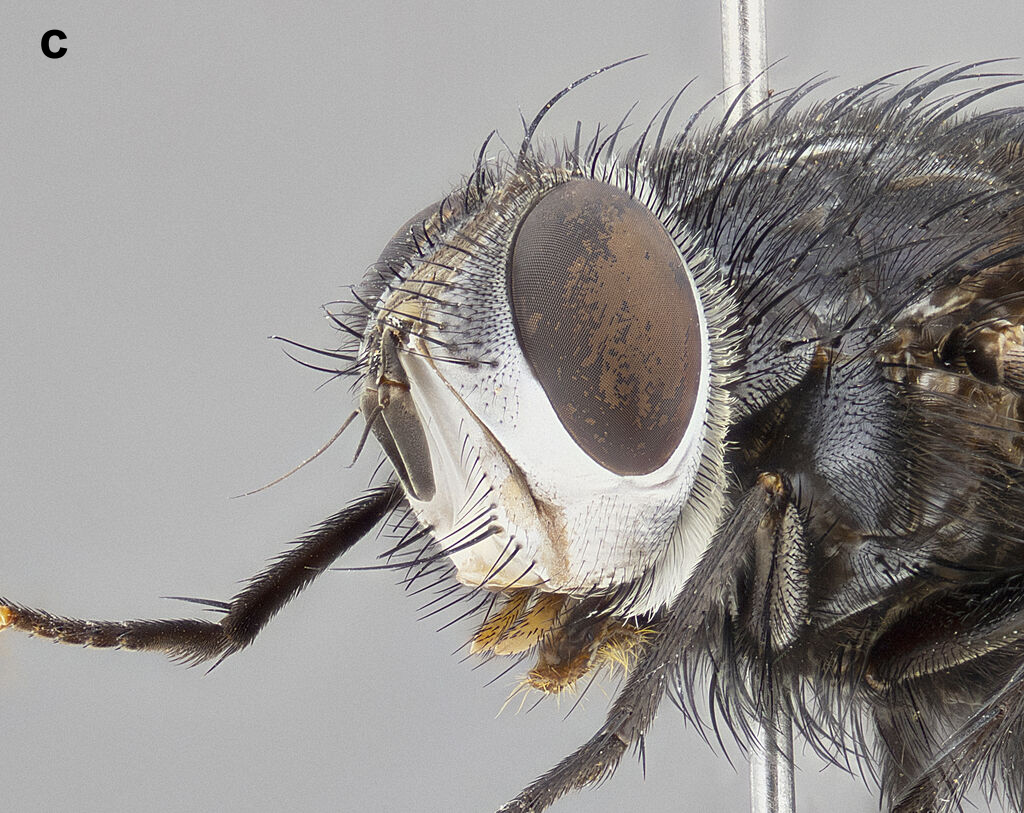
three quarters view

**Figure 93d.**
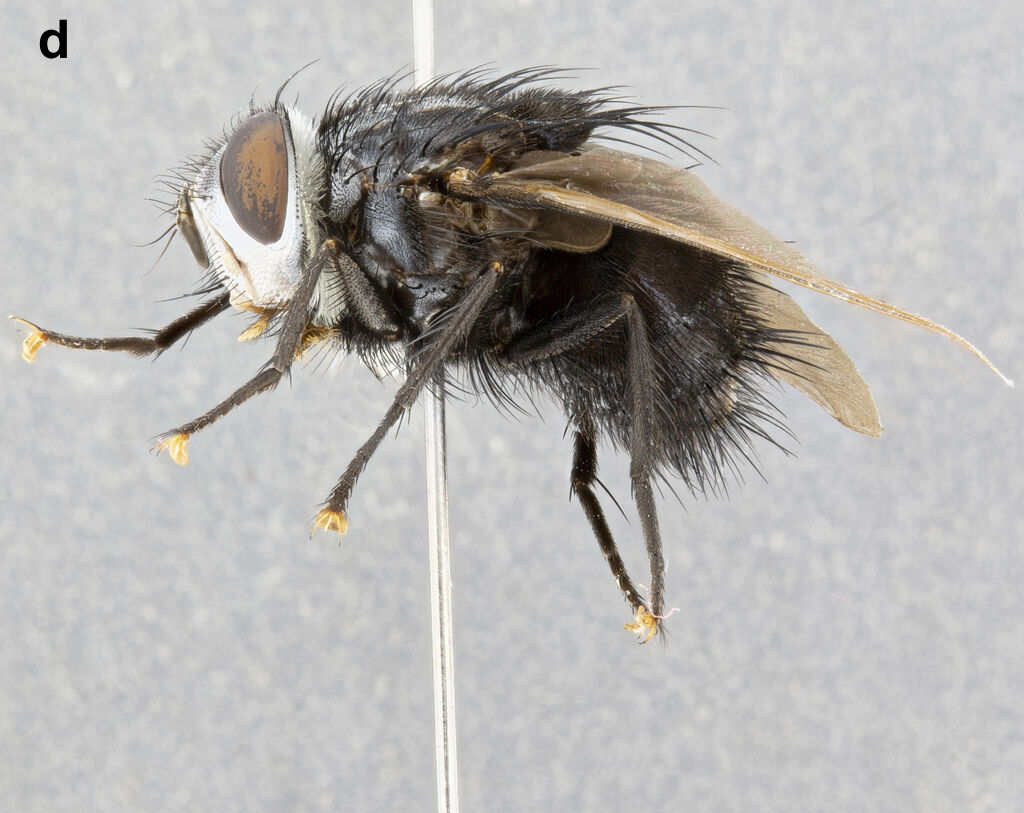
lateral view

**Figure 94a.**
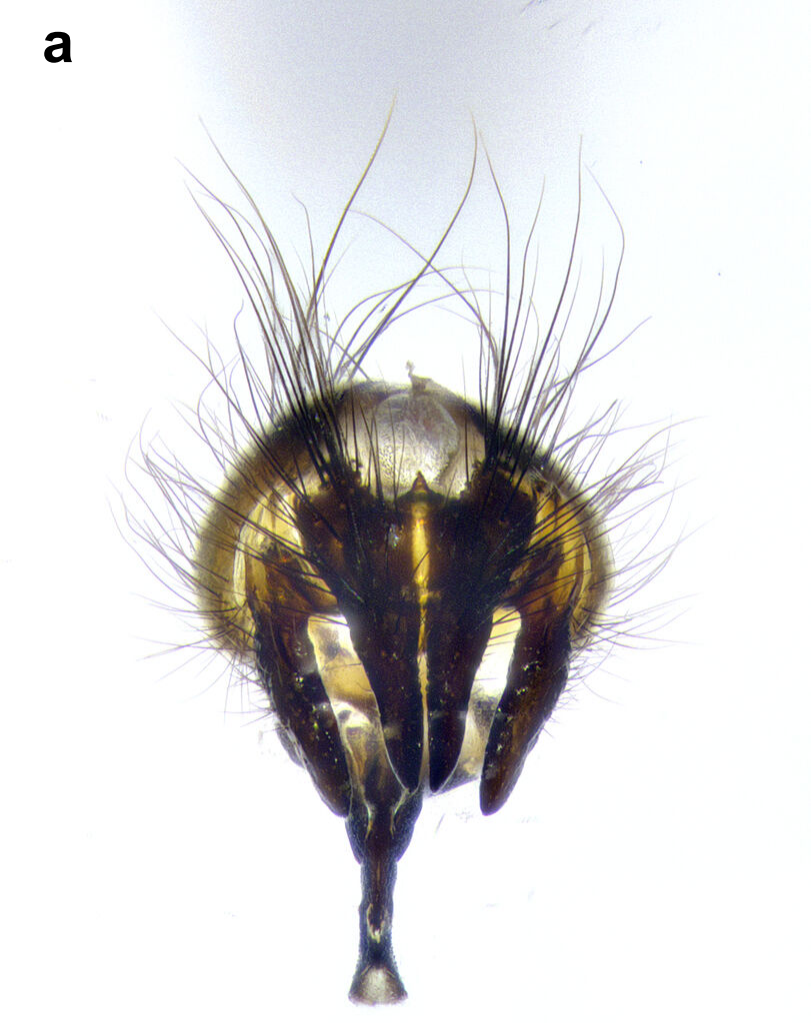
caudal view

**Figure 94b.**
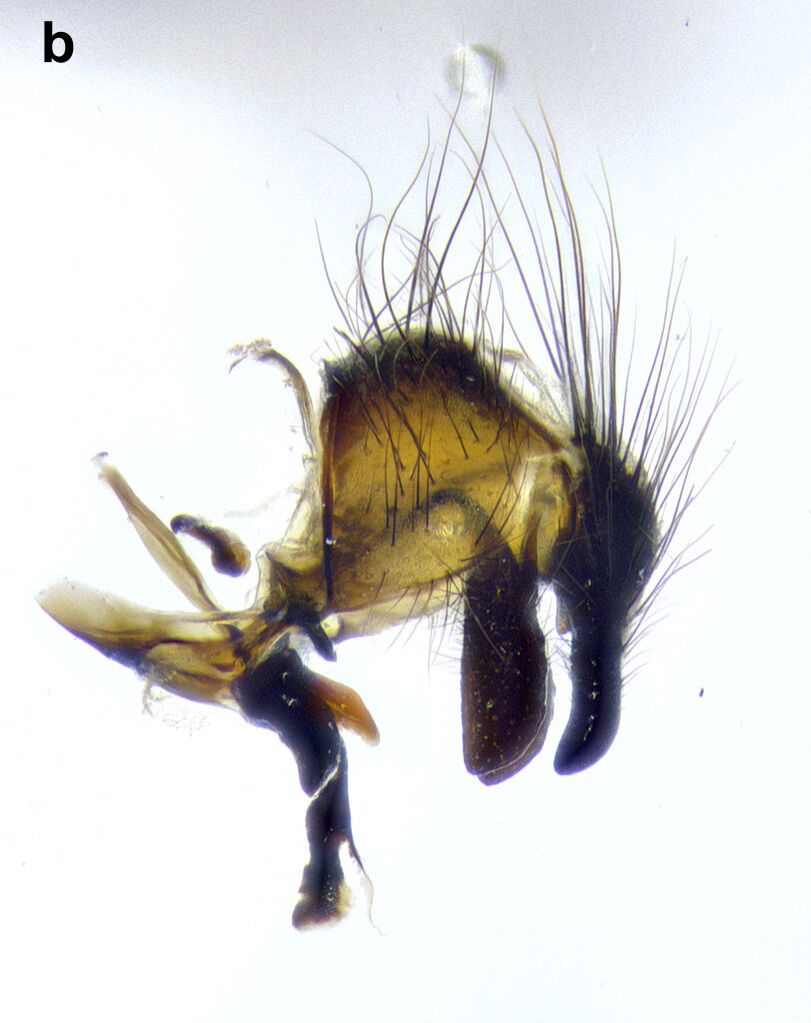
lateral view

**Figure 94c.**
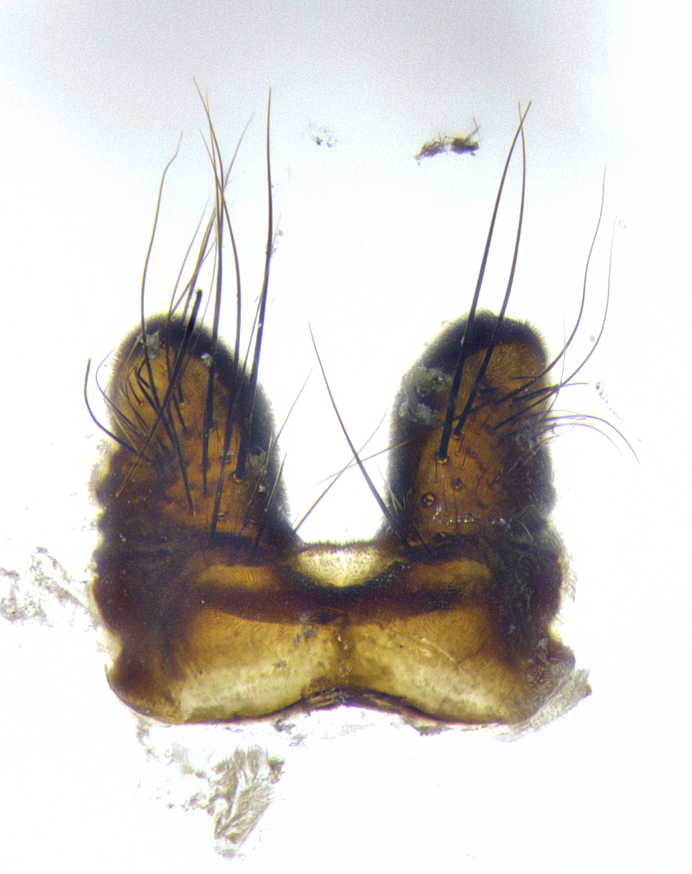
sternite 5, ventral view

**Figure 95a.**
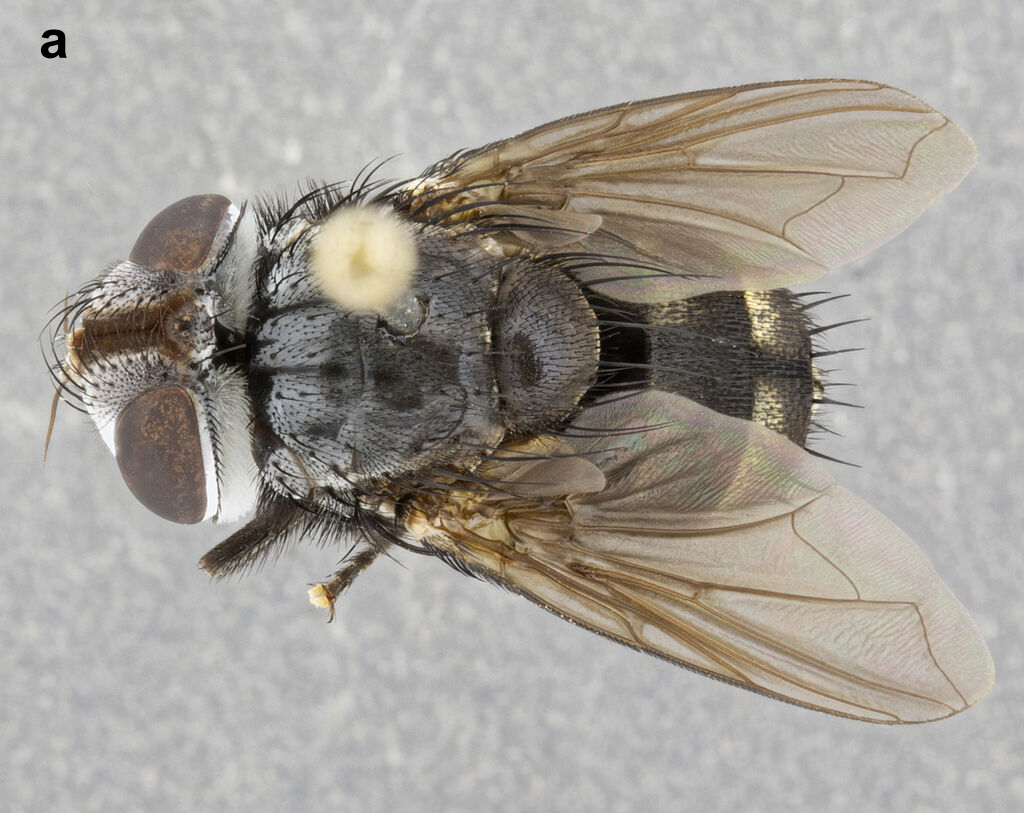
dorsal view

**Figure 95b.**
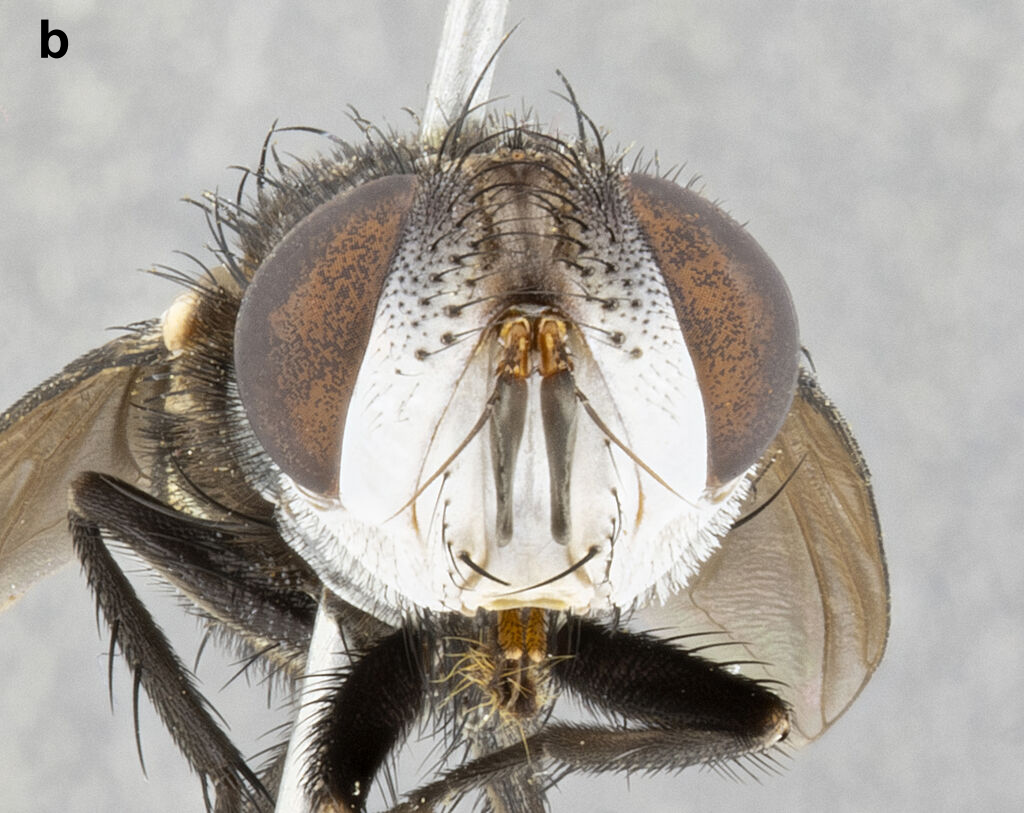
frontal view

**Figure 95c.**
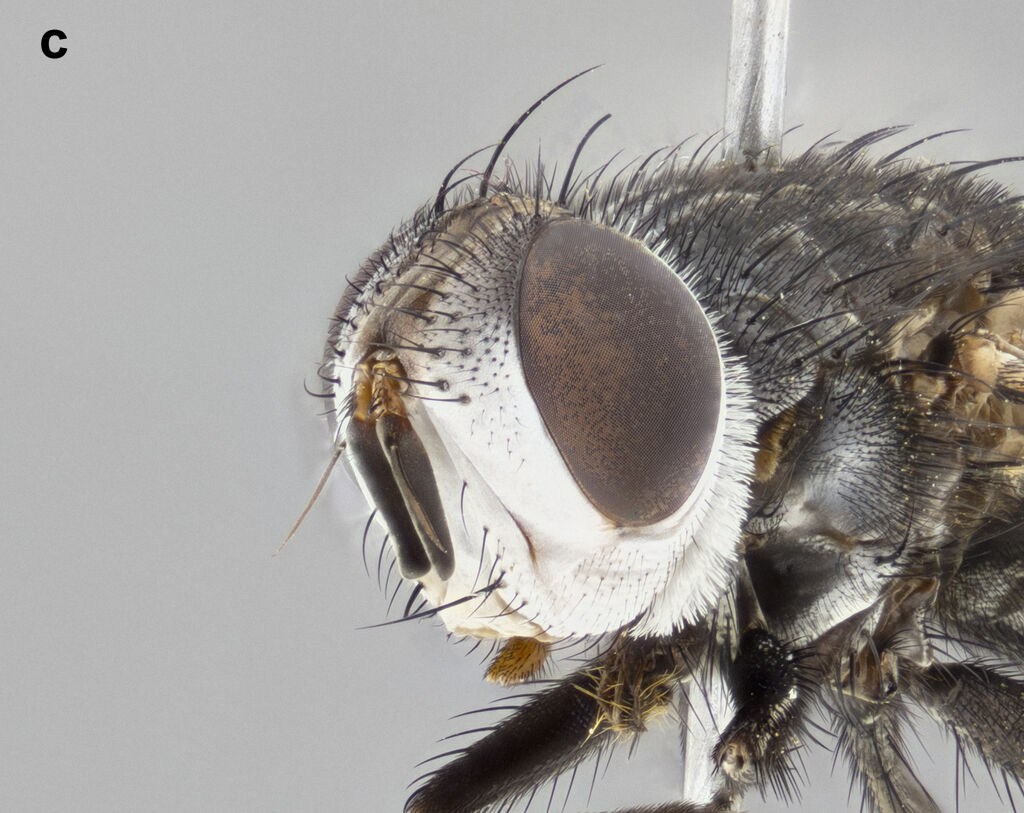
three quarters view

**Figure 95d.**
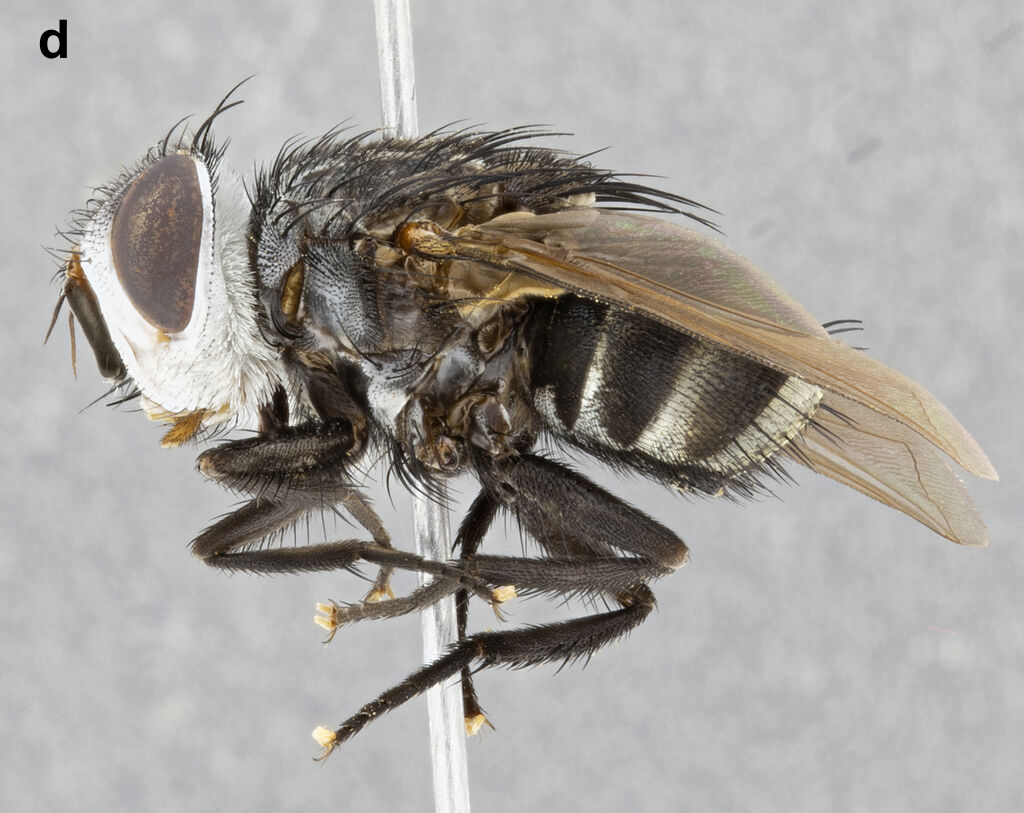
lateral view

**Figure 96a.**
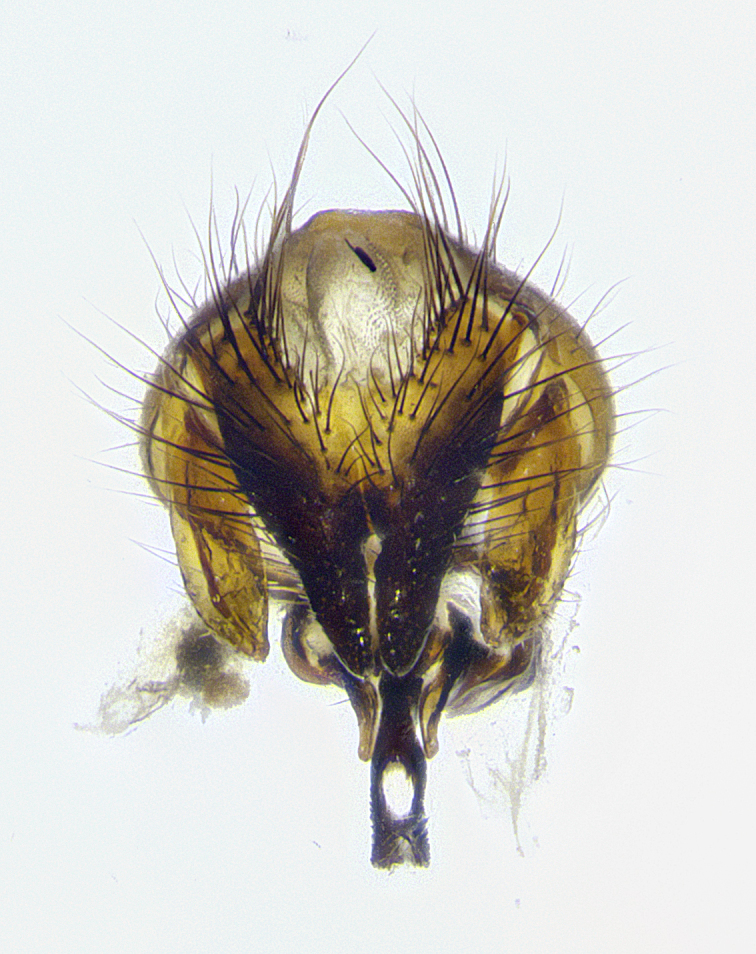
caudal view

**Figure 96b.**
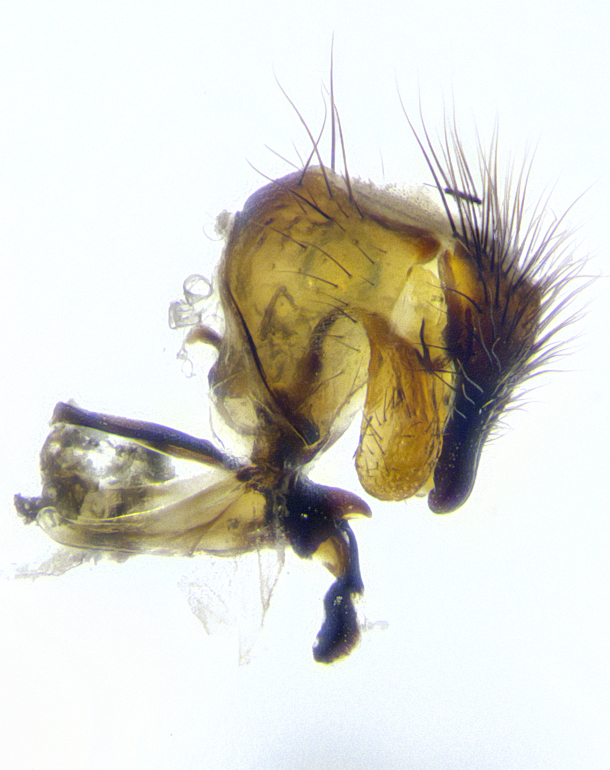
lateral view

**Figure 96c.**
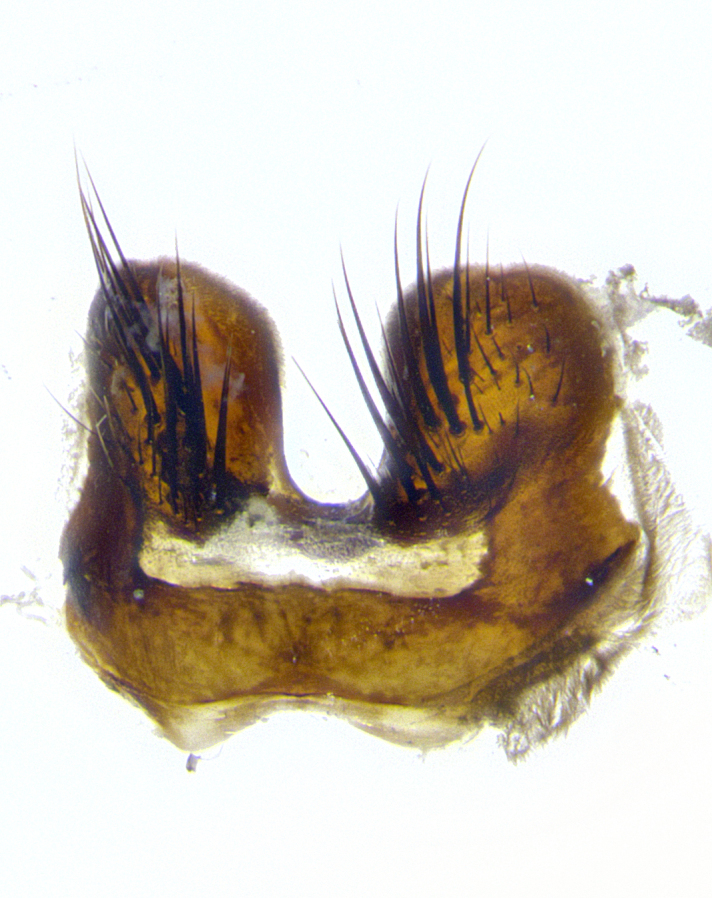
sternite 5, ventral view

**Figure 97a.**
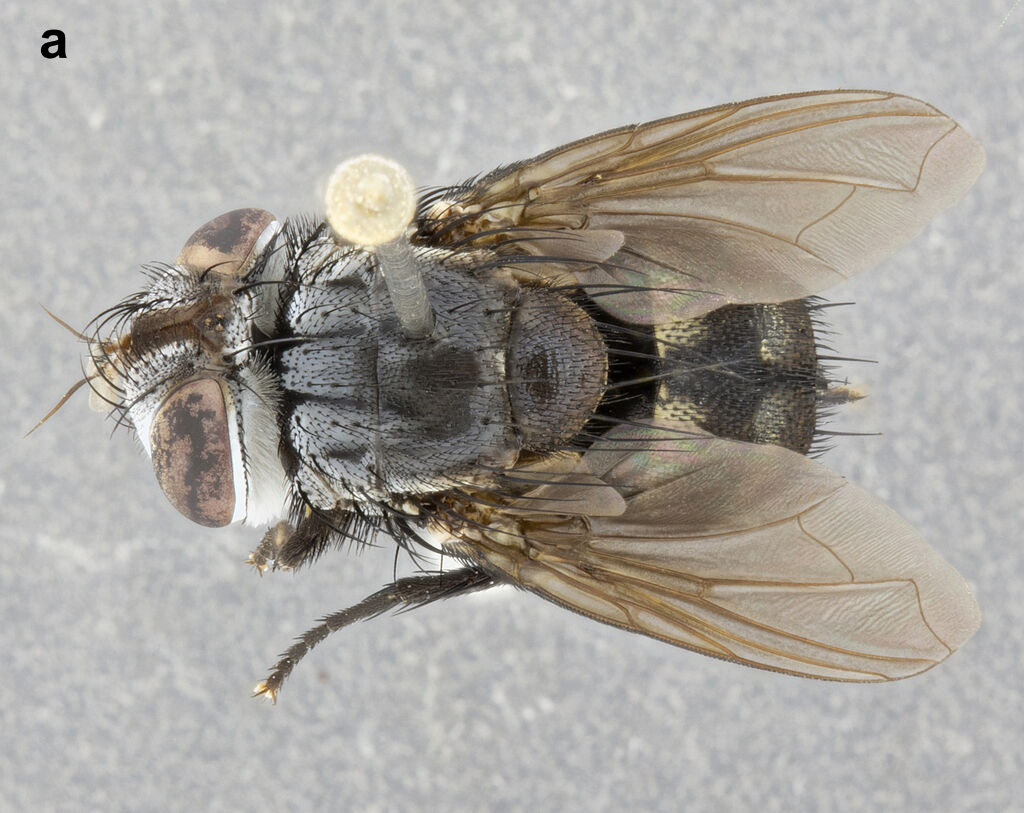
dorsal view

**Figure 97b.**
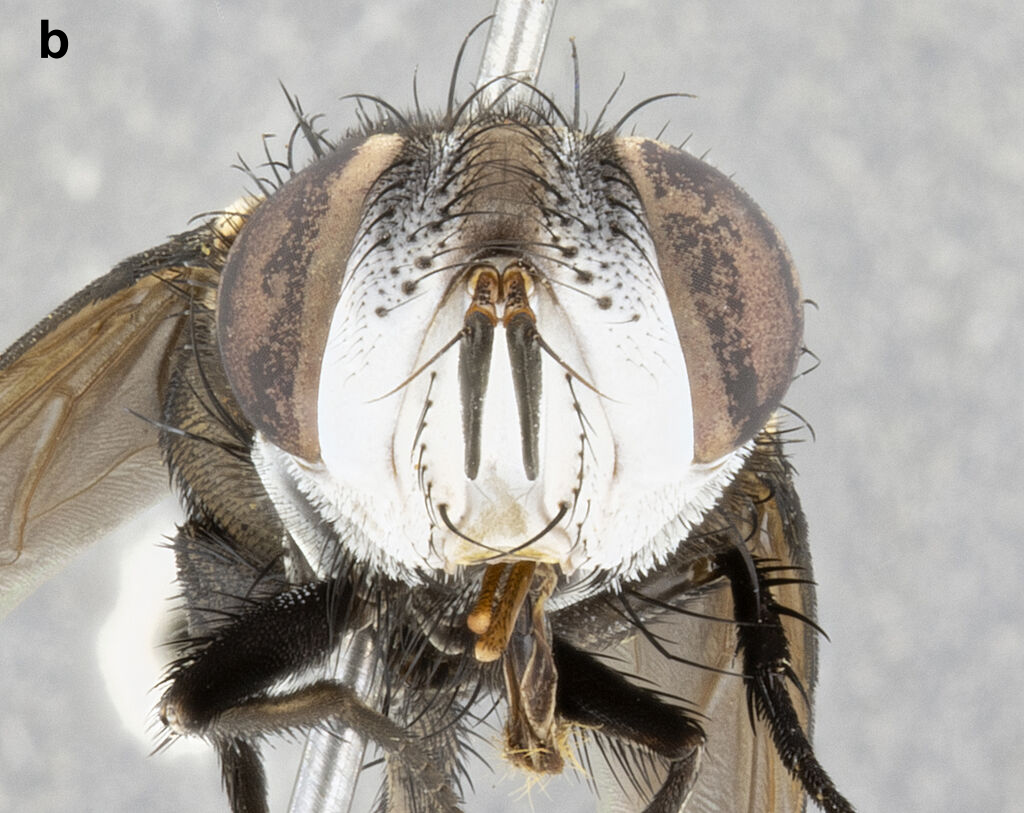
frontal view

**Figure 97c.**
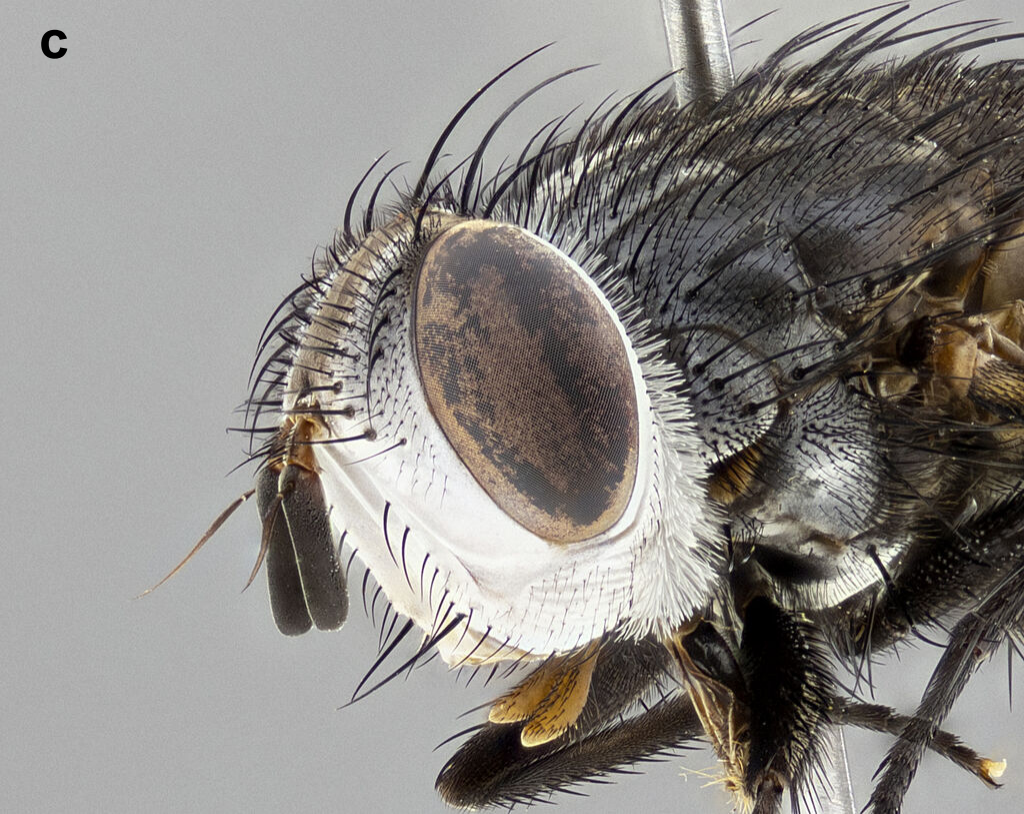
three quarters view

**Figure 97d.**
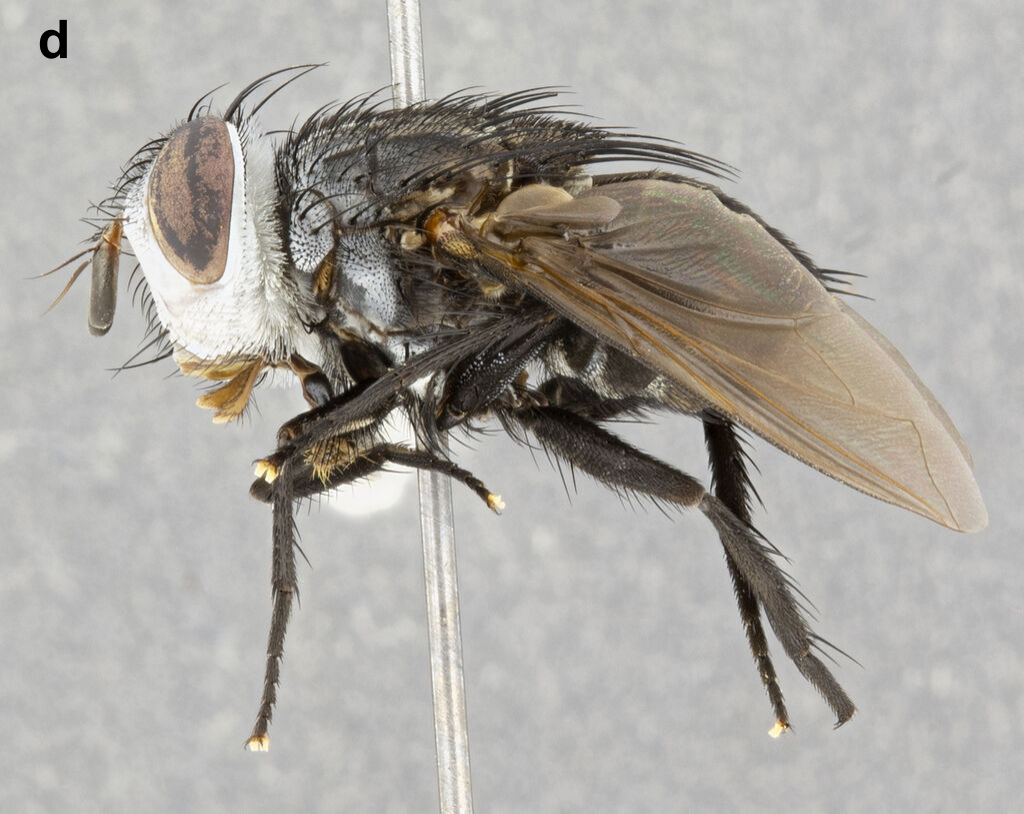
lateral view

**Figure 98a.**
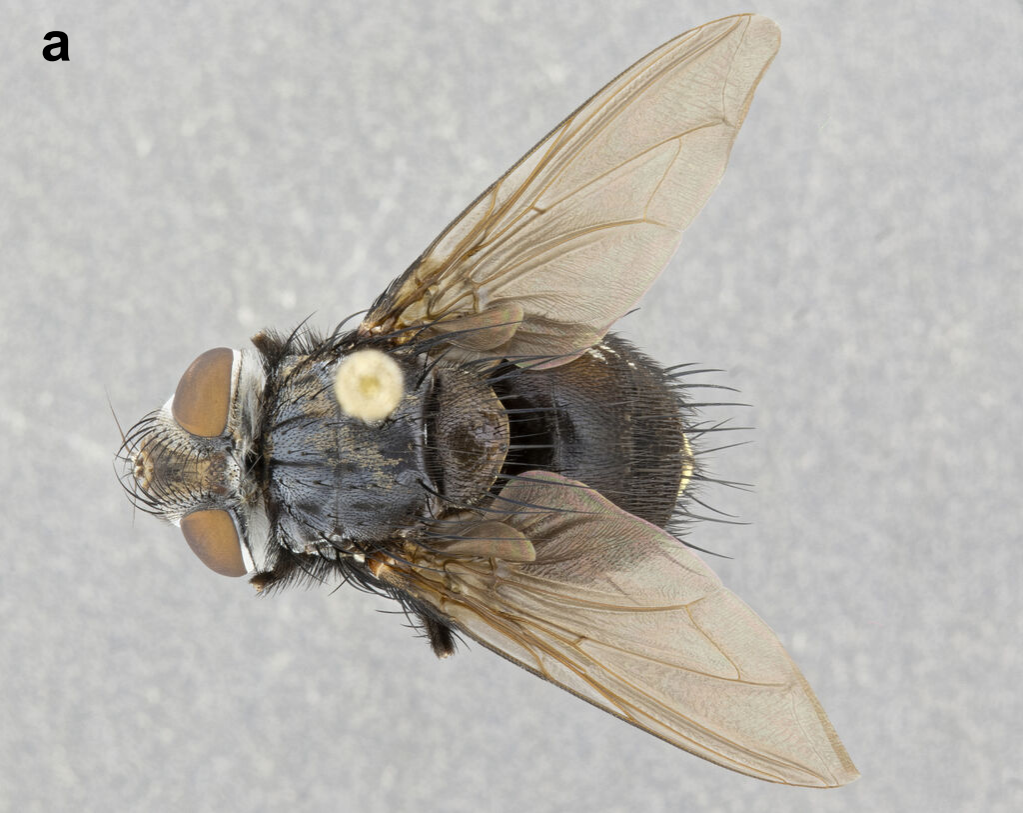
dorsal view

**Figure 98b.**
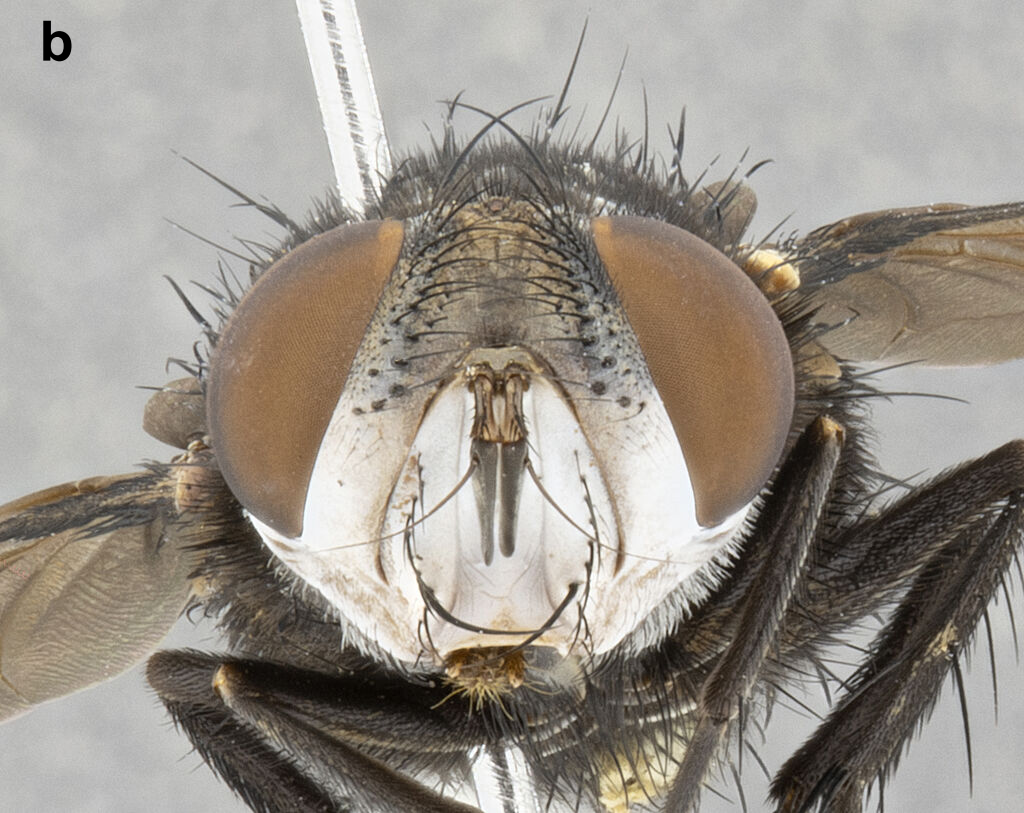
frontal view

**Figure 98c.**
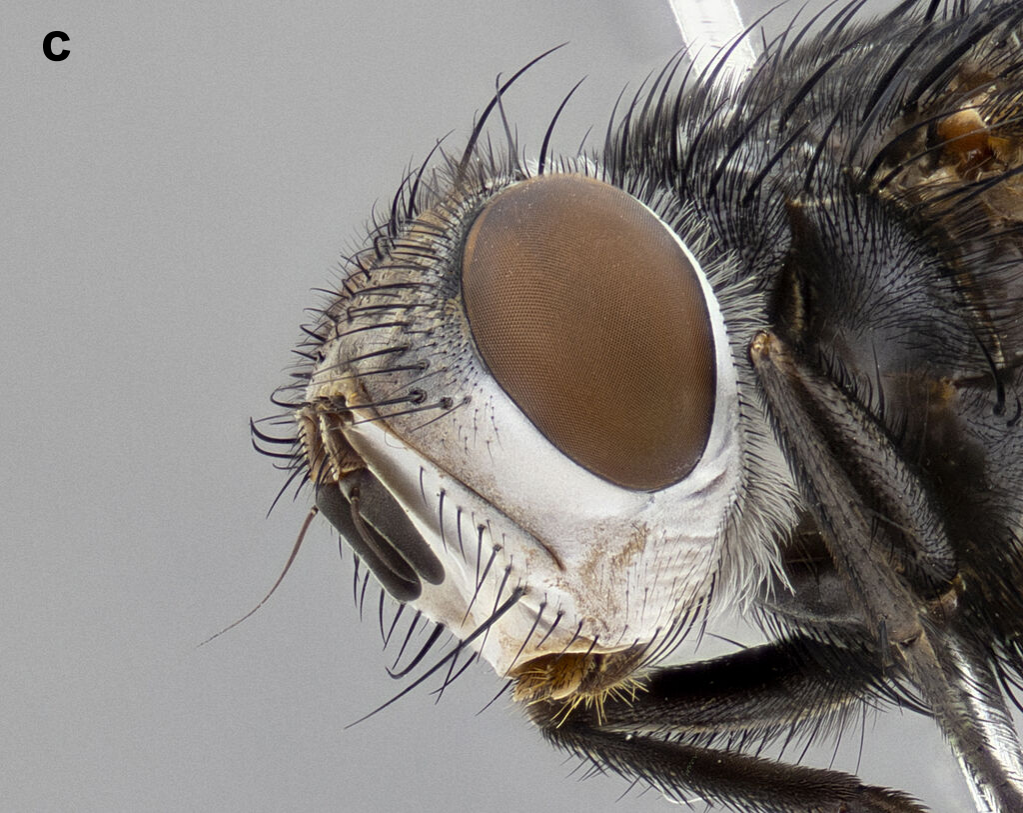
three quarters view

**Figure 98d.**
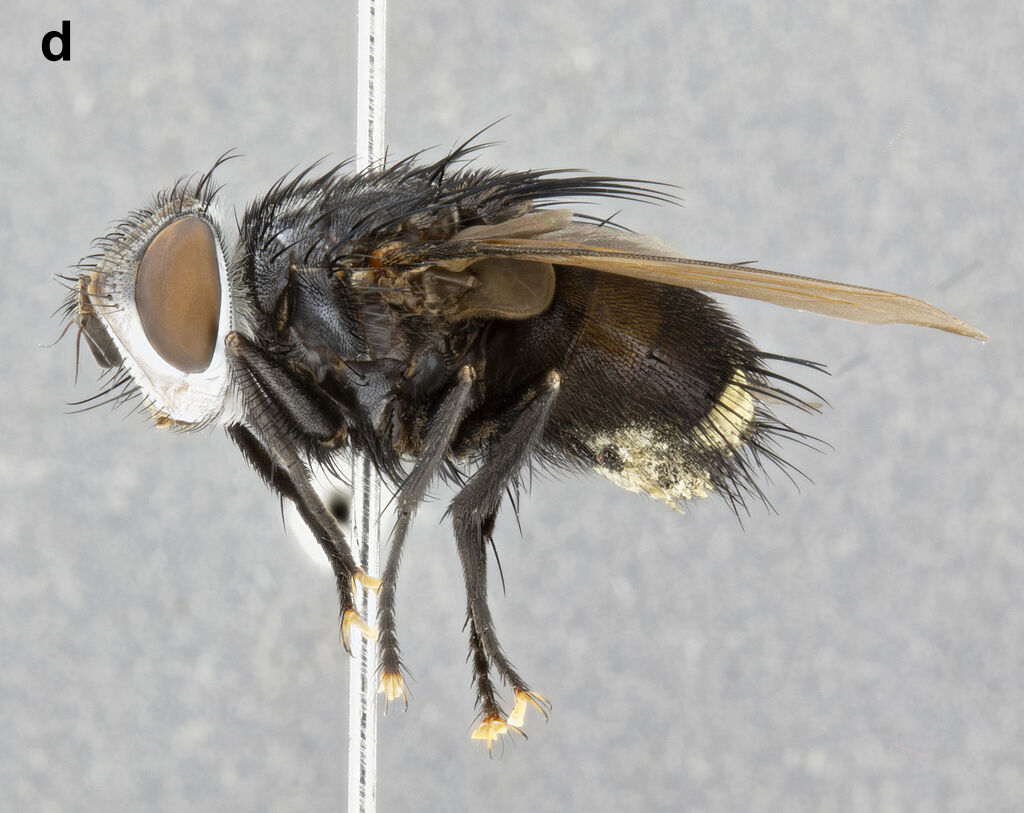
lateral view

**Figure 99a.**
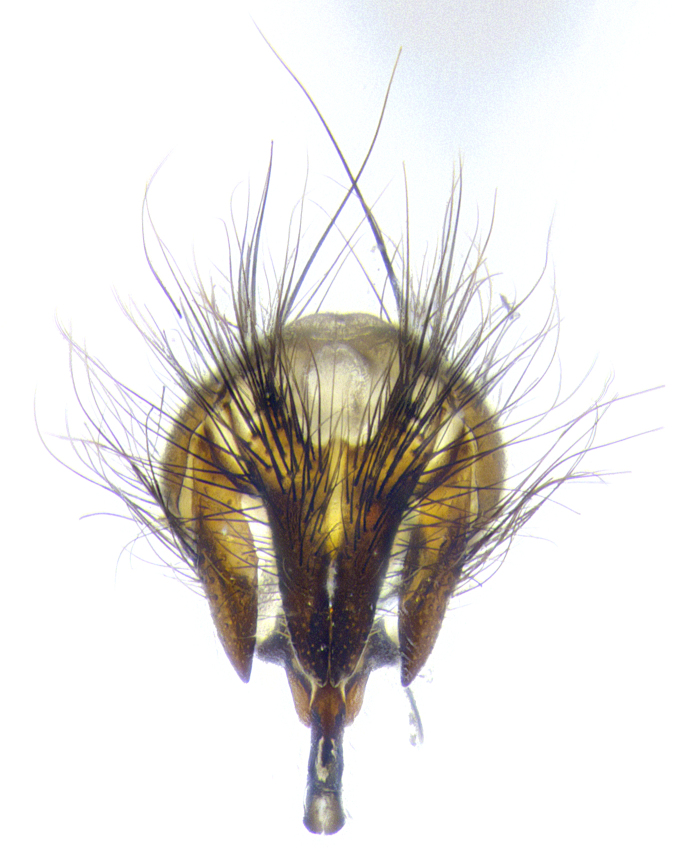
caudal view

**Figure 99b.**
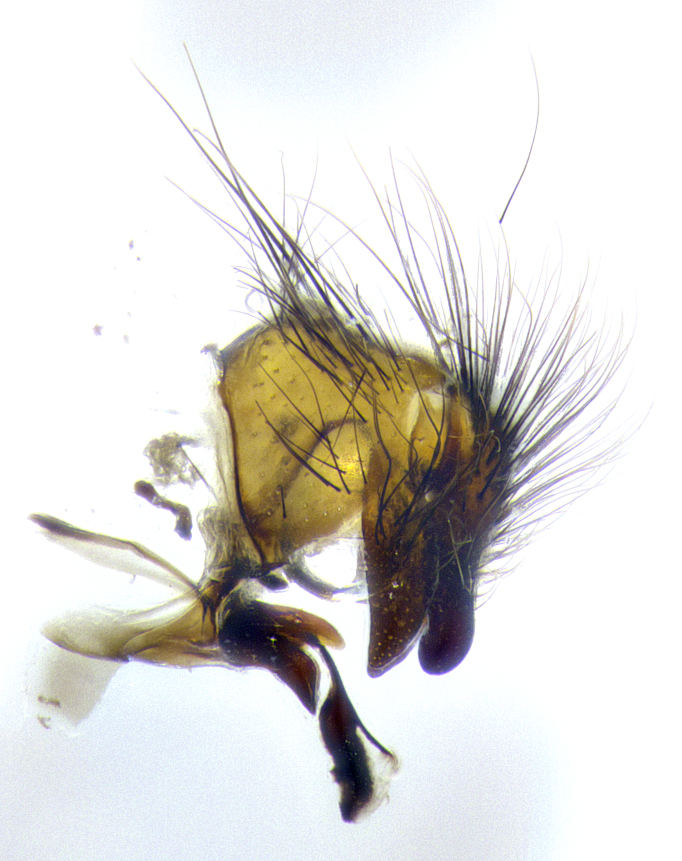
lateral view

**Figure 99c.**
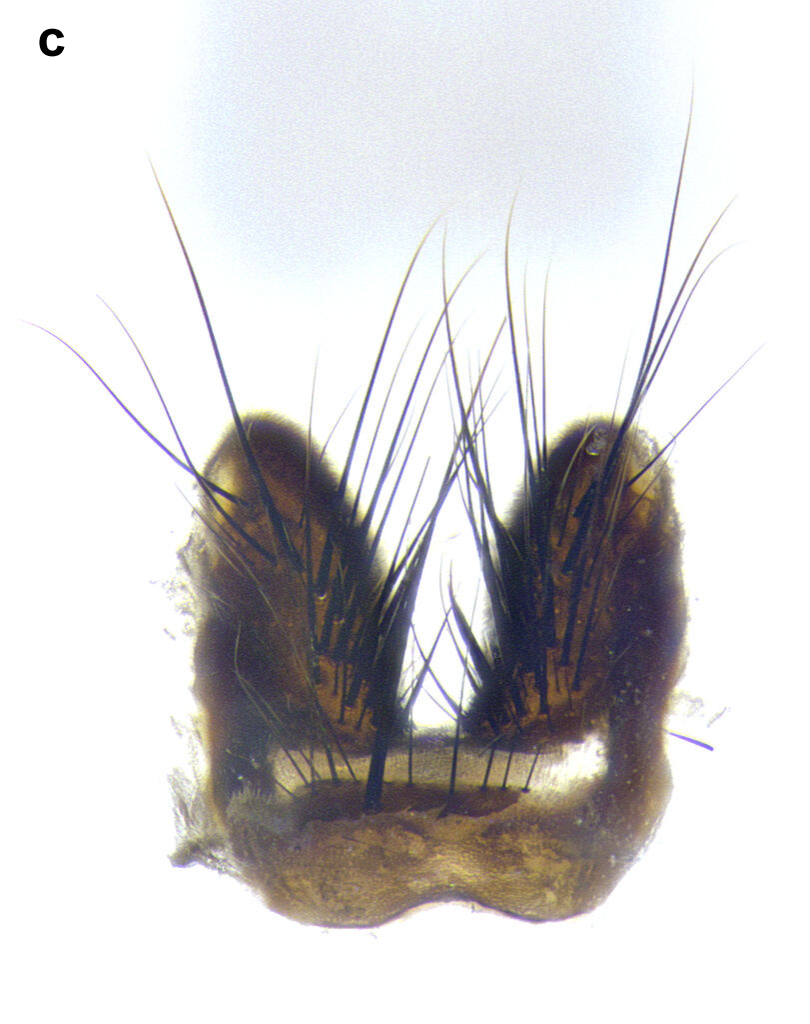
sternite 5, ventral view
